# Synthesis and anti-mycobacterial activity of novel medium-chain β-lactone derivatives: a multi-target strategy to combat *Mycobacterium abscessus*†

**DOI:** 10.1039/d5md00102a

**Published:** 2025-04-25

**Authors:** Thomas Francis, Christina Dedaki, Phoebe Ananida-Dasenaki, Dimitra Bolka, Kanellos Albanis, Filippos Foteinakis, Julie Mezquida, Marie Hance, Alexandros Athanasoulis, Anna-Krinio Papagiorgou, Ioanna-Foteini Karampoula, George Georgitsis, Celia Jardin, Stéphane Audebert, Luc Camoin, Céline Crauste, Stéphane Canaan, Victoria Magrioti, Jean-François Cavalier

**Affiliations:** a Aix-Marseille Univ., CNRS, LISM, Institut de Microbiologie de la Méditerranée FR3479 Marseille France jfcavalier@imm.cnrs.fr; b Department of Chemistry, National and Kapodistrian University of Athens Panepistimiopolis Athens 15771 Greece vmagriot@chem.uoa.gr; c Aix-Marseille Univ., INSERM, CNRS, Institut Paoli-Calmettes, CRCM, Marseille Protéomique Marseille France; d IBMM, Univ Montpellier, CNRS, ENSCM Montpellier France

## Abstract

The constant emergence of drug-resistant mycobacteria, together with the lack of new antibiotics entering the market, has become a global public health problem that threatens the effective treatment of infectious diseases. The development of single molecules targeting different proteins should significantly reduce the emergence of resistant strains, and therefore represent a promising strategy to overcome such an issue. In this challenging context, a new series of 30 lipophilic compounds based on the β-lactone-core has been synthesized by varying the nature of the substituents on the lactone ring. The evaluation of their antibacterial activity against *M. tuberculosis* and *M. abscessus*, two major pathogenic mycobacteria, highlighted potential candidates. The VM038, VM040 and VM045 were active only against *M. tuberculosis*, while VM025, VM026 and VM043 inhibited the growth of both *M. tuberculosis* and the S and R variants of *M. abscessus*. Competitive click chemistry activity-based protein profiling revealed several potential *M. abscessus* target enzymes of VM043, the best extracellular growth inhibitor. Finally, when tested against intracellular bacteria, although VM043 was found inactive, VM025 & VM026 proved to be potent and promising inhibitors of intramacrophagic *M. abscessus* growth with minimal inhibitory concentrations (MIC_50Raw_) comparable to the standard antibiotic imipenem. Overall, these results strengthen the added value of our VM β-lactone derivatives not only in the fight against pathogenic mycobacteria, leading to the arrest of *M. abscessus* and/or *M. tuberculosis* growth through multitarget enzyme inhibition, but also as efficient probes to identify novel potential therapeutic targets using chemoproteomics approaches.

## Abstract 2

Novel lipophilic β-lactone VM derivatives inhibit the extracellular and/or intracellular growth of *M. abscessus*, while being nontoxic to host macrophages.

## Graphical abstract

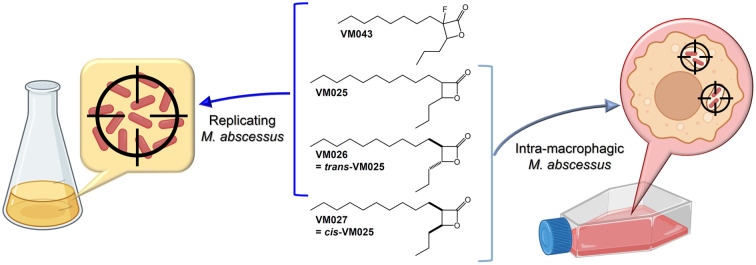

## 1. Introduction

The *Mycobacterium* genus consists of more than 200 species, which are mainly classified according to their pathogenicity and growth rate.^1,2^ In addition to *Mycobacterium tuberculosis*, the causative agent of tuberculosis (TB),^3,4^ nontuberculous mycobacteria (NTM) are opportunistic pathogens responsible for clinical syndromes ranging from skin to pulmonary infections (*e.g.*, *Mycobacterium abscessus*^5–7^) especially in immunocompromised individuals.^8–10^ Furthermore, the emergence of multidrug-resistant isolates of *M. tuberculosis* or *M. abscessus* has significantly reduced treatment success rates, leading to higher incidence of treatment failure and mortality.^6,11–13^ Known as the “antibiotic and clinical nightmare”,^6,14^*M. abscessus* is indeed considered one of the most drug-resistant mycobacterial species.^15^ This mycobacterium exists as two distinct colony morphotypes: smooth (S) and rough (R), which can adapt and develop differently in response to the host immune system therefore leading to different outcomes for the mycobacteria within its host.^7,16,17^ The R variant is particularly associated with severe lung infections^5,12^ and can persist for years, especially in patients with cystic fibrosis (CF).^18^

A key factor limiting the treatment of mycobacterial infections is the presence of a complex, waxy, lipid-rich cell wall containing unique lipids such as mycolic acids.^19,20^ These lipids play a critical role in maintaining the structural integrity of the bacterial cell envelope and in modulating interactions with the host immune system.^20,21^ Moreover, this general composition and architecture is shared by all mycobacterial species contributing to their low permeability to many antibiotics thus limiting therapeutic options.^19,20^ Therefore, targeting the enzymes involved in mycobacterial lipid metabolism;^22,23^ which are mainly serine and cysteine enzymes (*i.e.*, (Ser/Cys)-based enzymes);^24–26^ has emerged as a promising strategy to combat not only *M. tuberculosis*,^27–31^ but also other chronic mycobacterial infections like those caused by *M. abscessus*.^24,32^

**Fig. 1:**
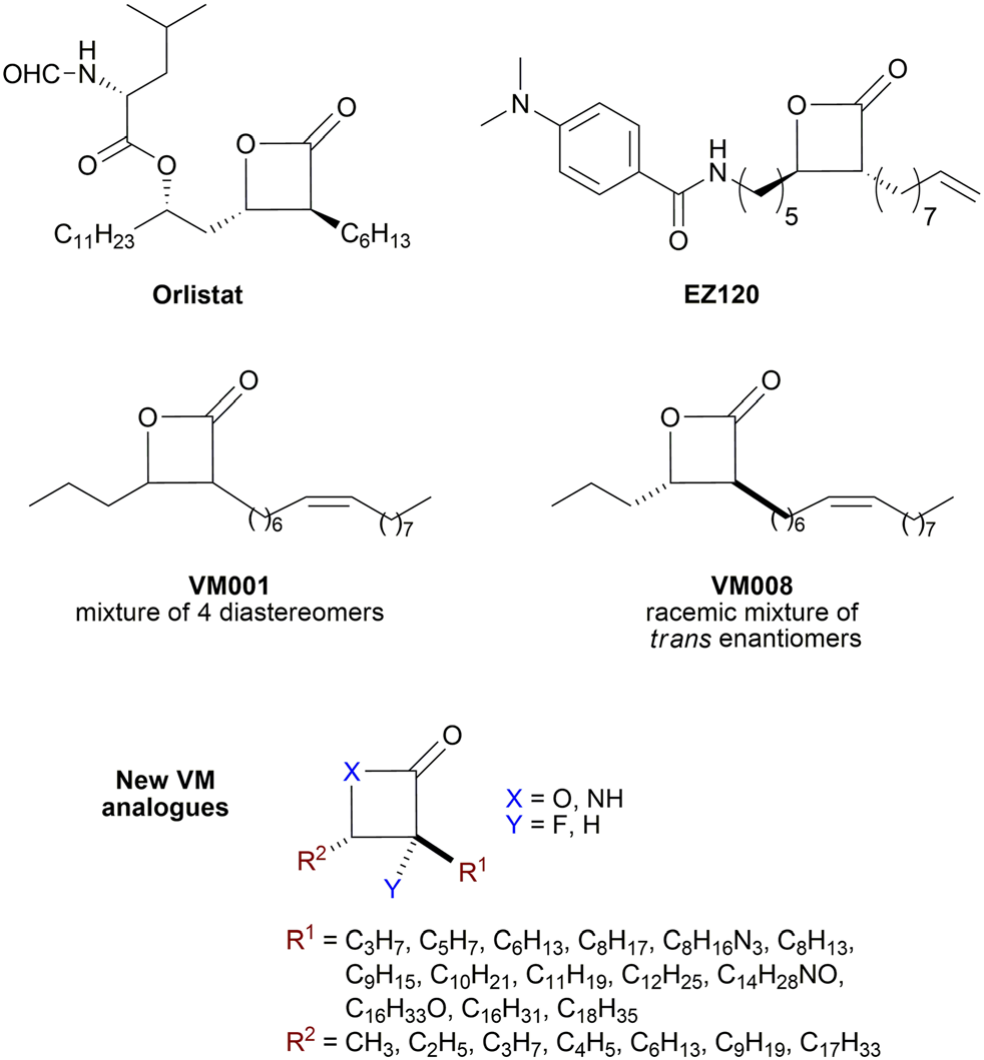
Chemical structures of **Orlistat**,^42^ and EZ120;^47^ former VM001 and VM008 (ref. 45) as well as new synthesized related analogues (see Table S1† for structure details).

β-Lactones are a class of four-membered cyclic esters characterized by a highly strained ring structure that confers significant reactivity and makes them potent inhibitors of (Ser/Cys)-based enzymes. The unique reactivity of β-lactones allows them to form a covalent, but often reversible, long-lived acyl enzyme complex with (Ser/Cys)-based enzymes as a result of nucleophilic attack of the catalytic serine (or cysteine) residue on the β-lactone ring.^33,34^ This characteristic has driven interest in their development as pharmacological agents, particularly for inhibiting enzymes involved in lipid metabolism. The best example of this family is the FDA-approved anti-obesity drug **Orlistat** (Fig. 1),^33,35^ which is also known to inhibit microbial (Ser/Cys)-based enzymes.^36^ Acting as a versatile (Ser/Cys)-hydrolase inhibitor, **Orlistat** impairs the activity of key mycobacterial enzymes involved in critical processes related to lipid metabolism, particularly in the biosynthesis of mycolic acids which are essential for the integrity of the bacterium's cell wall.^37^ Among them are the enzymes belonging to the cutinase-like family proteins including the essential *M. tuberculosis* phospholipase/thioesterase Cut6 (Rv3802c);^38–40^ enzymes belonging to the hormone-sensitive lipase (HSL) family member proteins (*i.e.*, Lip-HSL);^41,42^ the polyketide synthase-13 thioesterase domain (Pks13-TE) as well as the mycolyltransferase antigen 85C.^43,44^ Finally, **Orlistat** has been shown to inhibit the growth of various mycobacterial species with minimum inhibitory concentrations (MIC) of 10–60 μg mL^−1^ (ref. 45) and demonstrated strong synergistic effects with vancomycin against *M. tuberculosis* H37Rv, reducing its MIC (50 μg mL^−1^) by approximately 16-fold.^46^ Various structural modifications of the **Orlistat** pharmacophore have been explored to enhance the specificity and antibacterial potency of newly synthesized analogs.^40,45,47,48^ In particular, the β-lactone EZ120 (Fig. 1) displayed promising antitubercular activity (MIC ∼0.7 μg mL^−1^ = 1.6 μM) and was found to target the antigen 85 enzymes and Pks13-TE.^47^

The multi-target nature of β-lactones makes them promising candidates for the development of new derivatives that may target lipid-processing enzymes critical for mycobacterial growth and survival.

A few years ago, we reported the synthesis and antimycobacterial activity of a first series of 16 long- and medium-chain mono- and disubstituted β-lactones, namely VM001–VM016.^45^ Although *M. abscessus* growth was barely affected, six β-lactones were active against *M. tuberculosis* (MIC_50_ ∼ 20–65 μg mL^−1^), with VM008 [*trans*-(*Z*)-3-(hexadec-7-en-1-yl)-4-propyloxetan-2-one = *trans*-VM001] (Fig. 1) being the best growth inhibitor (MIC_50_ ∼ 19.7 μg mL^−1^).^45^

Given the promising antibacterial activities of this best compound,^45^VM008 chemical structure has been used as a template to synthesize a new set of 27 β-lactone and 3 β-lactam derivatives (VM compounds – Fig. 1) by varying the nature of the R^1^ and R^2^ alkyl chains to modulate their lipophilicity as a means of improving their activity. Their respective anti-mycobacterial activity was further assessed against *M. tuberculosis* and the two variants S & R of *M. abscessus*. Remarkably, and contrary to the first series of β-lactone derivatives,^45^ the determined MIC revealed that some VM β-lactones and β-lactams were able to inhibit *M. abscessus* growth *in vitro* in culture broth medium and/or inside infected macrophages. In addition, using a competitive activity-based protein profiling approach,^28,29^ the potential target enzymes of the newly synthesized VM043, identified as the most active inhibitor of extracellular bacterial growth, were further identified.

## 2. Experimental section

### 2.1. Chemistry

Compounds 1a–g, 3a–c, 8a–b, 16, 21, 27, 32a–b and 38 were commercially available. The synthetic methods and the characterization data of all synthesized new β-lactones as well as intermediates are included in the ESI† file.

#### 2.1.1. General procedure for the synthesis of final β-lactone derivatives

##### General procedure I. Aldol reaction for the synthesis of α,β-substituted β-hydroxy acids (see Schemes 1, 5 and 6, methods A, F, G, H and I)

To a stirring solution of diisopropylamine (3 mmol) in dry THF (2 mL), under argon at 0 °C, a solution of 1.6 M *n*-BuLi in hexane (3 mmol) was slowly added *via* syringe and the solution of LDA was stirred at 0 °C for 10 min. The carboxylic acid (1 mmol) in dry THF (3 mL) was then added and the solution was stirred at 0 °C for 1 h. Then, the appropriate aldehyde (1.3 mmol) in dry THF (2 mL) was added and the solution was stirred at 0 °C for 1 h and at room temperature overnight. The solvent was removed under reduced pressure. The reaction mixture was acidified with 1 N HCl and extracted with Et_2_O (3 × 30 mL). The organic layers were combined, washed with brine (30 mL) and dried. The solvent was removed and the product was purified by column chromatography eluting with a gradient of CHCl_3_/MeOH 97 : 3 to 95 : 5 (v/v).

**Scheme 1:**
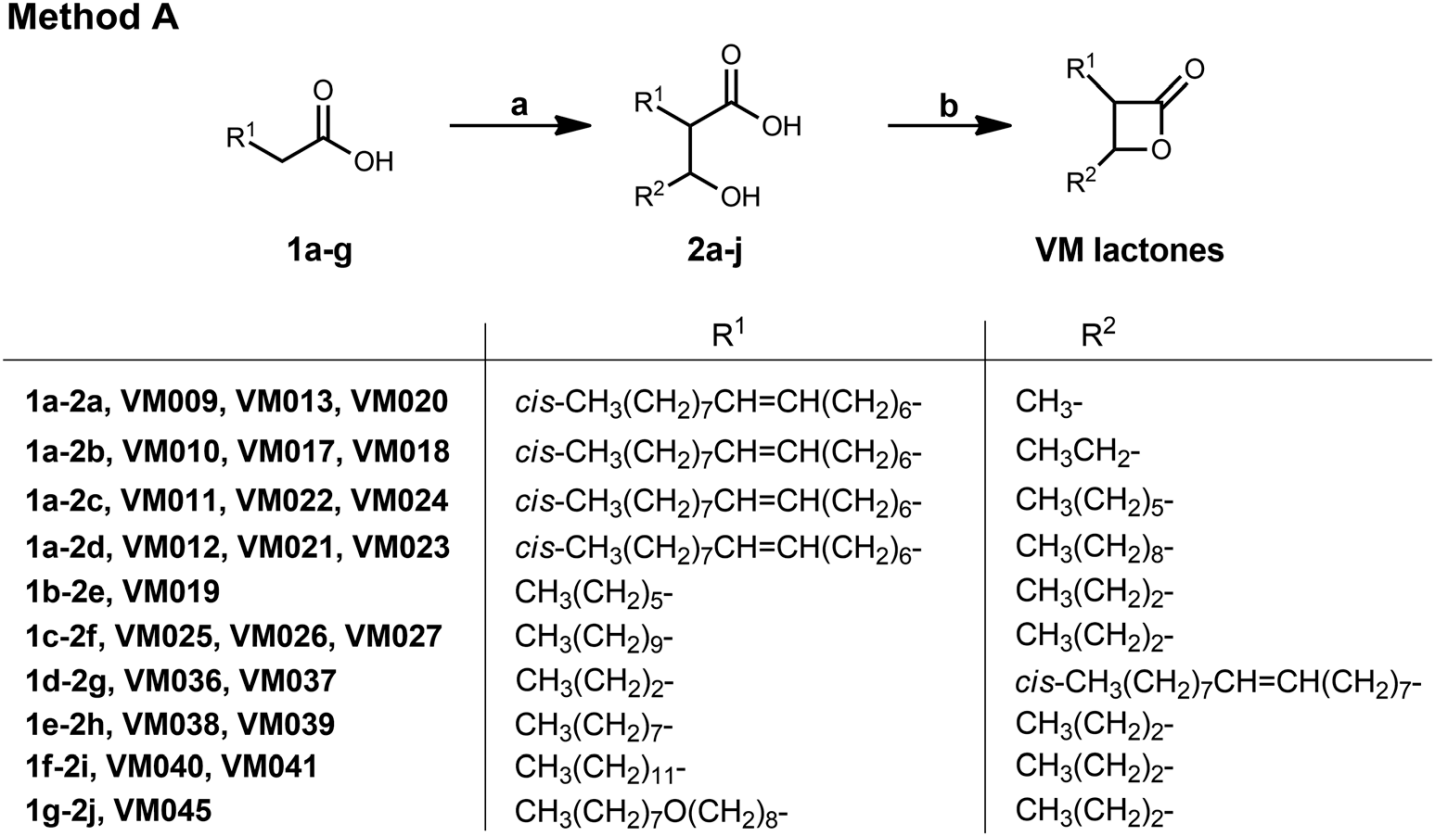
Synthesis of β-lactones form an aldol reaction (method A). Reagents and conditions: (a) i) LDA (prepared *in situ* from (i-Pr)_2_NH and *n*-BuLi), dry THF, 0 °C, 1 h; ii) R^2^CHO, dry THF, 0 °C, 1 h, then r.t. 16 h; iii) 1 N HCl, 33–93%; (b) i) *p*-TsCl, dry pyridine, 0 °C, 1 h, then 4 °C, 3 days, 36–70%; ii) chromatographic separation of the racemic mixtures of *cis*- and *trans*-β-lactones.

##### General procedure II. β-Lactone cyclization using p-TsCl in pyridine (see Scheme 1, method A)

To a stirring solution of the β-hydroxy acid (1 mmol) in dry pyridine (2 mL), under argon at 0 °C, *p*-toluenesulfonyl chloride (2 mmol) in dry pyridine (1 mL) was added slowly *via* a syringe. The solution was stirred at 0 °C for 1 h and kept at 4 °C for 3 days. Then, Et_2_O (30 mL) was added, and the organic layer was washed with 10% Na_2_CO_3_ (2 × 30 mL), 1 N HCl (2 × 30 mL) and brine (30 mL). The organic layer was dried, and the solvent was removed *in vacuo*. The product was purified by column chromatography eluting with a gradient of hexane/EtOAc.

##### General procedure III. β-Lactone cyclization using EDC·HCl and DMAP (see Schemes 2 and 3, methods B–C)

In a flame-dried flask under argon, a solution of the β-hydroxy acid (1 mmol) in dry CH_2_Cl_2_ (18 mL) was added, followed by EDC·HCl (1.6 mmol) and DMAP (0.1 mmol) and the solution was stirred at r.t. for 3 days. CH_2_Cl_2_ (20 mL) and H_2_O (20 mL) were added and then the organic layer was washed with brine (20 mL). The organic layer was dried, and the solvent was removed *in vacuo*. The product was purified by column chromatography eluting with a gradient of hexane/EtOAc.

**Scheme 2:**
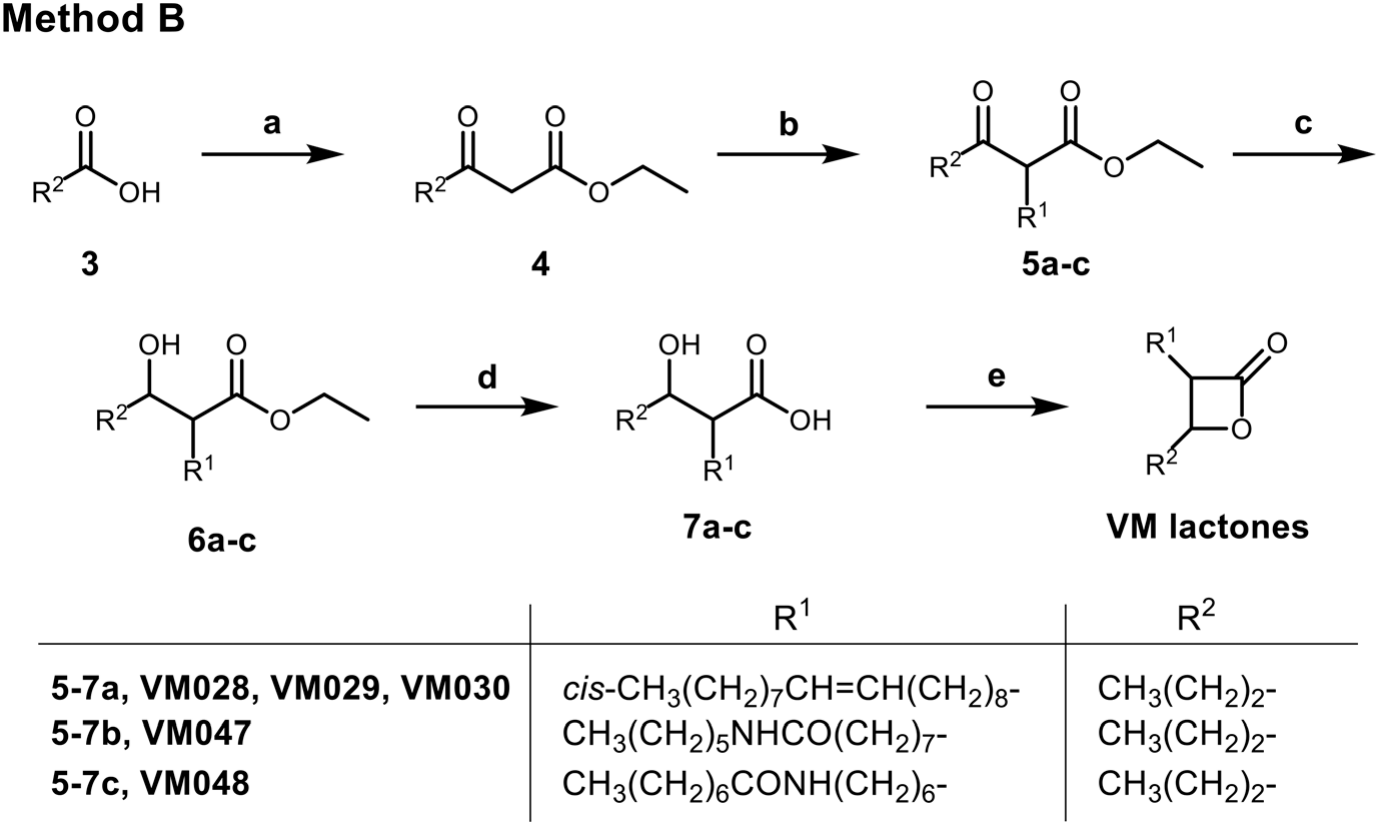
Synthesis of β-lactones from appropriately substituted β-keto ester (method B). Reagents and conditions: (a) i) CDI, dry THF, r.t., 6 h; ii) MgCl_2_, EtOCOCH_2_COOK, r.t., 18 h; iii) aq. HCl 1 N, 95%; (b) K_2_CO_3_, R^1^I, acetone/DMF, reflux, 18 h, 50–78%; (c) NaBH_4_, EtOH, 0 °C 30 min, then r.t. 3 h, 70–86%; (d) NaOH 1 N, 1,4-dioxane, r.t., 16 h, 54–85%; (e) for VM028: i) *p*-TsCl, dry pyridine, 0 °C, 1 h, then 4 °C, 3 days, 60%; ii) chromatographic separation of the racemic mixtures of *cis*- and *trans*-β-lactones; or for VM047 and VM048: EDC·HCl, DMAP, dry DCM, r.t., 72 h, 39–45%.

**Scheme 3:**
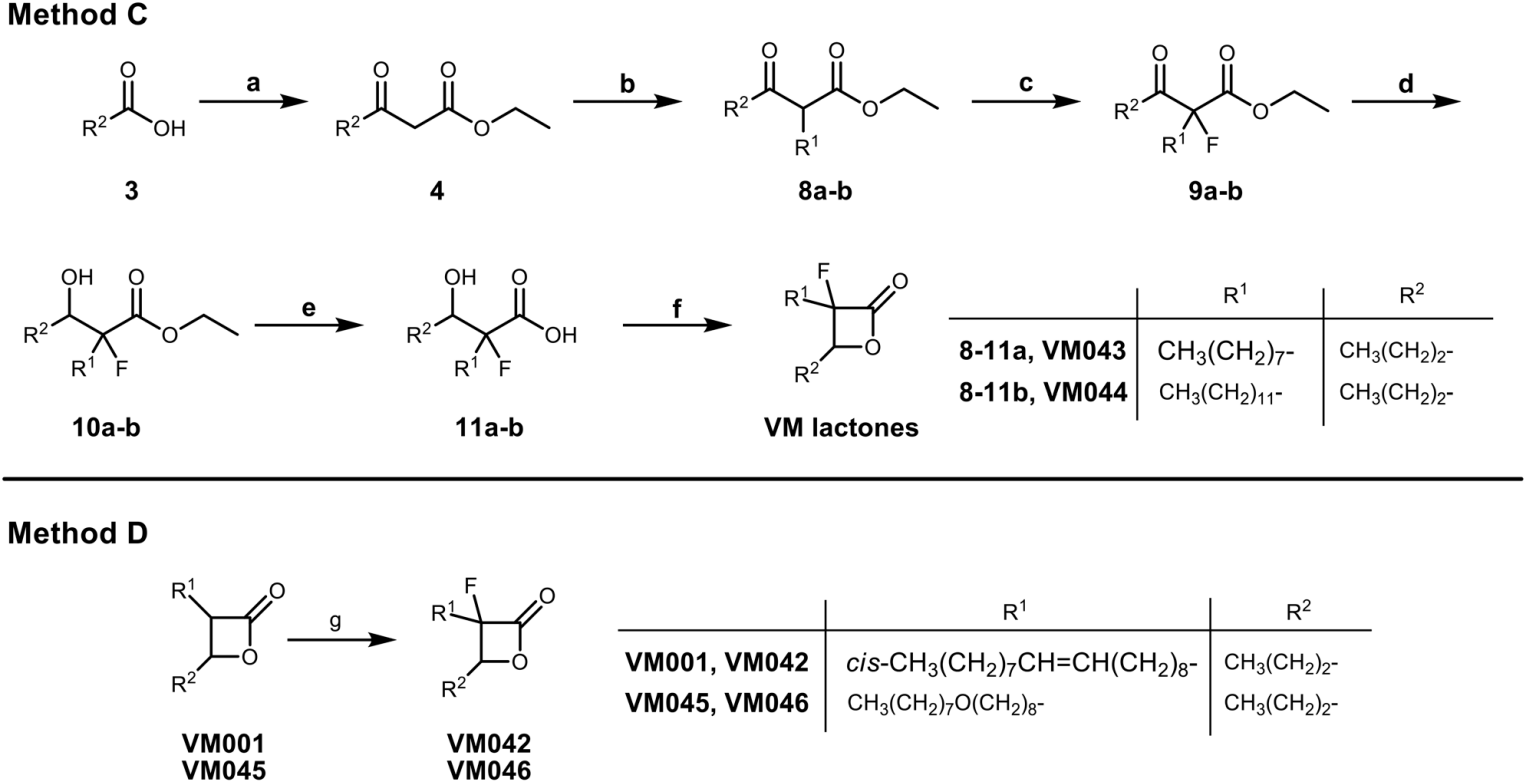
Synthesis of fluorinated β-lactones (methods C & D). Reagents and conditions: (a) i) CDI, dry THF, r.t., 6 h; ii) MgCl_2_, EtOCOCH_2_COOK, r.t., 18 h; iii) aq. HCl 1 N, 95%; (b) K_2_CO_3_, R^1^I, acetone/DMF, reflux, 18 h, 67–70%; (c) i) NaH, dry THF, −20 °C, 1 h, ii) Selectfluor, dry MeCN, −20 °C, 2.5 h, 78–82%; (d) NaBH_4_, EtOH, 0 °C 30 min, then r.t. 3 h, 61–82%; (e) NaOH 1 N or LiOH 1 N, THF or 1,4-dioxane, r.t., 16 h, 51–75%; (f) EDC·HCl, DMAP, dry DCM, r.t., 72 h, 29–36%; (g) i) LDA (prepared *in situ* from (i-Pr)_2_NH and *n*-BuLi), dry THF, −78 °C, 30 min, ii) NFSI, dry THF, −78 °C to r.t., 2.5 h, 23–39%.

##### General procedure IV. Late-stage fluorination of β-lactones (see Scheme 3, method D)

In a flame-dried flask with a solution of diisopropylamine (1.6 mmol) in dry THF (1 mL), under argon at 0 °C, a solution of *n*-BuLi 1.6 μ in hexane (1.6 mmol, 1 mL) was slowly added *via* a syringe and the solution of LDA was stirred at 0 °C for 10 min and then cooled at −78 °C. At −78 °C a solution of the β-lactone (1 mmol) in dry THF (7 mL) was added *via* a syringe and the solution was left stirring for 30 min at −78 °C. Then a solution of NFSI (2 mmol) in dry THF (2 mL) was added and the reaction was left to warm up to −15 °C and left stirring for additional 1.5 h at −15 °C. Then THF was removed *in vacuo*, EtOAc (30 mL) and 10% NaHCO_3_ (30 mL) were added. The organic layer was washed with brine (30 mL), dried and the solvent was removed *in vacuo*. The product was purified by column chromatography eluting with a gradient of hexane/EtOAc.

##### General procedure V. Deprotection of TIPS-protected terminal alkyne β-lactones using TBAF (see Scheme 5, methods F–H)

In a flame-dried flask with a solution of the TIPS-protected terminal alkyne β-lactone (1 mmol) in dry THF (5 mL) under argon, TBAF (2 mmol) in dry THF (2 mL) was added and the mixture was left stirring for 3 h at r.t. The progress of the reaction was monitored by TLC. Once the starting material was consumed, saturated NH_4_Cl was added (20 mL) and the crude product was extracted with Et_2_O (3 × 20 mL). The organic layer was washed with brine (20 mL), dried and the solvent was removed *in vacuo*. The product was purified by column chromatography eluting with a gradient of hexane/EtOAc or hexane/Et_2_O.

#### 2.1.2. Synthesis of new β-lactone derivatives VM025, VM026, VM027, VM043, VM045 and VM046

##### 3-Decyl-4-propyloxetan-2-one (VM025)

Prepared according to general procedure II using 2f; purified by column chromatography eluting with a gradient of hexane/EtOAc starting 97 : 3 to 95 : 5 (v/v). VM025 gives rise to VM026 and VM027.

Isolated mixture of diastereomers after column chromatography (dr 1 : 1). Yield 70%; ^1^H NMR (200 MHz, CDCl_3_) *δ* 4.59–4.46 (m, 0.5H), 4.26–4.15 (m, 0.5H), 3.65–3.50 (m, 0.5H), 3.21–3.08 (m, 0.5H), 1.91–1.08 (m, 24H), 0.96 (t, *J* = 7 Hz, 3H), 0.86 (t, *J* = 7 Hz, 3H). ^13^C NMR (50 MHz, CDCl_3_) *δ* 172.5, 171.8, 78.1, 75.6, 56.3, 52.8, 36.7, 32.4, 32.1, 29.8, 29.7, 29.6, 29.5, 28.1, 27.8, 27.2, 24.1, 22.8, 19.1, 18.6, 14.3, 14.0, 13.9. HRMS (ESI) [M + Na]^+^: calcd for C_16_H_30_NaO_2_^+^ 277.2138, found 277.2135.

##### (±)-*trans* 3-Decyl-4-propyloxetan-2-one (VM026)

Purified by 2nd column chromatography of VM025 eluting with a gradient of hexane/EtOAc 98 : 2 to 95 : 5 (v/v). ^1^H NMR (200 MHz, CDCl_3_) *δ* 4.26–4.15 (m, 1H), 3.21–3.08 (m, 1H), 1.91–1.56 (m, 4H), 1.55–1.08 (m, 18H), 1.00–0.65 (m, 6H). ^13^C NMR (50 MHz, CDCl_3_) *δ* 172.2, 78.2, 56.4, 36.7, 32.1, 29.7, 29.5, 28.1, 27.2, 22.8, 18.6, 14.3, 14.0. HRMS (ESI) [M + Na]^+^: calcd for C_16_H_30_NaO_2_^+^ 277.2138, found 277.2137.

##### (±)-*cis* 3-Decyl-4-propyloxetan-2-one (VM027)

Purified by 2nd column chromatography of VM025 eluting with a gradient of hexane/EtOAc 98 : 2 to 95 : 5 (v/v). ^1^H NMR (200 MHz, CDCl_3_) *δ* 4.59–4.46 (m, 1H), 3.65–3.50 (m, 1H), 1.90–1.40 (m, 8H), 1.40–1.11 (m, 14H), 0.98 (t, *J* = 7 Hz, 3H), 0.88 (t, *J* = 7 Hz, 3H). ^13^C NMR (50 MHz, CDCl_3_) *δ* 171.8, 75.7, 52.8, 32.4, 32.0, 29.6, 29.5, 29.4, 29.3, 27.8, 24.1, 22.8, 19.0, 14.3, 14.0. HRMS (ESI) [M + Na]^+^: calcd for C_16_H_30_NaO_2_^+^ 277.2138, found 277.2138.

##### 3-Fluoro-3-octyl-4-propyloxetan-2-one (VM043)

Prepared according to general procedure III using 11a; purified by column chromatography eluting with hexane/EtOAc 95 : 5 (v/v). Isolated mixture of diastereomers after column chromatography (dr 65 : 35). Yield 36%; yellowish oil; ^1^H NMR (400 MHz, CDCl_3_) *δ* 4.71–4.61 (m, 0.35H), 4.50–4.42 (m, 0.65H), 2.07–1.65 (m, 4H), 1.65–1.25 (m, 14H), 1.02 (t, *J* = 7.4 Hz, 3H), 0.90 (t, *J* = 6.6 Hz, 3H). ^13^C NMR (101 MHz, CDCl_3_) *δ* 167.66 (d, *J* = 25.3 Hz), 166.93 (d, *J* = 24.2 Hz), 102.90 (d, *J* = 217.2 Hz), 102.00 (d, *J* = 224.2 Hz), 83.44 (d, *J* = 25.3 Hz), 82.26 (d, *J* = 22.2 Hz), 32.36 (d, *J* = 23.2 Hz), 31.80, 31.77, 31.46 (d, *J* = 3.0 Hz), 30.95 (d, *J* = 5.1 Hz), 29.65, 29.39, 29.21, 29.11, 29.08, 28.72 (d, *J* = 23.2 Hz, *C*H_2_CF), 22.63, 22.62, 22.58, 22.54, 22.16, 22.12, 18.76, 17.95, 14.07, 13.79, 13.66. ^19^F NMR (376 MHz, CDCl3) *δ* −159.76, −173.43. HRMS (ESI) [M + Na]^+^: calcd for C_14_H_25_FNaO_2_^+^ 267.1731, found 267.1732.

##### 3-(8-(Octyloxy)octyl)-4-propyloxetan-2-one (VM045)

Prepared according to general procedure II using 2j; purified by column chromatography eluting with a gradient of hexane/EtOAc starting 97 : 3 to 95 : 5 (v/v). Isolated mixture of diastereomers after column chromatography (dr 3 : 7). Yield 60%; colorless oil; ^1^H NMR (200 MHz, CDCl_3_) *δ* 4.59–4.50 (m, 0.7H), 4.26–4.20 (m, 0.3H), 3.65–3.55 (m, 0.7H), 3.32 (t, *J* = 7.0 Hz, 4H), 3.20–3.14 (m, 0.3H), 1.91–1.50 (m, 10H), 1.50–1.22 (m, 20H), 1.00 (t, *J* = 7 Hz, 3H), 0.86 (t, *J* = 7 Hz, 3H). ^13^C NMR (50 MHz, CDCl_3_) *δ* 172.28, 171.59, 77.92, 75.45, 70.98, 70.88, 56.17, 52.67, 36.50, 32.21, 31.83, 29.78, 29.75, 29.46, 29.36, 29.33, 29.27, 29.25, 29.22, 27.87, 27.58, 26.97, 26.20, 26.15, 23.92, 22.65, 18.89, 18.43, 14.08, 13.79, 13.75. HRMS (ESI) [M + Na]^+^: calcd for C_22_H_42_NaO_3_^+^ 377.3026, found 377.3029.

##### 3-Fluoro-3-(8-(octyloxy)octyl)-4-propyloxetan-2-one (VM046)

Prepared according to general procedure IV using VM045; purified by column chromatography eluting with hexane/Et_2_O 95 : 5 (v/v). Isolated mixture of diastereomers after column chromatography (dr 55 : 45). Yield 23%; ^1^H NMR (400 MHz, CDCl_3_) *δ* 4.61–4.53 (m, 0.55H), 4.40–4.34 (m, 0.45H), 3.32 (t, *J* = 6.8 Hz, 4H), 1.98–1.14 (m, 30H), 0.93 (t, *J* = 7.3 Hz, 3H), 0.81 (t, *J* = 6.5 Hz, 3H). ^13^C NMR (101 MHz, CDCl_3_) *δ* 166.63 (d, *J* = 25.2 Hz), 165.91 (d, *J* = 23.90 Hz), 101.91 (d, *J* = 215.5 Hz), 101.00 (d, *J* = 224.3 Hz), 82.40 (d, *J* = 25.2 Hz), 81.24 (d, *J* = 21.4 Hz), 69.98, 69.86, 69.84, 31.31 (d, *J* = 2.5 Hz), 30.82, 30.43 (d, *J* = 2.5 Hz), 29.92 (d, *J* = 3.8 Hz), 28.75, 28.71, 28.56, 28.45, 28.30, 28.27, 28.24, 28.19, 28.18, 27.67 (d, *J* = 22.7), 25.18, 25.11, 21.65, 21.52 (d, *J* = 3.8 Hz), 21.10 (d, *J* = 3.8 Hz), 17.73, 16.92, 13.09, 12.78, 12.66. ^19^F NMR (376 MHz, CDCl3) *δ* −159.71, −173.39. HRMS (ESI) [M + Na]^+^: calcd for C_22_H_41_FNaO_3_^+^ 395.2932, found 395.2935.

#### 2.1.3. Synthesis of β-lactone probes VM053_*p*_ and VM055_*p*_

##### (±)-*trans* 3-(Oct-7-yn-1-yl)-4-propyloxetan-2-one (VM053_*p*_)

Prepared according to general procedure V using 35b; purified by column chromatography eluting with a gradient of hexane/EtOAc starting 97 : 3 to 92 : 8 (v/v). Only (±)-*trans* diastereomers were isolated after column chromatography. Yield 98%; colorless oil; ^1^H NMR (400 MHz, CDCl_3_) *δ* 4.27–4.23 (m, 1H), 3.21–3.17 (m, 1H), 2.21 (t, *J* = 7 Hz, 2H), 1.96 (s, 1H), 1.91–1.80 (m, 2H), 1.79–1.69 (m, 2H), 1.57–1.42 (m, 10H), 1.01 (t, *J* = 7.4 Hz, 3H). ^13^C NMR (101 MHz, CDCl_3_) *δ* 171.57, 84.47, 77.93, 68.29, 56.14, 36.51, 28.77, 28.35, 28.26, 27.82, 26.86, 18.45, 18.33, 13.78. HRMS (ESI) [M + Na]^+^: calcd for C_14_H_22_NaO_2_^+^ 245.1512, found 245.1510.

##### (±)-*trans* 3-(8-Azidooctyl)-4-propyloxetan-2-one (VM055_*p*_)

Prepared according to general procedure I using 39, followed by general procedure II; purified by column chromatography eluting with hexane/EtOAc 95 : 5 (v/v). Only (±)-*trans* diastereomers were isolated after column chromatography. Yield 12%; colorless oil; ^1^H NMR (400 MHz, CDCl_3_) *δ* 4.27–4.23 (m, 1H), 3.28 (t, *J =* 7 Hz, 2H), 3.22–3.17 (m, 1H), 1.92–1.80 (m, 2H), 1.78–1.69 (m, 2H), 1.52–1.34 (m, 14H), 1.01 (t, *J* = 7.4 Hz, 3H). ^13^C NMR (101 MHz, CDCl_3_) *δ* 171.58, 77.91, 56.16, 51.45, 36.50, 29.18, 29.15, 29.00, 28.80, 27.87, 26.93, 26.65, 18.44, 13.77. HRMS (ESI) [M + Na]^+^: calcd for C_14_H_25_NaN_3_O_2_^+^ 290.1839, found 290.1835.

### 2.2. Biological evaluation

#### 2.2.1. Chemicals

Amikacin, kanamycin, isoniazid and imipenem were purchased from Euromedex (Souffelweyersheim, France). Stock solutions of each β-lactone derivative (5 mg mL^−1^) in which the compounds were found to be completely soluble in dimethyl sulfoxide (DMSO), were prepared and stored at −20 °C before use.

#### 2.2.2. Bacterial strains and growth conditions

*M. abscessus* CIP104536^T^ with either a smooth (S) or a rough (R) morphotype, and *M. tuberculosis* mc^2^6230 (H37Rv Δ*RD1* Δ*panCD*^49^) were cultured in Middlebrook 7H9 liquid media (BD Difco) supplemented with 0.05% Tween 80 (Sigma-Aldrich, Saint-Quentin Fallavier, France), 0.2% glycerol (Euromedex, France) and 10% Oleic Albumin Dextrose Catalase (OADC enrichment, BD Difco) (7H9-S^OADC^). In the case of *M. tuberculosis* mc^2^6230, 24 μg mL^−1^d-pantothenate (Sigma-Aldrich) was also added in the 7H9-S^OADC^ medium. Recombinant *M. abscessus* S bacterial luciferase (Lux) reporter strains was used for measurement of bacterial load inside infected macrophages. This latter *M. abscessus* S-Lux strain was generated by electroporation of the *M. abscessus* S strain with the integrating shuttle plasmid pMV306hsp + LuxG13 (ref. 50) (Addgene plasmid #26161) optimized for mycobacteria, and carrying the constitutive P_hsp60_ and P_G13_ promoters driving expression of *luxAB* and *luxCDE* luciferase genes.^51,52^ The *M. abscessus* S-LuxG13 strains were grown in 7H9-S^OADC^ with 250 μg mL^−1^ kanamycin (Euromedex, France). All cultures were incubated at 37 °C under mild agitation at 50 rpm.

#### 2.2.3. Antimycobacterial susceptibility testing

Antimycobacterial susceptibility testing was performed using the Middlebrook 7H9 broth microdilution method. MICs were determined in 96-well flat-bottom Nunclon Delta Surface microplates with lid (Thermo-Fisher Scientific, Illkirch, France) using the resazurin microtiter assay (REMA).^45,53,54^ Briefly, log-phase bacteria were diluted to a cell density of 5 × 10^6^ CFU mL^−1^ and 100 μL of this inoculum was grown in a 96-well plate in the presence of serial dilutions of each β-lactone analog. After 3–5 days (*M. abscessus*) or 10–14 days (*M. tuberculosis*) incubation at 37 °C, 20 μL of a 0.025% (w/v) resazurin solution was added to each well (200 μL) and incubation was continued until the appearance of a color change (from blue to pink) in the control well (*i.e.*, bacteria without antibiotics). Fluorescence of the resazurin metabolite resorufin (*λ*_excitation_, 530 nm; *λ*_emission_, 590 nm) was then measured and the lowest compound concentration leading to 50% and 90% inhibition of bacterial growth was defined as the MIC_50_ and MIC_90_, respectively. Amikacin (AMK) (Euromedex, France) was used as reference drugs. All experiments were performed independently at least three times. See ESI† for detailed protocol.

#### 2.2.4. Determination of cytotoxicity (resazurin assay)

The cytotoxicity of the new synthesized β-lactone analogs against eukaryotic cells was measured based on the reduction of resazurin as a value of cellular viability by metabolic activity, as previously described.^45,54,55^ Murine (Raw264.7) macrophages (American Type Culture Collection TIB-71) were incubated with two-fold dilution of each compound for 24 h at 37 °C and 5% CO_2_. Then, 20 μL of a 0.025% (w/v) resazurin solution was added to each well, and fluorescence was measured following a 4 h incubation as described above. The compound concentration leading to 50% macrophage cell death was defined as the CC_50_.^28,29,56^ All experiments were performed as three independent biological replicates. See ESI† for detailed protocol.

#### 2.2.5. Intramacrophage killing assay

The intracellular growth of *M. abscessus* S-LuxG13 (ref. 51 and 52) luminescent strain was assessed following a 24 h exposure of infected Raw264.7 murine macrophages cell line (American Type Culture Collection TIB-71) to two-fold dilutions of the selected β-lactone analogs. To avoid growth of extracellular mycobacteria, cells were extensively washed and treated with amikacin (250 μg mL^−1^) prior to treatment with the β-lactone analogs. Imipenem (IMP; 80 μg mL^−1^) was used as positive control for this intracellular killing assay. After incubation, luminescence measurement was used to assess intracellular bacterial viability of *M. abscessus* S-LuxG13 strain.^51,52^ The lowest compound concentration leading to 50% of the relative luminescence unit (RLU%) was defined as the MIC_50Raw_. Each experiment was performed as three independent biological replicates. See ESI† for detailed protocol.

#### 2.2.6. Copper-free click chemistry activity-based protein profiling^57–59^

Bacterial suspension of *M. abscessus* S in 7H9S^OADC^ was adjusted at a final theoretical OD_600nm_ of 20 and then incubated with VM043 (122 μg mL^−1^ final concentration) or DMSO (control) at 37 °C for 4 h under shaking at 200 rpm. Bacteria were then washed 3 times with PBS containing 0.05% Tween 80, resuspended in 7H9TG^OADC^ and then re-incubated at 37 °C with VM055_*p*_ (54 μg mL^−1^) or DMSO for 4 h at 200 rpm. Bacteria were harvested, washed, resuspended in PBS at a 1 : 1 (w/v) ratio and then lysed by mechanical disruption on a BioSpec Beadbeater. Each sample (300 μL – 0.3 mg total proteins) was further subjected to copper-free azide-alkyne cycloaddition and enrichment with DBCO-agarose bead 50% slurry (Click Chemistry Tools, ref. 1034) according to the manufacturer's instructions. The beads containing bound, biotinylated proteins were resuspended in 30 μL PBS buffer pH 7.4, snap frozen in liquid nitrogen and stored at −80 °C before mass spectrometry experiments. See ESI† for detailed protocol.

#### 2.2.7. Mass spectrometry analysis for protein identification and quantification

The beads were further processed for mass spectrometry as described in Babin *et al.*^59^ Briefly, proteins on beads were digested with 0.5 μg trypsin sequencing grade (Promega Inc.) in 50 mM TEAB for 16 h at 37 °C. Peptides were extracted with 20% acetonitrile, dried, and further desalted on C18 Micro SpinColumns (Harvard Bioscience, Inc), dried again and diluted in 15 μL water/acetonitrile/ (98/2, v/v) containing 0.05% TFA. 20% of each sample was analyzed twice by liquid chromatography (LC)-tandem MS (MS/MS) using a Q-Exactive plus Mass Spectrometer (Thermo Fisher Scientific, San Jose, CA) online with a nanoRSLC Ultimate 3000 chromatography system (Thermo Fisher Scientific, Sunnyvale, CA). Relative intensity-based label-free quantification (LFQ) was processed using the DIA-NN 1.8 algorithm. Raw files were searched against the *M. abscessus* database (UP000007137) extracted from UniProt (date 2021-11-15; 4940 entries).^60^ The statistical analysis was done with the Perseus program (version 1.6.15.0)^61^ from the MaxQuant environment (https://www.maxquant.org). Differential proteins were detected using a two-sample *t*-test using permutation-based FDR-controlled at 5 and employing 250 permutations. The *p*-value was adjusted using a scaling factor s0 with a value of 1.^62^ See ESI† for detailed protocol.

The mass spectrometry proteomics data have been deposited to the ProteomeXchange Consortium (https://www.proteomexchange.org)^63^*via* the PRIDE partner repository^64^ (https://www.ebi.ac.uk/pride/login) with the dataset identifiers PXD057836.

## 3. Results and discussion

### 3.1. Chemistry

Based on our previous results on the activity of long and medium chain substituted β-lactones,^45^ we decided to synthesize a new set of β-lactone derivatives bearing saturated aliphatic chains located at the α-position such as C_6_, C_8_, C_10_, C_12_, and an ether analogue of our most potent β-lactone VM001; while at the β-position a propyl chain that had proved to be the optimal substituent^45^ was used. We also synthesized β-lactone VM036, which has an oleyl chain at the β-position and a propyl chain at the α-position, *i.e.* in opposite positions compared to β-lactone VM001. Finally, we resynthesized β-lactones VM009, VM010, VM011 and VM012 to separate the racemic mixtures of *cis*- and *trans*-β-lactones to be tested independently. For the synthesis of these β-lactones, method A was followed (Scheme 1). The first step is an aldol reaction between the appropriate carboxylic acid and aldehyde using LDA as the base which deprotonates the proton in the α-position of the acid function and yields the corresponding β-hydroxy acids 2 after treatment with aqueous HCl. The β-hydroxy acids 2 were then cyclized using *para*-toluenesulfonyl chloride (*p*-TsCl) in dry pyridine, and the resulting β-lactones were further purified by column chromatography (Scheme 1). In all cases, the mixture of all 4 diastereomers was obtained. In the cases of VM019 and VM045 the *cis*- and *trans*-diastereomeric mixture were inseparable, while in the cases of VM009–VM012, VM025, VM036, VM038 and VM040 both *cis*- and *trans*-pure racemic mixtures were obtained after a second chromatography of the initial mixture of all 4 diastereomers. The *cis*- and *trans*-isomers were assigned by comparison of the corresponding ^1^H NMR and ^13^C NMR chemical shifts of α-CH and β-CH to our previous work for both β-hydroxy acids and β-lactones.^45^

For the synthesis of β-lactones VM028, VM047 and VM048, a different synthetic route (method B – Scheme 2) was chosen either to insert the desired substituent from a commercially available starting material or to avoid any side reactions that would be caused by the use of LDA in the aldol reaction mentioned above. In this procedure, butyric acid 3 reacted with 1,1′-carbonyl diimidazole (CDI) and monoethyl malonic acid magnesium salt using Masamune's method^65^ to yield the corresponding β-keto esters 4. Deprotonation of the more acidic proton in α-position of the β-keto ester carbonyl function using less reactive K_2_CO_3_ base, followed by substitution reaction with an alkyl iodide bearing the desired R^1^ chain as substituent yielded the substituted β-keto esters 5 which were subsequently reduced using NaBH_4_. Hydrolysis of the resulting β-hydroxy esters 6 to the corresponding β-hydroxy acids 7 and a final cyclization reaction as described above using *p*-TsCl/pyridine afforded the final β-lactone VM028. For β-lactones VM047 and VM048, 1-ethyl-3-(3-dimethylaminopropyl)carbodiimide hydrochloride (EDC·HCl) with a catalytic amount of 4-dimethylaminopyridine (DMAP) was used. In the case of VM028 both racemic mixtures of *cis*- and *trans*-β-lactones were isolated by column chromatography, whereas the diastereomers of VM047 and VM048 were inseparable by column chromatography.

We also decided to incorporate a fluorine atom as second substituent at the α-position of the β-lactone ring. Fluorine atoms are widely used in compounds of medicinal interest as they enhance physicochemical properties such as solubility and lipophilicity, but also often increase the affinity of bioactive compounds for enzymes and receptors. To that end, two different synthetic procedures, methods C & D, were established as depicted in Scheme 3. Method C is similar to the previously described method B (Scheme 2) with the addition of an extra synthetic fluorination step (Scheme 3 – method C, step c) after the synthesis of the monosubstituted β-keto esters 8 and before the cyclisation step. The fluorination step that we introduced here for the first time included the use of sodium hydride for the deprotonation of the monosubstituted β-keto ester 8, followed by the use of Selectfluor to insert the fluorine atom. The β-lactones VM043 and VM044 were successfully prepared according to this procedure.

Furthermore, in order to incorporate the fluorine atom directly on the β-lactone ring, we further developed a more efficient fluorination method where the fluorination step would be performed at the last step of the synthesis, after the cyclisation. This proposed late-stage fluorination method would be much more efficient since it could be applied to already synthesized β-lactone molecules, or to new β-lactones such as VM045. Indeed, this new method D (Scheme 3) includes the use of LDA on the β-lactone and treatment with *N*-fluorobenzenesulfonimide (NFSI) as fluorinating electrophile. VM042 and VM046 which are the fluorinated analogues of VM001 and VM045, respectively, have been prepared *via* this late-stage fluorination method D.

**Scheme 4:**
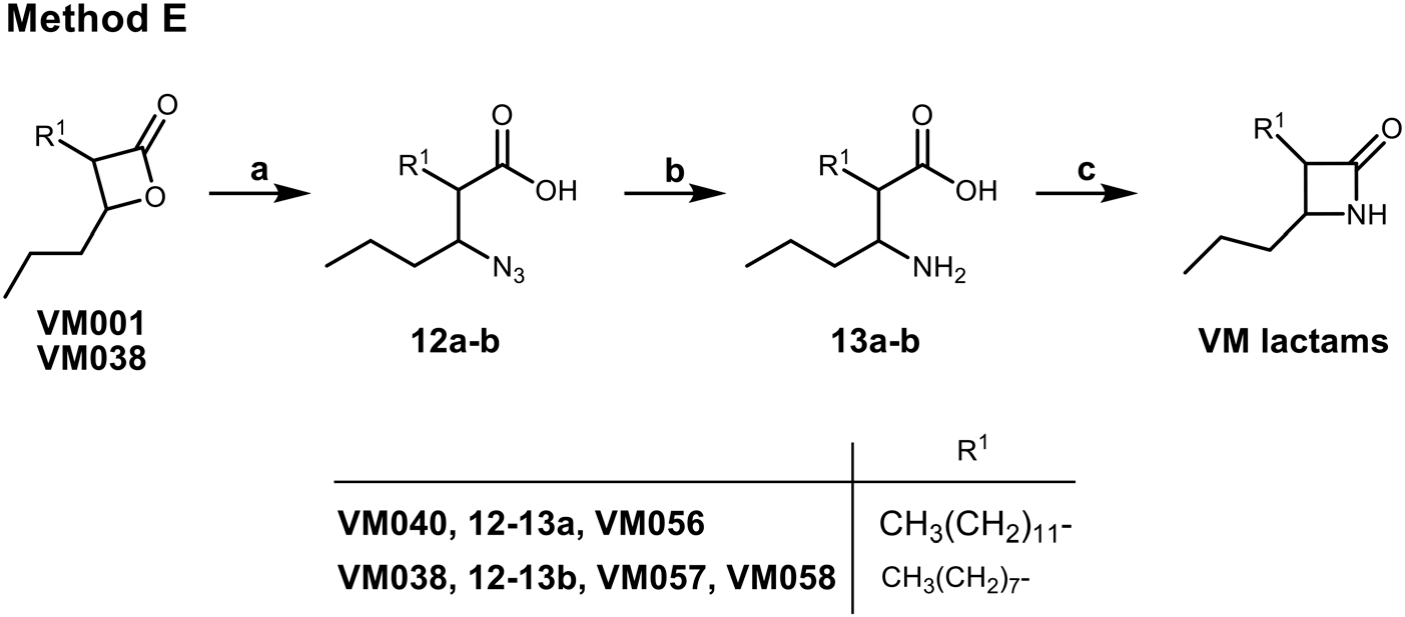
Synthesis of β-lactams VM056 and VM057 (method E). Reagents and conditions: (a) NaN_3_, DMF, 60 °C, 16 h, 65–77%; (b) H_2_, 10% Pd/C, MeOH, r.t., 2 h, 36–44%; (c) i) Mukaiyama's reagent, Et_3_N, CH_3_CN, reflux, 4 h, then r.t., 16 h, 65–71%; ii) chromatographic separation of the racemic mixture of the *trans*-β-lactone VM058.

Finally, three β-lactam analogues were synthesized. For this purpose, a synthetic method (Scheme 4 – method E) starting from the corresponding β-lactone was established. After opening the β-lactone ring by the nucleophilic attack of NaN_3_ to give the corresponding β-azido acids 12, the azide group was reduced by catalytic hydrogenation using 10% Pd/C, and finally the β-lactam ring of 13 was closed using Mukaiyama's reagent^66^ in a very dilute solution to avoid the intermolecular reaction (Scheme 4).

### 3.2. Antimycobacterial activity of the new β-lactone compounds

Drug susceptibility testing of the 27 β-lactone and 3 β-lactam derivatives was assessed against two pathogenic mycobacterial species: *M. tuberculosis* mc^2^6230,^49^ and the two S & R variants of *M. abscessus*. The corresponding MIC_50_/MIC_90_ values for each molecule, as determined by the resazurin microtiter assay (REMA),^30,45,53,54^ are reported in Table 1. Amikacin (AMK) which is a core drug used in the treatment of *M. abscessus*, with MIC_50_/MIC_90_ of 4/32 and 8/32 μg mL^−1^ against the S & R variant,^67^ respectively; as well as a second-line injectable drug for the treatment of multidrug resistant TB, with MIC_50_/MIC_90_ ∼1 μg mL^−1^ against *M. tuberculosis* H37Rv,^68^ was used as reference drug.

**Table 1:** Antibacterial activities of **Orlistat** and β-lactone derivatives against *M. tuberculosis* mc^2^6230 and *M. abscessus* S and R variants^a^

Compounds	MIC_50_/MIC_90_^a^ (μg mL^−1^)		
	*M. abscessus* CIP 104536^T^		*M. tuberculosis* mc^2^6230
	Smooth	Rough	
**Orlistat** b	44.4 ± 0.68/45.6 ± 0.56	57.0 ± 0.11/73.5 ± 0.50	11.0 ± 0.50/13.5 ± 1.0
VM001 (C16:1 ω9)^b^	>100	>100	31.8 ± 1.5/>100
VM008 = *trans*-(*R*,*R*)-VM001^b^	>100	>100	19.7 ± 1.3/31.9 ± 1.4
VM009^b^	>100	>100	>100
VM013 = *trans*-VM009^b^	>100	>100	33.6 ± 0.21/42.9 ± 1.3
VM020 = *cis*-VM009	>100	>100	>100
VM010^b^	>100	>100	33.5 ± 0.26/69.9 ± 0.94
VM017 = *trans*-VM010	>100	>100	45.8 ± 1.0/47.8 ± 1.8
VM018 = *cis*-VM010	>100	>100	47.2 ± 1.2/53.1 ± 2.0
VM011^b^	>100	>100	>100
VM022 = *trans*-VM011	>100	59.9 ± 2.1/68.9 ± 2.7	>100
VM024 = *cis*-VM011	>100	41.9 ± 2.9/64.7 ± 0.66	>100
VM012^b^	>100	>100	>100
VM021 = *trans*-VM012	>100	>100	>100
VM023 = *cis*-VM012	>100	31.0 ± 2.8/>100	>100
VM019 (*trans*)	>100	>100	65.2 ± 0.93/67.2 ± 0.61
VM025	49.9 ± 1.3/52.4 ± 2.5	27.1 ± 0.80/>100	63.9 ± 1.6/65.1 ± 1.5
VM026 = *trans*-VM025	47.2 ± 1.5/56.7 ± 2.2	25.6 ± 1.4/>100	59.2 ± 2.8/>100
VM027 = *cis*-VM025	>100	>100	>100
VM028 (C18:1 ω9)	>100	>100	>100
VM029 = *trans*-VM028	>100	>100	>100
VM030 = *cis*-VM028	>100	>100	>100
VM037	>100	>100	>100
VM036 = *trans*-VM037	>100	>100	>100
VM039	>100	>100	24.7 ± 1.7/51.3 ± 1.3
VM038 = *trans*-VM039	>100	>100	17.7 ± 0.22/46.5 ± 1.6
VM040 (*trans*)	>100	>100	9.6 ± 0.45/29.1 ± 1.6
VM041 (*cis*)	>100	>100	17.4 ± 1.6/44.2 ± 1.7
VM042	>100	>100	10.2 ± 3.6/40.1 ± 7.5
VM043 = fluorinated VM038–039	33.8 ± 1.2/49.2 ± 3.4	59.8 ± 5.6/88.9 ± 0.3	30.2 ± 1.3/45.9 ± 2.5
VM044 = fluorinated VM040–041	>100	>100	20.8 ± 0.2/24.1 ± 0.1
VM045	>100	>100	>100
VM046 = fluorinated VM045	>100	>100	32.2 ± 4.2/45.0 ± 3.3
VM047	>100	>100	46.2 ± 4.0/>100
VM048	>100	>100	46.8 ± 8.7/>100
VM056 = VM040 β-lactam	47.3 ± 6.0/74.1 ± 3.9	>100	15.2 ± 0.6/21.8 ± 0.7
VM057 = *trans*-VM058 β-lactam	41.9 ± 7.4/44.4 ± 6.2	>100	35.5 ± 1.2/44.2 ± 1.1
VM058 = β-lactam	41.8 ± 6.3/46.6 ± 4.0	>100	23.7 ± 0.2/44.2 ± 2.4
**AMK**	2.3 ± 0.12/3.4 ± 0.11	4.3 ± 0.15/5.9 ± 0.26	0.24 ± 0.01/0.37 ± 0.02

a Minimum inhibitory concentration leading to 50% or 90% growth inhibition (MIC_50_/MIC_90_) as determined by the resazurin microtiter assay (REMA).

b Data from our previous study.^45^ AMK: amikacin. All reported values are expressed as mean ± SD of three independent assays.

Nearly all β-lactone derivatives were active against *M. tuberculosis* mc^2^6230 (*i.e.*, 18 compounds) growth (Table 1). With *M. tuberculosis*, MIC_50_ values were indicative of either a poor (MIC_50_ > 100 μg mL^−1^ for VM020 to VM024, VM027 to VM030, VM036, VM037, and VM045), a weak (MIC_50_ ∼45–66 μg mL^−1^ for VM017 to VM019, VM025, VM026, VM047, and VM048), a moderate (MIC_50_ ∼20–35 μg mL^−1^ for VM039, VM043, VM044, VM046, VM057, and VM058), or a quite good (∼9.6–17.7 μg mL^−1^ for VM038, VM040 to VM042, and VM056) antibacterial activity comparable to that of **Orlistat** (11.0 μg mL^−1^) (Table 1). Of note, 15 of the 18 active β-lactone derivatives exhibited interesting MIC_90_ values in the range of 21–67 μg mL^−1^. Remarkably, most of these latter β-lactones exhibited MIC_50_ and MIC_90_ values in the same range. Collectively, the best growth inhibitors of *M. tuberculosis* mc^2^6230 were VM040, VM044 and VM056 with MIC_50_/MIC_90_ in the range 9.6–20.8/21.8–29.1 μg mL^−1^, as compared to 0.24/0.37 μg mL^−1^ for amikacin (Table 1).

In the case of *M. abscessus*, only 6 β-lactones were found to block the growth of the S variant. These included VM025 (MIC_50_ = 49.9 ± 1.3 μg mL^−1^/MIC_90_ = 52.4 ± 2.5 μg mL^−1^), VM026 (MIC_50_ = 47.2 ± 1.5 μg mL^−1^/MIC_90_ = 56.7 ± 2.2 μg mL^−1^), VM043 (MIC_50_ = 33.8 ± 1.2 μg mL^−1^/MIC_90_ = 49.2 ± 3.4 μg mL^−1^), VM056 (MIC_50_ = 47.3 ± 6.0 μg mL^−1^/MIC_90_ = 74.1 ± 3.9 μg mL^−1^), VM057 (MIC_50_ = 41.9 ± 7.4 μg mL^−1^/MIC_90_ = 44.4 ± 6.2 μg mL^−1^), and VM058 (MIC_50_ = 41.8 ± 6.3 μg mL^−1^/MIC_90_ = 46.6 ± 4.0 μg mL^−1^) for which both MIC_50_ & MIC_90_ values were in the same order of magnitude (Table 1). Among these, three molecules were also active against *M. abscessus* R: VM025 (MIC_50_ = 27.1 ± 0.80 μg mL^−1^) and VM026 (MIC_50_ = 49.9 ± 1.3 μg mL^−1^) but with MIC_90_ values higher than 100 μg mL^−1^; and VM043 (MIC_50_ = 59.8 ± 5.6 μg mL^−1^ and a MIC_90_ = 88.9 ± 0.3 μg mL^−1^). Interestingly, although VM022 and VM024 were inactive against *M. abscessus* S (MIC > 100 μg mL^−1^), these two molecules were however found to impair the growth of the R morphotype with interesting, albeit moderate, MIC_50_/MIC_90_ values (MIC_50_ = 59.9 ± 2.1 μg mL^−1^/MIC_90_ = 68.9 ± 2.7 μg mL^−1^, and MIC_50_ = 41.9 ± 2.9 μg mL^−1^/MIC_90_ = 64.7 ± 0.66 μg mL^−1^, respectively). In addition, although VM023, which is also inactive against the S variant, has an interesting MIC_50_ value (31.0 ± 2.8 μg mL^−1^), its MIC_90_ was however higher than 100 μg mL^−1^. Overall, the best inhibitors of *M. abscessus* R growth were VM022, VM024, and VM043 which exhibited similar MIC_50_/MIC_90_ values, respectively (Table 1).

From all these results, some structure–activity relationships (SAR) tendencies can however be set up. First, and as mentioned in our previous report,^45^*trans*-β-lactones were almost >2 times more active than the corresponding *cis* isomers, and nearly as active as the *cis/trans* isomeric mixtures. This is best illustrated by the antibacterial activity of VM026 (*trans*-VM025) on *M. tuberculosis* mc^2^6230 and *M. abscessus* compared to the *cis* isomer VM027 (*cis*-VM025) and the *cis*/*trans* isomeric mixture VM025 (Table 1).

Remarkably, the three β-lactones VM025, VM026 (*trans*-VM025), and VM043 which were able to efficiently inhibit the growth of all mycobacteria were bearing a medium R^1^ octyl or decyl chain, and a short R^2^ propyl chain (Fig. 1). In terms of lipophilicity this translates into a narrow range of calculated log *P*_o/w_, determined using the iLOGP^69^ method from the SwissADME web tool,^70^ between 4.3 and 4.8 for these latter three compounds (Table S1†). Indeed, the insertion of a fluorine atom on the β-lactone ring of VM038 and VM039 (log *P*_o/w_ ∼ 4.12) slightly increased the lipophilicity of the fluorinated derivative VM043 (log *P*_o/w_ ∼ 4.31). This fluorine will also affect the electrophilic nature of the carbonyl, making it even more reactive and sensitive to nucleophilic attack by enzymes with nucleophilic residues, thus resulting in a significantly improved antibacterial activity for VM043 as compared to the parent molecules. Finally, switching from a β-lactone core (VM038 and VM039, and VM040 and VM041) to a β-lactam core (VM057 and VM058, and VM056, respectively) led to a partial improvement in antibacterial properties against *M. abscessus* S.

In summary, while VM025, VM026 and VM043 are active against all three mycobacterial strains, VM043 is the best growth inhibitor with MIC_50_/MIC_90_ values very similar to those of **Orlistat**.^45,46^ As already discussed in the case of others families of mycobacterial growth inhibitors,^28,54^ the reason for such differences in β-lactones activity against *M. tuberculosis* compared to *M. abscessus* S and R variants still remains unclear and would require further study. However, differences in membrane composition and permeability between these three mycobacterial species are likely to play a key role in this phenotype.

### 3.3. Synthesis of β-lactone activity-based probes

**Scheme 5:**
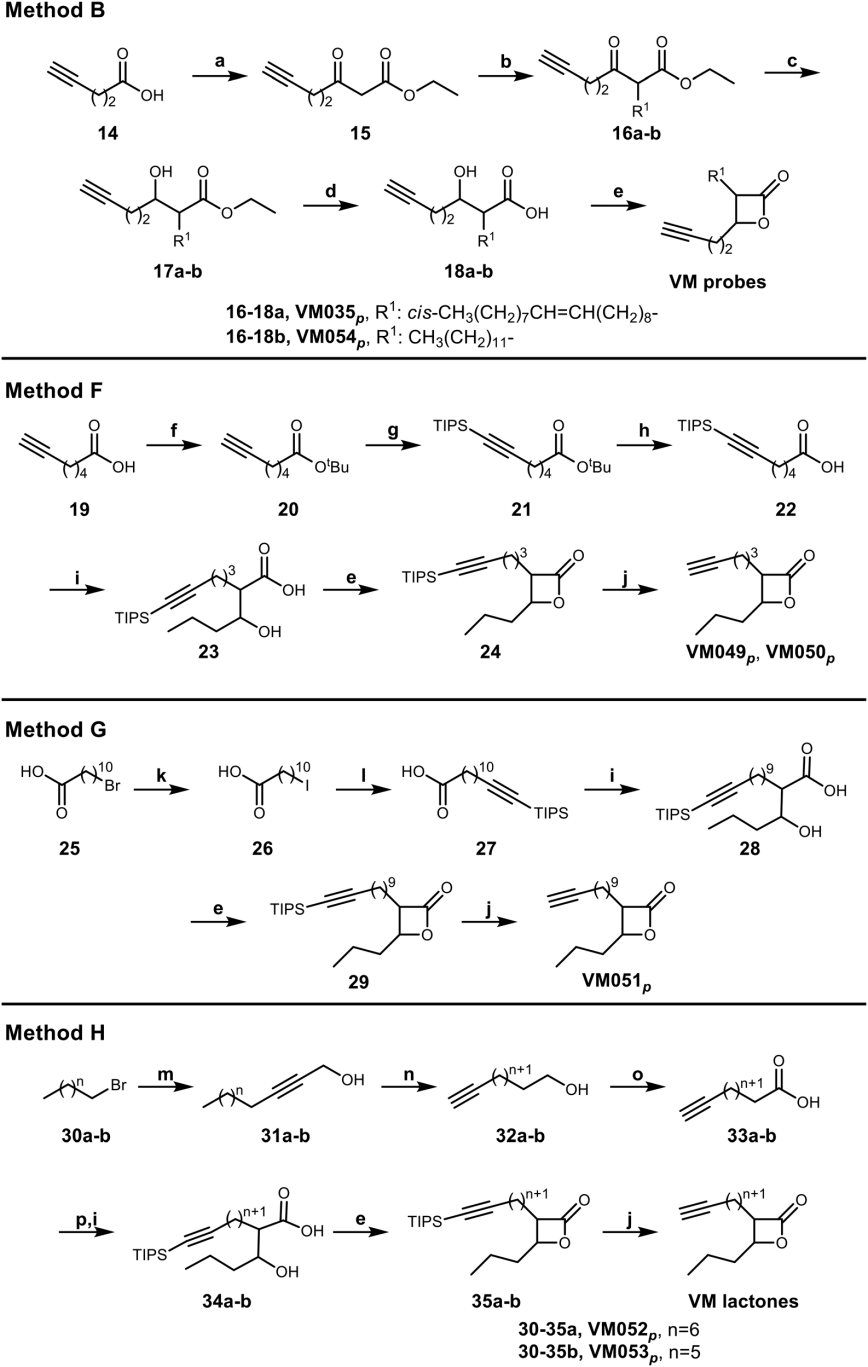
Synthesis of β-lactone probes bearing a terminal alkyne. Reagents and conditions: method B. (a) i) CDI, dry THF, r.t., 6 h; ii) MgCl_2_, EtOCOCH_2_COOK, r.t., 18 h; iii) aq. HCl 1 N, 82%; (b) K_2_CO_3_, R^1^I, acetone/DMF, reflux, 18 h, 76–83%; (c) NaBH_4_, EtOH, 0 °C 30 min, then r.t. 3 h, 69–75%; (d) NaOH 1 N, 1,4-dioxane, r.t., 16 h, 30–87%; (e) *p*-TsCl, dry pyridine, 0 °C, 1 h, then 4 °C, 3 days, 40–71%. Method F. (f) *t*-BuOH, DMAP, dry DCM, DCC, 75%; (g) i) *n*-BuLi, dry THF; ii) TIPS-Cl, 77%; (h) 15% TFA in DCM, 75%; (i) i) LDA (prepared *in situ* from (i-Pr)_2_NH and *n*-BuLi), dry THF; ii) butyraldehyde, dry THF, 0 °C to r.t., 66–67%; (j) TBAF, dry THF, 65–100%. Method G. (k) NaI, acetone, 95%; (l) *n*-BuLi, TIPS-acetylene, dry THF, −78 °C, 62%. Method H. (m) Propargyl alcohol, *n*-BuLi, THF/HMPA, −78 °C to r.t., 83–84%; (n) NaH, 1,2-ethylenediamine, 0 °C to 70 °C, 65–66%; (o) Jones reagent, acetone, 0 °C, 81%; (p) i) *n*-BuLi, dry THF; ii) TIPS-Cl, iii) K_2_CO_3_, THF/MeOH/H_2_O 3 : 1 : 1 (v/v/v), 38–64%.

**Scheme 6:**
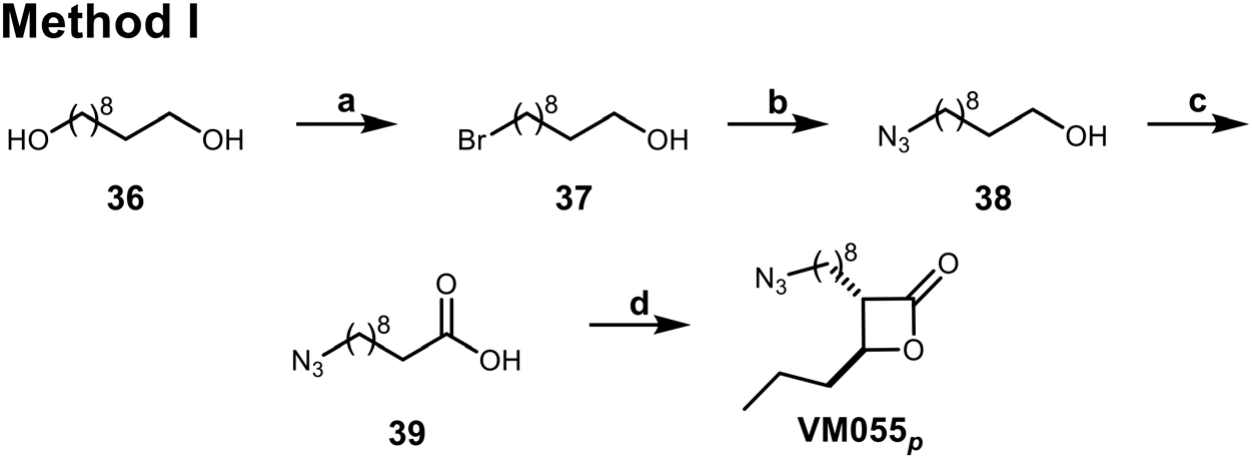
Synthesis of β-lactone probes bearing a terminal azide (method I). Reagents and conditions: (a) conc. HBr, toluene, reflux, 68%; (b) TBAB, NaN_3_, benzene, reflux, 95%; (c) Jones reagent, acetone, 0 °C, 71%; (d) i) LDA (prepared *in situ* from (i-Pr)_2_NH and *n*-BuLi), dry THF; ii) butyraldehyde, dry THF, 0 °C to r.t.; iii) column chromatography; iv) *p*-TsCl, dry pyridine, 0 °C to 4 °C, 12%.

Having established the promising antibacterial activity of VM025, VM026 and VM043 against *M. tuberculosis* mc^2^6230 and more especially *M. abscessus*, we further aimed at identifying their target enzymes using click-chemistry activity-based protein profiling (CC-ABPP) approach.^26,71–74^ Considering the covalent mechanism of action attributed to **Orlistat**^34,42^ and related β-lactone derivatives,^72^ a series of eight new molecules were synthesized by substituting the R^1^ or R^2^ alkyl chain with a terminal alkyne or azide group, to provide a panel of new activity-based probes (ABP), *i.e.*, VM035_*p*_, and VM049_*p*_ to VM055_*p*_ probes, that would be capable of retaining the antibacterial activity of their respective parent molecules (Schemes 5 and 6 and Table 2).

**Table 2:** Antibacterial activities of β-lactone probes against *M. tuberculosis* mc^2^6230 and *M. abscessus* S and R variants^a^

Compounds		MIC_50_/MIC_90_^a^ (μg mL^−1^)		
		*M. abscessus* CIP 104536^T^		*M. tuberculosis* mc^2^6230
		Smooth	Rough	
VM035_*p*_	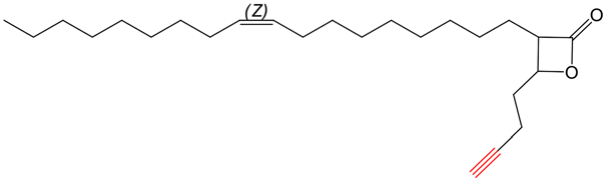	>100	>100	>100
(= VM028 probe)		(>100)	(>100)	(>100)
VM049_*p*_	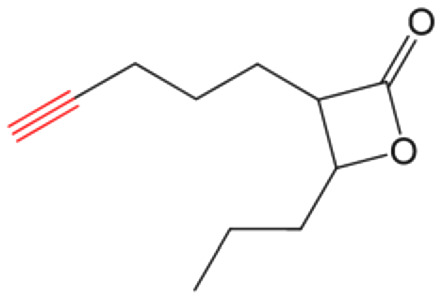	>100	>100	>100
(= VM019 probe)		(>100)	(>100)	(65.2 ± 0.93/67.2 ± 0.61)
VM050_*p*_	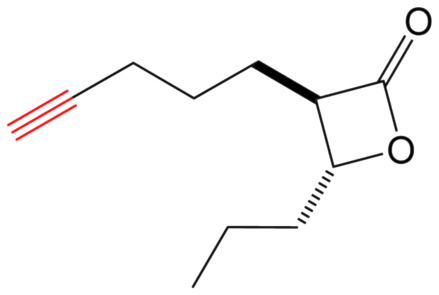	>100	>100	>100
(= VM019 probe)		(>100)	(>100)	(65.2 ± 0.93/67.2 ± 0.61)
VM051_*p*_	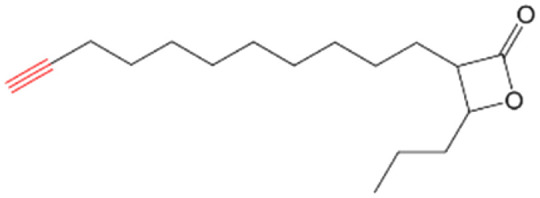	35.0 ± 7.1/>100	64.6 ± 0.1/>100	18.0 ± 0.2/23.9 ± 1.0
(= VM025 probe)		(49.9 ± 1.3/52.4 ± 2.5)	(27.1 ± 0.80/>100)	(63.9 ± 1.6/65.1 ± 1.5)
VM052_*p*_	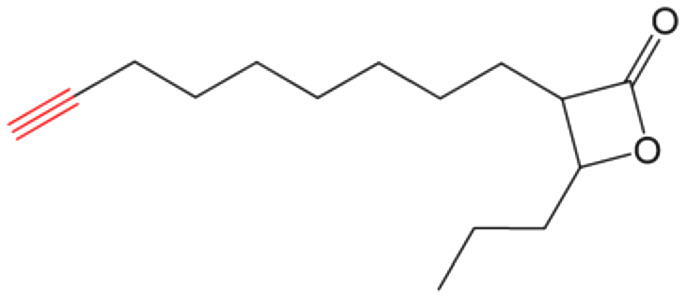	52.1 ± 5.5/>100	>100	35.2 ± 0.1/45.7 ± 0.2
(= VM038–VM039 probe)		(>100)	(>100)	(17.7–24.7/46.5–51.3)
(= VM043 probe)		(33.8 ± 1.2/49.2 ± 3.4)	(59.8 ± 5.6/88.9 ± 0.3)	(10.2 ± 3.6/40.1 ± 7.5)
VM053_*p*_	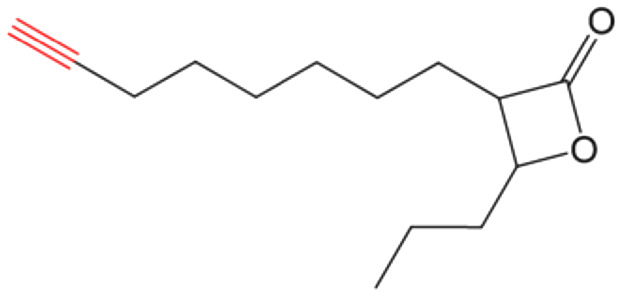	63.4 ± 1.3/>100	>100	35.1 ± 3.6/39.4 ± 6.4
(= VM038–VM039 probe)		(>100)	(>100)	(17.7–24.7/46.5–51.3)
(= VM043 probe)		(33.8 ± 1.2/49.2 ± 3.4)	(59.8 ± 5.6/88.9 ± 0.3)	(30.2 ± 1.3/45.9 ± 2.5)
VM054_*p*_	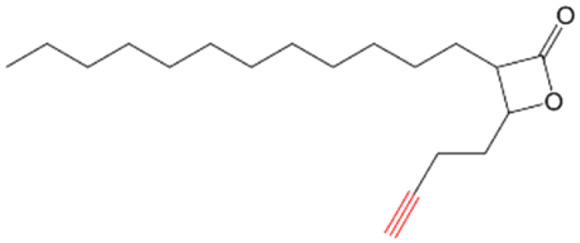	>100	>100	18.5 ± 0.8/24.5 ± 1.0
(= VM040–VM041 probe)		(>100)	(>100)	(9.6–17.4/29.1–44.2)
VM055_*p*_	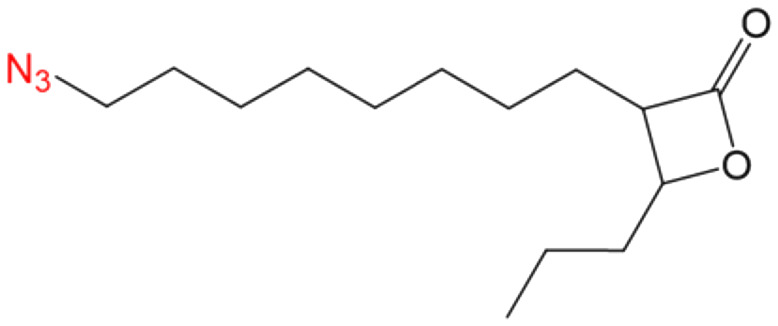	39.0 ± 4.5/>100	55.7 ± 3.3/>100	34.2 ± 1.5/52.7 ± 1.4
(= VM038–VM039 probe)		(>100)	(>100)	(17.7–24.7/46.5–51.3)
(= VM043 probe)		(33.8 ± 1.2/49.2 ± 3.4)	(59.8 ± 5.6/88.9 ± 0.3)	(30.2 ± 1.3/45.9 ± 2.5)

a Minimum inhibitory concentration leading to 50% or 90% growth inhibition (MIC_50_/MIC_90_) as determined by the resazurin microtiter assay (REMA). All values are expressed as mean ± SD of three independent assays. The MIC values of the parent molecules derived from Table 1 are shown in parentheses.

For the synthesis of the β-lactones ABP that would be tested in CC-ABPP, several different synthetic routes were established (Scheme 5). The previous Method B was first applied on 4-pentynoic acid 14 as starting material following the steps previously presented in Scheme 2. Reaction of 14 with CDI and monoethyl malonic acid magnesium salt afforded the β-keto ester 15. Then, substitution with K_2_CO_3_ as base and the appropriate alkyl iodide followed by NaBH_4_ reduction and hydrolysis, led to the β-hydroxy acids 18. Finally, β-lactone ring formation using *p*-TsCl in dry pyridine produced the final VM035_*p*_ and VM054_*p*_ probes that bear a terminal alkyne at the β-position of the ring. Methods F–H were further designed to synthesize β-lactones that bear the terminal alkyne at the α-position of the ring. Method F utilizes commercially available 6-heptynoic acid 19 that was protected as a *tert*-butyl ester at the carboxyl end using DCC, catalytic DMAP and *tert*-butanol. Then, a TIPS protecting group was added with *n*-BuLi and TIPS-Cl at the terminal alkyne for protection, followed by TFA deprotection of the ester to yield the free acid 22. With the terminal alkyne group protected, the acid undergoes an aldol reaction using LDA and butyraldehyde, as previously described in Scheme 1, followed by cyclization with *p*-TsCl in dry pyridine. The final step of this synthesis is the deprotection of the terminal alkyne using TBAF in dry THF for the production of the diastereomeric mixture of β-lactone VM049_*p*_ and the racemic *trans*-mixture of VM050_*p*_.

In method G, an ω-bromo carboxylic acid may be used. Here, 11-bromoundecanoic acid 25 is transformed into the corresponding 11-iodoundecanoic acid 26 using NaI in acetone, that is then used to a substitution reaction to TIPS-acetylene using *n*-BuLi in dry THF and HMPA, to yield the corresponding TIPS protected tridec-12-ynoic acid 27. This acid is then used in an aldol reaction, cyclization and final deprotection, as described in the above-mentioned method B to yield β-lactone VM051_*p*_.

All of the above-mentioned methods used commercially ω-alkynoic acids or ω-bromo acids as starting materials that are expensive and only few are commercially available. To that end, we designed a synthetic procedure (method H) starting from any bromoalkane and propargyl alcohol that would lead to any ω-alkynoic acid using inexpensive starting materials. Indeed, starting from 1-bromooctane or 1-bromononane 30 using propargyl alcohol and *n*-BuLi in dry THF and HMPA, the corresponding internal alkynyl alcohols 31 were prepared. A Zipper reaction^75^ using NaH and ethylenediamine moved the triple bond to the ω-end of the alcohol 32 and subsequent oxidation using Jones reagent in acetone yielded the desired terminal alkyne carboxylic acids 33. Protection of the alkyne with TIPS group, aldol reaction with butyraldehyde, cyclization and finally TIPS deprotection produced the β-lactones VM052_*p*_ and VM053_*p*_.

For the terminal azide β-lactone probes, we started from 1,10-decanediol 36 which was monobrominated using HBr in toluene under reflux (Scheme 6, method I). Then, the bromine was substituted by the azide group using NaN_3_ and TBAB in benzene, followed by Jones oxidation, aldol reaction and cyclization to yield the terminal azide β-lactone VM055_*p*_.

### 3.4. Activity-based protein profiling (ABPP) approach for targets identification

The antimicrobial potency of the synthesized β-lactone probes [VM035_*p*_, VM049_*p*_ to VM055_*p*_] was first evaluated against *M. tuberculosis* mc^2^6230 and *M. abscessus* S and R variants (Table 2). Partial loss of activity was observed for VM049_*p*_ and VM050_*p*_ (*i.e.*, pentynyl version of VM019), and even no activity for VM035_*p*_ (*i.e.*, butynyl version of VM028) as compared to unmarked parent molecules (Table 2). On the other hand, VM054_*p*_ (*i.e.*, pentynyl version of VM040 and VM041) bearing the alkyne on the alkyl chain in β-position, and VM051_*p*_ (*i.e.*, undecynyl version of VM025) retained comparable activity to their parent molecules against *M. tuberculosis* mc^2^6230. The same trend was observed with VM052_*p*_ and VM053_*p*_, the nonyl and octyl derivatives, respectively, of VM038 and VM039, which gained some antibacterial activity against *M. abscessus* S while displaying similar activity to the parent β-lactones against *M. tuberculosis* mc^2^6230. Remarkably, the azide derivative VM055_*p*_ not only retained good antibacterial activities as the unmarked parent VM038 and VM039 molecules against *M. tuberculosis* mc^2^6230, but also showed same moderate antibacterial activity as VM043 against *M. abscessus* S and R variants despite the absence of the fluorine atom, with fold changes in MIC_50_/MIC_90_ of around ×0.9–1.2/>2.0.

Overall, these results indicate that the choice of the alkyl chain length for the incorporation of a terminal alkyne bond, as well as its location in either the R^1^ or R^2^ position of the lactone cycle, is of great importance to avoid major changes in the antibacterial activity of the resulting β-lactone ABPs.

According to these results, VM055_*p*_ proved to be the best inhibitor of *M. abscessus* S & R and *M. tuberculosis* mc^2^6230 growth among the eight β-lactone probes tested, with MICs very similar to those of its parent molecules. This probe was therefore a logical choice for target enzyme identification through CC-ABPP workflow.^26^

**Fig. 2:**
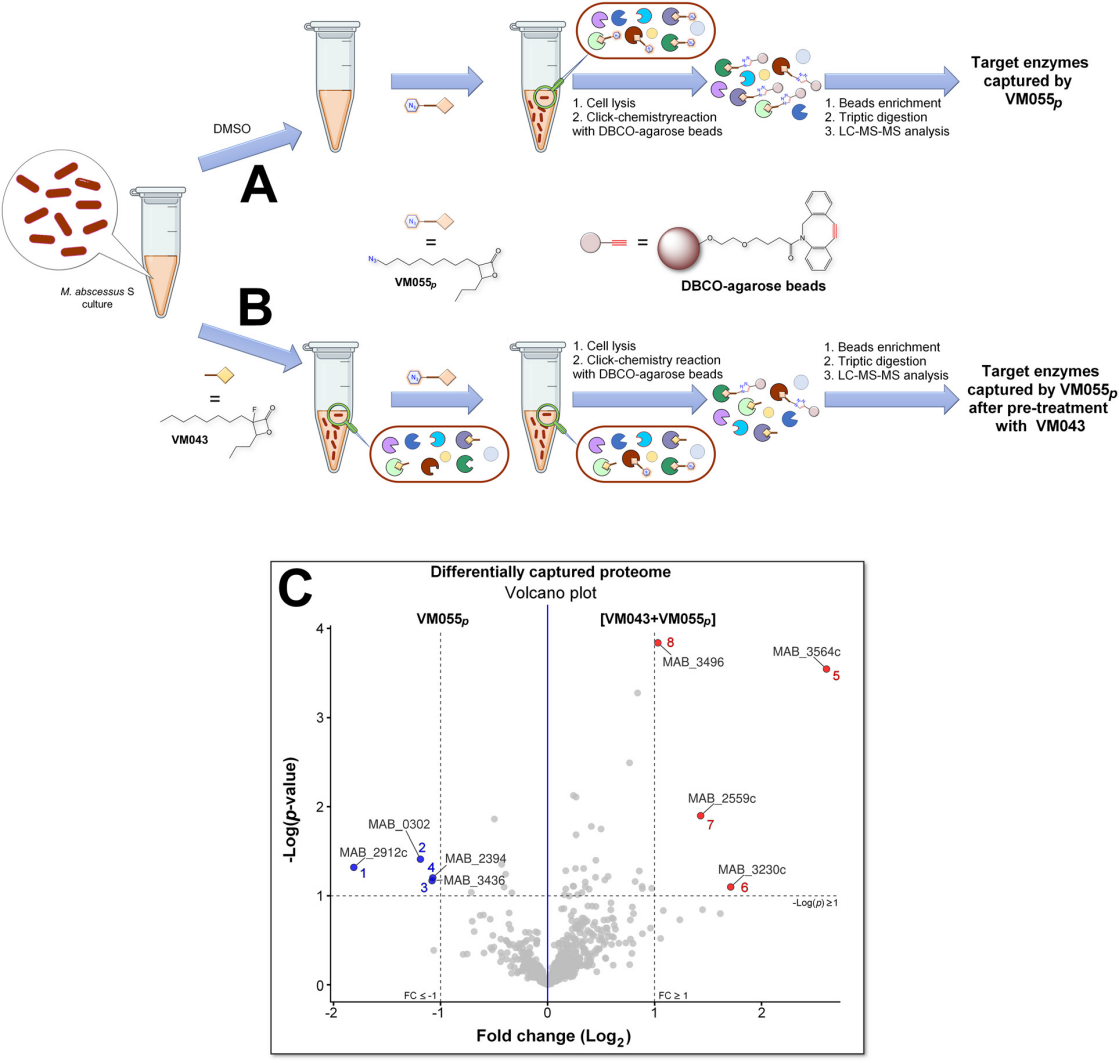
Click chemistry activity-based protein profiling on living *M. abscessus* S culture. CC-ABPP typical workflow for the identification of proteins covalently bound to (A) VM055_*p*_ or (B) significantly outcompeted by VM043. A mid-log phase culture of *M. abscessus* S was pre-treated with either DMSO or VM043, and then labelled with VM055_*p*_ probe. After cell lysis and strain-promoted azide–alkyne cycloaddition with DBCO-agarose beads, enriched samples were subjected to tryptic digestion. Tandem mass spectrometry analyses and subsequent differential peptides analysis allowed the identification of the target enzymes captured by VM055_*p*_ and those outcompeted by addition of VM043. (C) Volcano plot of the differential analysis of the labeled proteome of samples pre-incubated with VM043 inhibitor prior to VM055_*p*_ probe labeling *vs.*VM055_*p*_ probe-labeled samples only, and showing the significance of two-sample *t*-test (−log(*p*-value)) *versus* fold change (log_2_(LFQ normalized intensity in [VM043 + VM055_*p*_] *vs.*VM055_*p*_)) on the *y* and *x* axes, respectively (*n* = 3 biological independent experiments per group). The dashed lines indicate the threshold of *p*-value ≤ 0.1 and a fold change ≥ 1.0. Blue dots indicate VM055_*p*_ targets proteins that are outcompeted by addition of VM043; while red dots represent VM055_*p*_ targets proteins that were not inhibited by VM043. See also Tables S2–S6.†

To do so, exponential growth phase *M. abscessus* S cells were incubated with dimethyl sulfoxide (DMSO) and then treated with VM055_*p*_ ABP (Fig. 2A). Bacteria were lysed, and the probe-labeled proteins was subjected to strain-promoted azide–alkyne cycloaddition with DBCO-agarose beads.^58^ Indeed, such strained cyclooctynes can exclusively react with azide-tagged biomolecules to form a stable 1,2,3-triazole linkage without the need for copper catalyst.^76^ Here, the use of crosslinked agarose resin activated with DBCO functional groups, allows affinity enrichment of VM055_*p*_-labeled proteins. Following on-bead tryptic digestion, the resulting peptides were analyzed by liquid chromatography-tandem mass spectrometry (LC-MS/MS) and quantified by label-free quantitative analysis^59^ (Fig. 2). The comparative proteomic analysis between a control sample (*i.e.* DMSO-treated *M. abscessus* cells for non-specific binding to DBCO-agarose beads) and VM055_*p*_-treated samples led to the identification of 12 target enzymes, when applying *p*-value ≤ 0.05 and fold change ≥ 1.0 thresholds on the proteomics analysis results (Fig. 2A, Tables 3 and S2†).

**Table 3:** VM055_*p*_ target proteins identified in *M. abscessus* S culture by LC-ESI-MS/MS analysis^a^

Gene name	Protein name	*M. tuberculosis* orthologs				
		Rv number	Essentiality^b^	Location^c^	Activity/function	Functional category^d^
*MAB_3436*	Non-specific serine/threonine protein kinase	Rv0014c	*In vitro* growth	CF; WCL	Serine/threonine-protein kinase PknB	RP
*MAB_4551c*	Possible acylglycerol lipase	Rv0183		CM; CW; WCL	Monoacylglycerol lipase	LM
*MAB_1026c*	Hypothetical protein	Rv1926c		CF; CM; WCL	Immunogenic protein Mpt63	CW/CP
*MAB_0302*	Putative quinolone synthase	Rv1260		WCL	Probable oxidoreductase	IM/R
*MAB_4284c*	Hypothetical protein	Rv3514			PE-PGRS family protein PE_PGRS57	PE/PPE
*MAB_1895c*	Hypothetical protein	Rv0278c			PE-PGRS family protein PE_PGRS3	PE/PPE
*MAB_0974*	Hypothetical protein	Rv3876		CW; M; WCL	ESX-1 secretion-associated protein EspI	CW/CP
*MAB_3501*	Putative hydrolase	Rv3195			Conserved hypotheticals	—
*MAB_3566c*	Putative cyclase	—				—
*MAB_4924*	Hypothetical protein	Rv0040c		CL; WCL	Secreted proline rich protein Mtc28	CW/CP
*MAB_2394*	Hypothetical protein	Rv0910			Conserved hypotheticals	—
*MAB_2912c*	Probable aldolase	Rv0727c		M	Possible l-fuculose phosphate aldolase FucA	IM/R

a See also Tables S2 and S4.†

b From ref. 82 and 83.

c CF: culture filtrate; CW: cell wall; M: membrane fraction; WCL: whole cell lysate.

d CW/CP: cell wall/cell processes; IM/R: intermediary metabolism/respiration; IP: information pathways; LM: lipid metabolism; Pe/PPE: PE-PGRS family protein; RP: regulatory protein.

Even though at this stage no genes have been reported to be essential for *M. abscessus* growth by saturated transposon mutagenesis;^77^ their corresponding orthologs in *M. tuberculosis* H37Rv were nevertheless reported using the KEGG database^78,79^ and then cross-referenced with the Mycobrowser database^80^ to provide further information on their essentiality, activity and predicted location.^81^ Although three of the captured proteins are only conserved hypotheticals, the remaining nine ranged in their functional category from intermediary metabolism/respiration (2 proteins), lipid metabolism (1 protein), regulatory pathways (1 protein), cell wall/cell processes (3 proteins), and PE/PPE family proteins (2 proteins). Interestingly, these include the probable non-specific serine/threonine protein kinase MAB_3436 whose ortholog Rv0014c is annotated as essential for the *in vitro* growth of *M. tuberculosis*.^82,83^

Interestingly, several hydrolases were detected, including a putative hydrolase (MAB_3501/Rv3195), a putative cyclase (MAB_3566c), a probable aldolase (MAB_2912c/Rv0727c), a putative quinolone synthase (MAB_0302/Rv1260), and the monoacylglycerol lipase (MAB_4551c/Rv0183) which has previously been reported to be inhibited by **Orlistat**.^84^

Since all β-lactones share a similar mechanism of enzyme inhibition, resulting from the formation of a covalent bond between the β-lactone ring and the catalytic site of the enzyme,^33,34^ and since VM055_*p*_ showed comparable antibacterial activities to VM043 against both *M. abscessus* and *M. tuberculosis* mc^2^6230, we decided to also use this latter probe in an *in situ* competitive ABPP approach.^26^ The *M. abscessus* S culture was then pre-incubated with VM043 prior to treatment with the VM055_*p*_ probe and subsequent CC-ABPP experiment (Fig. 2B). To gain in statistically relevant results, a differential comparative proteomic analysis between the [VM043 + VM055_*p*_] captured proteome and that of the VM055_*p*_ probe alone was then performed. As shown in the resulting volcano plot (Fig. 2C), pre-incubation with VM043 significantly and selectively impaired (≥2-fold inhibition) the capture of four proteins by VM055_*p*_ (see blue dots in Fig. 2C), thereby suggesting that these latter enzymes are preferential targets of VM043*vs.*VM055_*p*_ (Table S3†). These included the probable aldolase MAB_2912c (Rv0727c), the putative quinolone synthase MAB_0302 (Rv1260), the uncharacterized protein MAB_2394 (Rv0910), and the non-specific serine/threonine protein kinase MAB_3436 (Rv0014c) (Tables 4 and S3†).

**Table 4:** VM055_*p*_ captured proteins that are either significantly outcompeted (#1–4) or not inhibited (#5–8) by VM043^a^

#	Protein IDs	Protein names	Gene names	[VM043 + VM055_*p*_] *vs.*VM055_*p*_	
				−log(*p*-value)	Fold change (log_2_)
1	B1MCL9	Probable aldolase	MAB_2912c	1.319	−1.810
2	B1MFK1	2-Heptyl-3-hydroxy-4(1*H*)-quinolone synthase	MAB_0302	1.411	−1.189
3	B1MEQ6	Non-specific serine/threonine protein kinase	MAB_3436	1.169	−1.080
4	B1MB54	Uncharacterized protein	MAB_2394	1.201	−1.073

5	B1MF33	Hypothetical dipeptidyl aminopeptidase	MAB_3564c	3.543	2.606
6	B1MDI7	SnoaL-like domain-containing protein	MAB_3230c	1.098	1.712
7	B1MBL9	Peptidyl-prolyl *cis*–*trans* isomerase	MAB_2559c	1.897	1.431
8	B1MEW6	Uncharacterized protein	MAB_3496	3.839	1.030

a See also Tables S2–S6.† The two lists of proteins are deduced from the differential volcano plot depicted in Fig. 2C.

Remarkably, this pre-incubation with VM043 also resulted in a statistically significant number of four enzymes being captured by the VM055_*p*_ probe (see red dots in Fig. 2C); namely a hypothetical dipeptidyl aminopeptidase MAB_3564c, a snoaL-like domain-containing protein MAB_3230c (Rv2910c), an uncharacterized protein MAB_3496, and the peptidyl-prolyl *cis*–*trans* isomerase MAB_2559c whose ortholog Rv3909 is annotated as essential for *M. tuberculosis* H37Rv *in vitro* growth (Fig. 2C, Tables 4 and S3†). Of particular interest, although these latter four enzymes were also captured by the VM055_*p*_ probe alone, they were however found to be below our *p*-value ≤ 0.05 and fold change ≥ 1.0 thresholds when compared to DMSO non-specific conditions (Table S3†).

These later findings thus suggest that the antibacterial activity of VM043 and VM055_*p*_ against *M. abscessus* growth should be due to the inhibition of a comparable spectrum of target enzymes by these two molecules, resulting in a similar anti-mycobacterial activity. Therefore, *a posteriori*, it is not surprising that pre-incubation of *M. abscessus* S cells with VM043 modifies the availability/accessibility/selectivity of these target enzymes with respect to VM055_*p*_, since proteins that were covalently inhibited by VM043 can no longer react and be captured by VM055_*p*_.

Moreover, these proteomic data also suggest that, as previously reported for two other families of multi-target inhibitors,^24,28,29,54,85^VM β-lactones would impair the growth of *M. abscessus* and *M. tuberculosis* by inhibiting of the activity of several enzymes involved in various important physiological processes. As a direct consequence, the likelihood of a strain developing resistance to such inhibitors would be very low, because resistant mutants should acquire multiple mutations in the same bacterial genome; making it difficult or impossible for the bacteria to adapt and survive.

### 3.5. Cell toxicity of beta-lactone derivatives

One of the keys to the success of *M. tuberculosis*^86,87^ as well as *M. abscessus*^7,16,88^ as lung pathogens is their ability to survive and replicate inside infected macrophages. As a result, new drugs must be able to inhibit only the intracellular growth of the pathogen and not be cytotoxic to host cells. In this regard, we determined for each β-lactone analog the concentration required to induce a 50% decrease in Raw264.7 murine macrophage cells viability,^89^*i.e.* CC_50_ (ref. 55) (Table S7†). Among the 38 tested derivatives, VM049_*p*_ and VM050_*p*_ bearing a short pentynyl R^2^ chain and the three β-lactam analogs VM056 to VM058 exhibited high toxicity towards Raw264.7 cells with CC_50_ values in the range of 30–70 μg mL^−1^, close to their MIC_90_. Otherwise, except VM039 which is slightly toxic (CC_50_ = 119 ± 4 μg mL^−1^), the other molecules had no cytotoxic effect against Raw264.7 macrophages at the highest concentration tested (125 μg mL^−1^).

### 3.6. Intramacrophagic susceptibility of *M. abscessus* to selected β-lactone derivatives

**Fig. 3:**
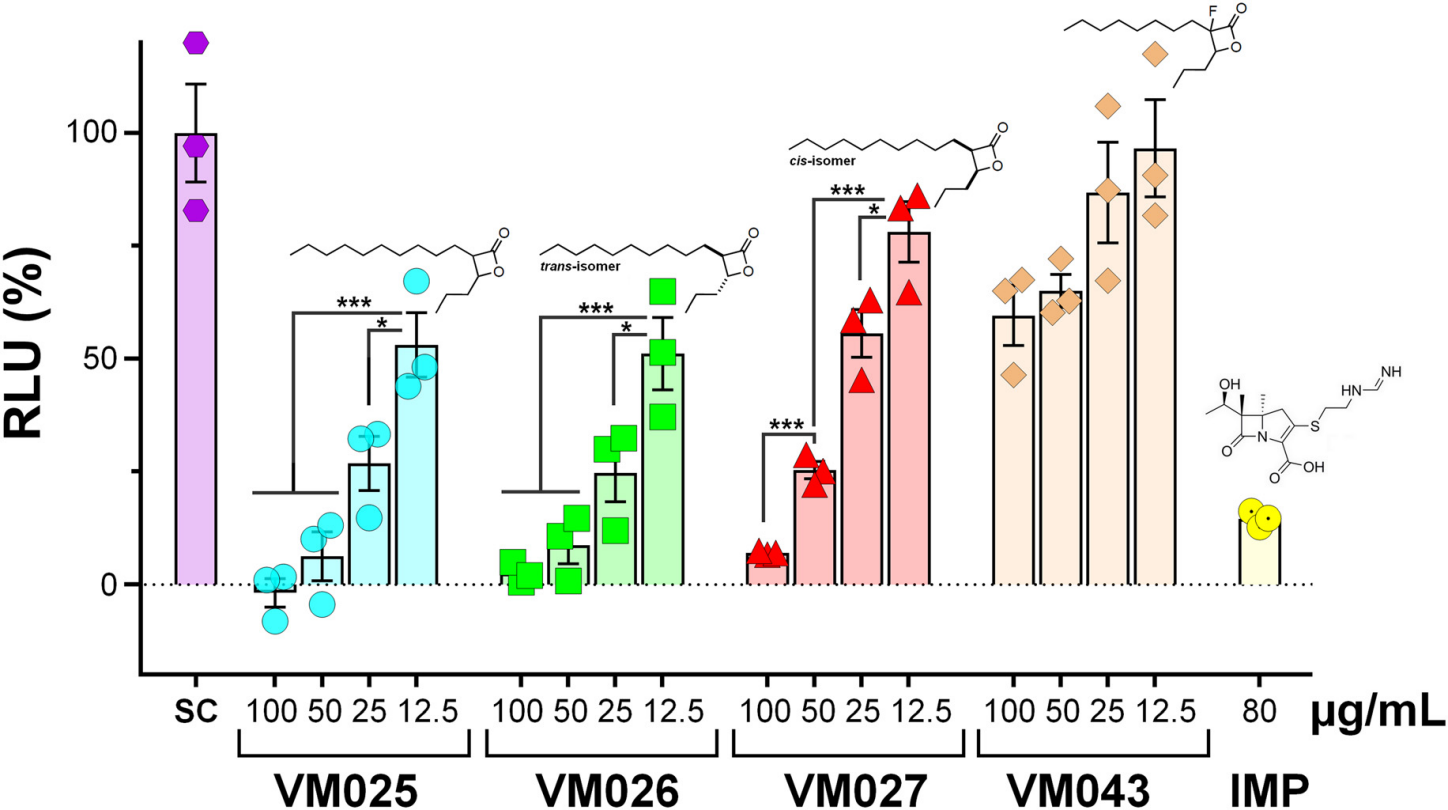
Intracellular activity of VM025, VM026, VM027, and VM043 as compared to imipenem (IMP). The activity of selected β-lactones on intracellular *M. abscessus* growth was tested in Raw264.7 murine macrophages. Cells were infected at a multiplicity of infection (MOI) of 10 with *M. abscessus* S-LuxG13 and treated with various concentrations of each inhibitor or IMP for 24 h. The viable mycobacteria were quantified by measurement of luminescence from luciferase-expressing *M. abscessus* S-LuxG13 within Raw264.7 macrophages. Untreated infected macrophages were used as control representing 100% of bacterial viability. Untreated infected macrophages were used as control representing 100% of bacterial viability. Results are shown as mean ± standard error of the mean (SEM) of three independent assays performed in triplicate. SC, solvent control (DMSO). ***, *p*-value <0.001. *, *p*-value <0.05. Statistical analysis was done using a Student's *t*-test.

Given these latter findings, we further investigated the ability of the β-lactones to inhibit the intra-macrophagic growth of *M. abscessus* S.^30,54,85,90,91^ To achieve this goal, Raw264.7 cells were infected with *M. abscessus* S at a multiplicity of infection (MOI) of 10, and then incubated for 24 h with selected β-lactone compounds or with imipenem (IMP; 80 μg mL^−1^ ∼ 9.5 × MIC_50Raw_) used as positive drug control for this intracellular killing assay.^54,85^ The selected inhibitors tested were VM025, VM026 (= *trans*-VM025), and VM043 which are the best inhibitors of *M. abscessus* S & R growth in broth medium. The inactive growth inhibitor VM027 (= *cis*-VM025), as well as the two probes of VM043, VM053_*p*_ and VM055_*p*_ which differ in their “clickable” group (alkyne *vs.* azide), were also included (Table 5 and Fig. 3).

**Table 5:** Intracellular antibacterial activities of the selected β-lactone derivatives against *M. abscessus* S-LuxG13 infected macrophages^a^

Compounds		CC_50_ (μg mL^−1^)	MIC_50Raw_ (μg mL^−1^)	SI
VM025	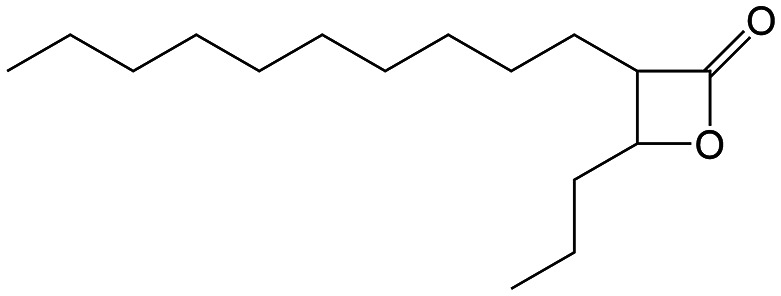	>125	13.6 ± 1.1	>9.2
VM026 = *trans*-VM025	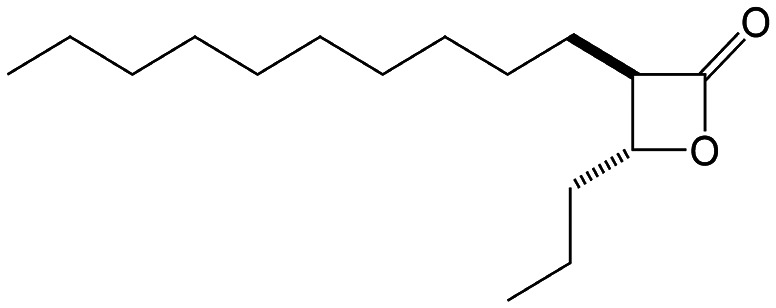	>125	12.9 ± 1.2	>9.7
VM027 = *cis*-VM025	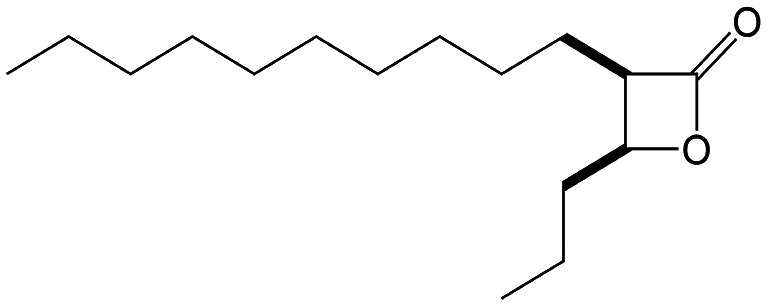	>125	27.9 ± 1.4	>4.5
VM043	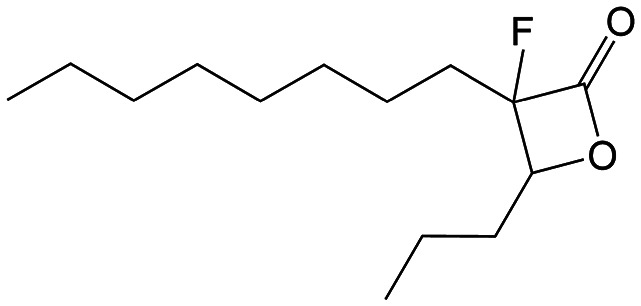	>125	>100	—
VM045	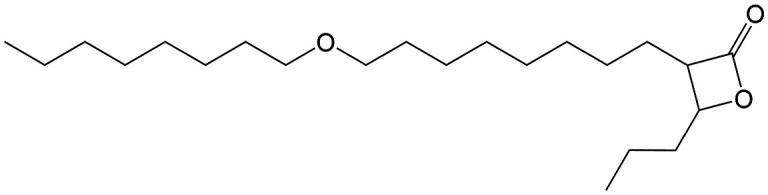	>125	18.9 ± 1.3	>6.5
VM046 = fluorinated-VM045	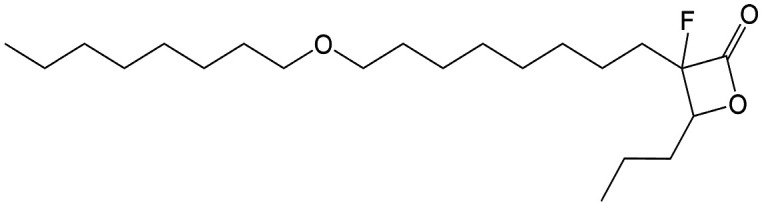	>125	>100	—
VM053_*p*_ = non-fluorinated VM043 probe	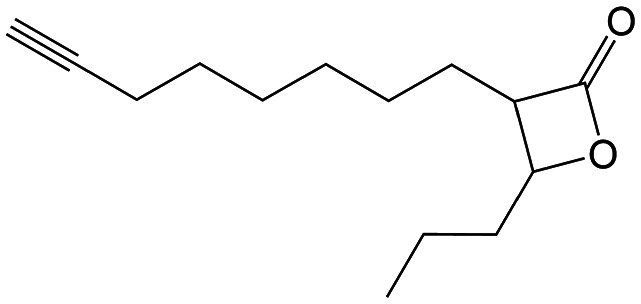	>125	48.6 ± 3.8	>2.6
VM055_*p*_ = non-fluorinated VM043 probe	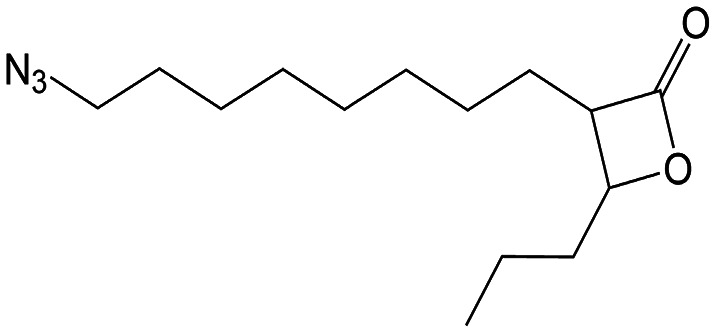	>125	24.9 ± 3.3	>5.0
**IMP** b			8.5 ± 1.4	—

a Experiments were performed as described in the Experimental section. CC_50_: compound concentration leading to 50% Raw264.7 macrophages toxicity. IC_50Raw_: minimal compound concentration leading to a 50% decrease in RLU count as compared to untreated cells. Raw264.7 macrophages were infected by *M. abscessus* S-LuxG13 at a MOI of 10, and further treated with each β-lactone or IMP for 24 h. The viable mycobacteria were quantified by measurement of luminescence from luciferase-expressing *M. abscessus* S-LuxG13 within Raw264.7 macrophages. Untreated infected macrophages were used as control representing 100% of bacterial viability. MIC_50Raw_ were calculated from curve fitting of RLU% as a function of the inhibitor concentration and are expressed as mean values of three independent assays.

b Data from ref. 54. IMP, imipenem. ND: not determined. SI, selectivity index, SI = CC_50_/MIC_50Raw_.

Among the compounds tested, VM025 and its *trans*-isomer VM026 exhibited promising antibacterial activity against intracellular *M. abscessus* growth, with an approximated MIC_50Raw_ of around 13 μg mL^−1^ which is only 1.5 times higher than that of IMP (MIC_50Raw_ = 8.5 μg mL^−1^) (Fig. 3 and Table 5). Interestingly, the *cis*-isomer VM027 which was not active against extracellular mycobacteria (Table 1), was however able to significantly decrease the intramacrophagic *M. abscessus* present 24 h post-infection, with a MIC_50Raw_ of around 28 μg mL^−1^. On the other hand, VM043 bearing a fluorine atom at the C-3 position of the β-lactone ring had no activity against the intracellular growth of *M. abscessus* (Fig. 3, Table 5 and Fig. S1†). The potential “negative” effect of the fluorine atom was further confirmed by testing VM046 and its non-fluorinated analog, VM045 (Table 5 and Fig. S1†). Indeed, although VM046 was inactive, VM045 was able to significantly inhibit the intracellular growth of *M. abscessus* with a MIC_50Raw_ of around 19 μg mL^−1^. Finally, the two non-fluorinated probes displayed quite good (MIC_50Raw_ = 24.9 μg mL^−1^ for VM055_*p*_) to moderate (MIC_50Raw_ = 48.6 μg mL^−1^ for VM053_*p*_) antibacterial activities against intracellular *M. abscessus* S growth (Table 5 and Fig. S1†).

The fact that MIC values determined in broth medium do not always correlate with the activity of the compounds against intracellular bacteria, is not new. In 2015, Vanderven *et al.* demonstrated that among 1359 hits tested in a screening assay against *M. tuberculosis*, only 141 compounds were able to inhibit both the extracellular and intracellular growth of the bacillus, and 132 inhibited bacterial replication inside macrophages with little or no inhibitory activity in broth medium.^92^ Another best example is the first-line anti-TB drug pyrazinamide^93^ which is inactive *in vitro* at neutral pH in conventional culture media, but displays potent activity against *M. tuberculosis* in an acidic environment (pH 5.5 or below) mimicking the endolysosomal pH of macrophages. Regarding multi-target inhibitors, such a discrepancy between extracellular and intracellular activities has also been reported and discussed in previous works with two other families of growth inhibitors of *M. tuberculosis* and *M. abscessus*; namely the oxadiazolone (OX) derivatives^28,85^ and the cyclipostins & cyclophostin (CyC) analogs.^29,30,32,54^ In particular, we have recently demonstrated that the CyCs which were active against intracellular *M. abscessus* growth accumulated in acidic compartments inside macrophage cells, and that this accumulation was essential for their delivery to mycobacteria-containing phagosomes and thus for their antimicrobial activity against intramacrophagic *M. abscessus*.^30^

Here, the fact that fluorine derivatives were fully inactive against intramacrophagic *M. abscessus*, as in the case of the best extracellular growth inhibitor VM043, may suggest a specific behavior or mode of action of these molecules inside infected macrophages. On the other hand, VM025 and VM026 which had a promising extracellular activity (MIC_90_ ∼54 μg mL^−1^) were 4 times more active against *M. abscessus* intracellular growth within macrophages (MIC_50Raw_ ∼13 μg mL^−1^). One hypothesis to explain this clear preference for intracellularly replicating mycobacteria could be that the corresponding target enzyme(s) of these inhibitors would be more accessible and/or vulnerable during the intracellular lifestyle of *M. abscessus* compared to its extracellular replication. However, a specific response of the infected macrophages resulting from the stress effect of these compounds and leading to bacterial clearance cannot be excluded.

Given the previously determined very low toxicity of the β-lactones toward Raw264.7 cells with CC_50_ > 125 μg mL^−1^, the selectivity index (SI = CC_50_/MIC_50Raw_) of the intracellular inhibitors on *M. abscessus vs.* Raw264.7 cells was calculated and found to be in a promising range from >2.6 and up to >9.0 (Table 4).

From these findings, it can be assumed that the observed inhibitory potency of the β-lactone derivatives i) might result from the inhibition of specific but most likely distinct mycobacterial target enzymes between intramacrophagic- *vs.* extracellularly-replicating mycobacteria; or ii) might reflect differences in the uptake and accumulation of the different compounds inside the macrophage. Overall, these results suggest that the non-fluorinated derivatives would be able to enter the macrophages and arrest bacterial replication without exhibiting significant toxicity to the host cell, with comparable antibacterial activity to imipenem.

## 4. Conclusion

In the present work, a new series of lipophilic compounds based on β-lactone-core were synthesized by varying the nature of the substituents on the lactone ring. Evaluation of their antibacterial activity first highlighted VM038, VM040, VM043 and VM045 as potential candidates against *M. tuberculosis*. With respect to *M. abscessus*, the MIC determination of this set of 30 derivatives provided VM025 and VM026, in addition to the latter VM043, as efficient inhibitors of both S and R variants. A competitive click-chemistry ABPP approach and comparative chemical proteomics with a newly synthesized customized activity-based probe VM055_*p*_ revealed several *M. abscessus* target enzymes of VM043, the best inhibitor of extracellular growth, compared to VM055_*p*_, thus confirming the multi-target nature of this family of molecules. When tested against intracellular bacteria, while VM043 was found inactive, VM025 & VM026 emerged as potent and promising inhibitors of intramacrophagic *M. abscessus* growth with MIC_50Raw_ values comparable to the standard antibiotic imipenem. This dual activity is of major importance as it may affect the different stages of the infection process.

Hence, thanks to their multitargeted covalent mechanism of action, our results underscore the added value of β-lactone probes. In particular, we anticipate that these probes would represent attractive tools against mycobacterial infections, and provide interesting insights into the different stages of the infection process that may lead to the arrest of two main mycobacterial pathogens, *M. tuberculosis* and/or *M. abscessus*. Identifying the proteins inactivated by our antibacterial activity-based probes would indeed reveal new potential targets for treating mycobacterial-related diseases, and contribute to background information for the development of new therapeutic strategies for elimination of either actively replicating or latent mycobacteria from infected individuals.

Further work to better understand the behavior of our β-lactone derivatives inside macrophage cells and consequently to elucidate their mode of action against intracellular *M. abscessus* is currently in progress.

## Abbreviations

ABP: Activity-based probe
ABPP: Activity-based protein profiling
AMK: Amikacin
CC_50_: Compound concentration leading to 50% of cell cytotoxicity
CC-ABPP: Click-chemistry activity-based protein profiling
*CF*
: Cystic fibrosis
IMP: Imipenem
MIC_50_/MIC_90_: Minimal inhibitory concentration leading to 50% and 90% of bacterial growth inhibition, respectively
MIC_50Raw_: Minimal inhibitory concentration leading to 50% of bacterial growth inhibition inside infected macrophages
NTM: Nontuberculous mycobacterial
REMA: Resazurin microtiter assay
RLU: Relative luminescence unit
SI: Selectivity index

## Author contributions

Thomas Francis: formal analysis – investigation – visualization – writing-review & editing. Christina Dedaki: formal analysis – investigations – resources – writing-review & editing. Phoebe Ananida-Dasenaki: investigations – resources. Dimitra Bolka: investigations – resources. Kanellos Albanis: investigations – resources. Filippos Foteinakis: investigations – resources. Julie Mezquida: formal analysis – investigation. Marie Hance: formal analysis – investigation. Alexandros Athanasoulis: investigations – resources. Anna-Krinio Papagiorgou: investigations – resources. Ioanna-Foteini Karampoula: investigations – resources. George Georgitsis: investigations – resources. Celia Jardin: data curation – formal analysis – investigation. Stéphane Audebert: data curation – formal analysis – investigation – writing-review & editing. Luc Camoin: data curation – writing-review & editing. Céline Crauste: writing-review & editing. Stéphane Canaan: writing-review & editing. Victoria Magrioti: conceptualization – formal analysis – methodology – project administration – resources – supervision – validation – visualization – writing-original draft – writing-review & editing. Jean-François Cavalier: conceptualization – data curation – formal analysis – investigation – methodology – project administration – supervision – validation – visualization – writing-original draft – writing-review & editing.

## Conflicts of interest

The authors declare that they have no known competing financial interests or personal relationships that could have appeared to influence the work reported in this paper.

## Supplementary Material

MD-016-D5MD00102A-s001

MD-016-D5MD00102A-s002

## Data Availability

All data generated or analyzed during this study are included in this published article and its supplementary information files. The mass spectrometry proteomics data are available online through the ProteomeXchange Consortium (http://www.proteomexchange.org) with the dataset identifiers PXD057836.
