# Supplementary material for: Synthesis and anti-mycobacterial activity of novel medium-chain β-lactone derivatives: a multi-target strategy to combat Mycobacterium abscessus
Source: RSC Med Chem. 2025 Apr 25;16(7):3251–72. doi: 10.1039/d5md00102a (PMC12101465; doi:10.1039/d5md00102a)
Supplement: MD-016-D5MD00102A-s001 [file MD-016-D5MD00102A-s001.pdf]

Supplementary Information (SI) for RSC Medicinal Chemistry.
This journal is © The Royal Society of Chemistry 2025

Synthesis and Anti-mycobacterial Activity of Novel Medium-chain β-Lactone Derivatives: A Multi-Target Strategy to combat *Mycobacterium abscessus*

Thomas Francis,[#,a] Christina Dedaki,[#,b] Phoebe Ananida-Dasenaki,[b] Dimitra Bolka,[b] Kanellos Albanis,[b] Filippos Foteinakis,[b] Julie Mezquida,[a] Marie Hance,[a] Alexandros Athanasoulis,[b] Anna-Krinio Papagiorgou,[b] Ioanna-Foteini Karampoula,[b] George Georgitsis,[b] Celia Jardin,[c] Stéphane Audebert,[c] Luc Camoin,[c] Céline Crauste,[d] Stéphane Canaan,[a] Victoria Magrioti,*[,b] Jean-François Cavalier*[,a]

[a] Aix-Marseille Univ., CNRS, LISM, Institut de Microbiologie de la Méditerranée FR3479, Marseille, France

[b] Department of Chemistry, National and Kapodistrian University of Athens, Panepistimiopolis, Athens, 15771, Greece

[c] Aix-Marseille Univ., INSERM, CNRS, Institut Paoli-Calmettes, CRCM, Marseille Protéomique, Marseille, France

[d] IBMM, Univ Montpellier, CNRS, ENSCM, Montpellier, France

* Corresponding authors: Jean-François Cavalier (jfcavalier@imm.cnrs.fr), and Victoria Magrioti (vmagriot@chem.uoa.gr)

[#] These authors have contributed equally to this work.

Contents

Detailled protocols S2

Supplementary **Table S1**: Code numbers, structures and cLogP of tested compounds S48

Supplementary **Figures S1**: intracellular activity of **VM044-046**, **VM053** & **VM055** S54

^{1}H, ^{13}C NMR spectra of new synthesized compounds S55

References S102

Detailed protocols

Biological evaluation

Antimycobacterial susceptibility testing

Antimycobacterial susceptibility testing was performed using the Middlebrook 7H9 broth microdilution method. MICs were determined in 96-well flat-bottom Nunclon Delta Surface microplates with lid (Thermo-Fisher Scientific, Illkirch, France) using the resazurin microtiter assay (REMA).[1-3] Briefly, log-phase bacterial cultures were diluted to a cell density of 5×10^6 CFU/mL in appropriate 7H9-S^{OADC} medium. Then 100 µL of the above inoculum were added to each well containing 100 µL of serial two-fold dilutions of each β-lactone analog to a final volume of 200 µL per well of the 96-well plate (final bacterial load of 5×10^5 CFU per well). Growth controls containing no inhibitor or with the DMSO vehicle (*i.e.*, bacteria only), inhibition controls containing 50 µg/mL kanamycin, and sterility controls (*i.e.*, medium only) without inoculation were also included. Microplates were incubated at 37 °C in a humidity chamber to prevent evaporation for 3-5 days (*M. abscessus*) or 10-14 days (*M. tuberculosis*). Then, 20 µL of a 0.025% (*w/v*) resazurin solution was added to each well, and the plates were incubated at 37 °C until color change from blue to pink or violet in the control well (*i.e.*, bacteria alone). Fluorescence units (FU) of the metabolite resorufin ($\lambda_{ex}/\lambda_{em}$ = 530/590 nm) were quantified using a Tecan Spark 10M™ multimode microplate reader (Tecan Group Ltd, France). Relative fluorescence units (RFU) were defined as: RFU% = (test well FU/mean FU of control *B* wells) × 100. MIC values were determined by fitting the RFU% sigmoidal dose–response curves in Kaleidagraph 4.2 software (Synergy Software). The lowest compound concentration leading to 50% and 90% inhibition of bacterial growth was defined as the MIC_{50} and MIC_{90}, respectively. Amikacin (AMK) (Euromedex, France) was used as reference drug. All experiments were performed independently at least three times.

Determination of cytotoxicity (resazurin assay)

The cytotoxicity of the new synthesized β-lactone analogs against eukaryotic cells was measured based on the reduction of resazurin as a value of cellular viability by metabolic activity.[2-4] Murine (Raw264.7) macrophages (American Type Culture Collection TIB-71) were cultured from a freezer stock in Dulbecco's modified Eagle medium (DMEM; Gibco) supplemented with 10% heat-inactivated fetal calf serum (FBS, Invitrogen) ($DMEM^{FBS}$). Cells were grown at 37 °C and 5% CO_2 to subconfluent concentrations. Then 1×10^5 cells/well were seeded in 96-well flat-bottom Nunclon Delta Surface microplates with lid (ThermoFisher Scientific) in a final volume of 200 µL per well, and incubated for additional 24 h. The medium was removed by aspiration, and 200 µL of serial two-fold dilution of each compound in $DMEM^{FBS}$ were then added to each well. After 24 h incubation, 20 µL of a 0.025% (*w/v*) resazurin solution was added to each well. Fluorescence was measured following a 4 h incubation at 37 °C and 5% CO_2 in the dark, by excitation at 530 nm and emission at 590 nm as described above, leading to relative metabolic activities. Addition of DMSO was used as 100% viability reference and addition of 0.2% Triton X-100 solution served as negative standard (0% viability). The compound

concentration leading to 50% macrophage cell death was defined as the CC_{50}[5-7]. All experiments were performed as three independent biological replicates.

Intramacrophage killing assay

Raw264.7 macrophages were grown in $DMEM^{FBS}$ medium at 37 °C and 5% CO_2 to subconfluent concentrations. Then 1×10^5 cells/well were seeded in 96-well flat-bottom Nunclon Delta Surface microplates with lid in a final volume of 200 µL per well and cultured for additional 24 h. The cells were infected with the *M. abscessus* S-LuxG13 strain at a multiplicity of infection (MOI) of 1:10, and incubated at 37 °C in the presence of 5% CO_2 for 4 h. Cells were washed three times with DMEM then refed with $DMEM^{FBS}$ supplemented with 250 µg/mL amikacin for 2 h. at 37 °C and 5% CO_2 to kill any extracellular bacteria; washed again three times with DMEM prior to the addition of 2-fold dilutions of the selected β-lactone analogs or imipenem (IMP; Euromedex, France) in $DMEM^{FBS}$ supplemented with 50 µg/mL amikacin (200 µL final volume). In each plate, negative controls consisting of amikacin (50 µg/mL) with 1% DMSO (*i.e.*, infected macrophages only); as well as positive controls containing amikacin (50 µg/mL) plus 80 µg/mL (*i.e.*, 9.5×MIC_{50Raw}) IMP were also included.[2, 8, 9] Plates were incubated for 24 h at 37°C, 5% CO_2.

Luminescence measurement was then used to check intracellular bacterial viability of *M. abscessus* S-LuxG13 strain[10, 11] following treatment with each selected β-lactone concentration. Infected macrophage cells were first washed three times with PBS, and luminescence was measured using a Tecan Spark 10M multimode microplate reader (Tecan Group Ltd, France). DMSO-treated infected macrophages corresponded as control representing 100% of bacterial viability. Intracellular MIC_{Raw} values were further determined by fitting the relative luminescence unit (RLU%) sigmoidal dose-response curves in Kaleidagraph 4.2 software (Synergy Software). The lowest compound concentration leading to 50% of RLU% was defined as the MIC_{50Raw}. Each experiment was performed as three independent biological replicates.

Copper-free click chemistry activity-based protein profiling[12-14]

Mid-log phase *M. abscessus* S culture ($OD_{600\ nm}$ ~ 1.5-2) in $7H9S^{OADC}$ was harvested by centrifugation at 5,000 rpm for 15 min, and bacterial cells were resuspended in 7H9-S^{OADC} at a final theoretical $OD_{600\ nm}$ of 20. One mL sample of this homogeneous bacterial suspension was incubated with **VM043** (122 µg/mL final concentration) or DMSO (control) at 37 °C for 4 h under shaking at 200 rpm. Bacteria were harvested (11,000 rpm, 4 °C, 15 min) and washed with 3× 500 µL PBS-Tween 80 (0.05%, *v/v*). Cell pellets were resuspended in 1 mL $7H9TG^{OADC}$ and then re-incubated at 37 °C with **VM055*p*** (54 µg/mL) or DMSO for 4 h at 200 rpm. After centrifugation (11,000 rpm, 4 °C, 15 min), the bacterial pellets were washed with 3 × 500 µL PBS-Tween 80 (0.05%, *v/v*), and resuspended in PBS supplemented with EDTA-free protease inhibitors (c*O*mplete Mini, EDTA-free; Roche, Mannheim, Germany) at a 1:1 (*w/v*) ratio. The bacterial cells were mixed with 200 µL of 0.1 mm diameter glass beads (BioSpec), and disrupted during 3 × 4 min of violent shaking, with ice cooling between each run, using Mini-Beadbeater-96 (BioSpec, Bartlesville, OK, USA). The resulting total lysates were cooled down in ice for 5 min and centrifuged at 4 °C and at 1300 rpm for 10 min to remove the cell debris and unbroken cells. The concentration of total proteins in the supernatants was determined *via* the Bradford method

and adjusted to a concentration of 1 mg/mL. Each sample (300 µL – 0.3 mg total proteins) was further subjected to copper-free azide-alkyne cycloaddition and enrichment with DBCO-agarose bead 50% slurry (Click Chemistry Tools, ref. 1034; prewashed 3 × 250 µL PBS-SDS (0.8%, *v/v*), centrifugation was performed at 1300 rpm for 2 min). First, 90 mg of urea is added to each total lysate (final concentration 5M) before transfer to the washed DBCO agarose beads, and incubation for 16 h at room temperature with rotary shaking (15 rpm). Beads were stringently washed following pull-down (500 µL each time): 2× PBS-SDS 0.4% (*v/v*), and 2× PBS-Urea 10M. All centrifugation steps were conducted at 1300 rpm for 2 min at room temperature. The beads containing bound, biotinylated proteins were resuspended in 30 µL PBS buffer pH 7.4, then snap frozen in liquid nitrogen and stored at −80 °C before mass spectrometry experiments.

Mass spectrometry analysis for protein identification and quantification

Mass spectrometry analysis. The beads were washed two times with water and then incubated with 1 mL TCEP (1 mM final concentration) in 50 mM TEAB, 20 min at 56 °C. After centrifugation (1,000 *g*, 5 min, 4 °C), the supernatant was discarded and replaced with 40 mM iodoacetamide in 50 mM TEAB for 30 min at RT in dark. Beads were then washed two times with 0.8% SDS in 50 mM TEAB, two times with 2M urea in 50 mM TEAB, and two times with 50 mM TEAB only. Proteins on beads were digested with 0.5 µg trypsin sequencing grade (Promega Inc.) in 50 mM TEAB for 16h at 37 °C. Peptides were extracted with 20% acetonitrile (2×150 µL) and dried under speed vacuum concentration. Peptides were further desalted on C18 Micro SpinColumns (Harvard Bioscience, Inc) dried and diluted in 15 µL water/acetonitrile/ (98/2, *v/v*) containing 0.05% TFA. 20% of each sample was analyzed twice by liquid chromatography (LC)-tandem MS (MS/MS) using a Q-Exactive plus Mass Spectrometer (Thermo Fisher Scientific, San Jose, CA) online with a nanoRSLC Ultimate 3000 chromatography system (Thermo Fisher Scientific, Sunnyvale, CA). First, peptides were concentrated and purified on a pre-column PepMap100 C18 (2 cm × 100 µm I.D, 100 Å pore size, 5 µm particle size) in solvent A (0.1% formic acid in 2% acetonitrile). In the second step, peptides were separated on a reverse phase LC EASY-Spray C18 column PepMap RSLC C18 (50 cm × 75 µm I.D, 100 Å pore size, 2 µm particle size) (Thermo Fisher) at 300 nL.min^{-1} flow rate and 40 °C. After column equilibration peptides were eluted from the analytical column by a three-step linear gradient (2.5%–27.5% solvent B [80% acetonitrile 0.1% formic acid in water] for 100 min, 27.5%–40% for 20 min and a last elution 40%–90% for 2 min). For peptide ionization in the EASY-Spray nanosource in front of the mass spectrometer, the spray voltage was set at 2.2 kV and the capillary temperature at 275°C. The mass spectrometer was used in data-independent acquisition (DIA) mode with the following parameters. First, MS spectra were acquired in the Orbitrap in the range of *m/z* 375–1500 at a FWHM resolution of 35,000 measured at 200 *m/z*. AGC target was set at $1×10^6$ with a Maximum Injection Time of 60 ms. MS2 spectra were acquired in the Orbitrap with a resolution of 17,500 after isolation of the parent ion in the quadrupole and fragmentation in the HCD cell under collision Energy of 27%. DIA parent ion range was from 400 to 1000 *m/z* divided into 24 *m/z* windows.

Protein identification and quantification. Relative intensity-based label-free quantification (LFQ) was processed using the DIA-NN 1.8 algorithm. Raw files were searched against the *Mycobacterium abscessus* database extracted from UniProt (UP000007137) extracted on the 15th of November 2021 and containing 4940

entries with the addition of a protein contaminant bank.[15] The following parameters were used for searches: (i) trypsin allowing cleavage before proline; (ii) one missed cleavage was allowed; (iii) cysteine carbamidomethylation (+57.02146) as a fixed modification and methionine oxidation (+15.99491) and N-terminal acetylation (+42.0106) as variable modifications; (iv) a maximum of one variable modification per peptide allowed; and (v) minimum peptide length was 7 amino acids and a maximum of 30 amino acids. The match between runs option was enabled. The precursor false discovery was set to 1%. DIA-NN parameters were set on Single-pass mode for the Neural Network classifier, Robust LC High precision for quantification strategy and RT-dependent mode for Cross-run normalization. The library was generated using a Smart profiling setup. The main output file from DIA-NN was further filtered at 1% FDR and LFQ intensity was calculated using our DIAgui package at 1% *q*-value (https://github.com/marseille-proteomique/DIAgui).[16] The statistical analysis was done with the Perseus program (version 1.6.15.0)[17] from the MaxQuant environment (www.maxquant.org). Quantifiable proteins were defined as those detected in above 70% of samples in at least one condition. Protein LFQ normalized intensities were base 2 logarithmized to obtain a normal distribution. Missing values were replaced using data imputation by randomly selecting from a normal distribution centered on the lower edge of the intensity values that simulate signals of low abundant proteins using default parameters (a downshift of 1.8 standard deviations and a width of 0.3 of the original distribution). To determine whether a given detected protein was specifically differential, a two-sample *t*-test was done using permutation-based FDR-controlled at 5 and employing 250 permutations. The *p*-value was adjusted using a scaling factor s0 with a value of 1[18].

The mass spectrometry proteomics data have been deposited to the ProteomeXchange Consortium (www.proteomexchange.org)[19] *via* the PRIDE partner repository[20] (https://www.ebi.ac.uk/pride/login) with the dataset identifiers PXD057836.

Chemistry

General methods of synthesis

Air- or moisture-sensitive reactions were carried out under an argon atmosphere. Thin-layer chromatography (TLC) was performed on Silica Gel 60 F254 aluminum plates. TLC spots were visualized with UV light and/or phosphomolybdic acid in EtOH and/or ninhydrin in EtOH. Chromatographic purification of products was accomplished using Silica Gel 60 (230-400 mesh). ^{1}H, ^{13}C and ^{19}F spectra were recorded on a Varian Mercury (200 MHz, 50 MHz and 188 MHz, respectively) or a Bruker Avance Neo (400 MHz, 101 MHz and 377 MHz, respectively) in $CDCl_3$, CD_3OD or $(CD_3)_2SO$, as indicated. Chemical shifts are given in ppm using solvent as an internal standard and coupling constants (J) in Hz. Peak multiplicities are described as follows: s, singlet, brs, broad singlet, d, doublet, t, triplet, q, quartet and m, multiplet. Electron spray ionization (ESI) mass spectra were recorded on a Finnigan, Surveyor MSQ Plus spectrometer. HRMS spectra were recorded on a Bruker Maxis Impact QTOF Spectrometer. All final products were characterized by ^{1}H, ^{13}C and ^{19}F (where applicable) and HRMS spectra and were found to be >95% pure. Dichloromethane was dried by standard procedures and stored over molecular sieves. Extra dry THF, dry pyridine and dry acetonitrile over molecular sieves were purchased from Thermo Scientific Chemicals. Compounds **1a-g**, **3a-c**, **8a-b**, **16**, **21**, **27**, **32a-b** and **38** were commercially available.

General Synthetic Procedures

General procedure I. Aldol reaction for the synthesis of α,β-substituted β-hydroxy acids.

To a stirring solution of diisopropylamine (3 mmol) in dry THF (2 mL), under argon at 0 °C, a solution of 1.6M *n*-BuLi in hexane (3 mmol) was slowly added *via* syringe and the solution of LDA was stirred at 0 °C for 10 min. The carboxylic acid (1 mmol) in dry THF (3 mL) was then added and the solution was stirred at 0 °C for 1 h. Then, the appropriate aldehyde (1.3 mmol) in dry THF (2 mL) was added and the solution was stirred at 0 °C for 1 h and at room temperature overnight. The solvent was removed under reduced pressure. The reaction mixture was acidified with 1N HCl and extracted with Et_2O (3×30 mL). The organic layers were combined, washed with brine (30 mL) and dried. The solvent was removed and the product was purified by column chromatography eluting with a gradient of $CHCl_3$/MeOH 97:3 to 95:5 (*v/v*).

General procedure II. β-Lactone cyclization using p-TsCl in pyridine.

To a stirring solution of the β-hydroxy acid (1 mmol) in dry pyridine (2 mL), under argon at 0 °C, *p*-toluenesulfonyl chloride (2 mmol) in dry pyridine (1 mL) was added slowly *via* syringe. The solution was stirred at 0 °C for 1 h and kept at 4 °C for 3 days. Then, Et_2O (30 mL) was added, and the organic layer was washed with 10% Na_2CO_3 (2×30 mL), 1N HCl (2×30 mL) and brine (30 mL). The organic layer was dried, and the solvent was removed in vacuo. The product was purified by column chromatography eluting with a gradient of hexane/EtOAc.

General procedure III. β-Lactone cyclization using EDC·HCl and DMAP.

In a flame-dried flask under argon, a solution of the β-hydroxy acid (1 mmol) in dry CH_2Cl_2 (18 mL) was added, followed by EDC·HCl (1.6 mmol) and DMAP (0.1 mmol) and the solution was stirred at r.t. for 3 days. CH_2Cl_2 (20 mL) and H_2O (20 mL) were added and then the organic layer was washed with brine (20 mL). The organic layer was dried, and the solvent was removed *in vacuo*. The product was purified by column chromatography eluting with a gradient of hexane/EtOAc.

General procedure IV. Late-stage fluorination of β-lactones.

In a flame-dried flask with a solution of diisopropylamine (1.6 mmol) in dry THF (1 mL), under argon at 0 ºC, a solution of *n*-BuLi 1.6 M in hexane (1.6 mmol, 1 mL) was slowly added *via* a syringe and the solution of LDA was stirred at 0 ºC for 10 min and then cooled at -78 °C. At -78 °C a solution of the β-lactone (1 mmol) in dry THF (7 mL) was added *via* a syringe and the solution was left stirring for 30 min at -78 °C. Then a solution of NFSI (2 mmol) in dry THF (2 mL) was added and the reaction was left to warm up to -15 °C and left stirring for additional 1.5 h at -15 °C. Then THF was removed *in vacuo,* EtOAc (30 mL) and 10% $NaHCO_3$ (30 mL) were added. The organic layer was washed with brine (30 mL), dried and the solvent was removed *in vacuo*. The product was purified by column chromatography eluting with a gradient of hexane/EtOAc.

General procedure V. Deprotection of TIPS-protected terminal alkyne β-lactones using TBAF.

In a flame-dried flask with a solution of the TIPS-protected terminal alkyne β-lactone (1 mmol) in dry THF (5 mL) under argon, TBAF (2 mmol) in dry THF (2 mL) was added and the mixture was left stirring for 3 h at r.t. The progress of the reaction was monitored by TLC. Once the starting material was consumed, saturated NH_4Cl was added (20 mL) and the crude product was extracted with Et_2O (3×20mL). The organic layer was washed with brine (20 mL), dried and the solvent was removed *in vacuo*. The product was purified by column chromatography eluting with a gradient of hexane/EtOAc or hexane/Et_2O.

General procedure VI. β-Lactam cyclization using Mukaiyama's reagent and Et_3N

In a flame-dried flask with a solution of the β-amino acid (1 mmol) in dry CH_3CN (10 mL), Mukaiyama's reagent (2-chloro-1-methylpyridinium iodide, 1.1 mmol) and Et_3N (2.3 mmol) were slowly added under stirring. The resulting mixture was heated to reflux for 4 h, then allowed to warm to r.t., and stirring continued for 16 h. Then, the solvent was removed *in vacuo* and the crude product was purified by column chromatography eluting with a gradient of hexane/EtOAc.

General procedure VII. Synthesis of β-keto esters using Masamune's method.

To a stirring solution of the appropriate acid (1 mmol) in dry THF under argon, 1,1′-carbonyl diimidazole (CDI) (1.2 mmol) was slowly added and the reaction mixture was left stirring for 6 h at room temperature. In another flask a solution of monoethyl malonic acid potassium (1.4 mmol) and $MgCl_2$ (1 mmol) in dry THF (2 mL) were stirred for 2 h. After the 6 h stirring of the first reaction mixture, the mixture of the second flask with monoethyl malonic acid magnesium salt was added to the first flask and was stirred for another 16 h. The solvent was removed under reduced pressure. The reaction mixture was dissolved in EtOAc (30 mL) and washed with 1N HCl (30 mL), then with brine (30 mL) and the organic layer was dried. The solvent was removed and the product was purified by column chromatography eluting with a gradient of hexane/EtOAc 95:5 to 9:1 (*v/v*).

General procedure VIII. Substitution of β-keto esters.

To a stirring solution of the appropriate β-keto ester (1 mmol) and bromide or iodide (1.1 mmol) in acetone (3.3 mL) and DMF (0.4 mL) under argon, K_2CO_3 (2.2 mmol) was added and the reaction mixture was left stirring at reflux under argon for 16 h. The solvent was removed under reduced pressure. The reaction mixture was dissolved in EtOAc (30 mL) and washed with H_2O (30 mL), the aqueous layer was washed with EtOAc (30 mL), the combined organic layers were washed with brine (30 mL) and then were dried. The solvent was removed and the product was purified by column chromatography eluting with a gradient of hexane/EtOAc 95:5 to 9:1 (*v/v*) or CH_2Cl_2/MeOH 98:2 to 95:5 (*v/v*).

General procedure IX. Reduction of α-substituted β-keto esters.

To a stirring solution of the appropriate α-substituted β-keto ester (1 mmol) in MeOH (2 mL) under argon and at 0 °C, $NaBH_4$ was added and the reaction mixture was left stirring for 1 h at 0 °C and at room temperature for another 2 h. Then the reaction was quenched with 1N HCl (1 mL) and stirred for another 5 min. CH_2Cl_2 (20 mL) and 1N HCl (20 mL) were added, the aqueous layer was washed twice with CH_2Cl_2 (2x20 mL), the combined organic layers were washed with brine (30 mL) and then were dried. The solvent was removed and the product

was purified by column chromatography eluting with a gradient of hexane/EtOAc 95:5 to 9:1 (*v/v*) or CH_2Cl_2/MeOH 98:2 to 95:5 (*v/v*).

General procedure X. Saponification of α-substituted β-hydroxy esters.

To a stirring solution of the appropriate α-substituted β-hydroxy ester (1 mmol) in THF (10 mL), 1 N NaOH aqueous solution (2 mmol) was added and the reaction mixture was left stirring for 16 h at room temperature. The solvent was removed under reduced pressure. The reaction mixture was dissolved in CH_2Cl_2 (30 mL) and washed with 1N HCl (20 mL), the aqueous layer was washed twice with CH_2Cl_2 (2×20 mL), the combined organic layers were washed with brine (30 mL) and then were dried. The solvent was removed and the product was purified by column chromatography eluting with a gradient of CH_2Cl_2/MeOH 98:2 to 95:5 (*v/v*).

General procedure XI. a-Fluorination of α-substituted β-keto esters.

To a stirring solution of the appropriate α-substituted β-keto ester (1 mmol) in dry THF (4.2 mL) at -20 °C under argon, NaH 60% (1.2 mmol) was added and the reaction mixture was left stirring at -20 °C under argon for 1 h. A solution of Selectfluor (1.2 mmol) in dry MeCN (8mL) was prepared in another flask under argon and was cooled at -20 °C. After the 1 h stirring of the first solution, the solution of Selectfluor was added -20 °C and was left stirring for another 2.5 h. Then the reaction was quenched with H_2O (1 mL) and stirred for 5 min. The solvent was removed under reduced pressure. The reaction mixture was dissolved in CH_2Cl_2 (30 mL) and washed with H_2O (30 mL). The organic layer was washed with brine (30 mL) and then was dried. The solvent was removed and the product was purified by column chromatography eluting with a gradient of hexane/ CH_2Cl_2 8:2 to 1:1 (*v/v*).

General procedure XII. β-Lactone ring opening by NaN_3.

To a stirring solution of the appropriate β-lactone (1 mmol) in DMF (4.8 mL), NaN_3 (1 mmol) was added and the reaction mixture was left stirring at 60 °C for 16 h. Then the reaction was quenched with H_2O (10 mL) and stirred for 5 min. The reaction mixture was diluted with CH_2Cl_2 (30 mL), the aqueous layer was washed with CH_2Cl_2 (30 mL), the combined organic layers were washed with brine (30 mL) and then were dried. The solvent was removed and the product was purified by column chromatography eluting with a gradient of hexane/EtOAc 95:5 to 9:1 (*v/v*).

General procedure XIII. Reduction of azide group to amine.

To a stirring solution of the appropriate β-azido acid (1 mmol) in MeOH (20 mL), a catalytic amount of 10% Pd/C was added, and the reaction mixture was left stirring under hydrogen atmosphere for 2 h. After filtration of the catalyst through Celite, the solvent of the filtrate was removed, and the product was purified by column chromatography eluting with a gradient of CH_2Cl_2/MeOH 8:2 to 7:3 (*v/v*).

General procedure XIV. Substitution of propargyl alcohol.

To a stirring solution of the propargyl alcohol (1.3 mmol) in dry THF/HMPA (4:1, 3.2 mL) at -78 °C under argon, *n*-BuLi 1.6 M solution (2.5 mmol) was added, and the reaction mixture was left stirring and warming up to -30 °C under argon for 1 h. At -30 °C a solution of the appropriate bromide (1 mmol) in dry THF (1 mL) was

added dropwise. The reaction mixture warmed up slowly to room temperature and left stirring at room temperature for 16 h. Then the reaction was quenched with sat. NH_4Cl (10 mL). The solvent was removed under reduced pressure. The reaction mixture was dissolved in EtOAc (30 mL) and washed twice with H_2O (2×30 mL). The combined aqueous layers were washed with EtOAc (30 mL). The combined organic layers were washed with brine (30 mL) and then were dried. The solvent was removed, and the product was purified by column chromatography eluting with a gradient of hexane/EtOAc 9:1 to 8:2 (*v/v*).

General procedure XIV. Zipper reaction for the synthesis of terminal alkyne alcohols.

Under argon, NaH 60% (6 mmol) was suspended in ethylendiamine (1.5 mL) at 0 °C and was slowly heated up to room temperature, stirred for 1 h and then warmed up to 60 °C and stirred for 2 h at this temperature. The mixture was cooled to 40 °C before the appropriate alkynyl alcohol (1 mmol) was added and stirred for 1 h at 60 °C. The reaction was quenched by carefully adding H_2O (10 ml) at 0 °C. The pH of the resulting mixture was adjusted to pH 1 by adding 1N HCl. Then the mixture was extracted with EtOAc (3×20 mL) and the combined organic layers were washed with 1N HCl (20 mL), brine (20 mL) and then dried. The solvent was removed, and the product was purified by column chromatography eluting with a gradient of hexane/EtOAc 95:5 to 8:2 (*v/v*).

General procedure XV. Jones oxidation of terminal alkyne alcohols to terminal alkyne acids.

To a stirring solution of the appropriate terminal alkyne alcohol (1 mmol) in acetone (8.5 mL) at 0 °C, Jones reagent (0.4 mL) was added, and the reaction mixture was left stirring at 0 °C for 2 h. The solvent was removed under reduced pressure. The reaction mixture was dissolved in Et_2O (30 mL) and washed twice with brine (2×30 mL). The combined aqueous layers were washed with Et_2O (30 mL). The combined organic layers were washed with brine (30 mL) and then were dried. The solvent was removed, and the product was purified by column chromatography eluting with a gradient of hexane/EtOAc 9:1 to 8:2 (*v/v*).

Synthesis of new β-lactone derivatives

(*Z*)-3-(Hexadec-7-en-1-yl)-4-methyloxetan-2-one (VM009)[3]

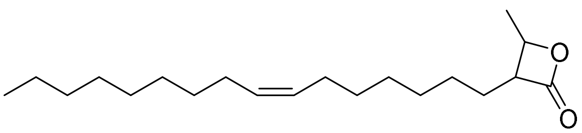

$C_{20}H_{36}O_2$
Exact Mass: 308,2715

Prepared according to **General procedure II** using **2a**; purified by column chromatography eluting with a gradient of hexane/EtOAc starting 97:3 to 95:5 (*v/v*). **VM009** gives rise to **VM013** and **VM020**. Isolated mixture of diastereomers after column chromatography (dr 7:3). Yield 36%; Colorless oil; ^{1}H NMR (200 MHz, $CDCl_3$) δ 5.44-5.23 (m, 2H), 4.81-4.66 (m, 0.3H), 4.46-4.33 (m, 0.7H), 3.67-3.52 (m, 0.3H), 3.22-3.10 (m, 0.7H), 2.11-1.90 (m, 4H), 1.89-1.60 (m, 2H), 1.56 (d, J = 6 Hz, 2.1H), 1.46 (d, J = 6 Hz, 0.9H), 1.42-1.13 (m, 20H), 0.89 (t, J = 7 Hz, 3H). ^{13}C NMR (50 MHz, $CDCl_3$) δ 172.0, 171.3, 130.1, 130.0, 129.6, 129.5, 74.6, 71.7, 57.6, 52.8,

31.9, 29.7, 29.6, 29.5, 29.3, 29.2, 29.1, 28.9, 27.7, 27.3, 27.2, 27.1, 26.8, 23.9, 22.7, 20.3, 15.6, 14.1. HRMS (ESI) $[M+Na]^+$: calcd for $C_{20}H_{36}NaO_2^+$ 331.2607, found 331.2611.

(±)-*trans* (*Z*)-3-(Hexadec-7-en-1-yl)-4-methyloxetan-2-one (VM013)[3]

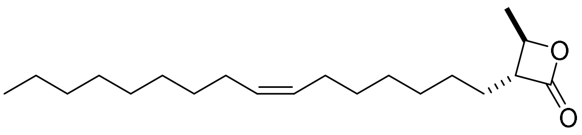

$C_{20}H_{36}O_2$
Exact Mass: 308,2715

Purified by 2nd column chromatography of **VM009** eluting with a gradient of hexane/EtOAc 98:2 to 95:5 (*v/v*). Colorless oil; ^{1}H NMR (200 MHz, $CDCl_3$) δ 5.44-5.23 (m, 2H), 4.40 (dq, J = 6 Hz, 4 Hz, 1H), 3.16 (ddd, J = 9 Hz, 7 Hz, 4 Hz, 1H), 2.11-1.90 (m, 4H), 1.89-1.60 (m, 2H), 1.56 (d, J = 6 Hz, 3H), 1.50-1.13 (m, 20H), 0.89 (t, J = 7 Hz, 3H). ^{13}C NMR (50 MHz, $CDCl_3$) δ 171.3, 130.1, 129.5, 74.6, 57.6, 31.9, 29.7, 29.5, 29.3, 29.2, 29.1, 28.9, 27.7, 27.2, 27.1, 26.8, 22.7, 20.3, 14.1. HRMS (ESI) $[M+Na]^+$: calcd for $C_{20}H_{36}NaO_2^+$ 331.2607, found 331.2610.

(±)-*cis* (*Z*)-3-(Hexadec-7-en-1-yl)-4-methyloxetan-2-one (VM020)

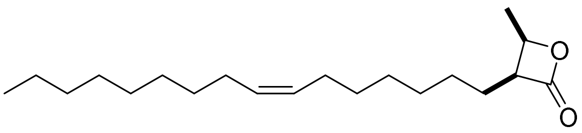

$C_{20}H_{36}O_2$
Exact Mass: 308,2715

Purified by 2nd column chromatography of **VM009** eluting with a gradient of hexane/EtOAc 98:2 to 95:5 (*v/v*). Colorless oil; ^{1}H NMR (200 MHz, $CDCl_3$) δ 5.44-5.23 (m, 2H), 4.81-4.66 (m, 1H), 3.67-3.52 (m, 1H), 2.11-1.90 (m, 4H, CH_2), 1.89-1.60 (m, 2H, CH_2), 1.46 (d, J = 6 Hz, 3H), 1.42-1.13 (m, 20H), 0.89 (t, J = 7 Hz, 3H). ^{13}C NMR (101 MHz, $CDCl_3$) δ 171.95, 130.14, 129.59, 71.71, 52.85, 31.91, 29.77, 29.62, 29.53, 29.33, 29.28, 28.95, 27.35, 27.23, 27.12, 23.91, 22.68, 15.64, 14.11. HRMS (ESI) $[M+Na]^+$: calcd for $C_{20}H_{36}NaO_2^+$ 331.2607, found 331.2610.

(*Z*)-4-Ethyl-3-(hexadec-7-en-1-yl)oxetan-2-one (VM010)[3]

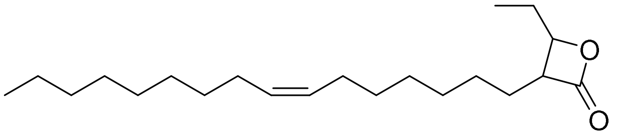

$C_{21}H_{38}O_2$
Exact Mass: 322,2872

Prepared according to **General procedure II** using **2b**; purified by column chromatography eluting with a gradient of hexane/EtOAc starting 97:3 to 95:5 (*v/v*). **VM010** gives rise to **VM017** and **VM018**. Isolated mixture of diastereomers after column chromatography (dr 6:4). Yield 52%; Colorless oil; ^{1}H NMR (200 MHz, $CDCl_3$) δ 5.42-5.20 (m, 2H), 4.49-4.35 (m, 0.4H), 4.19-4.06 (m, 0.6H), 3.64-3.47 (m, 0.4H), 3.20-3.05 (m, 0.6H), 2.09-1.89 (m, 4H), 1.89-1.55 (m, 4H), 1.53-1.10 (m, 20H), 1.08-0.92 (m, 3H), 0.85 (t, J = 7 Hz, 3H). ^{13}C NMR (50

MHz, $CDCl_3$) δ 172.1, 171.4, 130.0, 129.9, 129.5, 129.4, 79.0, 76.8, 55.6, 52.4, 31.8, 29.7, 29.5, 29.4, 29.2, 29.1, 28.8, 27.8, 27.5, 27.4, 27.1, 27.0, 26.9, 23.8, 23.4, 22.6, 14.0, 9.0. HRMS (ESI) $[M+Na]^+$: calcd for $C_{21}H_{38}NaO_2^+$ 345.2764, found 345.2764.

(±)-*trans* (*Z*)-4-Ethyl-3-(hexadec-7-en-1-yl)oxetan-2-one (VM017)

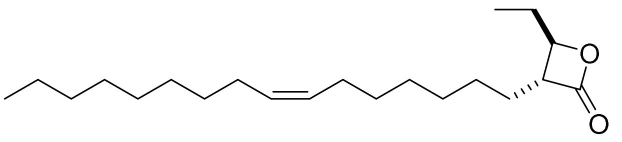

$C_{21}H_{38}O_2$
Exact Mass: 322,2872

Purified by 2nd column chromatography of **VM010** eluting with a gradient of hexane/EtOAc 98:2 to 95:5 (*v/v*). Colorless oil; ^{1}H NMR (200 MHz, $CDCl_3$) δ 5.45-5.24 (m, 2H), 4.23-4.11 (m, 1H), 3.23-3.11 (m, 1H), 2.12-1.93 (m, 4H), 1.93-1.65 (m, 4H), 1.53-1.15 (m, 20H), 1.02 (t, J = 7 Hz, 3H), 0.89 (t, J = 7 Hz, 3H). ^{13}C NMR (50 MHz, $CDCl_3$) δ 172.4, 130.3, 129.7, 79.4, 55.9, 32.1, 29.9, 29.8, 29.7, 29.5, 29.4, 29.1, 28.1, 27.7, 27.4, 27.3, 27.2, 22.9, 14.3, 9.3. HRMS (ESI) $[M+Na]^+$: calcd for $C_{21}H_{38}NaO_2^+$ 345.2764, found 345.2763.

(±)-*cis* (*Z*)-4-Ethyl-3-(hexadec-7-en-1-yl)oxetan-2-one (VM018)

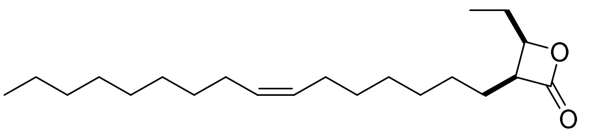

$C_{21}H_{38}O_2$
Exact Mass: 322,2872

Purified by 2nd column chromatography of **VM010** eluting with a gradient of hexane/EtOAc 98:2 to 95:5 (*v/v*). Colorless oil; ^{1}H NMR (200 MHz, $CDCl_3$) δ 5.45-5.23 (m, 2H), 4.49-4.35 (m, 1H), 3.64-3.47 (m, 1H), 2.09-1.89 (m, 4H), 1.87-1.55 (m, 4H), 1.53-1.10 (m, 20H), 1.07 (t, J = 7 Hz, 3H), 0.85 (t, J = 7 Hz, 3H). ^{13}C NMR (101 MHz, $CDCl_3$) δ 172.23, 130.10, 129.60, 76.96, 52.56, 31.91, 29.76, 29.61, 29.52, 29.32, 28.94, 27.60, 27.23, 27.11, 23.88, 23.53, 22.68, 14.10, 9.88. HRMS (ESI) $[M+Na]^+$: calcd for $C_{21}H_{38}NaO_2^+$ 345.2764, found 345.2764.

(*Z*)-3-(Hexadec-7-en-1-yl)-4-hexyloxetan-2-one (VM011)[3]

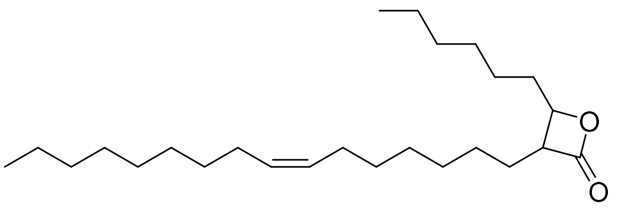

$C_{25}H_{46}O_2$
Exact Mass: 378,3498

Prepared according to **General procedure II** using **2c**; purified by column chromatography eluting with a gradient of hexane/EtOAc starting 97:3 to 95:5 (*v/v*). **VM011** gives rise to **VM022** and **VM024**. Isolated mixture of diastereomers after column chromatography (dr 6:4). Yield 56%; Colorless oil; ^{1}H NMR (200 MHz, $CDCl_3$)

δ 5.43-5.22 (m, 2H), 4.57-4.42 (m, 0.4H), 4.25-4.12 (m, 0.6H), 3.64-3.48 (m, 0.4H), 3.21-3.07 (m, 0.6H), 2.11-1.89 (m, 4H), 1.89-1.58 (m, 4H), 1.58-1.10 (m, 28H), 0.95-0.78 (m, 6H). ^{13}C NMR (50 MHz, $CDCl_3$) δ 172.1, 171.4, 130.0, 129.9, 129.5, 129.4, 78.0, 75.6, 56.0, 52.5, 34.4, 31.8, 31.5, 30.1, 29.7, 29.5, 29.4, 29.2, 29.1, 28.9, 28.8, 27.8, 27.5, 27.1, 27.0, 26.9, 25.4, 24.9, 23.8, 22.6, 22.4, 14.0, 13.9. HRMS (ESI) $[M+Na]^+$: calcd for $C_{25}H_{46}NaO_2^+$ 401.3390, found 401.3396.

(±)-*trans* (*Z*)-3-(Hexadec-7-en-1-yl)-4-hexyloxetan-2-one (VM022)

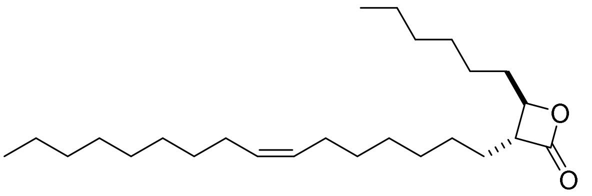

$C_{25}H_{46}O_2$
Exact Mass: 378,3498

Purified by 2nd column chromatography of **VM011** eluting with a gradient of hexane/EtOAc 98:2 to 95:5 (*v/v*). Colorless oil; ^{1}H NMR (200 MHz, $CDCl_3$) δ 5.43-5.22 (m, 2H), 4.25-4.12 (m, 1H), 3.21-3.07 (m, 1H), 2.11-1.89 (m, 4H), 1.89-1.58 (m, 4H), 1.53-1.12 (m, 28H), 0.95-0.78 (m, 6H). ^{13}C NMR (50 MHz, $CDCl_3$) δ 173.5, 130.3, 129.8, 78.3, 56.3, 34.6, 32.1, 31.8, 29.9, 29.8, 29.7, 29.5, 29.4, 29.1, 28.0, 27.4, 27.3, 27.1, 25.2, 22.9, 22.7, 14.3, 14.2. HRMS (ESI) $[M+Na]^+$: calcd for $C_{25}H_{46}NaO_2^+$ 401.3390, found 401.3394.

(±)-*cis* (*Z*)-3-(Hexadec-7-en-1-yl)-4-hexyloxetan-2-one (VM024)

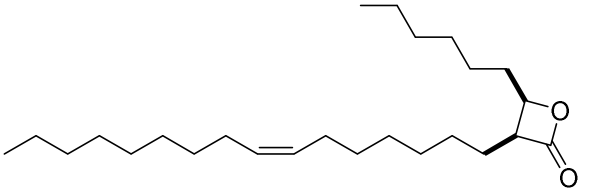

$C_{25}H_{46}O_2$
Exact Mass: 378,3498

Purified by 2nd column chromatography of **VM011** eluting with a gradient of hexane/EtOAc 98:2 to 95:5 (*v/v*). Colorless oil; ^{1}H NMR (200 MHz, $CDCl_3$) δ 5.43-5.22 (m, 2H), 4.57-4.42 (m, 1H), 3.64-3.48 (m, 1H), 2.11-1.89 (m, 4H), 1.89-1.48 (m, 6H), 1.48-1.12 (m, 26H, CH_2), 0.95-0.78 (m, 6H). ^{13}C NMR (101 MHz, $CDCl_3$) δ 172.30, 130.12, 129.61, 75.72, 52.66, 31.91, 31.61, 30.21, 29.77, 29.62, 29.53, 29.33, 29.31, 28.98, 28.95, 27.60, 27.23, 27.12, 25.52, 23.94, 22.68, 22.52, 14.11, 14.02. HRMS (ESI) $[M+Na]^+$: calcd for $C_{25}H_{46}NaO_2^+$ 401.3390, found 401.3391.

(*Z*)-3-(Hexadec-7-en-1-yl)-4-nonyloxetan-2-one (VM012)[3]

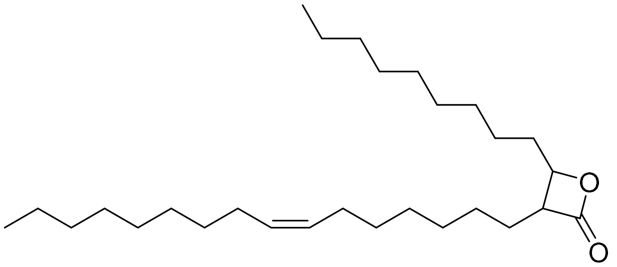

$C_{28}H_{52}O_2$
Exact Mass: 420,3967

Prepared according to **General procedure II** using **2d**; purified by column chromatography eluting with a gradient of hexane/EtOAc starting 97:3 to 95:5 (*v/v*). **VM012** gives rise to **VM021** and **VM023**. Isolated mixture of diastereomers after column chromatography (dr 7:3). Yield 63%; Colorless oil; ^{1}H NMR (200 MHz, $CDCl_3$) δ 5.44-5.23 (m, 2H), 4.59-4.45 (m, 0.3H), 4.26-4.13 (m, 0.7H), 3.65-3.50 (m, 0.3H), 3.22-3.07 (m, 0.7H), 2.14-1.91 (m, 4H), 1.91-1.56 (m, 4H), 1.56-1.10 (m, 34H), 0.89 (t, J = 7 Hz, 6H). ^{13}C NMR (50 MHz, $CDCl_3$) δ 172.2, 171.5, 130.0, 129.9, 129.5, 129.4, 78.0, 75.6, 56.0, 52.5, 34.4, 31.8, 31.7, 30.1, 29.7, 29.6, 29.5, 29.4, 29.3, 29.2, 29.1, 28.9, 27.8, 27.5, 27.1, 27.0, 26.9, 25.5, 25.0, 23.9, 22.6, 14.0. HRMS (ESI) [M+Na]$^+$: calcd for $C_{28}H_{52}NaO_2^+$ 443.3859, found 443.3861.

(±)-*trans* (*Z*)-3-(Hexadec-7-en-1-yl)-4-nonyloxetan-2-one (VM021)

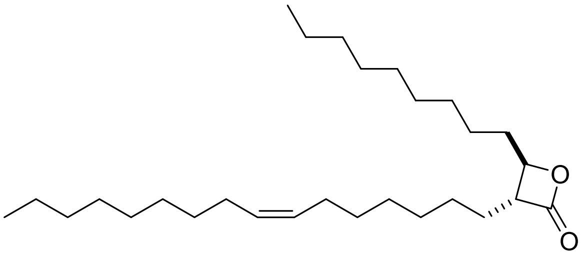

$C_{28}H_{52}O_2$
Exact Mass: 420,3967

Purified by 2nd column chromatography of **VM012** eluting with a gradient of hexane/EtOAc 98:2 to 95:5 (*v/v*). Colorless oil; ^{1}H NMR (200 MHz, $CDCl_3$) δ 5.44-5.23 (m, 2H), 4.26-4.15 (m, 1H), 3.22-3.10 (m, 1H), 2.14-1.91 (m, 4H), 1.91-1.56 (m, 6H), 1.56-1.10 (m, 32H), 0.89 (t, J = 7 Hz, 6H). ^{13}C NMR (50 MHz, $CDCl_3$) δ 172.8, 130.3, 129.8, 78.7, 56.3, 34.6, 32.1, 32.0, 29.9, 29.8, 29.7, 29.6, 29.5, 29.4, 29.1, 28.1, 27.4, 27.3, 27.1, 25.5, 22,8, 14.3. HRMS (ESI) [M+Na]$^+$: calcd for $C_{28}H_{52}NaO_2^+$ 443.3859, found 443.3863.

(±)-*cis* (*Z*)-3-(Hexadec-7-en-1-yl)-4-nonyloxetan-2-one (VM023)

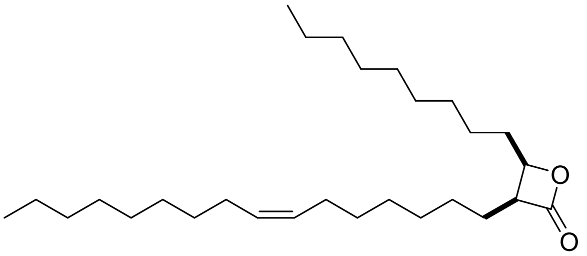

$C_{28}H_{52}O_2$
Exact Mass: 420,3967

Purified by 2nd column chromatography of **VM012** eluting with a gradient of hexane/EtOAc 98:2 to 95:5 (*v/v*). Colorless oil; ^{1}H NMR (200 MHz, $CDCl_3$) δ 5.44-5.23 (m, 2H), 4.60-4.46 (m, 1H), 3.67-3.52 (m, 1H), 2.14-1.91 (m, 4H), 1.87-1.48 (m, 4H), 1.48-1.10 (m, 34H), 0.89 (t, J = 7 Hz, 6H). ^{13}C NMR (101 MHz, $CDCl_3$) δ 172.27, 130.10, 129.60, 75.70, 52.65, 31.91, 31.87, 30.21, 29.77, 29.62, 29.53, 29.46, 29.43, 29.32, 29.26, 28.95, 27.60, 27.23, 27.12, 25.56, 23.94, 22.68, 22.66, 14.09. HRMS (ESI) $[M+Na]^+$: calcd for $C_{28}H_{52}NaO_2^+$ 443.3859, found 443.3861.

(±)-*trans* 3-Hexyl-4-propyloxetan-2-one (VM019)

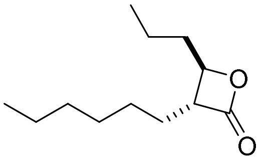

$C_{12}H_{22}O_2$
Exact Mass: 198,1620

Prepared according to **General procedure II** using **2e**; purified by column chromatography eluting with a gradient of hexane/EtOAc starting 97:3 to 95:5 (*v/v*) and then by 2nd column chromatography eluting with a gradient of hexane/EtOAc 98:2 to 95:5 (*v/v*). Yield 50%; Colorless oil; ^{1}H NMR (200 MHz, $CDCl_3$) δ 4.29-4.17 (m, 1H), 3.24-3.11 (m, 1H), 1.94-1.58 (m, 4H), 1.58-1.17 (m, 10H), 0.99 (t, J = 7 Hz, 3H), 0.89 (t, J = 7 Hz, 3H). ^{13}C NMR (50 MHz, $CDCl_3$) δ 171.9, 79.3, 61,0, 36.7, 31.7, 29.1, 28.0, 27.1, 22.7, 18.6, 14.2, 13.9. HRMS (ESI) $[M+Na]^+$: calcd for $C_{12}H_{22}NaO_2^+$ 221.1512, found 221.1510.

3-Decyl-4-propyloxetan-2-one (VM025)

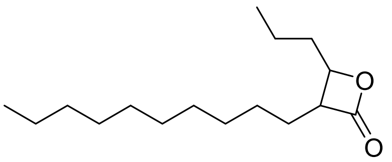

$C_{16}H_{30}O_2$
Exact Mass: 254,2246

Prepared according to **General procedure II** using **2f**; purified by column chromatography eluting with a gradient of hexane/EtOAc starting 97:3 to 95:5 (*v/v*). **VM025** gives rise to **VM026** and **VM027**. Isolated mixture of diastereomers after column chromatography (dr 1:1). Yield 70%; ^{1}H NMR (200 MHz, $CDCl_3$) δ 4.59-4.46 (m, 0.5H), 4.26-4.15 (m, 0.5H), 3.65-3.50 (m, 0.5H), 3.21-3.08 (m, 0.5H), 1.91-1.08 (m, 24H), 0.96 (t, J = 7 Hz, 3H), 0.86 (t, J = 7 Hz, 3H). ^{13}C NMR (50 MHz, $CDCl_3$) δ 172.5, 171.8, 78.1, 75.6, 56.3, 52.8, 36.7, 32.4, 32.1, 29.8, 29.7, 29.6, 29.5, 28.1, 27.8, 27.2, 24.1, 22.8, 19.1, 18.6, 14.3, 14.0, 13.9. HRMS (ESI) $[M+Na]^+$: calcd for $C_{16}H_{30}NaO_2^+$ 277.2138, found 277.2135.

(±)-*trans* 3-Decyl-4-propyloxetan-2-one (VM026)

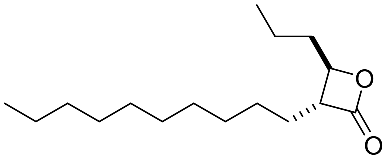

$C_{16}H_{30}O_2$
Exact Mass: 254,2246

Purified by 2nd column chromatography of **VM025** eluting with a gradient of hexane/EtOAc 98:2 to 95:5 (*v/v*). ^{1}H NMR (200 MHz, $CDCl_3$) δ 4.26-4.15 (m, 1H), 3.21-3.08 (m, 1H), 1.91-1.56 (m, 4H), 1.55-1.08 (m, 18H), 1.00-0.65 (m, 6H). ^{13}C NMR (50 MHz, $CDCl_3$) δ 172.2, 78.2, 56.4, 36.7, 32.1, 29.7, 29.5, 28.1, 27.2, 22.8, 18.6, 14.3, 14.0. HRMS (ESI) $[M+Na]^+$: calcd for $C_{16}H_{30}NaO_2^+$ 277.2138, found 277.2137.

(±)-*cis* 3-Decyl-4-propyloxetan-2-one (VM027)

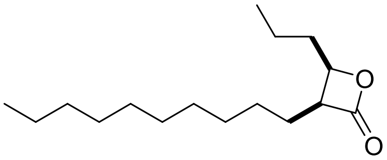

$C_{16}H_{30}O_2$
Exact Mass: 254,2246

Purified by 2nd column chromatography of **VM025** eluting with a gradient of hexane/EtOAc 98:2 to 95:5 (*v/v*). ^{1}H NMR (200 MHz, $CDCl_3$) δ 4.59-4.46 (m, 1H), 3.65-3.50 (m, 1H), 1.90-1.40 (m, 8H), 1.40-1.11 (m, 14H), 0.98 (t, J = 7 Hz, 3H), 0.88 (t, J = 7 Hz, 3H). ^{13}C NMR (50 MHz, $CDCl_3$) δ 171.8, 75.7, 52.8, 32.4, 32.0, 29.6, 29.5, 29.4, 29.3, 27.8, 24.1, 22.8, 19.0, 14.3, 14.0. HRMS (ESI) $[M+Na]^+$: calcd for $C_{16}H_{30}NaO_2^+$ 277.2138, found 277.2138.

(Z)-3-(Octadec-9-en-1-yl)-4-propyloxetan-2-one (VM028)

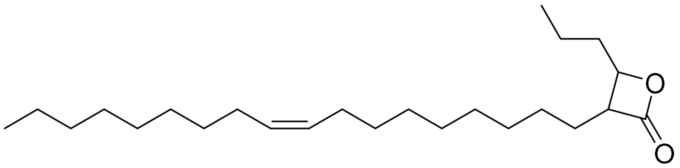

$C_{24}H_{44}O_2$
Exact Mass: 364,3341

Prepared according to **General procedure II** using **7a**; purified by column chromatography eluting with hexane/EtOAc 9:1 (*v/v*). **VM028** gives rise to **VM029** and **VM030**. Isolated mixture of diastereomers after column chromatography (dr 8:2). Yield 60%; Yellowish oil; ^{1}H NMR (200 MHz, $CDCl_3$) δ 5.43-5.26 (m, 2H), 4.63-4.43 (m, 0.2H), 4.25-4.15 (m, 0.8H), 3.67-3.52 (m, 0.2 H), 3.25-3.10 (m, 0.8H), 2.12-1.92 (m, 4H), 1.84-1.43 (m, 8H), 1.40-1.17 (m, 22H), 1.04-0.95 (m, 3H), 0.93-0.82 (m, 3H). ^{13}C NMR (50 MHz, $CDCl_3$) δ 172.3, 171.6, 130.2, 130.1,129.6, 129.5, 77.9, 75.5, 56.1, 52.6, 36.5, 32.2, 31.9, 29.7, 29.6, 29.5, 29.4, 29.3, 28.9, 27.8, 27.6, 27.2, 27.1, 27.0, 23.9, 22.7, 18.9, 18.4, 14.1, 13.8, 13.7. HRMS (ESI) $[M+Na]^+$: calcd for $C_{24}H_{44}NaO_2^+$ 387.3234, found 387.3228.

(±)-*trans* (Z)-3-(Octadec-9-en-1-yl)-4-propyloxetan-2-one (VM029)

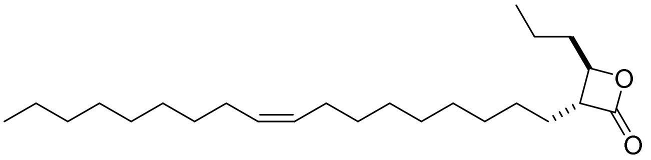

$C_{24}H_{44}O_2$
Exact Mass: 364,3341

Purified by 2nd column chromatography of **VM028** eluting with a gradient of hexane/EtOAc 98:2 to 95:5 (*v/v*). Yellowish oil; ^{1}H NMR (200 MHz, $CDCl_3$) δ 5.43-5.26 (m, 2H), 4.23 (ddd, J = 7 Hz, 6 Hz, 4 Hz, 1H), 3.17 (ddd, J = 9 Hz, 7 Hz, 4 Hz, 1H), 2.13-1.89 (m, 4H), 1.88-1.54 (m, 6H), 1.54-1.17 (m, 24H), 0.99 (t, J = 7 Hz, 3H), 0.93-0.79 (m, 3H). ^{13}C NMR (50 MHz, $CDCl_3$) δ 171.8, 130.2, 130.0, 78.1, 56.4, 36.7, 32.1, 30.0, 29.9, 29.7, 29.6, 29.5, 29.4, 28.1, 27.4, 27.2, 22.9, 18.7, 14.3, 14.0. HRMS (ESI) $[M+Na]^+$: calcd for $C_{24}H_{44}NaO_2^+$ 387.3234, found 387.3229.

(±)-*cis* (Z)-3-(Octadec-9-en-1-yl)-4-propyloxetan-2-one (VM030)

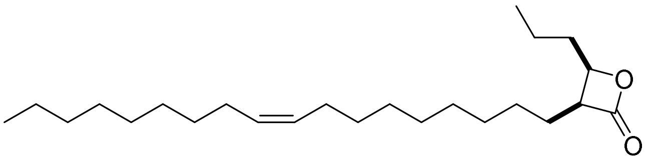

$C_{24}H_{44}O_2$
Exact Mass: 364,3341

Purified by 2nd column chromatography of **VM028** eluting with a gradient of hexane/EtOAc 98:2 to 95:5 (*v/v*). Yellowish oil; ^{1}H NMR (200 MHz, $CDCl_3$) δ 5.43-5.26 (m, 2H), 4.63-4.43 (m, 1H), 3.67-3.52 (m, 1H), 2.12-1.92 (m, 4H), 1.84-1.43 (m, 8H), 1.40-1.17 (m, 22H), 1.04-0.95 (m, 3H), 0.93-0.82 (m, 3H). ^{13}C NMR (50 MHz, $CDCl_3$) δ 172.6, 130.2, 130.0, 75.7, 52.8, 32.4, 32.1, 29.9, 29.7, 29.6, 29.5, 29.4, 27.8, 27.4, 24.1, 22.9, 19.1, 14.3, 14.0. HRMS (ESI) $[M+Na]^+$: calcd for $C_{24}H_{44}NaO_2^+$ 387.3234, found 387.3227.

(±)-*trans* 4-((Z)-Heptadec-8-en-1-yl)-3-propyloxetan-2-one (VM036)

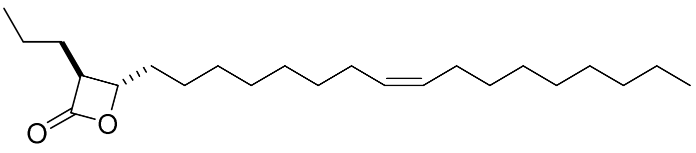

$C_{23}H_{42}O_2$
Exact Mass: 350,3185

Prepared according to **General procedure II** using **2g**; purified by column chromatography eluting with a gradient of hexane/EtOAc starting 97:3 to 95:5 (*v/v*) and then by 2nd column chromatography of the mixture **VM036/VM037** eluting with a gradient of hexane/EtOAc 98:2 to 95:5 (*v/v*). Colorless oil; ^{1}H NMR (400 MHz, $CDCl_3$) δ 5.43-5.26 (m, 2H), 4.21 (ddd, J = 7 Hz, 6 Hz, 4 Hz, 1H), 3.18 (ddd, J= 8 Hz, 6 Hz, 4 Hz, 1H), 2.08-1.95 (m, 4H), 1.95-1.55 (m, 4H), 1.55-1.21 (m, 24H), 0.96 (t, 3H, J = 7 Hz), 0.89 (t, 3H, J = 7 Hz). ^{13}C NMR (101 MHz, $CDCl_3$) δ 171.89, 130.28, 129.91, 78.39, 56.18, 34.67, 32.13, 30.18, 29.98, 29.90, 29.75, 29.54, 29.44, 29.33, 27.44, 27.37, 25.25, 22.91, 20.54, 14.35, 14.01. HRMS (ESI) $[M+Na]^+$: calcd for $C_{23}H_{42}NaO_2^+$ 373.3077, found 373.3079.

(±)-*cis* 4-((Z)-Heptadec-8-en-1-yl)-3-propyloxetan-2-one (VM037)

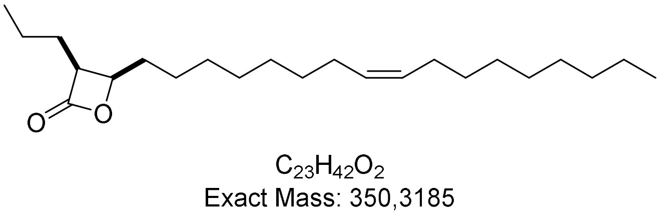

Purified by 2nd column chromatography of the mixture **VM036/VM037** eluting with a gradient of hexane/EtOAc 98:2 to 95:5 (*v/v*). Colorless oil; ^{1}H NMR (400 MHz, $CDCl_3$) δ 5.45-5.25 (m, 2H), 4.54 (ddd, J = 9 Hz, 6 Hz, 4 Hz, 1H), 3.55-3.68 (m, 1H), 2.08-1.95 (m, 4H), 1.76-1.40 (m, 8H), 1.41-1.21 (m, 20H), 0.96 (t, 3H, J = 7 Hz), 0.89 (t, 3H, J = 7 Hz). ^{13}C NMR (101 MHz, $CDCl_3$) δ 172.40, 130.06, 129.74, 75.73, 52.41, 31.93, 30.23, 29.78, 29.71, 29.55, 29.35, 29.32, 29.16, 27.24, 27.18, 25.97, 25.57, 22.71, 20.90, 14.16, 13.90. HRMS (ESI) $[M+Na]^+$: calcd for $C_{23}H_{42}NaO_2^+$ 373.3077, found 373.3078.

(±)-*trans* 3-Octyl-4-propyloxetan-2-one (VM038)

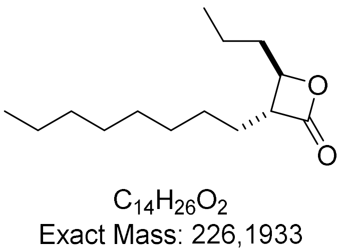

Prepared according to **General procedure II** using **2h**; purified by column chromatography eluting with a gradient of hexane/EtOAc starting 97:3 to 95:5 (*v/v*) and then by 2nd column chromatography of the mixture **VM038/VM039** eluting with a gradient of hexane/EtOAc 98:2 to 95:5 (*v/v*). Low m.p. white solid; ^{1}H NMR (400 MHz, $CDCl_3$) δ 4.23 (ddd, J = 7 Hz, 6 Hz, 4 Hz, 1H), 3.17 (ddd, J = 8.5 Hz, 6.5 Hz, 4 Hz, 1H), 1.90-1.62 (m, 4H), 1.50-1.21 (m, 14H), 0.98 (t, J = 7 Hz, 3H), 0.88 (t, J = 7 Hz, 3H). ^{13}C NMR (101 MHz, $CDCl_3$) δ 172.45, 78.16, 56.37, 36.70, 32.00, 29.47, 29.36, 28.08, 27.19, 22.83, 18.64, 14.28, 13.96. HRMS (ESI) $[M+Na]^+$: calcd for $C_{14}H_{26}NaO_2^+$ 249.1825, found 249.1824.

(±)-*cis* 3-Octyl-4-propyloxetan-2-one (VM039)

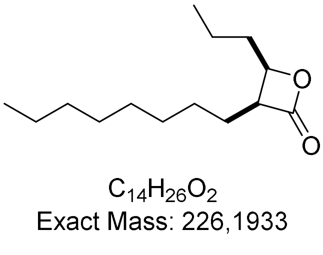

Prepared according to **General procedure II**; purified by column chromatography eluting with a gradient of hexane/EtOAc starting 97:3 to 95:5 (*v/v*) and then by 2nd column chromatography of the mixture **VM038/VM039** eluting with a gradient of hexane/EtOAc 98:2 to 95:5 (*v/v*). Yellowish oil; ^{1}H NMR (400 MHz, $CDCl_3$) δ 4.55 (ddd, J= 9 Hz, 6 Hz, 4 Hz, 1H), 3.53-3.67 (m, 1H), 1.81-1.30 (m, 8H), 1.30-1.21 (m, 10H), 0.99 (t, J = 7 Hz, 3H), 0.88 (t, J = 7 Hz, 3H). ^{13}C NMR (101 MHz, $CDCl_3$) δ 172.08, 75.71, 52.87, 32.42, 32.03, 29.61, 29.48,

29.40, 27.81, 24.14, 22.85, 19.11, 14.30, 14.02. HRMS (ESI) $[M+Na]^+$: calcd for $C_{14}H_{26}NaO_2^+$ 249.1825, found 249.1824.

(±)-*trans* 3-Dodecyl-4-propyloxetan-2-one (VM040)

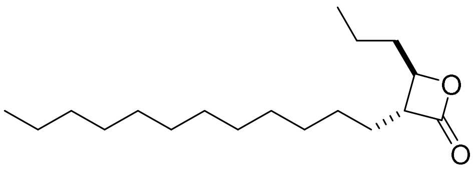

$C_{18}H_{34}O_2$
Exact Mass: 282,2559

Prepared according to **General procedure II** using **2i**; purified by column chromatography eluting with a gradient of hexane/EtOAc starting 97:3 to 95:5 (*v/v*) and then by 2nd column chromatography of the mixture **VM040/VM041** eluting with a gradient of hexane/EtOAc 98:2 to 95:5 (*v/v*). Low m.p. white solid; ^1H NMR (400 MHz, $CDCl_3$) δ 4.21 (ddd, J = 7.5 Hz, 6 Hz, 4 Hz, 1H), 3.15 (ddd, J =8.5 Hz, 6.5 Hz, 4 Hz, 1H), 1.90-1.61 (m, 4H), 1.53-1.11 (m, 22H), 0.97 (t, J = 7 Hz, 3H), 0.87 (t, J = 7 Hz, 3H). ^{13}C NMR (101 MHz, $CDCl_3$) δ 171.91, 78.19, 56.38, 36.72, 32.13, 29.85, 29.81, 29.72, 29.56, 29.53, 29.50, 28.09, 27.21, 22.90, 18.66, 14.33, 13.99. HRMS (ESI) $[M+Na]^+$: calcd for $C_{18}H_{34}NaO_2^+$ 305.2451, found 305.2446.

(±)-*cis* 3-Dodecyl-4-propyloxetan-2-one (VM041)

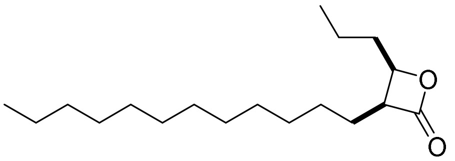

$C_{18}H_{34}O_2$
Exact Mass: 282,2559

Prepared according to **General procedure II**; purified by column chromatography eluting with a gradient of hexane/EtOAc starting 97:3 to 95:5 (*v/v*) and then by 2nd column chromatography of the mixture **VM040/VM041** eluting with a gradient of hexane/EtOAc 98:2 to 95:5 (*v/v*). Low m.p. white solid; ^1H NMR (400 MHz, $CDCl_3$) δ 4.55 (ddd, J = 10 Hz, 6.5 Hz, 4 Hz, 1H), 3.60 (ddd, J = 9.0 Hz, 7.5 Hz, 6.5 Hz, 1H), 1.81-1.59 (m, 4H), 1.51-1.22 (m, 22H), 1.00 (t, J = 7 Hz, 3H), 0.89 (t, J = 7 Hz, 3H). ^{13}C NMR (101 MHz, $CDCl_3$) δ 172.34, 75.49, 52.69, 32.22, 31.92, 29.65, 29.63, 29.53, 29.40, 29.34, 29.32, 27.62, 23.94, 22.69, 18.91, 14.11, 13.81. HRMS (ESI) $[M+Na]^+$: calcd for $C_{18}H_{34}NaO_2^+$ 305.2451, found 305.2448.

(*Z*)-3-Fluoro-3-(hexadec-7-en-1-yl)-4-propyloxetan-2-one (VM042)

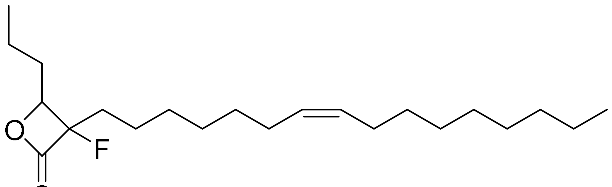

$C_{22}H_{39}FO_2$
Exact Mass: 354,2934

Prepared according to **General procedure IV** using **VM001**; purified by column chromatography eluting with a gradient of hexane/EtOAc starting 98:2 to 97:3 (*v/v*). Isolated mixture of diastereomers after column chromatography (dr 6:4). Yield 39%; ^{1}H NMR (400 MHz, $CDCl_3$) δ 5.44-5.32 (m, 2H), 4.69-4.60 (m, 0.4H), 4.49-4.41 (m, 0.6H), 2.11-2.00 (m, 4H), 2.00-1.40 (m, 8H), 1.40-1.25 (m, 18H), 1.03 (t, J = 7.4 Hz, 3H), 0.91 (t, J = 6.8 Hz, 3H). ^{13}C NMR (101 MHz, $CDCl_3$) δ 167.62 (d, J = 25.3 Hz), 166.89 (d, J = 24.2 Hz), 130.22, 130.17, 129.55, 129.49, 102.96 (d, J = 217.2 Hz), 102.02 (d, J = 224.2 Hz), 83.43 (d, J = 25.3 Hz), 82.27 (d, J = 22.2 Hz), 32.37 (d, J = 23.2 Hz), 31.91, 31.47 (d, J = 3.0 Hz), 30.95 (d, J = 4.0 Hz), 29.76, 29.53, 29.33, 28.88, 28.72 (d, J = 24.2 Hz), 27.24, 27.10, 27.07, 22.69, 22.54 (d, J = 4.0 Hz), 22.14 (d, J = 4.0 Hz), 18.76, 17.95, 14.11, 13.79, 13.67. ^{19}F NMR (376 MHz, CDCl3) δ -159.77, -173.46. HRMS (ESI) $[M+Na]^+$: calcd for $C_{22}H_{39}FNaO_2^+$ 377.2826, found 377.2820.

3-Fluoro-3-octyl-4-propyloxetan-2-one (VM043)

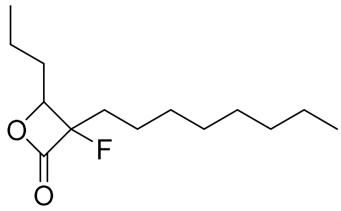

$C_{14}H_{25}FO_2$
Exact Mass: 244,1839

Prepared according to **General procedure III** using **11a**; purified by column chromatography eluting with hexane/EtOAc 95:5 (*v/v*). Isolated mixture of diastereomers after column chromatography (dr 65:35). Yield 36%; Yellowish oil; ^{1}H NMR (400 MHz, $CDCl_3$) δ 4.71-4.61 (m, 0.35H), 4.50-4.42 (m, 0.65H), 2.07-1.65 (m, 4H), 1.65-1.25 (m, 14H), 1.02 (t, J = 7.4 Hz, 3H), 0.90 (t, J = 6.6 Hz, 3H). ^{13}C NMR (101 MHz, $CDCl_3$) δ 167.66 (d, J = 25.3 Hz), 166.93 (d, J = 24.2 Hz), 102.90 (d, J = 217.2 Hz), 102.00 (d, J = 224.2 Hz), 83.44 (d, J = 25.3 Hz), 82.26 (d, J = 22.2 Hz), 32.36 (d, J = 23.2 Hz), 31.80, 31.77, 31.46 (d, J = 3.0 Hz), 30.95 (d, J = 5.1 Hz), 29.65, 29.39, 29.21, 29.11, 29.08, 28.72 (d, J = 23.2 Hz, *C*H_2CF), 22.63, 22.62, 22.58, 22.54, 22.16, 22.12, 18.76, 17.95, 14.07, 13.79, 13.66. ^{19}F NMR (376 MHz, CDCl3) δ -159.76, -173.43. HRMS (ESI) $[M+Na]^+$: calcd for $C_{14}H_{25}FNaO_2^+$ 267.1731, found 267.1732.

3-Dodecyl-3-fluoro-4-propyloxetan-2-one (VM044)

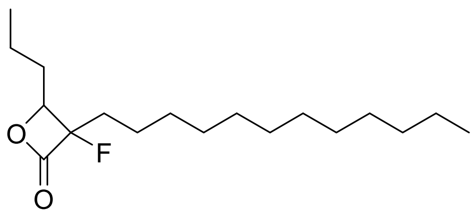

$C_{18}H_{33}FO_2$
Exact Mass: 300,2465

Prepared according to **General procedure III** using **11b**; purified by column chromatography eluting with hexane/EtOAc 96:4 (*v/v*). Isolated mixture of diastereomers after column chromatography (dr 1:1). Yield 29%; Yellowish oil; ^{1}H NMR (400 MHz, $CDCl_3$) 4.71-4.62 (m, 0.5H), 4.50-4.43 (m, 0.5H), 2.05-1.45 (m, 8H), 1.45-

1.25 (m, 18H), 1.02 (t, J = 7.4 Hz, 3H), 0.90 (t, J = 6.8 Hz, 3H). ^{13}C NMR (101 MHz, $CDCl_3$) δ 167.67 (d, J = 25.3 Hz), 166.93 (d, J = 24.2 Hz), 102.97 (d, J = 217.2 Hz), 102.05 (d, J = 224.2 Hz), 83.46 (d, J = 25.3 Hz), 82.28 (d, J = 22.2 Hz), 32.36 (d, J = 23.2 Hz), 31.92, 31.47 (d, J = 1.0 Hz), 30.95 (d, J = 4.0 Hz), 29.66, 29.63, 29.59, 29.57, 29.46, 29.42, 29.40, 29.34, 29.26, 28.73 (d, J = 23.2 Hz), 22.69, 22.57 (d, J = 4.0 Hz), 22.15 (d, J = 3.0 Hz), 18.76, 17.95, 14.12, 13.80, 13.68. ^{19}F NMR (376 MHz, CDCl3) δ -159.77, -173.46. HRMS (ESI) $[M+Na]^+$: calcd for $C_{18}H_{33}FNaO_2^+$ 323.2357, found 323.2359.

3-(8-(Octyloxy)octyl)-4-propyloxetan-2-one (VM045)

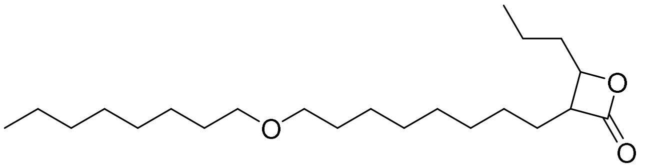

$C_{22}H_{42}O_3$
Exact Mass: 354,3134

Prepared according to **General procedure II** using **2j**; purified by column chromatography eluting with a gradient of hexane/EtOAc starting 97:3 to 95:5 (*v/v*). Isolated mixture of diastereomers after column chromatography (dr 3:7). Yield 60%; Colorless oil; ^{1}H NMR (200 MHz, $CDCl_3$) δ 4.59-4.50 (m, 0.7H), 4.26-4.20 (m, 0.3H), 3.65-3.55 (m, 0.7H), 3.32 (t, J = 7.0 Hz, 4H), 3.20-3.14 (m, 0.3H), 1.91-1.50 (m, 10H), 1.50-1.22 (m, 20H), 1.00 (t, J = 7 Hz, 3H), 0.86 (t, J = 7 Hz, 3H). ^{13}C NMR (50 MHz, $CDCl_3$) δ 172.28, 171.59, 77.92, 75.45, 70.98, 70.88, 56.17, 52.67, 36.50, 32.21, 31.83, 29.78, 29.75, 29.46, 29.36, 29.33, 29.27, 29.25, 29.22, 27.87, 27.58, 26.97, 26.20, 26.15, 23.92, 22.65, 18.89, 18.43, 14.08, 13.79, 13.75. HRMS (ESI) $[M+Na]^+$: calcd for $C_{22}H_{42}NaO_3^+$ 377.3026, found 377.3029.

3-Fluoro-3-(8-(octyloxy)octyl)-4-propyloxetan-2-one (VM046)

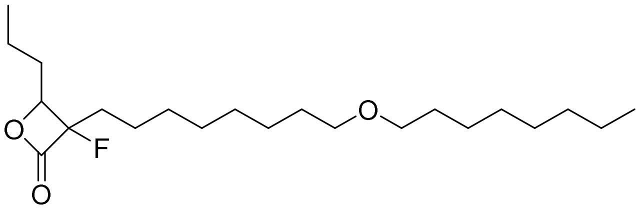

$C_{22}H_{41}FO_3$
Exact Mass: 372,3040

Prepared according to **General procedure IV** using **VM045**; purified by column chromatography eluting with hexane/Et_2O 95:5 (*v/v*). Isolated mixture of diastereomers after column chromatography (dr 55:45). Yield 23%; ^{1}H NMR (400 MHz, $CDCl_3$) δ 4.61-4.53 (m, 0.55H), 4.40-4.34 (m, 0.45H), 3.32 (t, J = 6.8 Hz, 4H), 1.98-1.14 (m, 30H), 0.93 (t, J = 7.3 Hz, 3H), 0.81 (t, J = 6.5 Hz, 3H). ^{13}C NMR (101 MHz, $CDCl_3$) δ 166.63 (d, J = 25.2 Hz), 165.91 (d, J = 23.90 Hz), 101.91 (d, J = 215.5 Hz), 101.00 (d, J = 224.3 Hz), 82.40 (d, J = 25.2 Hz), 81.24 (d, J = 21.4 Hz), 69.98, 69.86, 69.84, 31.31 (d, J = 2.5 Hz), 30.82, 30.43 (d, J = 2.5 Hz), 29.92 (d, J = 3.8 Hz), 28.75, 28.71, 28.56, 28.45, 28.30, 28.27, 28.24, 28.19, 28.18, 27.67 (d, J = 22.7), 25.18, 25.11, 21.65, 21.52 (d, J = 3.8 Hz), 21.10 (d, J = 3.8 Hz), 17.73, 16.92, 13.09, 12.78, 12.66. ^{19}F NMR (376 MHz, CDCl3) δ -159.71, -173.39. HRMS (ESI) $[M+Na]^+$: calcd for $C_{22}H_{41}FNaO_3^+$ 395.2932, found 395.2935.

N-Hexyl-8-(2-oxo-4-propyloxetan-3-yl)octanamide (VM047)

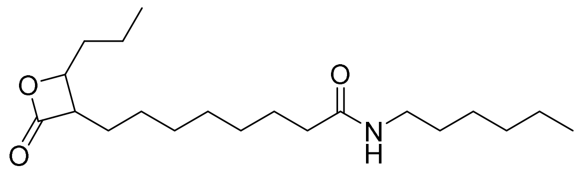

$C_{20}H_{37}NO_3$
Exact Mass: 339,2773

Prepared according to **General procedure III** using **7b**; purified by column chromatography eluting with hexane/EtOAc 1:1 (*v/v*). Isolated mixture of diastereomers after column chromatography (dr 9:1). Yield 39%; White solid; ^{1}H NMR (400 MHz, $CDCl_3$) 5.48 (s ,1H), 4.59-4.54 (m, 0.1H), 4.27-4.21 (m, 0.9H), 3.64-3.57 (m, 0.1H), 3.28-3.22 (m, 2H), 3.21-3.15 (m, 0.9H), 2.16 (t, J = 7.6 Hz, 2H), 1.91-1.25 (m, 24H), 1.00 (t, J = 7.4 Hz, 3H), 0.90 (t, J = 6.8 Hz, 3H). ^{13}C NMR (101 MHz, $CDCl_3$) δ 172.93, 172.37, 171.66, 77.94, 75.49, 56.13, 52.61, 39.52, 36.80, 36.49, 32.22, 31.48, 29.64, 29.16, 29.11, 29.09, 29.01, 27.83, 27.50, 26.89, 26.58, 25.69, 23.90, 22.56, 18.90, 18.44, 14.01, 13.81, 13.78. HRMS (ESI) [M+Na]$^+$: calcd for $C_{20}H_{37}NaNO_3^+$ 362.2665, found 362.2668.

N-(6-(2-Oxo-4-propyloxetan-3-yl)hexyl)octanamide (VM048)

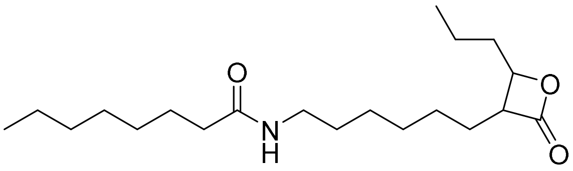

$C_{20}H_{37}NO_3$
Exact Mass: 339,2773

Prepared according to **General procedure III** using **7c**; purified by column chromatography eluting with hexane/EtOAc 1:1 (*v/v*). Isolated mixture of diastereomers after column chromatography (dr 75:25). Yield 45%; White solid; ^{1}H NMR (400 MHz, $CDCl_3$) δ 5.48 (s, 1H), 4.61-4.53 (m, 0.25H), 4.28-4.20 (m, 0.75H), 3.65-3.57 (m, 0.25H), 3.26 (q, J = 6.4 Hz, 2H), 3.22-3.15 (m, 0.75H), 2.18 (t, J = 7.6 Hz, 2H), 1.91-1.24 (m, 24H), 1.04-0.97 (m, 3H), 0.93-0.87 (m, 3H). ^{13}C NMR (101 MHz, $CDCl_3$) δ 173.19, 172.31, 171.59, 77.89, 75.45, 56.09, 52.58, 39.32, 36.89, 36.47, 32.23, 31.70, 29.58, 29.55, 29.27, 29.02, 28.95, 29.91, 27.77, 27.45, 26.84, 26.56, 25.84, 23.84, 22.60, 18.89, 18.44, 14.07, 13.80, 13.77. HRMS (ESI) [M+Na]$^+$: calcd for $C_{20}H_{37}NaNO_3^+$ 362.2665, found 362.2670.

3-Dodecyl-4-propylazetidin-2-one (VM056)

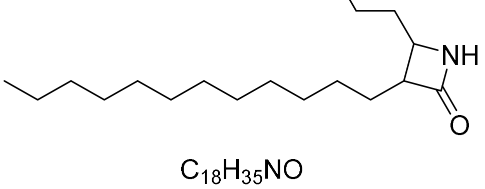

$C_{18}H_{35}NO$
Exact Mass: 281,2719

Prepared according to **General procedure VI** using **13a**; purified by column chromatography eluting with a gradient of hexane/EtOAc starting 7:3 to 1:1 (v/v). Isolated mixture of diastereomers after column chromatography (dr 6:4). Yield 71%; Colorless oil; ^{1}H NMR (400 MHz, $CDCl_3$) δ 6.06 (br s, 0.6H), 5.98 (br s, 0.4H), 3.72-3.63 (m, 0.6H), 3.34-3.26 (m, 0.4H), 3.22-3.12 (m, 0.6H), 2.78-2.70 (m, 0.4H), 1.85-1.20 (m, 26H), 1.05-0.95 (m, 3H), 0.90 (t, J = 6.6 Hz, 3H). ^{13}C NMR (101 MHz, $CDCl_3$) δ 172.16, 171.43, 56.99, 55.23, 53.14, 52.12, 37.33, 32.91, 31.92, 29.70, 29.68, 29.65, 29.61, 29.56, 29.42, 29.36, 28.60, 28.08, 27.37, 24.80, 22.70, 20.04, 19.77, 14.12, 14.03, 13.97. HRMS (ESI) $[M+Na]^+$: calcd for $C_{18}H_{35}NaNO^+$ 304.2613, found 304.2611.

3-Octyl-4-propylazetidin-2-one (VM058)

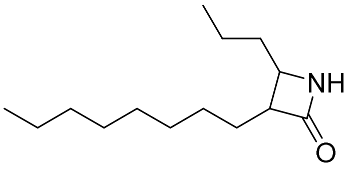

$C_{14}H_{27}NO$
Exact Mass: 225,2093

Prepared according to **General procedure VI** using **13b**; purified by column chromatography eluting with a gradient of hexane/EtOAc starting 8:2 to 6:4 (*v/v*). **VM058** gives rise to **VM057**. Isolated mixture of diastereomers after column chromatography (dr 6:4). Yield 65%; Colorless oil; ^{1}H NMR (400 MHz, $CDCl_3$) δ 6.01 (br s, 0.6H), 5.93 (br s, 0.4H), 3.71-3.64 (m, 0.6H), 3.34-3.27 (m, 0.4H), 3.22-3.13 (m, 0.6H), 2.79-2.70 (m, 0.4H), 1.87-1.22 (m, 18H), 0.98 (m, 3H), 0.90 (t, J = 6.7 Hz, 3H). ^{13}C NMR (101 MHz, $CDCl_3$) δ 172.18, 171.45, 56.99, 55.22, 53.15, 52.12, 37.32, 32.92, 31.86, 29.69, 29.55, 29.37, 29.25, 29.23, 28.60, 28.07, 27.35, 24.80, 22.65, 20.02, 19.76, 14.09, 14.01, 13.96. HRMS (ESI) $[M+Na]^+$: calcd for $C_{14}H_{27}NaNO^+$ 248.1983, found 248.1985.

(±)-*trans* 3-Octyl-4-propylazetidin-2-one (VM057)

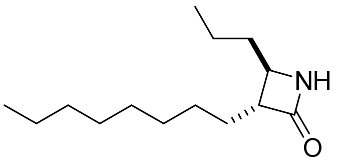

$C_{14}H_{27}NO$
Exact Mass: 225,2093

Purified by 2nd column chromatography of **VM058** eluting with a gradient of hexane/EtOAc 95:5 to 8:2 (*v/v*). Colorless oil; ^{1}H NMR (400 MHz, $CDCl_3$) δ 6.07 (br s, 1H), 3.34-3.26 (m, 1H), 2.77-2.69 (m, 1H), 1.78 (m, 1H), 1.69-1.56 (m, 3H), 1.48-1.21 (m, 14H), 0.97 (t, J = 7.3 Hz, 3H), 0.89 (t, J = 6.7 Hz, 3H). ^{13}C NMR (101 MHz, $CDCl_3$) δ 171.53, 56.98, 55.23, 37.31, 31.84, 29.55, 29.37, 29.23, 28.59, 27.35, 22.64, 19.76, 14.08, 13.96. HRMS (ESI) $[M+Na]^+$: calcd for $C_{14}H_{27}NaNO^+$ 248.1983, found 248.1985.

Synthesis of β-lactone probes

(*Z*)-4-(But-3-yn-1-yl)-3-(octadec-9-en-1-yl)oxetan-2-one (VM035$_p$)

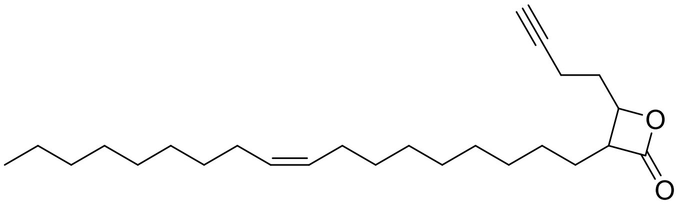

$C_{25}H_{42}O_2$
Exact Mass: 374,3185

Prepared according to **General procedure II** using **18a**; purified by column chromatography eluting with hexane/EtOAc 9:1 (*v/v*). Isolated mixture of diastereomers after column chromatography (dr 6:4). Yield 52%; Yellowish oil; ^{1}H NMR (200 MHz, $CDCl_3$) δ 5.42-5.24 (m, 2H), 4.71 (ddd, J = 9 Hz, 6 Hz, 5 Hz, 0.4H), 4.37 (ddd, J = 7, 6, 4 Hz, 0.6H), 3.71-3.57 (m, 0.4H), 3.26 (ddd, J = 8, 7, 4 Hz, 0.6H), 2.44-2.27 (m, 2H), 2.13-1.72 (m, 9H), 1.56-1.02 (m, 24H), 1.01-0.77 (m, 3H). ^{13}C NMR (50 MHz, $CDCl_3$) δ 172.8, 171.0, 129.9, 129.7, 82.3, 82.0, 76.3, 73.7, 69.8, 69.5, 56.1, 52.6, 39.1, 33.0, 31.9, 29.7, 29.7, 29.5, 29.4, 29.3, 29.3, 29.2, 29.2, 27.7, 27.5, 27.2, 27.1, 26.8, 23.9, 22.6, 14.8, 14.6, 14.1. HRMS (ESI) $[M+Na]^+$: calcd for $C_{25}H_{42}NaO_2^+$ 397.3077, found 397.3078.

3-(Pent-4-yn-1-yl)-4-propyloxetan-2-one (VM049$_p$)

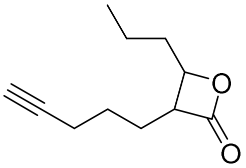

$C_{11}H_{16}O_2$
Exact Mass: 180,1150

Prepared according to **General procedure V** using **24**; purified by column chromatography eluting with hexane/EtOAc 8:2 (*v/v*). **VM049$_p$** gives rise to **VM050$_p$**. Isolated mixture of diastereomers after column chromatography (dr 6:4). Yield 96%; Colorless oil; ^{1}H NMR (400 MHz, $CDCl_3$) δ 4.59 (ddd, J = 10.1 Hz, 6.4 Hz, 3.9 Hz, 0.6H), 4.27 (ddd, J = 7.5 Hz, 5.9 Hz, 4.0 Hz, 0.4H), 3.70-3.62 (m, 0.6H), 3.22 (ddd, J = 8.4 Hz, 7.1 Hz, 3.9 Hz, 0.4H), 2.38-2.22 (m, 2H), 1.99 (t, J = 2.7 Hz, 1H), 1.97-1.38 (m, 8H), 1.01 (t, J = 7.4, 3H). ^{13}C NMR (101 MHz, $CDCl_3$) δ 171.87, 171.12, 83.30, 83.15, 77.83, 75.36, 69.26, 69.16, 55.67, 52.16, 36.44, 32.24, 26.87, 26.15, 25.66, 22.92, 18.89, 18.43, 18.17, 18.15, 13.78, 13.75. HRMS (ESI) $[M+Na]^+$: calcd for $C_{11}H_{16}NaO_2^+$ 203.1043, found 203.1042.

(±)-*trans* 3-(Pent-4-yn-1-yl)-4-propyloxetan-2-one (VM050$_p$)

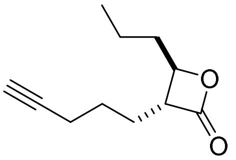

$C_{11}H_{16}O_2$
Exact Mass: 180,1150

Purified by 2nd column chromatography of **VM049$_p$** eluting with a gradient of hexane/EtOAc 95:5 to 8:2 (*v/v*). Colorless oil; ^{1}H NMR (400 MHz, $CDCl_3$) δ 4.30-4.25 (m, 1H), 3.25-3.20 (m, 1H), 2.28 (t, J = 6.8 Hz, 2H), 1.99

(s, 1H), 1.96-1.37 (m, 8H), 1.01 (t, *J* = 6.6 Hz, 3H). ^{13}C NMR (101 MHz, $CDCl_3$) δ 171.15, 83,15, 77.84, 69.27, 55.66, 36.44, 26.87, 25.66, 18.43, 18.14, 13.75. HRMS (ESI) $[M+Na]^+$: calcd for $C_{11}H_{16}NaO_2^+$ 203.1043, found 203.1042.

4-Propyl-3-(undec-10-yn-1-yl)oxetan-2-one (VM051$_p$)

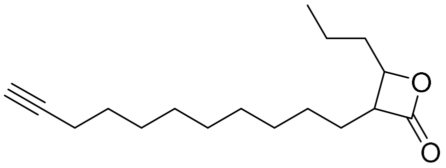

$C_{17}H_{28}O_2$
Exact Mass: 264,2089

Prepared according to **General procedure V** using **29**; purified by column chromatography eluting with a gradient of hexane/EtOAc starting 97:3 to 9:1 (*v/v*). Isolated mixture of diastereomers after column chromatography (dr 6:4). Yield 65%; Colorless oil; ^{1}H NMR (400 MHz, $CDCl_3$) δ 4.59-4.54 (m, 0.4H), 4.27-4.22 (m, 0.6H), 3.64-3.58 (m, 0.4H), 3.21-3.16 (m, 0.6H), 2.20 (t, *J* = 6.8 Hz, 2H), 1.96 (s, 1H), 1.91-1.27 (m, 20H), 1.01 (t, *J* = 7.4 Hz, 3H). ^{13}C NMR (101 MHz, $CDCl_3$) δ 172.34, 171.66, 84.75, 77.96, 75.49, 68.08, 56.18, 52.67, 36.51, 32.23, 29.37, 29.34, 29.27, 29.24, 29.03, 28.70, 28.46, 27.88, 27.60, 26.98, 23.94, 18.91, 18.64, 18.45, 18.39, 13.82, 13.78. HRMS (ESI) $[M+Na]^+$: calcd for $C_{17}H_{28}NaO_2^+$ 287.1982, found 287.1980.

(±)-*trans* 3-(Non-8-yn-1-yl)-4-propyloxetan-2-one (VM052$_p$)

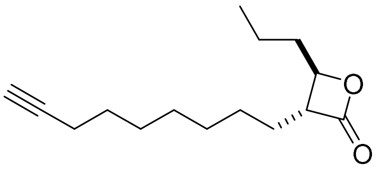

$C_{15}H_{24}O_2$
Exact Mass: 236,1776

Prepared according to **General procedure V** using **35a**; purified by column chromatography eluting with a gradient of hexane/EtOAc starting 97:3 to 92:8 (*v/v*). Only (±)-*trans* diastereomers were isolated after column chromatography. Yield 100%; Colorless oil; ^{1}H NMR (400 MHz, $CDCl_3$) δ 4.27-4.23 (m, 1H), 3.21-3.16 (m, 1H), 2.21 (t, *J* = 7 Hz, 2H), 1.96 (s, 1H), 1.91-1.80 (m, 2H), 1.75-1.69 (m, 2H), 1.59-1.33 (m, 12H), 1.01 (t, *J* = 7.4 Hz, 3H). ^{13}C NMR (101 MHz, $CDCl_3$) δ 171.62, 84.61, 77.95, 68.19, 56.15, 36.51, 29.15, 28.77, 28.55, 28.36, 27.86, 26.92, 18.45, 18.35, 13.78. HRMS (ESI) $[M+Na]^+$: calcd for $C_{15}H_{24}NaO_2^+$ 259.1658, found 259.1664.

(±)-*trans* 3-(Oct-7-yn-1-yl)-4-propyloxetan-2-one (VM053$_p$)

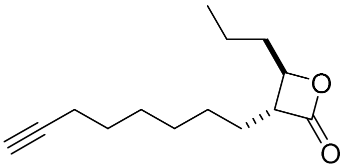

$C_{14}H_{22}O_2$
Exact Mass: 222,1620

Prepared according to **General procedure V** using **35b**; purified by column chromatography eluting with a gradient of hexane/EtOAc starting 97:3 to 92:8 (*v/v*). Only (±)-*trans* diastereomers were isolated after column chromatography. Yield 98%; Colorless oil; ^{1}H NMR (400 MHz, $CDCl_3$) δ 4.27-4.23 (m, 1H), 3.21-3.17 (m, 1H), 2.21 (t, J = 7 Hz, 2H), 1.96 (s, 1H), 1.91-1.80 (m, 2H), 1.79-1.69 (m, 2H), 1.57-1.42 (m, 10H), 1.01 (t, J = 7.4 Hz, 3H). ^{13}C NMR (101 MHz, $CDCl_3$) δ 171.57, 84.47, 77.93, 68.29, 56.14, 36.51, 28.77, 28.35, 28.26, 27.82, 26.86, 18.45, 18.33, 13.78. HRMS (ESI) $[M+Na]^+$: calcd for $C_{14}H_{22}NaO_2^+$ 245.1512, found 245.1510.

4-(But-3-yn-1-yl)-3-dodecyloxetan-2-one (VM054*p*)

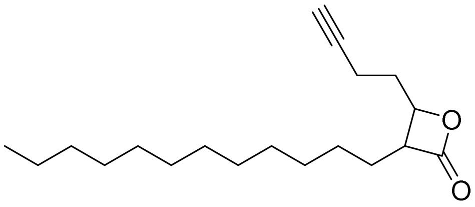

$C_{19}H_{32}O_2$
Exact Mass: 292,2402

Prepared according to **General procedure II** using **18b**; purified by column chromatography eluting with hexane/EtOAc 95:5 (*v/v*). Isolated mixture of diastereomers after column chromatography (dr 6:4). Yield 52%; Colorless oil; ^{1}H NMR (400 MHz, $CDCl_3$) δ 4.78-4.73 (m, 0.4H), 4.43-4.39 (m, 0.6H) 3.71-3.65 (m, 0.4H), 3.32-3.27 (m, 0.6H), 2.45-2.37 (m, 2H), 2.13-1.75 (m, 5H), 1.67-1.28 (m, 20H), 0.90 (t, J = 6.6 Hz, 3H). ^{13}C NMR (101 MHz, $CDCl_3$) δ 171.68, 171.04, 82.32, 82.11, 76.41, 73.80, 69.80, 69.58, 56.20, 52.72, 33.12, 31.92, 29.64, 29.60, 29.51, 29.37, 29.34, 29.31, 29.28, 27.77, 27.57, 26.90, 24.01, 22.69, 14.85, 14.65, 14.12. HRMS (ESI) $[M+Na]^+$: calcd for $C_{19}H_{32}NaO_2^+$ 315.2294, found 315.2304.

(±)-*trans* 3-(8-Azidooctyl)-4-propyloxetan-2-one (VM055*p*)

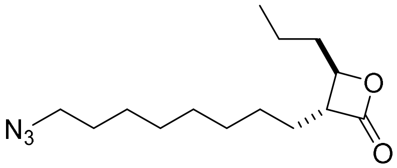

$C_{33}H_{57}N_3O_4$
Exact Mass: 559,4349

Prepared according to **General procedure I** using **39**, followed by **General procedure II**; purified by column chromatography eluting with hexane/EtOAc 95:5 (*v/v*). Only (±)-*trans* diastereomers were isolated after column chromatography. Yield 12%; Colorless oil; ^{1}H NMR (400 MHz, $CDCl_3$) δ 4.27-4.23 (m, 1H), 3.28 (t, J = 7 Hz, 2H), 3.22-3.17 (m, 1H), 1.92-1.80 (m, 2H), 1.78-1.69 (m, 2H), 1.52-1.34 (m, 14H), 1.01 (t, J = 7.4 Hz, 3H). ^{13}C NMR (101 MHz, $CDCl_3$) δ 171.58, 77.91, 56.16, 51.45, 36.50, 29.18, 29.15, 29.00, 28.80, 27.87, 26.93, 26.65, 18.44, 13.77. HRMS (ESI) $[M+Na]^+$: calcd for $C_{14}H_{25}NaN_3O_2^+$ 290.1839, found 290.1835.

Synthesis of intermediates

(*Z*)-2-(1-Hydroxyethyl)octadec-9-enoic acid (2a)

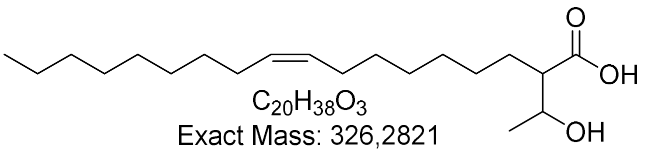

Prepared according to **General procedure I**; purified by column chromatography eluting with CH_2Cl_2/MeOH 95:5 (*v/v*). Mixture of diastereomers (dr 7:3). Yield 52%; Yellowish oil. ^{1}H NMR (200 MHz, $CDCl_3$): δ 5.43-5.23 (m, 2H), 4.12-3.99 (m, 0.3H), 3.99-3.85 (m, 0.7H), 2.52-2.25 (m, 1H), 2.11-1.85 (m, 4H), 1.77-1.55 (m, 2H), 1.55-1.03 (m, 23H), 0.87 (t, J = 7 Hz, 3H). ^{13}C NMR (50 MHz, $CDCl_3$) δ 179.9, 179.6, 129.9, 129.6, 68.5, 68.2, 52.9, 51.9, 34.1, 31.8, 29.7, 29.6, 29.5, 29.3, 29.2, 29.1, 29.0, 27.7, 27.1, 24.8, 22.6, 21.2, 19.8, 14.0. HRMS *m/z* [*M*-H]$^-$ calcd for $C_{20}H_{37}O_3^-$: 325.2743, found: 325.2740

(*Z*)-2-(1-Hydroxypropyl)octadec-9-enoic acid (2b)

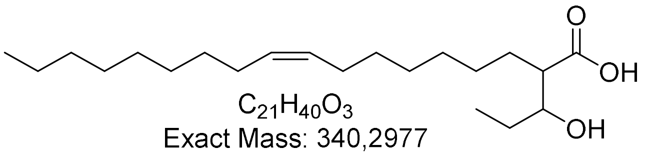

Prepared according to **General procedure I**; purified by column chromatography eluting with CH_2Cl_2/MeOH 95:5 (*v/v*). Mixture of diastereomers (dr 7:3). Yield 88%; Yellowish oil. ^{1}H NMR (200 MHz, $CDCl_3$): δ 5.41-5.20 (m, 2H), 3.80-3.68 (m, 0.3H), 3.68-3.53 (m, 0.7H), 2.51-2.33 (m, 1H), 2.11-1.85 (m, 4H), 1.77-1.07 (m, 24H), 0.95 (t, J = 7 Hz, 3H), 0.85 (t, J = 7 Hz, 3H). ^{13}C NMR (50 MHz, $CDCl_3$): δ 179.8, 129.8, 129.5, 73.6, 50.7, 31.8, 29.7, 29.6, 29.5, 29.4, 29.2, 29.1, 29.0, 27.8, 27.7, 27.2, 27.1, 26.8, 22.6, 14.0, 10.2, 9.8. HRMS *m/z* [*M*-H]$^-$ calcd for $C_{21}H_{39}O_3^-$: 339.2899, found: 339.2894

(*Z*)-2-(1-Hydroxyheptyl)octadec-9-enoic acid (2c)

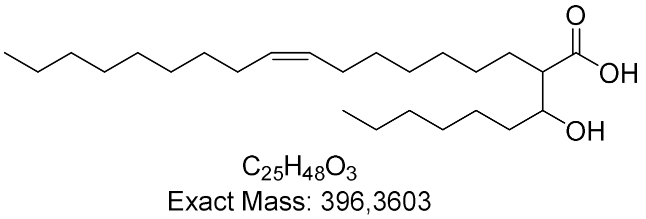

Prepared according to **General procedure I**; purified by column chromatography eluting with a gradient of CH_2Cl_2/MeOH 97:3 to 95:5 (*v/v*). Mixture of diastereomers (dr 6:4). Yield 84%; Yellowish oil. ^{1}H NMR (200 MHz, $CDCl_3$): δ 5.44-5.23 (m, 2H), 3.92-3.77 (m, 0.4H), 3.77-3.58 (m, 0.6H), 2.54-2.30 (m, 1H), 2.14-1.85 (m, 4H), 1.82-1.09 (m, 32H), 0.95-0.75 (m, 6H). ^{13}C NMR (50 MHz, $CDCl_3$): δ 180.1, 129.8, 129.5, 72.2, 72.1, 51.2, 50.9, 35.1, 33.9, 31.8, 31.7, 29.7, 29.5, 29.4, 29.2, 29.1, 29.0, 27.7, 27.3, 27.1, 26.7, 25.9, 25.6, 22.6, 22.5, 14.0, 13.9. HRMS *m/z* [*M*-H]$^-$ calcd for $C_{25}H_{47}O_3^-$: 395.3525, found: 395.3520.

(*Z*)-2-(1-Hydroxydecyl)octadec-9-enoic acid (2d)

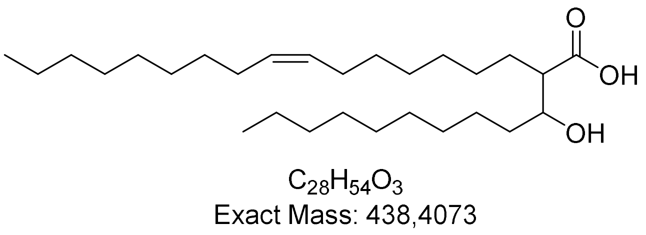

Prepared according to **General procedure I**; purified by column chromatography eluting with a gradient of CH_2Cl_2/MeOH 97:3 to 95:5 (*v/v*). Mixture of diastereomers (dr 7:3). Yield 83%; Yellowish oil. ^{1}H NMR (200 MHz, $CDCl_3$): δ 5.44-5.23 (m, 2H), 3.90-3.76 (m, 0.3H), 3.76-3.55 (m, 0.7H), 2.54-2.31 (m, 1H), 2.14-1.85 (m, 4H), 1.80-1.03 (m, 38H), 0.95-0.75 (m, 6H). ^{13}C NMR (50 MHz, $CDCl_3$): δ 180.4, 129.9, 129.6, 72.2, 72.1, 51.2, 50.8, 35.2, 33.8, 31.9, 29.7, 29.6, 29.5, 29.4, 29.3, 29.1, 27.8, 27.3, 27.2, 26.5, 26.0, 25.7, 22.6, 14.0. HRMS *m/z* [*M*-H]$^-$ calcd for $C_{28}H_{53}O_3^-$: 437.3995, found: 437.3994.

2-(1-Hydroxybutyl)octanoic acid (2e)

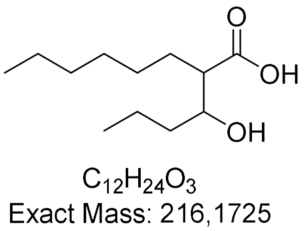

Prepared according to **General procedure I**; purified by column chromatography eluting with a gradient of CH_2Cl_2/MeOH 97:3 to 95:5 (*v/v*). Mixture of diastereomers (dr 7:3). Yield 33%; White solid. ^{1}H NMR (200 MHz, $CDCl_3$) δ 3.94-3.80 (m, 0.3H), 3.80-3.63 (m, 0.7H), 2.53-2.35 (m, 1H), 1.85-1.15 (m, 14H), 1.03-0.77 (m, 6H). ^{13}C NMR (50 MHz, $CDCl_3$) δ 180.7, 72.0, 71.8, 51.1, 50.9, 37.4, 36.0, 31.6, 29.4, 29.3, 29.2, 27.7, 27.3, 26.6, 22.5, 19.1, 18.8, 14.0, 13.9. HRMS *m/z* [*M*+Na]$^+$ calcd for $C_{12}H_{24}NaO_3$: 239.1618, found 239.1619.

2-(1-Hydroxybutyl)dodecanoic acid (2f)

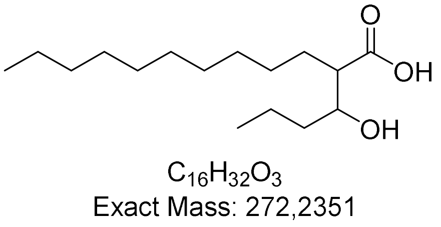

Prepared according to **General procedure I**; purified by column chromatography eluting with a gradient of CH_2Cl_2/MeOH 97:3 to 95:5 (*v/v*). Mixture of diastereomers (dr 7:3). Yield 61%; Yellowish solid. ^{1}H NMR (200 MHz, $CDCl_3$) δ 3.93-3.79 (m, 0.3H), 3.79-3.63 (m, 0.7H), 2.52-2.30 (m, 1H), 1.82-1.07 (m, 22H), 1.02-0.77 (m, 6H). ^{13}C NMR (50 MHz, $CDCl_3$) δ 180.4, 72.0, 71.9, 51.1, 50.9, 37.3, 36.0, 31.9, 29.6, 29.5, 29.4, 29.3, 27.8, 27.3, 26.7, 22.6, 19.1, 18.8, 14.0, 13.9. HRMS *m/z* [*M*+Na]$^+$ calcd for $C_{16}H_{32}NaO_3$: 295.2243, found 295.2228.

(*Z*)-3-Hydroxy-2-propylicos-11-enoic acid (2g)

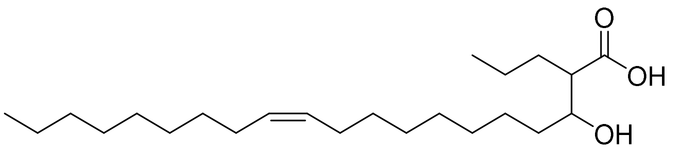

$C_{23}H_{44}O_3$
Exact Mass: 368,3290

Prepared according to **General procedure I**; purified by column chromatography eluting with CH_2Cl_2/MeOH 97:3 (*v/v*). Mixture of diastereomers (dr 7:3). Yield 52%; White solid. ^1H NMR (200 MHz, $CDCl_3$) δ 10.74 (s, 1H), 6.81 (s, 1H), 5.42-5.26 (m, 2H), 3.91-3.78 (m, 0.3H), 3.76-3.61 (m, 0.7H), 2.51-2.28 (m, 1H), 2.08-1.91 (m, 4H), 1.42-1.17 (m, 28H), 0.99-0.82 (m, 6H). ^{13}C NMR (50 MHz, $CDCl_3$) δ 181.02, 130.08, 129.91, 72.40, 72.19, 51.36, 50.72, 35.55, 32.05, 31.76, 29.91, 29.67, 29.47, 27.35, 25.87, 22.83, 20.70, 14.26, 14.12. HRMS *m/z* $[M+Na]^+$ calcd for $C_{23}H_{44}NaO_3$: 391.3182, found 391.3184.

2-(1-Hydroxybutyl)decanoic acid (2h)

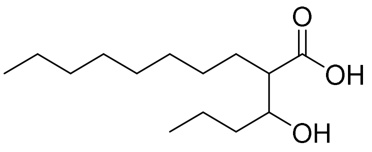

$C_{14}H_{28}O_3$
Exact Mass: 244,2038

Prepared according to **General procedure I**; purified by column chromatography eluting with a gradient of CH_2Cl_2/MeOH 97:3 to 9:1 (*v/v*). Mixture of diastereomers (dr 7:3). Yield 89%; Yellowish oil. ^1H NMR (200 MHz, $CDCl_3$) δ 10.75 (br s, 1H), 6.01 (s,1H), 3.93-3.80 (m, 0.3H), 3.78-3.65 (m, 0.7H), 2.52-2.30 (m, 1H), 1.82-1.07 (m, 18H), 1.02-0.77 (m, 6H). ^{13}C NMR (50 MHz, $CDCl_3$) δ 180.8, 180.1, 72.2, 72.0, 51.5, 51.2, 37.4, 36.3, 31.9, 29.7, 29.7, 29.6, 29.5, 29.4, 27.9, 27.5, 26.8, 22.8, 19.0, 18.9, 14.2, 14.2, 14.0. HRMS *m/z* $[M+Na]^+$ calcd for $C_{14}H_{28}NaO_3$: 267.1930, found 267.1929.

2-(1-Hydroxybutyl)tetradecanoic acid (2i)

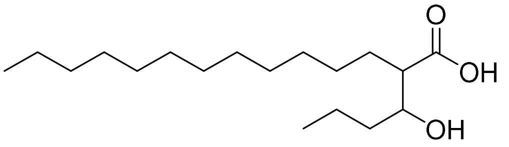

$C_{18}H_{36}O_3$
Exact Mass: 300,2664

Prepared according to **General procedure I**; purified by column chromatography eluting with a gradient of CH_2Cl_2/MeOH 97:3 to 9:1 (*v/v*). Mixture of diastereomers (dr 7:3). Yield 37%; White solid. ^1H NMR (200 MHz, $CDCl_3$) δ 10.75 (br s, 1H), 6.13 (s,1H), 3.96-3.76 (m, 0.3H), 3.77-3.63 (m, 0.7H), 2.49-2.36 (m, 1H), 1.70-1.16 (m, 26H), 1.02-0.80 (m, 6H). ^{13}C NMR (50 MHz, $CDCl_3$) δ 181.1, 179.8, 72.1, 72.0, 51.5, 51.1, 37.6, 36.2, 32.0, 29.8, 29.7, 29.6, 29.5, 27.6, 26.9, 22.8, 22.7, 19.2, 19.0, 14.3, 14.2, 14.1, 14.0. HRMS *m/z* $[M+Na]^+$ calcd for $C_{18}H_{36}NaO_3$: 323.2556, found 323.2553.

2-(1-Hydroxybutyl)-10-(octyloxy)decanoic acid (2j)

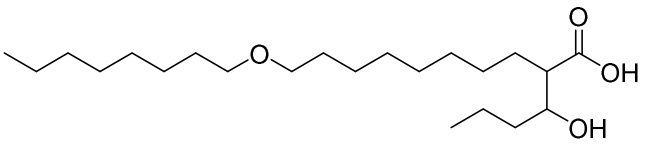

$C_{22}H_{44}O_4$
Exact Mass 372.3240

Prepared according to **General procedure I**; purified by column chromatography eluting with CH_2Cl_2/MeOH 97:3 (*v/v*). Mixture of diastereomers (dr 6:4). Yield 93%; Yellowish oil. ^{1}H NMR (400 MHz, $CDCl_3$) δ 3.90-3.84 (m, 0.6H), 3.77-3.70 (m, 0.4H), 3.42 (t, J = 6.8 Hz, 4H), 2.51-2.42 (m, 1H), 1.79-1.23 (m, 30H), 0.95 (t, J = 7 Hz, 3H), 0.89 (t, J = 6.6 Hz, 3H). ^{13}C NMR (101 MHz, $CDCl_3$) δ 179.92, 71.87, 71.80, 71.00, 70.94, 50.90, 50.80, 38.28, 36.15, 31.83, 29.69, 29.66, 29.50, 29.46, 29.40, 29.38, 29.36, 29.31, 29.27, 22.66, 19.16, 18.92, 14.10, 13.93. HRMS *m/z* [M+Na]$^+$ calcd for $C_{22}H_{44}NaO_4$: 395.3132, found 395.3145.

Ethyl 3-oxohexanoate (4) [3]

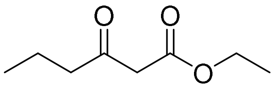

$C_8H_{14}O_3$
Exact Mass: 158.0943

Prepared according to **General procedure VII**; purified by column chromatography eluting with a gradient of hexane/EtOAc 95:5 to 9:1 (*v/v*). Yield 95%; Colorless oil. ^{1}H NMR (400 MHz, $CDCl_3$) δ 4.19 (q, J = 7.2 Hz, 2H), 3.43 (s, 2H), 2.52 (t, J = 7.4 Hz, 2H), 1.68-1.57 (m, 2H), 1.28 (t, J = 7.2 Hz, 3H), 0.93 (t, J = 7.4 Hz, 3H). ^{13}C NMR (101 MHz, $CDCl_3$) δ 202.24, 168.43, 61.31, 49.31, 44.87, 16.91, 14.08, 13.52.

Ethyl (*Z*)-2-butyrylicos-11-enoate (5a)

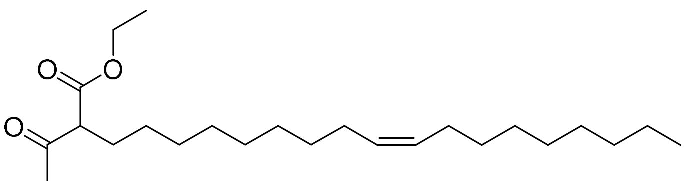

$C_{26}H_{48}O_3$
Exact Mass: 408,3603

Prepared according to **General procedure VIII** using oleyl iodide; purified by column chromatography eluting with hexane/EtOAc 95:5 (*v/v*). Yield 78%; Colorless oil. ^{1}H NMR (200 MHz, $CDCl_3$) δ 5.36- 5.31 (m, 2H), 4.18 (q, J = 7 Hz, 2H), 3.39 (t, J = 7 Hz, 1H), 2.60-2.34 (m, 2H), 2.06-1.91 (m, 4H), 1.88- 1.72 (m, 2H), 1.65 – 1.54 (m, 2H), 1.37-1.20 (m, 27H), 0.95 – 0.81 (m, 6H). ^{13}C NMR (50 MHz, $CDCl_3$) δ 205.4, 169.9, 129.9, 129.7, 61.1, 59.1, 43.61, 31.9, 29.7, 29.5, 29.4, 29.3, 29.3, 29.2, 28.1, 27.4, 27.1, 22.63 16.9, 14.1, 13.5. HRMS *m/z* [M+Na]$^+$ calcd for $C_{26}H_{48}NaO_3$: 431.3496, found 431.3492.

Ethyl 2-butyryl-10-(hexylamino)-10-oxodecanoate (5b)

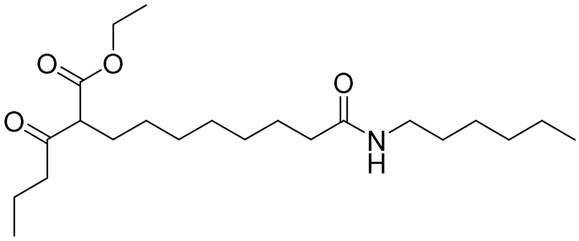

$C_{22}H_{41}NO_4$
Exact Mass: 383,3036

Prepared according to **General procedure VIII** using *N*-hexyl-8-iodooctanamide; purified by column chromatography eluting with a gradient of CH_2Cl_2/MeOH 98:2 to 95:5 (*v/v*). Yield 50%; Colorless oil. ^{1}H NMR (400 MHz, $CDCl_3$) δ 5.50 (s, 1H), 4.19 (q, J = 7.2 Hz, 2H), 3.42 (t, J = 7.4 Hz, 1H), 3.24 (q, J = 6.8 Hz, 2H), 2.60-2.41 (m, 2H), 2.16 (t, J = 7.8 Hz, 2H), 1.90-1.77 (m, 2H), 1.68-1.57 (m, 4H), 1.55-1.46 (m, 2H), 1.39-1.21 (m, 17H), 0.96-0.86 (m, 6H). ^{13}C NMR (101 MHz, $CDCl_3$) δ 206.33, 172.99, 169.96, 61.20, 59.17, 43.72, 39.52, 36.82, 31.47, 29.63, 29.16, 29.13, 28.98, 28.11, 27.37, 26.58, 25.72, 22.55, 16.94, 14.11, 14.00, 13.57. HRMS *m/z* $[M+Na]^+$ calcd for $C_{22}H_{41}NNaO_4$: 406.2928, found 406.2940.

Ethyl 2-butyryl-8-octanamidooctanoate (5c)

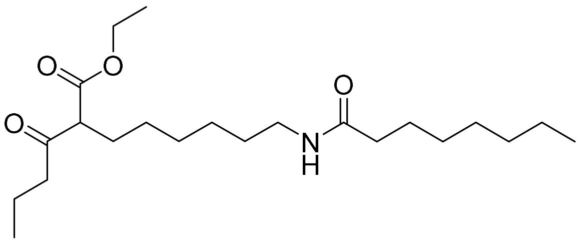

$C_{22}H_{41}NO_4$
Exact Mass: 383,3036

Prepared according to **General procedure VIII** using *N*-(6-bromohexyl)octanamide; purified by column chromatography eluting with a gradient of CH_2Cl_2/MeOH 98:2 to 95:5 (*v/v*). Yield 68%; Colorless oil. ^{1}H NMR (400 MHz, $CDCl_3$) δ 5.52 (s, 1H), 4.20 (q, J = 7.2 Hz, 2H), 3.42 (t, J = 7.4 Hz, 1H), 3.24 (q, J = 6.8 Hz, 2H), 2.61-2.41 (m, 2H), 2.17 (t, J = 7.8 Hz, 2H), 1.88-1.78 (m, 2H), 1.69-1.57 (m, 4H), 1.54-1.44 (m, 2H), 1.39-1.21 (m, 17H), 0.96-0.86 (m, 6H). ^{13}C NMR (101 MHz, $CDCl_3$) δ 205.39, 173.17, 169.92, 61.24, 59.10, 43.77, 39.38, 37.27, 31.69, 29.52, 29.27, 29.01, 28.97, 28.00, 27.30, 26.53, 25.84, 22.60, 16.93, 14.11, 14.06, 13.57. HRMS *m/z* $[M+Na]^+$ calcd for $C_{22}H_{41}NNaO_4$: 406.2928, found 406.2935.

Ethyl (*Z*)-2-(1-hydroxybutyl)icos-11-enoate (6a)

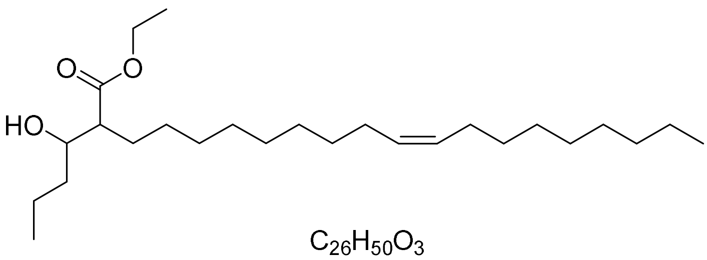

$C_{26}H_{50}O_3$
Exact Mass: 410,3760

Prepared according to **General procedure IX** using **5a**; purified by column chromatography eluting with hexane/EtOAc 9:1 (*v/v*). Mixture of diastereomers (dr 1:1). Yield 70%; Colorless oil. ^{1}H NMR (200 MHz, $CDCl_3$) δ 5.36-5.31 (m, 2H), 4.18 (q, J = 7 Hz, 2H), 3.85-3.75 (m, 0.5H), 3.75-3.60 (m, 0.5H), 2.58-2.32 (m, 2H), 2.10-1.90 (m, 4H), 1.79-1.20 (m, 33H), 1.00-0.80 (m, 6H). ^{13}C NMR (50 MHz, $CDCl_3$) δ 175.8, 175.7, 129.9, 129.8, 72.0, 71.8, 60.4, 51.0, 50.8, 37.9, 36.4, 31.9, 29.7, 29.6, 29.5, 29.50, 29.4, 29.3, 29.2, 27.7, 27.3, 27.2, 26.9, 22.7, 19.1, 18.9, 14.3, 14.1, 14.0.

Ethyl 10-(hexylamino)-2-(1-hydroxybutyl)-10-oxodecanoate (6b)

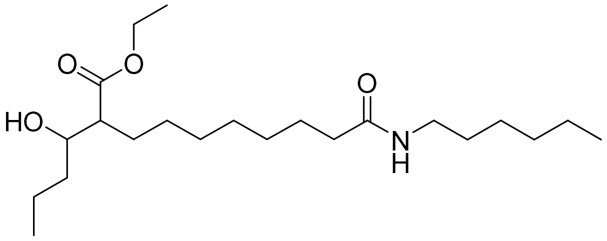

$C_{22}H_{43}NO_4$
Exact Mass: 385,3192

Prepared according to **General procedure IX** using **5b**; purified by column chromatography eluting with a gradient of CH_2Cl_2/MeOH 98:2 to 96:4 (*v/v*). Mixture of diastereomers (dr 1:1). Yield 86%; Colorless oil. ^{1}H NMR (400 MHz, $CDCl_3$) δ 5.40 (br s, 1H), 4.15-4.05 (m, 2H), 3.75-3.68 (m, 0.5H), 3.63-3.55 (m, 0.5H), 3.20-3.13 (m, 2H), 2.37-2.30 (m, 1H), 2.12-2.05 (m, 2H), 1.67-1.15 (m, 27H), 0.89-0.78 (m, 6H). ^{13}C NMR (101 MHz, $CDCl_3$) δ 175.85, 175.69, 173.08, 72.04, 71.84, 60.49, 60.46, 51.05, 50.83, 39.53, 37.89, 36.86, 36.46, 31.49, 29.64, 29.59, 29.38, 29.33, 29.20, 29.11, 27.64, 27.31, 26.89, 26.60, 25.78, 22.58, 19.14, 19.00, 14.34, 14.31, 14.04, 14.01. HRMS *m/z* $[M+Na]^+$ calcd for $C_{22}H_{43}NNaO_4$: 408.3084, found 408.3076.

Ethyl 2-(1-hydroxybutyl)-8-octanamidooctanoate (6c)

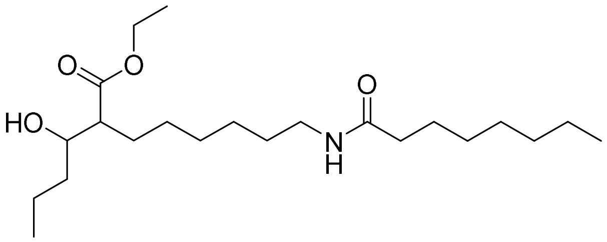

$C_{22}H_{43}NO_4$
Exact Mass: 385,3192

Prepared according to **General procedure IX** using **5c**; purified by column chromatography eluting with a gradient of CH_2Cl_2/MeOH 98:2 to 95:5 (*v/v*). Mixture of diastereomers (dr 7:3). Yield 75%; Colorless oil. ^{1}H NMR (400 MHz, $CDCl_3$) δ 5.54 (br s, 1H), 4.23-4.16 (m, 2H), 3.83-3.77 (m, 0.7H), 3.70-3.65 (m, 0.3H), 3.24 (q, J = 6.8 Hz, 2H), 2.45-2.39 (m, 1H), 2.20-2.14 (m, 2H), 1.78-1.23 (m, 27H), 0.94 (t, J = 7 Hz, 3H), 0.89 (t, J = 6.8 Hz, 3H). ^{13}C NMR (101 MHz, $CDCl_3$) δ 175.71, 175.54, 173.18, 72.03, 71.85, 60.47, 60.44, 51.09, 50.84, 39.43, 37.88, 36.88, 36.52, 31.69, 29.59, 29.48, 29.27, 29.17, 29.13, 29.00, 27.57, 27.24, 26.86, 26.64, 25.84, 22.59, 19.11, 18.98, 14.31, 14.28, 14.04, 13.96. HRMS *m/z* $[M+Na]^+$ calcd for $C_{22}H_{43}NNaO_4$: 408.3084, found 408.3074.

(*Z*)-2-(1-Hydroxybutyl)icos-11-enoic acid (7a)

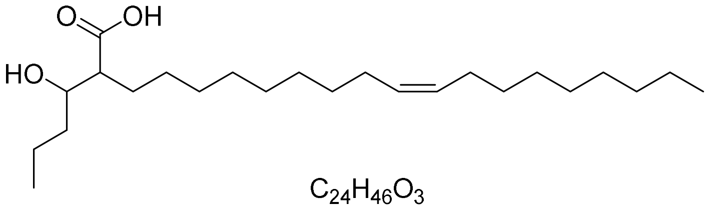

$C_{24}H_{46}O_3$
Exact Mass: 382,3447

Prepared according to **General procedure X** using **6a**; purified by column chromatography eluting with CH_2Cl_2 9:1 (*v/v*). Mixture of diastereomers (dr 1:1). Yield 54%; Colorless oil. ^{1}H NMR (200 MHz, $CDCl_3$) δ 5.45-5.32 (m, 2H), 4.00-3.80 (m, 0.5H), 3.80-3.60 (m, 0.5H), 2.54-2.36 (m, 1H), 2.14-1.87 (m, 4H), 1.83-1.25 (m, 30H), 1.03-0.77 (m, 6H). ^{13}C NMR (50 MHz, $CDCl_3$) δ 180.6, 129.2, 129.8, 71.9, 71.8, 51.0, 50.8, 37.6, 36.0, 32.6, 31.9, 29.8, 29.6, 29.5, 29.3, 27.8, 27.3, 27.2, 22.7, 19.2, 18.9, 14.1, 13.9. HRMS *m/z* $[M+Na]^+$ calcd for $C_{24}H_{46}NaO_3$: 405.3339, found 405.3334.

10-(Hexylamino)-2-(1-hydroxybutyl)-10-oxodecanoic acid (7b)

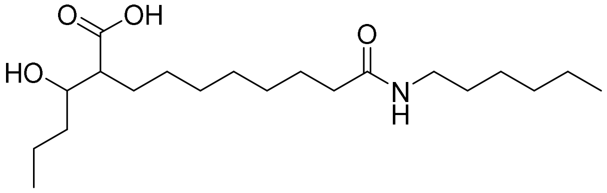

$C_{20}H_{39}NO_4$
Exact Mass: 357,2879

Prepared according to **General procedure IX** using **6b**; purified by column chromatography eluting with a gradient of CH_2Cl_2/MeOH 97:3 to 95:5 (*v/v*). Mixture of diastereomers (dr 1:1). Yield 85%; Colorless oil. ^{1}H NMR (400 MHz, $CDCl_3$) δ 5.81 (s, 1H), 3.90-3.84 (m, 0.5H), 3.76-3.68 (m, 0.5H), 3.25 (q, J = 6.8 Hz, 2H), 2.51-2.42 (m, 1H), 2.20 (t, J = 7.8 Hz, 2H), 1.80-1.27 (m, 24H), 0.95 (t, J = 7 Hz, 3H), 0.90 (t, J = 6.8 Hz, 3H). ^{13}C NMR (101 MHz, $CDCl_3$) δ 178.43, 178.37, 173.96, 71.86, 71.73, 50.71, 50.61, 39.74, 36.76, 36.70, 36.03, 31.47, 29.56, 29.49, 29.08, 28.96, 28.89, 28.83, 28.73, 27.53, 27.10, 26.58, 26.53, 25.69, 25.66, 22.55, 19.17, 19.00, 14.01, 13.96. HRMS *m/z* $[M+Na]^+$ calcd for $C_{20}H_{39}NNaO_4$: 380.2771, found 380.2782.

2-(1-Hydroxybutyl)-8-octanamidooctanoic acid (7c)

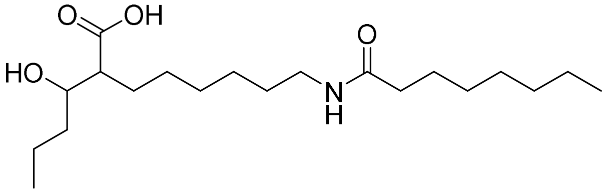

$C_{20}H_{39}NO_4$
Exact Mass: 357,2879

Prepared according to **General procedure IX** using **6c**; purified by column chromatography eluting with a gradient of CH_2Cl_2/MeOH 97:3 to 95:5 (*v/v*). Mixture of diastereomers (dr 45:55). Yield 56%; Colorless oil. ^{1}H NMR (400 MHz, $CDCl_3$) δ 5.94 (s, 1H), 3.90-3.83 (m, 0.45H), 3.76-3.68 (m, 0.55H), 3.24 (q, J = 6.8 Hz, 2H),

2.53-2.42 (m, 1H), 2.20 (t, J = 7.8 Hz, 2H), 1.81-1.22 (m, 24H), 0.95 (t, J = 7 Hz, 3H), 0.89 (t, J = 6.8 Hz, 3H). ^{13}C NMR (101 MHz, $CDCl_3$) δ 178.56, 178.45, 174.07, 71.82, 71.73, 50.69, 39.54, 37.72, 36.76, 36.05, 31.68, 29.42, 29.34, 29.24, 28.98, 28.86, 27.46, 27.05, 26.49, 26.44, 25.86, 22.59, 19.16, 18.98, 14.04, 13.94. HRMS *m/z* $[M{+}Na]^+$ calcd for $C_{20}H_{39}NNaO_4$: 380.2771, found 380.2780.

Ethyl 2-butyryldecanoate (8a)

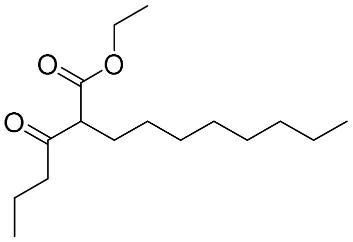

$C_{16}H_{30}O_3$
Exact Mass: 270,2195

Prepared according to **General procedure VIII**; purified by column chromatography eluting with a gradient of hexane/EtOAc 98:2 to 97:3 (*v/v*). Yield 70%; Colorless oil. ^{1}H NMR (400 MHz, $CDCl_3$) δ 4.20 (q, J = 7.2 Hz, 2H), 3.43 (t, J = 7.4 Hz, 1H), 2.60-2.42 (m, 2H), 1.91-1.77 (m, 2H), 1.67-1.59 (m, 2H), 1.36-1.22 (m, 15H), 0.96-0.86 (m, 6H). ^{13}C NMR (101 MHz, $CDCl_3$) δ 204.82, 170.38, 61.19, 59.26, 43.68, 31.82, 31.08, 29.36, 29.29, 29.18, 28.22, 27.47, 22.65, 16.49, 14.13, 13.59. HRMS *m/z* $[M{+}H]^+$ calcd for $C_{16}H_{31}O_3$: 271.2268, found 271.2274.

Ethyl 2-butyryltetradecanoate (8b)

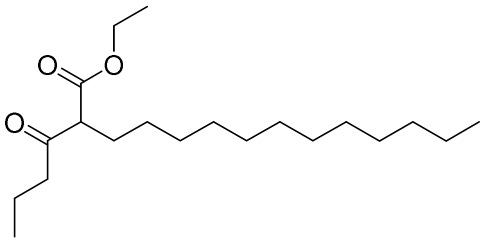

$C_{20}H_{38}O_3$
Exact Mass: 326,2821

Prepared according to **General procedure VIII**; purified by column chromatography eluting with a gradient of hexane/EtOAc 97:3 to 95:5 (*v/v*). Yield 67%; Colorless oil. ^{1}H NMR (400 MHz, $CDCl_3$) δ 4.20 (q, J = 7.2 Hz, 2H), 3.42 (t, J = 7.6 Hz, 1H), 2.61-2.41 (m, 2H), 1.90-1.76 (m, 2H), 1.69-1.57 (m, 2H), 1.36-1.21 (m, 23H), 0.96-0.86 (m, 6H). ^{13}C NMR (101 MHz, $CDCl_3$) δ 205.50, 170.37, 61.68, 59.25, 43.68, 31.92, 29.65, 29.63, 29.60, 29.53, 29.36, 29.35, 29.33, 28.21, 27.47, 22.69, 16.94, 14.12, 13.59. HRMS *m/z* $[M{+}H]^+$ calcd for $C_{20}H_{39}O_3$: 327.2894, found 327.2897.

Ethyl 2-butyryl-2-fluorodecanoate (9a)

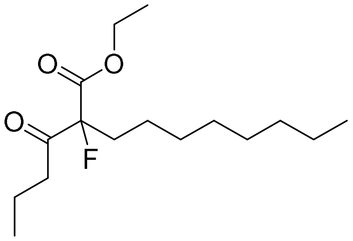

$C_{16}H_{29}FO_3$
Exact Mass: 288,2101

Prepared according to **General procedure XI** starting from **8a**; purified by column chromatography eluting with hexane/CH_2Cl_2 1:1 (*v/v*). Yield 82%; Colorless oil. ^{1}H NMR (400 MHz, $CDCl_3$) δ 4.27 (q, J = 6.9 Hz, 2H), 2.67-2.61 (m, 2H), 2.22-1.97 (m, 2H), 1.69-1.59 (m, 2H), 1.39-1.24 (m, 15H), 0.97-0.82 (m, 6H). ^{19}F NMR (376 MHz, $CDCl_3$) δ -169.49. ^{13}C NMR (101 MHz, $CDCl_3$) δ 204.32 (d, J = 28.3 Hz), 166.53 (d, J = 26.3), 100.7 (d, J = 198.0 Hz), 62.41, 39.81, 34.03 (d, J = 21.2 Hz), 31.78, 29.36, 29.19, 29.09, 22.68 (d, J = 3.0 Hz), 22.62, 16.35 (d, J = 2.0 Hz), 14.06 (d, J = 3.0 Hz), 13.49. HRMS *m/z* [*M*+H]$^+$ calcd for $C_{16}H_{30}FO_3$: 289.2184, found 289.2174.

Ethyl 2-butyryl-2-fluorotetradecanoate (9b)

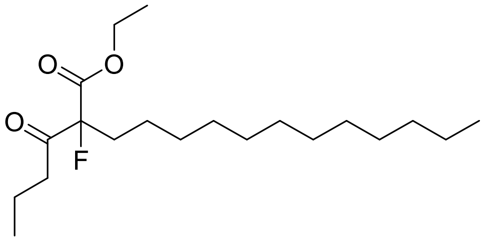

$C_{20}H_{37}FO_3$
Exact Mass: 344,2727

Prepared according to **General procedure XI** starting from **8b**; purified by column chromatography eluting with a gradient of hexane/CH_2Cl_2 8:2 to 6:4 (*v/v*). Yield 78%; Yellowish oil. ^{1}H NMR (400 MHz, $CDCl_3$) δ 4.32-4.23 (m, 2H), 2.67-2.60 (m, 2H), 2.21-1.97 (m, 2H), 1.68-1.58 (m, 2H), 1.38-1.21 (m, 23H), 0.97-0.86 (m, 6H). ^{19}F NMR (376 MHz, $CDCl_3$) δ -169.44. ^{13}C NMR (101 MHz, $CDCl_3$) δ 204.31 (d, J = 27.3 Hz), 166.52 (d, J = 26.3 Hz), 100.70 (d, J = 197.0 Hz), 62.40, 39.80, 34.03 (d, J = 21.2 Hz), 31.91, 29.62, 29.57, 29.43, 29.36, 29.34, 29.23, 22.68, 16.35 (d, J = 2.0 Hz), 14.07 (d, J = 7.1 Hz), 13.49. HRMS *m/z* [*M*+H]$^+$ calcd for $C_{20}H_{38}FO_3$: 345.2800, found 345.2802.

Ethyl 2-fluoro-2-(1-hydroxybutyl)decanoate (10a)

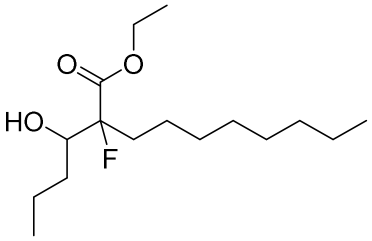

$C_{16}H_{31}FO_3$
Exact Mass: 290,2257

Prepared according to **General procedure IX** starting from **9a**; purified by column chromatography eluting with a gradient of hexane/EtOAc 95:5 to 9:1 (*v/v*). Mixture of diastereomers. Yield 82%; Colorless oil. ^{1}H NMR (400 MHz, $CDCl_3$) δ 4.36-4.25 (m, 2H), 3.86-3.73 (m, 1H), 2.05-1.13 (m, 21H), 1.00-0.92 (m, 3H), 0.90 (t, J = 6.8 Hz, 3H). ^{19}F NMR (376 MHz, $CDCl_3$) δ -176.47, -177.79. ^{13}C NMR (101 MHz, $CDCl_3$) δ 171.03 (d, J = 26.3 Hz), 170.38 (d, J = 25.3 Hz), 99.91 (d, J = 189.9 Hz), 99.56 (d, J = 191.9 Hz), 74.38 (d, J = 22.2 Hz), 74.32 (d, J = 25.3 Hz), 61.64, 61.57, 33.70 (d, J = 22.2 Hz), 33.59 (d, J = 22.2 Hz), 33.39 (d, J = 4.0 Hz), 33.03 (d, J = 3.0 Hz), 31.81, 29.53, 29.27, 29.13, 23.05, 23.02, 22.99, 22.64, 19.17, 18.94, 14.25, 14.23, 14.09, 13.89, 13.79. HRMS *m/z* $[M+H]^+$ calcd for $C_{16}H_{32}FO_3$: 291.2330, found 291.2338.

Ethyl 2-fluoro-2-(1-hydroxybutyl)tetradecanoate (10b)

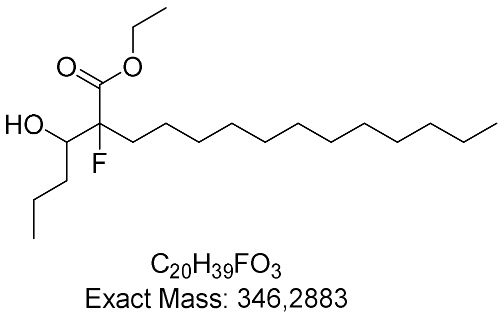

Prepared according to **General procedure IX** starting from **9b**; purified by column chromatography eluting with hexane/EtOAc 9:1 (*v/v*). Mixture of diastereomers. Yield 61%; Colorless oil. ^{1}H NMR (400 MHz, $CDCl_3$) δ 4.36-4.25 (m, 2H), 3.87-3.73 (m, 1H), 2.04-1.20 (m, 29H), 1.00-0.93 (m, 3H), 0.93-0.87 (m, 3H). ^{19}F NMR (376 MHz, $CDCl_3$) δ -176.42, -177.76. ^{13}C NMR (101 MHz, $CDCl_3$) δ 171.03 (d, J = 25.3 Hz), 170.38 (d, J = 26.3 Hz), 99.87 (d, J = 189.9 Hz), 99.56 (d, J = 191.9 Hz), 74.39 (d, J = 23.2 Hz), 74.31 (d, J = 24.2 Hz), 61.65, 61.57, 33.70 (d, J = 22.2 Hz), 33.59 (d, J = 22.2 Hz), 33.39 (d, J = 4.0 Hz), 33.04 (d, J = 2.0 Hz), 31.92, 29.65, 29.63, 29.60, 29.54, 29.48, 29.35, 29.32, 22.99, 22.69, 19.17, 18.94, 14.23, 14.12, 13.89, 13.80. HRMS *m/z* $[M+H]^+$ calcd for $C_{20}H_{40}FO_3$: 347.2956, found 347.2957.

2-Fluoro-2-(1-hydroxybutyl)decanoic acid (11a)

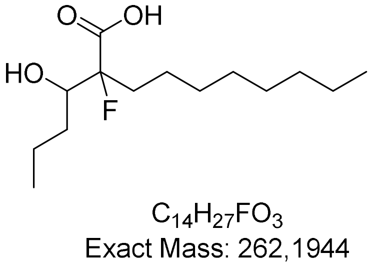

Prepared according to **General procedure X** starting from **10a**; purified by column chromatography eluting with a gradient of CH_2Cl_2/MeOH 98:2 to 8:2 (*v/v*). Mixture of diastereomers. Yield 75%; Colorless oil. ^{1}H NMR (400 MHz, $CDCl_3$) δ 5.95 (br s, 1H), 3.93-3.82 (m, 1H), 2.03-1.21 (m, 18H), 0.97 (t, J = 6.2Hz, 3H), 0.89 (t, J = 6.6 Hz, 3H). ^{19}F NMR (376 MHz, $CDCl_3$) δ -172.27, -178.75. ^{13}C NMR (101 MHz, $CDCl_3$) δ 174.43 (d, J = 27.3 Hz), 173.88 (d, J = 26.3 Hz), 100.17 (d, J = 188.9 Hz), 100.01 (d, J = 191.9 Hz), 74.64 (d, J = 23.2 Hz), 74.31 (d, J = 23.2 Hz), 33.45 (d, J = 22.2 Hz), 33.31 (d, J = 22.2 Hz), 32.96 (d, J = 4.0 Hz), 32.38 (d, J = 3.0

Hz), 31.81, 29.55, 29.26, 29.16, 23.10 (d, *J* = 3.0 Hz), 22.94 (d, *J* = 3.0 Hz), 22.63, 19.20, 18.83, 14.08, 13.81, 13.73. HRMS *m/z* $[M+H]^+$ calcd for $C_{14}H_{28}FO_3$: 263.2017, found 263.2024.

2-Fluoro-2-(1-hydroxybutyl)tetradecanoic acid (11b)

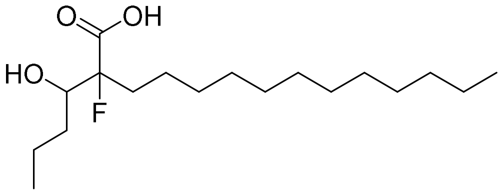

$C_{18}H_{35}FO_3$
Exact Mass: 318,2570

Prepared according to **General procedure X** starting from **10b**; purified by column chromatography eluting with a gradient of hexane/EtOAc 8:2 to CH_2Cl_2/MeOH 8:2 (*v/v*). Mixture of diastereomers. Yield 51%; Colorless oil. ^{1}H NMR (400 MHz, $CDCl_3$) δ 6.32 (br s, 1H), 3.92-3.81 (m, 1H), 2.03-1.19 (m, 26H), 1.00-0.93 (m, 3H), 0.89 (t, *J* = 6.6 Hz, 3H). ^{19}F NMR (376 MHz, $CDCl_3$) δ -171.58, -179.12. ^{13}C NMR (101 MHz, $CDCl_3$) δ 174.49 (d, *J* = 28.3 Hz), 173.88 (d, *J* = 27.3 Hz), 100.16 (d, *J* = 187.9 Hz), 100.10 (d, *J* = 191.9 Hz), 74.72 (d, *J* = 22.2 Hz), 74.32 (d, *J* = 23.2 Hz), 33.47 (d, *J* = 21.2 Hz), 33.31 (d, *J* = 21.2 Hz), 32.92, 32.26, 31.92, 29.67, 29.65, 29.63, 29.59, 29.55, 29.36, 23.14 (d, *J* = 3 Hz), 22.95 (d, *J* = 3 Hz), 22.69, 19.21, 28.82, 14.10, 13.82, 13.73. HRMS *m/z* $[M+Na]^+$ calcd for $C_{18}H_{35}FNaO_3$: 341.2462, found 341.2467.

2-(1-Azidobutyl)tetradecanoic acid (12a)

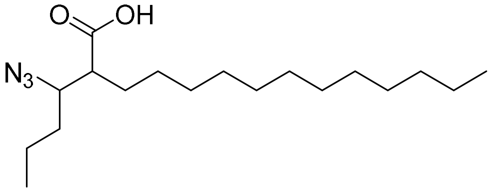

$C_{18}H_{35}N_3O_2$
Exact Mass: 325,2729

Prepared according to **General procedure XII** starting from **VM040**; purified by column chromatography eluting with hexane/EtOAc 95:5 (*v/v*). Mixture of diastereomers. Yield 77%; Colorless oil. ^{1}H NMR (400 MHz, $CDCl_3$) δ 3.57-3.52 (m, 1H), 2.53-2.46 (m, 1H), 1.75-1.19 (m, 26H), 0.99 (t, *J* = 4.8 Hz, 3H), 0.90 (t, *J* = 6.7 Hz, 3H). ^{13}C NMR (101 MHz, $CDCl_3$) δ 179.38,179.23, 63.68, 63.60, 50.19, 50.01, 34.88, 33.87, 31.93, 29.65, 29.58, 29.49, 29.40, 29.36, 28.87, 28.81, 27.52, 27.36, 27.21, 22.70, 19.52, 19.24, 14.12, 13.79, 13.71. HRMS *m/z* $[M+Na]^+$ calcd for $C_{18}H_{35}N_3NaO_2$: 348.2621, found 348.2632.

2-(1-Azidobutyl)decanoic acid (12b)

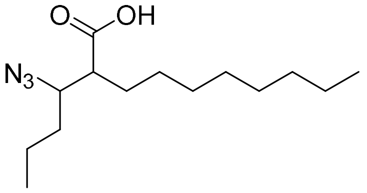

$C_{14}H_{27}N_3O_2$
Exact Mass: 269,2103

Prepared according to **General procedure XII** starting from **VM038**; purified by column chromatography eluting with hexane/EtOAc 9:1 (*v/v*). Mixture of diastereomers. Yield 65%; Colorless oil. ^{1}H NMR (400 MHz, $CDCl_3$) δ 3.57-3.52 (m, 1H), 2.52-2.46 (m, 1H), 1.75-1.29 (m, 18H), 0.98 (t, J = 6.7 Hz, 3H), 0.90 (t, J = 6.7 Hz, 3H). ^{13}C NMR (101 MHz, $CDCl_3$) δ 180.24, 63.65, 50.30, 34.88, 31.83, 29.48, 29.33, 29.21, 28.75, 27.35, 22.65, 19.51, 14.07, 13.69. HRMS *m/z* [*M*+Na]$^+$ calcd for $C_{14}H_{27}N_3NaO_2$: 292.1999, found 292.1995.

2-(1-Aminobutyl)tetradecanoic acid (13a)

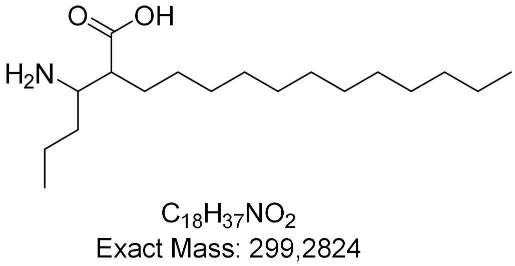

Prepared according to **General procedure XIII** starting from **12a**; purified by column chromatography eluting with a gradient of CH_2Cl_2/MeOH 8:2 to 7:3 (*v/v*). Mixture of diastereomers. Yield 44%; White solid. ^{1}H NMR (500 MHz, Methanol-d_4) δ 4.90 (s, 2H), 3.31-3.27 (m, 0.65H), 3.24-3.21 (m, 0.35H), 2.44-2.38 (m, 1H), 1.74-1.31 (m, 26H), 0.99 (m, 3H), 0.92 (t, J = 6.8 Hz, 3H). ^{13}C NMR (126 MHz, Methanol-d_4) δ 179.39, 52.80, 52.58, 34.13, 32.35, 31.68, 30.18, 29.40, 29.26, 29.09, 27.63, 27.28, 26.44, 22.34, 18.56, 18.51, 13.05, 12.71. HRMS *m/z* [*M*+H]$^+$ calcd for $C_{18}H_{38}NO_2$: 300.2909, found 300.2897.

2-(1-Aminobutyl)decanoic acid (13b)

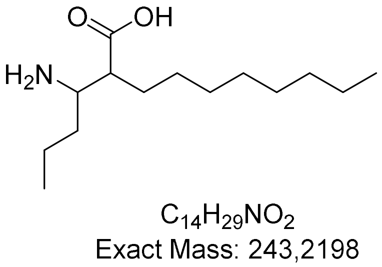

Prepared according to **General procedure XIII** starting from **12b**; purified by column chromatography eluting with a gradient of CH_2Cl_2/MeOH 8:2 to 6:4 (*v/v*). Mixture of diastereomers. Yield 36%; White solid. ^{1}H NMR (500 MHz, Methanol-d_4) δ 4.91 (s, 2H), 3.30-3.26 (m, 0.5H), 3.23-3.19 (m, 0.5H), 2.46-2.31 (m, 1H), 1.81-1.19 (m, 18H), 0.99 (m, 3H), 0.92 (t, J = 6.6 Hz, 3H). ^{13}C NMR (126 MHz, Methanol-d_4) δ 179.76, 179.57, 52.84, 52.62, 34.20, 32.36, 31.64, 30.28, 29.42, 29.35, 29.22, 29.00, 27.66, 27.29, 26.35, 22.31, 18.58, 18.54, 13.02, 12.71, 12.69. HRMS *m/z* [*M*+H]$^+$ calcd for $C_{14}H_{30}NO_2$: 244.2273, found 244.2271.

Ethyl 3-oxohept-6-ynoate (15) [21]

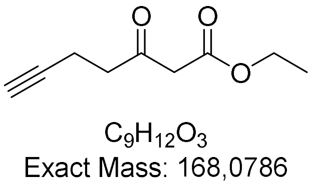

Prepared according to **General procedure VII**; purified by column chromatography eluting with hexane/EtOAc 9:1 (*v/v*). Yield 82%; Colorless oil. ^{1}H NMR (400 MHz, $CDCl_3$) δ 4.22 (q, J = 7.1 Hz, 2H), 3.49 (s, 2H), 2.84 (t, J = 7.2 Hz, 2H), 2.52-2.48 (m, 2H), 1.98 (s, 1H), 1.31 (t, J = 6.6Hz, 3H). ^{13}C NMR (101 MHz, $CDCl_3$) δ 200.56, 166.91, 82.52, 68.99, 61.32, 49.23, 41.62, 13.9, 12.7.

Ethyl (*Z*)-2-(pent-4-ynoyl)icos-11-enoate (16a)

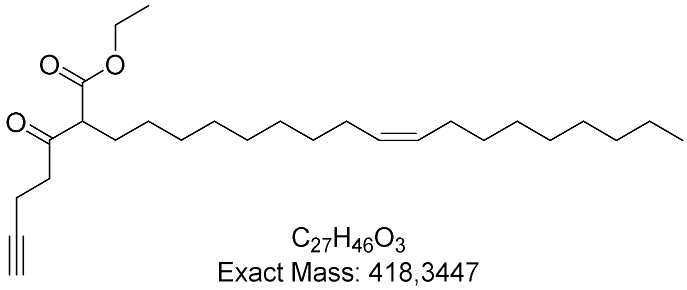

Prepared according to **General procedure VIII** starting from **15**; purified by column chromatography eluting with hexane/EtOAc 95:5 (*v/v*). Yield 83%; Colorless oil. ^{1}H NMR (200 MHz, $CDCl_3$) δ 5.43-5.24 (m, 2H), 4.18 (q, J = 7 Hz, 2H), 3.41 (t, J = 7 Hz, 1H), 2.86-2.65 (m, 2H), 2.55-2.35 (m, 2H), 2.08-1.74 (m, 6H), 1.93 (t, J = 3 Hz, 1H), 1.40-1.05 (m, 27H), 0.94 – 0.77 (m, 3H). ^{13}C NMR (50 MHz, $CDCl_3$) δ 203.0, 169.5, 129.8, 129.7, 82.6, 68.7, 61.2, 58.9, 40.4, 31.8, 29.7, 29.4, 29.3, 29.2, 29.1, 28.0, 27.3, 27.1, 22.6, 14.0, 12.7. HRMS *m/z* $[M+Na]^+$ calcd for $C_{27}H_{46}NaO_3$: 441.3339, found 441.3348.

Ethyl 2-(pent-4-ynoyl)tetradecanoate (16b)

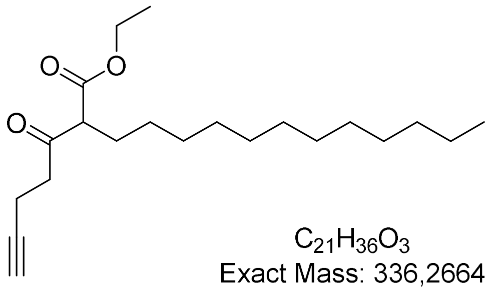

Prepared according to **General procedure VIII** starting from **15**; purified by column chromatography eluting with a gradient of hexane/EtOAc 98:2 to 9:1 (*v/v*). Yield 76%; Colorless oil. ^{1}H NMR (400 MHz, $CDCl_3$) δ 4.15 (q, J = 7.1 Hz, 2H), 3.45 (t, J = 7.4 Hz, 1H), 2.89-2.71 (m, 2H), 2.48 (td, J = 7.2, 2.4 Hz, 2H), 1.96 (t, J = 2.6 Hz, 1H), 1.31-1.27 (m, 25H), 0.90 (t, J = 6.8 Hz, 3H). ^{13}C NMR (101 MHz, $CDCl_3$) δ 203.26, 169.69, 82.77, 68.80, 61.37, 49.09, 40.48, 31.92, 29.64, 29.60, 29,54, 29.34, 29.32, 28.18, 27.40, 22.69, 14.12, 12.88. HRMS *m/z* $[M+Na]^+$ calcd for $C_{21}H_{36}NaO_3$: 359.2556, found 359.2555.

Ethyl (*Z*)-2-(1-hydroxypent-4-yn-1-yl)icos-11-enoate (17a)

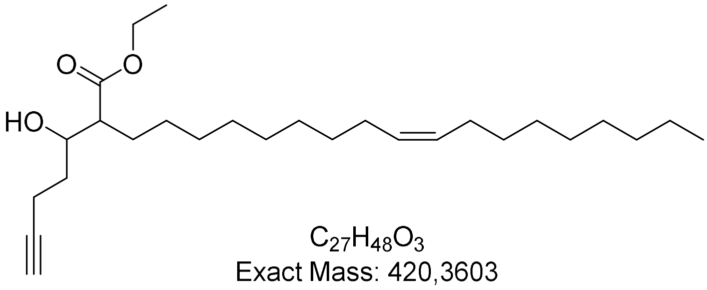

Prepared according to **General procedure IX** starting from **16a**; purified by column chromatography eluting with hexane/EtOAc 9:1 (*v/v*). Mixture of diastereomers. Yield 75%; Colorless oil. ^{1}H NMR (200 MHz, $CDCl_3$) δ 5.35-5.30 (m, 2H), 4.15 (q, J = 7 Hz, 2H), 3.96-3.84 (m, 0.5H), 3.83-3.70 (m, 0.5H) 2.79-2.64 (m, 1H), 2.50-2.25 (m, 3H), 2.09-1.83 (m, 5H), 1.62 (m, 4H), 1.38-1.13 (m, 27H), 0.91-0.76 (m, 3H). ^{13}C NMR (50 MHz, $CDCl_3$) δ 175.5, 175.2, 129.9, 129.7, 83.8, 70.9, 70.8, 68.8, 68.7, 60.5, 51.0, 50.7, 34.1, 32.8, 31.9, 29.7, 29.5, 29.4, 29.4, 29.3, 29.2, 27.6, 27.1, 22.6, 15.1, 15.0, 14.2, 14.0. HRMS *m/z* $[M+Na]^+$ calcd for $C_{27}H_{48}NaO_3$: 443.3496, found 443.3500.

Ethyl 2-(1-hydroxypent-4-yn-1-yl)tetradecanoate (17b)

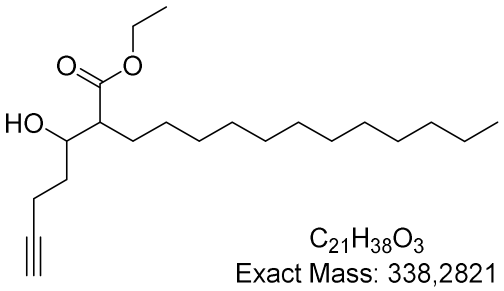

Prepared according to **General procedure VIII** starting from **16b**; purified by column chromatography eluting with hexane/EtOAc 9:1 (*v/v*). Mixture of diastereomers. Yield 69%; Colorless oil. ^{1}H NMR (400 MHz, $CDCl_3$) δ 4.23-4.17 (m, 2H), 3.98-3.93 (m, 0.5H), 3.86-3.82 (m. 0.5H), 2.50-2.43 (m, 1H), 2.42-2.35 (m, 2H), 1.99 (s, 1H), 1.78-1.56 (m, 4H), 1.34-1.28 (m, 23H), 0.90 (t, J = 6.6 Hz, 3H). ^{13}C NMR (101 MHz, $CDCl_3$) δ 175.64, 175.33, 83.89, 83.86, 70.96, 70.86, 68.82, 69.74, 60.60, 50.93, 50.74, 34.24, 32.79, 31.92, 29.67, 29.64, 29.60, 29.56, 29.48, 29.42, 29.35, 27.65, 27.25, 27.10, 22.69, 15.23, 15.10, 14.28, 14.12. HRMS *m/z* $[M+Na]^+$ calcd for $C_{21}H_{38}NaO_3$: 361.2713, found 361.2709.

(*Z*)-2-(1-Hydroxypent-4-yn-1-yl)icos-11-enoic acid (18a)

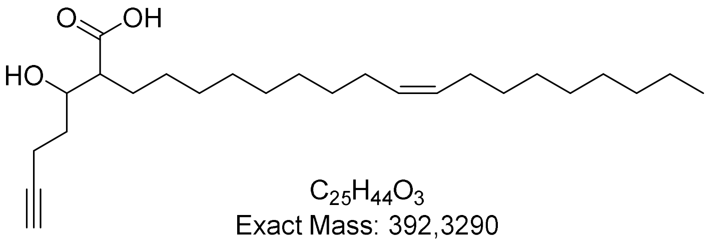

Prepared according to **General procedure X** starting from **17a**; purified by column chromatography eluting with CH_2Cl_2/MeOH 9:1 (*v/v*). Mixture of diastereomers. Yield 87%; Colorless oil. ^{1}H NMR (200 MHz, $CDCl_3$) δ 5.40-5.24 (m, 2H), 4.04-3.90 (m, 0.5H), 3.90-3.75 (m, 0.5H), 2.55-2.22 (m, 3H), 2.13-1.87 (m, 5H), 1.67 (m,

4H), 1.44-1.06 (m, 24H), 0.93-0.74 (m, 3H). ^{13}C NMR (50 MHz, $CDCl_3$) δ 180.6, 180.5, 129.9, 129.8, 83.6, 83.6, 70.8, 69.1, 69.0, 51.0, 50.8, 33.7, 32.4, 31.9, 29.73, 29.6, 29.5, 29.37, 29.3, 27.7, 27.2, 26.9, 22.6, 15.2, 15.0, 14.1. HRMS *m/z* [*M*+Na]$^+$ calcd for $C_{25}H_{44}NaO_3$: 415.3183, found 415.3185.

2-(1-Hydroxypent-4-yn-1-yl)tetradecanoic acid (18b)

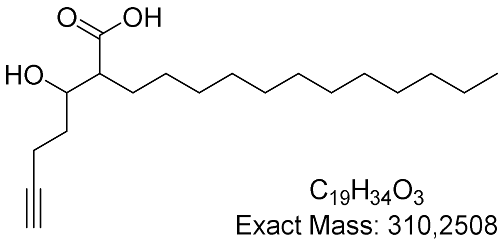

Prepared according to **General procedure X** starting from **17b**; purified by column chromatography eluting with CH_2Cl_2/MeOH 9:1 (*v/v*). Mixture of diastereomers. Yield 30%; Colorless oil. ^{1}H NMR (400 MHz, $CDCl_3$) δ 4.06-4.02 (m, 0.5H), 3.95-3.89 (m, 0.5H), 2.56-2.39 (m, 3H), 2.01 (s, 1H), 1.81-1.66 (m, 4H), 1.34-1.28 (m, 20H), 0.90 (t, J = 6.6 Hz, 3H). ^{13}C NMR (101 MHz, $CDCl_3$) δ 180.36, 83.66, 83.61, 70.84, 69.17, 69.10, 50.86, 50.73, 33.83, 32.43, 31.92, 29.67, 29.64, 29.59, 29.51, 29.42, 29.35, 27.70, 27.23, 26.94, 22.69, 15.28, 15.08, 14.12. HRMS *m/z* [*M*+Na]$^+$ calcd for $C_{19}H_{34}NaO_3$: 333.2400, found 333.2404.

***tert*-Butyl hept-6-ynoate (20)** [22]

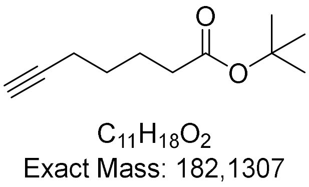

To a stirring solution of 6-heptynoic acid (1 mmol) in CH_2Cl_2 (0.2 mL), *t*-BuOH (2 mmol) and DMAP (0.05 mmol) were added and the reaction mixture was stirred for 10 min at room temperature. Then DCC (1.1 mmol) dissolved in CH_2Cl_2 (0.2 mL) was added and the reaction mixture was stirred for another 16 h at room temperature. After filtration of the solids and washing with CH_2Cl_2, the filtrate was washed with 0.5N HCl (2×5 mL), 1N $NaHCO_3$ (2×5 mL) and brine (5 mL) and the organic layer was dried. The solvent was removed and the product was purified by column chromatography eluting with CH_2Cl_2. Yield 75%; Yellowish oil. ^{1}H NMR (400 MHz, $CDCl_3$) δ 2.22-2.13 (m, 4H), 1.91 (t, J = 2.7 Hz, 1H), 1.70-1.62 (m, 2H), 1.57-1.47 (m, 2H), 1.40 (s, 9H). ^{13}C NMR (101 MHz, $CDCl_3$) δ 172.66, 83.93, 79.99, 68.48, 34.91, 28.03, 27.79, 24.10, 18.09.. HRMS *m/z* [*M*+Na]$^+$ calcd for $C_{11}H_{18}NaO_2$: 205.1188, found 205.1199.

***tert*-Butyl 7-(triisopropylsilyl)hept-6-ynoate (21)**

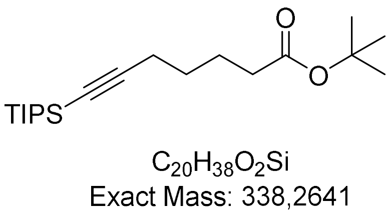

To a stirring solution of **20** (1 mmol) in dry THF (3 mL), under argon at -78 °C, a solution of 1.6M *n*-BuLi in hexane (1.2 mmol) was slowly added *via* syringe and the solution was stirred for 10 min. Then the -78 °C bath was replaced by an ice-water bath at 0 °C and TIPS-Cl (1.2 mmol) was slowly added. The reaction mixture's temperature was left to rise at room temperature and was stirred for 3.5 h while monitoring by TLC the consumption of the starting material. Then the reaction was quenched with sat. NH_4Cl (8 mL), THF was removed under reduced pressure and in the crude product H_2O (8 mL) and EtOAc (8 mL) were added. The aqueous layer was washed with additional EtOAc (2×8mL), the organic layers were combined, washed with brine (15 mL) and dried. The solvent was removed and the product was purified by column chromatography eluting with hexane/Et_2O 9:1 (*v/v*). Yield 77%; Yellow oil. ^{1}H NMR (400 MHz, $CDCl_3$) δ 2.30-2.21 (m, 4H), 1.77-1.68 (m, 2H), 1.60-1.53 (m, 2H), 1.44 (s, 9H), 1.12-1.00 (m, 21H). ^{13}C NMR (101 MHz, $CDCl_3$) δ 172.80, 108.48, 80.44, 79.94, 35.06, 28.15, 28.06, 24.23, 19.51, 18.59, 11.27. HRMS *m/z* [*M*+Na]$^+$ calcd for $C_{20}H_{38}NaO_2Si$: 361.2527, found 361.2533.

7-(Triisopropylsilyl)hept-6-ynoic acid (22)

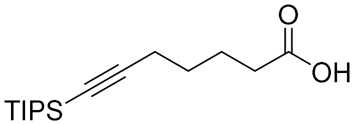

$C_{16}H_{30}O_2Si$
Exact Mass: 282,2015

At room temperature, **21** (1 mmol) is dissolved in a 15% TFA/CH_2Cl_2 (*v/v*) (12 mL) and the solution was stirred for 3 h while monitoring by TLC the consumption of the starting material. After 3 h the reaction was quenched with 1N aqueous ammonium acetate, the aqueous layer was washed with CH_2Cl_2 (3×20mL), the organic layers were combined, washed with brine (20 mL) and dried. The solvent was removed, and the product was purified by column chromatography eluting with hexane/EtOAc/AcOH 67/29/4 (*v/v*). Yield 75%; Colorless oil. ^{1}H NMR (400 MHz, $CDCl_3$) δ 2.41 (t, J = 7.4 Hz, 2H), 2.31 (t, J = 6.9 Hz, 2H), 1.81 (p, J = 7.4 Hz, 2H), 1.62 (p, J = 7.3 Hz, 2H), 1.13–1.03 (m, 21H). ^{13}C NMR (101 MHz, $CDCl_3$) δ 179.35, 108.19, 80.74, 33.38, 28.09, 23.69, 19.51, 18.61, 11.28. HRMS *m/z* [*M*-H]$^-$ calcd for $C_{16}H_{29}O_2Si$: 281.1933, found 281.1937.

2-(1-Hydroxybutyl)-7-(triisopropylsilyl)hept-6-ynoic acid (23)

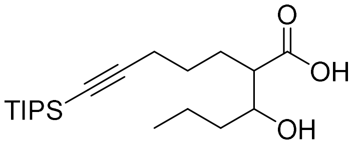

$C_{20}H_{38}O_3Si$
Exact Mass: 354,2590

Prepared according to **General procedure I** starting with **22**; purified by column chromatography eluting with CH_2Cl_2/MeOH 95:5 (*v/v*). Mixture of diastereomers (dr 7:3). Yield 67%; Yellow oil. ^{1}H NMR (400 MHz, $CDCl_3$) δ 3.94-3.89 (m, 0.3H), 3.80-3.73 (m, 0.7H), 2.56-2.45 (m, 1H), 2.33 (t, J = 6.6 Hz, 2H), 2.23 (s, 1H), 1.90-1.36 (m, 8H), 1.18-1.01 (m, 21H), 0.96 (t, J = 6.9, 3H). ^{13}C NMR (101 MHz, $CDCl_3$) δ 179.90, 107.99, 80.93, 72.15,

50.85, 37.31, 28.24, 26.33, 18.79, 18.61, 13.88, 11.26. HRMS *m/z* [*M*-H]$^-$ calcd for $C_{20}H_{37}O_3Si$: 353.2513, found 353.2512.

4-Propyl-3-(5-(triisopropylsilyl)pent-4-yn-1-yl)oxetan-2-one (24)

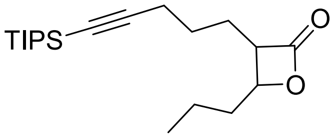

$C_{20}H_{36}O_2Si$
Exact Mass: 336,2485

Prepared according to **General procedure II** starting from **23**; purified by column chromatography eluting with hexane/EtOAc 9:1 (*v/v*). Mixture of diastereomers. Yield 40%; Yellow oil. ^{1}H NMR (400 MHz, $CDCl_3$) δ 4.58 (ddd, J = 10.1, 6.4, 4.0 Hz, 0.3H), 4.26 (ddd, J = 7.3, 6.0, 4.0 Hz, 0.7H), 3.70-3.62 (m, 0.3H), 3.26-3.19 (m, 0.7H), 2.34 (t, J = 6.8 Hz, 2H), 2.02-1.38 (m, 8H), 1.11-1.05 (m, 21H), 1.01 (t, J = 7.3 Hz, 3H). ^{13}C NMR (101 MHz, $CDCl_3$) δ 171.89, 171.15, 107.58, 107.46, 81.43, 81.36, 77.84, 75.32, 55.69, 52.16, 36.44, 32.26, 26.84, 26.40, 26.05, 22.98, 19.59, 19.51, 18.86, 18.61, 18.41, 13.79, 13.74, 11.25. HRMS *m/z* [*M*+Na]$^+$ calcd for $C_{20}H_{36}NaO_2Si$: 359.2375, found 359.2377.

11-Iodoundecanoic acid (26) [23]

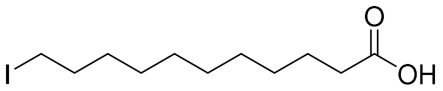

$C_{11}H_{21}IO_2$
Exact Mass: 312,0586

To a stirring solution of 11-bromoundecanoic acid (1 mmol) in acetone (8 mL), under argon at room temperature, NaI (3 mmol) was added and the solution was stirred at room temperature for 16 h. Then acetone was removed under reduced pressure and in the crude product H_2O (10 mL) and CH_2Cl_2 (10 mL) were added. The aqueous layer was washed with additional CH_2Cl_2 (10 mL), the organic layers were combined, washed with 10% $Na_2S_2O_3$ (20 mL), brine (20 mL) and dried. The solvent was removed and the product was used it the next step without further purification. Yield 95%; Yellow solid. ^{1}H NMR (400 MHz, $CDCl_3$) δ 3.21 (t, J = 7 Hz, 2H), 2.37 (t, J = 7,6 Hz, 2H), 1.88-1.81 (m, 2H), 1.69-1.62 (m, 2H), 1.44-1.28 (m, 12H). ^{13}C NMR (101 MHz, $CDCl_3$) δ 179.06, 33.85, 33.55, 30.49, 29.33, 29.30, 29.18, 29.02, 28.50, 24.66, 7.30.

13-(Triisopropylsilyl)tridec-12-ynoic acid (27)

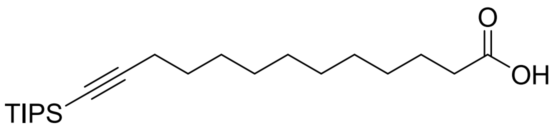

$C_{22}H_{42}O_2Si$
Exact Mass: 366,2954

To a stirring solution of TIPS-acetylene (1.8 mmol) in dry THF (0.9 mL), under argon at -78 °C, a solution of 1.6M *n*-BuLi in hexane (3 mmol) was slowly added via syringe and the solution was stirred for 30 min at -78 °C. Then a solution of **26** (1 mmol) in dry THF (6 mL) and dry HMPS (2 mL) was slowly added. The reaction

mixture was stirred for 3 h at -78 °C. Then the reaction was quenched with sat. NH_4Cl (10 mL) and the aqueous layer was washed with EtOAc (3×10 mL). The organic layers were combined, washed with brine (30 mL) and dried. The solvent was removed, and the product was purified by column chromatography eluting with a gradient of CH_2Cl_2/MeOH 99:1 to 97:3 (*v/v*). Yield 62%; Colorless oil. ^1H NMR (400 MHz, $CDCl_3$) δ 2.37 (t, J = 7.4 Hz, 2H), 2.26 (t, J = 7Hz, 2H), 1.69-1.61 (m, 2H), 1.58-1.50 (m, 2H), 1.45-1.30 (m, 12H) 1.09-1.05 (m, 21H). ^{13}C NMR (101 MHz, $CDCl_3$) δ 179.17, 109.33, 79.98, 33.89, 29.47, 29.35, 29.23, 29.10, 29.02, 29.83, 29.65, 24.69, 19.82, 18.64, 11.30. HRMS *m/z* [*M*+Na]$^+$ calcd for $C_{21}H_{40}NaO_2Si$: 389.2846, found 389.2854.

2-(1-Hydroxybutyl)-13-(triisopropylsilyl)tridec-12-ynoic acid (28)

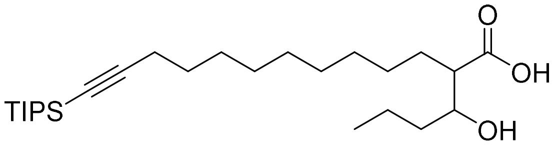

$C_{26}H_{50}O_3Si$
Exact Mass: 438,3529

Prepared according to **General procedure VI** starting with **27**; purified by column chromatography eluting with a gradient of CH_2Cl_2/MeOH 98:2to 9:1 (*v/v*). Mixture of diastereomers (dr 6:4). Yield 66%; Yellow oil. ^1H NMR (400 MHz, $CDCl_3$) δ 3.89-3.86 (m, 0.4H), 3.77-3.72 (m, 0.6H), 2.50-2.44 (m, 1H), 2.25 (t, J = 7.0 Hz, 2H), 1.78-1.27 (m, 20H), 1.09–1.07 (m, 21H), 0.96 (t, J = 7.2 Hz, 3H). ^{13}C NMR (101 MHz, $CDCl_3$) δ 180.48, 109.32, 79.97, 71.89, 71.82, 50.99, 50.85, 37.58, 36.14, 29.63, 29.52, 29.50, 29.48, 29.45, 29.38, 29.36, 29.02, 28.84, 28.66, 27.82, 26.67, 19.81, 19.17, 18.91, 18.63, 13.93, 11.31. HRMS *m/z* [*M*+H]$^+$ calcd for $C_{26}H_{51}O_3Si$: 439.3602, found 439.3606.

4-Propyl-3-(11-(triisopropylsilyl)undec-10-yn-1-yl)oxetan-2-one (29)

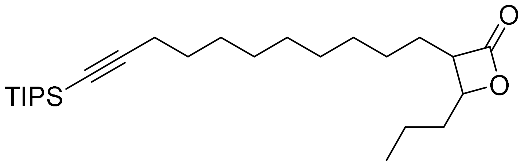

$C_{26}H_{48}O_2Si$
Exact Mass: 420,3424

Prepared according to **General procedure II** starting from **28**; purified by column chromatography eluting with a gradient of hexane/EtOAc 99:1 to 9:1 (*v/v*). Mixture of diastereomers. Yield 71%; Yellow oil. ^1H NMR (400 MHz, $CDCl_3$) δ 4.59-4.54 (m, 0.4H), 4.27-4.22 (m, 0.6H) 3.64-3.59 (m, 0.4H), 3.21-3.16 (m, 0.6H), 2.26 (t, J = 6.8 Hz, 2H), 1.85-1.28 (m, 20H), 1.09-1.06 (m, 21H), 1.0 (t, J = 7.4 Hz, 3H). ^{13}C NMR (101 MHz, $CDCl_3$) δ 172.33, 171.65, 109.27, 80.01, 77.97, 75.49, 56.19, 52.68, 36.52, 36.28 32.23, 29.43, 29.40, 29.29, 29.25, 28.99, 28.81, 28.61, 27.90, 27.62, 27.02, 23.95, 19.81, 18.91, 18.64, 18.45, 18.39, 13.82, 13.78, 11.31. HRMS *m/z* [*M*+H]$^+$ calcd for $C_{26}H_{49}NaO_2Si$: 421.3497, found 421.3506.

Undec-2-yn-1-ol (31a) [24]

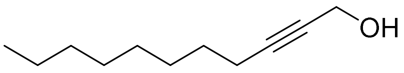

$C_{11}H_{20}O$
Exact Mass: 168,1514

Prepared according to **General procedure XIV** starting from bromooctane; purified by column chromatography eluting with hexane/EtOAc 9:1 (*v/v*). Yield 83%; Yellow oil. ^{1}H NMR (400 MHz, $CDCl_3$) δ 4.27 (s, 2H), 2.23 (t, J = 7.2Hz, 2H), 1.56-1.49 (m, 2H), 1.41-1.30 (m, 10H), 0.90 (t, J = 6.4 Hz, 3H). ^{13}C NMR (101 MHz, $CDCl_3$) δ 86.73, 78.25, 51.48, 31.85, 29.19, 29.11, 28.89, 28.61, 22,67, 18.75, 14.11.

Dec-2-yn-1-ol (31b) [25]

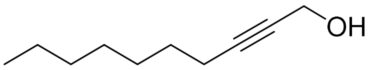

$C_{10}H_{18}O$
Exact Mass: 154,1358

Prepared according to **General procedure XIV** starting from bromoheptane; purified by column chromatography eluting with hexane/EtOAc 9:1 (*v/v*). Yield 84%; Yellow oil. ^{1}H NMR (400 MHz, $CDCl_3$) δ 4.27 (s, 2H), 2.23 (t, J = 7.2 Hz, 2H) , 1.56-1.49 (m, 2H), 1.41-1.29 (m, 8H), 0.91 (t, 6,6 Hz, 3H). ^{13}C NMR (101 MHz, $CDCl_3$) δ 86.72, 78.25, 51.47, 31.73, 28.84, 28.81, 28.61, 22,63, 18.74, 14.08.

Undec-10-yn-1-ol (32a) [26]

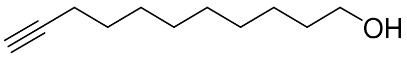

$C_{11}H_{20}O$
Exact Mass: 168,1514

Prepared according to **General procedure XV** starting from **31a**; purified by column chromatography eluting with a gradient of hexane/EtOAc 95:5 to 8:2 (*v/v*). Yield 65%; Yellow oil. ^{1}H NMR (400 MHz, $CDCl_3$) δ 3.66 (t, J = 6.8 Hz, 2H), 2.20 (td, J = 7.0, 3.3 Hz, 2H), 1.96 (t, J = 2.8Hz, 1H), 1.62-1.31 (m, 14H). ^{13}C NMR (101 MHz, $CDCl_3$) δ 86.79, 68.07, 63.08, 32.79, 29.44, 29.36, 29.03, 28.72, 28.47, 25.11, 18.40.

Dec-9-yn-1-ol (32b) [27]

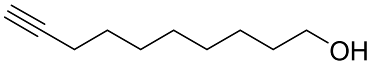

$C_{10}H_{18}O$
Exact Mass: 154,1358

Prepared according to **General procedure XV** starting from **31b**; purified by column chromatography eluting with hexane/EtOAc 9:1 (*v/v*). Yield 66%; Yellow oil. ^{1}H NMR (400 MHz, $CDCl_3$) δ 3.67 (t, J = 6.8 Hz, 2H), 2.21 (td, J = 7.0, 2.8 Hz, 2H), 1.96 (t, J = 2.8Hz, 1H), 1.60-1.32 (m, 12H). ^{13}C NMR (101 MHz, $CDCl_3$) δ 86.78, 68.11, 63.08, 32.78, 29.28, 29.07, 26.68, 24.46, 25.69, 18.40.

Undec-10-ynoic acid (33a) [28]

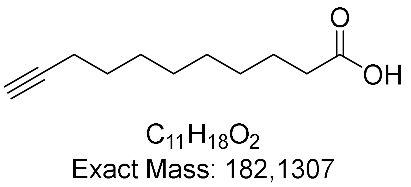

Prepared according to **General procedure XVI** starting from **32a**; purified by column chromatography eluting with a gradient of hexane/EtOAc 9:1 to 8:2 (*v/v*). Yield 81%; White solid. ^{1}H NMR (400 MHz, $CDCl_3$) δ 2.37 (t, J = 7.4 Hz, 2H), 2.20 (t, J = 7.0 Hz, 2H), 1.96 (s, 1H), 1.69-1.62 (m, 2H), 1.58-1.51 (m, 2H), 1.43-1.32 (m, 8H). ^{13}C NMR (101 MHz, $CDCl_3$) δ 178.87, 84.73, 68.10, 33.82, 29.08, 28.98, 28.88, 28.65, 28.43, 28.65, 18.38.

Dec-9-ynoic acid (33b) [29]

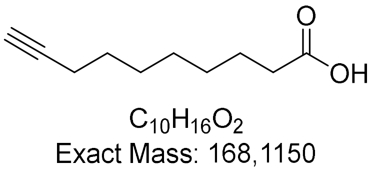

Prepared according to **General procedure XVI** starting from **32b**; purified by column chromatography eluting with a gradient of hexane/EtOAc 9:1 to 8:2 (*v/v*). Yield 81%; Colorless oil. ^{1}H NMR (400 MHz, $CDCl_3$) δ 2.29 (t, J = 7.5 Hz, 2H), 2.12 (td, J = 7.0, 2.8 Hz, 2H), 1.87 (t, J = 2.5Hz, 1H), 1.60-1.54 (m, 2H), 1.49-1.54 (m, 2H), 1.37-1.24 (m, 6H). ^{13}C NMR (101 MHz, $CDCl_3$) δ 178.87, 84.73, 68.10, 33.82, 29.08, 28.98, 28.88, 28.65, 28.43, 28.65, 18.38.

2-(1-Hydroxybutyl)-11-(triisopropylsilyl)undec-10-ynoic acid (34a)

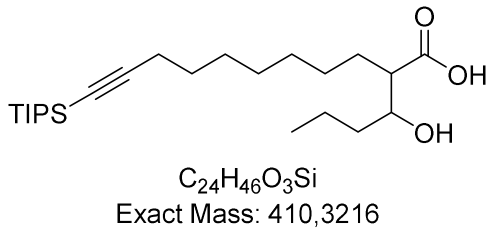

Prepared according to **General procedure I** starting with **33a**; purified by column chromatography eluting with hexane/EtOAc 7:3 (*v/v*). Mixture of diastereomers (dr 6:4). Yield 41%; Yellow oil. ^{1}H NMR (400 MHz, $CDCl_3$) δ 3.91-3.87 (m, 0.4H), 3.78-3.73 (m, 0.6H), 2.523-2.46 (m, 1H), 2.26 (t, J = 7.0 Hz, 2H), 1.80-1.28 (m, 16H), 1.10–1.06 (m, 21H), 0.97 (t, J = 7.2 Hz, 3H). ^{13}C NMR (101 MHz, $CDCl_3$) δ 179.36, 109.22, 80.05, 71.86, 71.82, 50.80, 50.68, 37.65, 36.12, 29.55, 29.47, 29.43, 28.88, 28.80, 28.65, 28.61, 27.78, 27.31, 26.69, 19.80, 19.17, 18.93, 18.63, 13.91, 11.31. HRMS *m/z* [*M*+Na]$^+$ calcd for $C_{24}H_{46}NaO_3Si$: 433.3108, found 433.3114.

2-(1-Hydroxybutyl)-10-(triisopropylsilyl)dec-9-ynoic acid (34b)

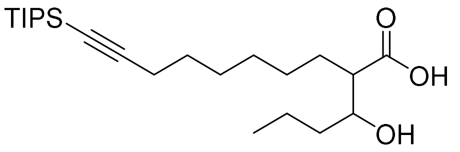

$C_{23}H_{44}O_3Si$
Exact Mass: 396,3060

Prepared according to **General procedure I** starting with **33b**; purified by column chromatography eluting with hexane/EtOAc 7:3 (*v/v*). Mixture of diastereomers (dr 6:4). Yield 44%; Yellow oil. ^{1}H NMR (400 MHz, $CDCl_3$) δ 3.91-3.86 (m, 0.4H), 3.77-3.74 (m, 0.6H), 2.52-2.45 (m, 1H), 2.26 (t, J = 6.6 Hz, 2H), 1.77-1.28 (m, 14H), 1.09-1.05 (m, 21H), 0.96 (t, J = 7.0 Hz, 3H). ^{13}C NMR (101 MHz, $CDCl_3$) δ 179.97, 109.13, 109.10, 80.14, 80.12, 71.85, 71.81, 50.92, 50.80, 37.61, 36.14, 29.70, 29.37, 29.07, 28.96, 28.75, 28.73, 28.47, 28.43, 27.74, 27.25, 26.66, 19.17, 19.16, 18.92, 18.63, 13.91, 11.31. HRMS *m/z* [*M*+Na]$^+$ calcd for $C_{23}H_{44}NaO_3Si$: 419.2952, found 419.2950.

4-Propyl-3-(9-(triisopropylsilyl)non-8-yn-1-yl)oxetan-2-one (35a)

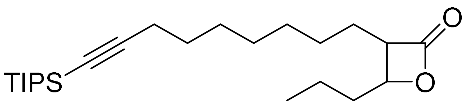

$C_{24}H_{44}O_2Si$
Exact Mass: 392,3111

Prepared according to **General procedure II** starting from **34a**; purified by column chromatography eluting with hexane/EtOAc 95:5 (*v/v*). Mixture of diastereomers (dr 9:1). Yield 48%; Colorless oil. ^{1}H NMR (400 MHz, $CDCl_3$) δ 4.26-4.22 (m, 1H), 3.20-3.16 (m, 1H), 2.26 (t, J = 6.8 Hz, 2H), 1.91-1.80 (m, 2H), 1.77-1.68 (m, 2H), 1.56-1.32 (m, 12H), 1.09-1.07 (m, 21H), 1.01 (t, J = 7.4 Hz, 3H). ^{13}C NMR (101 MHz, $CDCl_3$) δ 171.61, 109.12, 80.11, 77.95, 56.17, 36.52, 29.23, 28.77, 28.74, 28.53, 27.89, 26.96, 19.77, 18.63, 18.45, 13.78, 11.30. HRMS *m/z* [*M*+Na]$^+$ calcd for $C_{24}H_{44}NaO_2Si$: 415.3033, found 415.3003.

4-Propyl-3-(8-(triisopropylsilyl)oct-7-yn-1-yl)oxetan-2-one (35b)

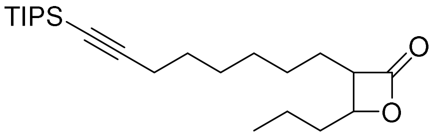

$C_{23}H_{42}O_2Si$
Exact Mass: 378,2954

Prepared according to **General procedure II** starting from **34b**; purified by column chromatography eluting with hexane/EtOAc 95:5 (*v/v*). Mixture of diastereomers (dr 8:2). Yield 48%; Colorless oil. ^{1}H NMR (400 MHz, $CDCl_3$) δ 4.59-4.55 (m, 0.2H), 4.27-4.22 (m, 0.8H) 3.64-3.59 (m, 0.2H), 3.21-3.16 (m, 0.8H), 2.27 (t, J = 6.6Hz, 2H), 1.90-1.80 (m, 2H), 1.77-1.70 (m, 2H), 1.60-1.38 (m, 10H), 1.09-1.05 (m, 21H), 1.01 (t, J = 7.6 Hz, 3H). ^{13}C NMR (101 MHz, $CDCl_3$) δ 171.56, 108.95, 80.55, 77.95, 56.17, 36.51, 28.74, 28.62, 28.32, 28.32, 27.84, 26.94, 21.06, 18.64, 18.45, 13.78, 11.30. HRMS *m/z* [*M*+Na]$^+$ calcd for $C_{23}H_{42}NaO_2Si$: 401.2846, found 401.2845.

10-Bromodecan-1-ol (37) [30]

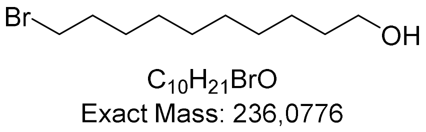

To a stirring solution of 1,10-decanediol (1 mmol) in toluene (2 mL), conc. HBr (1.26 mmol) was added and the reaction mixture was refluxed for 16 h. Then Et_2O (30 mL) was added and the organic layer was washed with H_2O (30 mL), 1N NaOH (30 mL) and then brine (30 mL) and was dried. The solvent was removed and the product was purified by column chromatography eluting with a gradient of hexane/EtOAc 9:1 to 8:2 (*v/v*). Yield 68%; Colorless oil. ^{1}H NMR (400 MHz, $CDCl_3$) δ δ 3.66 (t, J = 6.6Hz, 2H), 3.43 (t, J = 7.0Hz, 2H), 1.91-1.87 (m, 2H), 1.62-1.55 (m, 2H), 1.46-1.32 (m, 12H). ^{13}C NMR (101 MHz, $CDCl_3$) δ 63.08, 34.05, 32.83, 32.79, 29.47, 29.37, 28.74, 28.16, 25.72.

10-Azidodecan-1-ol (38) [31]

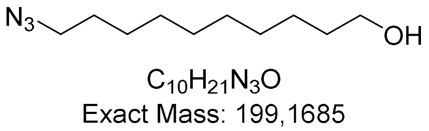

To a stirring solution of **37** (1 mmol) in benzene (4 mL), TBAB (0.1 mmol) and NaN_3 (2 mmol) were added, and the reaction mixture was refluxed for 16 h. Then the crude mixture was filtered, and the solvent of the filtrate was removed under *vacuo*. The product was purified by column chromatography eluting with CH_2Cl_2/EtOAc 1:1 (*v/v*). Yield 95%; Colorless oil. ^{1}H NMR (400 MHz, $CDCl_3$) δ 3.67 (t, J = 6.6 Hz, 2H) 3.28 (t, J = 6.8 Hz, 2H), 1.66-1.56 (m, 4H), 1.44-1.28 (m, 12H). ^{13}C NMR (101 MHz, $CDCl_3$) δ 63.07, 51.48, 32.19, 29.46, 29.39, 29.37, 29.12, 28.83, 26.70, 25.72. HRMS *m/z* [*M*+Na]$^+$ calcd for $C_{10}H_{21}NaN_3O$: 222.1577, found 222.1588.

10-Azidodecanoic acid (39) [32]

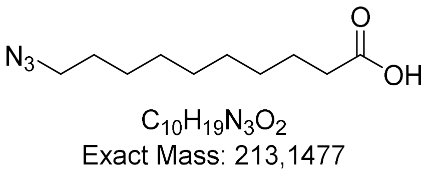

Prepared according to **General procedure XVI** starting from **38**; purified by column chromatography eluting with hexane/EtOAc 8:2 (*v/v*). Yield 71%; Colorless oil. ^{1}H NMR (400 MHz, $CDCl_3$) δ 3.28 (t, J = 7.0 Hz, 2H) 2.37 (t, J = 7.4 Hz, 2H),1.69-1.58 (m, 4H), 1.40-1.33 (m, 10H). ^{13}C NMR (101 MHz, $CDCl_3$) δ 179.75, 51.48, 33.96, 29.24, 29.11, 29.06, 28.99, 28.82, 26.68, 24.63. HRMS *m/z* [*M*+Na]$^+$ calcd for $C_{10}H_{19}N_3NaO_2$i: 236.1369, found 236.1370.

Table S1. Code numbers, structures and calculated octanol-water partition coefficient Log $P_{O/W}$ of tested compounds. Log $P_{o/w}$ were computed using the iLOGP method[33] developed by the SwissADME web tool (http://www.swissadme.ch/).[34]

Code	Structure	Log $P_{o/w}$
**VM001**	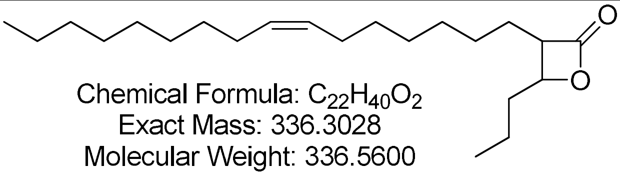	6.70
**VM008**	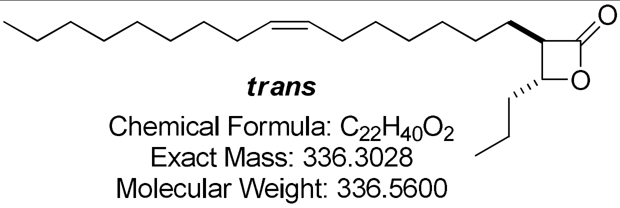	6.56
**VM009**	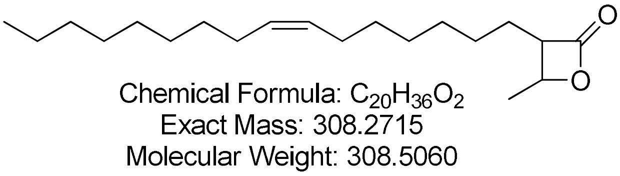	5.91
**VM010**	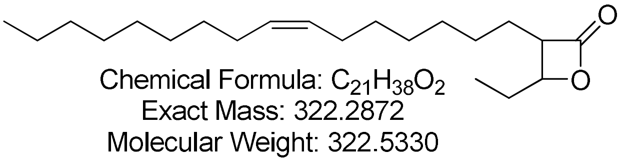	6.37
**VM011**	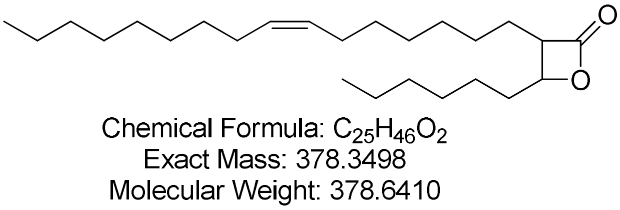	7.60
**VM012**	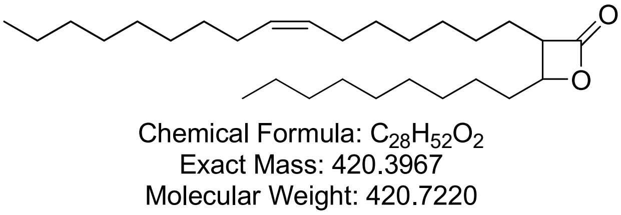	8.75
**VM013**	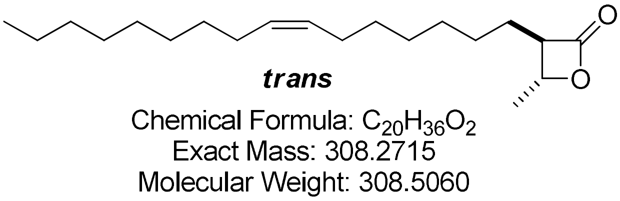	5.91

**VM017**	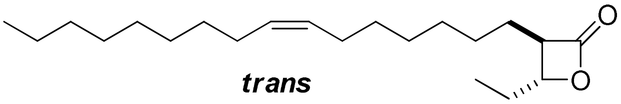 *trans* Chemical Formula: $C_{21}H_{38}O_2$ Exact Mass: 322.2872 Molecular Weight: 322.5330	6.37
**VM018**	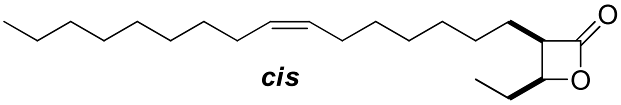 *cis* Chemical Formula: $C_{21}H_{38}O_2$ Exact Mass: 322.2872 Molecular Weight: 322.5330	6.37
**VM019**	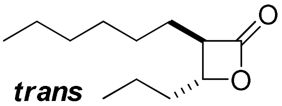 *trans* Chemical Formula: $C_{12}H_{22}O_2$ Exact Mass: 198.1620 Molecular Weight: 198.3060	3.38
**VM020**	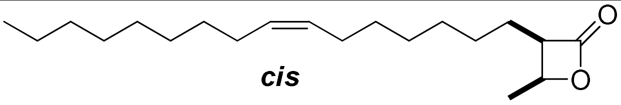 *cis* Chemical Formula: $C_{20}H_{36}O_2$ Exact Mass: 308.2715 Molecular Weight: 308.5060	5.91
**VM021**	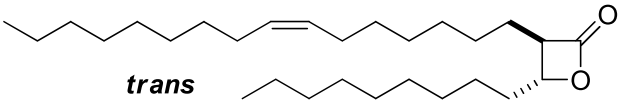 *trans* Chemical Formula: $C_{28}H_{52}O_2$ Exact Mass: 420.3967 Molecular Weight: 420.7220	8.87
**VM022**	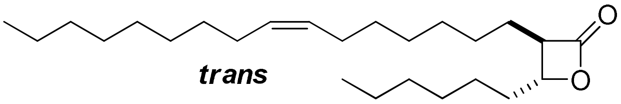 *trans* Chemical Formula: $C_{25}H_{46}O_2$ Exact Mass: 378.35 Molecular Weight: 378.63	7.78
**VM023**	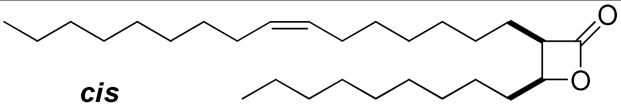 *cis* Chemical Formula: $C_{28}H_{52}O_2$ Exact Mass: 420.3967 Molecular Weight: 420.7220	8.87

**VM024**	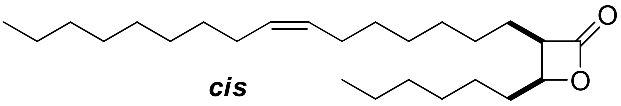 ***cis*** Chemical Formula: $C_{25}H_{46}O_2$ Exact Mass: 378.3498 Molecular Weight: 378.6410	7.78
**VM025**	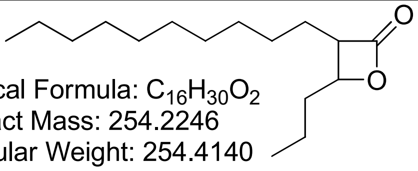 Chemical Formula: $C_{16}H_{30}O_2$ Exact Mass: 254.2246 Molecular Weight: 254.4140	4.82
**VM026**	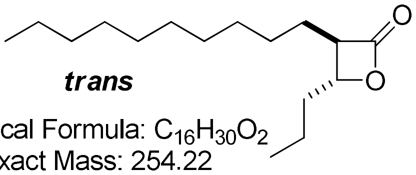 ***trans*** Chemical Formula: $C_{16}H_{30}O_2$ Exact Mass: 254.22 Molecular Weight: 254.41	4.82
**VM027**	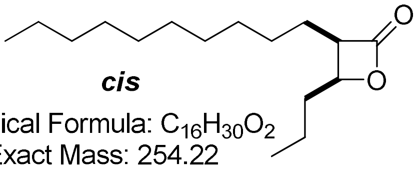 ***cis*** Chemical Formula: $C_{16}H_{30}O_2$ Exact Mass: 254.22 Molecular Weight: 254.41	4.82
**VM028**	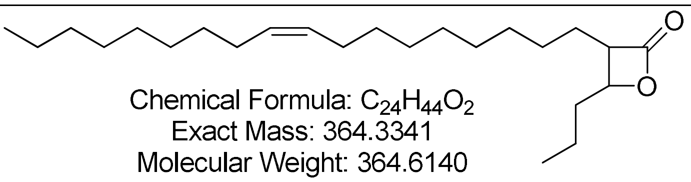 Chemical Formula: $C_{24}H_{44}O_2$ Exact Mass: 364.3341 Molecular Weight: 364.6140	7.30
**VM029**	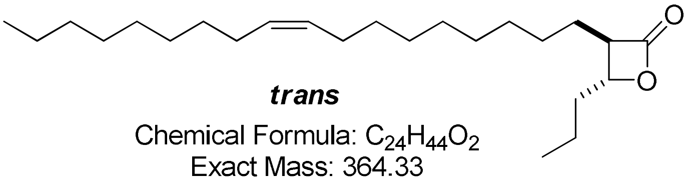 ***trans*** Chemical Formula: $C_{24}H_{44}O_2$ Exact Mass: 364.33 Molecular Weight: 364.60	7.30
**VM030**	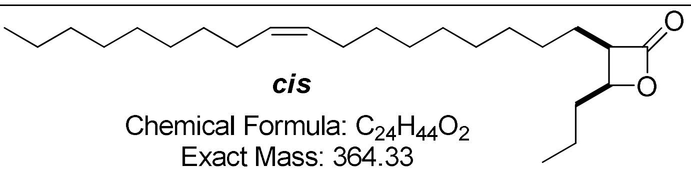 ***cis*** Chemical Formula: $C_{24}H_{44}O_2$ Exact Mass: 364.33 Molecular Weight: 364.60	7.30
**VM035*p***	Chemical Formula: $C_{25}H_{42}O_2$ Exact Mass: 374.3185 Molecular Weight: 374.6090 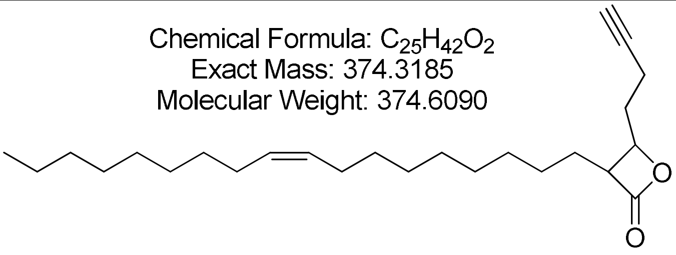	7.37

**VM036**	Chemical Formula: $C_{23}H_{42}O_2$<br>Exact Mass: 350.3185<br>Molecular Weight: 350.5870<br>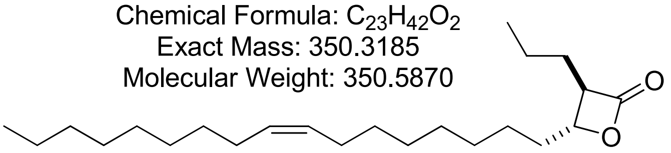<br>***trans***	7.07
**VM037**	Chemical Formula: $C_{23}H_{42}O_2$<br>Exact Mass: 350.32<br>Molecular Weight: 350.58<br>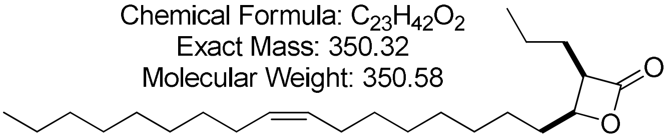<br>***cis***	7.07
**VM038**	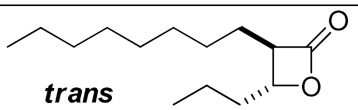<br>***trans***<br>Chemical Formula: $C_{14}H_{26}O_2$<br>Exact Mass: 226.1933<br>Molecular Weight: 226.3600	4.12
**VM039**	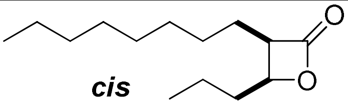<br>***cis***<br>Chemical Formula: $C_{14}H_{26}O_2$<br>Exact Mass: 226.1933<br>Molecular Weight: 226.3600	4.12
**VM040**	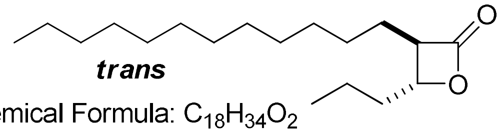<br>***trans***<br>Chemical Formula: $C_{18}H_{34}O_2$<br>Exact Mass: 282.2559<br>Molecular Weight: 282.4680	5.58
**VM041**	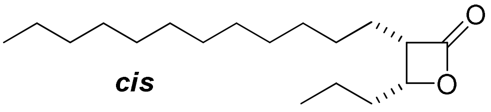<br>***cis***<br>Chemical Formula: $C_{18}H_{34}O_2$<br>Exact Mass: 282.2559<br>Molecular Weight: 282.4680	5.58
**VM042**	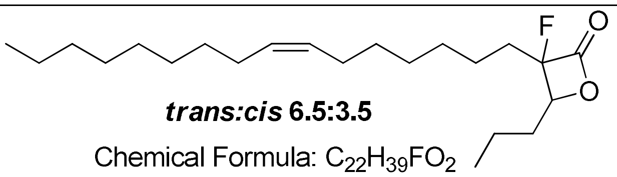<br>***trans:cis* 6.5:3.5**<br>Chemical Formula: $C_{22}H_{39}FO_2$<br>Exact Mass: 354.2934<br>Molecular Weight: 354.5504	6.92
**VM043**	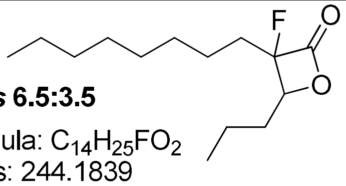<br>***trans:cis* 6.5:3.5**<br>Chemical Formula: $C_{14}H_{25}FO_2$<br>Exact Mass: 244.1839<br>Molecular Weight: 244.3504	4.31

**VM044**	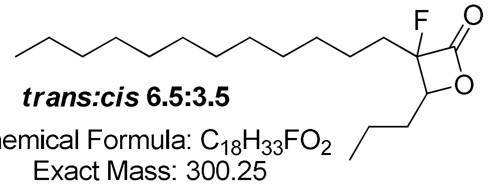 ***trans:cis* 6.5:3.5** Chemical Formula: $C_{18}H_{33}FO_2$ Exact Mass: 300.25 Molecular Weight: 300.45	5.80
**VM045**	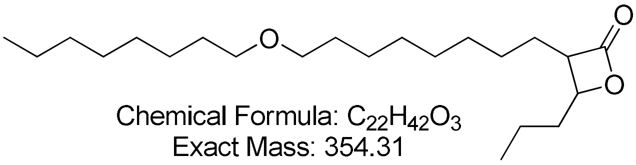 Chemical Formula: $C_{22}H_{42}O_3$ Exact Mass: 354.31 Molecular Weight: 354.57	6.19
**VM046**	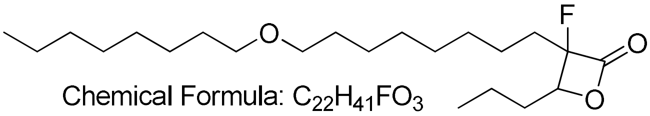 Chemical Formula: $C_{22}H_{41}FO_3$ Exact Mass: 372.3040 Molecular Weight: 372.5654	6.46
**VM047**	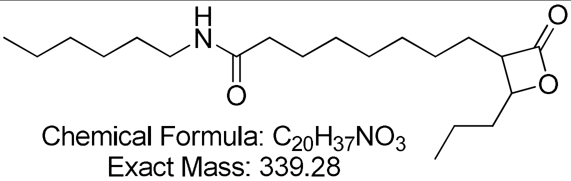 Chemical Formula: $C_{20}H_{37}NO_3$ Exact Mass: 339.28 Molecular Weight: 339.51	4.74
**VM048**	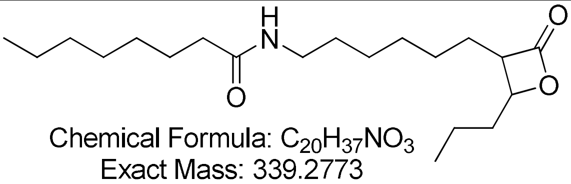 Chemical Formula: $C_{20}H_{37}NO_3$ Exact Mass: 339.2773 Molecular Weight: 339.5200	4.74
**VM049*p***	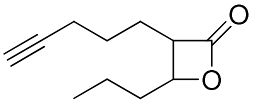 Chemical Formula: $C_{11}H_{16}O_2$ Exact Mass: 180.1150 Molecular Weight: 180.2470	2.57
**VM050*p***	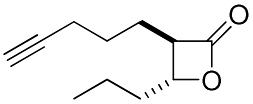 Chemical Formula: $C_{11}H_{16}O_2$ Exact Mass: 180.1150 Molecular Weight: 180.2470	2.57
**VM051*p***	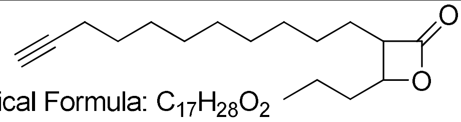 Chemical Formula: $C_{17}H_{28}O_2$ Exact Mass: 264.2089 Molecular Weight: 264.4090	4.75

**VM052$_p$**	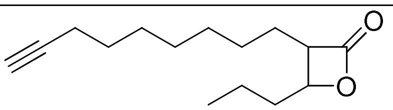<br>Chemical Formula: $C_{15}H_{24}O_2$<br>Exact Mass: 236.1776<br>Molecular Weight: 236.3550	4.04
**VM053$_p$**	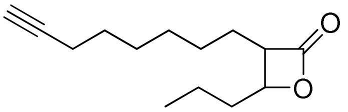<br>Chemical Formula: $C_{14}H_{22}O_2$<br>Exact Mass: 222.1620<br>Molecular Weight: 222.3280	3.68
**VM054$_p$**	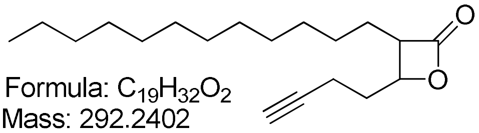<br>Chemical Formula: $C_{19}H_{32}O_2$<br>Exact Mass: 292.2402<br>Molecular Weight: 292.4630	5.53
**VM055$_p$**	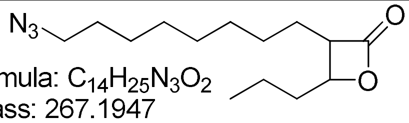<br>Chemical Formula: $C_{14}H_{25}N_3O_2$<br>Exact Mass: 267.1947<br>Molecular Weight: 267.3730	3.72
**VM056**	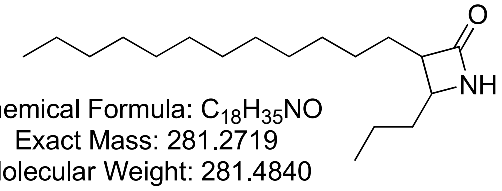<br>Chemical Formula: $C_{18}H_{35}NO$<br>Exact Mass: 281.2719<br>Molecular Weight: 281.4840	5.29
**VM057**	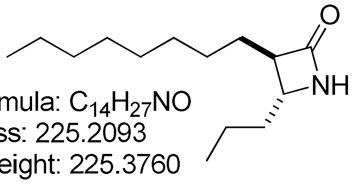<br>Chemical Formula: $C_{14}H_{27}NO$<br>Exact Mass: 225.2093<br>Molecular Weight: 225.3760	3.81
**VM058**	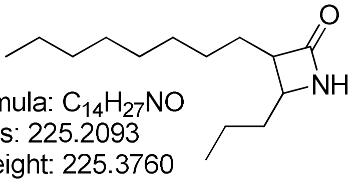<br>Chemical Formula: $C_{14}H_{27}NO$<br>Exact Mass: 225.2093<br>Molecular Weight: 225.3760	3.81

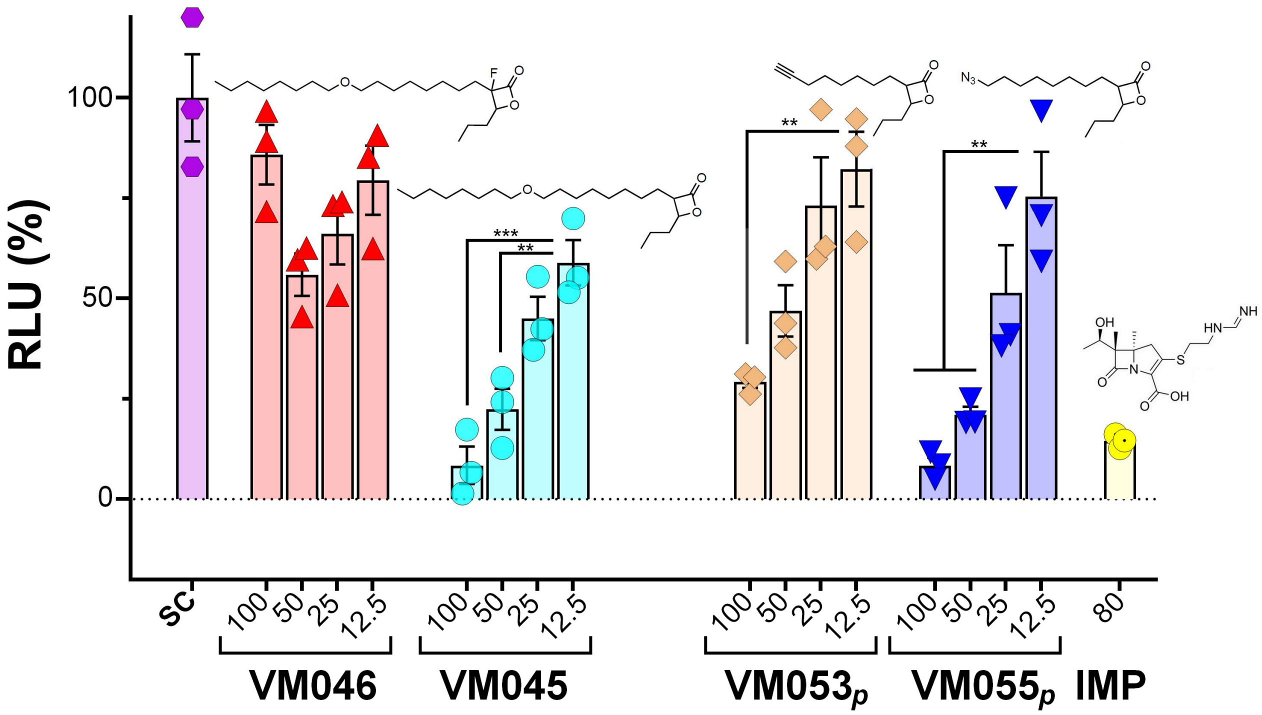

Figure S1. Intracellular activity of **VM045**, **VM046**, as well as the two probes **VM053*p*** and **VM055*p*** against Raw264.7 infected macrophages by *M. abscessus* S, as compared to imipenem (IMP). The activity of selected β-lactones on intracellular *M. abscessus* was tested in Raw264.7 murine macrophages. Cells were infected at a multiplicity of infection (MOI) of 10 with *M. abscessus* S-LuxG13 and treated with various concentrations of each inhibitor or IMP for 24 h. The viable mycobacteria were quantified by measurement of luminescence from luciferase-expressing *M. abscessus* S-LuxG13 within Raw264.7 macrophages. Untreated infected macrophages were used as control representing 100% of bacterial viability. Untreated infected macrophages were used as control representing 100% of bacterial viability. Results are shown as mean ± standard error of the mean (SEM) of three independent assays performed in triplicate. SC, solvent control (DMSO). ***, *p*-value <0.001. **, *p*-value <0.01. Statistical analysis was done using a Student's *t*-test.

^{1}H, ^{13}C and ^{19}F NMR spectra of final compounds

VM009

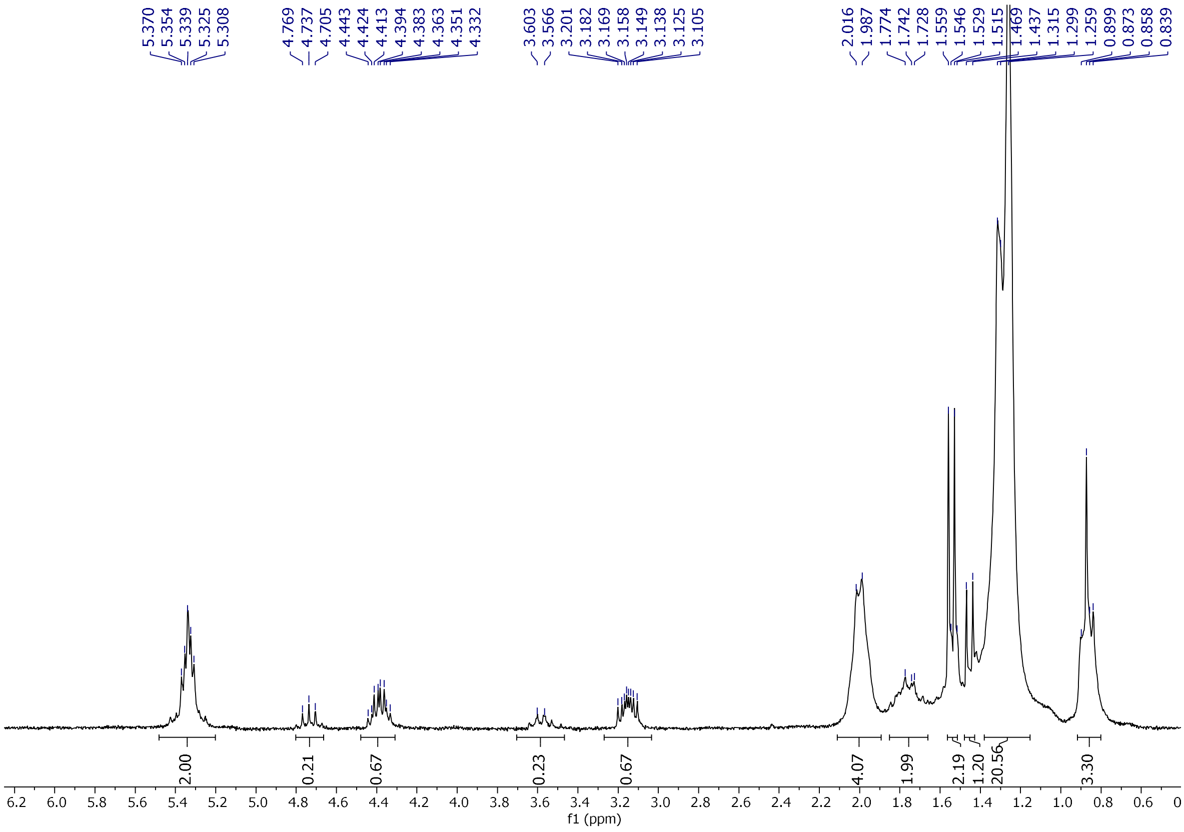

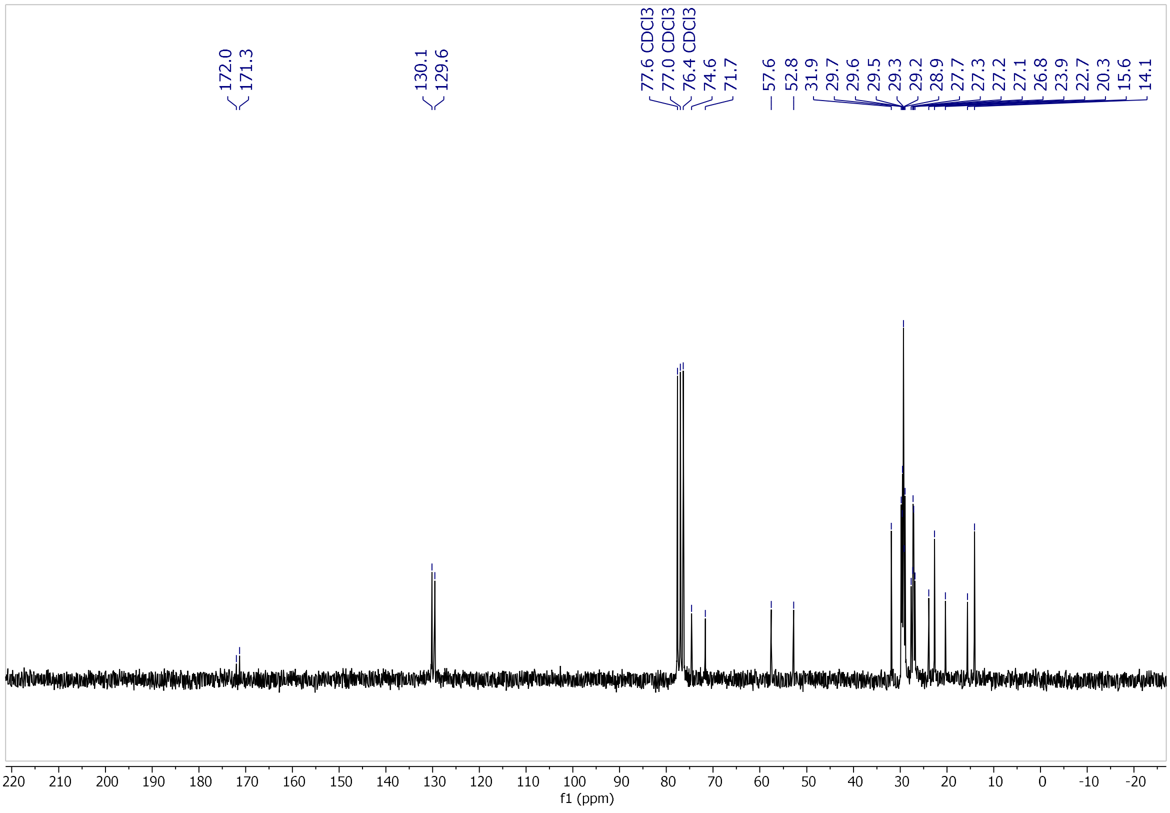

VM013

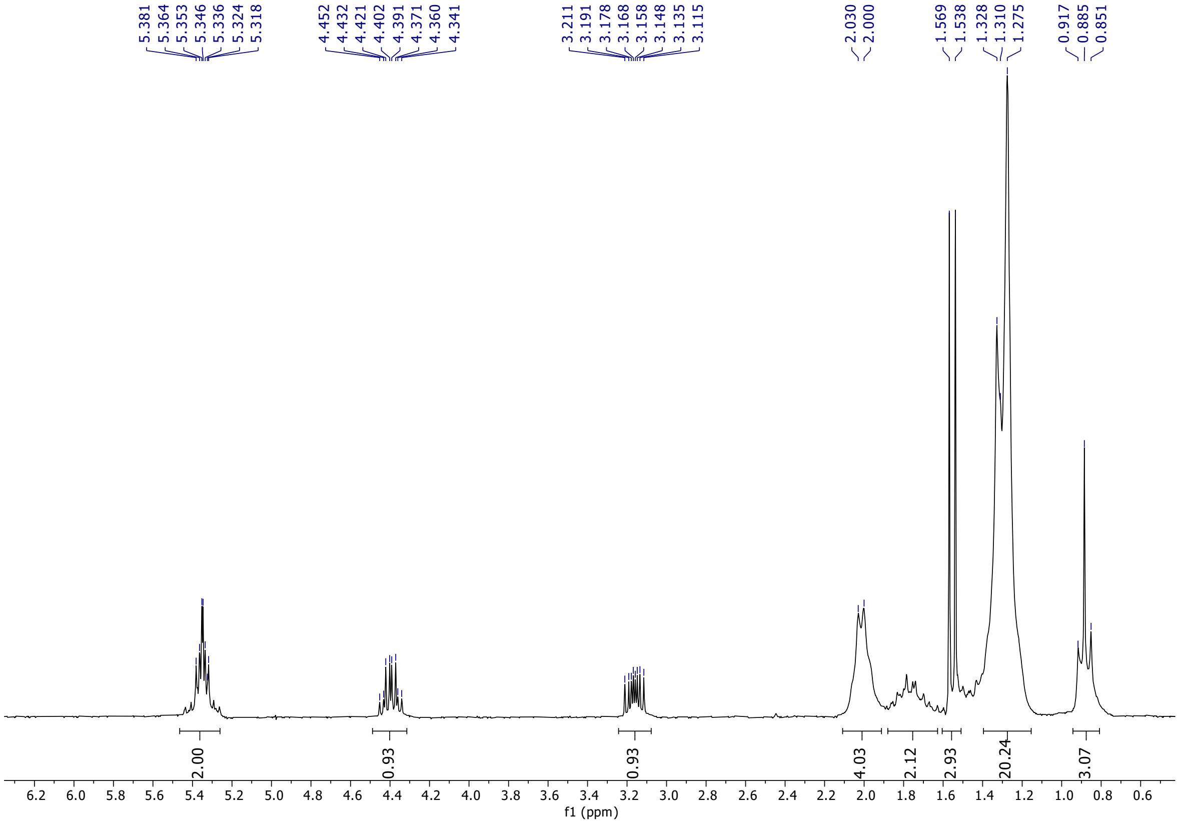
5.381
5.364
5.353
5.346
5.336
5.324
5.318
4.452
4.432
4.421
4.402
4.391
4.371
4.360
4.341
3.211
3.191
3.178
3.168
3.158
3.148
3.135
3.115
2.030
2.000
1.569
1.538
1.328
1.310
1.275
0.917
0.885
0.851
2.00
0.93
0.93
4.03
2.12
2.93
20.24
3.07
f1 (ppm)

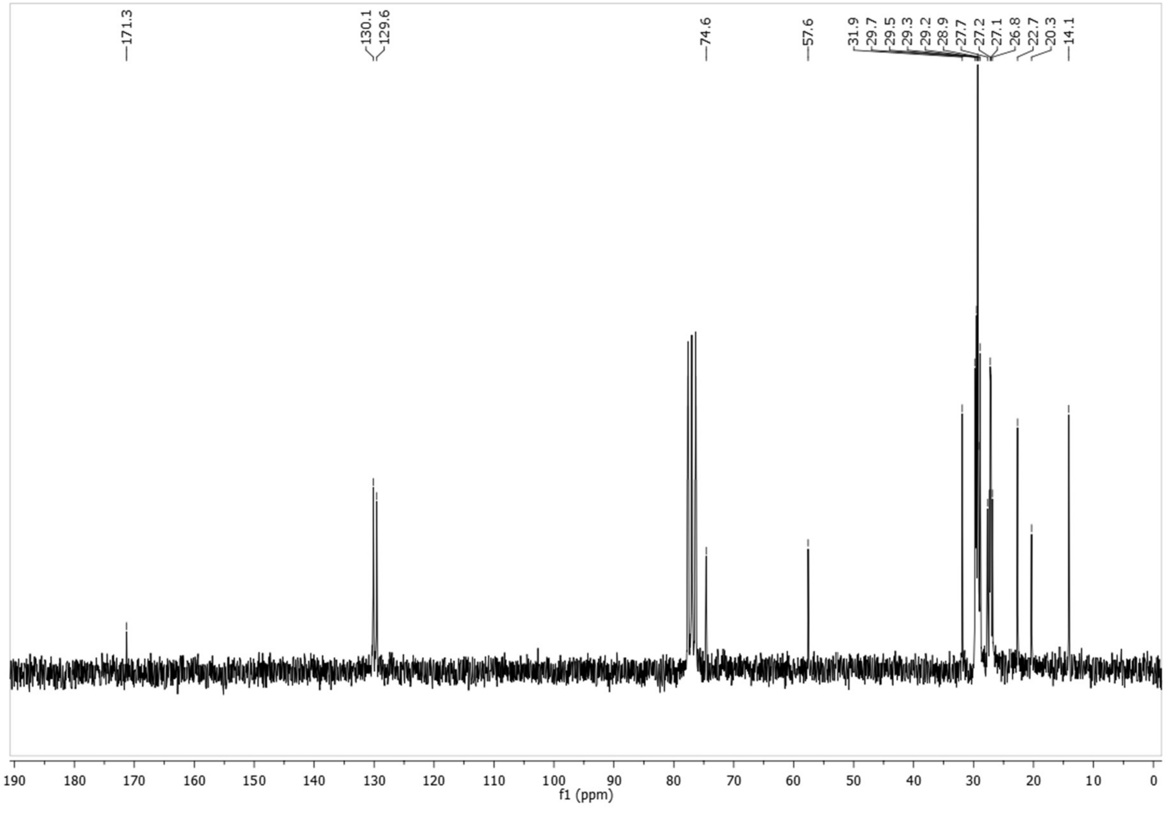
171.3
130.1
129.6
74.6
57.6
31.9
29.7
29.5
29.3
29.2
28.9
27.7
27.2
27.1
26.8
22.7
20.3
14.1
f1 (ppm)

VM020

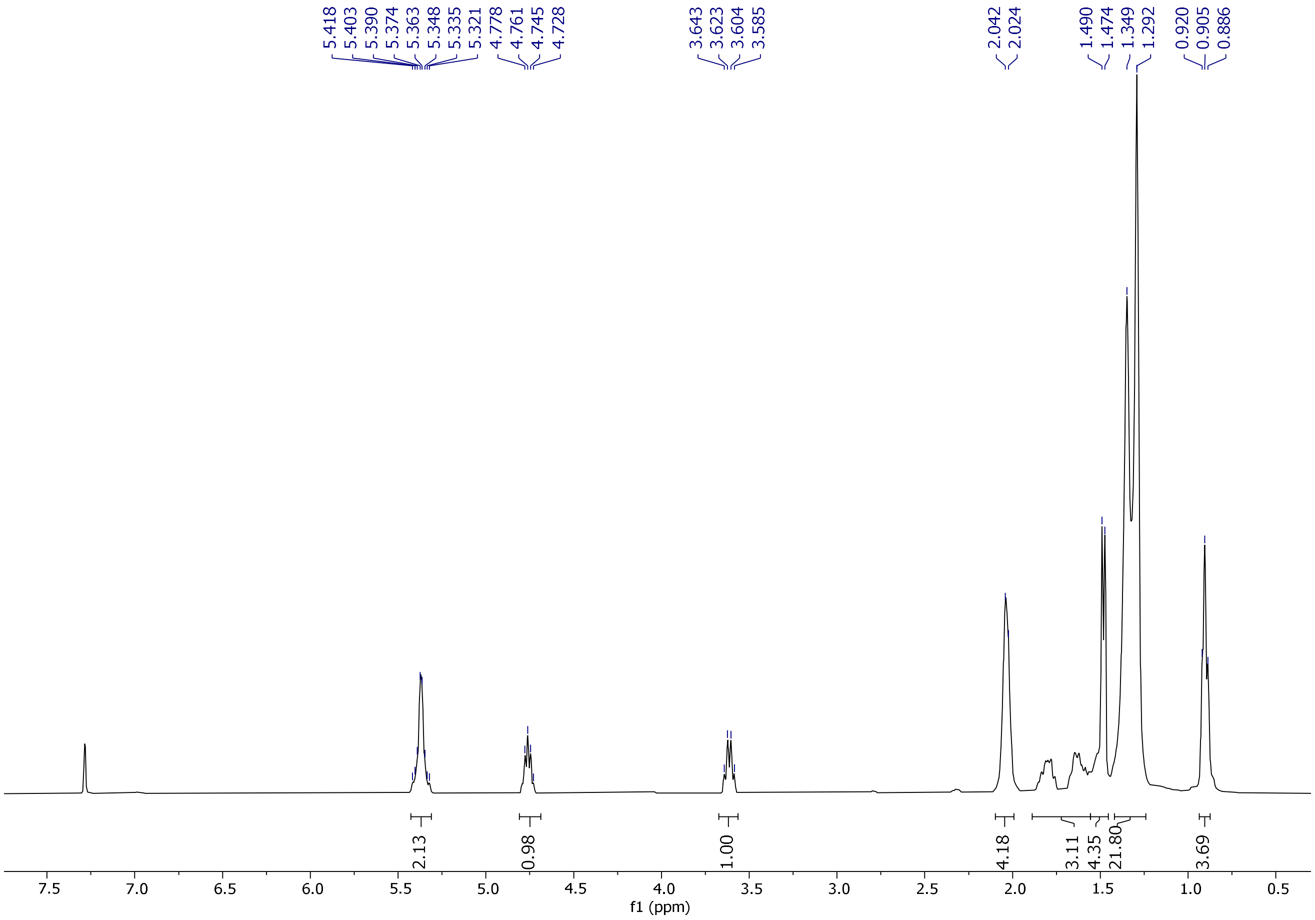
5.418
5.403
5.390
5.374
5.363
5.348
5.335
5.321
4.778
4.761
4.745
4.728
3.643
3.623
3.604
3.585
2.042
2.024
1.490
1.474
1.349
1.292
0.920
0.905
0.886
2.13
0.98
1.00
4.18
3.11
4.35
21.80
3.69
f1 (ppm)

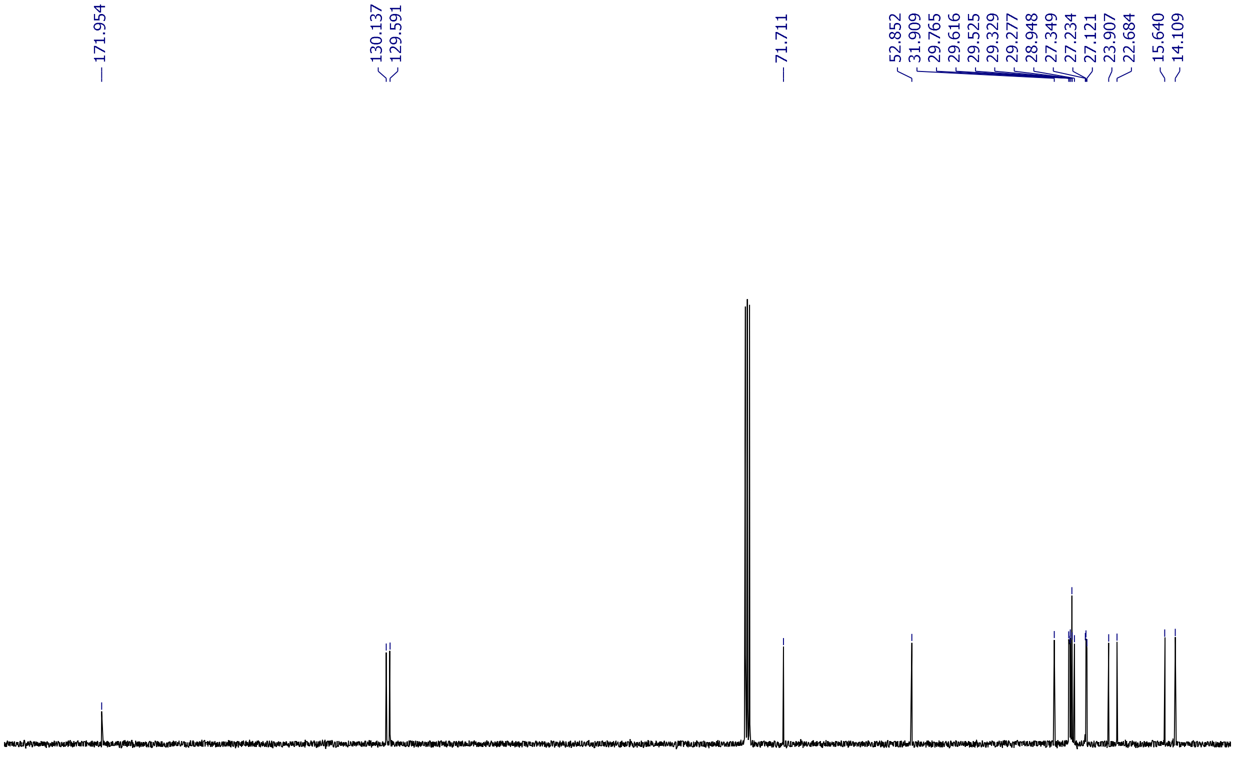
171.954
130.137
129.591
71.711
52.852
31.909
29.765
29.616
29.525
29.329
29.277
28.948
27.349
27.234
27.121
23.907
22.684
15.640
14.109
f1 (ppm)

VM010

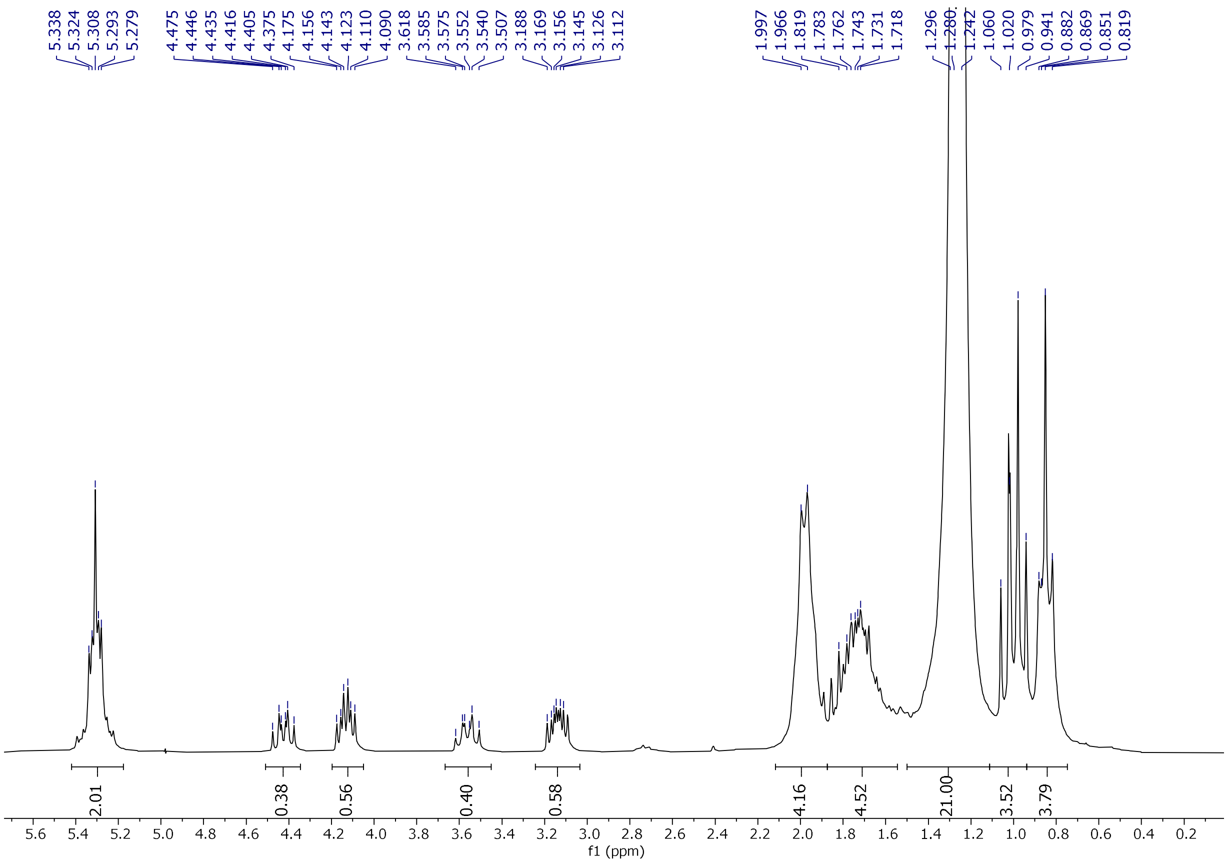

5.338
5.324
5.308
5.293
5.279
4.475
4.446
4.435
4.416
4.405
4.375
4.175
4.156
4.143
4.123
4.110
4.090
3.618
3.585
3.575
3.552
3.540
3.507
3.188
3.169
3.156
3.145
3.126
3.112
1.997
1.966
1.819
1.783
1.762
1.743
1.731
1.718
1.296
1.280
1.242
1.060
1.020
0.979
0.941
0.882
0.869
0.851
0.819
2.01
0.38
0.56
0.40
0.58
4.16
4.52
21.00
3.52
3.79
5.6 5.4 5.2 5.0 4.8 4.6 4.4 4.2 4.0 3.8 3.6 3.4 3.2 3.0 2.8 2.6 2.4 2.2 2.0 1.8 1.6 1.4 1.2 1.0 0.8 0.6 0.4 0.2
f1 (ppm)

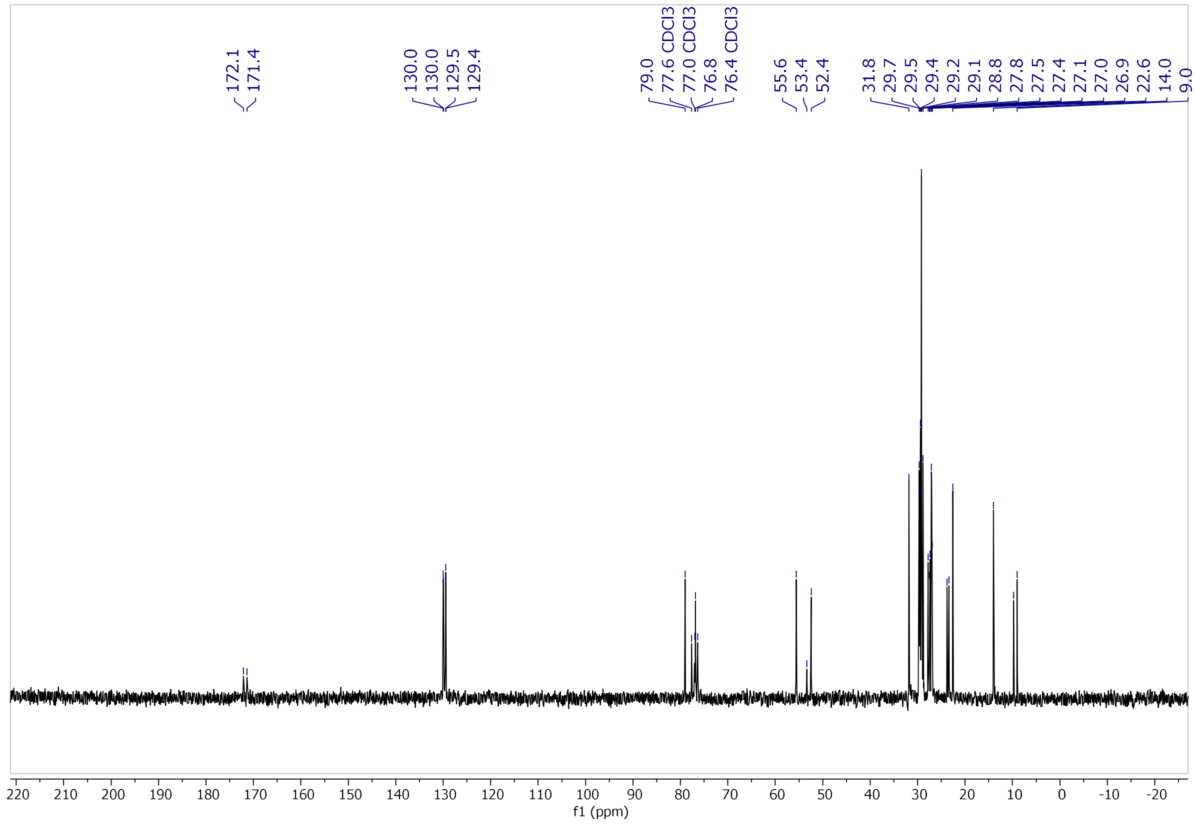

172.1
171.4
130.0
130.0
129.5
129.4
79.0
77.6 CDCl3
77.0 CDCl3
76.8
76.4 CDCl3
55.6
53.4
52.4
31.8
29.7
29.5
29.4
29.2
29.1
28.8
27.8
27.5
27.4
27.1
27.0
26.9
22.6
14.0
9.0
220 210 200 190 180 170 160 150 140 130 120 110 100 90 80 70 60 50 40 30 20 10 0 -10 -20
f1 (ppm)

VM017

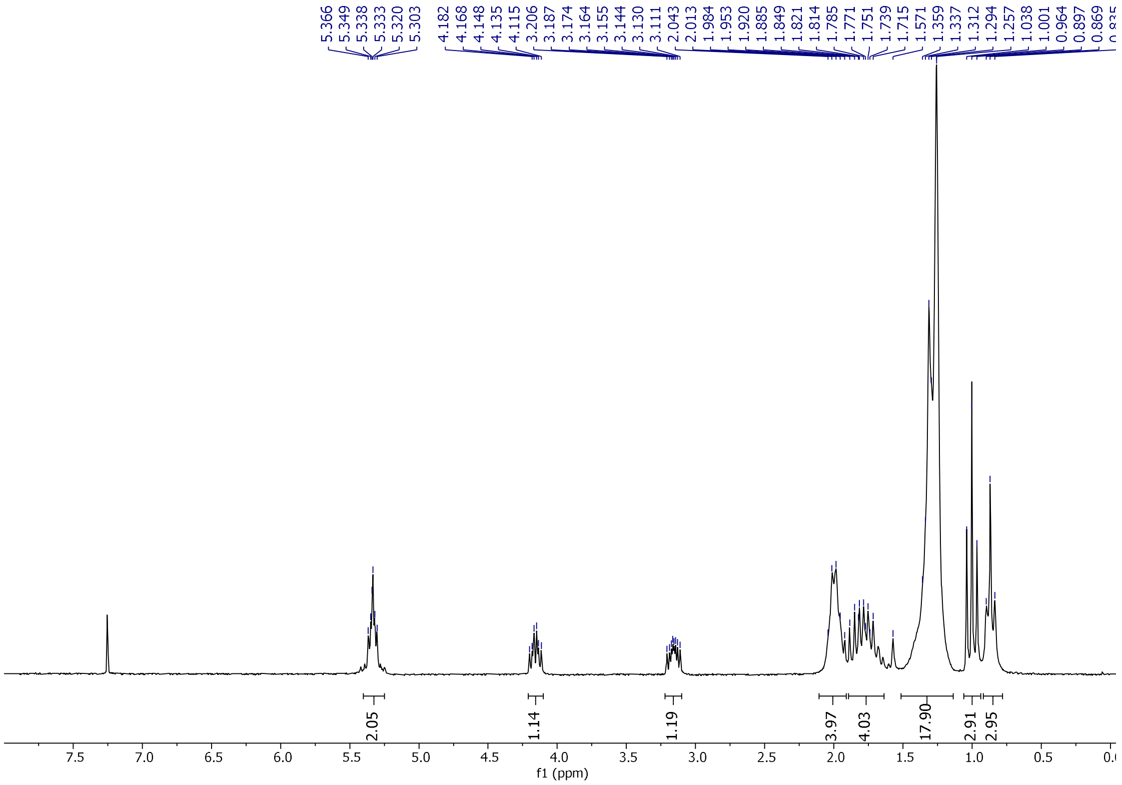
5.366
5.349
5.338
5.333
5.320
5.303
4.182
4.168
4.148
4.135
4.115
3.206
3.187
3.174
3.164
3.155
3.144
3.130
3.111
2.043
2.013
1.984
1.953
1.920
1.885
1.849
1.821
1.814
1.785
1.771
1.751
1.739
1.715
1.571
1.359
1.337
1.312
1.294
1.257
1.038
1.001
0.964
0.897
0.869
2.05
1.14
1.19
3.97
4.03
17.90
2.91
2.95
7.5
7.0
6.5
6.0
5.5
5.0
4.5
4.0
3.5
3.0
2.5
2.0
1.5
1.0
0.5
f1 (ppm)

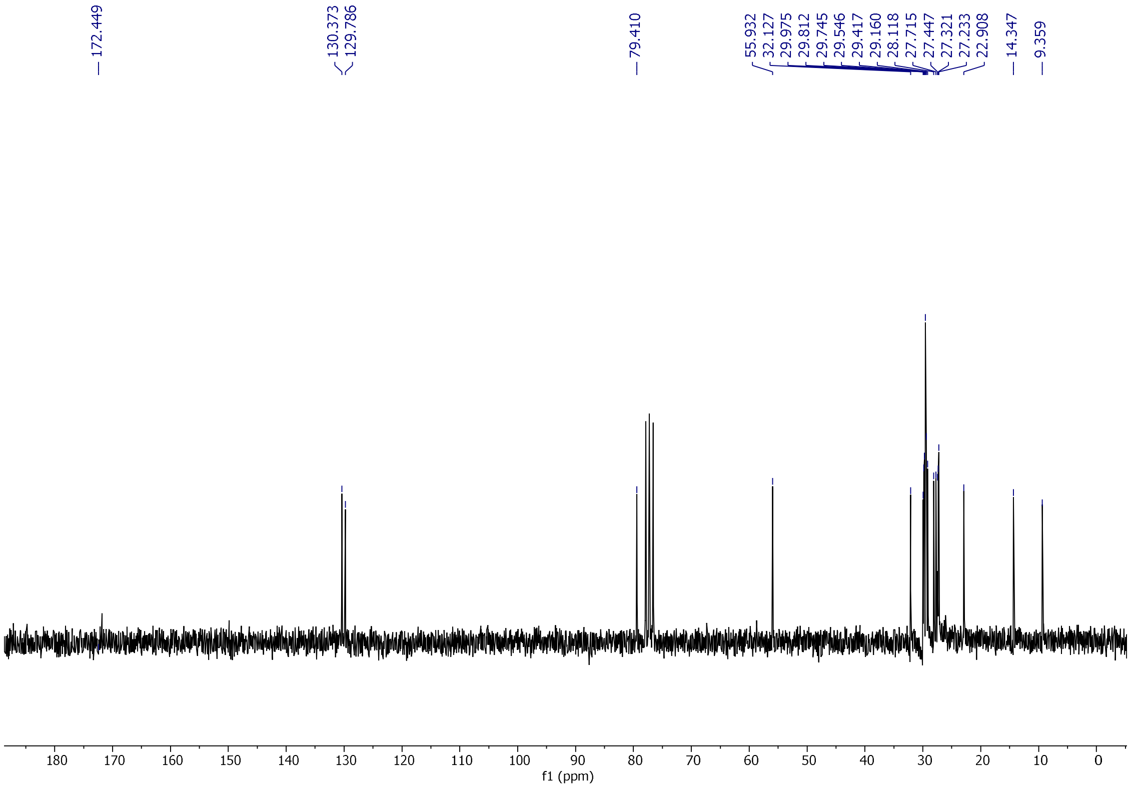
172.449
130.373
129.786
79.410
55.932
32.127
29.975
29.812
29.745
29.546
29.417
29.160
28.118
27.715
27.447
27.321
27.233
22.908
14.347
9.359
180
170
160
150
140
130
120
110
100
90
80
70
60
50
40
30
20
10
0
f1 (ppm)

VM018

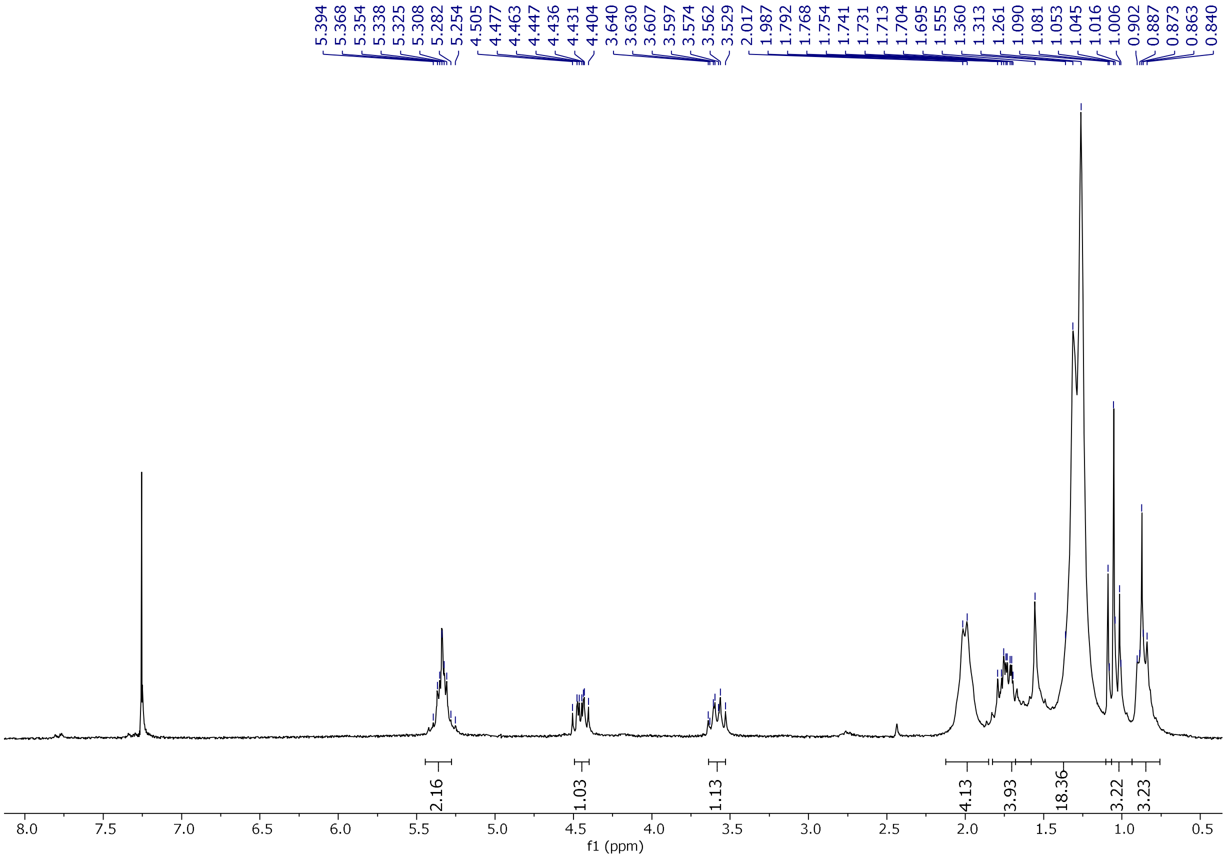
5.394
5.368
5.354
5.338
5.325
5.308
5.282
5.254
4.505
4.477
4.463
4.447
4.436
4.431
4.404
3.640
3.630
3.607
3.597
3.574
3.562
3.529
2.017
1.987
1.792
1.768
1.754
1.741
1.731
1.713
1.704
1.695
1.555
1.360
1.313
1.261
1.090
1.081
1.053
1.045
1.016
1.006
0.902
0.887
0.873
0.863
0.840
2.16
1.03
1.13
4.13
3.93
18.36
3.22
3.23
8.0
7.5
7.0
6.5
6.0
5.5
5.0
4.5
4.0
3.5
3.0
2.5
2.0
1.5
1.0
0.5
f1 (ppm)

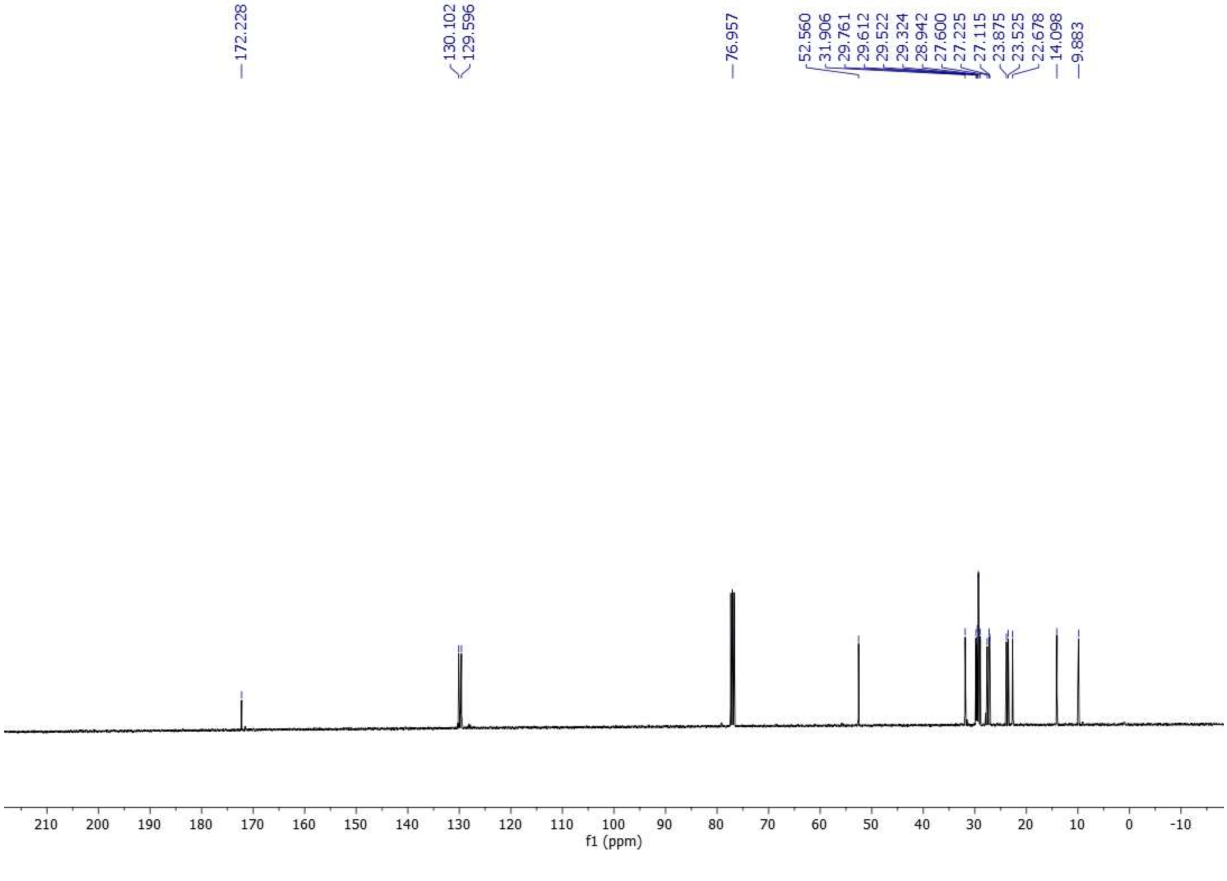
172.228
130.102
129.596
76.957
52.560
31.906
29.761
29.612
29.522
29.324
28.942
27.600
27.225
27.115
23.875
23.525
22.678
14.098
9.883
210
200
190
180
170
160
150
140
130
120
110
100
90
80
70
60
50
40
30
20
10
0
-10
f1 (ppm)

VM019

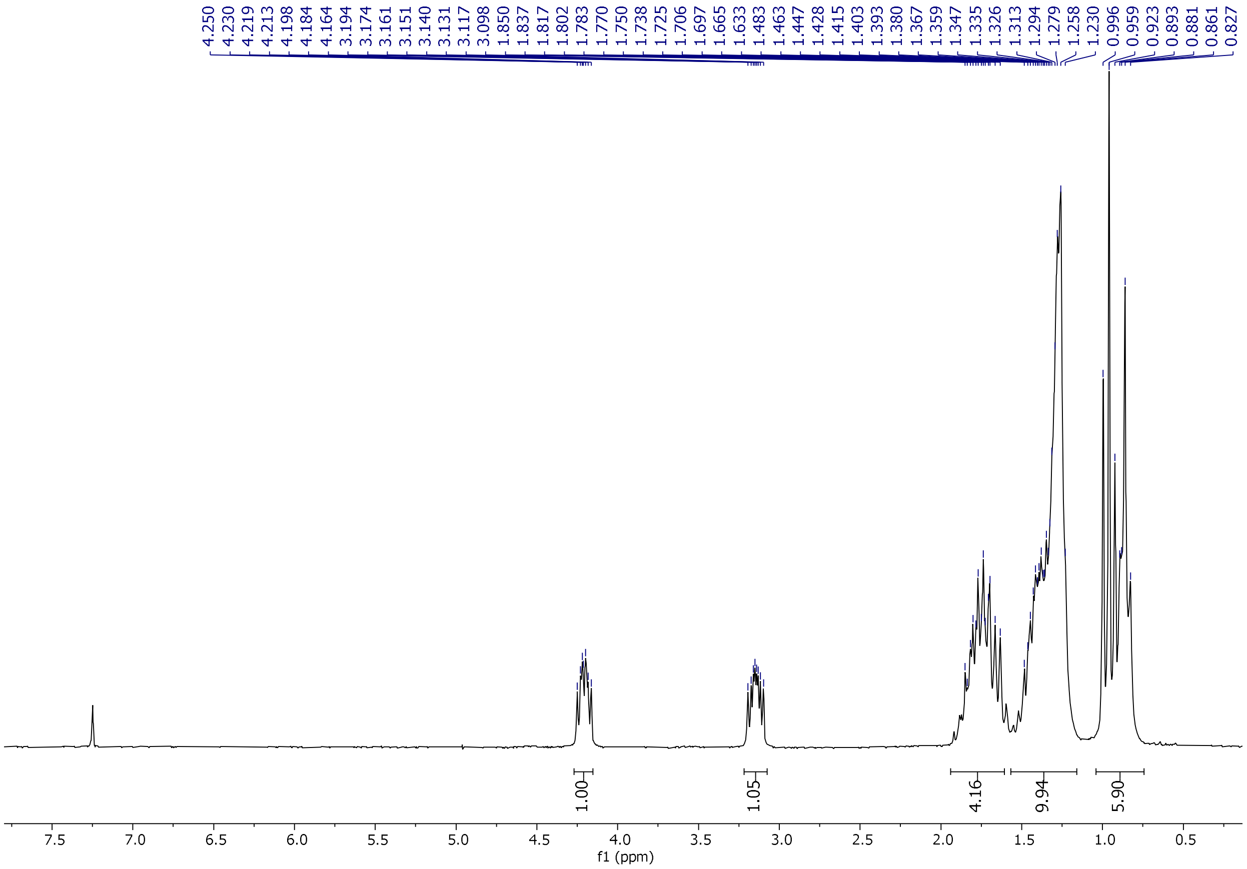
4.250
4.230
4.219
4.213
4.198
4.184
4.164
3.194
3.174
3.161
3.151
3.140
3.131
3.117
3.098
1.850
1.837
1.817
1.802
1.783
1.770
1.750
1.738
1.725
1.706
1.697
1.665
1.633
1.483
1.463
1.447
1.428
1.415
1.403
1.393
1.380
1.367
1.359
1.347
1.335
1.326
1.313
1.294
1.279
1.258
1.230
0.996
0.959
0.923
0.893
0.881
0.861
0.827
1.00
1.05
4.16
9.94
5.90
f1 (ppm)

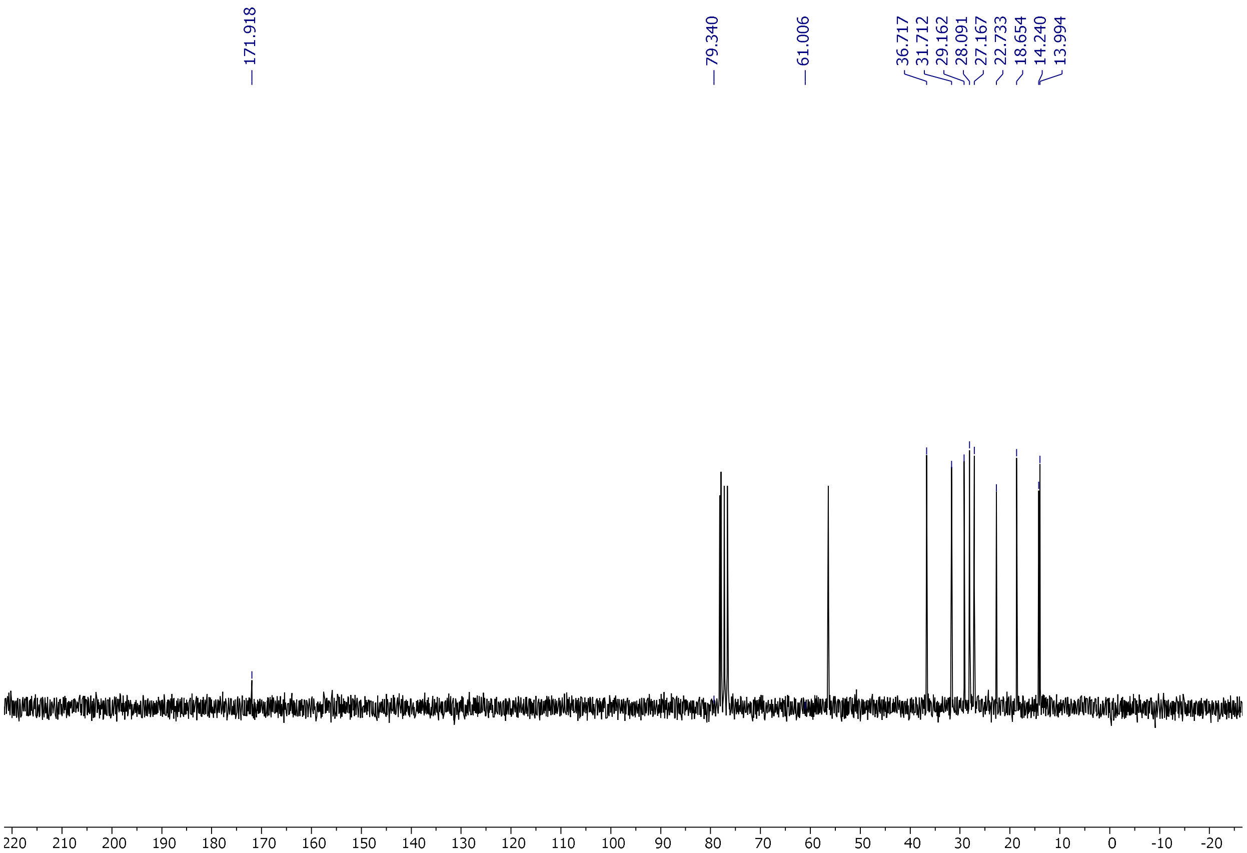
171.918
79.340
61.006
36.717
31.712
29.162
28.091
27.167
22.733
18.654
14.240
13.994
f1 (ppm)

VM011

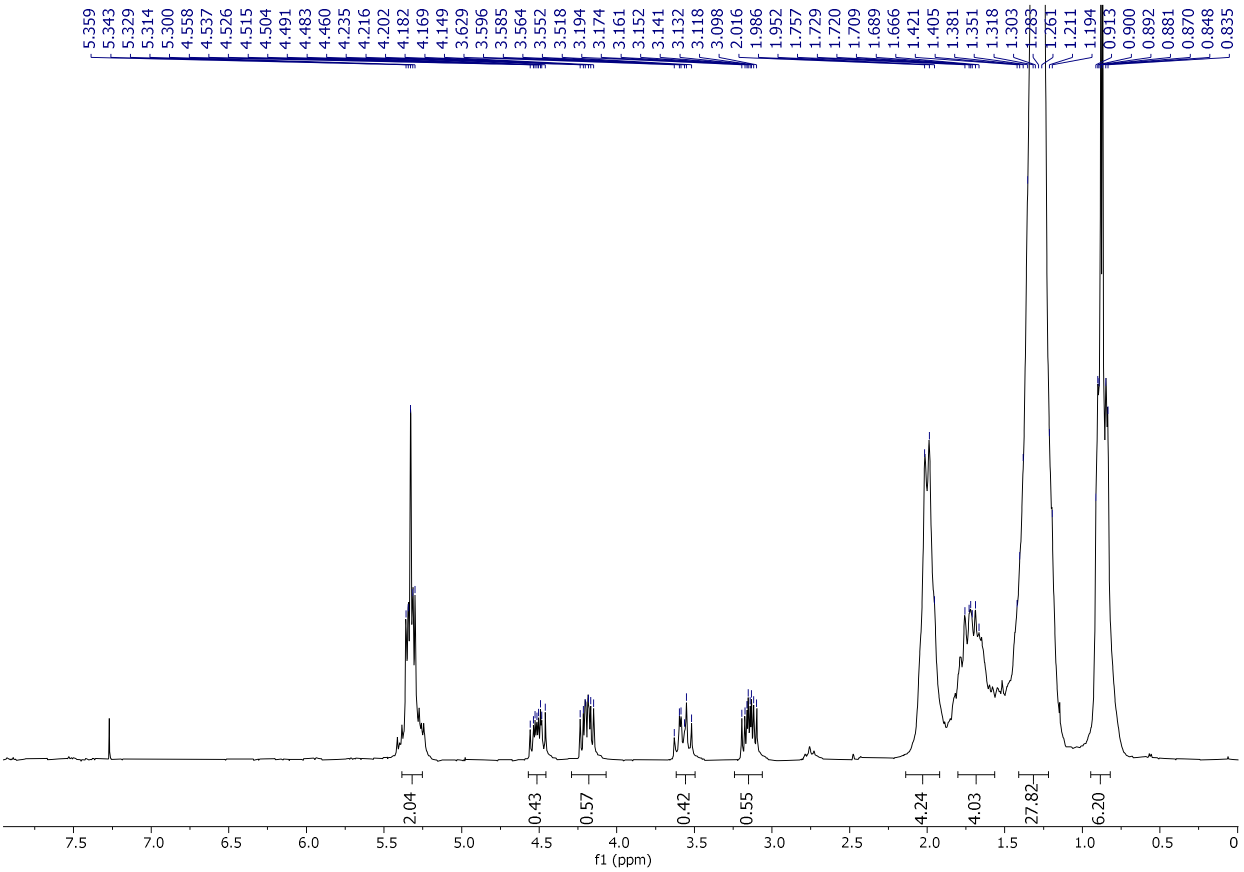

5.359
5.343
5.329
5.314
5.300
4.558
4.537
4.526
4.515
4.504
4.491
4.483
4.460
4.235
4.216
4.202
4.182
4.169
4.149
3.629
3.596
3.585
3.564
3.552
3.518
3.194
3.174
3.161
3.152
3.141
3.132
3.118
3.098
2.016
1.986
1.952
1.757
1.729
1.720
1.709
1.689
1.666
1.421
1.405
1.381
1.351
1.318
1.303
1.283
1.261
1.211
1.194
0.913
0.900
0.892
0.881
0.870
0.848
0.835
2.04
0.43
0.57
0.42
0.55
4.24
4.03
27.82
6.20
f1 (ppm)

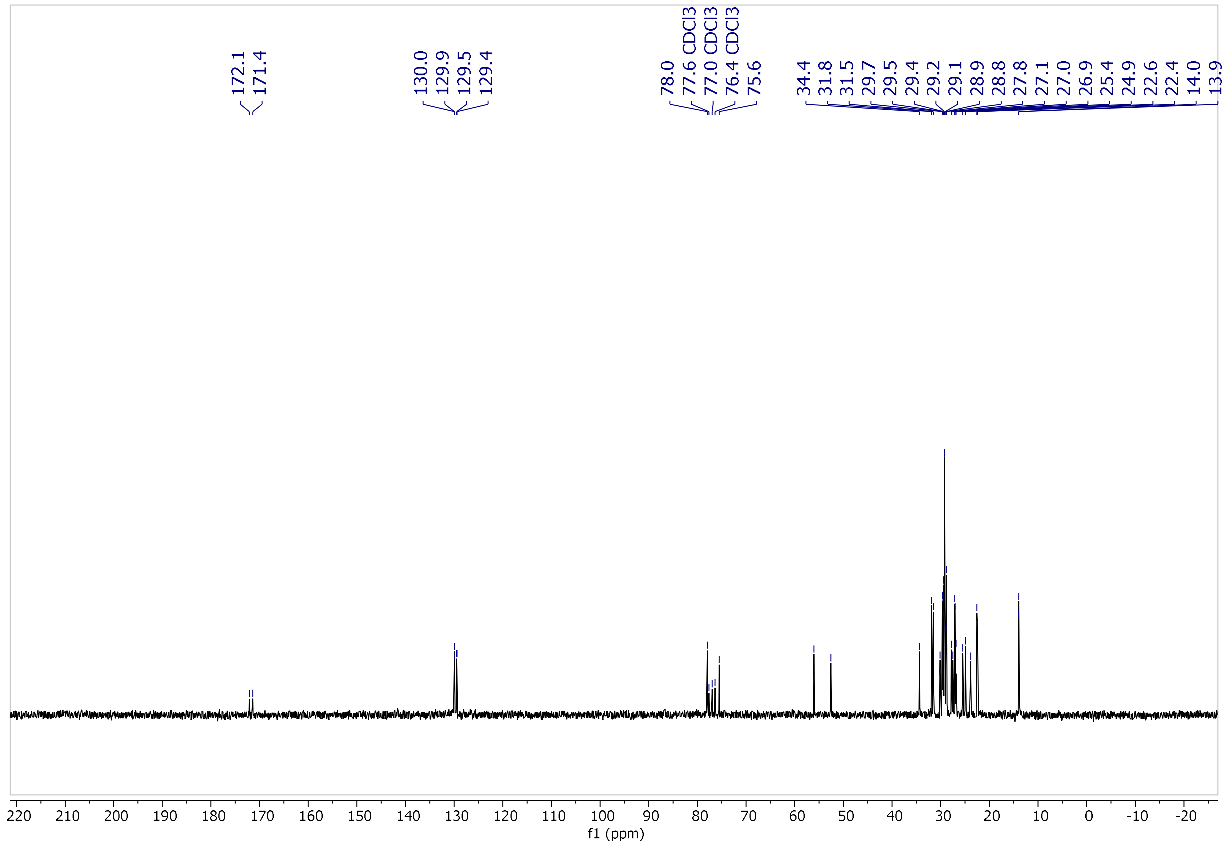

172.1
171.4
130.0
129.9
129.5
129.4
78.0
77.6 CDCl3
77.0 CDCl3
76.4 CDCl3
75.6
34.4
31.8
31.5
29.7
29.5
29.4
29.2
29.1
28.9
28.8
27.8
27.1
27.0
26.9
25.4
24.9
22.6
22.4
14.0
13.9
f1 (ppm)

VM022

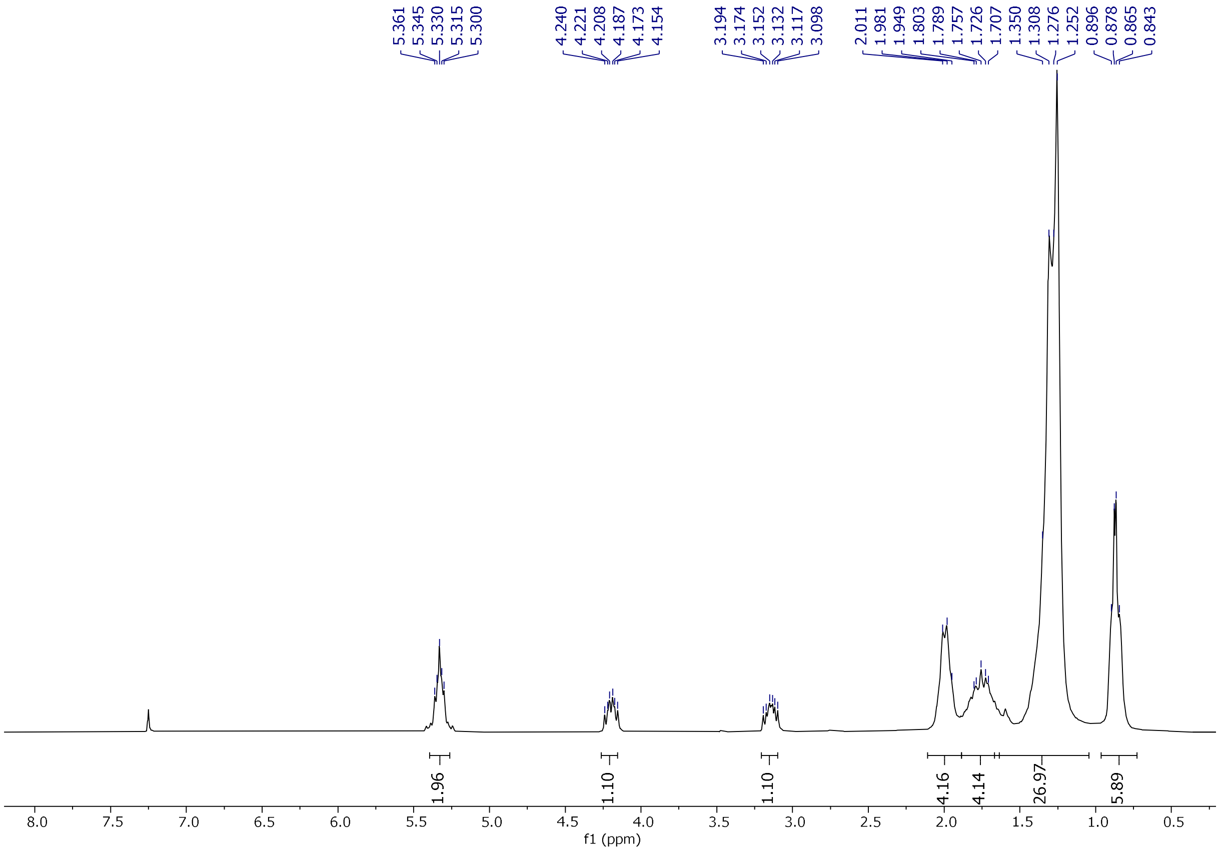
5.361
5.345
5.330
5.315
5.300
4.240
4.221
4.208
4.187
4.173
4.154
3.194
3.174
3.152
3.132
3.117
3.098
2.011
1.981
1.949
1.803
1.789
1.757
1.726
1.707
1.350
1.308
1.276
1.252
0.896
0.878
0.865
0.843
1.96
1.10
1.10
4.16
4.14
26.97
5.89
f1 (ppm)

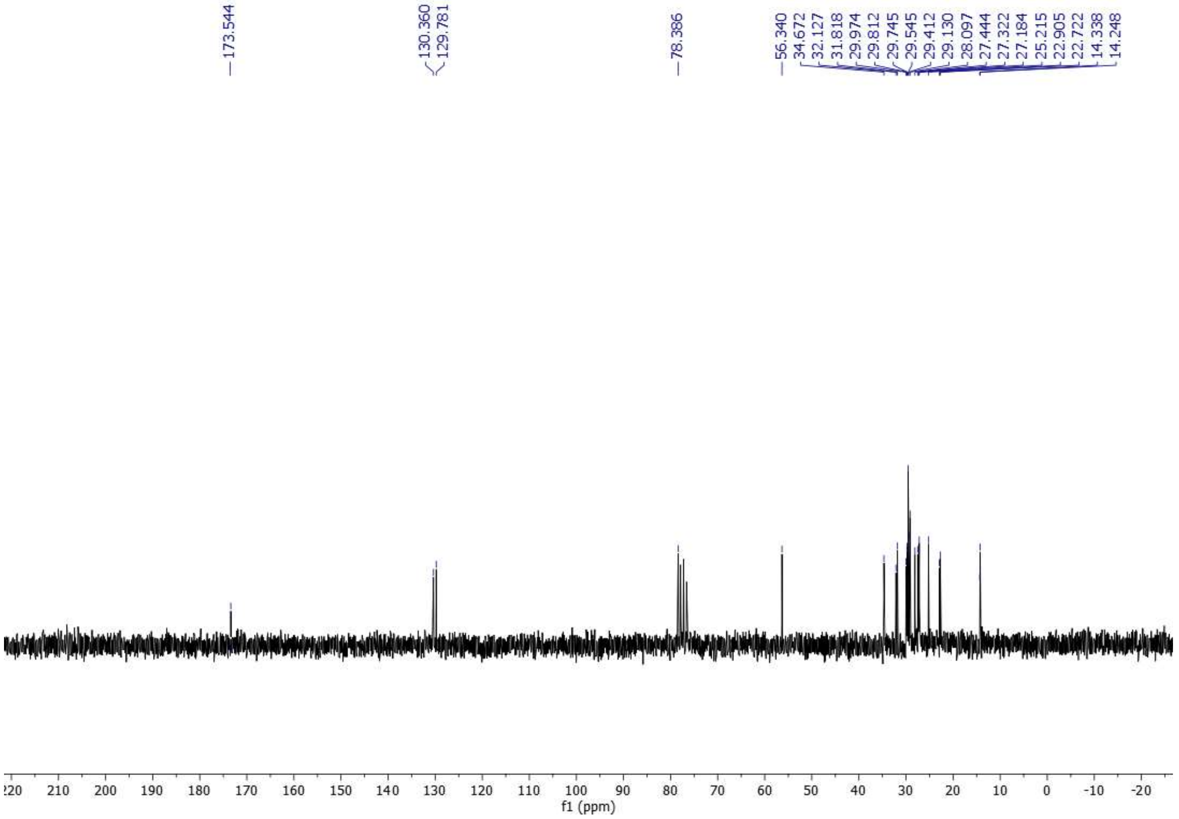
173.544
130.360
129.781
78.386
56.340
34.672
32.127
31.818
29.974
29.812
29.745
29.545
29.412
29.130
28.097
27.444
27.322
27.184
25.215
22.905
22.722
14.338
14.248
f1 (ppm)

VM024

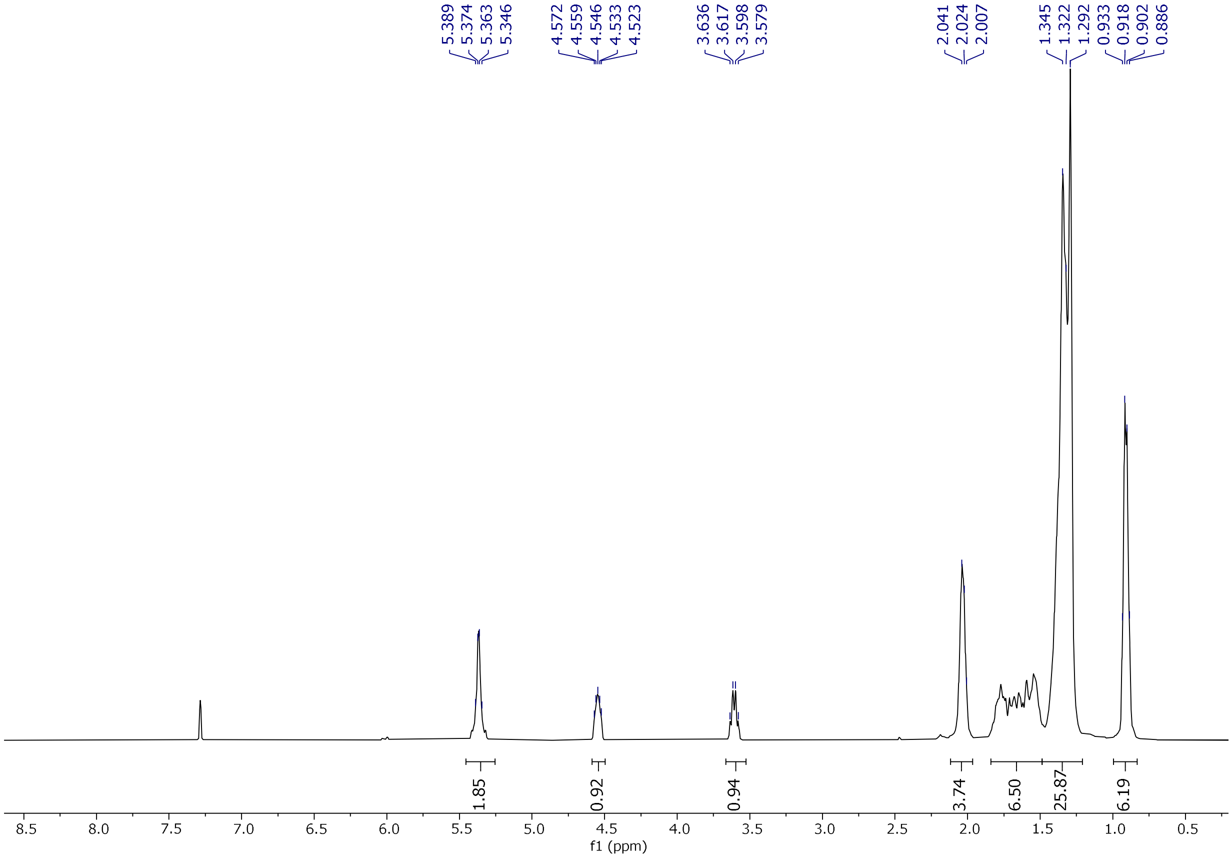
5.389
5.374
5.363
5.346
4.572
4.559
4.546
4.533
4.523
3.636
3.617
3.598
3.579
2.041
2.024
2.007
1.345
1.322
1.292
0.933
0.918
0.902
0.886
1.85
0.92
0.94
3.74
6.50
25.87
6.19
8.5 8.0 7.5 7.0 6.5 6.0 5.5 5.0 4.5 4.0 3.5 3.0 2.5 2.0 1.5 1.0 0.5
f1 (ppm)

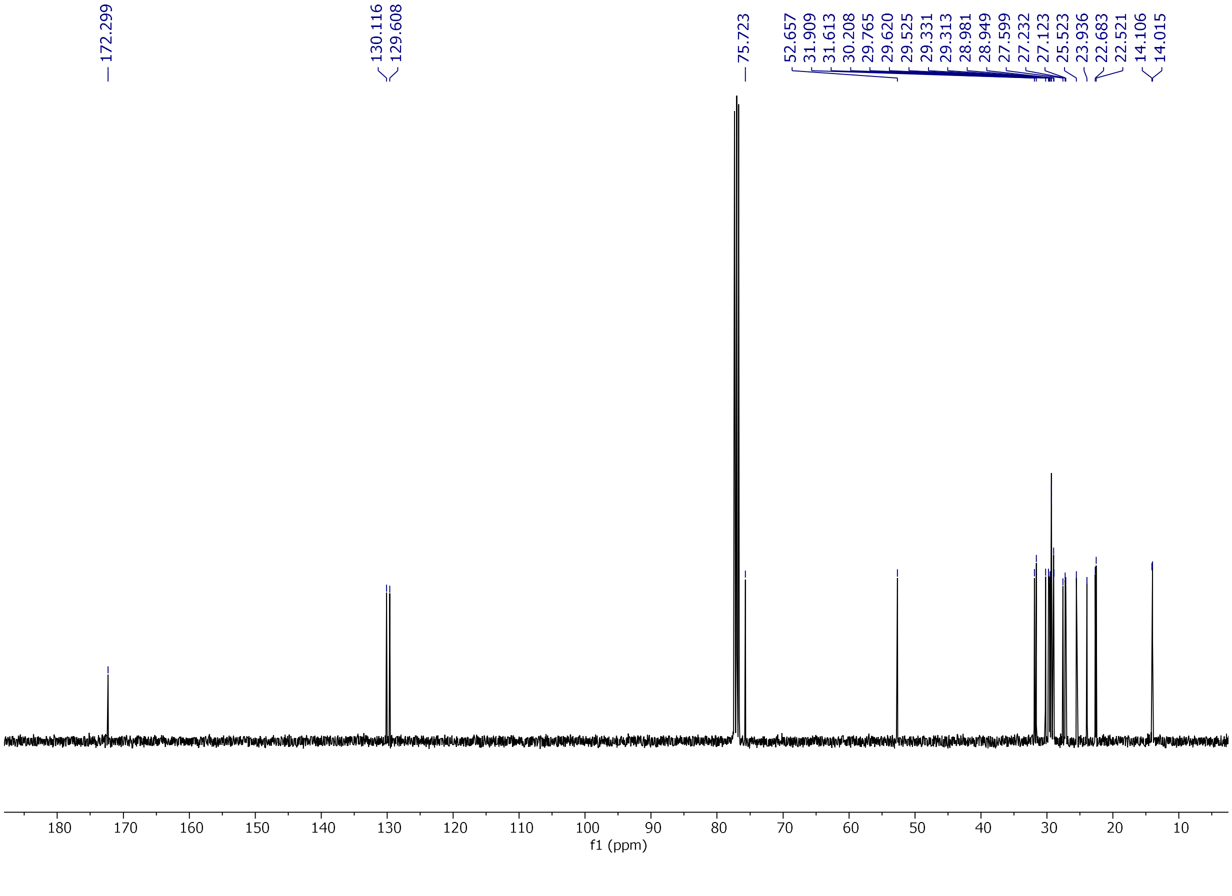
172.299
130.116
129.608
75.723
52.657
31.909
31.613
30.208
29.765
29.620
29.525
29.331
29.313
28.981
28.949
27.599
27.232
27.123
25.523
23.936
22.683
22.521
14.106
14.015
180 170 160 150 140 130 120 110 100 90 80 70 60 50 40 30 20 10
f1 (ppm)

VM012

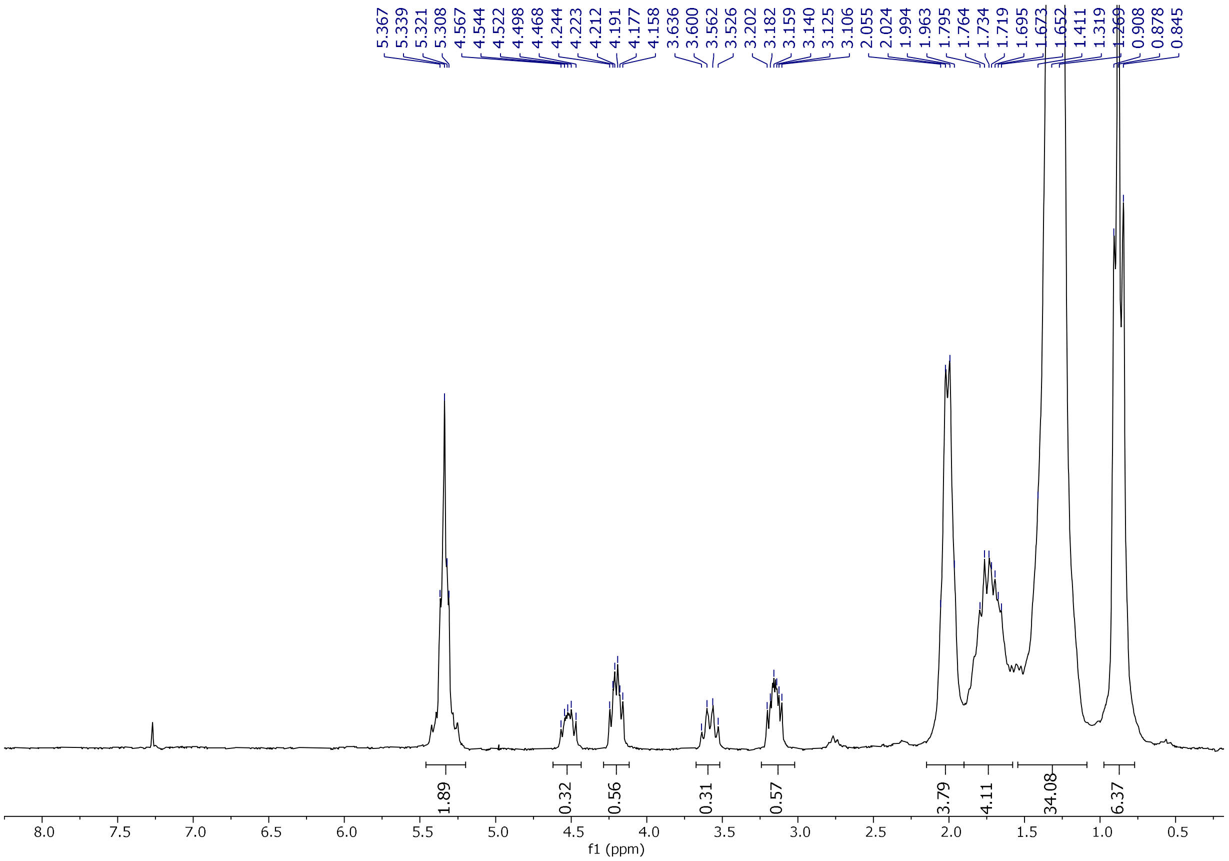
5.367
5.339
5.321
5.308
4.567
4.544
4.522
4.498
4.468
4.244
4.223
4.212
4.191
4.177
4.158
3.636
3.600
3.562
3.526
3.202
3.182
3.159
3.140
3.125
3.106
2.055
2.024
1.994
1.963
1.795
1.764
1.734
1.719
1.695
1.673
1.652
1.411
1.319
1.269
0.908
0.878
0.845
1.89
0.32
0.56
0.31
0.57
3.79
4.11
34.08
6.37
8.0
7.5
7.0
6.5
6.0
5.5
5.0
4.5
4.0
3.5
3.0
2.5
2.0
1.5
1.0
0.5
f1 (ppm)

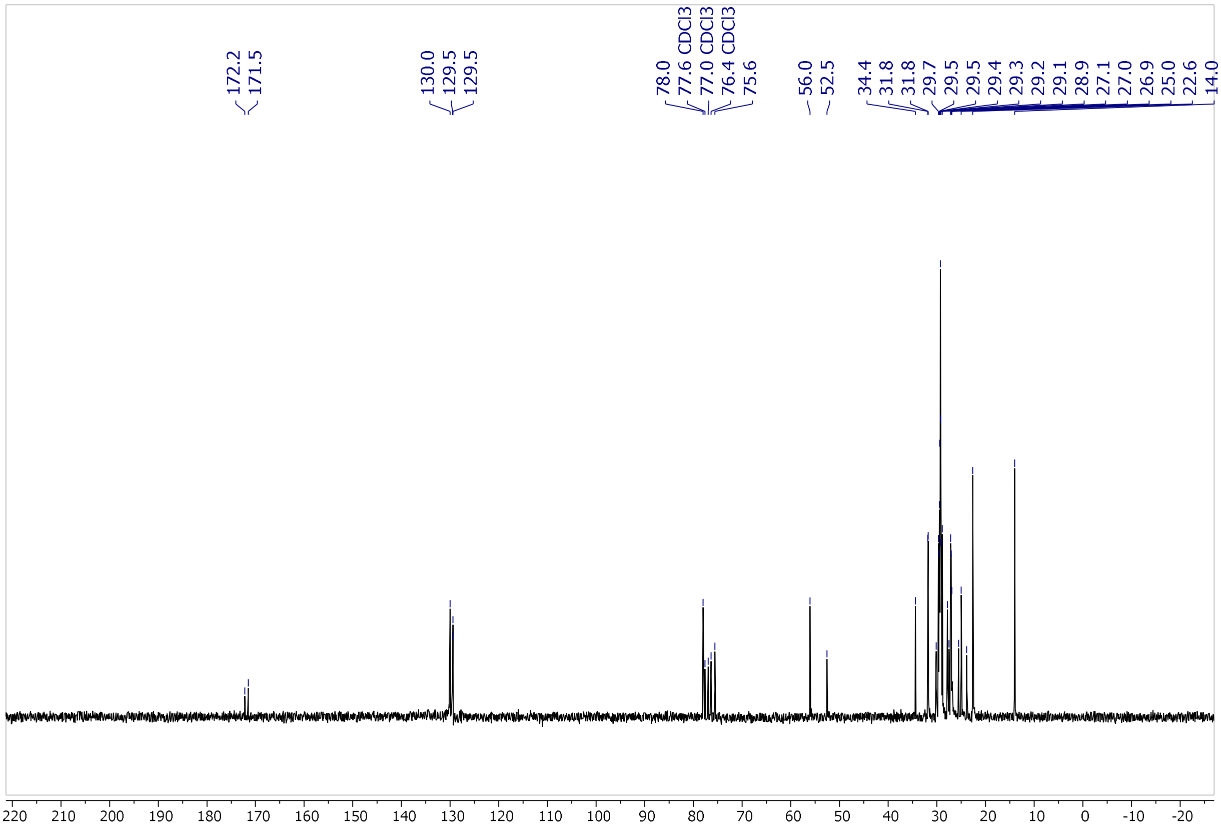
172.2
171.5
130.0
129.5
129.5
78.0
77.6 CDCl3
77.0 CDCl3
76.4 CDCl3
75.6
56.0
52.5
34.4
31.8
31.8
29.7
29.5
29.5
29.4
29.3
29.2
29.1
28.9
27.1
27.0
26.9
25.0
22.6
14.0
220
210
200
190
180
170
160
150
140
130
120
110
100
90
80
70
60
50
40
30
20
10
0
-10
-20
f1 (ppm)

VM021

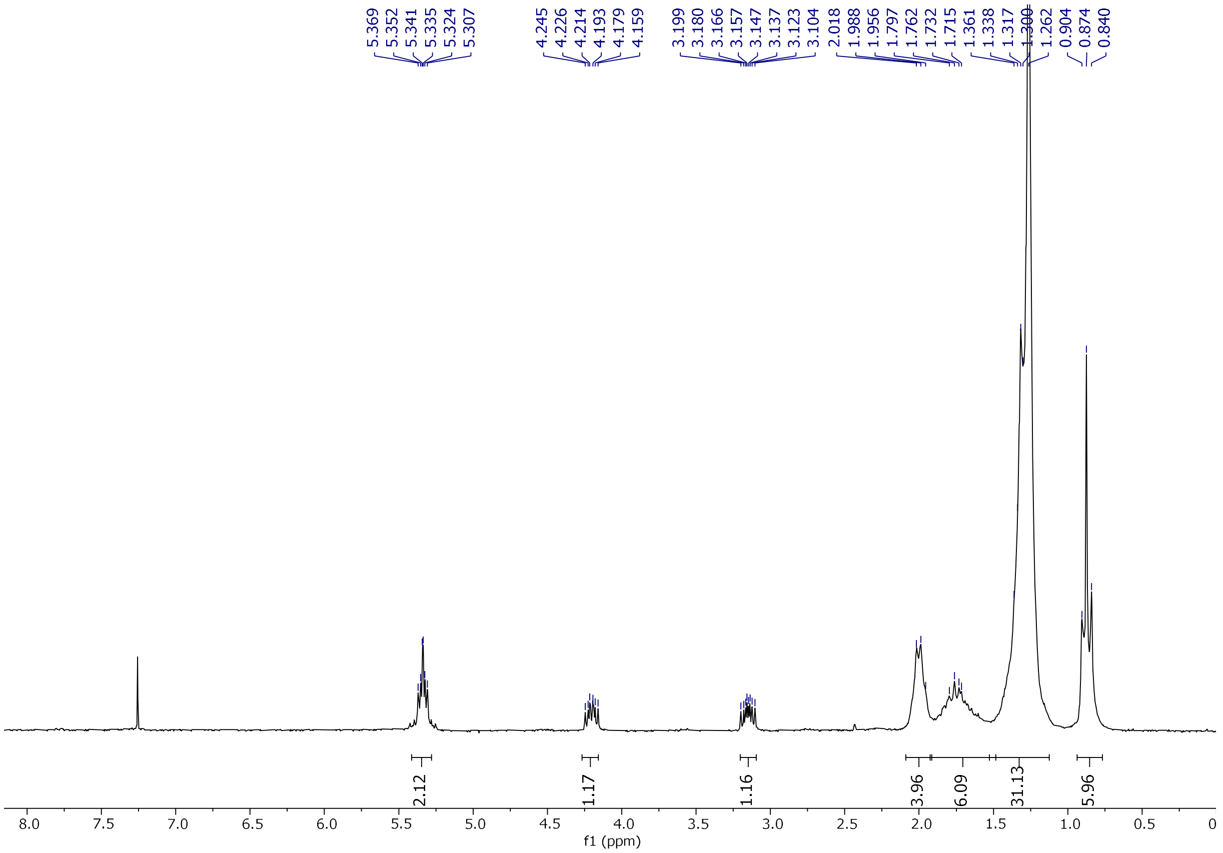
5.369
5.352
5.341
5.335
5.324
5.307
4.245
4.226
4.214
4.193
4.179
4.159
3.199
3.180
3.166
3.157
3.147
3.137
3.123
3.104
2.018
1.988
1.956
1.797
1.762
1.732
1.715
1.361
1.338
1.317
1.300
1.262
0.904
0.874
0.840
2.12
1.17
1.16
3.96
6.09
31.13
5.96
8.0 7.5 7.0 6.5 6.0 5.5 5.0 4.5 4.0 3.5 3.0 2.5 2.0 1.5 1.0 0.5 0
f1 (ppm)

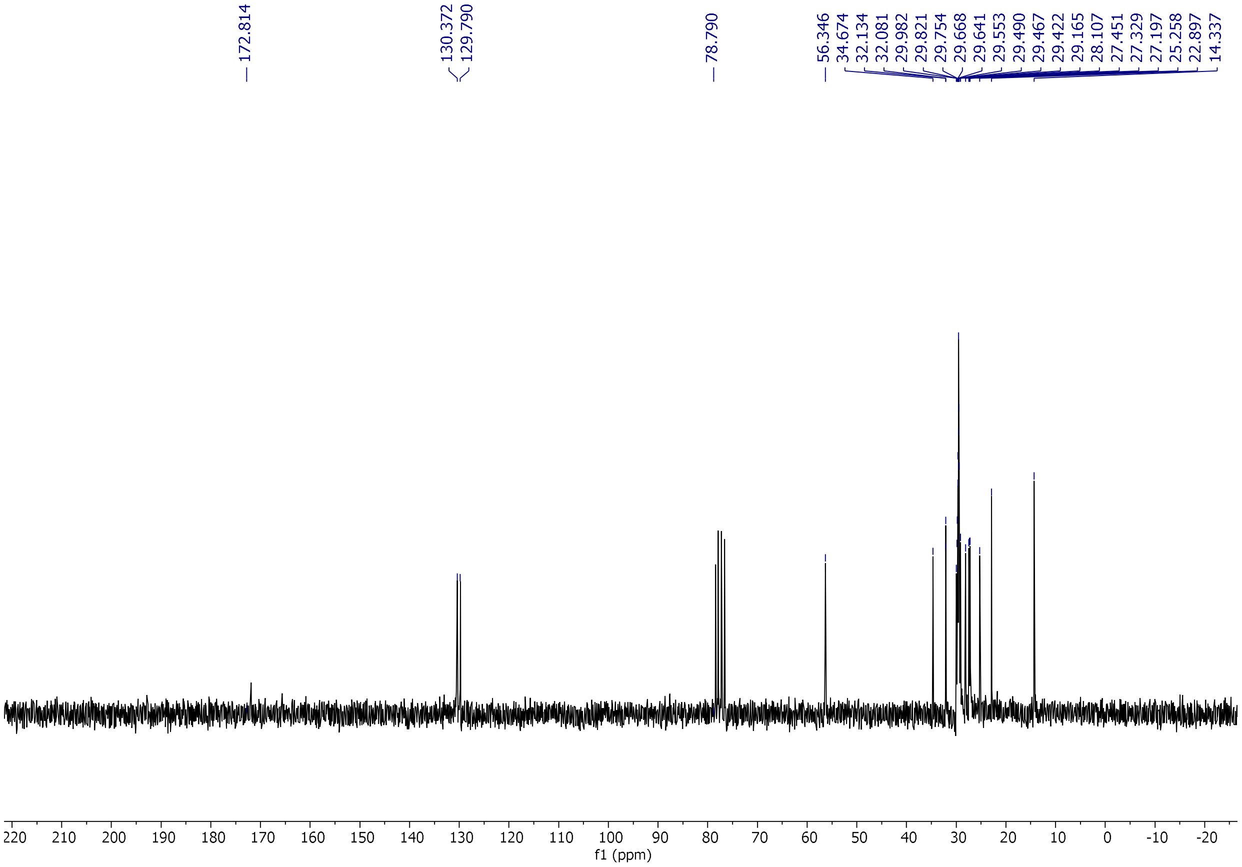
172.814
130.372
129.790
78.790
56.346
34.674
32.134
32.081
29.982
29.821
29.754
29.668
29.641
29.553
29.490
29.467
29.422
29.165
28.107
27.451
27.329
27.197
25.258
22.897
14.337
220 210 200 190 180 170 160 150 140 130 120 110 100 90 80 70 60 50 40 30 20 10 0 -10 -20
f1 (ppm)

VM023

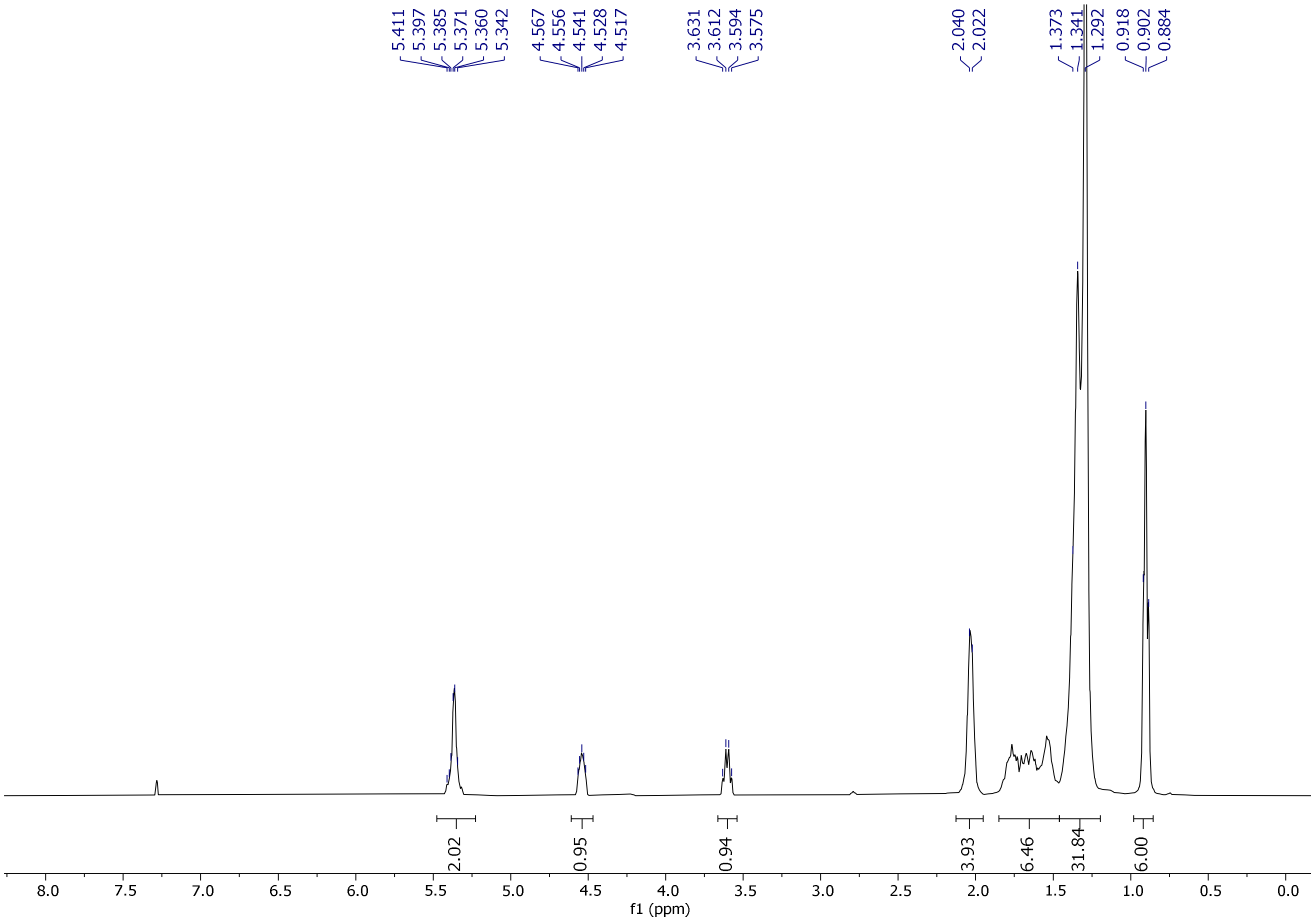
5.411
5.397
5.385
5.371
5.360
5.342
4.567
4.556
4.541
4.528
4.517
3.631
3.612
3.594
3.575
2.040
2.022
1.373
1.341
1.292
0.918
0.902
0.884
2.02
0.95
0.94
3.93
6.46
31.84
6.00
f1 (ppm)

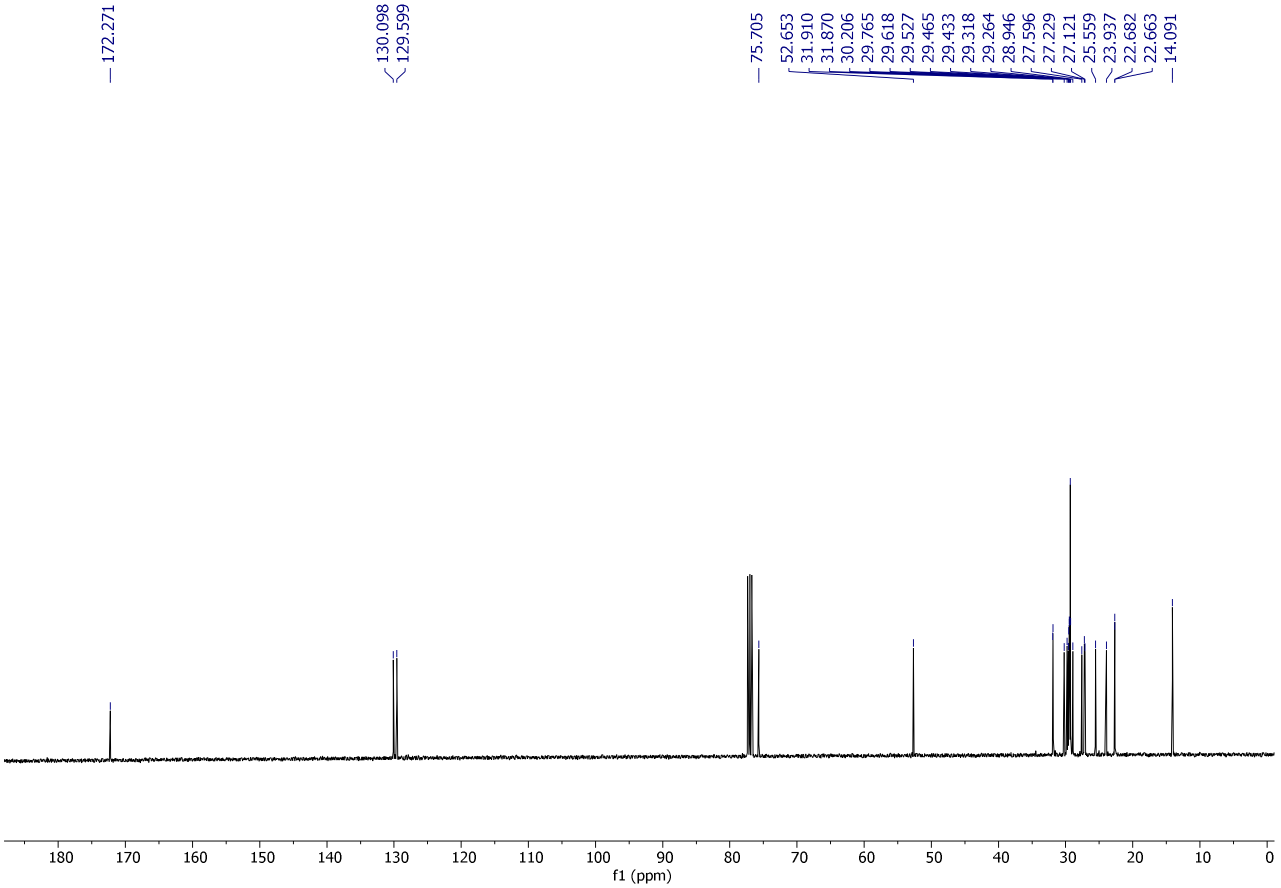
172.271
130.098
129.599
75.705
52.653
31.910
31.870
30.206
29.765
29.618
29.527
29.465
29.433
29.318
29.264
28.946
27.596
27.229
27.121
25.559
23.937
22.682
22.663
14.091
f1 (ppm)

VM025

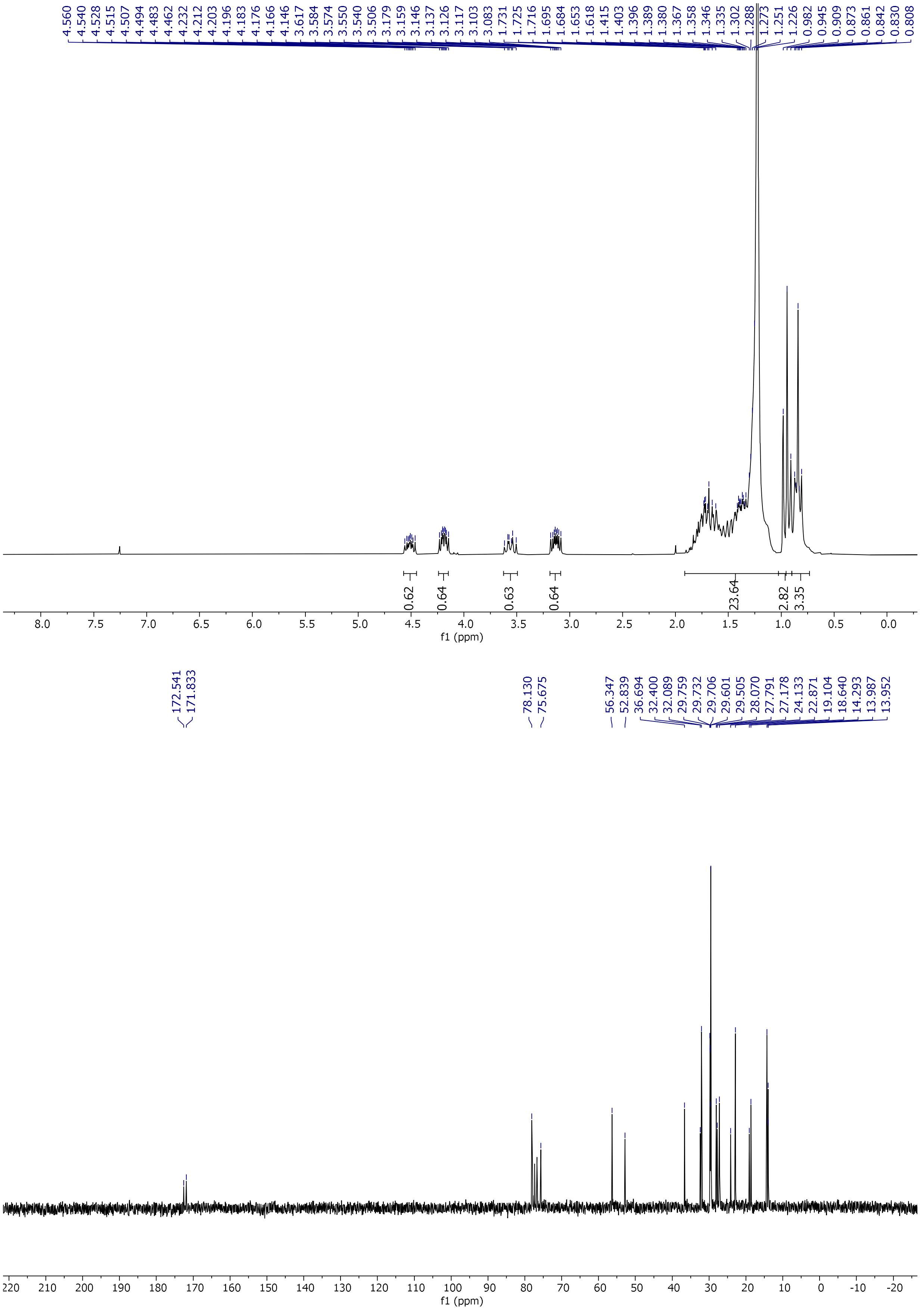
4.560
4.540
4.528
4.515
4.507
4.494
4.483
4.462
4.232
4.212
4.203
4.196
4.183
4.176
4.166
4.146
3.617
3.584
3.574
3.550
3.540
3.506
3.179
3.159
3.146
3.137
3.126
3.117
3.103
3.083
1.731
1.725
1.716
1.695
1.684
1.653
1.618
1.415
1.403
1.396
1.389
1.380
1.367
1.358
1.346
1.335
1.302
1.288
1.273
1.251
1.226
0.982
0.945
0.909
0.873
0.861
0.842
0.830
0.808
0.62
0.64
0.63
0.64
23.64
2.82
3.35
f1 (ppm)
172.541
171.833
78.130
75.675
56.347
52.839
36.694
32.400
32.089
29.759
29.732
29.706
29.601
29.505
28.070
27.791
27.178
24.133
22.871
19.104
18.640
14.293
13.987
13.952
f1 (ppm)

VM026

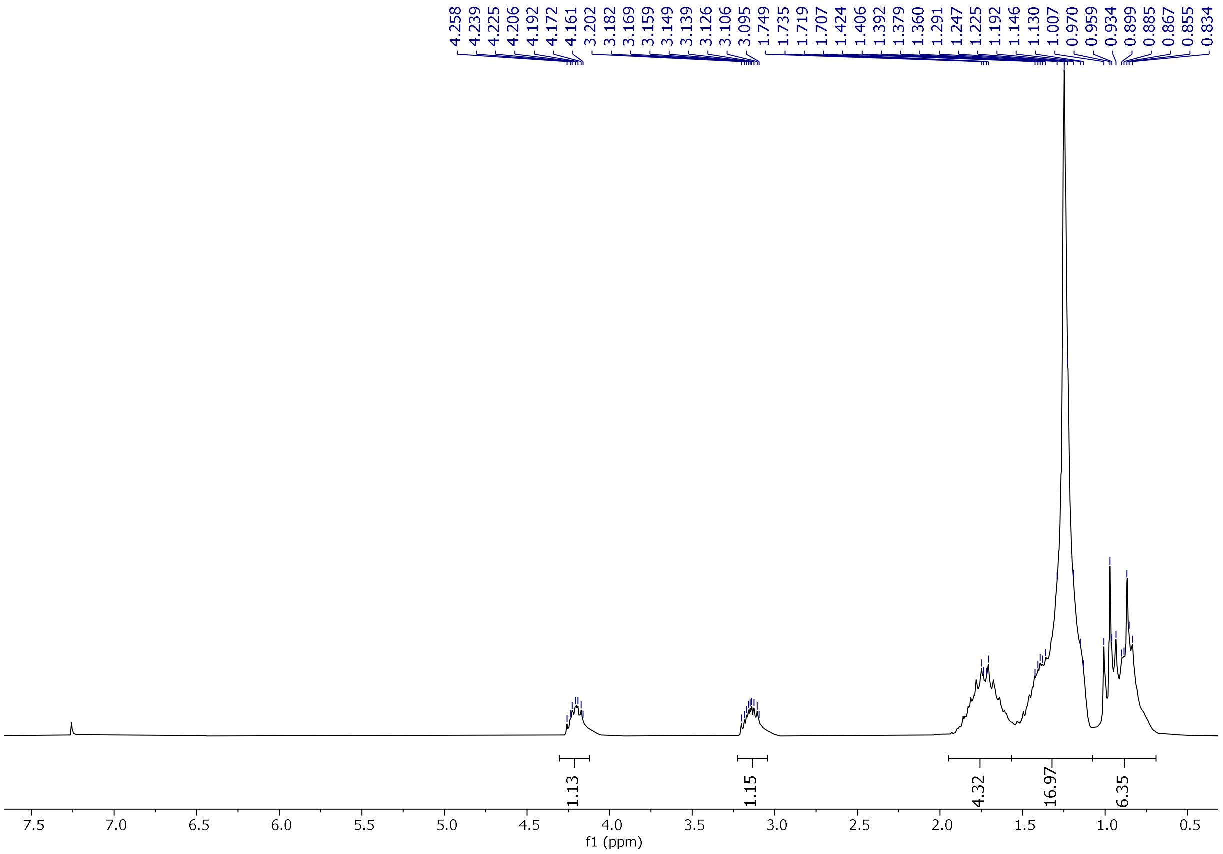
4.258
4.239
4.225
4.206
4.192
4.172
4.161
3.202
3.182
3.169
3.159
3.149
3.139
3.126
3.106
3.095
1.749
1.735
1.719
1.707
1.424
1.406
1.392
1.379
1.360
1.291
1.247
1.225
1.192
1.146
1.130
1.007
0.970
0.959
0.934
0.899
0.885
0.867
0.855
0.834
1.13
1.15
4.32
16.97
6.35
f1 (ppm)

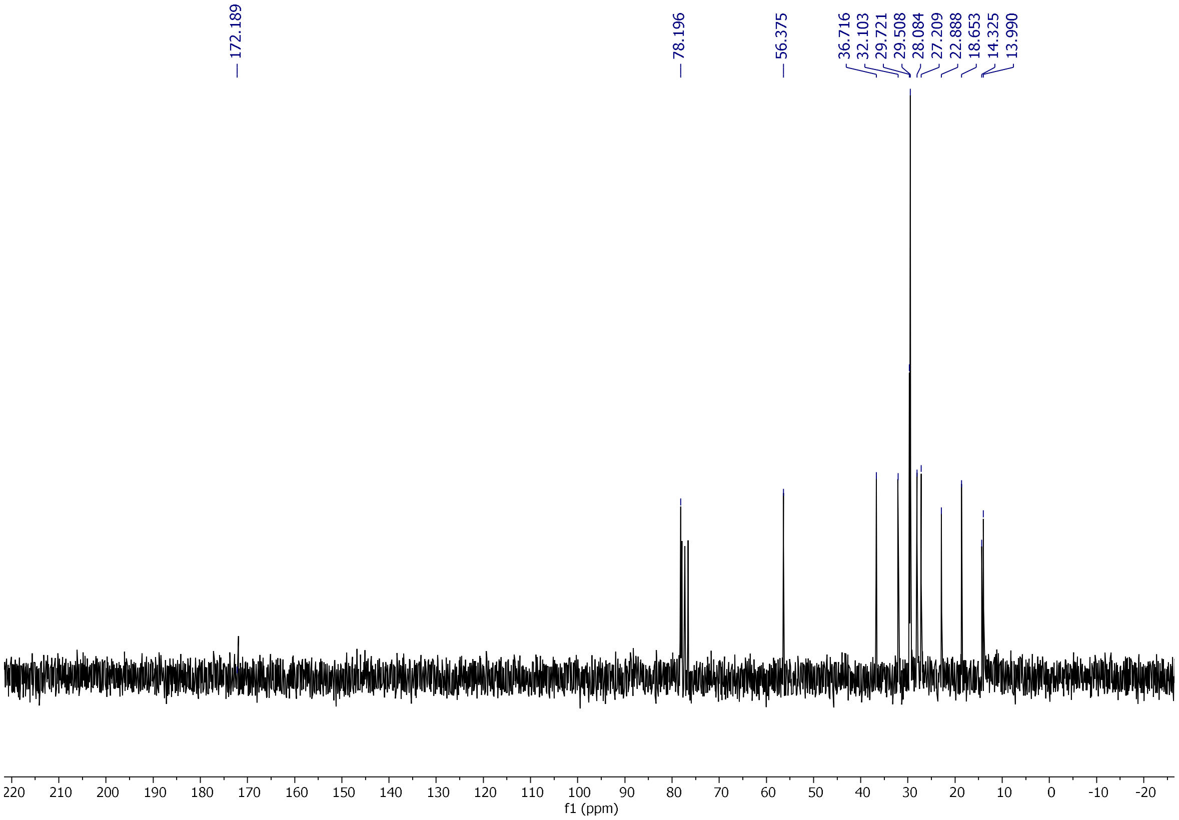
172.189
78.196
56.375
36.716
32.103
29.721
29.508
28.084
27.209
22.888
18.653
14.325
13.990
f1 (ppm)

VM027

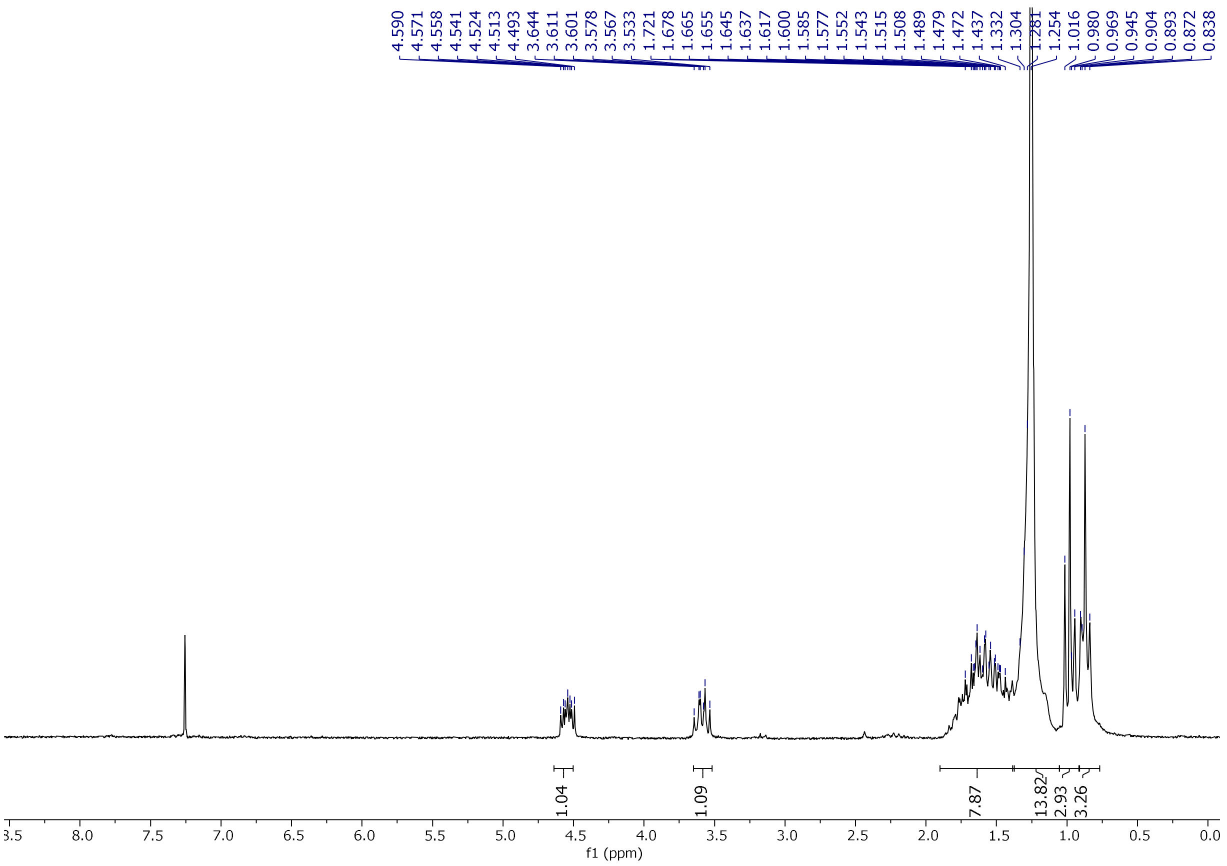
4.590 4.571 4.558 4.541 4.524 4.513 4.493 3.644 3.611 3.601 3.578 3.567 3.533 1.721 1.678 1.665 1.655 1.645 1.637 1.617 1.600 1.585 1.577 1.552 1.543 1.515 1.508 1.489 1.479 1.472 1.437 1.332 1.304 1.281 1.254 1.016 0.980 0.969 0.945 0.904 0.893 0.872 0.838
1.04
1.09
7.87
13.82
2.93
3.26
f1 (ppm)

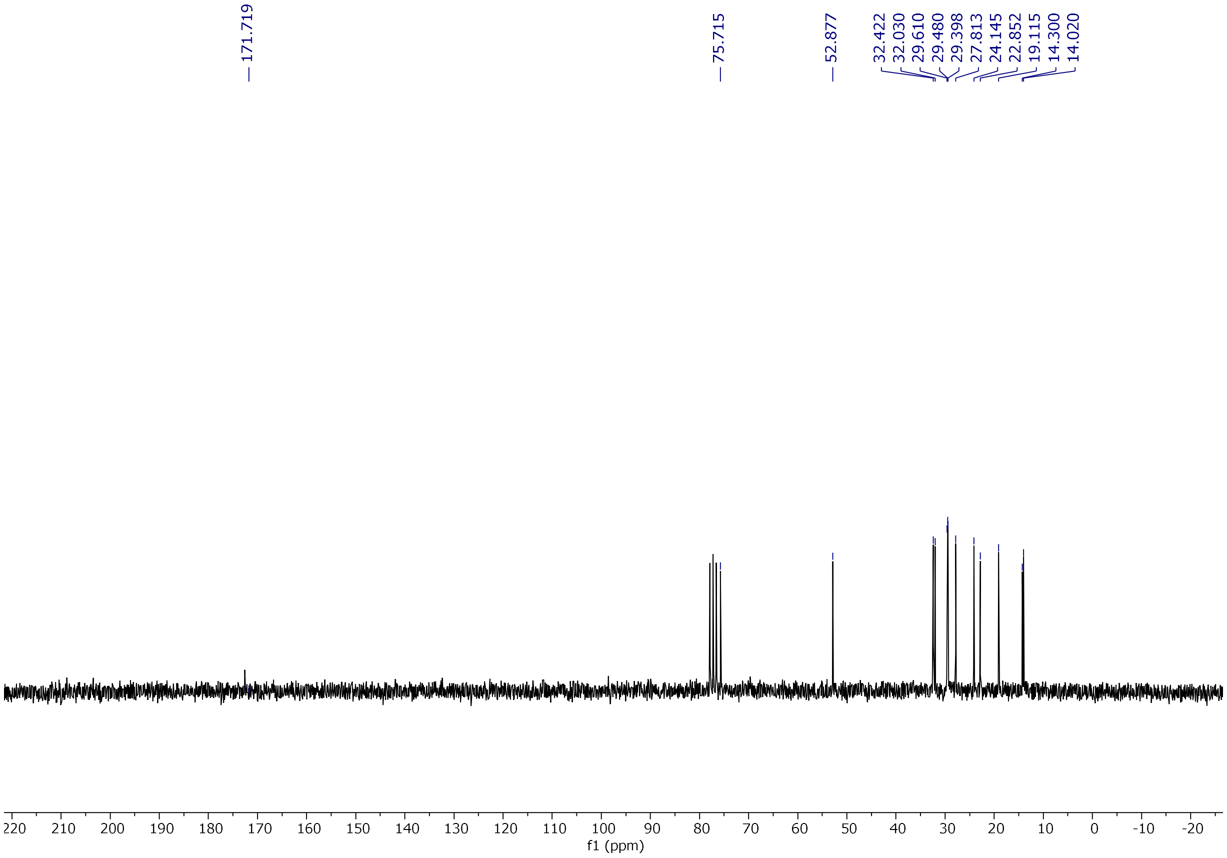
171.719
75.715
52.877
32.422 32.030 29.610 29.480 29.398 27.813 24.145 22.852 19.115 14.300 14.020
f1 (ppm)

VM028

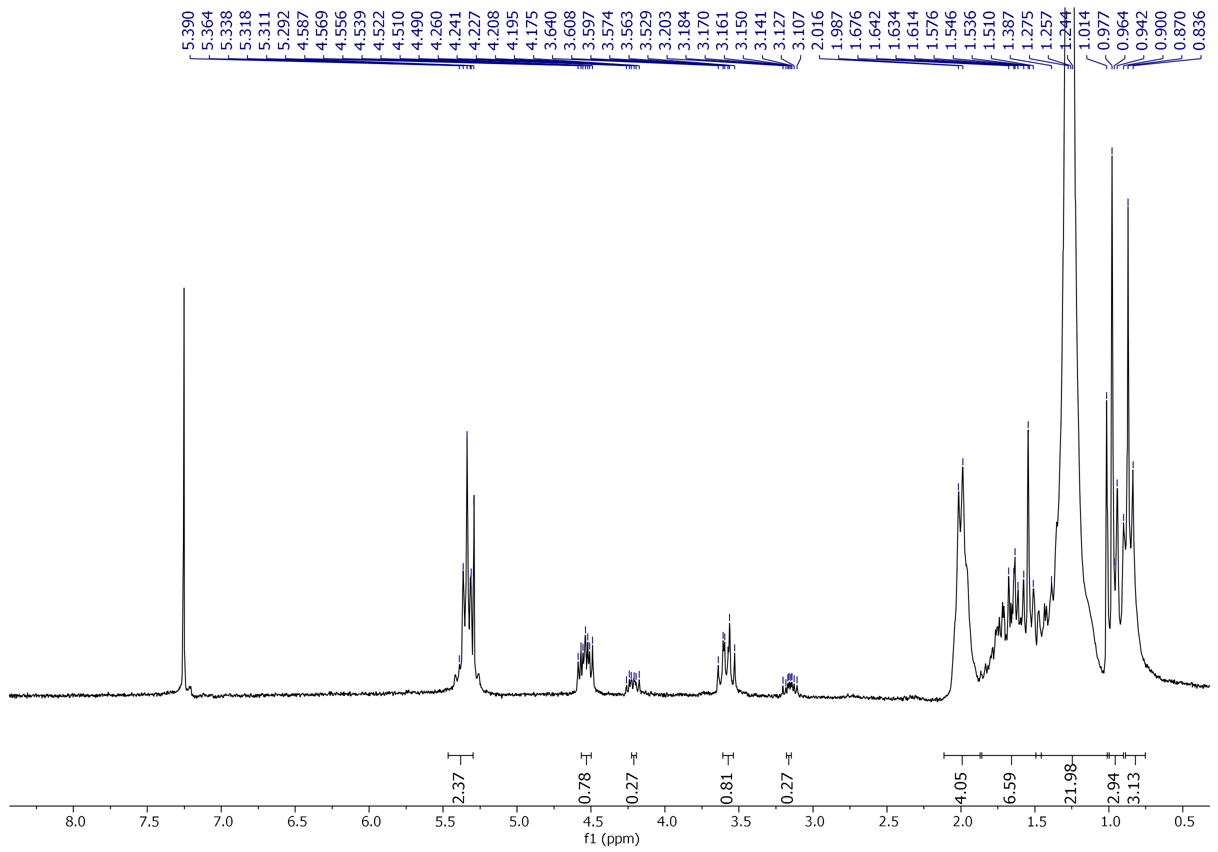
5.390
5.364
5.338
5.318
5.311
5.292
4.587
4.569
4.556
4.539
4.522
4.510
4.490
4.260
4.241
4.227
4.208
4.195
4.175
3.640
3.608
3.597
3.574
3.563
3.529
3.203
3.184
3.170
3.161
3.150
3.141
3.127
3.107
2.016
1.987
1.676
1.642
1.634
1.614
1.576
1.546
1.536
1.510
1.387
1.275
1.257
1.244
1.014
0.977
0.964
0.942
0.900
0.870
0.836
2.37
0.78
0.27
0.81
0.27
4.05
6.59
21.98
2.94
3.13
8.0 7.5 7.0 6.5 6.0 5.5 5.0 4.5 4.0 3.5 3.0 2.5 2.0 1.5 1.0 0.5
f1 (ppm)

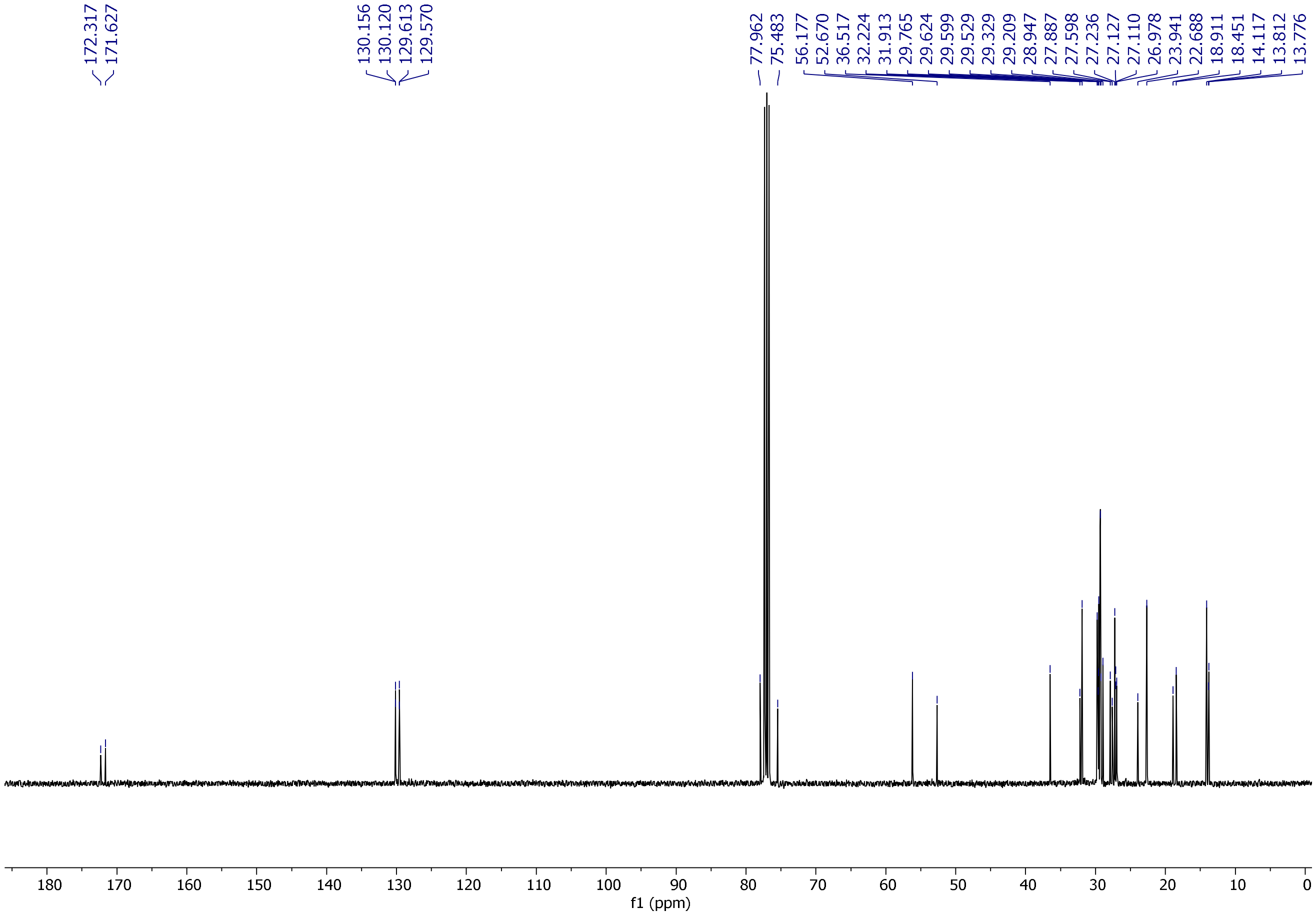
172.317
171.627
130.156
130.120
129.613
129.570
77.962
75.483
56.177
52.670
36.517
32.224
31.913
29.765
29.624
29.599
29.529
29.329
29.209
28.947
27.887
27.598
27.236
27.127
27.110
26.978
23.941
22.688
18.911
18.451
14.117
13.812
13.776
180 170 160 150 140 130 120 110 100 90 80 70 60 50 40 30 20 10 0
f1 (ppm)

VM029

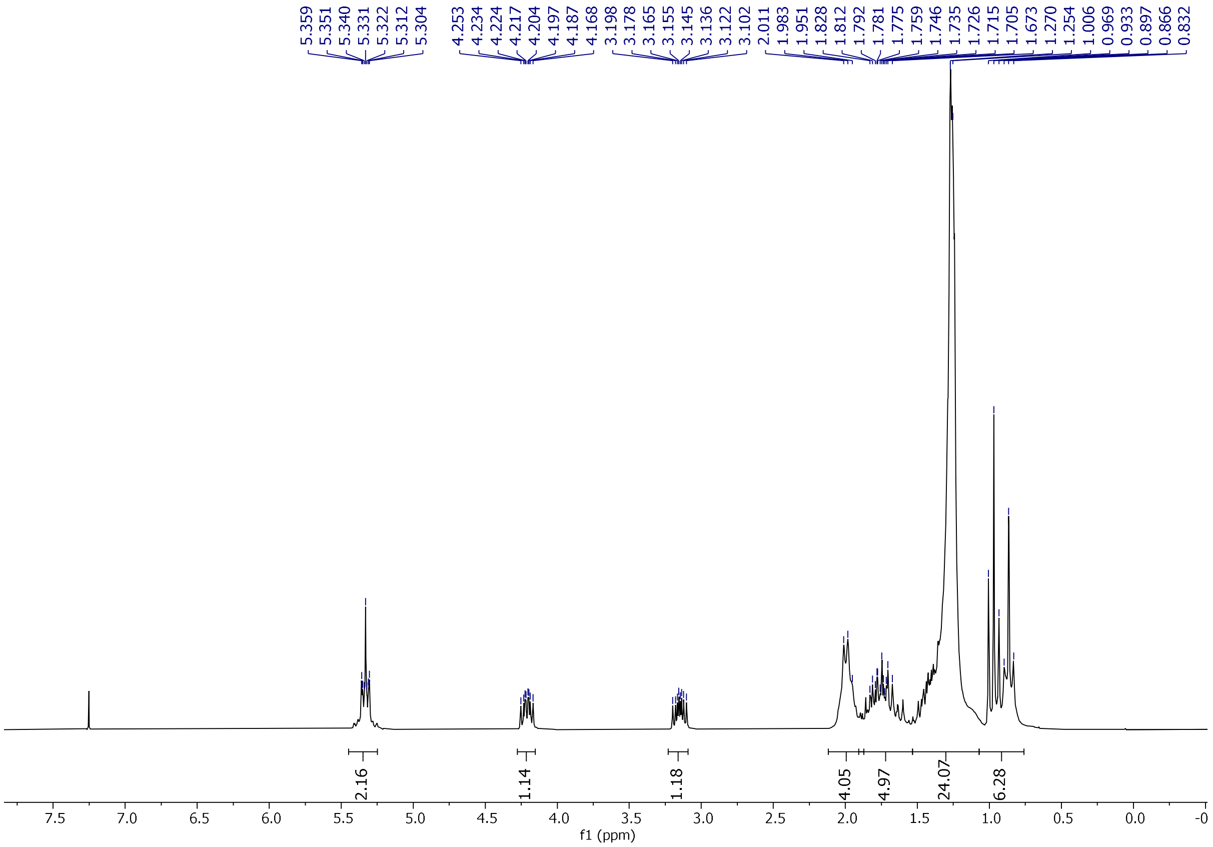
5.359
5.351
5.340
5.331
5.322
5.312
5.304
4.253
4.234
4.224
4.217
4.204
4.197
4.187
4.168
3.198
3.178
3.165
3.155
3.145
3.136
3.122
3.102
2.011
1.983
1.951
1.828
1.812
1.792
1.781
1.775
1.759
1.746
1.735
1.726
1.715
1.705
1.673
1.270
1.254
1.006
0.969
0.933
0.897
0.866
0.832
2.16
1.14
1.18
4.05
4.97
24.07
6.28
f1 (ppm)

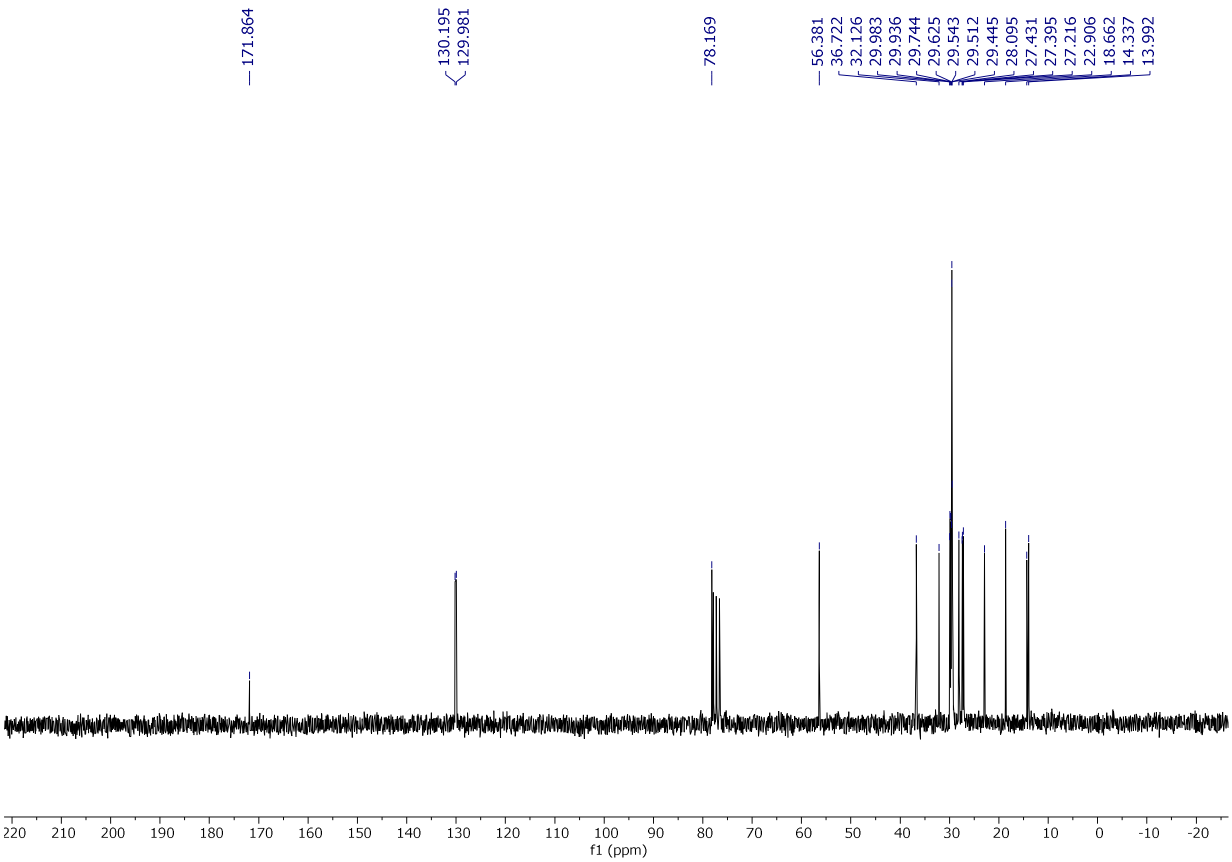
171.864
130.195
129.981
78.169
56.381
36.722
32.126
29.983
29.936
29.744
29.625
29.543
29.512
29.445
28.095
27.431
27.395
27.216
22.906
18.662
14.337
13.992
f1 (ppm)

VM030

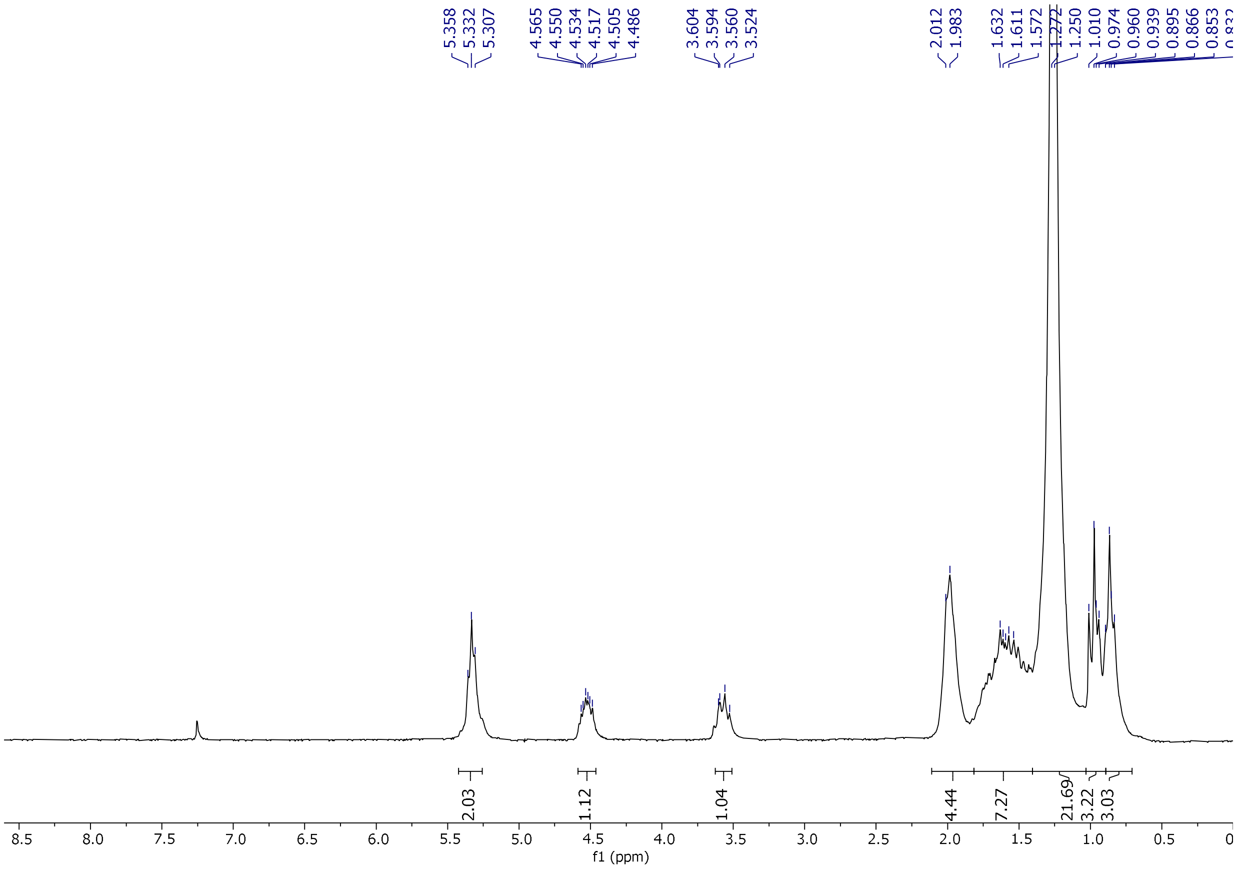
5.358
5.332
5.307
4.565
4.550
4.534
4.517
4.505
4.486
3.604
3.594
3.560
3.524
2.012
1.983
1.632
1.611
1.572
1.272
1.250
1.010
0.974
0.960
0.939
0.895
0.866
0.853
2.03
1.12
1.04
4.44
7.27
21.69
3.22
3.03
f1 (ppm)

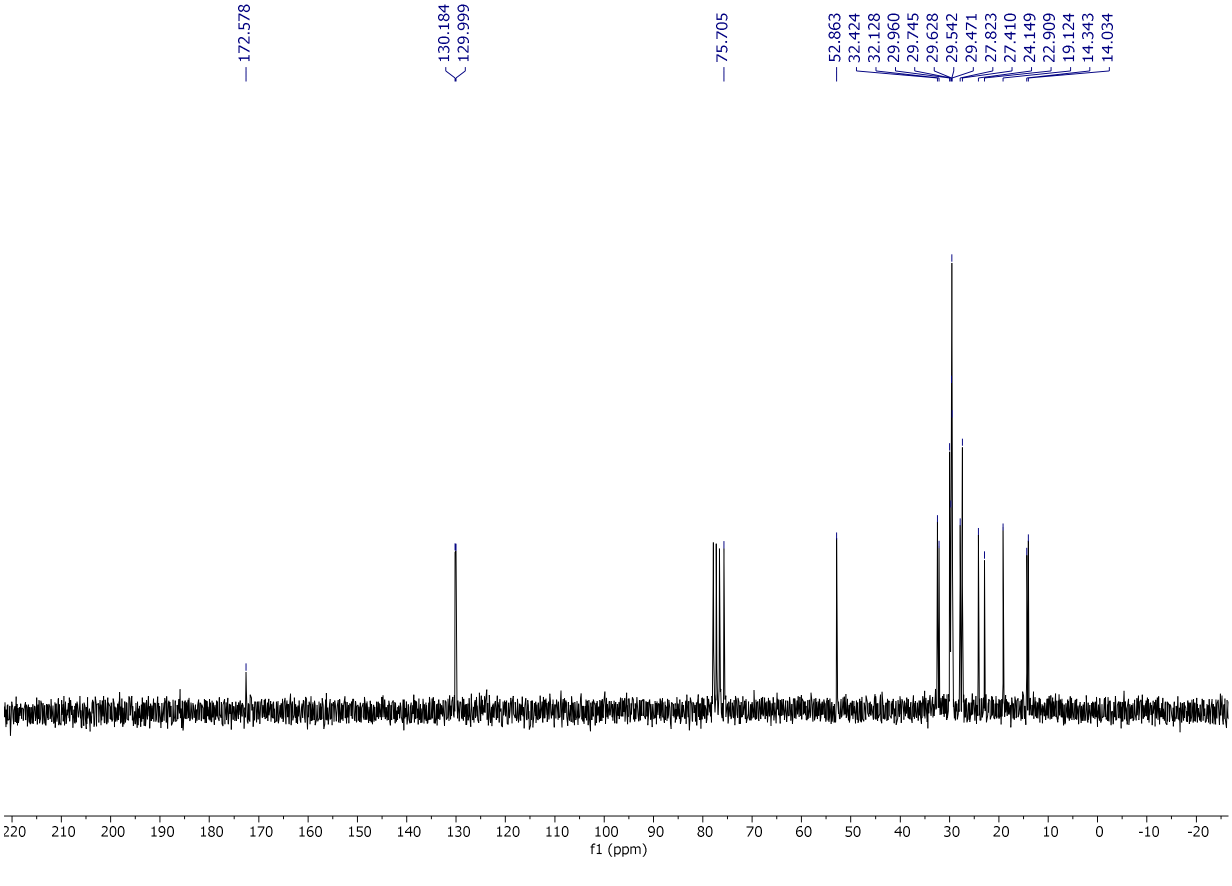
172.578
130.184
129.999
75.705
52.863
32.424
32.128
29.960
29.745
29.628
29.542
29.471
27.823
27.410
24.149
22.909
19.124
14.343
14.034
f1 (ppm)

VM036

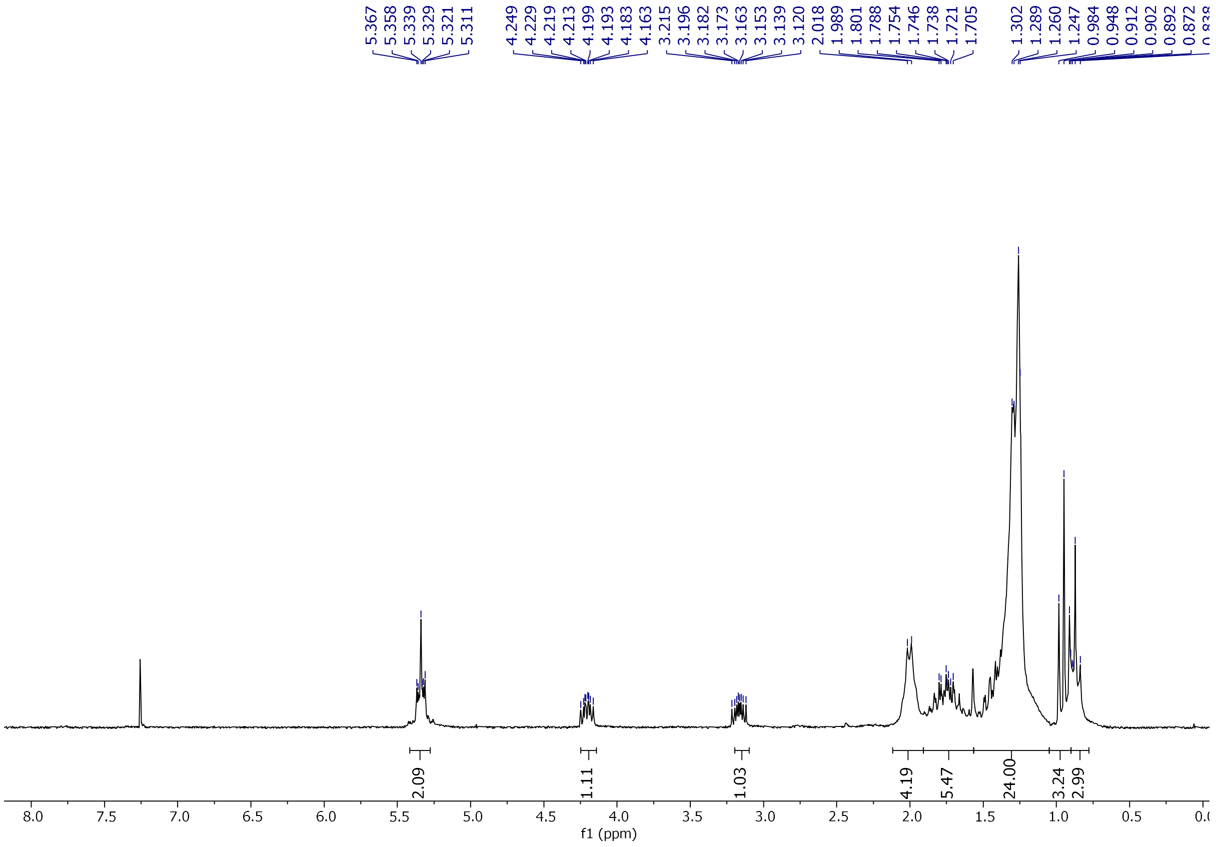
5.367
5.358
5.339
5.329
5.321
5.311
4.249
4.229
4.219
4.213
4.199
4.193
4.183
4.163
3.215
3.196
3.182
3.173
3.163
3.153
3.139
3.120
2.018
1.989
1.801
1.788
1.754
1.746
1.738
1.721
1.705
1.302
1.289
1.260
1.247
0.984
0.948
0.912
0.902
0.892
0.872
2.09
1.11
1.03
4.19
5.47
24.00
3.24
2.99
8.0
7.5
7.0
6.5
6.0
5.5
5.0
4.5
4.0
3.5
3.0
2.5
2.0
1.5
1.0
0.5
f1 (ppm)

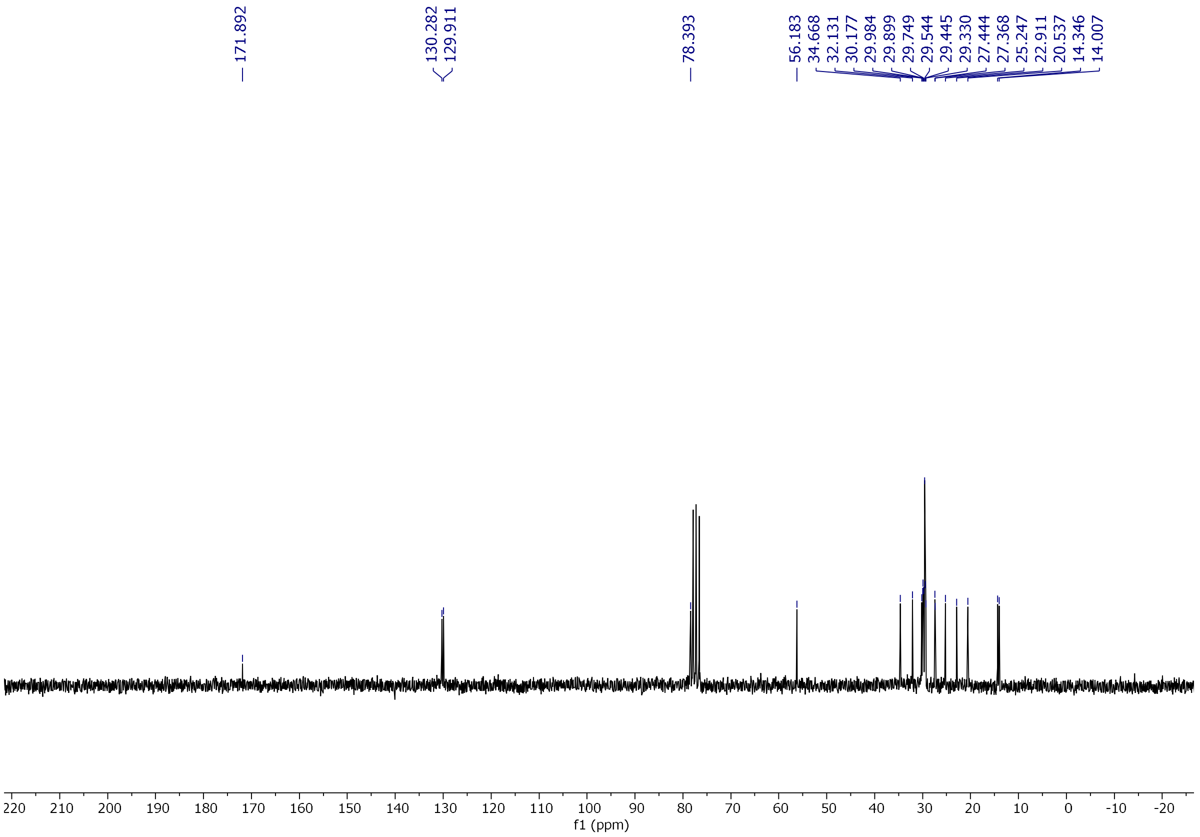
171.892
130.282
129.911
78.393
56.183
34.668
32.131
30.177
29.984
29.899
29.749
29.544
29.445
29.330
27.444
27.368
25.247
22.911
20.537
14.346
14.007
220
210
200
190
180
170
160
150
140
130
120
110
100
90
80
70
60
50
40
30
20
10
0
-10
-20
f1 (ppm)

VM037

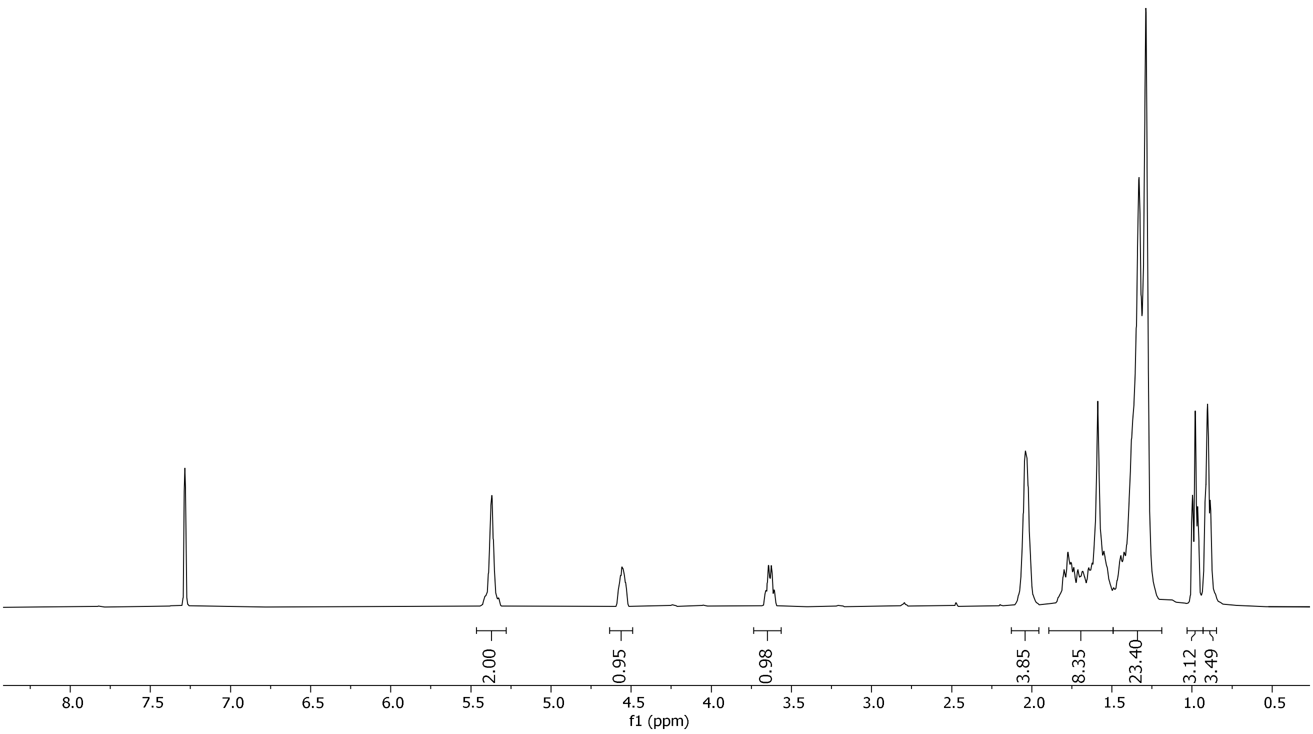
2.00
0.95
0.98
3.85
8.35
23.40
3.12
3.49
8.0 7.5 7.0 6.5 6.0 5.5 5.0 4.5 4.0 3.5 3.0 2.5 2.0 1.5 1.0 0.5
f1 (ppm)

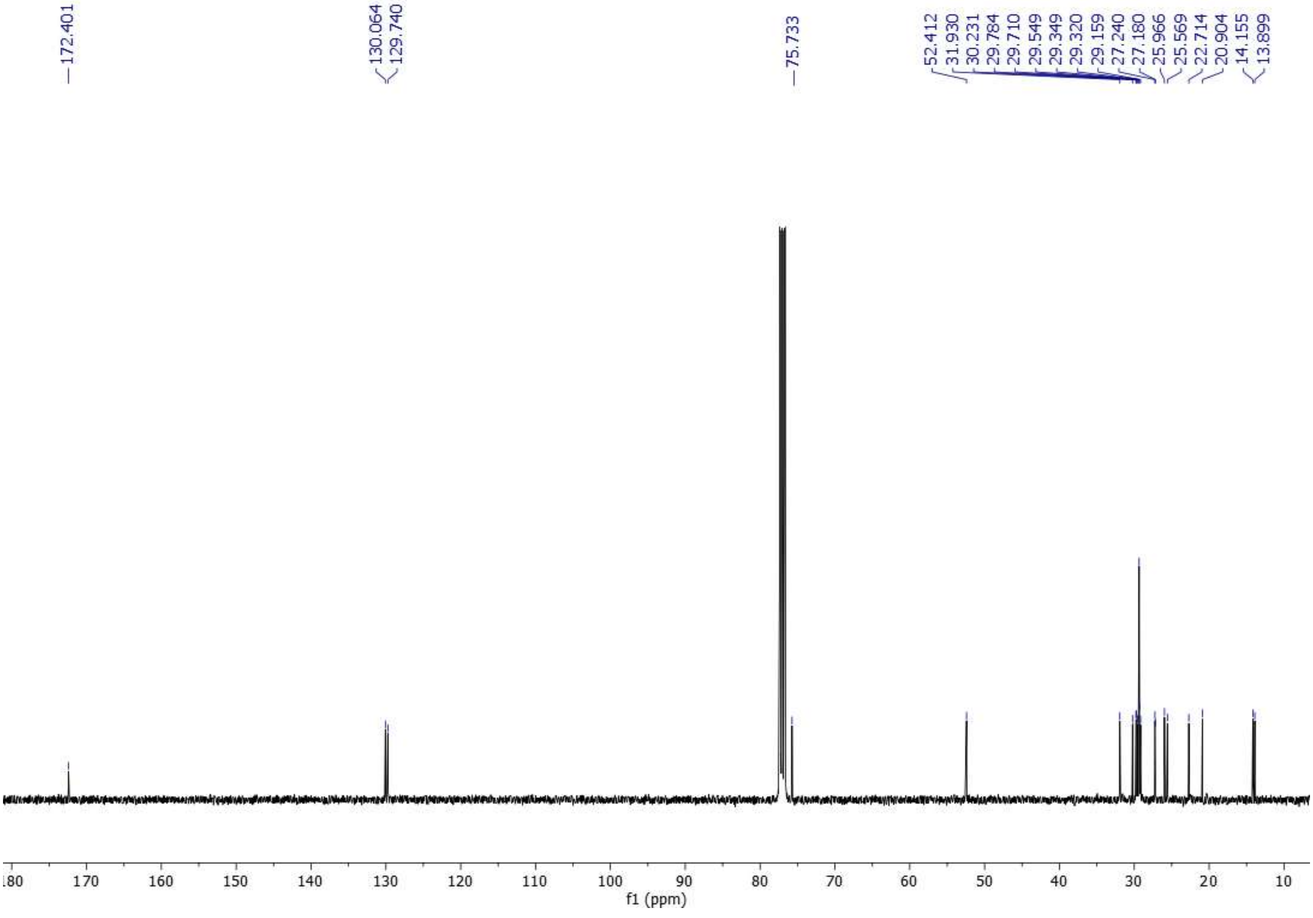
172.401
130.064
129.740
75.733
52.412
31.930
30.231
29.784
29.710
29.549
29.349
29.320
29.159
27.240
27.180
25.966
25.569
22.714
20.904
14.155
13.899
180 170 160 150 140 130 120 110 100 90 80 70 60 50 40 30 20 10
f1 (ppm)

VM038

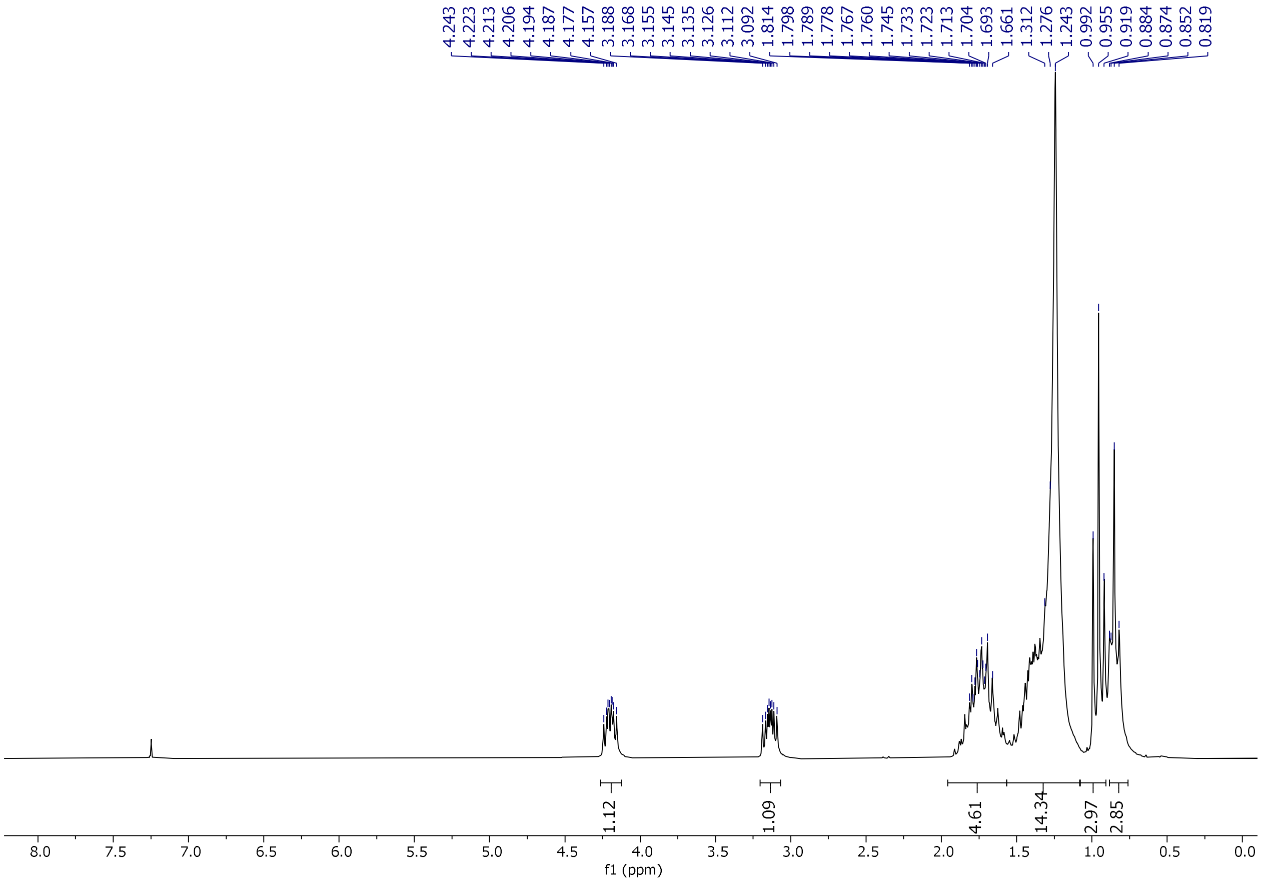
4.243
4.223
4.213
4.206
4.194
4.187
4.177
4.157
3.188
3.168
3.155
3.145
3.135
3.126
3.112
3.092
1.814
1.798
1.789
1.778
1.767
1.760
1.745
1.733
1.723
1.713
1.704
1.693
1.661
1.312
1.276
1.243
0.992
0.955
0.919
0.884
0.874
0.852
0.819
1.12
1.09
4.61
14.34
2.97
2.85
f1 (ppm)

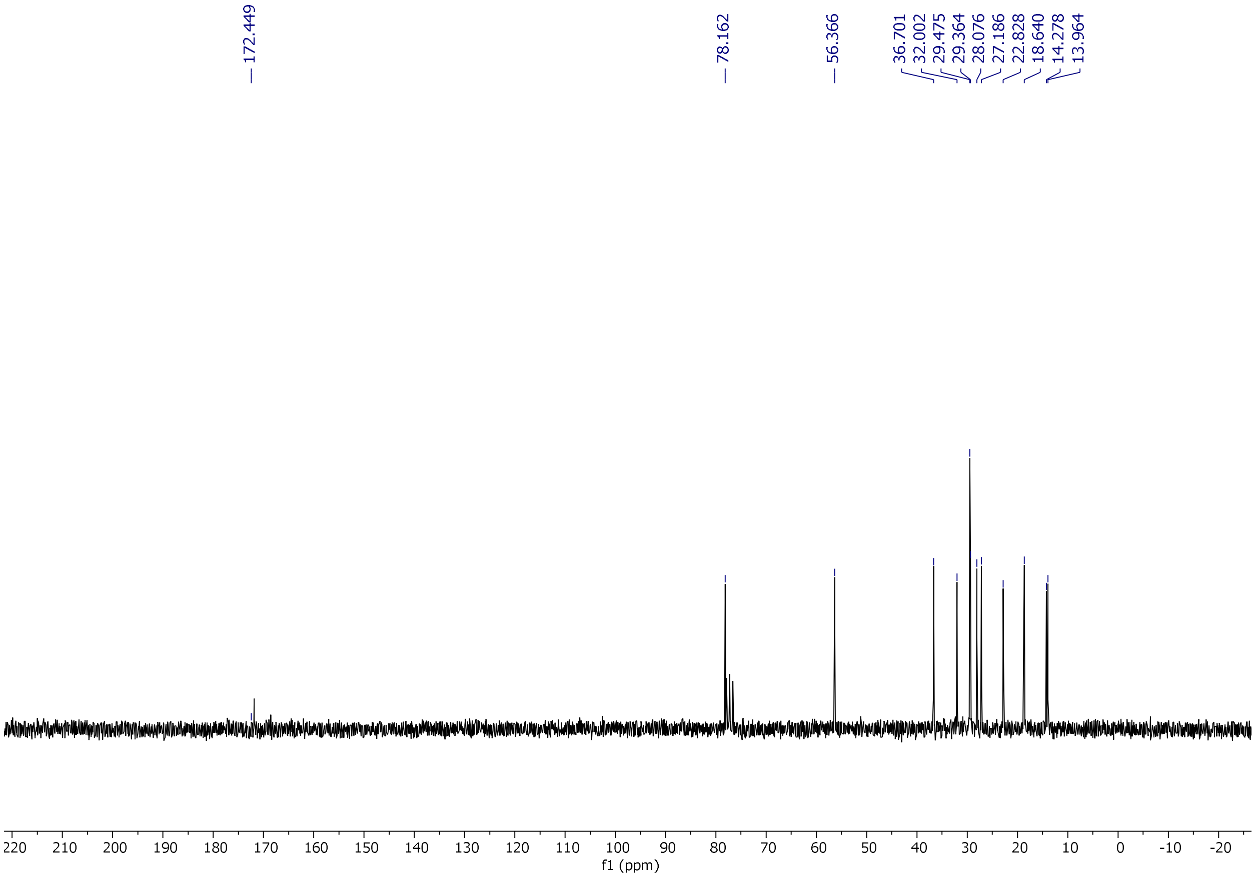
172.449
78.162
56.366
36.701
32.002
29.475
29.364
28.076
27.186
22.828
18.640
14.278
13.964
f1 (ppm)

VM039

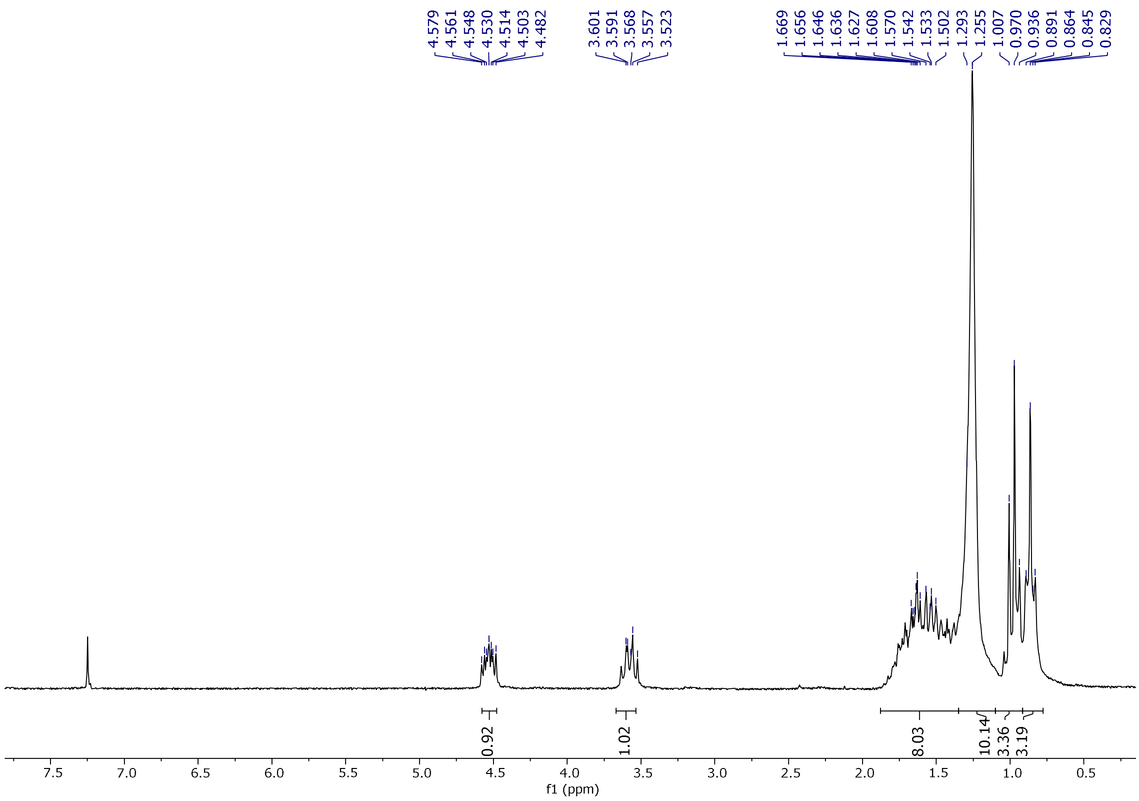

4.579
4.561
4.548
4.530
4.514
4.503
4.482
3.601
3.591
3.568
3.557
3.523
1.669
1.656
1.646
1.636
1.627
1.608
1.570
1.542
1.533
1.502
1.293
1.255
1.007
0.970
0.936
0.891
0.864
0.845
0.829
0.92
1.02
8.03
10.14
3.36
3.19
7.5 7.0 6.5 6.0 5.5 5.0 4.5 4.0 3.5 3.0 2.5 2.0 1.5 1.0 0.5
f1 (ppm)

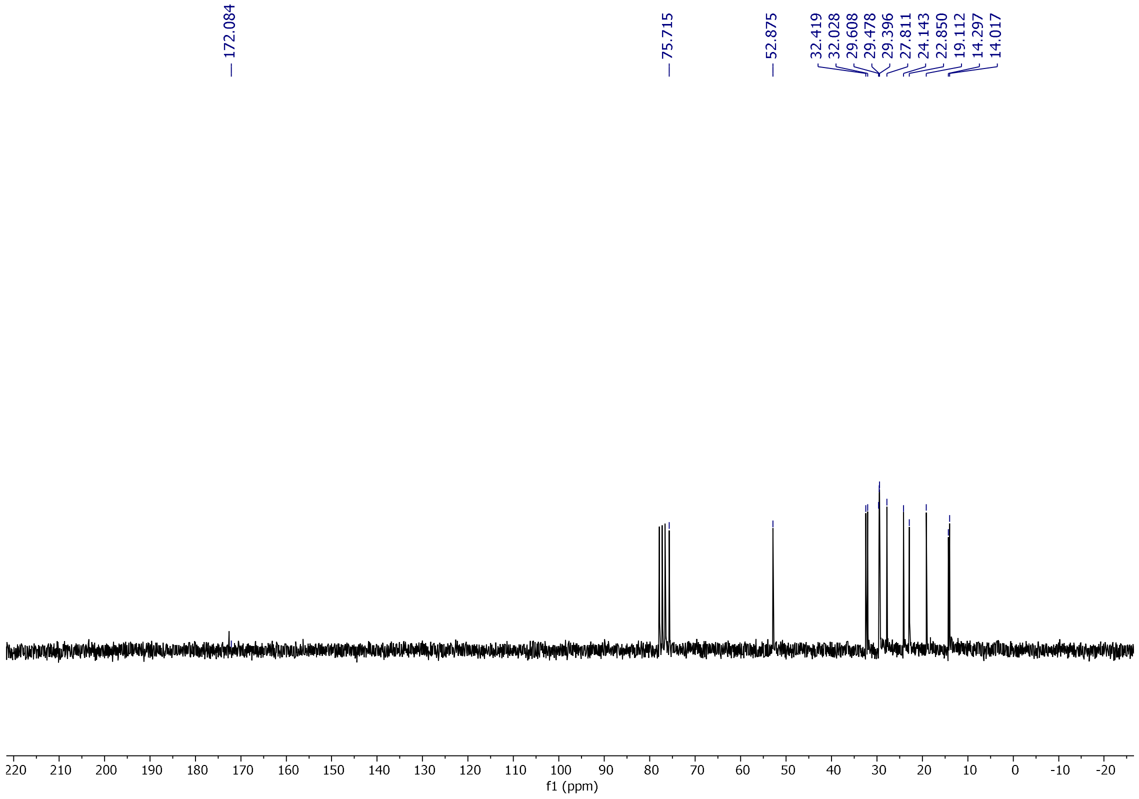

172.084
75.715
52.875
32.419
32.028
29.608
29.478
29.396
27.811
24.143
22.850
19.112
14.297
14.017
220 210 200 190 180 170 160 150 140 130 120 110 100 90 80 70 60 50 40 30 20 10 0 -10 -20
f1 (ppm)

VM040

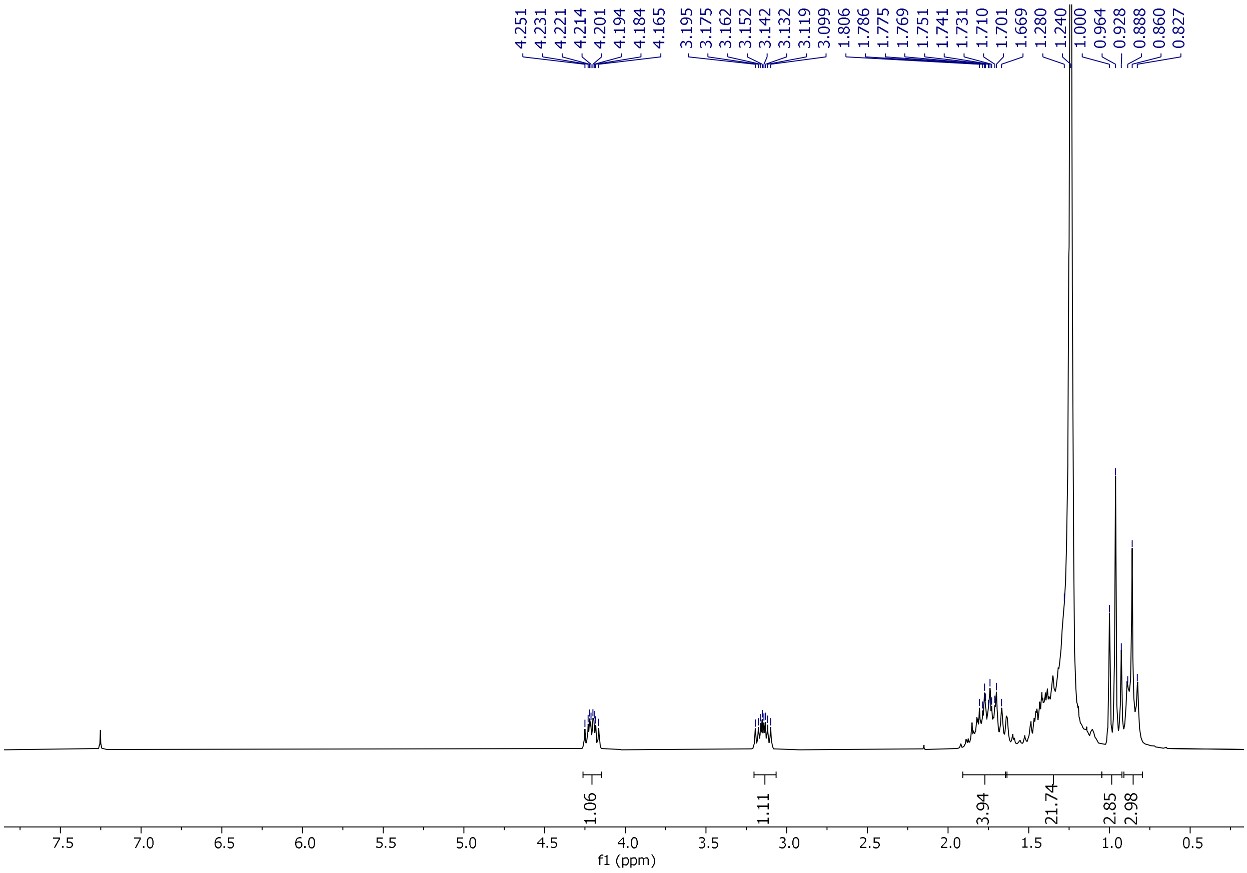
4.251
4.231
4.221
4.214
4.201
4.194
4.184
4.165
3.195
3.175
3.162
3.152
3.142
3.132
3.119
3.099
1.806
1.786
1.775
1.769
1.751
1.741
1.731
1.710
1.701
1.669
1.280
1.240
1.000
0.964
0.928
0.888
0.860
0.827
1.06
1.11
3.94
21.74
2.85
2.98
f1 (ppm)

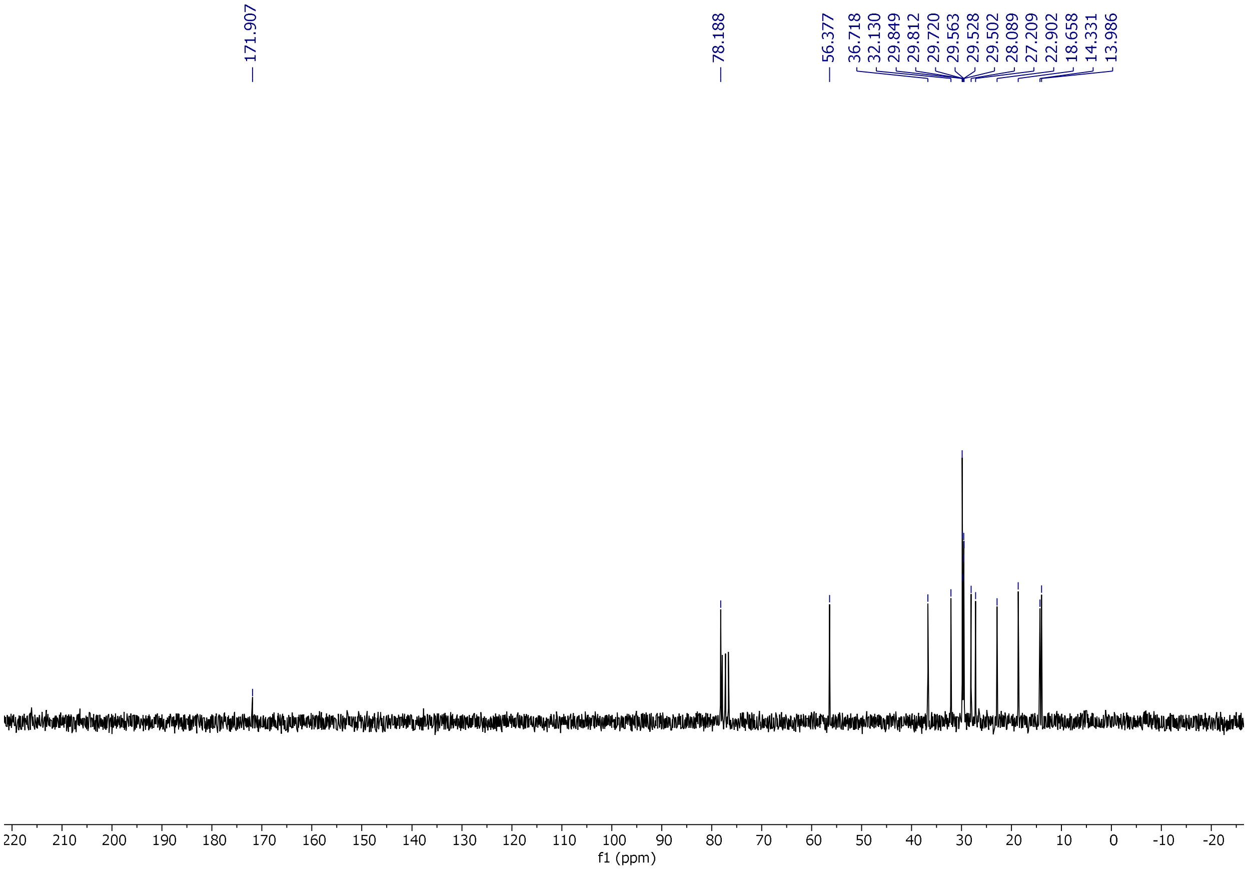
171.907
78.188
56.377
36.718
32.130
29.849
29.812
29.720
29.563
29.528
29.502
28.089
27.209
22.902
18.658
14.331
13.986
f1 (ppm)

VM041

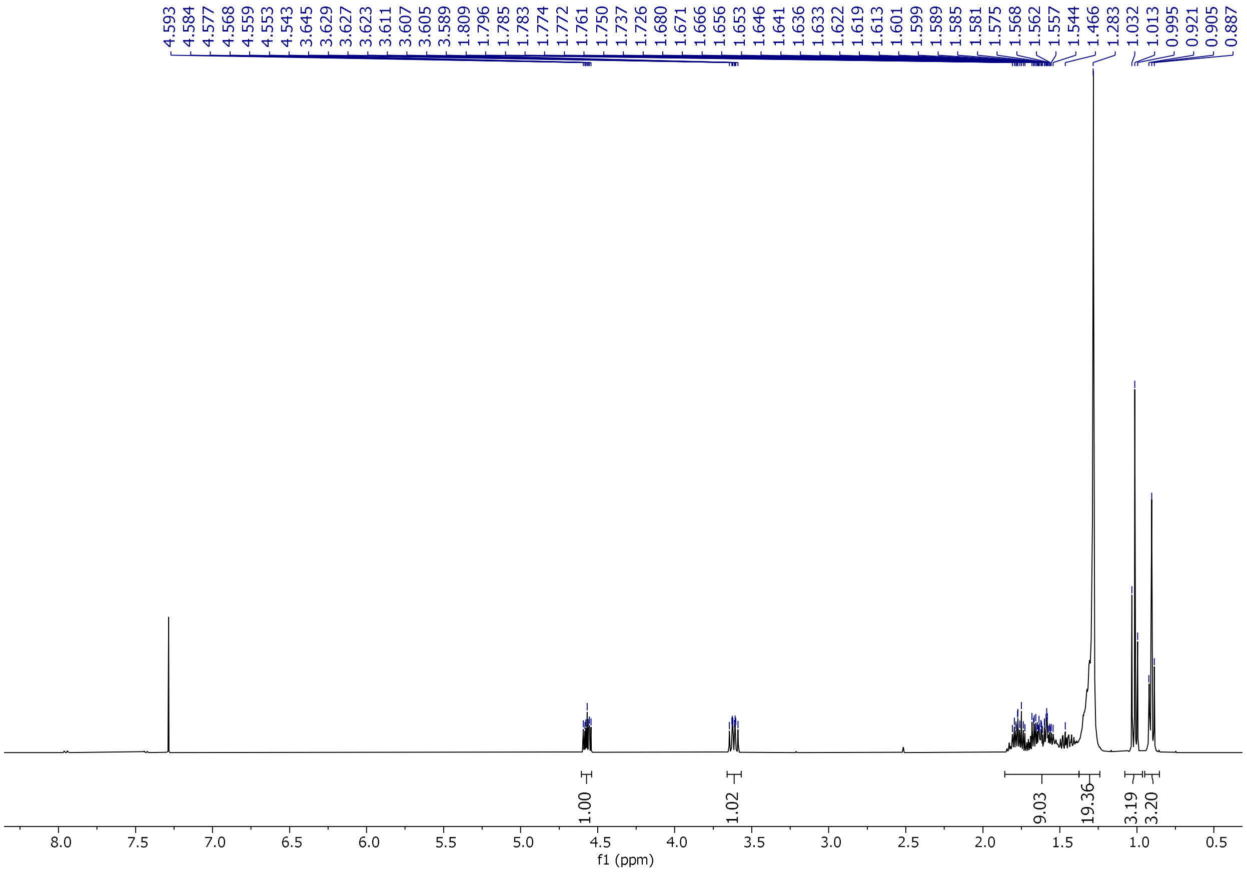

4.593
4.584
4.577
4.568
4.559
4.553
4.543
3.645
3.629
3.627
3.623
3.611
3.607
3.605
3.589
1.809
1.796
1.785
1.783
1.774
1.772
1.761
1.750
1.737
1.726
1.680
1.671
1.666
1.656
1.653
1.646
1.641
1.636
1.633
1.622
1.619
1.613
1.601
1.599
1.589
1.585
1.581
1.575
1.568
1.562
1.557
1.544
1.466
1.283
1.032
1.013
0.995
0.921
0.905
0.887
1.00
1.02
9.03
19.36
3.19
3.20
8.0
7.5
7.0
6.5
6.0
5.5
5.0
4.5
4.0
3.5
3.0
2.5
2.0
1.5
1.0
0.5
f1 (ppm)

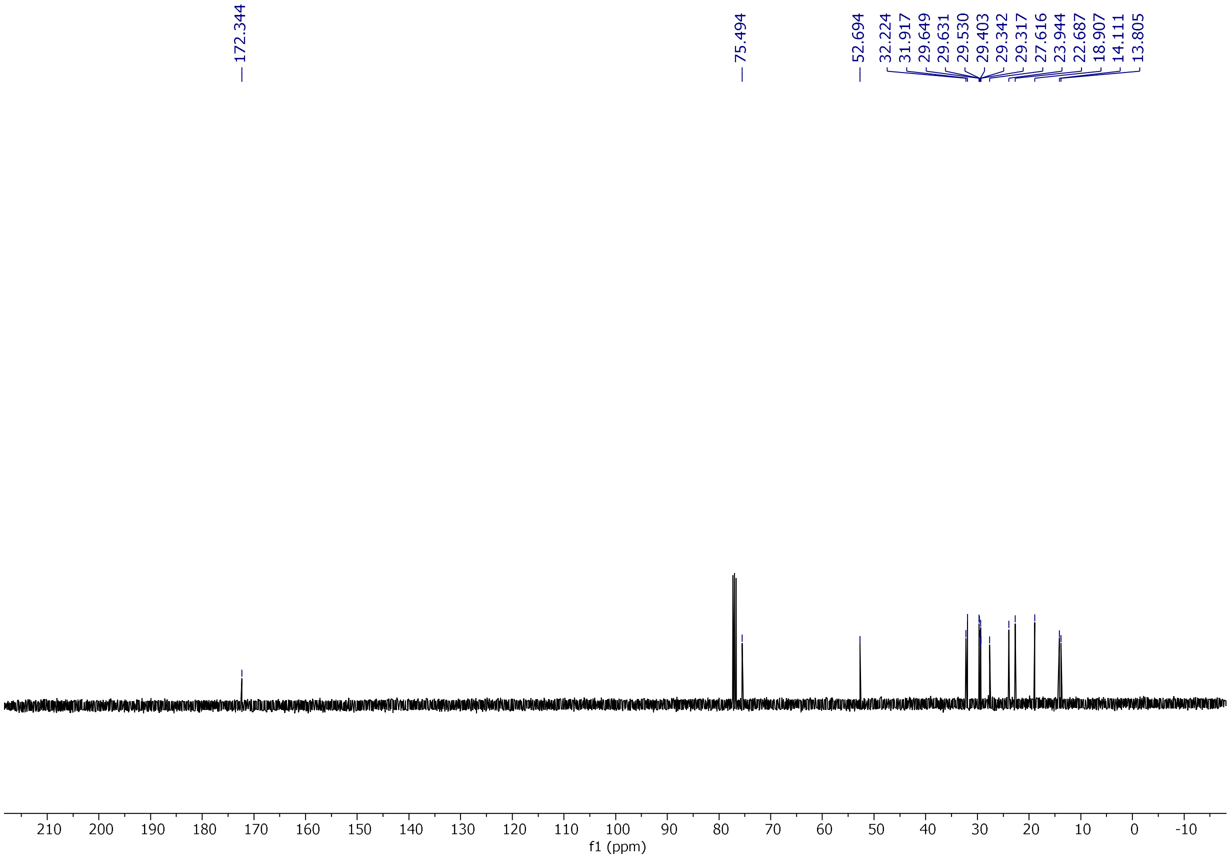

172.344
75.494
52.694
32.224
31.917
29.649
29.631
29.530
29.403
29.342
29.317
27.616
23.944
22.687
18.907
14.111
13.805
210
200
190
180
170
160
150
140
130
120
110
100
90
80
70
60
50
40
30
20
10
0
-10
f1 (ppm)

VM042

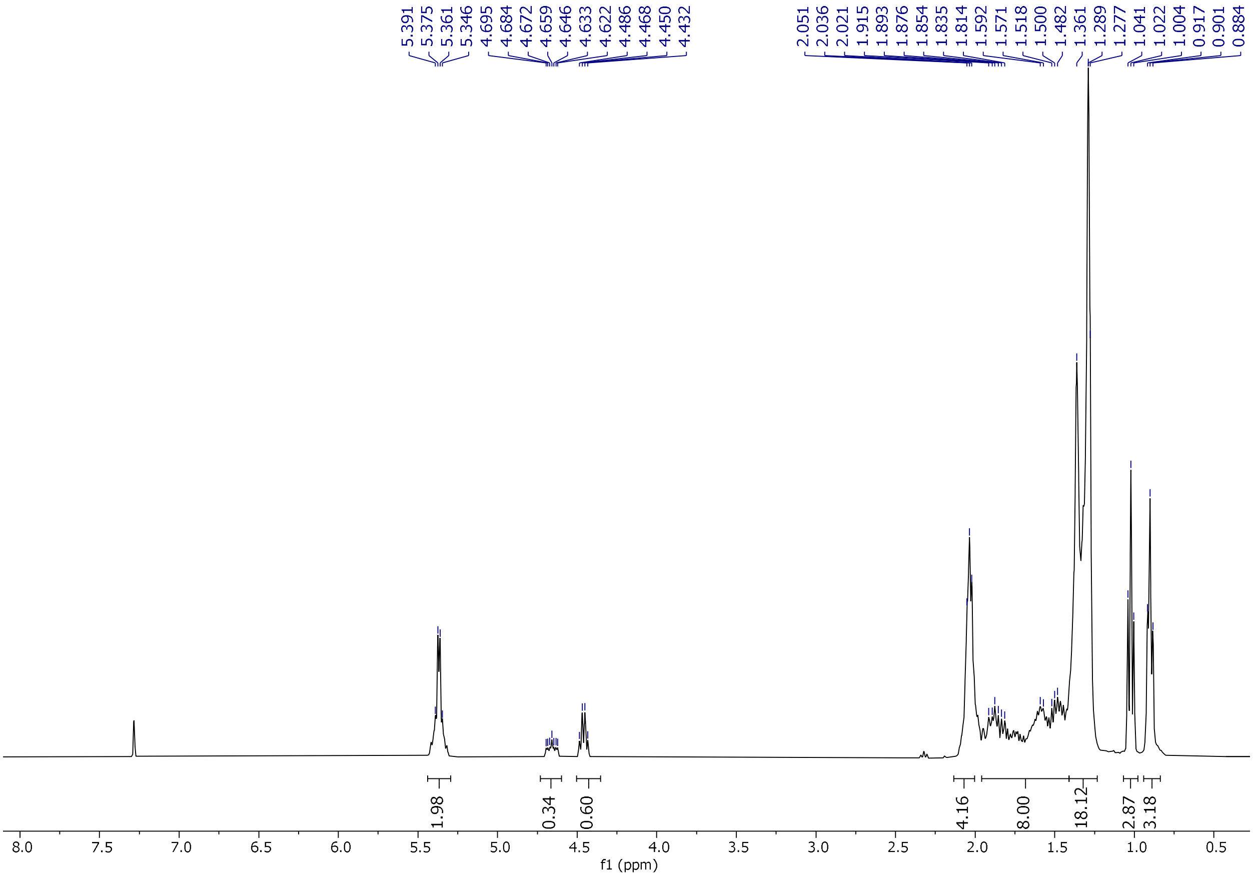
5.391
5.375
5.361
5.346
4.695
4.684
4.672
4.659
4.646
4.633
4.622
4.486
4.468
4.450
4.432
2.051
2.036
2.021
1.915
1.893
1.876
1.854
1.835
1.814
1.592
1.571
1.518
1.500
1.482
1.361
1.289
1.277
1.041
1.022
1.004
0.917
0.901
0.884
1.98
0.34
0.60
4.16
8.00
18.12
2.87
3.18
f1 (ppm)

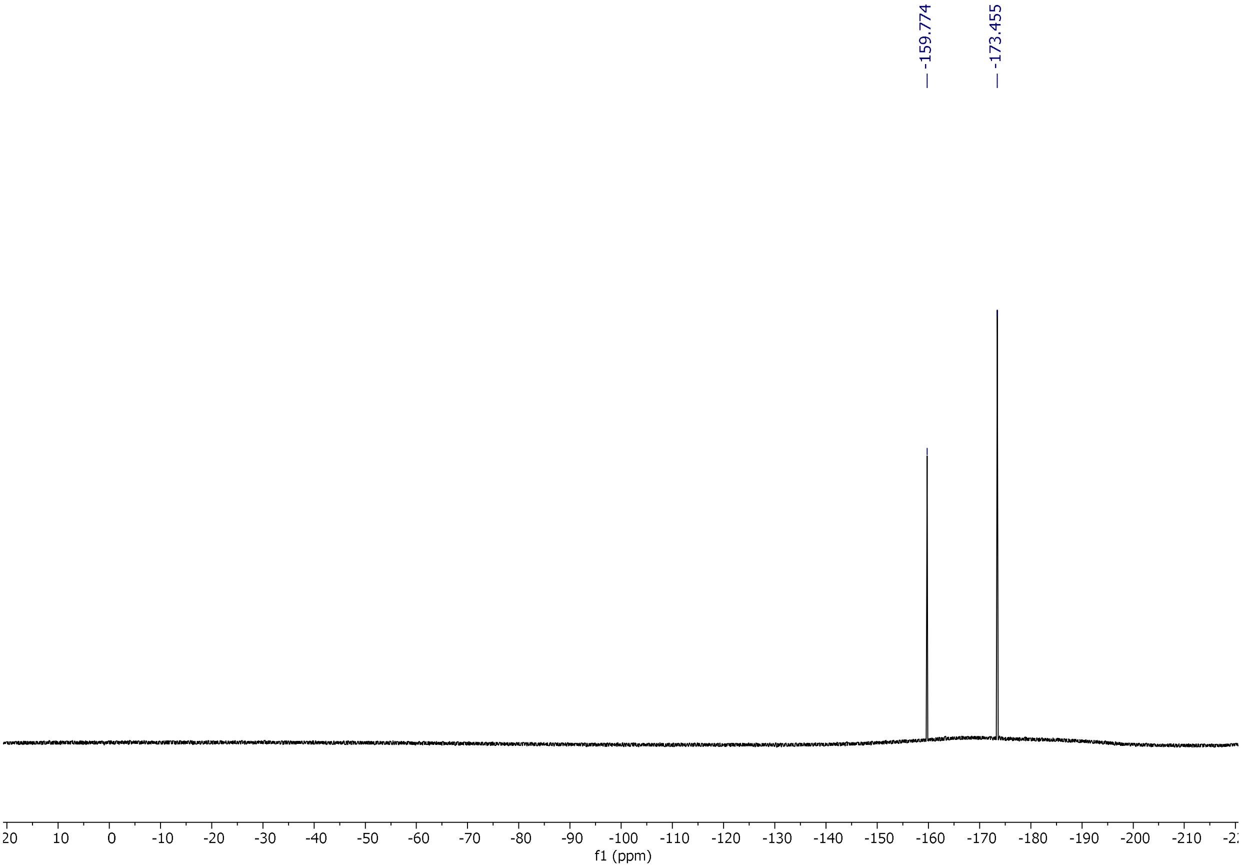
-159.774
-173.455
f1 (ppm)

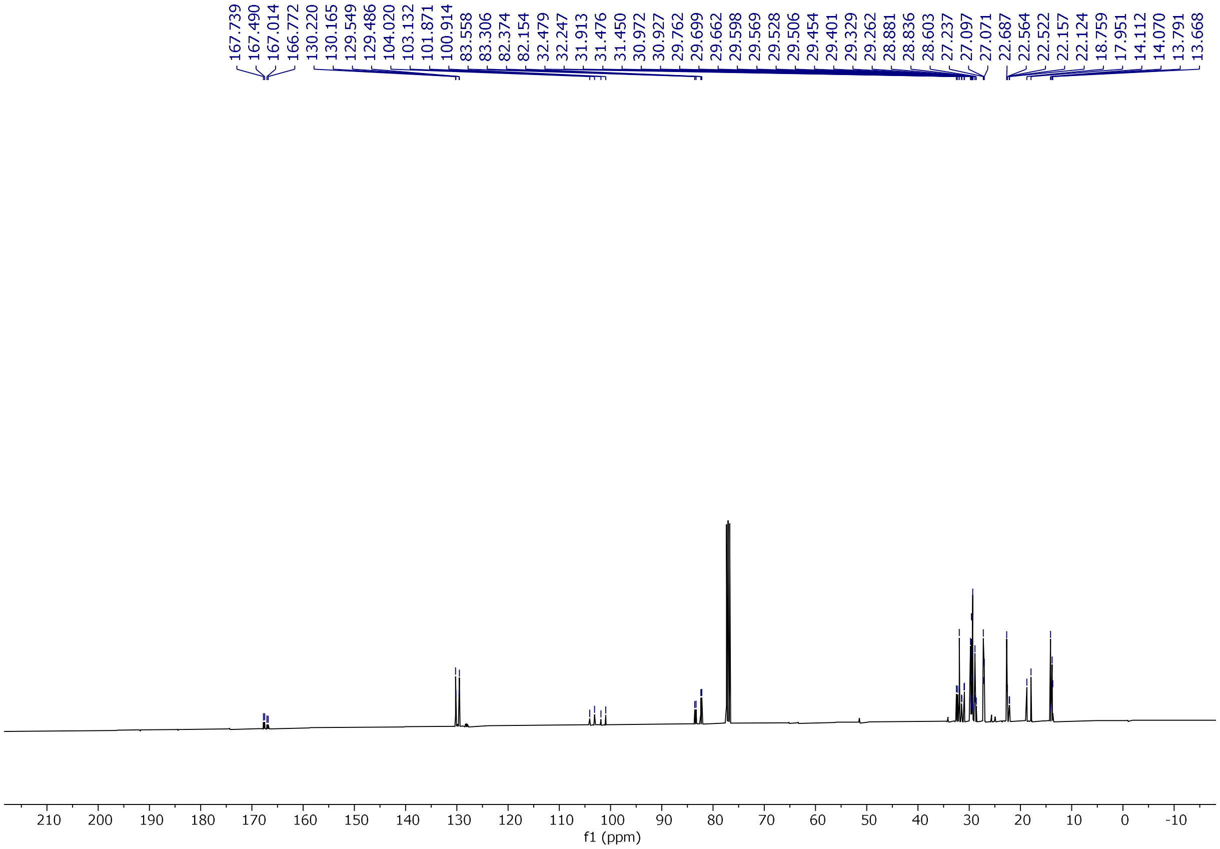
167.739
167.490
167.014
166.772
130.220
130.165
129.549
129.486
104.020
103.132
101.871
100.914
83.558
83.306
82.374
82.154
32.479
32.247
31.913
31.476
31.450
30.972
30.927
29.762
29.699
29.662
29.598
29.569
29.528
29.506
29.454
29.401
29.329
29.262
28.881
28.836
28.603
27.237
27.097
27.071
22.687
22.564
22.522
22.157
22.124
18.759
17.951
14.112
14.070
13.791
13.668
210 200 190 180 170 160 150 140 130 120 110 100 90 80 70 60 50 40 30 20 10 0 -10
f1 (ppm)

VM043

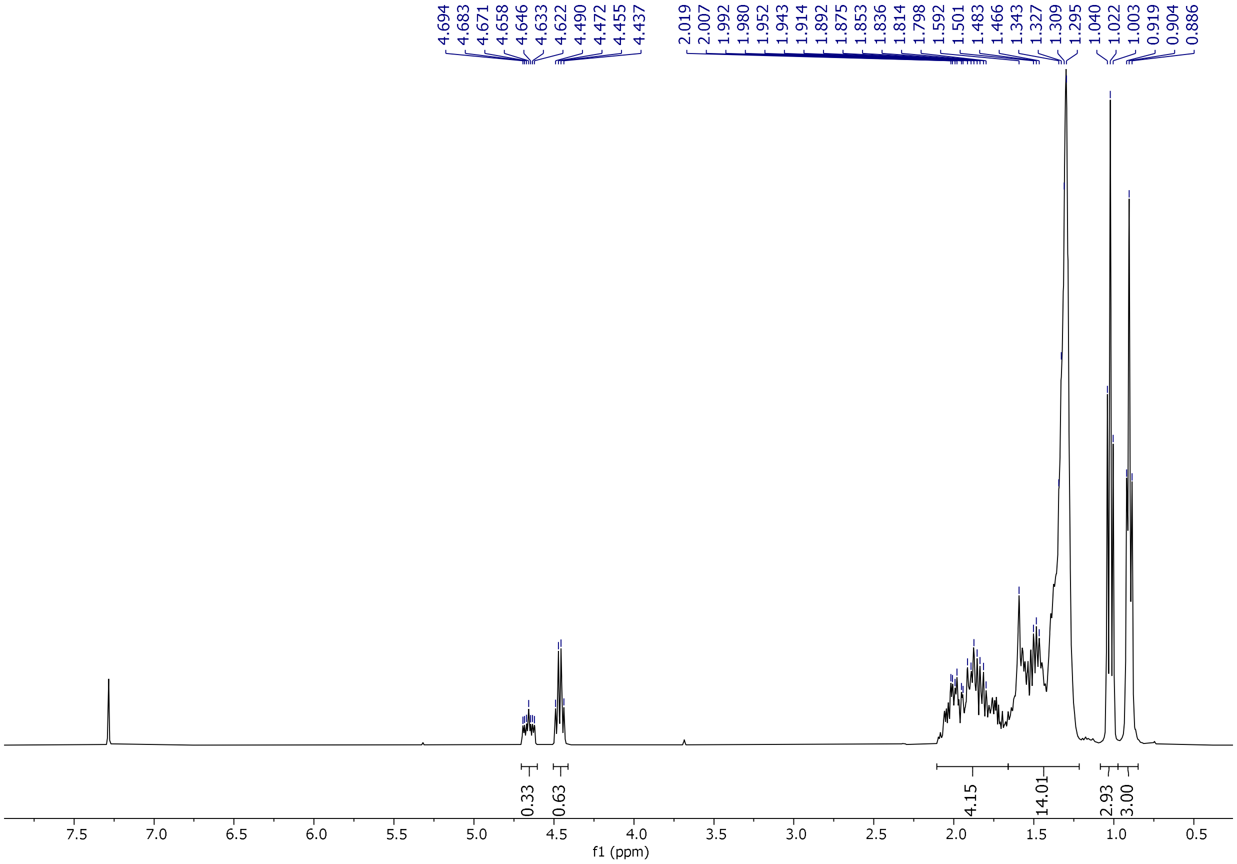

4.694
4.683
4.671
4.658
4.646
4.633
4.622
4.490
4.472
4.455
4.437
2.019
2.007
1.992
1.980
1.952
1.943
1.914
1.892
1.875
1.853
1.836
1.814
1.798
1.592
1.501
1.483
1.466
1.343
1.327
1.309
1.295
1.040
1.022
1.003
0.919
0.904
0.886
0.33
0.63
4.15
14.01
2.93
3.00
7.5 7.0 6.5 6.0 5.5 5.0 4.5 4.0 3.5 3.0 2.5 2.0 1.5 1.0 0.5
f1 (ppm)

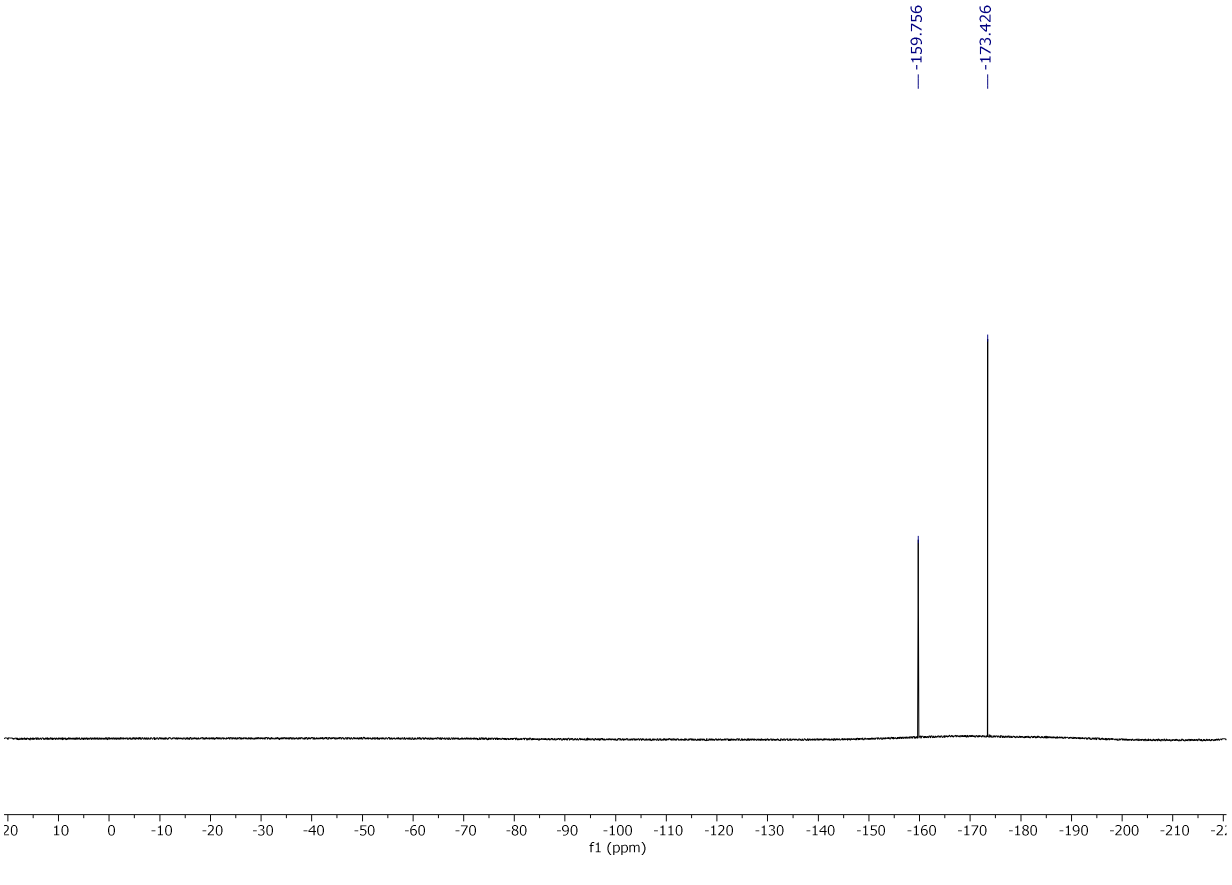

-159.756
-173.426
10 0 -10 -20 -30 -40 -50 -60 -70 -80 -90 -100 -110 -120 -130 -140 -150 -160 -170 -180 -190 -200 -210
f1 (ppm)

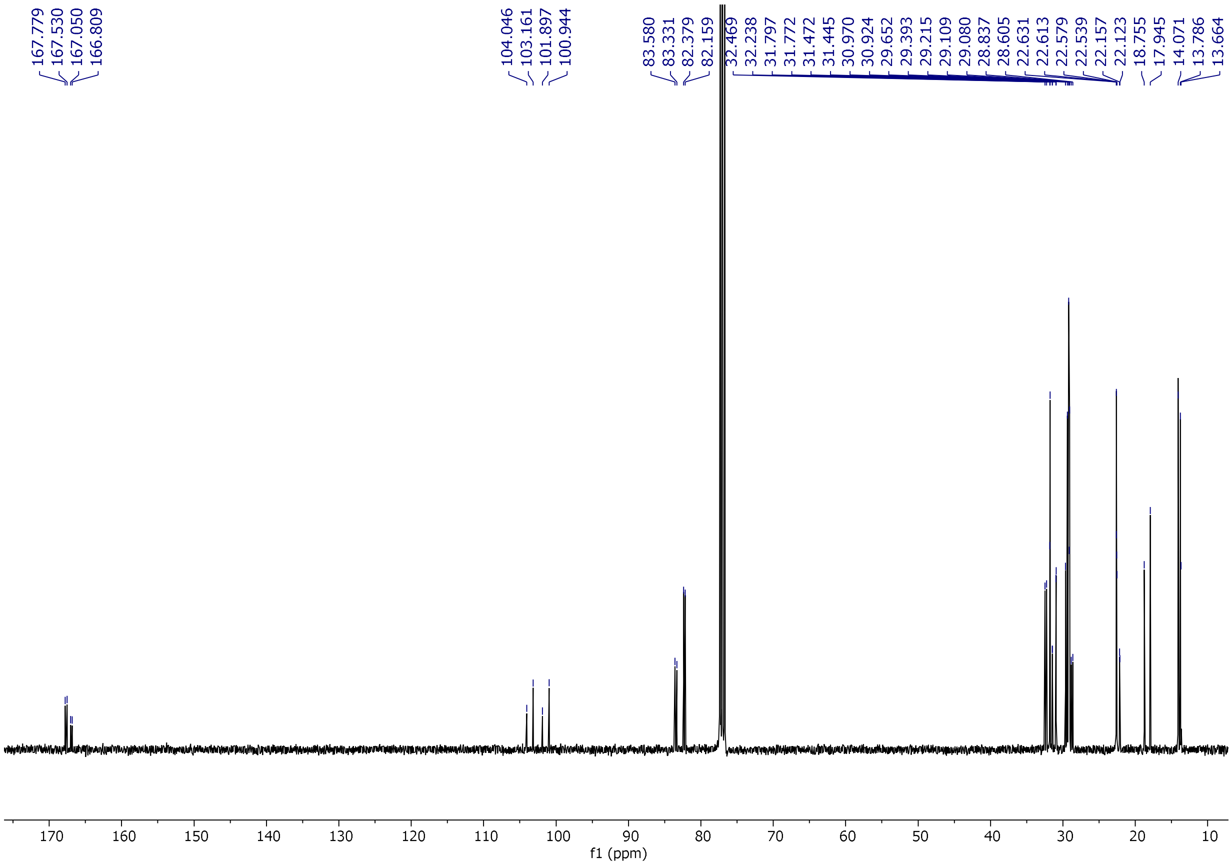
167.779
167.530
167.050
166.809
104.046
103.161
101.897
100.944
83.580
83.331
82.379
82.159
32.469
32.238
31.797
31.772
31.472
31.445
30.970
30.924
29.652
29.393
29.215
29.109
29.080
28.837
28.605
22.631
22.613
22.579
22.539
22.157
22.123
18.755
17.945
14.071
13.786
13.664
170 160 150 140 130 120 110 100 90 80 70 60 50 40 30 20 10
f1 (ppm)

VM044

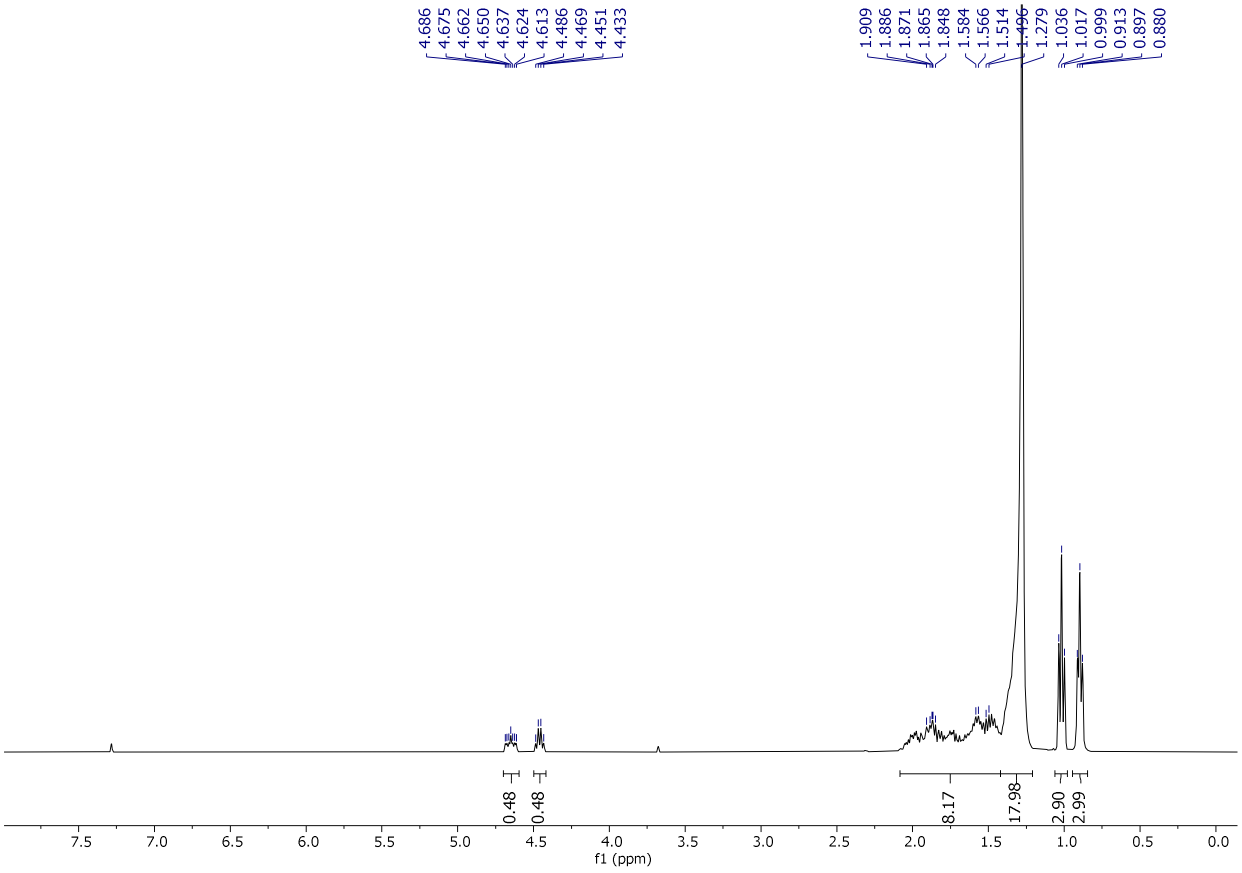
4.686
4.675
4.662
4.650
4.637
4.624
4.613
4.486
4.469
4.451
4.433
1.909
1.886
1.871
1.865
1.848
1.584
1.566
1.514
1.496
1.279
1.036
1.017
0.999
0.913
0.897
0.880
0.48
0.48
8.17
17.98
2.90
2.99
7.5
7.0
6.5
6.0
5.5
5.0
4.5
4.0
3.5
3.0
2.5
2.0
1.5
1.0
0.5
0.0
f1 (ppm)

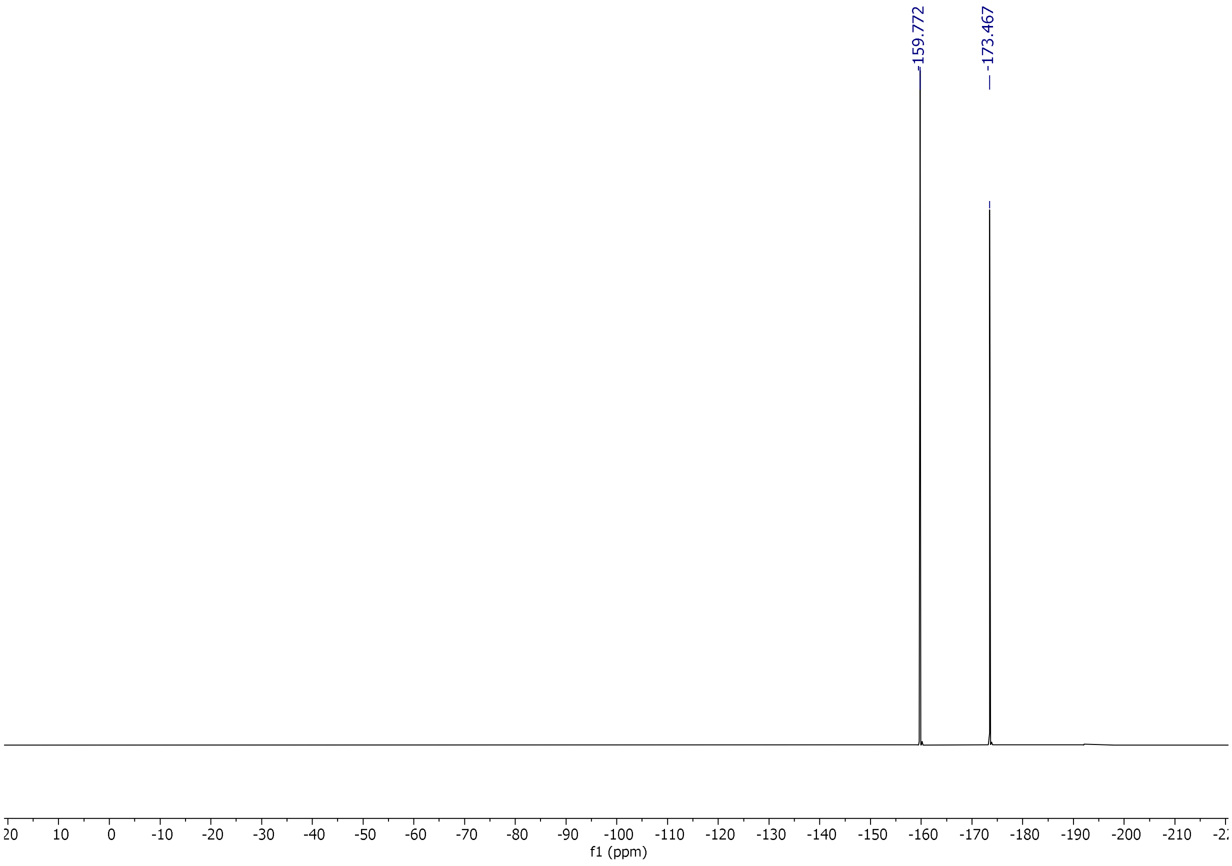
-159.772
-173.467
20
10
0
-10
-20
-30
-40
-50
-60
-70
-80
-90
-100
-110
-120
-130
-140
-150
-160
-170
-180
-190
-200
-210
f1 (ppm)

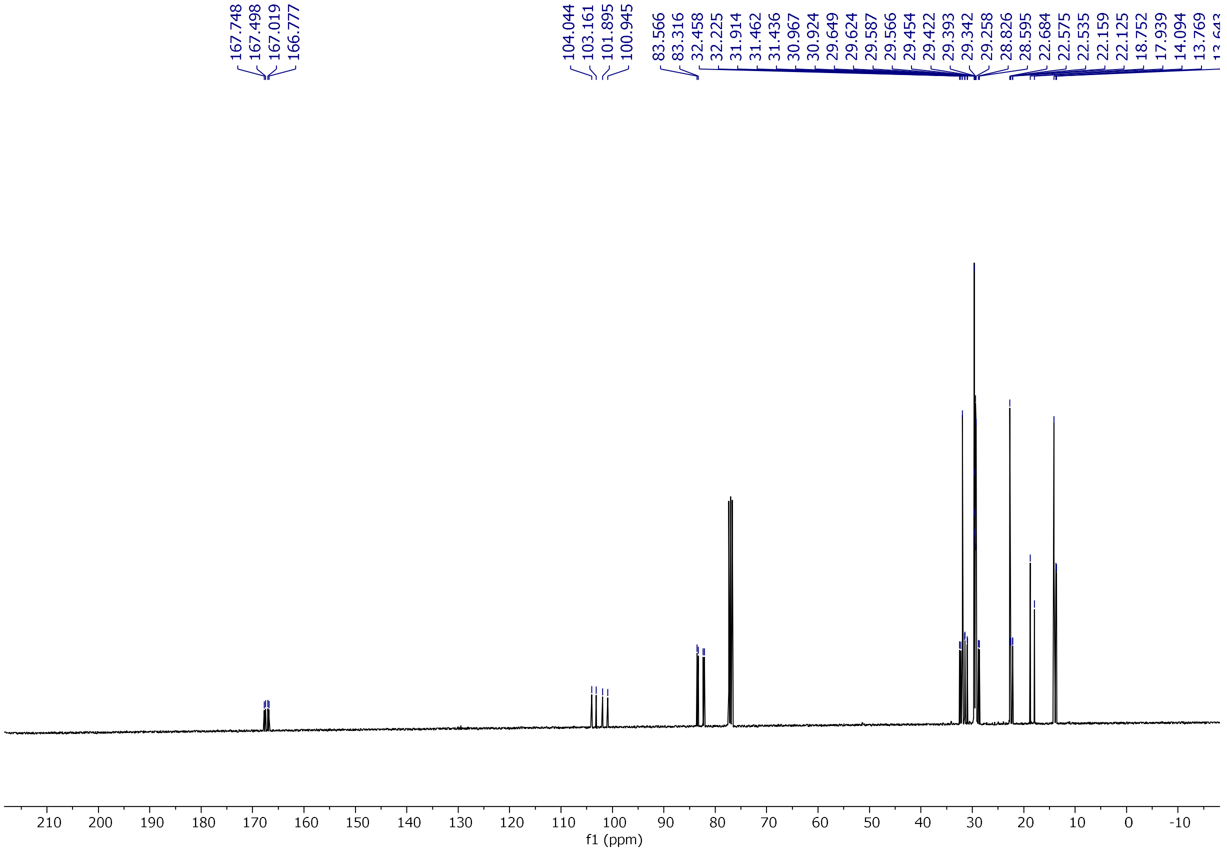
167.748
167.498
167.019
166.777
104.044
103.161
101.895
100.945
83.566
83.316
32.458
32.225
31.914
31.462
31.436
30.967
30.924
29.649
29.624
29.587
29.566
29.454
29.422
29.393
29.342
29.258
28.826
28.595
22.684
22.575
22.535
22.159
22.125
18.752
17.939
14.094
13.769
210
200
190
180
170
160
150
140
130
120
110
100
90
80
70
60
50
40
30
20
10
0
-10
f1 (ppm)

VM045

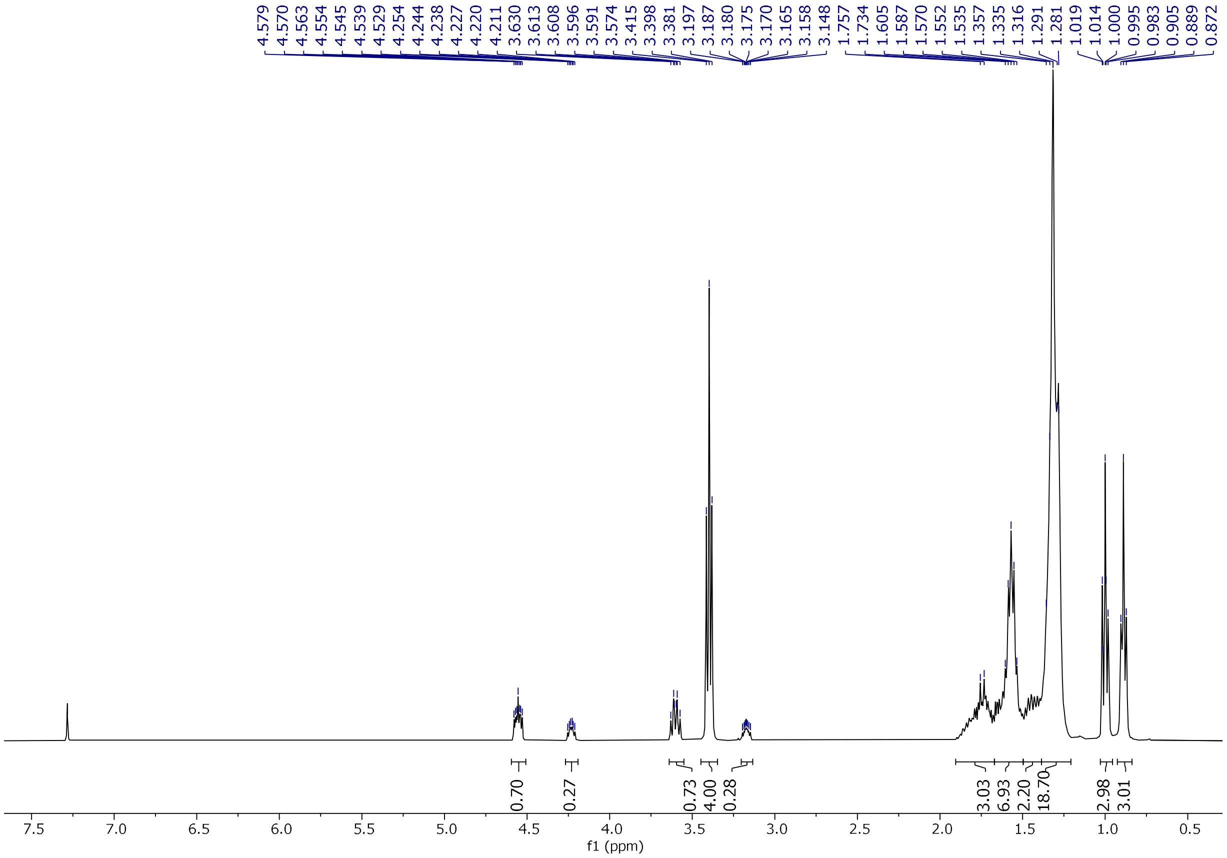
4.579
4.570
4.563
4.554
4.545
4.539
4.529
4.254
4.244
4.238
4.227
4.220
4.211
3.630
3.613
3.608
3.596
3.591
3.574
3.415
3.398
3.381
3.197
3.187
3.180
3.175
3.170
3.165
3.158
3.148
1.757
1.734
1.605
1.587
1.570
1.552
1.535
1.357
1.335
1.316
1.291
1.281
1.019
1.014
1.000
0.995
0.983
0.905
0.889
0.872
0.70
0.27
0.73
4.00
0.28
3.03
6.93
2.20
18.70
2.98
3.01
f1 (ppm)

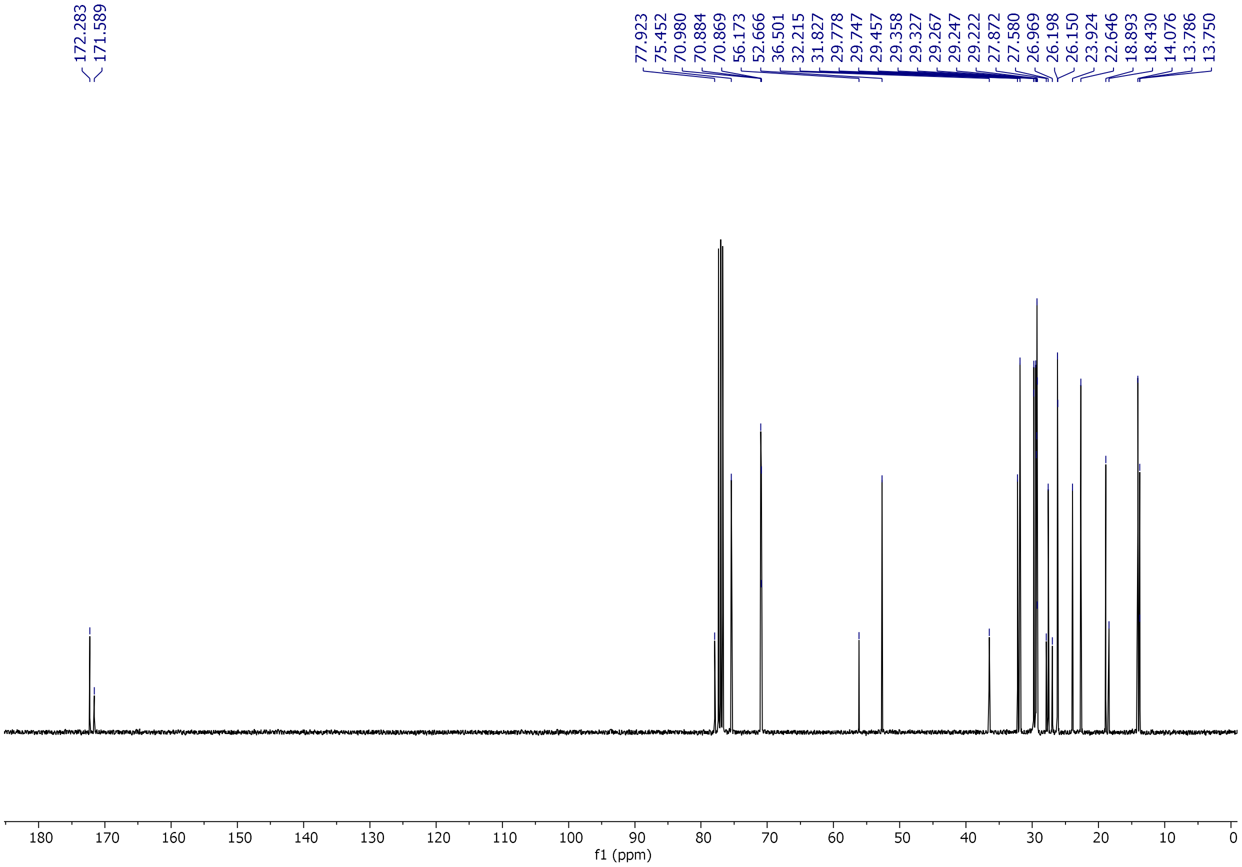
172.283
171.589
77.923
75.452
70.980
70.884
70.869
56.173
52.666
36.501
32.215
31.827
29.778
29.747
29.457
29.358
29.327
29.267
29.247
29.222
27.872
27.580
26.969
26.198
26.150
23.924
22.646
18.893
18.430
14.076
13.786
13.750
f1 (ppm)

VM046

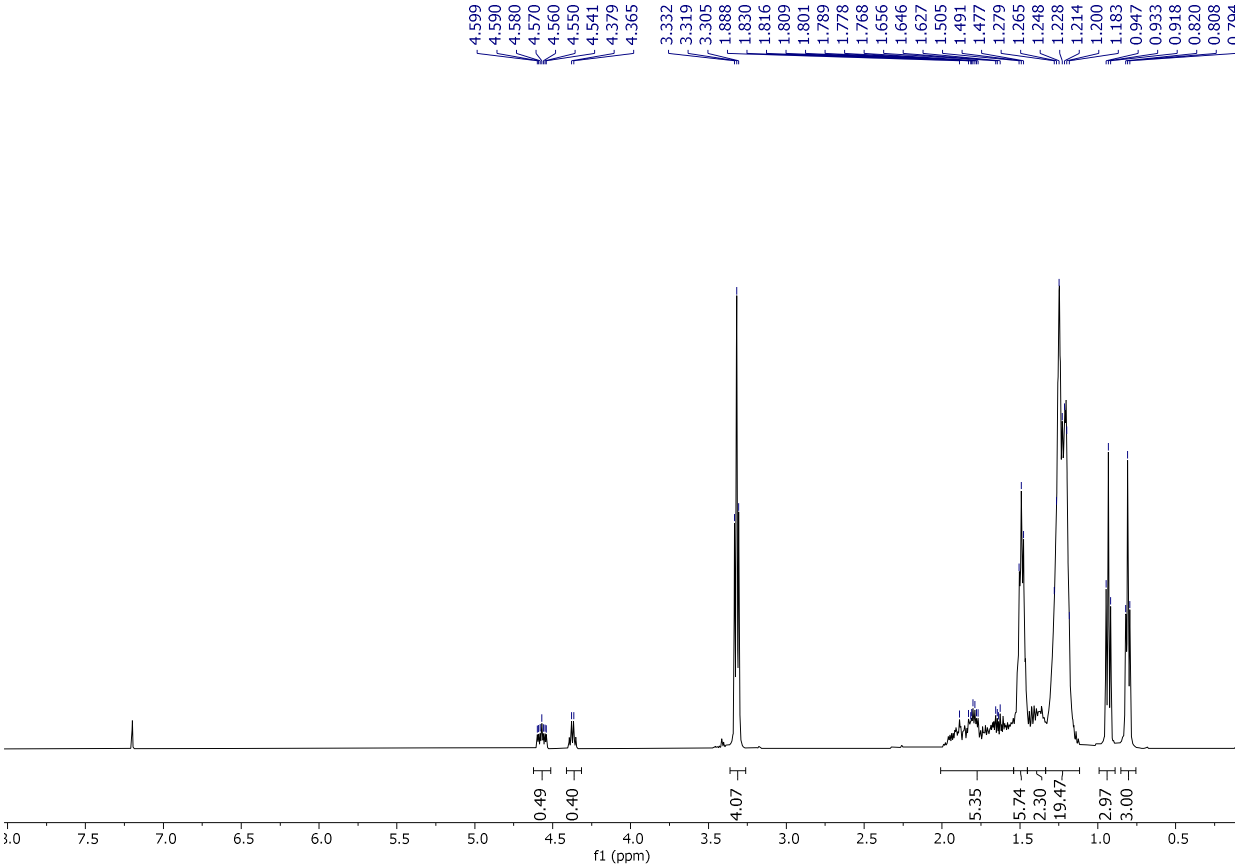

4.599
4.590
4.580
4.570
4.560
4.550
4.541
4.379
4.365
3.332
3.319
3.305
1.888
1.830
1.816
1.809
1.801
1.789
1.778
1.768
1.656
1.646
1.627
1.505
1.491
1.477
1.279
1.265
1.248
1.228
1.214
1.200
1.183
0.947
0.933
0.918
0.820
0.808
0.49
0.40
4.07
5.35
5.74
2.30
19.47
2.97
3.00
7.5
7.0
6.5
6.0
5.5
5.0
4.5
4.0
3.5
3.0
2.5
2.0
1.5
1.0
0.5
f1 (ppm)

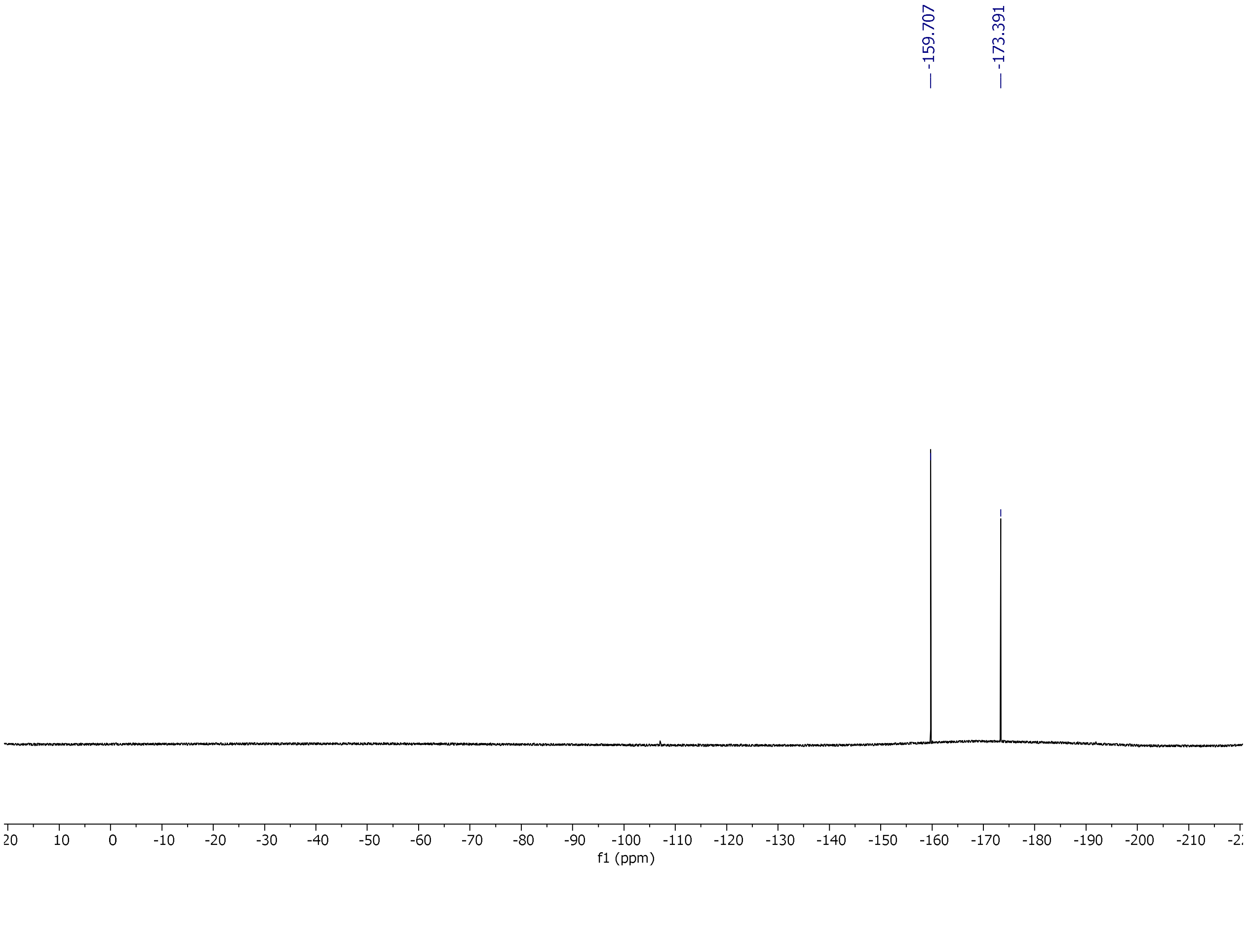

-159.707
-173.391
10
0
-10
-20
-30
-40
-50
-60
-70
-80
-90
-100
-110
-120
-130
-140
-150
-160
-170
-180
-190
-200
-210
f1 (ppm)

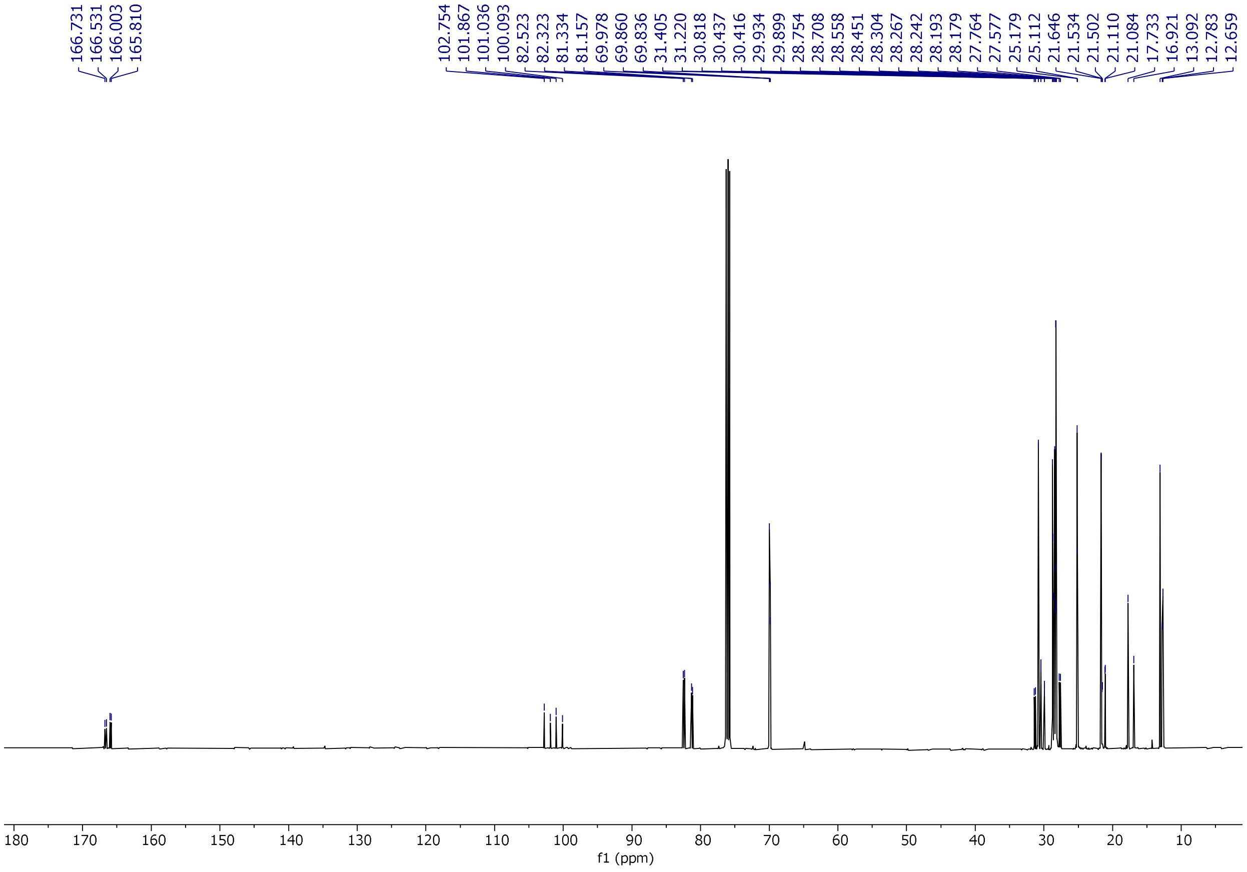
166.731
166.531
166.003
165.810
102.754
101.867
101.036
100.093
82.523
82.323
81.334
81.157
69.978
69.860
69.836
31.405
31.220
30.818
30.437
30.416
29.934
29.899
28.754
28.708
28.558
28.451
28.304
28.267
28.242
28.193
28.179
27.764
27.577
25.179
25.112
21.646
21.534
21.502
21.110
21.084
17.733
16.921
13.092
12.783
12.659
180
170
160
150
140
130
120
110
100
90
80
70
60
50
40
30
20
10
f1 (ppm)

VM047

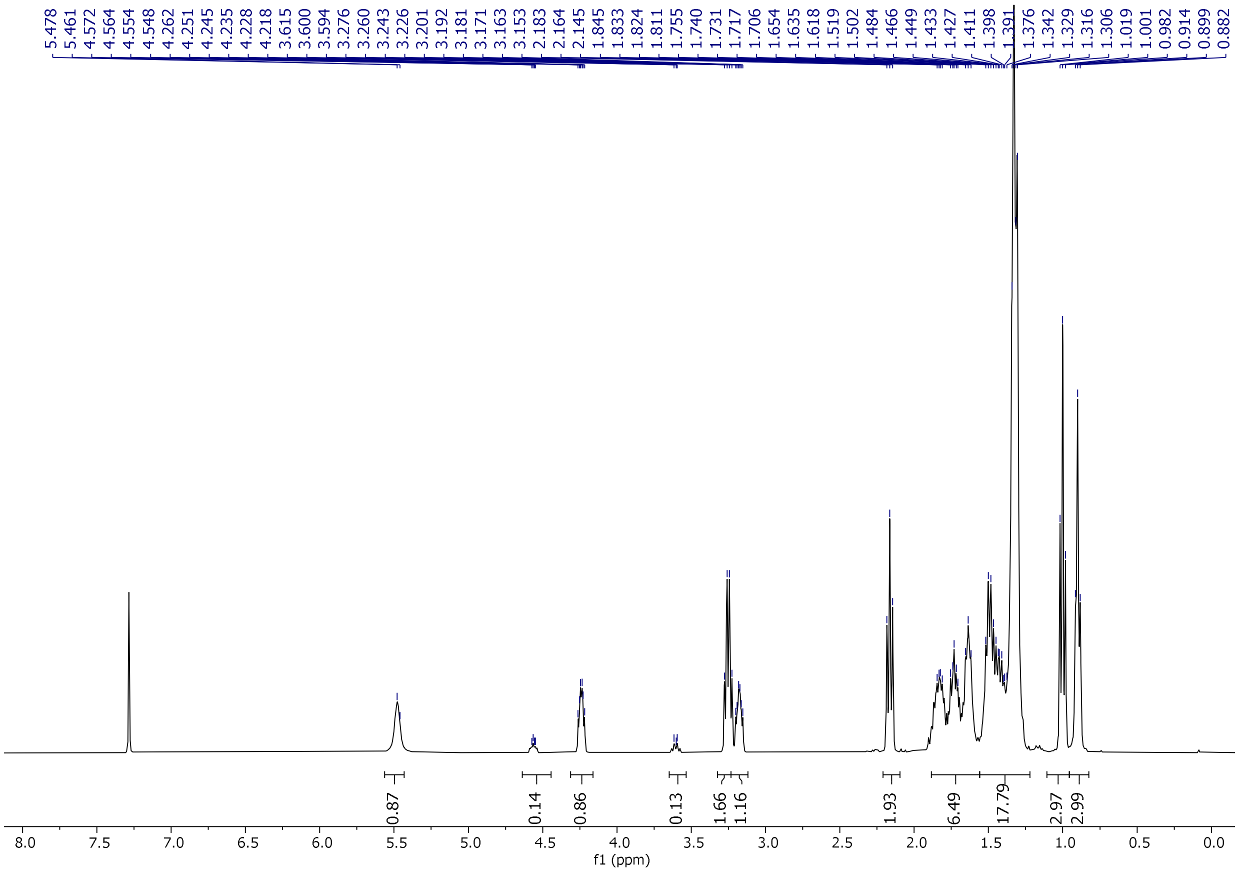
5.478
5.461
4.572
4.564
4.554
4.548
4.262
4.251
4.245
4.235
4.228
4.218
3.615
3.600
3.594
3.276
3.260
3.243
3.226
3.201
3.192
3.181
3.171
3.163
3.153
2.183
2.164
2.145
1.845
1.833
1.824
1.811
1.755
1.740
1.731
1.717
1.706
1.654
1.635
1.618
1.519
1.502
1.484
1.466
1.449
1.433
1.427
1.411
1.398
1.391
1.376
1.342
1.329
1.316
1.306
1.019
1.001
0.982
0.914
0.899
0.882
0.87
0.14
0.86
0.13
1.66
1.16
1.93
6.49
17.79
2.97
2.99
8.0
7.5
7.0
6.5
6.0
5.5
5.0
4.5
4.0
3.5
3.0
2.5
2.0
1.5
1.0
0.5
0.0
f1 (ppm)

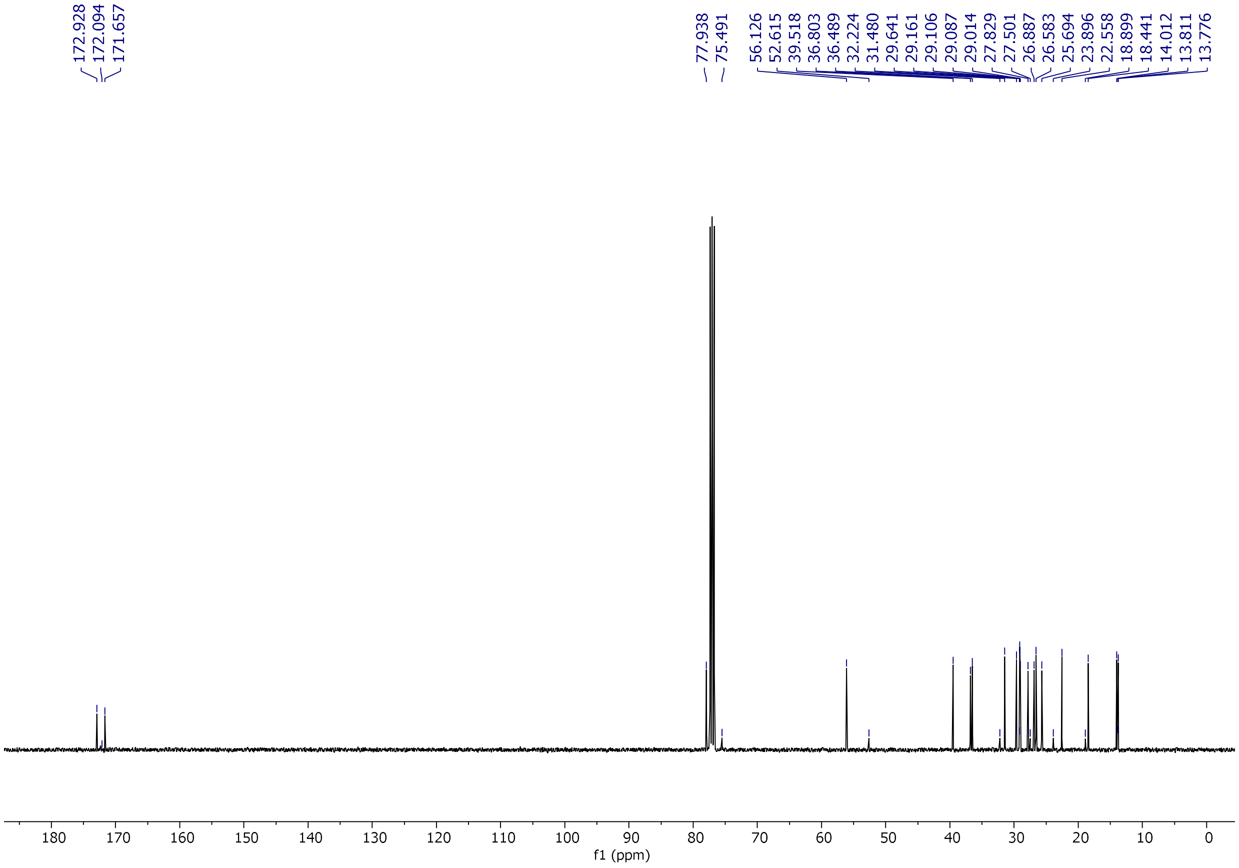
172.928
172.094
171.657
77.938
75.491
56.126
52.615
39.518
36.803
36.489
32.224
31.480
29.641
29.161
29.106
29.087
29.014
27.829
27.501
26.887
26.583
25.694
23.896
22.558
18.899
18.441
14.012
13.811
13.776
180
170
160
150
140
130
120
110
100
90
80
70
60
50
40
30
20
10
0
f1 (ppm)

VM048

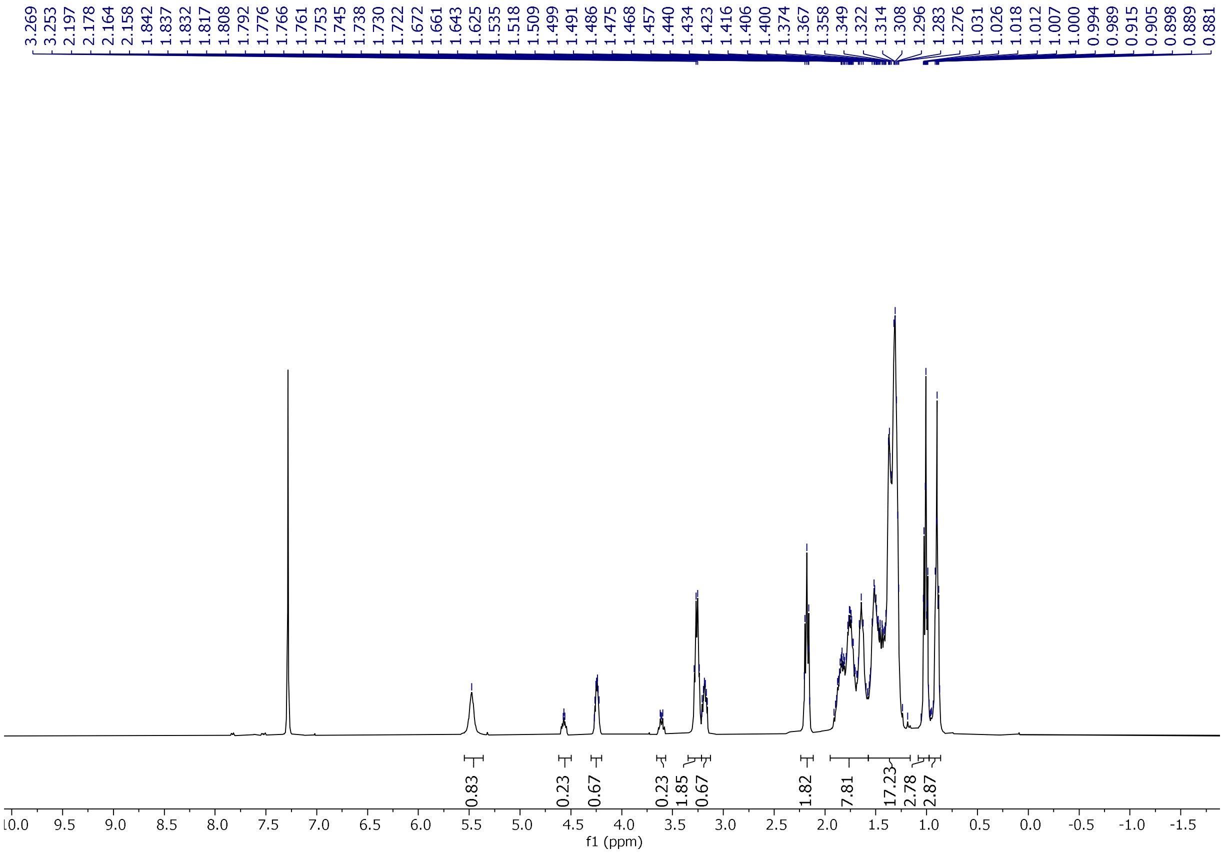

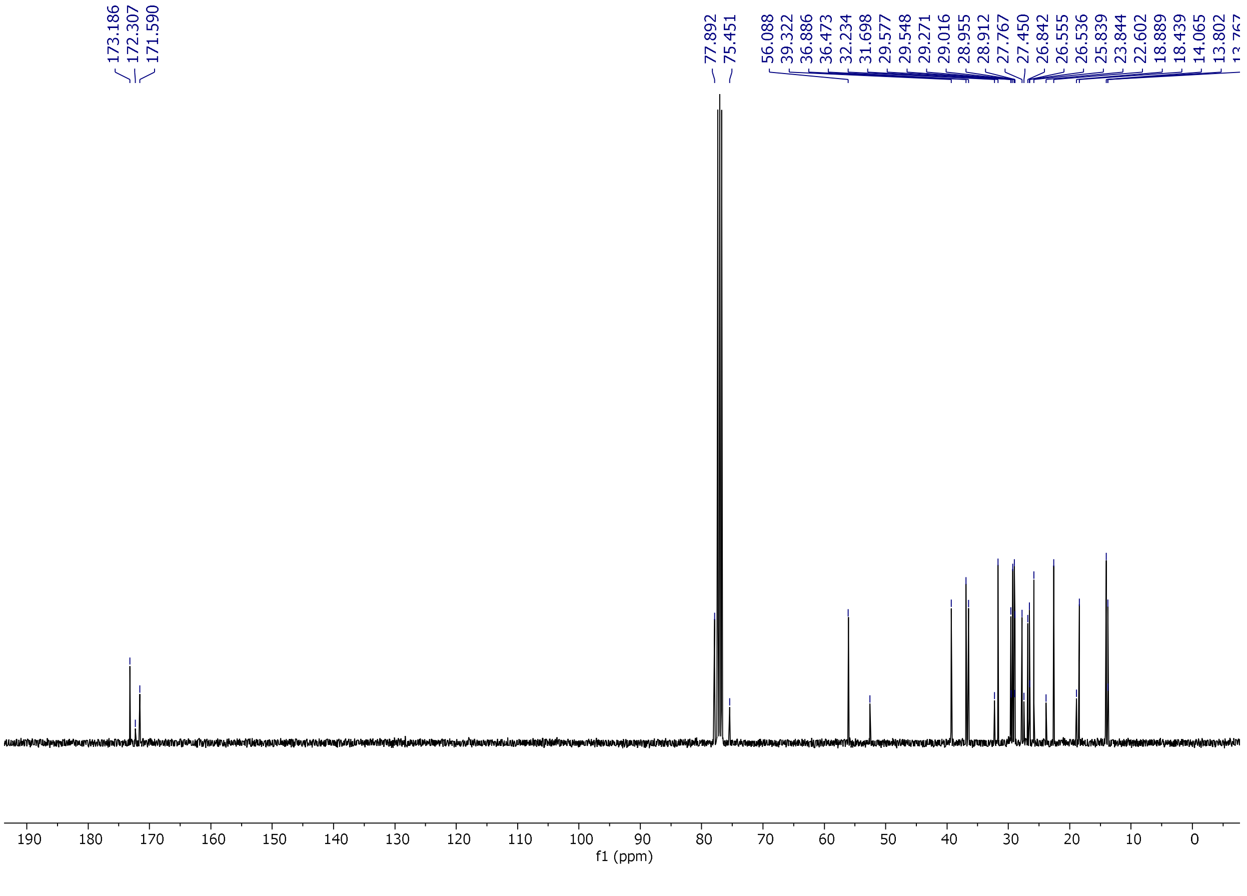

VM056

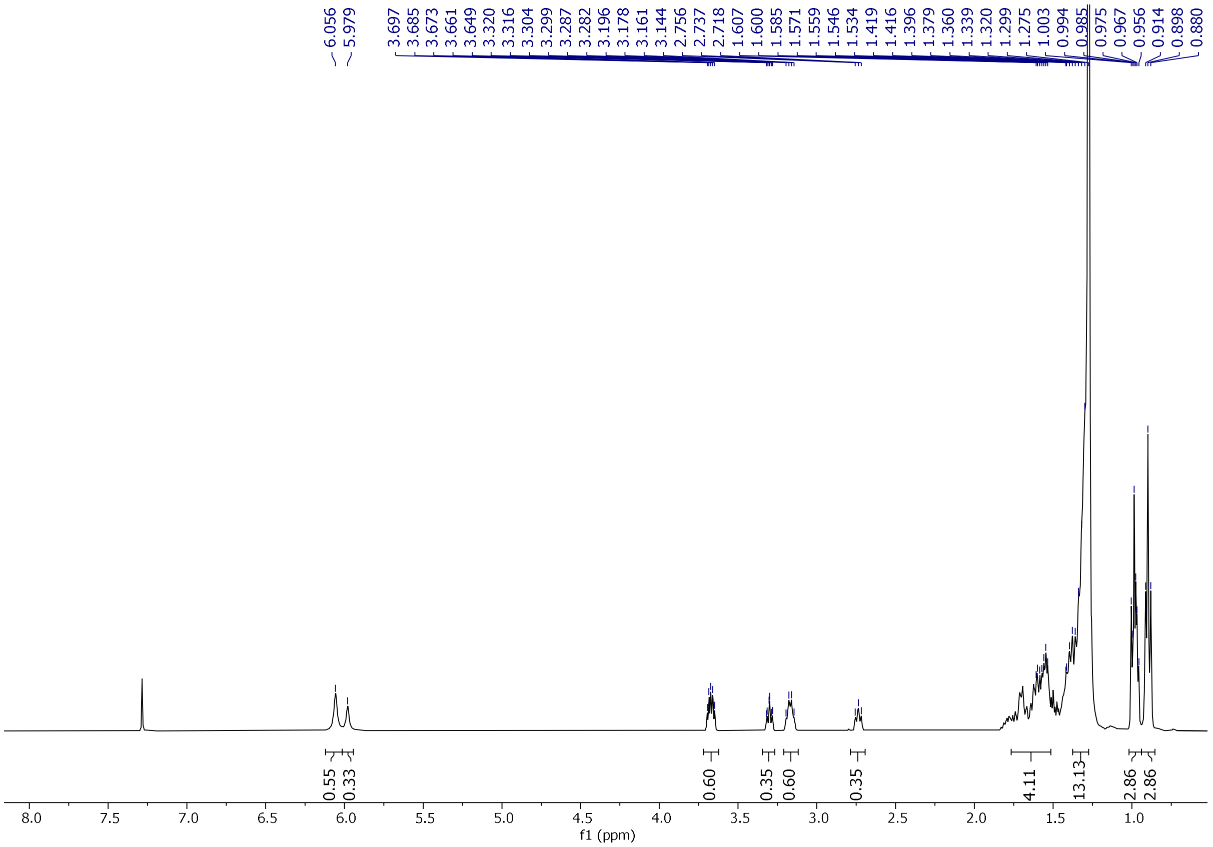

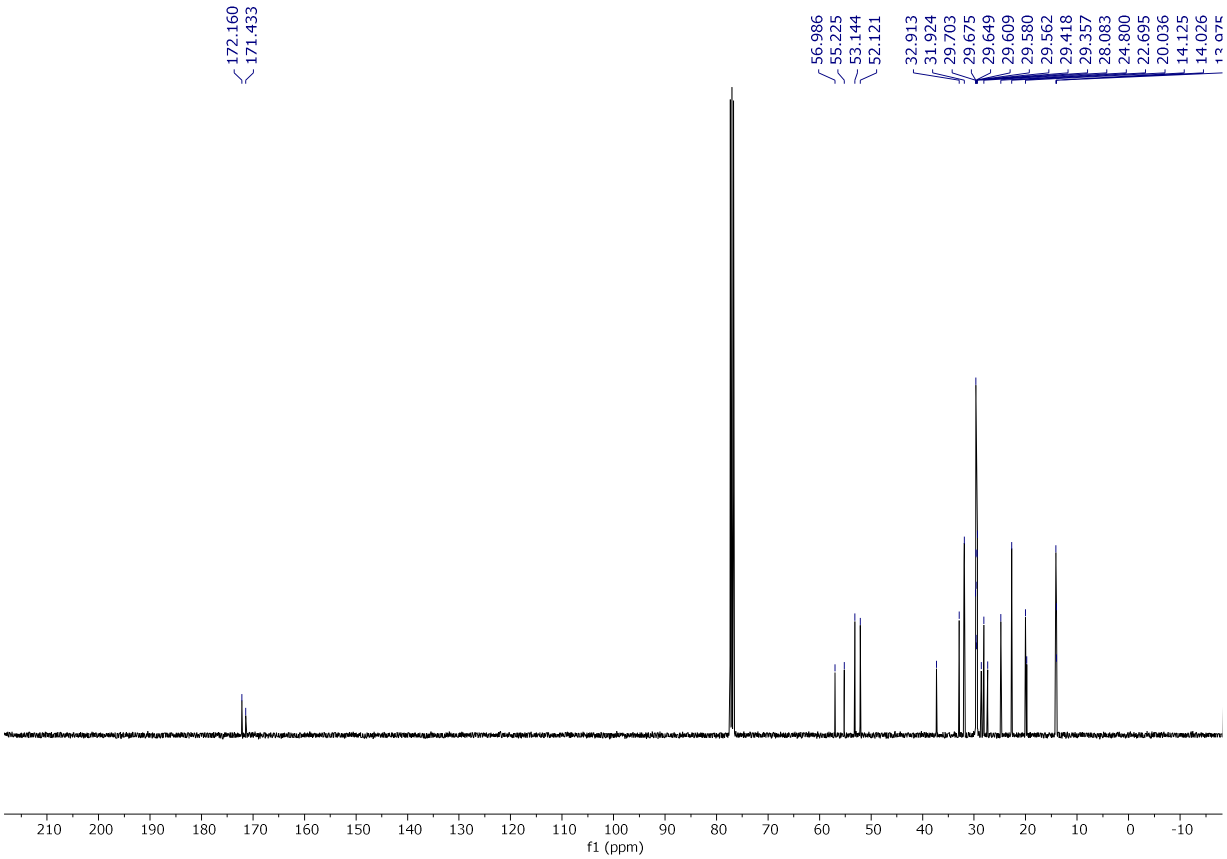

VM057

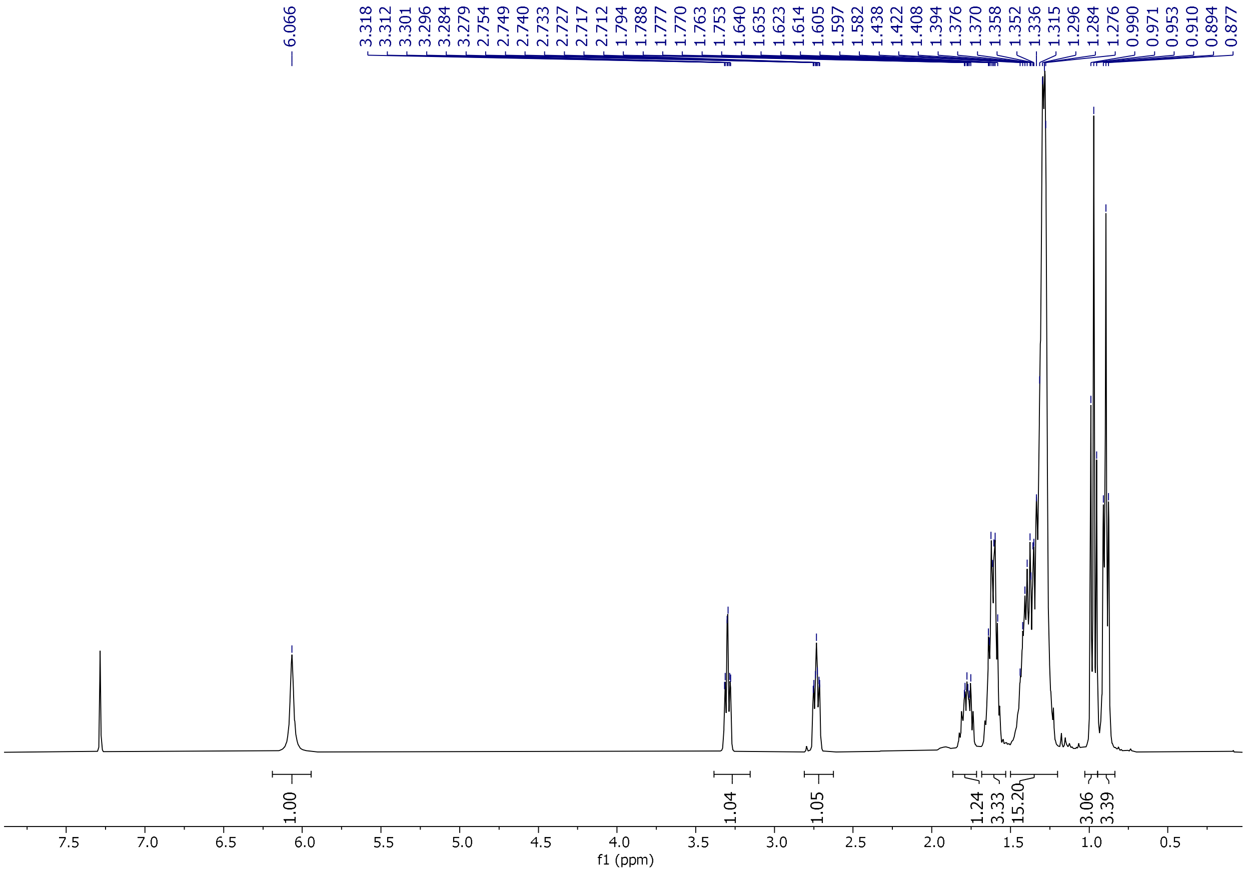
6.066
3.318 3.312 3.301 3.296 3.284 3.279 2.754 2.749 2.740 2.733 2.727 2.717 2.712 1.794 1.788 1.777 1.770 1.763 1.753 1.640 1.635 1.623 1.614 1.605 1.597 1.582 1.438 1.422 1.408 1.394 1.376 1.370 1.358 1.352 1.336 1.315 1.296 1.284 1.276 0.990 0.971 0.953 0.910 0.894 0.877
1.00
1.04
1.05
1.24
3.33
15.20
3.06
3.39
f1 (ppm)

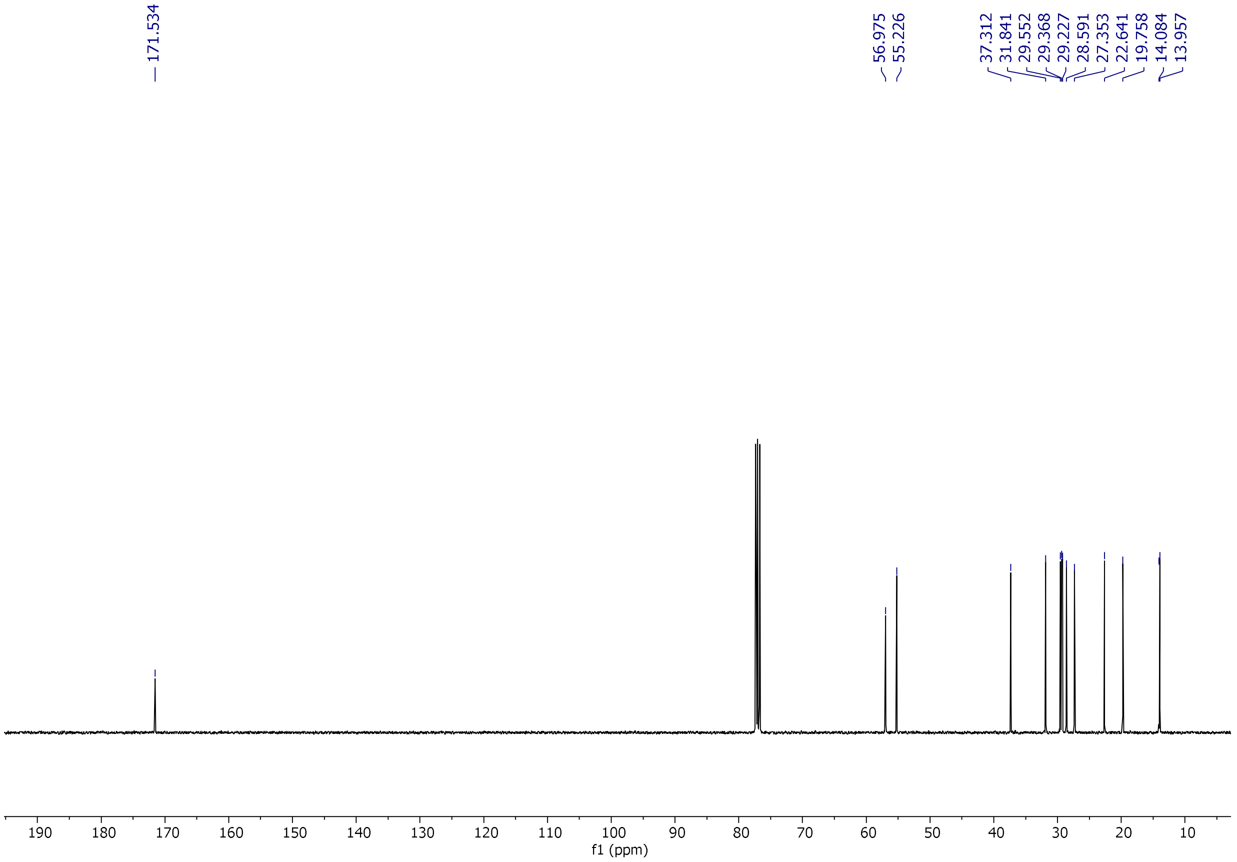
171.534
56.975
55.226
37.312 31.841 29.552 29.368 29.227 28.591 27.353 22.641 19.758 14.084 13.957
f1 (ppm)

VM058

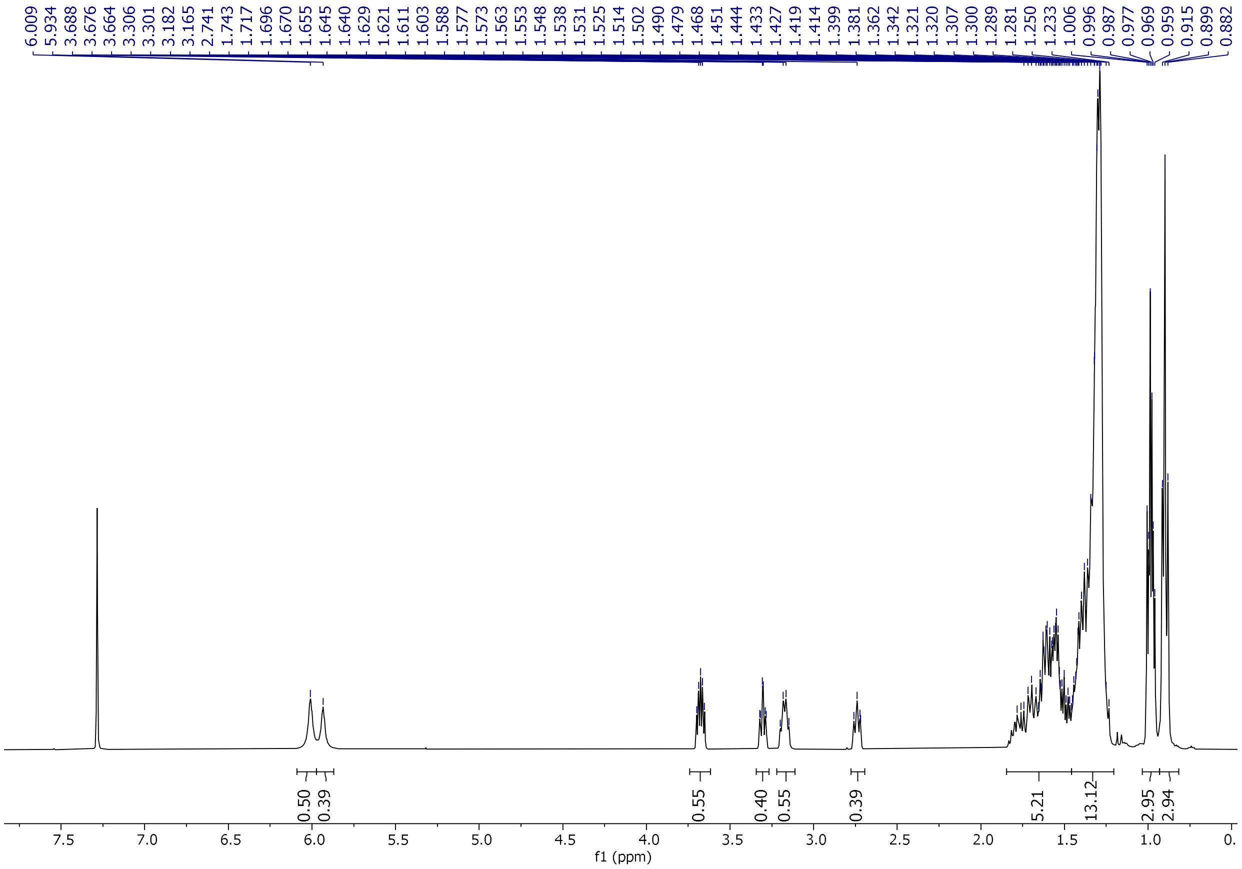
6.009
5.934
3.688
3.676
3.664
3.306
3.301
3.182
3.165
2.741
1.743
1.717
1.696
1.670
1.655
1.645
1.640
1.629
1.621
1.611
1.603
1.588
1.577
1.573
1.563
1.553
1.548
1.538
1.531
1.525
1.514
1.502
1.490
1.479
1.468
1.451
1.444
1.433
1.427
1.419
1.414
1.399
1.381
1.362
1.342
1.321
1.320
1.307
1.300
1.289
1.281
1.250
1.233
1.006
0.996
0.987
0.977
0.969
0.959
0.915
0.899
0.882
0.50
0.39
0.55
0.40
0.55
0.39
5.21
13.12
2.95
2.94
f1 (ppm)

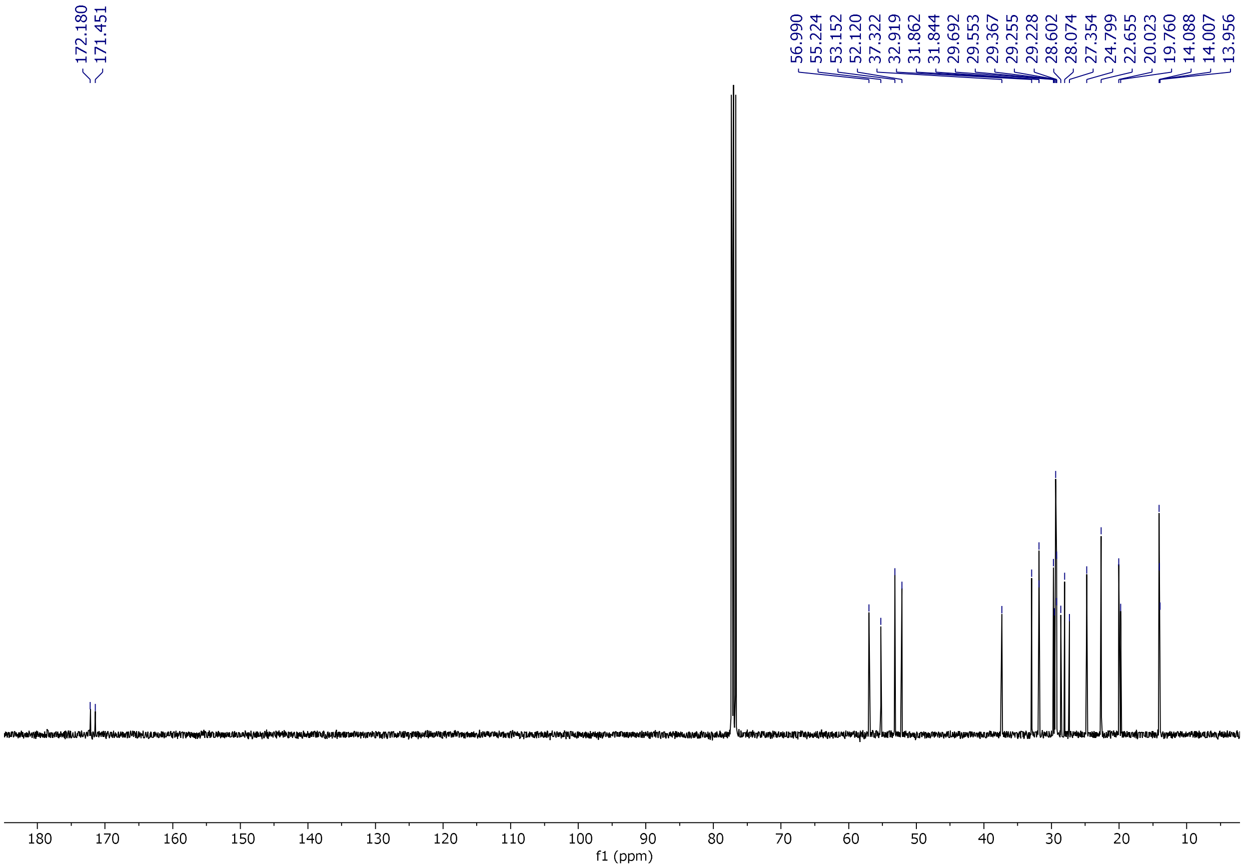
172.180
171.451
56.990
55.224
53.152
52.120
37.322
32.919
31.862
31.844
29.692
29.553
29.367
29.255
29.228
28.602
28.074
27.354
24.799
22.655
20.023
19.760
14.088
14.007
13.956
f1 (ppm)

^{1}H and ^{13}C NMR spectra of probes

VM035$_p$

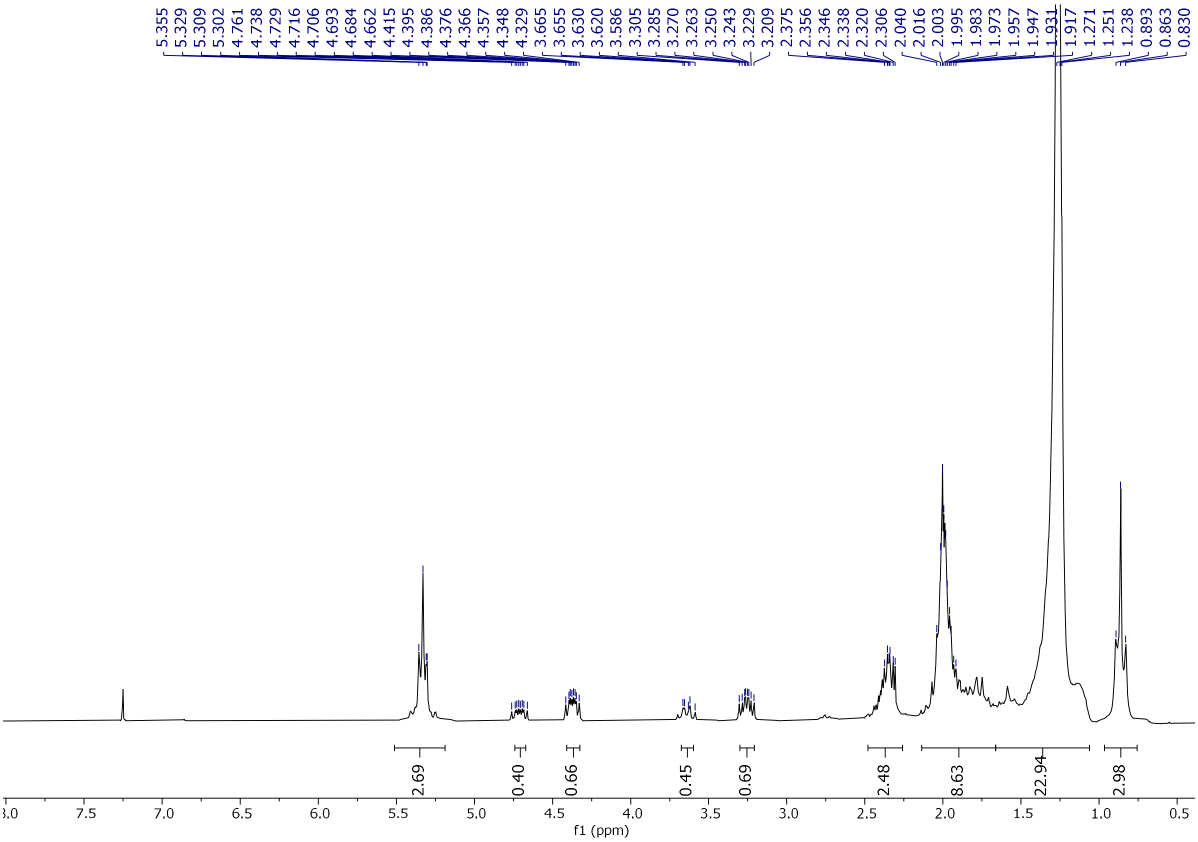

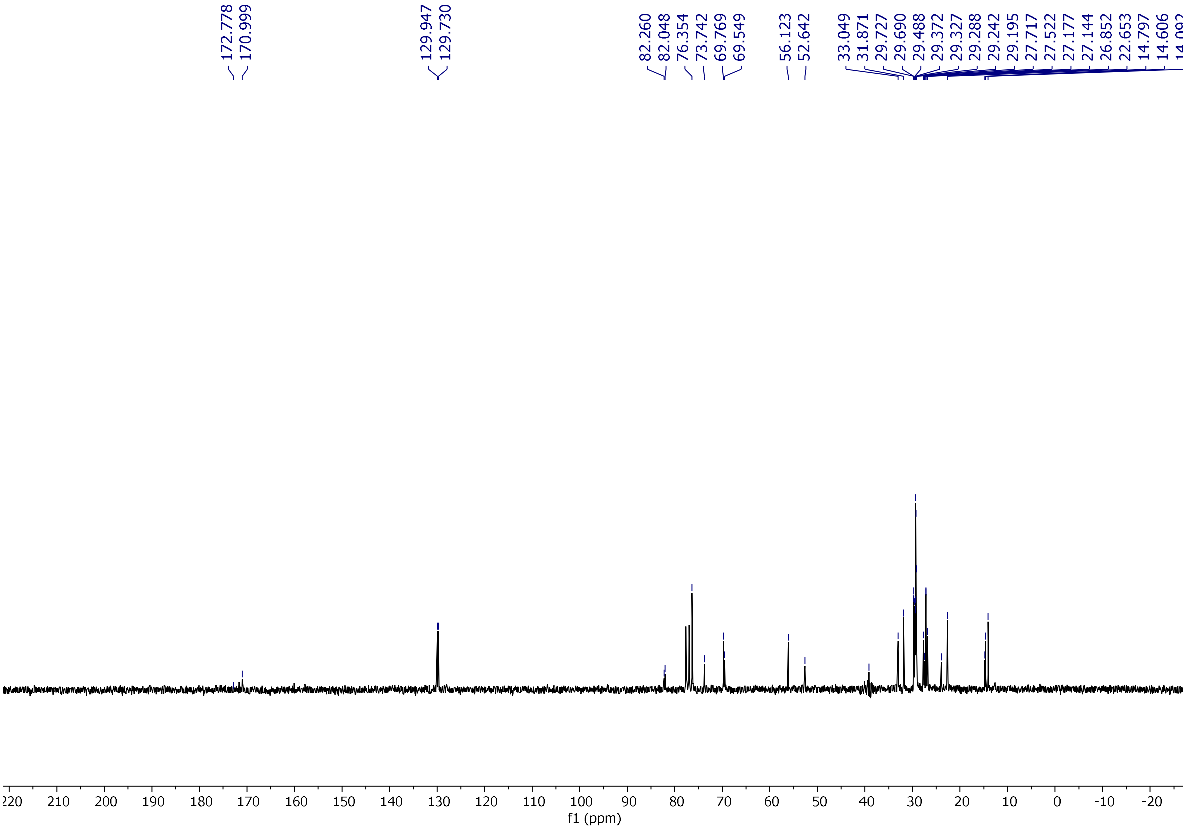

VM049*p*

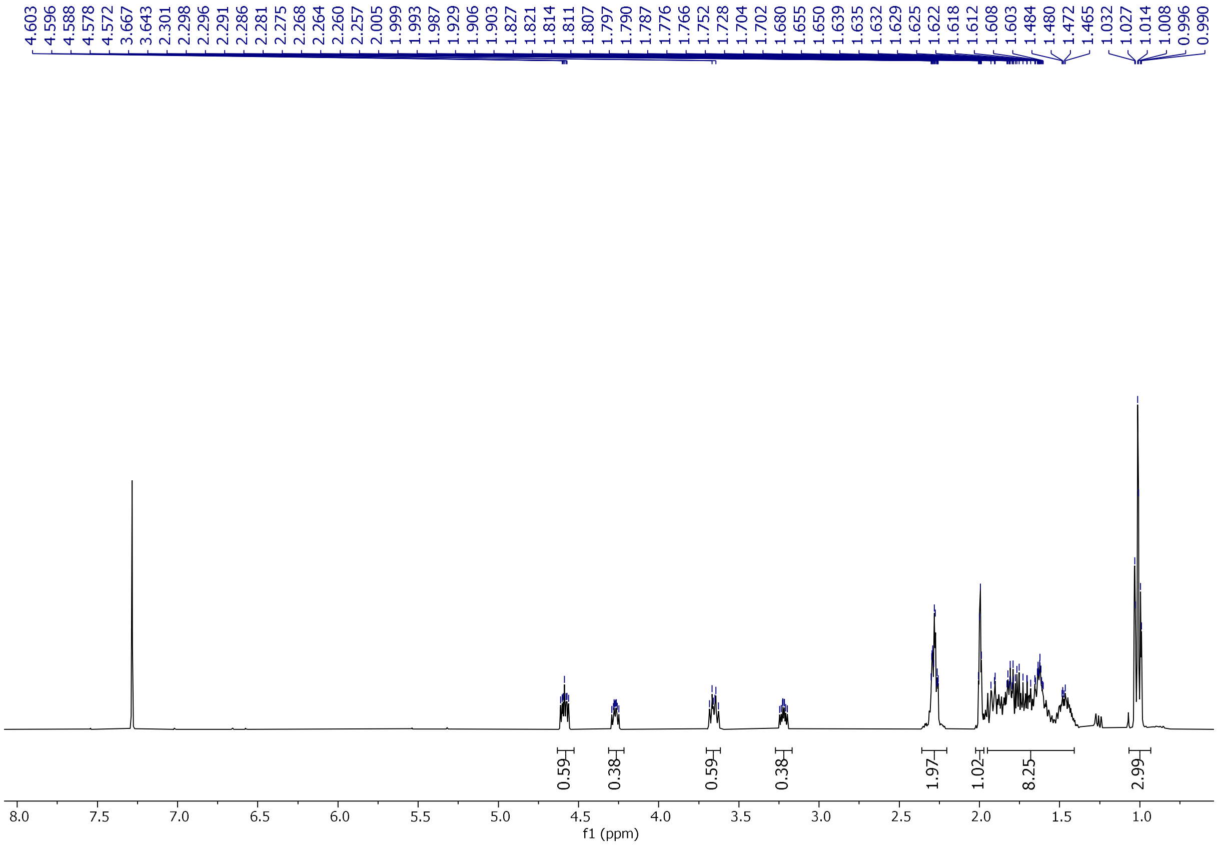
4.603
4.596
4.588
4.578
4.572
3.667
3.643
2.301
2.298
2.296
2.291
2.286
2.281
2.275
2.268
2.264
2.260
2.257
2.005
1.999
1.993
1.987
1.929
1.906
1.903
1.827
1.821
1.814
1.811
1.807
1.797
1.790
1.787
1.776
1.766
1.752
1.728
1.704
1.702
1.680
1.655
1.650
1.639
1.635
1.632
1.629
1.625
1.622
1.618
1.612
1.608
1.603
1.484
1.480
1.472
1.465
1.032
1.027
1.014
1.008
0.996
0.990
0.59
0.38
0.59
0.38
1.97
1.02
8.25
2.99
f1 (ppm)

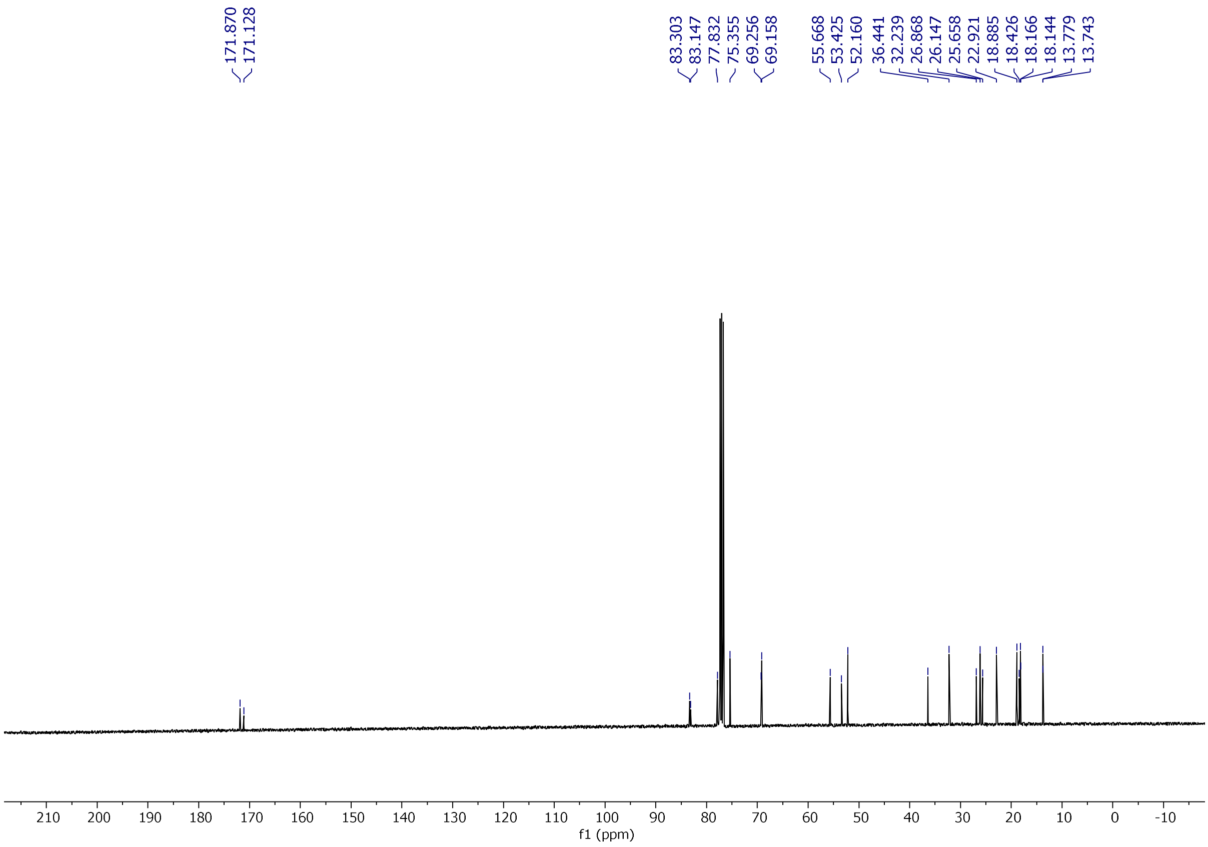
171.870
171.128
83.303
83.147
77.832
75.355
69.256
69.158
55.668
53.425
52.160
36.441
32.239
26.868
26.147
25.658
22.921
18.885
18.426
18.166
18.144
13.779
13.743
f1 (ppm)

VM050*p*

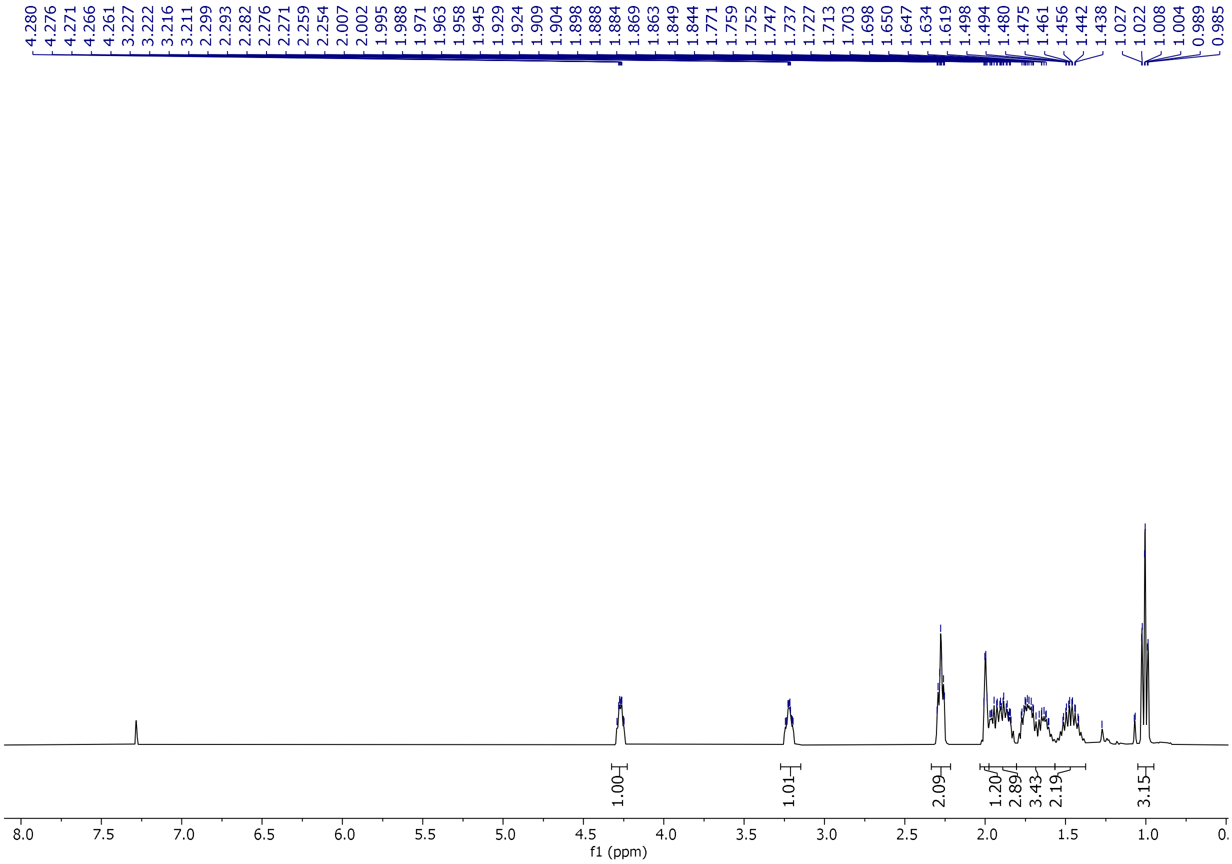
4.280
4.276
4.271
4.266
4.261
3.227
3.222
3.216
3.211
2.299
2.293
2.282
2.276
2.271
2.259
2.254
2.007
2.002
1.995
1.988
1.971
1.963
1.958
1.945
1.929
1.924
1.909
1.904
1.898
1.888
1.884
1.869
1.863
1.849
1.844
1.771
1.759
1.752
1.747
1.737
1.727
1.713
1.703
1.698
1.650
1.647
1.634
1.619
1.498
1.494
1.480
1.475
1.461
1.456
1.442
1.438
1.027
1.022
1.008
1.004
0.989
0.985
1.00
1.01
2.09
1.20
2.89
3.43
2.19
3.15
f1 (ppm)

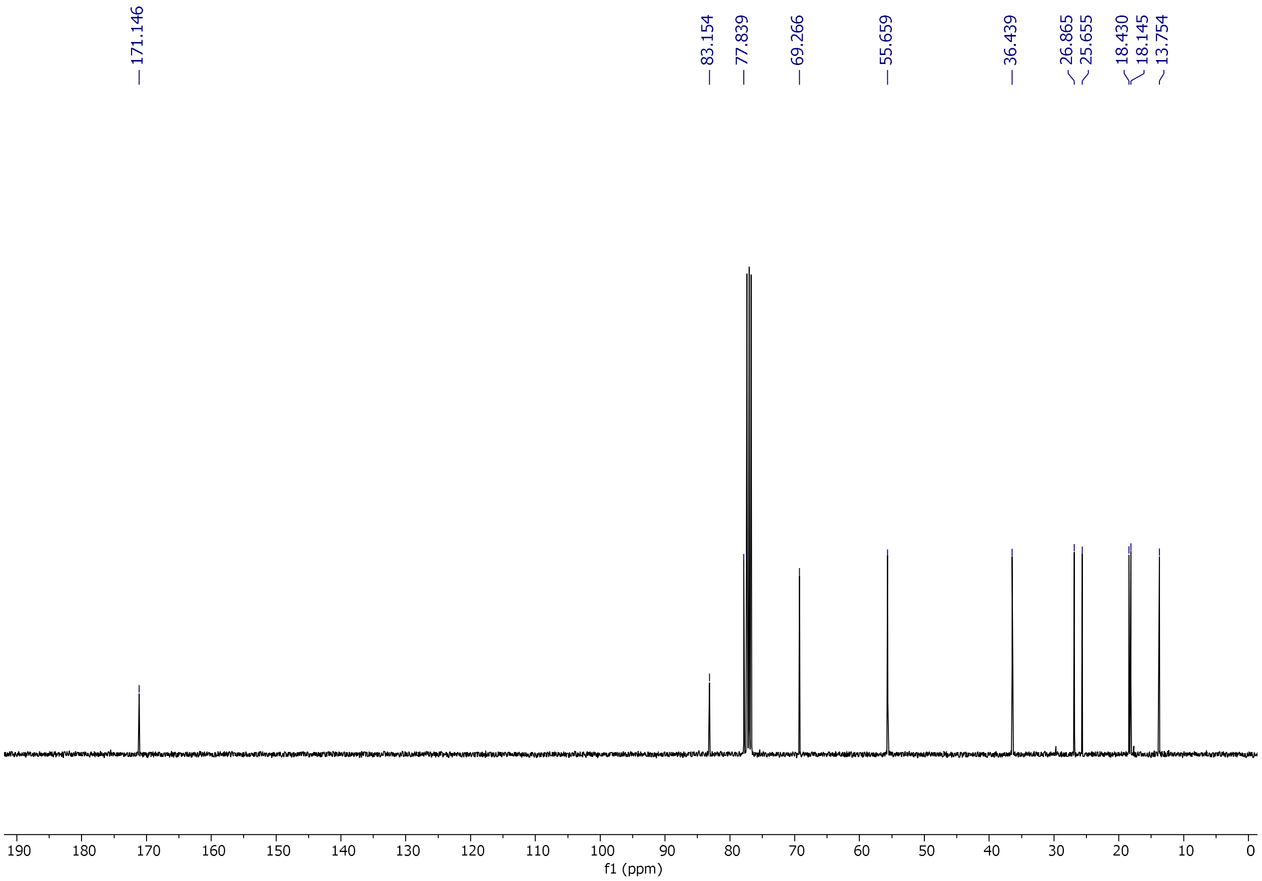
171.146
83.154
77.839
69.266
55.659
36.439
26.865
25.655
18.430
18.145
13.754
f1 (ppm)

VM051$_p$

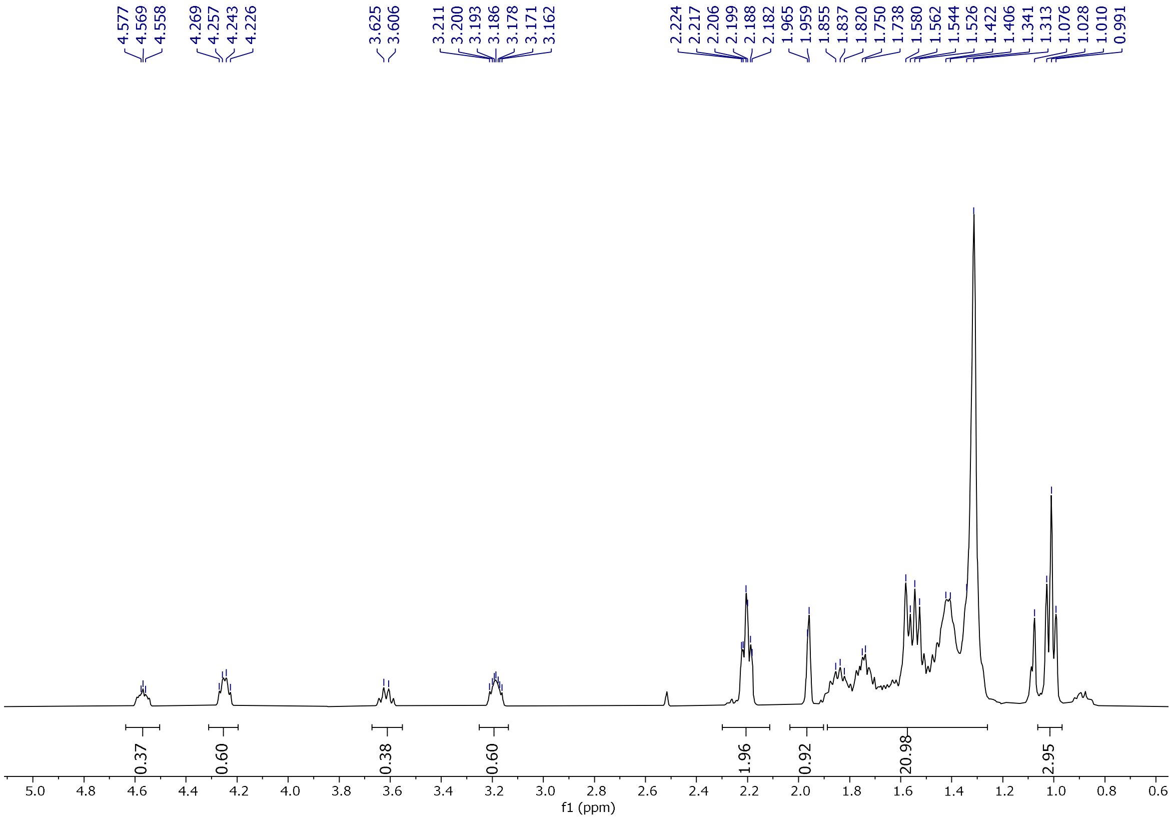
4.577
4.569
4.558
4.269
4.257
4.243
4.226
3.625
3.606
3.211
3.200
3.193
3.186
3.178
3.171
3.162
2.224
2.217
2.206
2.199
2.188
2.182
1.965
1.959
1.855
1.837
1.820
1.750
1.738
1.580
1.562
1.544
1.526
1.422
1.406
1.341
1.313
1.076
1.028
1.010
0.991
0.37
0.60
0.38
0.60
1.96
0.92
20.98
2.95
5.0 4.8 4.6 4.4 4.2 4.0 3.8 3.6 3.4 3.2 3.0 2.8 2.6 2.4 2.2 2.0 1.8 1.6 1.4 1.2 1.0 0.8 0.6
f1 (ppm)

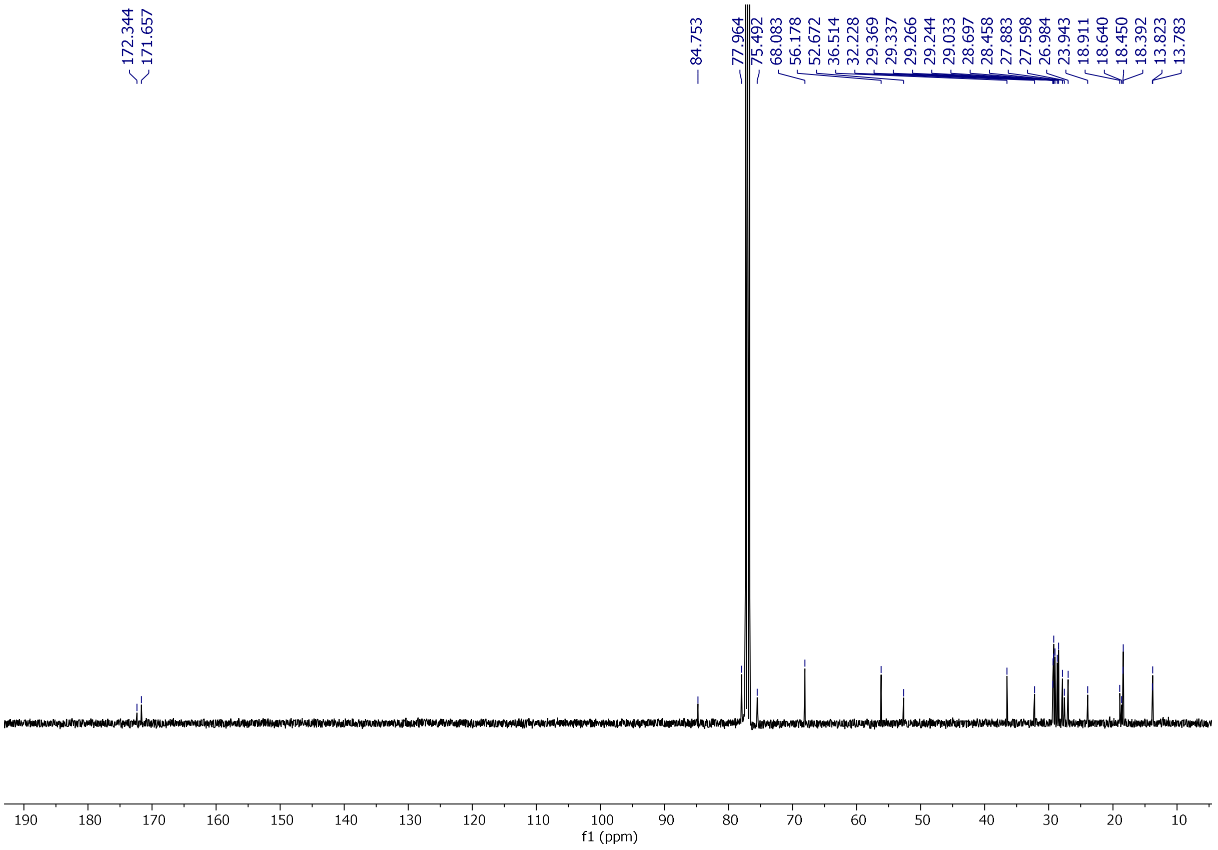
172.344
171.657
84.753
77.964
75.492
68.083
56.178
52.672
36.514
32.228
29.369
29.337
29.266
29.244
29.033
28.697
28.458
27.883
27.598
26.984
23.943
18.911
18.640
18.450
18.392
13.823
13.783
190 180 170 160 150 140 130 120 110 100 90 80 70 60 50 40 30 20 10
f1 (ppm)

VM052*p*

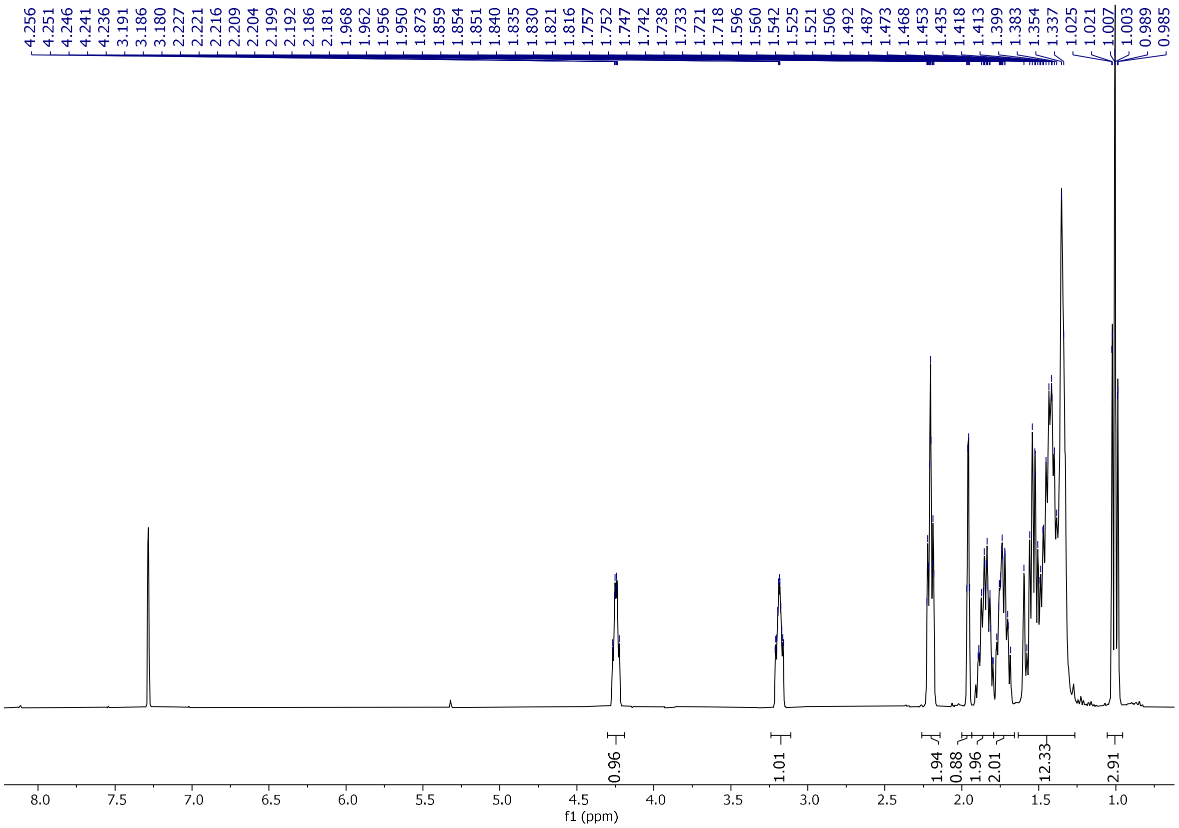
4.256 4.251 4.246 4.241 4.236 3.191 3.186 3.180 2.227 2.221 2.216 2.209 2.204 2.199 2.192 2.186 2.181 1.968 1.962 1.956 1.950 1.873 1.859 1.854 1.851 1.840 1.835 1.830 1.821 1.816 1.757 1.752 1.747 1.742 1.738 1.733 1.721 1.718 1.596 1.560 1.542 1.525 1.521 1.506 1.492 1.487 1.473 1.468 1.453 1.435 1.418 1.413 1.399 1.383 1.354 1.337 1.025 1.021 1.007 1.003 0.989 0.985
0.96
1.01
1.94
0.88
1.96
2.01
12.33
2.91
8.0 7.5 7.0 6.5 6.0 5.5 5.0 4.5 4.0 3.5 3.0 2.5 2.0 1.5 1.0
f1 (ppm)

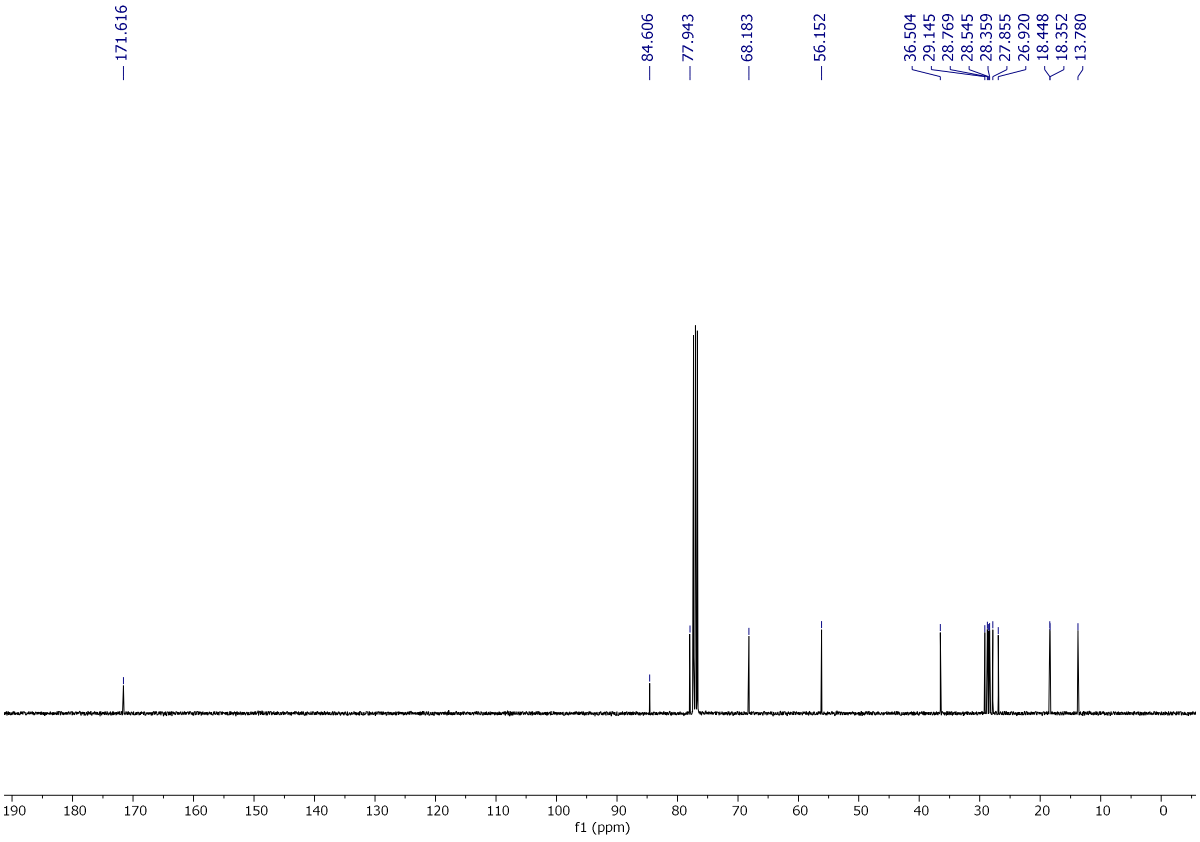
171.616
84.606
77.943
68.183
56.152
36.504 29.145 28.769 28.545 28.359 27.855 26.920 18.448 18.352 13.780
190 180 170 160 150 140 130 120 110 100 90 80 70 60 50 40 30 20 10 0
f1 (ppm)

VM053$_p$

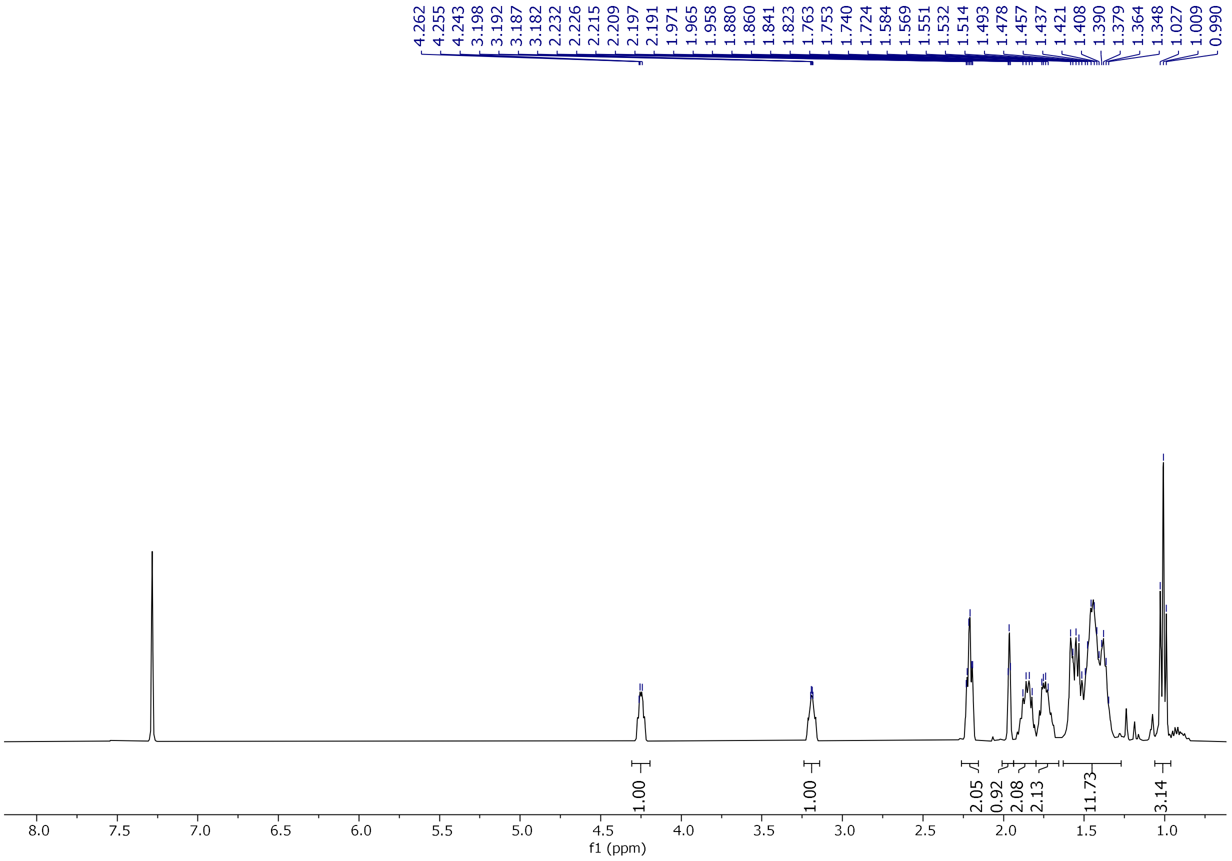
4.262
4.255
4.243
3.198
3.192
3.187
3.182
2.232
2.226
2.215
2.209
2.197
2.191
1.971
1.965
1.958
1.880
1.860
1.841
1.823
1.763
1.753
1.740
1.724
1.584
1.569
1.551
1.532
1.514
1.493
1.478
1.457
1.437
1.421
1.408
1.390
1.379
1.364
1.348
1.027
1.009
0.990
1.00
1.00
2.05
0.92
2.08
2.13
11.73
3.14
8.0
7.5
7.0
6.5
6.0
5.5
5.0
4.5
4.0
3.5
3.0
2.5
2.0
1.5
1.0
f1 (ppm)

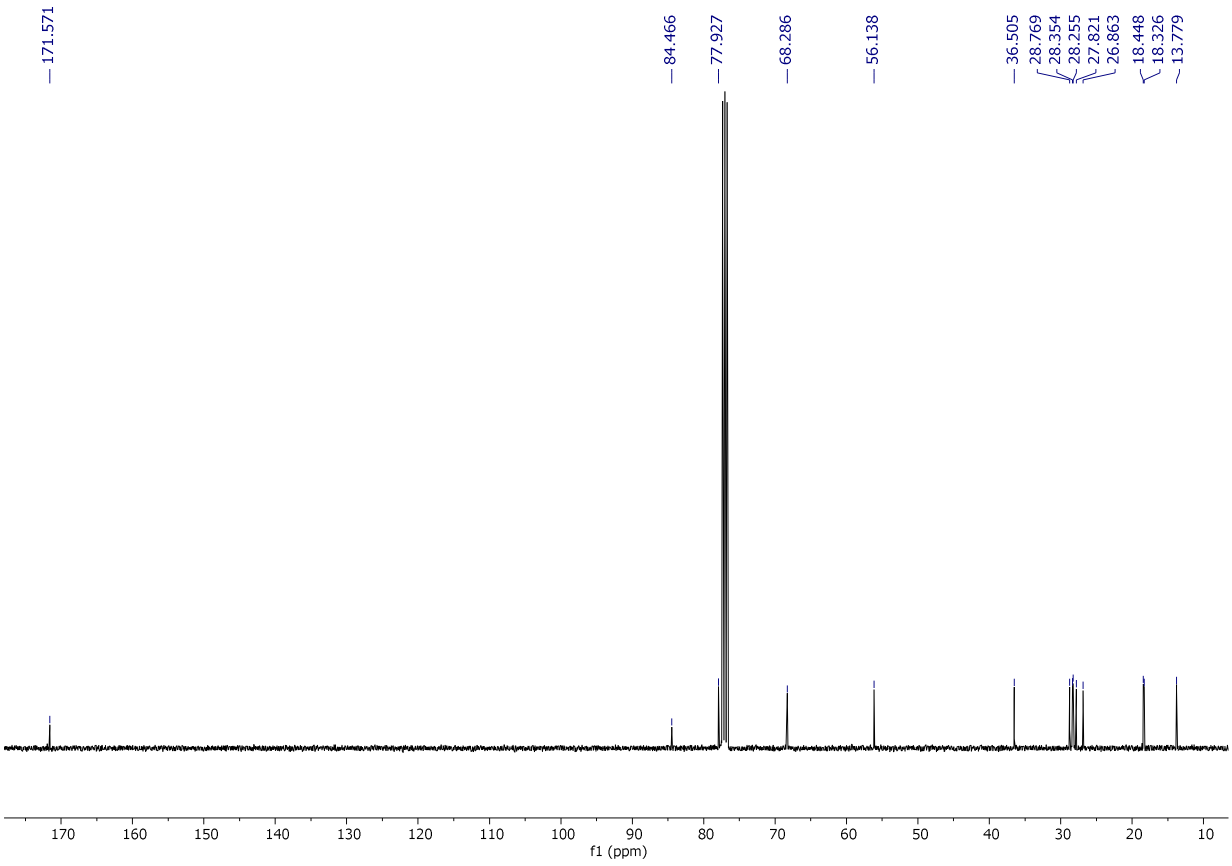
171.571
84.466
77.927
68.286
56.138
36.505
28.769
28.354
28.255
27.821
26.863
18.448
18.326
13.779
170
160
150
140
130
120
110
100
90
80
70
60
50
40
30
20
10
f1 (ppm)

VM054$_{p}$

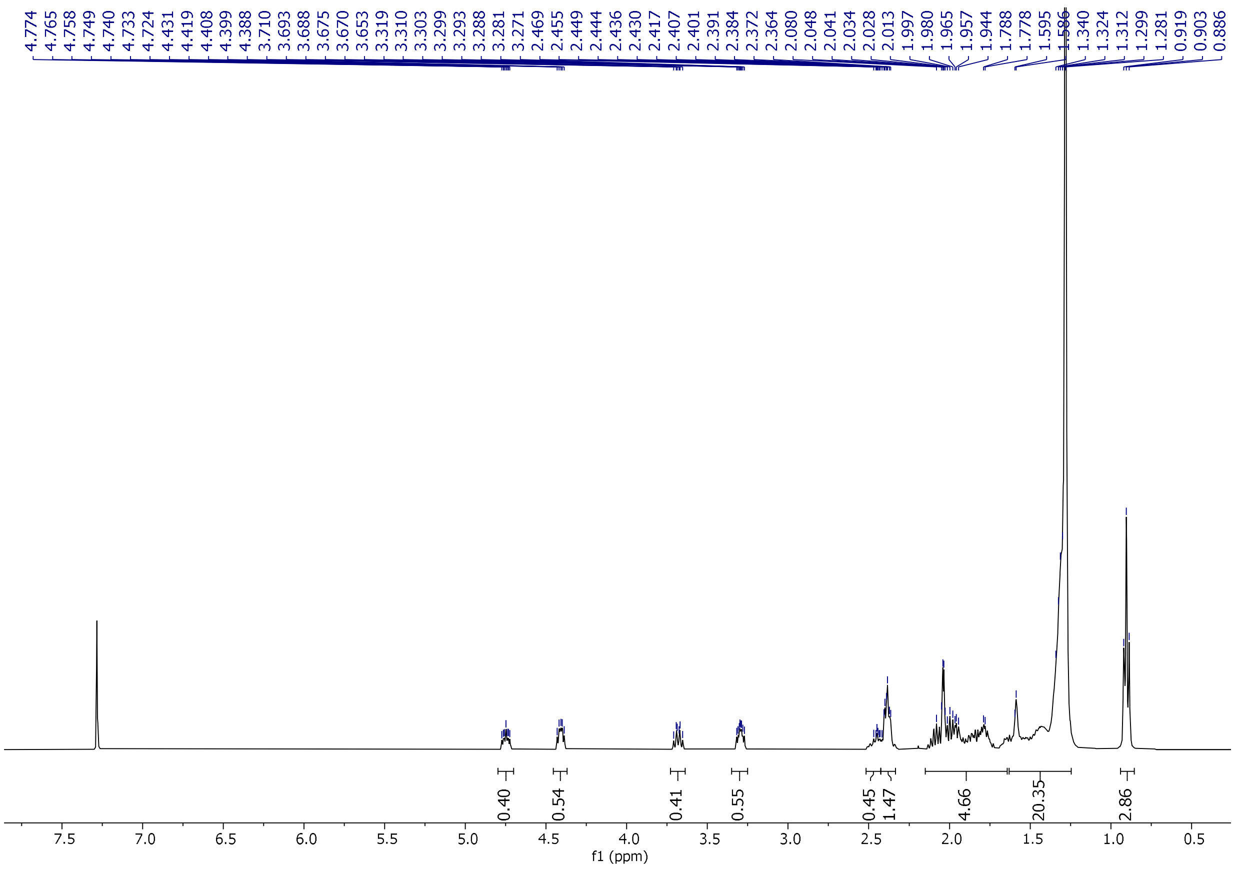
4.774
4.765
4.758
4.749
4.740
4.733
4.724
4.431
4.419
4.408
4.399
4.388
3.710
3.693
3.688
3.675
3.670
3.653
3.319
3.310
3.303
3.299
3.293
3.288
3.281
3.271
2.469
2.455
2.449
2.444
2.436
2.430
2.417
2.407
2.401
2.391
2.384
2.372
2.364
2.080
2.048
2.041
2.034
2.028
2.013
1.997
1.980
1.965
1.957
1.944
1.788
1.778
1.595
1.586
1.340
1.324
1.312
1.299
1.281
0.919
0.903
0.886
0.40
0.54
0.41
0.55
0.45
1.47
4.66
20.35
2.86
7.5
7.0
6.5
6.0
5.5
5.0
4.5
4.0
3.5
3.0
2.5
2.0
1.5
1.0
0.5
f1 (ppm)

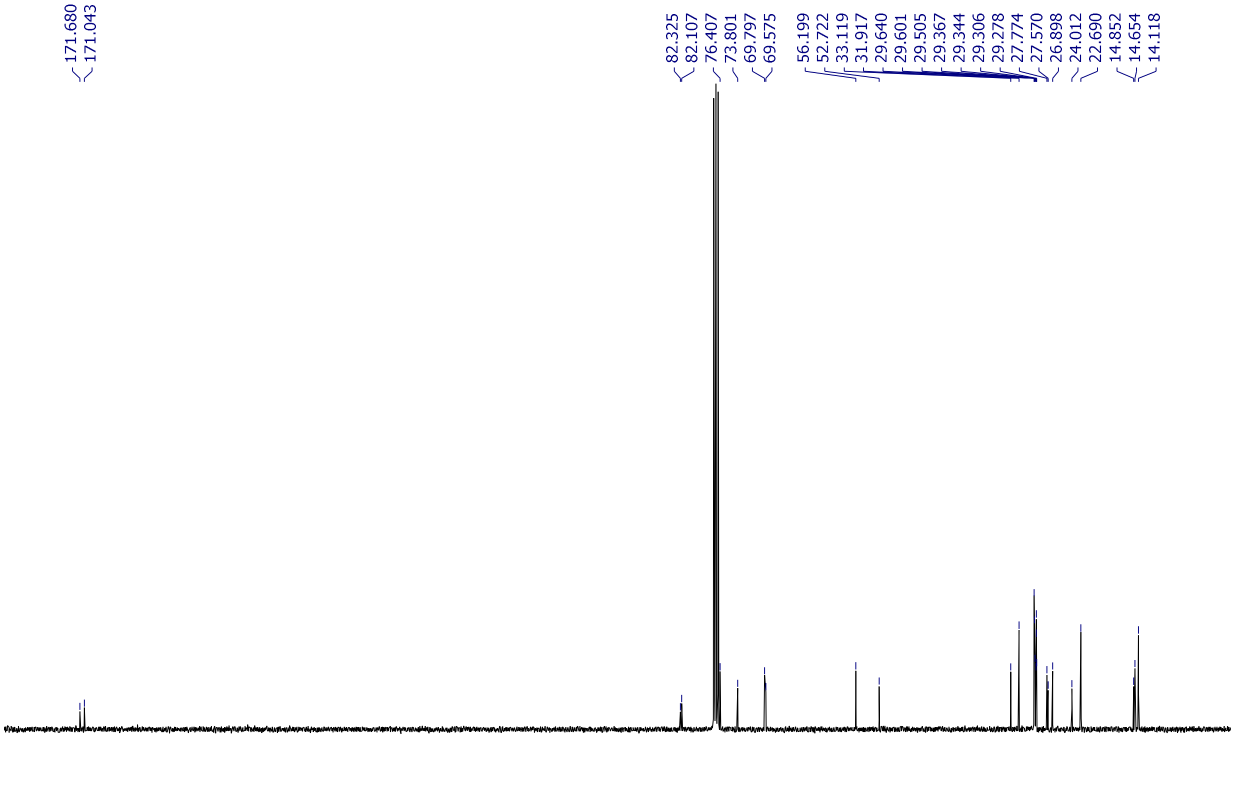
171.680
171.043
82.325
82.107
76.407
73.801
69.797
69.575
56.199
52.722
33.119
31.917
29.640
29.601
29.505
29.367
29.344
29.306
29.278
27.774
27.570
26.898
24.012
22.690
14.852
14.654
14.118
180
170
160
150
140
130
120
110
100
90
80
70
60
50
40
30
20
10
f1 (ppm)

VM055*p*

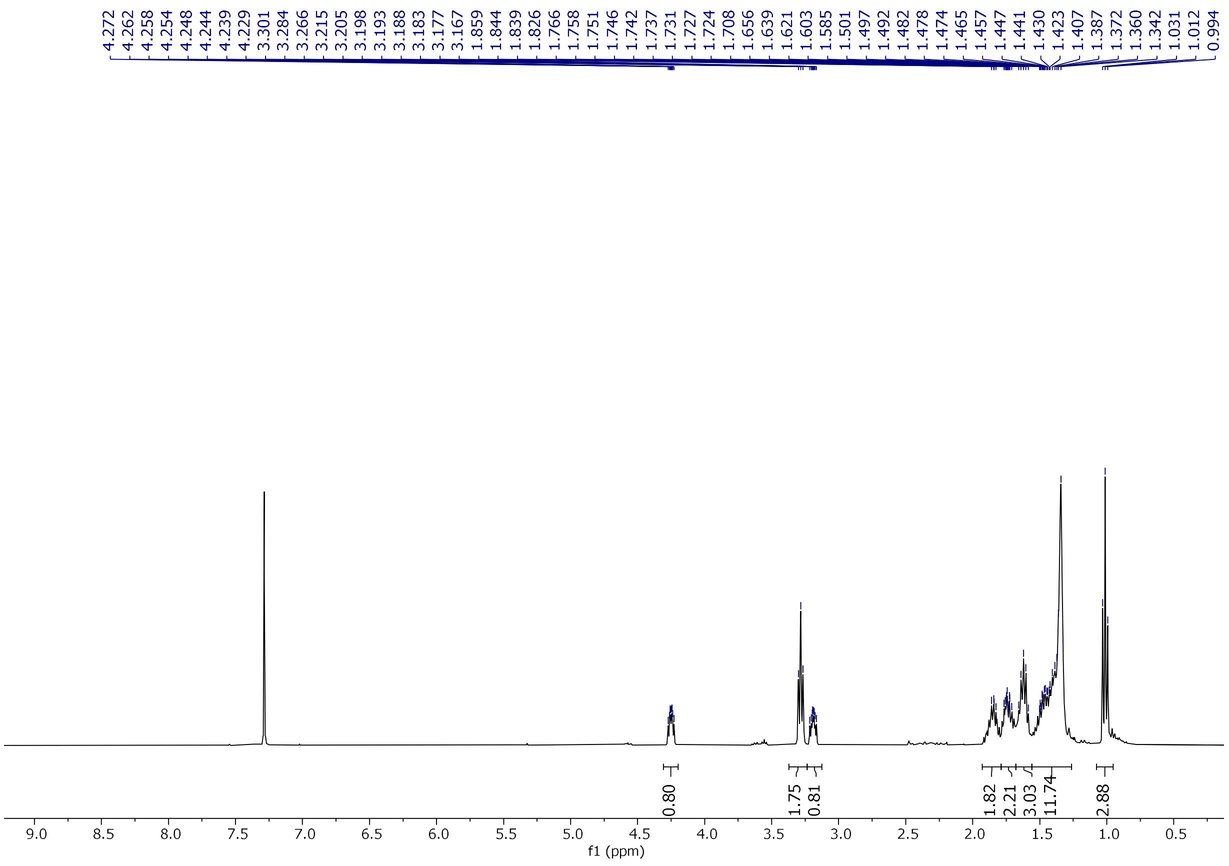

4.272
4.262
4.258
4.254
4.248
4.244
4.239
4.229
3.301
3.284
3.266
3.215
3.205
3.198
3.193
3.188
3.183
3.177
3.167
1.859
1.844
1.839
1.826
1.766
1.758
1.751
1.746
1.742
1.737
1.731
1.727
1.724
1.708
1.656
1.639
1.621
1.603
1.585
1.501
1.497
1.492
1.482
1.478
1.474
1.465
1.457
1.447
1.441
1.430
1.423
1.407
1.387
1.372
1.360
1.342
1.031
1.012
0.994
0.80
1.75
0.81
1.82
2.21
3.03
11.74
2.88
9.0
8.5
8.0
7.5
7.0
6.5
6.0
5.5
5.0
4.5
4.0
3.5
3.0
2.5
2.0
1.5
1.0
0.5
f1 (ppm)

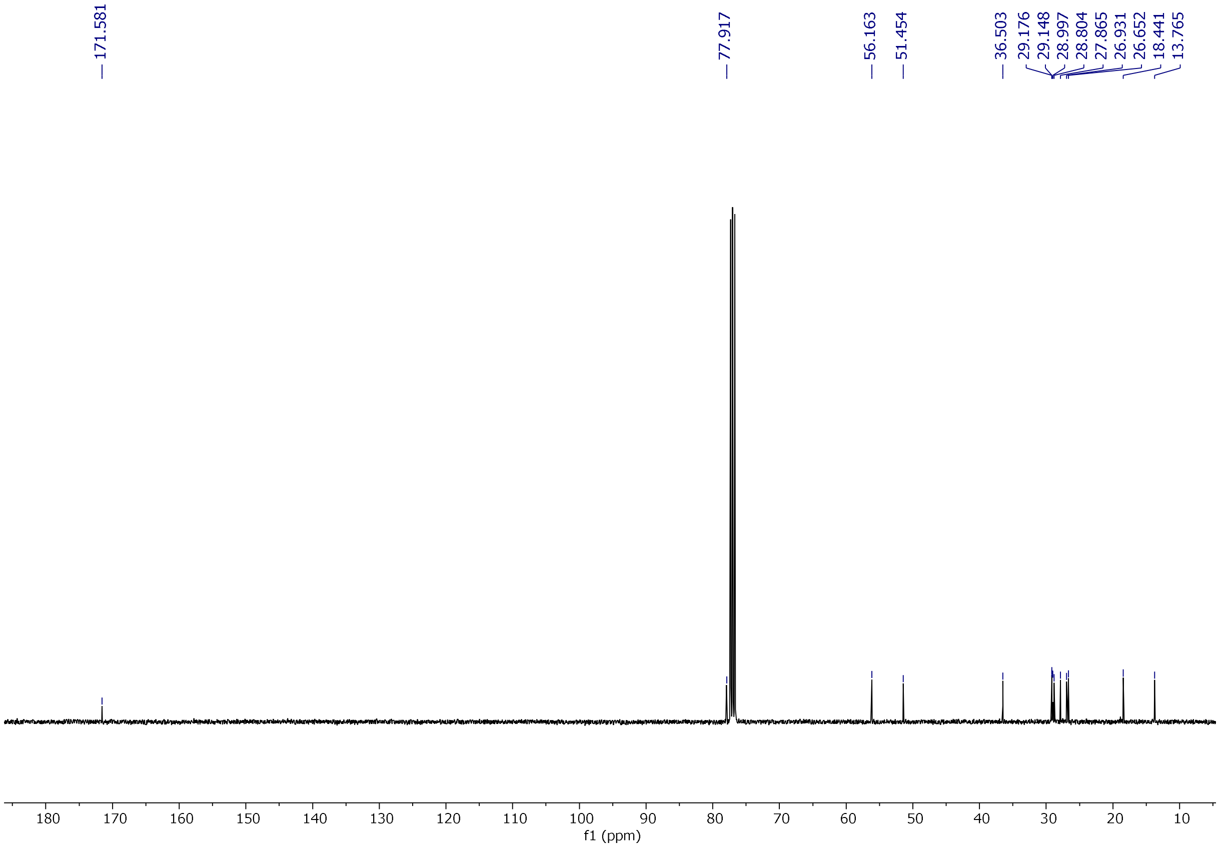

171.581
77.917
56.163
51.454
36.503
29.176
29.148
28.997
28.804
27.865
26.931
26.652
18.441
13.765
180
170
160
150
140
130
120
110
100
90
80
70
60
50
40
30
20
10
f1 (ppm)

References

1. J. C. Palomino, A. Martin, M. Camacho, H. Guerra, J. Swings and F. Portaels, Resazurin microtiter assay plate: simple and inexpensive method for detection of drug resistance in *Mycobacterium tuberculosis*, *Antimicrob Agents Chemother*, 2002, **46**, 2720-2722.
2. A. Madani, J. N. Ridenour, B. P. Martin, R. R. Paudel, A. Abdul Basir, V. Le Moigne, J. L. Herrmann, S. Audebert, L. Camoin, L. Kremer, C. D. Spilling, S. Canaan and J.-F. Cavalier, Cyclipostins and Cyclophostin Analogues as Multitarget Inhibitors That Impair Growth of *Mycobacterium abscessus*, *ACS Infect Dis*, 2019, **5**, 1597-1608.
3. P. Santucci, C. Dedaki, A. Athanasoulis, L. Gallorini, A. Munoz, S. Canaan, J.-F. Cavalier and V. Magrioti, Synthesis of long chain β-lactones and their antibacterial activities against pathogenic mycobacteria, *ChemMedChem*, 2019, **14**, 349-358.
4. C. Rodrigues Felix, R. Gupta, S. Geden, J. Roberts, P. Winder, S. A. Pomponi, M. C. Diaz, J. K. Reed, A. E. Wright and K. H. Rohde, Selective Killing of Dormant *Mycobacterium tuberculosis* by Marine Natural Products, *Antimicrob Agents Chemother*, 2017, **61**, e00743-00717.
5. T. Christophe, M. Jackson, H. K. Jeon, D. Fenistein, M. Contreras-Dominguez, J. Kim, A. Genovesio, J. P. Carralot, F. Ewann, E. H. Kim, S. Y. Lee, S. Kang, M. J. Seo, E. J. Park, H. Skovierova, H. Pham, G. Riccardi, J. Y. Nam, L. Marsollier, M. Kempf, M. L. Joly-Guillou, T. Oh, W. K. Shin, Z. No, U. Nehrbass, R. Brosch, S. T. Cole and P. Brodin, High content screening identifies decaprenyl-phosphoribose 2' epimerase as a target for intracellular antimycobacterial inhibitors, *PLoS Pathog*, 2009, **5**, e1000645.
6. P. C. Nguyen, V. Delorme, A. Benarouche, A. Guy, V. Landry, S. Audebert, M. Pophillat, L. Camoin, C. Crauste, J. M. Galano, T. Durand, P. Brodin, S. Canaan and J.-F. Cavalier, Oxadiazolone derivatives, new promising multi-target inhibitors against *M. tuberculosis*, *Bioorg Chem*, 2018, **81**, 414-424.
7. P. C. Nguyen, V. Delorme, A. Benarouche, B. P. Martin, R. Paudel, G. R. Gnawali, A. Madani, R. Puppo, V. Landry, L. Kremer, P. Brodin, C. D. Spilling, J.-F. Cavalier and S. Canaan, Cyclipostins and Cyclophostin analogs as promising compounds in the fight against tuberculosis, *Scientific Reports*, 2017, **7**, 11751.
8. C. Dupont, A. Viljoen, F. Dubar, M. Blaise, A. Bernut, A. Pawlik, C. Bouchier, R. Brosch, Y. Guerardel, J. Lelievre, L. Ballell, J. L. Herrmann, C. Biot and L. Kremer, A new piperidinol derivative targeting mycolic acid transport in *Mycobacterium abscessus*, *Molecular Microbiology*, 2016, **101**, 515-529.
9. M. Sarrazin, B. P. Martin, R. Avellan, G. R. Gnawali, I. Poncin, H. Le Guenno, C. D. Spilling, J. F. Cavalier and S. Canaan, Synthesis and Biological Characterization of Fluorescent Cyclipostins and Cyclophostin Analogues: New Insights for the Diagnosis of Mycobacterial-Related Diseases, *ACS Infect Dis*, 2022, **8**, 2564-2578.
10. B. S. Simcox, B. R. Tomlinson, L. N. Shaw and K. H. Rohde, *Mycobacterium abscessus* DosRS two-component system controls a species-specific regulon required for adaptation to hypoxia, *Front Cell Infect Microbiol*, 2023, **13**, 1144210.

11. D. G. Lee, Y. H. Hwang, E. J. Park, J. H. Kim and S. W. Ryoo, Clomiphene Citrate Shows Effective and Sustained Antimicrobial Activity against *Mycobacterium abscessus*, *Int J Mol Sci*, 2021, **22**.
12. J. C. Jewett and C. R. Bertozzi, Cu-free click cycloaddition reactions in chemical biology, *Chem Soc Rev*, 2010, **39**, 1272-1279.
13. J. C. Jewett, E. M. Sletten and C. R. Bertozzi, Rapid Cu-free click chemistry with readily synthesized biarylazacyclooctynones, *J Am Chem Soc*, 2010, **132**, 3688-3690.
14. B. M. Babin, L. Atangcho, M. B. van Eldijk, M. J. Sweredoski, A. Moradian, S. Hess, T. Tolker-Nielsen, D. K. Newman and D. A. Tirrell, Selective Proteomic Analysis of Antibiotic-Tolerant Cellular Subpopulations in *Pseudomonas aeruginosa* Biofilms, *mBio*, 2017, **8**.
15. A. M. Frankenfield, J. Ni, M. Ahmed and L. Hao, Protein Contaminants Matter: Building Universal Protein Contaminant Libraries for DDA and DIA Proteomics, *J Proteome Res*, 2022, **21**, 2104-2113.
16. M. A. Gerault, L. Camoin and S. Granjeaud, DIAgui: a Shiny application to process the output from DIA-NN, *Bioinform Adv*, 2024, **4**, vbae001.
17. S. Tyanova and J. Cox,Perseus: A Bioinformatics Platform for Integrative Analysis of Proteomics Data in Cancer Research, in *Cancer Systems Biology: Methods and Protocols*, ed. L. von Stechow, Springer New York, New York, NY, 2018, pp. 133-148. https://doi.org/10.1007/978-1-4939-7493-1_7
18. V. G. Tusher, R. Tibshirani and G. Chu, Significance analysis of microarrays applied to the ionizing radiation response, *Proc Natl Acad Sci U S A*, 2001, **98**, 5116-5121.
19. E. W. Deutsch, N. Bandeira, V. Sharma, Y. Perez-Riverol, J. J. Carver, D. J. Kundu, D. Garcia-Seisdedos, A. F. Jarnuczak, S. Hewapathirana, B. S. Pullman, J. Wertz, Z. Sun, S. Kawano, S. Okuda, Y. Watanabe, H. Hermjakob, B. MacLean, M. J. MacCoss, Y. Zhu, Y. Ishihama and J. A. Vizcaino, The ProteomeXchange consortium in 2020: enabling 'big data' approaches in proteomics, *Nucleic Acids Res*, 2020, **48**, D1145-D1152.
20. Y. Perez-Riverol, J. Bai, C. Bandla, D. Garcia-Seisdedos, S. Hewapathirana, S. Kamatchinathan, D. J. Kundu, A. Prakash, A. Frericks-Zipper, M. Eisenacher, M. Walzer, S. Wang, A. Brazma and J. A. Vizcaino, The PRIDE database resources in 2022: a hub for mass spectrometry-based proteomics evidences, *Nucleic Acids Res*, 2022, **50**, D543-D552.
21. S. P. Morcillo, D. Leboeuf, C. Bour and V. Gandon, Calcium-Catalyzed Synthesis of Polysubstituted 2-Alkenylfurans from beta-Keto Esters Tethered to Propargyl Alcohols, *Chemistry*, 2016, **22**, 16974-16978.
22. N. Zhang, J. Jiang, M. Liu, M. Taniguchi, A. K. Mandal, R. B. Evans-Storms, J. B. Pitner, D. F. Bocian, D. Holten and J. S. Lindsey, Bioconjugatable, PEGylated Hydroporphyrins for Photochemistry and Photomedicine. Narrow-Band, Near-Infrared-Emitting Bacteriochlorins, *New J Chem*, 2016, **40**, 7750-7767.
23. T. Kai, X. L. Sun, K. M. Faucher, R. P. Apkarian and E. L. Chaikof, Design and synthesis of asymmetric acyclic phospholipid bolaamphiphiles, *J Org Chem*, 2005, **70**, 2606-2615.

24. C. Cerutti-Delasalle, M. Mehiri, C. Cagliero, P. Rubiolo, C. Bicchi, U. J. Meierhenrich and N. Baldovini, The (+)-cis- and (+)-trans-Olibanic Acids: Key Odorants of Frankincense, *Angew Chem Int Ed Engl*, 2016, **55**, 13719-13723.

25. A. Gansauer, C. A. Fan, F. Keller and J. Keil, Titanocene-catalyzed regiodivergent epoxide openings, *J Am Chem Soc*, 2007, **129**, 3484-3485.

26. W. W. Zhang, T. T. Gao, L. J. Xu and B. J. Li, Macrolactonization of Alkynyl Alcohol through Rh(I)/Yb(III) Catalysis, *Org Lett*, 2018, **20**, 6534-6538.

27. S. Hamada, M. Sumida, R. Yamazaki, Y. Kobayashi and T. Furuta, Oxidative Deprotection of Benzyl Protecting Groups for Alcohols by an Electronically Tuned Nitroxyl-Radical Catalyst, *J Org Chem*, 2023, **88**, 12464-12473.

28. Y. Yu, D. Zhai, Z. Zhou, S. Jiang, H. Qian and S. Ma, Copper-catalyzed aerobic oxidation of primary alcohols to carboxylic acids, *Chem Commun (Camb)*, 2023, **59**, 5281-5284.

29. S. K. Rangaraju, U. M. Gonela, A. Kavita, J. S. Yadav and D. K. Mohapatra, Synergistic Gold and Copper Dual Catalysis for Intramolecular Glaser–Hay Coupling: Rapid Total Synthesis of Ivorenolide B, *Eur J Org Chem*, 2018, **2018**, 4376-4380.

30. H. Yamakoshi, H. Ohori, C. Kudo, A. Sato, N. Kanoh, C. Ishioka, H. Shibata and Y. Iwabuchi, Structure-activity relationship of C5-curcuminoids and synthesis of their molecular probes thereof, *Bioorg Med Chem*, 2010, **18**, 1083-1092.

31. M. J. Fer, A. Bouhss, M. Patrao, L. Le Corre, N. Pietrancosta, A. Amoroso, B. Joris, D. Mengin-Lecreulx, S. Calvet-Vitale and C. Gravier-Pelletier, 5'-Methylene-triazole-substituted-aminoribosyl uridines as MraY inhibitors: synthesis, biological evaluation and molecular modeling, *Org Biomol Chem*, 2015, **13**, 7193-7222.

32. E. Riva, I. Wilkening, S. Gazzola, W. M. Li, L. Smith, P. F. Leadlay and M. Tosin, Chemical probes for the functionalization of polyketide intermediates, *Angew Chem Int Ed Engl*, 2014, **53**, 11944-11949.

33. A. Daina, O. Michielin and V. Zoete, iLOGP: a simple, robust, and efficient description of n-octanol/water partition coefficient for drug design using the GB/SA approach, *J Chem Inf Model*, 2014, **54**, 3284-3301.

34. A. Daina, O. Michielin and V. Zoete, SwissADME: a free web tool to evaluate pharmacokinetics, drug-likeness and medicinal chemistry friendliness of small molecules, *Scientific Reports*, 2017, **7**, 42717.
