# Supplementary material for: Synthesis and anti-mycobacterial activity of novel medium-chain β-lactone derivatives: a multi-target strategy to combat Mycobacterium abscessus
Source: RSC Med Chem. 2025 Apr 25;16(7):3251–72. doi: 10.1039/d5md00102a (PMC12101465; doi:10.1039/d5md00102a)
Supplement: MD-016-D5MD00102A-s002 [file MD-016-D5MD00102A-s002.pdf]

This journal is © The Royal Society of Chemistry 2025

Overview Supplementary Tables

**Table S2**	**VM055*p*** target proteins identified from *M. abscessus* S culture through CC-ABPP by LC-ESI-MS/MS analysis by applying a *p* -value <0.05 and a fold change (Log2) ≥1, compared to DMSO-treated cells (*i.e* ., non-specific conditions, NS)	Suppl. Table 2
**Table S3**	Differentially enriched *M. abscessus* S proteins between **VM043** pre-incubated samples followed by **VM055*p*** probe labeling *vs* . **VM055*p*** probe-labeled samples only.	Suppl. Table 3
**Table S4**	Positively enriched proteins captured by **VM055*p*** probe from *M. abscessus* S culture through CC-ABPP by LC-ESI-MS/MS analysis, compared to DMSO-treated cells (*i.e* ., non-specific conditions, NS)	Suppl. Table 4
**Table S5**	Positively enriched proteins captured by **VM055*p*** probe from pre-incubated **VM043**-*M. abscessus* S culture through CC-ABPP by LC-ESI-MS/MS analysis by applying a *p* -value <0.05 and a fold change (Log2) ≥1, compared to DMSO-treated cells (i.e., non-specific conditions, NS)	Suppl. Table 5
**Table S6**	Differential analysis of the *M. abscessus* S labeled proteome of samples pre-incubated with **VM043** inhibitor followed by **VM055*p*** probe labeling *vs* . **VM055*p*** probe-labeled samples only	Suppl. Table 6
**Table S7**	Cytotoxic activities of the β-lactone analogs towards Raw264.7 murine macrophage cells.	Suppl. Table 7

Table S2. VM055*p* target proteins identified from *M. abscessus* S culture through CC-ABPP by LC-ESI-MS/MS analysis by applying a *p*-value <0.05 and a fold change (Log_2) ≥1, compared to DMSO-treated cells (*i.e.*, non-specific conditions, NS)

					***Mtb* ortholog**							**VM055 *vs*. NS**	
**Entry**	**Protein IDs**	**Protein names**	**Gene names**	**Essentiality**	**Rv number**	**Seq. identity**	**Overlap**	**Essentiality**	**Functional category**	**peptides counts all**	**nb Tryptic Peptides**	**-LOG(p-value)**	**Fold Change (Log2)**
1	B1MEQ6	non-specific serine/threonine protein kinase	MAB_3436	non essential	Rv0014c	0.385	96	*in vitro* growth	Regulatory proteins	1	5	3.755	2.503
2	B1MKX5	Possible acylglycerol lipase	MAB_4551c	non essential	Rv0183	0.649	273		Lipid metabolism	5	19	3.654	2.077
3	B1MJG0	Hypothetical protein	MAB_1026c	non essential	Rv1926c	0.47	151		Cell wall and cell processes	3	9	2.670	2.000
4	B1MFK1	Putative quinolone synthase/monooxygenase	MAB_0302, aqdB	non essential	Rv1260	0.263	250		Intermediary metabolism and respiration	3	18	1.658	1.753
5	B1MIY6	Hypothetical protein	MAB_4284c	non essential	Rv3514	0.336	553		Pe/ppe	4	8	1.751	1.466
6	B1MNS7	Hypothetical protein	MAB_1895c	non essential	Rv0278c	0.281	320		Pe/ppe	3	18	4.566	1.438
7	B1MJB0	Hypothetical protein	MAB_0974	non essential	Rv3876, espI	0.238	369		Cell wall and cell processes	13	29	2.691	1.411
8	B1MEX1	Putative hydrolase	MAB_3501	non essential	Rv3195	0.709	447		Conserved hypotheticals	11	20	3.841	1.403
9	B1MF35	Putative cyclase	MAB_3566c	non essential	-					4	15	3.921	1.253
10	B1MMK7	Hypothetical protein	MAB_4924	growth advantage	Rv0040c	0.434	304		Cell wall and cell processes	4	10	3.975	1.249
11	B1MB54	Hypothetical protein	MAB_2394	non essential	Rv0910	0.266	154		Conserved hypotheticals	3	10	3.537	1.200
12	B1MCL9	Probable aldolase	MAB_2912c	non essential	Rv0727c	0.275	218		Intermediary metabolism and respiration	8	13	1.641	1.173

The Essentiality of each *M. abscessus* gene was checked using the Tables S1B from Rifat et al. mBio (2021) 12:e01049-21. https://doi.org/10.1128/mBio.01049-21

Table S3. Differentially enriched *M. abscessus* S proteins between VM043 pre-incubated samples followed by VM055*p* probe labeling vs. VM055*p* probe-labeled samples only.

						Mtb ortholog							[VM043+VM055] *vs* . VM055		VM055 *vs* . NS		[VM043+VM055] *vs.* NS	
	Entry	Protein IDs	Protein names	Gene names	Essentiality	Rv number	Seq. identity	Overlap	Essantiality	Functional category	peptides counts all	nb Tryptic Peptides	-LOG(p-value)	Fold Change (Log2)	-LOG(p-value)	Fold Change (Log2)	-LOG(p-value)	Fold Change (Log2)
**VM055*p* target proteins inhibited by VM043**	1	B1MCL9	Probable aldolase	MAB_2912c	non essential	Rv0727c	0.275	218		Intermediary metabolism and respiration	8	13	1.319	-1.810	1.641	1.173	0.162	-0.637
	2	B1MFK1	Putative quinolone synthase	MAB_0302, aqdB	non essential	Rv1260	0.263	250		Intermediary metabolism and respiration	3	18	1.411	-1.189	1.658	1.753	0.911	0.780
	3	B1MEQ6	non-specific serine/threonine protein kinase	MAB_3436	non essential	Rv0014c	0.385	96	*in vitro* growth	Regulatory proteins	1	5	1.169	-1.080	3.755	2.503	1.326	1.423
	4	B1MB54	Uncharacterized protein	MAB_2394	non essential	Rv0910	0.266	154		Conserved hypotheticals	3	10	1.201	-1.073	3.537	1.200	0.390	0.128
**VM055*p* target proteins non inhibited by VM043**	5	B1MF33	Hypothetical dipeptidyl aminopeptidase	MAB_3564c	non essential	-					10	27	3.543	2.606	0.818	0.883	5.765	3.488
	6	B1MEW6	Uncharacterized protein	MAB_3496	non essential	-					2	6	3.839	1.030	0.790	0.579	3.179	1.608
	7	B1MBL9	Peptidyl-prolyl cis-trans isomerase	MAB_2559c	growth advantage	Rv3909	0.311	148	*in vitro* growth	Conserved hypotheticals	6	7	1.897	1.431	0.370	0.450	2.369	1.418
	8	B1MDI7	SnoaL-like domain-containing protein = small polyketide cyclase	MAB_3230c	non essential	Rv2910c	0.641	128		Conserved hypotheticals	3	6	1.098	1.712	0.324	0.375	1.347	1.874

The Essentiality of each *M. abscessus* gene was checked using the Tables S1B from Rifat et al. mBio (2021) 12:e01049-21. https://doi.org/10.1128/mBio.01049-21

Table S4. Positively enriched proteins captured by VM055*p* probe from *M. abscessus* S culture through CC-ABPP by LC-ESI-MS/MS analysis, compared to DMSO-treated cells (*i.e*., non-specific conditions, NS)

	Protein IDs	Gene names	Protein names	VM055*p* *vs*. NS -LOG(p-value)	VM055*p* *vs*. NS Fold Change (Log2)	peptides counts all	nb Tryptic Peptides
1	B1MEQ6	*MAB_3436*	non-specific serine/threonine protein kinase	3.755	2.503	3	18
2	B1MKX5	*MAB_4551c*	Possible lysophospholipase	3.654	2.077	1	6
3	B1MJG0	*MAB_1026c*	Uncharacterized protein	2.67	2.000	3	3
4	B1MFK1	*MAB_0302*	2-heptyl-3-hydroxy-4(1H)-quinolone synthase	1.658	1.753	11	20
5	B1MIY6	*MAB_4284c*	Uncharacterized protein	1.751	1.466	13	29
6	B1MNS7	*MAB_1895c*	Uncharacterized protein	4.566	1.438	1	5
7	B1MJB0	*MAB_0974*	Uncharacterized protein	2.691	1.411	3	18
8	B1MEX1	*MAB_3501*	Uncharacterized protein	3.841	1.403	3	9
9	B1MF35	*MAB_3566c*	Putative cyclase	3.921	1.253	2	4
10	B1MMK7	*MAB_4924*	Uncharacterized protein	3.975	1.249	5	19
11	B1MB54	*MAB_2394*	Uncharacterized protein	3.537	1.200	4	8
12	B1MCL9	*MAB_2912c*	Probable aldolase	1.641	1.173	4	15
13	B1MFL8	*MAB_0319*	Nucleoid-associated protein MAB_0319	1.276	2.019	4	10
14	B1MFY2	*MAB_3653*	Probable pyridoxine 5-phosphate oxidase	1.236	1.047	12	9
15	B1MCJ4	*MAB_2886c*	Uncharacterized protein	1.137	1.434	3	10
16	B1MI56	*MAB_0782*	Uncharacterized protein	1.111	1.231	2	10
17	B1MMX9	*ndk*	Nucleoside diphosphate kinase	0.969	1.183	8	13
18	B1MKV0	*MAB_4526*	Uncharacterized protein	0.641	1.751	2	27
19	B1MCT9	*MAB_2982c*	Uncharacterized protein	0.479	1.097	4	15
20	B1MKH2	*MAB_1176c*	Uncharacterized protein	0.856	0.958	2	10
21	B1MBJ7	*MAB_2537c*	Putative pyruvate decarboxylase	3.301	0.943	4	38
22	B1MB33	*MAB_2373*	Putative mannose-specific lectin	0.588	0.937	3	6
23	B1MIP4	*MAB_4192*	Uncharacterized protein	0.72	0.924	9	13
24	B1MJS5	*MAB_4361*	Hypothetical fumarylacetoacetate hydrolase family	0.717	0.923	15	16
25	B1MAV6	*rpsA*	30S ribosomal protein S1	1.744	0.905	31	37
26	B1MF33	*MAB_3564c*	Hypothetical dipeptidyl aminopeptidase/ acylaminoacyl-peptidase related protein	0.818	0.883	10	27
27	B1MKS6	*MAB_1280c*	Uncharacterized protein	4.577	0.882	2	17
28	B1MAK3	*MAB_2193c*	Putative_PNPOx domain-containing protein	0.919	0.88	4	12
29	B1MK18	*MAB_4456*	Putative cytochrome P450	0.746	0.842	16	28
30	B1MMU7	*MAB_1574*	Hypothetical carbohydrate-phosphate isomerase	0.317	0.84	12	11
31	B1MGT9	*MAB_0524c*	Probable conserved lipoprotein LpqG	2.14	0.817	3	12
32	B1MNW3	*MAB_1931c*	Conserved hypothetical integral membrane protein	0.769	0.814	1	2
33	B1MJ29	*MAB_0891c*	Putative HTH-type transcriptional regulator MarR	0.638	0.813	12	15
34	B1MHQ3	*MAB_4060*	Putative short chain dehydrogenase/reductase	1.411	0.801	10	15
35	B1MMX4	*MAB_1601c*	Putative monooxygenase	0.811	0.801	11	26
36	B1MJ72	*MAB_0936c*	PE-PPE domain-containing protein	1.208	0.781	9	16
37	B1MD60	*MAB_3104c*	Uncharacterized protein	0.526	0.763	2	6
38	B1MG83	*MAB_3754c*	ESAT-6-like protein	0.464	0.76	14	7
39	B1MAV3	*MAB_2293*	Putative transcriptional regulator, MarR family	2.172	0.752	6	11
40	B1MAH0	*MAB_2160c*	Putative lipoprotein LppK	0.817	0.75	9	10
41	B1MLT6	*MAB_1427c*	Putative cytochrome P450	1.583	0.746	18	31
42	B1MCW2	*MAB_3005c*	Uncharacterized protein	0.321	0.746	7	6
43	B1MKN7	*MAB_1241c*	CsbD domain-containing protein	1.261	0.731	6	6
44	B1MDG5	*MAB_3210c*	Uncharacterized protein	0.669	0.727	4	14
45	B1MHJ8	*proC*	Pyrroline-5-carboxylate reductase	0.657	0.72	10	15
46	B1MNR8	*MAB_1886*	Uncharacterized protein	0.371	0.708	4	7
47	B1MD17	*recA*	Protein RecA	1.828	0.698	26	23

Table S4. Positively enriched proteins captured by VM055*p* probe from *M. abscessus* S culture through CC-ABPP by LC-ESI-MS/MS analysis, compared to DMSO-treated cells (*i.e*., non-specific conditions, NS)

	Protein IDs	Gene names	Protein names	VM055*p* *vs*. NS -LOG(p-value)	VM055*p* *vs*. NS Fold Change (Log2)	peptides counts all	nb Tryptic Peptides
48	B1MM28	*MAB_4745*	Putative membrane protein, MmpS	0.136	0.694	1	8
49	B1MHG8	*MAB_3975c*	Putative cytochrome C biogenesis protein CcdA	0.698	0.693	1	5
50	B1MB28	*MAB_2368*	Uncharacterized protein	0.537	0.687	2	14
51	B1MCY5	*MAB_3028*	RNA polymerase sigma factor	0.756	0.686	12	25
52	B1MDH2	*MAB_3217*	CsbD domain-containing protein	1.161	0.684	7	11
53	B1MIN9	*def*	Peptide deformylase	0.375	0.675	4	8
54	B1MJ83	*MAB_0947c*	Putative luciferase	1.119	0.673	6	21
55	B1MMF1	*MAB_4868c*	Uncharacterized protein	0.586	0.67	10	15
56	B1MLR7	*MAB_1408c*	Putative short-chain dehydrogenase/reductase	0.385	0.662	6	9
57	B1MET0	*MAB_3460c*	Uncharacterized protein	0.642	0.656	4	16
58	B1MJV3	*MAB_4389*	Putative regulatory protein, TetR family	0.666	0.647	3	15
59	B1MGP4	*MAB_0479*	Putative regulatory protein, MarR	0.436	0.645	4	11
60	B1MF07	*MAB_3537c*	Uncharacterized protein	0.345	0.645	2	2
61	B1MK17	*MAB_4455c*	Probable acyl-CoA synthetase FadD	1.522	0.643	4	32
62	B1MKT6	*tpx*	Thiol peroxidase	0.655	0.641	9	9
63	B1MCT6	*MAB_2979*	Peptide-methionine (R)-S-oxide reductase	0.366	0.639	12	9
64	B1MKI1	*MAB_1185c*	Probable enoyl-CoA hydratase	2.786	0.638	14	16
65	B1ML36	*MAB_4612*	NTP_transf_9 domain-containing protein	0.378	0.635	4	8
66	B1MCH9	*MAB_2871c*	Uncharacterized protein	0.6	0.632	1	7
67	B1MIW7	*clpB*	Chaperone protein ClpB	0.706	0.628	77	60
68	B1ML91	*MAB_4667*	Putative amidohydrolase	0.37	0.603	9	17
69	B1MGU0	*MAB_0525c*	Probable conserved lipoprotein LpqG	1.642	0.601	5	15
70	B1MLM0	*MAB_1361c*	Putative O-methyltransferase MAB_1361c	1.389	0.594	3	14
71	B1MDE3	*frr*	Ribosome-recycling factor	0.716	0.594	9	14
72	B1MMY3	*rplU*	50S ribosomal protein L21	0.235	0.593	4	7
73	B1ME89	*MAB_0053c*	Probable cytidine/deoxycytidylate deaminase	0.788	0.591	3	13
74	B1MEN5	*MAB_0199*	Probable phosphoesterase, PA-phosphatase related protein	0.358	0.59	14	31
75	B1MCH5	*MAB_2867*	Uncharacterized protein	1.053	0.585	11	13
76	B1MNT0	*cobD*	Cobalamin biosynthesis protein CobD	1.18	0.584	10	19
77	B1MLP6	*MAB_1387*	Putative esterase/lipase/beta-lactamase	0.825	0.584	5	28
78	B1MF17	*MAB_3547*	Uncharacterized protein	0.521	0.583	5	21
79	B1MP00	*MAB_1969c*	Cytochrome aa3 subunit 3	0.325	0.58	5	4
80	B1MPA8	*MAB_2078*	Probable cytochrome P450	1.307	0.579	13	39
81	B1MEW6	*MAB_3496*	Uncharacterized protein	0.79	0.579	2	6
82	B1MAD5	*MAB_p02*	Uncharacterized protein	1.065	0.57	3	12
83	B1MKX2	*MAB_4548*	Probable O-methyltransferase	0.598	0.569	10	16
84	B1MGQ2	*MAB_0487*	Probable cold shock protein A (CspA)	0.224	0.567	3	6
85	B1MJ79	*MAB_0943*	DJ-1_PfpI domain-containing protein	0.818	0.565	7	12
86	B1ME04	*MAB_3397*	HTH tetR-type domain-containing protein	1.475	0.561	5	11
87	B1MMU6	*MAB_1573c*	Uncharacterized protein	0.512	0.557	5	13
88	B1MDV0	*MAB_3343*	ACT domain-containing protein	0.243	0.557	8	11
89	B1MPA9	*MAB_2079*	TGc domain-containing protein	1.531	0.55	4	18
90	B1MCD4	*pyrF*	Orotidine 5'-phosphate decarboxylase	0.317	0.549	8	12
91	B1MP99	*MAB_2069*	Uncharacterized protein	0.617	0.548	15	18
92	B1MIX4	*grpE*	Protein GrpE	0.681	0.546	17	13
93	B1MMP7	*MAB_1523c*	Uncharacterized protein	0.295	0.542	2	11
94	B1MP79	*MAB_2048c*	Probable cytochrome P450	0.458	0.539	29	32

Table S4. Positively enriched proteins captured by VM055*p* probe from *M. abscessus* S culture through CC-ABPP by LC-ESI-MS/MS analysis, compared to DMSO-treated cells (*i.e*., non-specific conditions, NS)

	Protein IDs	Gene names	Protein names	VM055*p* *vs*. NS		peptides counts all	nb Tryptic Peptides
				-LOG(p-value)	Fold Change (Log2)		
95	B1MNK9	*MAB_1827c*	Bacteriophage protein	0.462	0.534	1	5
96	B1MLL1	*MAB_1350*	Uncharacterized protein	0.318	0.53	2	5
97	B1MLJ7	*MAB_1336*	Probable succinyl-diaminopimelate desuccinylase	0.603	0.529	19	21
98	B1MFV3	*MAB_0404c*	Putative acetyltransferase	0.281	0.527	2	12
99	B1MCF0	*aroK*	Shikimate kinase	0.657	0.52	2	16
100	B1MJB2	*MAB_0976*	Putative glyoxalase/bleomycin resistance protein	0.244	0.52	3	9
101	B1MHD8	*menB*	1,4-dihydroxy-2-naphthoyl-CoA synthase	0.194	0.512	18	19
102	B1MHS6	*groL*	60 kDa chaperonin	0.899	0.511	66	33
103	B1MLF7	*MAB_1296*	Uncharacterized protein	0.613	0.507	4	6
104	B1MHE4	*MAB_3951*	Uncharacterized protein	0.514	0.507	2	10
105	B1MHI2	*MAB_3989c*	Uncharacterized protein	0.386	0.507	2	5
106	B1MEI4	*MAB_0148c*	PPE family protein	0.29	0.507	8	14
107	B1MES5	*MAB_3455c*	Putative acyl-CoA thiolase	0.69	0.502	8	24
108	B1MCB9	*fmt*	Methionyl-tRNA formyltransferase	1.506	0.499	12	16
109	B1MN41	*MAB_1668*	PhoH-like protein	0.744	0.487	7	24
110	B1MHH7	*MAB_3984c*	Putative metal transporter ATPase	0.548	0.477	31	32
111	B1MCL8	*MAB_2911c*	Putative dipeptidase	0.307	0.472	10	21
112	B1MIM8	*MAB_4176c*	NAD_binding_9 domain-containing protein	0.562	0.471	2	25
113	B1MK42	*MAB_4480c*	Putative glycosyl transferase	1.489	0.462	7	32
114	B1MDF9	*xerC*	Tyrosine recombinase XerC	0.428	0.462	4	20
115	B1MLY5	*MAB_1476*	DUF2017 domain-containing protein	0.247	0.459	5	9
116	B1MP12	*MAB_1981*	Uncharacterized protein	0.518	0.457	6	10
117	B1MKH0	*MAB_1174c*	Probable acyl-CoA synthase FadD	0.325	0.453	15	32
118	B1MHY5	*MAB_0711*	SCP_3 domain-containing protein	0.641	0.45	4	9
119	B1MB98	*MAB_2438*	Probable oxidoreductase	0.43	0.45	6	71
120	B1MEQ7	*MAB_3437c*	Putative transcriptional regulator, TetR family	2.838	0.448	4	15
121	B1MFH6	*MAB_0276*	Probable cytochrome P450	0.582	0.441	19	24
122	B1MBK2	*MAB_2542*	Putative_PNPOx domain-containing protein	0.276	0.436	8	11
123	B1MKH7	*MAB_1181c*	Putative lipoprotein LpqV	0.436	0.435	3	6
124	B1MG61	*groS*	10 kDa chaperonin	0.509	0.433	11	8
125	B1MIA7	*MAB_0835c*	Putative transcriptional regulator, TetR family	0.443	0.43	6	14
126	B1MFY1	*MAB_3652*	VOC domain-containing protein	1.135	0.429	6	10
127	B1MMG7	*MAB_4884c*	Uncharacterized protein	0.235	0.429	7	11
128	B1MNU8	*panB*	3-methyl-2-oxobutanoate hydroxymethyltransferase	1.115	0.426	9	16
129	B1MD64	*rpsO*	30S ribosomal protein S15	0.878	0.425	14	7
130	B1MFB2	*MAB_3644*	Uncharacterized protein	0.301	0.424	7	7
131	B1MP48	*MAB_2017*	Antigen 84	0.689	0.422	29	23
132	B1MAS5	*ectC*	L-ectoine synthase	0.383	0.422	4	5
133	B1MB41	*MAB_2381*	Probable oxidoreductase	0.532	0.419	6	17
134	B1MN94	*MAB_4952c*	R3H domain-containing protein	0.376	0.419	4	12
135	B1MAE3	*MAB_p10*	Putative resolvase/invertase/recombinase	0.414	0.418	6	15
136	B1MFR6	*MAB_0367c*	SnoaL-like domain-containing protein	1.045	0.414	6	10
137	B1MMI2	*rpsF*	30S ribosomal protein S6	0.119	0.413	4	7
138	B1MIZ6	*MAB_4294*	Probable aspartate aminotransferase AspC	1.667	0.41	19	27
139	B1MMQ1	*MAB_1527*	Probable monooxygenase	0.627	0.407	18	27
140	B1MNR0	*acpP*	Acyl carrier protein	0.377	0.405	9	7
141	B1MM40	*MAB_4757*	Putative transcriptional regulator, TetR family	0.34	0.405	5	18

Table S4. Positively enriched proteins captured by VM055*p* probe from *M. abscessus* S culture through CC-ABPP by LC-ESI-MS/MS analysis, compared to DMSO-treated cells (*i.e.*, non-specific conditions, NS)

	Protein IDs	Gene names	Protein names	VM055*p* *vs*. NS -LOG(p-value)	VM055*p* *vs*. NS Fold Change (Log2)	peptides counts all	nb Tryptic Peptides
142	B1MJZ3	*MAB_4430c*	Putative oxidoreductase	0.106	0.405	3	5
143	B1ME59	*MAB_0020*	DUF3566 domain-containing protein	0.391	0.404	12	11
144	B1MN74	*MAB_1701*	Uncharacterized protein	0.193	0.403	1	20
145	B1MMV3	*tig*	Trigger factor	0.85	0.398	21	33
146	B1MLU1	*MAB_1432*	Uncharacterized protein	0.752	0.398	5	10
147	B1ML11	*MAB_4587c*	Putative S-adenosyl-L-methionine-dependent methyltransferase MAB_4587c	0.479	0.398	10	21
148	B1ME23	*MAB_3416*	Probable membrane transport protein	0.488	0.396	4	11
149	B1MCS1	*MAB_2964*	SnoaL-like domain-containing protein	0.533	0.392	3	11
150	B1MH69	*rplL*	50S ribosomal protein L7/L12	0.655	0.39	11	7
151	B1MP63	*MAB_2032*	Probable 3-oxoacyl-[acyl-carrier protein] reductase	0.283	0.389	4	12
152	B1MP78	*MAB_2047c*	Probable ferredoxin reductase	0.902	0.388	18	25
153	B1MLP0	*MAB_1381*	Putative oxidoreductase EphD	0.315	0.387	9	21
154	B1MFQ0	*MAB_0351*	Catalase	0.76	0.386	8	31
155	B1MLR8	*MAB_1409c*	Putative drug antiporter protein	0.218	0.386	3	13
156	B1MK03	*MAB_4440c*	Uncharacterized protein	0.564	0.379	3	12
157	B1MHZ6	*MAB_0722*	Putative oligopeptide ABC transporter,ATP-binding protein	0.298	0.379	11	35
158	B1MJB8	*MAB_0982c*	Putative acyl-CoA hydrolase/thioesterase	0.146	0.377	4	10
159	B1MIN3	*MAB_4181*	ATP-sulfurylase small subunit	0.747	0.367	7	20
160	B1MFX3	*MAB_0424*	Putative protease	0.436	0.367	2	10
161	B1MC58	*MAB_2750c*	Probable transcriptional regulatory protein	0.835	0.363	9	12
162	B1MI00	*MAB_0726*	Rieske domain-containing protein	0.723	0.36	4	22
163	B1MCB6	*MAB_2808c*	Riboflavin biosynthesis protein RibD	0.371	0.36	11	23
164	B1MGX2	*MAB_0558c*	Probable adenine glycosylase (MutY)	0.503	0.357	10	22
165	B1MGX0	*MAB_0555*	Phospholipase C	0.361	0.357	8	28
166	B1MCC7	*MAB_2819*	Putative steroid dehydrogenase	0.642	0.356	5	13
167	B1MHS0	*deoC*	Deoxyribose-phosphate aldolase	0.494	0.356	10	14
168	B1MHV3	*MAB_0678*	Probable transcriptional regulator, LysR family	0.464	0.351	4	15
169	B1MC54	*MAB_2746c*	Cysteine desulfurase	0.309	0.351	25	25
170	B1MH70	*rplJ*	50S ribosomal protein L10	0.872	0.35	11	17
171	B1MLB2	*MAB_4689c*	Uncharacterized protein	0.313	0.349	4	8
172	B1MG60	*groL*	60 kDa chaperonin	0.847	0.346	35	36
173	B1MM33	*MAB_4750*	Putative short chain dehydrogenase/reductase	0.703	0.346	1	15
174	B1MCC4	*MAB_2816c*	Putative luciferase-like protein	0.593	0.346	9	25
175	B1ML82	*MAB_4658*	Putative transcriptional regulator, LysR family	0.48	0.346	8	20
176	B1MLK7	*MAB_1346*	Glyco_trans_2-like domain-containing protein	0.42	0.343	7	11
177	B1MGA7	*MAB_3777*	Band 7 protein	0.186	0.343	15	36
178	B1MGG9	*MAB_3840*	Uncharacterized protein	1.146	0.342	2	17
179	B1MEJ7	*MAB_0161*	Probable transcriptional regulator, LysR family	0.257	0.342	13	17
180	B1MMQ2	*MAB_1528c*	Probable oxidoreductase	0.578	0.34	7	17
181	B1MKG9	*MAB_1173c*	Uncharacterized protein	0.452	0.333	6	15
182	B1MPE2	*MAB_2112*	Uncharacterized protein	0.386	0.333	3	11
183	B1MBP5	*MAB_2585*	L-ectoine synthase	0.286	0.328	4	6
184	B1MCL6	*MAB_2908c*	2-dehydropantoate 2-reductase	0.172	0.328	14	22
185	B1MCD9	*pyrB*	Aspartate carbamoyltransferase	0.233	0.326	14	18
186	B1MIE0	*MAB_4088c*	Possible mycolic acid synthase UmaA1	0.525	0.324	29	20
187	B1MP42	*MAB_2011*	Pyridoxal phosphate homeostasis protein	0.432	0.324	22	19
188	B1ME77	*MAB_0039c*	FHA domain-containing protein	0.354	0.324	10	21

Table S4. Positively enriched proteins captured by VM055*p* probe from *M. abscessus* S culture through CC-ABPP by LC-ESI-MS/MS analysis, compared to DMSO-treated cells (*i.e*., non-specific conditions, NS)

	Protein IDs	Gene names	Protein names	VM055*p* *vs*. NS -LOG(p-value)	VM055*p* *vs*. NS Fold Change (Log2)	peptides counts all	nb Tryptic Peptides
189	B1MDJ1	*MAB_3234*	Probable D-alanyl-D-alanine carboxypeptidase DacB	0.537	0.322	14	16
190	B1MBG5	*MAB_2505c*	Probable phosphoketolase	0.697	0.321	8	47
191	B1MEA5	*MAB_0069*	Major facilitator family transporter	0.417	0.317	6	12
192	B1MG02	*MAB_3673*	Probable succinate dehydrogenase (Cytochrome b-556 subunit) SdhC	0.41	0.317	6	8
193	B1MFB9	*MAB_0219*	TED domain-containing protein	0.266	0.316	7	19
194	B1MI44	*MAB_0770*	tRNA_edit domain-containing protein	0.264	0.315	3	10
195	B1MMK1	*MAB_4918c*	Pyruvate dehydrogenase E1 component alpha subunit	0.342	0.312	2	21
196	B1MK14	*MAB_4452*	Putative transcriptional regulator, MerR family	0.429	0.31	2	16
197	B1MCR7	*MAB_2960*	Uncharacterized protein	0.303	0.307	10	39
198	B1MJE8	*MAB_1014c*	Probable glycosyl transferase	0.273	0.307	7	23
199	B1MB16	*MAB_2356*	TPR_5 domain-containing protein	0.563	0.304	12	26
200	B1MN46	*MAB_1673*	DUF559 domain-containing protein	0.454	0.302	2	18
201	B1MJ81	*MAB_0945*	Putative drug resistance transporter, EmrB/QacA family	0.383	0.302	2	14
202	B1MME7	*MAB_4864*	Putative arsenate reductase	0.208	0.301	3	10
203	B1MMV5	*clpP*	ATP-dependent Clp protease proteolytic subunit	1.074	0.298	9	14
204	B1MHV5	*MAB_0680c*	Amidohydro_3 domain-containing protein	0.553	0.296	44	38
205	B1MDE8	*MAB_3192c*	Putative glycosyl hydrolase (Beta-glucosidase)	0.188	0.296	25	27
206	B1MDX9	*MAB_3372*	Putative transcriptional regulator, TetR family	0.178	0.295	1	12
207	B1MBL2	*MAB_2552c*	Putative acetyltransferase	0.178	0.295	1	16
208	B1MMB6	*MAB_4833c*	Uncharacterized protein	0.264	0.293	10	12
209	B1MK15	*MAB_4453c*	YCII domain-containing protein	0.203	0.293	1	5
210	B1MEE4	*MAB_0108c*	Uncharacterized protein	0.649	0.289	12	9
211	B1ML19	*MAB_4595c*	Putative Mce family protein	0.205	0.289	19	23
212	B1MIN6	*MAB_4184c*	Superoxide dismutase [Cu-Zn]	0.339	0.288	9	10
213	B1MMC7	*MAB_4844c*	Putative regulatory protein, ArsR family	0.256	0.287	9	14
214	B1MIA2	*MAB_0830*	Probable NADH-dependent flavin oxidoreductase	0.686	0.286	11	18
215	B1MFC2	*MAB_0222c*	Putative DNA-binding protein	0.315	0.286	7	15
216	B1MP43	*sepF*	Cell division protein SepF	0.295	0.286	9	19
217	B1MJI2	*MAB_1048c*	Probable class II aldolase	0.132	0.284	14	14
218	B1MEQ0	*MAB_0214c*	Uncharacterized protein	0.102	0.284	3	8
219	B1MMI6	*MAB_4903*	Uncharacterized protein	0.36	0.283	3	7
220	B1MDE1	*MAB_3185*	Uncharacterized protein	0.69	0.281	4	12
221	B1MAG8	*MAB_2158c*	Probable ATP-dependent RNA helicase	0.48	0.28	9	31
222	B1MEP0	*MAB_0204c*	Bacterial proteasome activator	0.604	0.278	7	12
223	B1MPB0	*MAB_2080*	Probable medium chain fatty-acid-CoA ligase FadD	0.659	0.272	16	33
224	B1MIS7	*ackA*	Acetate kinase	0.186	0.271	19	21
225	B1MDT9	*MAB_3332c*	Uncharacterized protein	0.369	0.27	9	11
226	B1MLF3	*MAB_1292c*	Probable O-methyltransferase OMT	0.175	0.27	10	18
227	B1MGZ8	*MAB_0584*	Acyl-CoA_dh_2 domain-containing protein	0.59	0.269	23	20
228	B1MGG8	*MAB_3839c*	Putative transcriptional regulator, AsnC family	0.603	0.267	6	11
229	B1MM39	*MAB_4756c*	Monooxygenase, FAD-binding	0.531	0.267	14	30
230	B1MJC1	*MAB_0985*	Putative monooxygenase EthA	0.145	0.267	46	32
231	B1MJ21	*MAB_0883c*	Uncharacterized protein	0.418	0.266	16	17
232	B1MK68	*trmB*	tRNA (guanine-N(7)-)-methyltransferase	0.976	0.264	9	17
233	B1MHG4	*MAB_3971*	Uncharacterized protein	0.369	0.264	3	4
234	B1MP80	*MAB_2049c*	Probable ferredoxin	0.192	0.264	2	2
235	B1MDW3	*mnmA*	tRNA-specific 2-thiouridylase MnmA	0.35	0.263	12	22

Table S4. Positively enriched proteins captured by VM055*p* probe from *M. abscessus* S culture through CC-ABPP by LC-ESI-MS/MS analysis, compared to DMSO-treated cells (*i.e*., non-specific conditions, NS)

	Protein IDs	Gene names	Protein names	VM055*p* *vs*. NS -LOG(p-value)	VM055*p* *vs*. NS Fold Change (Log2)	peptides counts all	nb Tryptic Peptides
236	B1MLH9	*MAB_1318c*	Probable catechol-o-methyltransferase	0.318	0.263	7	19
237	B1MBX1	*MAB_2662*	Probable peroxidoxin BcpB	0.295	0.263	10	14
238	B1MKH6	*MAB_1180c*	Uncharacterized protein	0.213	0.263	12	10
239	B1MID1	*MAB_0859*	Uncharacterized protein	0.16	0.262	4	13
240	B1MMS9	*MAB_1556*	Uncharacterized protein	0.528	0.261	4	9
241	B1MAJ7	*tatA*	Sec-independent protein translocase protein TatA	0.378	0.261	2	4
242	B1MD79	*MAB_3123*	Putative acyl-CoA dehydrogenase	0.929	0.26	13	20
243	B1MJC0	*MAB_0984c*	Putative transcriptional regulator, TetR family	0.347	0.26	13	14
244	B1MNA5	*MAB_1721*	Histidine kinase	0.316	0.259	10	21
245	B1MAZ6	*argC*	N-acetyl-gamma-glutamyl-phosphate reductase	0.202	0.257	12	19
246	B1MIN7	*MAB_4185c*	Putative tuberculin related peptide	0.221	0.255	8	10
247	B1MJ23	*MAB_0885c*	Hypothetical lipoprotein lpqH	0.539	0.254	5	5
248	B1MGD6	*rplX*	50S ribosomal protein L24	0.523	0.254	6	6
249	B1MDY4	*MAB_3377*	Uncharacterized protein	0.54	0.253	3	12
250	B1MMN1	*MAB_1507*	PknH_C domain-containing protein	0.118	0.253	3	10
251	B1MFI5	*MAB_0285*	Putative oxidoreductase	0.448	0.252	7	20
252	B1MC39	*MAB_2731*	Putative transcriptional regulator, TetR family	0.442	0.252	7	17
253	B1MFZ4	*MAB_3665*	Uncharacterized protein	0.711	0.25	7	9
254	B1MAY2	*rpmI*	50S ribosomal protein L35	0.328	0.25	2	4
255	B1MGS4	*MAB_0509c*	Aminopeptidase N	0.213	0.25	16	23
256	B1ME28	*MAB_3421*	Uncharacterized protein	0.366	0.249	9	23
257	B1MGQ5	*MAB_0490c*	Putative adenylate cyclase	0.335	0.249	15	32
258	B1MKA9	*MAB_1113c*	Putative_PNPOx domain-containing protein	0.196	0.249	6	9
259	B1MEZ2	*MAB_3522c*	Assimilatory sulfite reductase (ferredoxin)	0.182	0.247	18	49
260	B1MPB4	*MAB_2084*	Alcohol dehydrogenase	0.09	0.247	22	17
261	B1MC55	*MAB_2747c*	Probable conserved ABC transporter, ATP-binding protein	0.368	0.246	14	18
262	B1MN52	*MAB_1679c*	Probable ArsR-family transcriptional regulator	0.337	0.246	3	8
263	B1MI96	*MAB_0824*	Putative L-carnitine dehydratase	0.34	0.245	12	24
264	B1MFR0	*MAB_0361*	PknH_C domain-containing protein	0.316	0.245	2	12
265	B1MCX1	*MAB_3014*	Putative FAD-dependent pyridine nucleotide-disulphide oxidoreductase, similar to mercuric reductases protein	0.207	0.244	23	24
266	B1MAT2	*MAB_2272c*	Putative transcriptional regulator, GntR	0.413	0.243	7	17
267	B1MMP5	*MAB_1521*	HisKA_3 domain-containing protein	0.691	0.242	3	13
268	B1MF67	*MAB_3598c*	Putative alkane-1-monooxygenase AlkB (Fatty acid omega-hydroxylase)	0.371	0.242	7	17
269	B1MLD7	*MAB_4714c*	Probable fatty-acid-coa ligase FadD	0.255	0.24	66	74
270	B1MDJ8	*ftsY*	Signal recognition particle receptor FtsY	0.246	0.239	13	23
271	B1ML20	*MAB_4596c*	Putative Mce family protein	0.196	0.239	18	28
272	B1MJ55	*MAB_0917c*	Probable cytochrome P450	0.491	0.237	17	25
273	B1MNQ0	*MAB_1868c*	Amidohydro_3 domain-containing protein	0.145	0.234	8	26
274	B1MD63	*MAB_3107c*	Possible lipoprotein LppU	0.187	0.228	4	8
275	B1MIU4	*metZ*	O-succinylhomoserine sulfhydrylase	0.412	0.227	27	24
276	B1MP21	*MAB_1990*	Uncharacterized protein	0.231	0.227	6	13
277	B1MBA9	*MAB_2449*	Bac_luciferase domain-containing protein	0.193	0.226	10	13
278	B1MGI8	*MAB_3859c*	Possible enoyl-CoA hydratase/isomerase	1.306	0.225	11	19
279	B1ME32	*MAB_3425c*	Putative cobalamin synthesis protein	0.418	0.225	9	17
280	B1MLN0	*MAB_1371*	Conserved hypothetical transmembrane protein	0.282	0.225	5	8
281	B1ML12	*MAB_4588c*	Probable enoyl-CoA hydratase/isomerase	0.277	0.224	17	16
282	B1MDR8	*MAB_3311c*	CMD domain-containing protein	0.243	0.224	2	7

Table S4. Positively enriched proteins captured by VM055*p* probe from *M. abscessus* S culture through CC-ABPP by LC-ESI-MS/MS analysis, compared to DMSO-treated cells (*i.e*., non-specific conditions, NS)

	Protein IDs	Gene names	Protein names	VM055*p* *vs*. NS -LOG(p-value)	VM055*p* *vs*. NS Fold Change (Log2)	peptides counts all	nb Tryptic Peptides
283	B1MN36	*MAB_1663*	Heme chaperone HemW	0.403	0.223	8	21
284	B1MCZ0	*MAB_3033*	Uncharacterized protein	0.257	0.223	6	20
285	B1MFY8	*MAB_3659c*	Peptidase M20 domain-containing protein 2	0.301	0.222	10	14
286	B1MAX6	*MAB_2316*	Probable acid-CoA ligase	0.119	0.221	10	22
287	B1ML35	*MAB_4611c*	Putative transcriptional regulator	0.187	0.22	12	17
288	B1MJS0	*MAB_4355*	Hypothetical fumarylacetoacetate (FAA) hydrolase family	0.523	0.219	7	18
289	B1MH40	*MAB_0626*	4-hydroxy-2-oxovalerate aldolase 1	0.342	0.219	12	20
290	B1MG03	*MAB_3674*	Probable succinate dehydrogenase, hydrophobic membrane anchor protein SdhD	0.439	0.218	14	10
291	B1MJR8	*MAB_4353*	4HBT domain-containing protein	0.547	0.217	5	9
292	B1MJA0	*MAB_0964*	Uncharacterized protein	0.267	0.217	13	10
293	B1MF26	*MAB_3556*	Putative hydrolase, alpha/beta fold	0.06	0.216	8	18
294	B1MFR1	*MAB_0362c*	N-acetylglucosamine-6-phosphate deacetylase NagA	0.441	0.215	8	13
295	B1MJE4	*MAB_1010c*	Putative MCE family protein	0.207	0.215	7	28
296	B1MCF5	*MAB_2847c*	Uncharacterized protein	0.421	0.214	7	27
297	B1MKW3	*MAB_4539c*	Putative acyl-CoA carboxylase alpha subunit AccA	0.694	0.213	23	42
298	B1MD49	*MAB_3093c*	Uncharacterized protein	0.171	0.213	3	11
299	B1MCE7	*MAB_2839*	Uncharacterized protein	0.098	0.212	2	8
300	B1MEZ8	*MAB_3528c*	Uncharacterized protein	0.356	0.211	4	7
301	B1MC53	*MAB_2745c*	Possible SUF system FeS assembly protein	0.371	0.21	4	9
302	B1MCY9	*MAB_3032*	Probable soluble pyridine nucleotide transhydrogenase	0.251	0.208	30	31
303	B1MKC5	*MAB_1129*	Probable deoxyribonuclease TatD	0.207	0.208	20	22
304	B1MDF2	*rpsB*	30S ribosomal protein S2	0.335	0.207	27	17
305	B1MKJ2	*MAB_1196*	Proline-rich antigen (36 kDa antigen)	0.215	0.206	9	6
306	B1MIX8	*MAB_4276c*	Probable conserved lipoprotein DsbF	0.307	0.205	8	12
307	B1MER1	*cobB*	NAD-dependent protein deacylase	0.292	0.205	15	14
308	B1MBQ5	*MAB_2595*	Putative pyridoxamine 5'-phosphate oxidase	0.351	0.204	5	12
309	B1MCE6	*MAB_2838c*	Putative cytoplasmic peptidase PepQ	0.341	0.202	13	16
310	B1MBY1	*MAB_2672c*	Quinolinate phosphoribosyltransferase [decarboxylating]	0.262	0.202	15	17
311	B1MIW2	*pyrE*	Orotate phosphoribosyltransferase	0.146	0.201	4	13
312	B1MHY7	*purF*	Amidophosphoribosyltransferase	0.369	0.2	20	31
313	B1MM90	*MAB_4807*	Bacteriophage protein	0.458	0.197	4	15
314	B1MBT6	*MAB_2627c*	Possible two-component response regulatory protein	0.162	0.197	11	12
315	B1MAM3	*MAB_2213*	Putative thioesterase	0.165	0.193	8	15
316	B1MAE2	*MAB_p09*	Probable FAD-dependent pyridine nucleotide-disulphide oxidoreductase	0.384	0.192	21	30
317	B1MDN7	*rpmB*	50S ribosomal protein L28	0.278	0.192	8	5
318	B1MIY7	*MAB_4285*	Thioredoxin domain-containing protein	0.227	0.192	3	15
319	B1MBJ8	*MAB_2538c*	Uncharacterized protein	0.765	0.19	8	22
320	B1MBP3	*MAB_2583c*	Putative transcription regulator, AraC family	0.043	0.189	4	10
321	B1MK58	*MAB_4496c*	Luciferase-like monooxygenase	0.654	0.188	13	12
322	B1MNX6	*MAB_1945c*	Dihydrolipoamide acetyltransferase component of pyruvate dehydrogenase complex	0.182	0.183	26	22
323	B1MGF5	*MAB_3825*	Cytochrome P450	0.245	0.182	8	25
324	B1MCD6	*carA*	Carbamoyl-phosphate synthase small chain	0.32	0.181	17	18
325	B1MIU7	*MAB_4245c*	Phosphoribosylglycinamide formyltransferase 2	0.287	0.181	28	30
326	B1MKY4	*MAB_4560*	Alcohol dehydrogenase	0.168	0.181	22	20
327	B1MGX5	*MAB_0561*	Uncharacterized protein	0.131	0.181	14	7
328	B1MAW2	*MAB_2302*	Probable conserved membrane protein, MmpS	0.101	0.18	7	10
329	B1MK67	*MAB_4505c*	Uncharacterized protein	0.436	0.179	12	21

Table S4. Positively enriched proteins captured by VM055*p* probe from *M. abscessus* S culture through CC-ABPP by LC-ESI-MS/MS analysis, compared to DMSO-treated cells (*i.e* ., non-specific conditions, NS)

	Protein IDs	Gene names	Protein names	VM055*p* *vs* . NS		peptides counts all	nb Tryptic Peptides
				-LOG(p-value)	Fold Change (Log2)		
330	B1MK61	*MAB_4499*	Histidine kinase	0.372	0.178	9	35
331	B1MEE3	*MAB_0107c*	4-hydroxy-4-methyl-2-oxoglutarate aldolase	0.255	0.178	8	5
332	B1MD35	*MAB_3078*	Putative transcriptional regulator, TetR family	0.475	0.177	6	15
333	B1ML69	*MAB_4645*	Uncharacterized protein	0.206	0.176	1	4
334	B1MKU3	*MAB_4519c*	Putative two-component system response regulator,LuxR family	0.195	0.176	3	17
335	B1MG16	*MAB_3687*	Probable o-acetylhomoserine sulfhydrylase MetC (Homocysteine synthase)	0.154	0.176	10	20
336	B1MFH7	*MAB_0277c*	Probable amino acid ABC transporter, permease	0.183	0.175	11	30
337	B1MLN4	*MAB_1375*	Probable sugar ABC transporter, ATP-binding protein SugC	0.376	0.174	11	21
338	B1MD55	*MAB_3099c*	Putative transcriptional regulator, AsnC family	0.208	0.174	3	12
339	B1MCD8	*pyrC*	Dihydroorotase	0.148	0.173	27	22
340	B1MFW7	*nth*	Endonuclease III	0.235	0.172	16	20
341	B1MCB2	*MAB_2804c*	Putative ABC-type transporter, periplasmic component	0.105	0.172	10	19
342	B1MGB3	*adk*	Adenylate kinase	0.293	0.171	12	14
343	B1MDK5	*MAB_3248c*	Uncharacterized protein	1.058	0.17	16	9
344	B1MBV2	*trpA*	Tryptophan synthase alpha chain	0.257	0.17	9	15
345	B1MF87	*purE*	N5-carboxyaminoimidazole ribonucleotide mutase	0.235	0.17	3	7
346	B1MJ65	*MAB_0929*	Uncharacterized protein	0.224	0.17	28	26
347	B1MCJ7	*MAB_2889*	Uncharacterized protein	0.133	0.17	2	14
348	B1MEK1	*MAB_0165*	Putative acyltransferase	0.334	0.168	20	16
349	B1MKI4	*MAB_1188c*	Probable acyl-CoA dehydrogenase	0.306	0.167	31	29
350	B1MPB3	*MAB_2083*	SCP2 domain-containing protein	0.23	0.167	15	13
351	B1MCE9	*aroB*	3-dehydroquinate synthase	0.154	0.166	11	25
352	B1MBY7	*MAB_2678*	FAD_binding_3 domain-containing protein	0.101	0.166	9	23
353	B1MDE4	*pyrH*	Uridylate kinase	0.313	0.165	15	16
354	B1MJL3	*dcd*	dCTP deaminase, dUMP-forming	0.233	0.164	6	12
355	B1MHA5	*MAB_3912*	UPF0234 protein MAB_3912	0.161	0.164	9	14
356	B1MIL6	*MAB_4164*	Possible enoyl-CoA hydratase	0.232	0.163	5	19
357	B1MBG3	*MAB_2503c*	Putative iron-sulfur binding oxidoreductase	0.127	0.163	2	30
358	B1MDI7	*MAB_3230c*	SnoaL-like domain-containing protein	0.053	0.162	3	6
359	B1ME65	*MAB_0027c*	Putative S-adenosyl-L-methionine-dependent methyltransferase MAB_0027c	0.099	0.16	4	19
360	B1MPB5	*MAB_2085*	Probable acyl CoA dehydrogenase	0.956	0.159	19	25
361	B1MMP9	*MAB_1525c*	Putative transcriptional regulator, TetR family	0.273	0.159	5	13
362	B1MG80	*rpsI*	30S ribosomal protein S9	0.268	0.158	8	12
363	B1MJX1	*MAB_4408c*	Alkyl hydroperoxide reductase C	0.233	0.158	21	13
364	B1MCI9	*MAB_2881c*	Uncharacterized protein	0.14	0.157	3	5
365	B1MGI9	*MAB_3860c*	Probable acyl-CoA dehydrogenase FadE	0.355	0.156	54	33
366	B1MC14	*MAB_2706c*	Putative transporter	0.298	0.156	3	13
367	B1MGH0	*MAB_3841*	Ornithine--oxo-acid aminotransferase	0.217	0.156	10	22
368	B1MNE1	*MAB_1757*	Bacteriophage protein	0.215	0.156	7	6
369	B1MM04	*MAB_1495*	Probable oxidoreductase	0.161	0.156	37	42
370	B1MIH9	*MAB_4127c*	Dihydrolipoyl dehydrogenase	0.218	0.155	31	30
371	B1MKW9	*ilvD*	Dihydroxy-acid dehydratase	0.188	0.155	67	38
372	B1MEU1	*MAB_3471*	Succinate-semialdehyde dehydrogenase	0.16	0.155	12	26
373	B1MCF8	*MAB_2850c*	Putative pre-16S rRNA nuclease	0.777	0.154	7	14
374	B1MFV1	*MAB_0402*	SGNH_hydro domain-containing protein	0.112	0.154	5	17
375	B1MC29	*cpfC*	Coproporphyrin III ferrochelatase	0.605	0.153	19	19
376	B1MEV8	*MAB_3488*	Uncharacterized protein	0.184	0.153	5	11

Table S4. Positively enriched proteins captured by VM055*p* probe from *M. abscessus* S culture through CC-ABPP by LC-ESI-MS/MS analysis, compared to DMSO-treated cells (*i.e* ., non-specific conditions, NS)

	Protein IDs	Gene names	Protein names	VM055*p* *vs* . NS		peptides counts all	nb Tryptic Peptides
				-LOG(p-value)	Fold Change (Log2)		
377	B1MCT4	*MAB_2977*	Abhydrolase_4 domain-containing protein	0.108	0.153	15	25
378	B1MJU1	*MAB_4377c*	Probable fatty-acid-CoA ligase	0.099	0.153	7	36
379	B1MAM5	*MAB_2215*	Uncharacterized protein	0.148	0.152	4	8
380	B1ME74	*MAB_0036c*	Probable cell division protein RodA	0.314	0.151	8	19
381	B1ML09	*MAB_4585c*	Putative S-adenosyl-L-methionine-dependent methyltransferase MAB_4585c	0.294	0.151	21	20
382	B1MLZ2	*MAB_1483*	Lactamase_B domain-containing protein	0.129	0.151	8	9
383	B1MDI1	*rplS*	50S ribosomal protein L19	0.119	0.15	14	9
384	B1MLF1	*MAB_4728c*	DNA-(apurinic or apyrimidinic site) lyase	0.106	0.15	6	21
385	B1MHK5	*MAB_4012c*	Uncharacterized protein	0.224	0.149	18	13
386	B1MFE4	*MAB_0244*	Uncharacterized protein	0.119	0.149	22	15
387	B1MC65	*MAB_2757*	Probable quinone reductase Qor	0.094	0.148	10	16
388	B1MLY1	*MAB_1472c*	Putative nicotinamidase/pyrazinamidase	0.089	0.148	2	11
389	B1MML8	*MAB_4935*	MutT/NUDIX family protein	0.174	0.147	4	16
390	B1MGV5	*MAB_0540*	Uncharacterized protein	0.235	0.146	7	13
391	B1MLS6	*MAB_1417*	Putative lipoprotein LprC	0.101	0.145	8	13
392	B1MLG1	*MAB_1300c*	Uncharacterized protein	0.136	0.144	2	20
393	B1MCY6	*MAB_3029*	Iron-dependent repressor IdeR	0.119	0.144	23	14
394	B1MII8	*MAB_4136c*	Aldehyde dehydrogenase	0.548	0.143	13	30
395	B1MGD0	*MAB_3800c*	Glutamate dehydrogenase	0.162	0.143	33	34
396	B1MMI1	*MAB_4898c*	Single-stranded DNA-binding protein	0.098	0.143	11	12
397	B1ML25	*MAB_4601c*	Putative YrbE family protein	0.071	0.143	10	11
398	B1MMJ3	*MAB_4910c*	Putative aminoglycoside phosphotransferase	0.158	0.142	4	13
399	B1MG35	*MAB_3706*	Polbeta domain-containing protein	0.133	0.141	4	18
400	B1MMV9	*MAB_1586c*	Uncharacterized protein	0.106	0.141	5	6
401	B1MMK8	*MAB_4925*	Uncharacterized protein	0.088	0.141	2	4
402	B1MI20	*MAB_0746*	Phosphate-binding protein PstS	0.134	0.14	18	23
403	B1MID5	*MAB_4083c*	Heparin-binding hemagglutinin (Adhesin)	0.622	0.139	14	15
404	B1MAG3	*MAB_2153*	MOSC domain-containing protein	0.264	0.139	3	16
405	B1MAQ5	*MAB_2245*	Putative anthranilate synthase component I TrpE2/ Salicylate synthase MbtI	0.183	0.139	23	35
406	B1MBK0	*MAB_2540c*	Putative Short-chain dehydrogenase/reductase	0.172	0.139	8	25
407	B1MPD2	*MAB_2102*	Probable peptidase	0.252	0.138	20	29
408	B1MJ04	*MAB_0866*	Probable molybdopterin-converting factor subunit 2 (MoaE)	0.206	0.137	6	12
409	B1ML32	*MAB_4608*	Beta-ketoacyl-[acyl-carrier-protein] synthase I	0.2	0.137	37	21
410	B1MJX9	*MAB_4416c*	Uncharacterized protein	0.123	0.137	3	5
411	B1MLC7	*MAB_4704c*	Probable membrane protein, MmpL	0.111	0.137	7	52
412	B1MJ60	*MAB_0924c*	Putative organic hydroperoxide resistance protein/OsmC-like protein	0.339	0.135	8	8
413	B1MGD9	*MAB_3809c*	Probable cutinase Cut4	0.236	0.134	12	12
414	B1MKS2	*MAB_1276c*	Uncharacterized protein	0.221	0.134	3	9
415	B1MM34	*MAB_4751*	PNPLA domain-containing protein	0.149	0.134	16	19
416	B1MJL6	*MAB_4300*	Putative hydrolase, alpha/beta fold	0.078	0.134	10	15
417	B1MHK8	*mca*	Mycothiol S-conjugate amidase	0.185	0.133	5	16
418	B1MAW1	*MAB_2301*	Putative membrane protein, mmpL	0.1	0.133	22	55
419	B1MJ93	*MAB_0957*	Uncharacterized protein	0.107	0.131	5	6
420	B1MMB5	*MAB_4832c*	Uncharacterized protein	0.227	0.13	3	11
421	B1MEH7	*MAB_0141c*	Putative esterase	0.073	0.13	3	22
422	B1MJS9	*MAB_4365c*	Putative dihydrodiol dehydrogenase	0.649	0.129	4	18
423	B1MH28	*MAB_0614*	Probable acyl-CoA dehydrogenase FadE	0.253	0.129	14	20

Table S4. Positively enriched proteins captured by VM055*p* probe from *M. abscessus* S culture through CC-ABPP by LC-ESI-MS/MS analysis, compared to DMSO-treated cells (*i.e*., non-specific conditions, NS)

	Protein IDs	Gene names	Protein names	VM055*p* *vs*. NS		peptides counts all	nb Tryptic Peptides
				-LOG(p-value)	Fold Change (Log2)		
424	B1MAI1	*pup*	Prokaryotic ubiquitin-like protein Pup	0.043	0.129	6	4
425	B1MLV5	*MAB_1446*	Uncharacterized protein	0.162	0.128	2	4
426	B1MDX0	*MAB_3363c*	Electron transfer flavoprotein beta-subunit FixA	0.139	0.127	20	20
427	B1MIH8	*MAB_4126c*	Carbonic anhydrase-related protein	0.378	0.126	4	13
428	B1MCT0	*MAB_2973c*	Putative methyltransferase	0.278	0.126	27	19
429	B1MJ99	*MAB_0963c*	Putative polyketide synthase protein	0.25	0.126	9	16
430	B1ML45	*MAB_4621c*	Putative acetyltransferase	0.126	0.126	3	8
431	B1MMA4	*MAB_4821c*	Uncharacterized protein	0.094	0.126	1	5
432	B1MCC5	*MAB_2817c*	Putative dihydropyrimidinase	0.099	0.125	13	28
433	B1MGI0	*rpsL*	30S ribosomal protein S12	0.57	0.124	6	9
434	B1MGE1	*rpsQ*	30S ribosomal protein S17	0.33	0.124	19	8
435	B1MJR4	*MAB_4349c*	DUF1989 domain-containing protein	0.246	0.124	6	10
436	B1MFT5	*MAB_0386c*	Conserved hypothetical membrane protein	0.097	0.124	2	13
437	B1MG55	*whiB*	Transcriptional regulator WhiB	0.083	0.124	6	5
438	B1MH77	*MAB_3884*	Possible flavoprotein	0.254	0.122	21	32
439	B1MEA0	*MAB_0064c*	Probable lipase LipE	0.203	0.121	26	26
440	B1MJZ8	*MAB_4435*	Non-specific serine/threonine protein kinase	0.178	0.121	12	33
441	B1MEF1	*MAB_0115c*	Uncharacterized protein	0.172	0.121	20	12
442	B1MJM7	*MAB_4312*	Putative TetR-family transcriptional regulator	0.117	0.121	2	13
443	B1MBX2	*hisI*	Phosphoribosyl-AMP cyclohydrolase	0.076	0.121	4	4
444	B1MEZ6	*MAB_3526c*	ZnMc domain-containing protein	0.113	0.12	15	22
445	B1MM47	*MAB_4764c*	Uncharacterized protein	0.109	0.12	10	23
446	B1MLI8	*MAB_1327*	Ferredoxin	0.047	0.12	6	4
447	B1MLG5	*MAB_1304*	Uncharacterized protein	0.155	0.118	4	19
448	B1MNT5	*MAB_1903*	GTP cyclohydrolase 1 type 2 homolog	0.126	0.118	14	16
449	B1MG68	*MAB_3739c*	Alanine racemase	0.41	0.117	9	18
450	B1MEY6	*MAB_3516c*	DNA helicase	0.158	0.117	17	66
451	B1MI61	*MAB_0788*	Uncharacterized protein	0.094	0.117	3	14
452	B1MIH1	*MAB_4119*	CMD domain-containing protein	0.132	0.116	14	14
453	B1MFW9	*MAB_0420*	Nudix hydrolase domain-containing protein	0.098	0.116	4	13
454	B1MBV3	*trpB*	Tryptophan synthase beta chain	0.458	0.115	28	23
455	B1MB78	*MAB_2418*	Putative phenylacetic acid degradation-related protein	0.11	0.114	6	5
456	B1MD86	*rbfA*	Ribosome-binding factor A	0.098	0.114	3	10
457	B1MK13	*MAB_4451c*	Uncharacterized protein	0.227	0.113	1	17
458	B1MAI3	*prcA*	Proteasome subunit alpha	0.176	0.113	14	17
459	B1MIU6	*MAB_4244c*	Uncharacterized protein	0.065	0.113	7	13
460	B1MJJ3	*MAB_1059c*	Bac_luciferase domain-containing protein	0.426	0.112	18	20
461	B1MDJ3	*MAB_3236c*	Probable amidohydrolase	0.063	0.111	4	16
462	B1MCV2	*MAB_2995*	Trk system potassium uptake protein TrkA	0.163	0.11	11	16
463	B1MFB4	*MAB_3646c*	Lysine 6-aminotransferase	0.091	0.11	20	30
464	B1MEH8	*MAB_0142c*	Putative monooxygenase	0.037	0.11	4	30
465	B1ML67	*MAB_4643c*	Amidase family protein	0.213	0.109	17	26
466	B1MGW1	*MAB_0546*	Probable ATP-dependent Clp protease ATP-binding subunit	0.134	0.109	69	57
467	B1MEB7	*MAB_0081*	Putative 3-ketosteroid 1-dehydrogenase or fumarate reductase/succinate dehydrogenase	0.138	0.108	6	35
468	B1MNS8	*MAB_1896c*	Uncharacterized protein	0.115	0.108	4	8
469	B1MCT8	*MAB_2981c*	Putative liporotein LppU	0.213	0.107	4	9
470	B1MD04	*MAB_3047c*	Uncharacterized protein	0.09	0.107	7	12

Table S4. Positively enriched proteins captured by VM055*p* probe from *M. abscessus* S culture through CC-ABPP by LC-ESI-MS/MS analysis, compared to DMSO-treated cells (*i.e*., non-specific conditions, NS)

	Protein IDs	Gene names	Protein names	VM055*p* *vs*. NS -LOG(p-value)	VM055*p* *vs*. NS Fold Change (Log2)	peptides counts all	nb Tryptic Peptides
471	B1ME98	*MAB_0062*	Pyridine nucleotide-disulphide oxidoreductase family	0.284	0.106	17	29
472	B1MCV4	*MAB_2997c*	RecG_wedge domain-containing protein	0.232	0.106	13	12
473	B1MBX7	*hisB*	Imidazoleglycerol-phosphate dehydratase	0.081	0.106	4	7
474	B1MHK1	*MAB_4008c*	Uncharacterized protein	0.07	0.106	19	10
475	B1MHC6	*menD*	2-succinyl-5-enolpyruvyl-6-hydroxy-3-cyclohexene-1-carboxylate synthase	0.284	0.105	23	25
476	B1MJA6	*MAB_0970c*	Probable drug resistance transporter	0.272	0.105	15	26
477	B1MEQ1	*MAB_0215*	Possible transcriptional regulator	0.073	0.105	5	14
478	B1MBY3	*nadA*	Quinolinate synthase A	0.18	0.104	25	21
479	B1MF57	*MAB_3588*	Putative acyl-CoA oxidase	0.161	0.104	28	41
480	B1MCG4	*MAB_2856c*	zinc-ribbon_6 domain-containing protein	0.091	0.103	13	13
481	B1MFQ6	*MAB_0357c*	Uncharacterized protein	0.043	0.103	4	8
482	B1ML04	*MAB_4580c*	GST C-terminal domain-containing protein	0.304	0.102	25	27
483	B1MGJ9	*MAB_0434c*	Uncharacterized protein	0.266	0.102	12	27
484	B1MKB9	*rsmI*	Ribosomal RNA small subunit methyltransferase I	0.228	0.102	19	17
485	B1MGZ4	*MAB_0580*	Probable acyl-CoA dehydrogenase FadE	0.808	0.1	18	31
486	B1MG17	*metXA*	Homoserine O-acetyltransferase	0.358	0.099	15	21
487	B1MLM3	*MAB_1364*	Probable serine protease HtrA	0.168	0.099	21	32
488	B1MC70	*MAB_2762*	Putative OxpP cycle protein OpcA	0.236	0.098	19	18
489	B1MHS5	*MAB_0649c*	Putative monooxygenase	0.11	0.098	12	29
490	B1MI92	*MAB_0820c*	Glutamate dehydrogenase	0.085	0.098	22	68
491	B1MKH9	*MAB_1183*	Rhodanese domain-containing protein	0.076	0.097	3	8
492	B1MP07	*MAB_1976*	Uncharacterized protein	0.105	0.096	20	24
493	B1MDQ1	*leuC*	3-isopropylmalate dehydratase large subunit	0.369	0.095	50	28
494	B1MH20	*MAB_0606c*	Probable enoyl-CoA hydratase EchA	0.196	0.095	9	15
495	B1MKG1	*eno*	Enolase	0.078	0.095	21	24
496	B1MD51	*MAB_3095c*	Uncharacterized protein	0.042	0.095	9	12
497	B1MCM3	*MAB_2916*	Uncharacterized protein	0.038	0.095	14	32
498	B1MCU9	*MAB_2992c*	TRAM domain-containing protein	0.518	0.094	17	25
499	B1MEE0	*MAB_0104*	Probable enoyl-CoA hydratase/isomerase	0.155	0.094	5	21
500	B1MKQ1	*xseA*	Exodeoxyribonuclease 7 large subunit	0.117	0.094	15	34
501	B1MBF6	*MAB_2496*	Probable acyl-CoA dehydrogenase	0.059	0.094	7	22
502	B1ME37	*MAB_3430c*	Uncharacterized protein	0.035	0.094	5	13
503	B1MGD5	*rplE*	50S ribosomal protein L5	0.43	0.093	19	14
504	B1MGY2	*MAB_0568*	Putative CarD-like transcriptional regulator	0.104	0.093	11	13
505	B1MIR7	*MAB_4215c*	Putative ABC transporter, ATP-binding protein	0.094	0.093	6	23
506	B1ML15	*MAB_4591*	Putative phosphotyrosine protein phosphatase	0.239	0.091	14	19
507	B1MFL6	*MAB_0317*	Uncharacterized protein	0.159	0.091	9	9
508	B1MNW2	*MAB_1930c*	Conserved hypothetical integral membrane protein	0.225	0.09	3	3
509	B1MHL9	*MAB_4026c*	Probable transcriptional regulatory protein TetR	0.113	0.09	8	14
510	B1MH91	*MAB_3898c*	UPF0336 protein MAB_3898c	0.077	0.089	13	10
511	B1MD90	*MAB_3134c*	Uncharacterized protein	0.523	0.088	7	9
512	B1MIC2	*MAB_0850*	Probable acyl-coa thiolase FadA	0.18	0.088	27	19
513	B1MHU6	*MAB_0670*	Uncharacterized protein	0.087	0.087	4	16
514	B1MGV4	*MAB_0539*	Conserved hypothetical transmembrane protein	0.078	0.087	14	19
515	B1ME26	*nadE*	NH(3)-dependent NAD(+) synthetase	0.261	0.086	21	20
516	B1MHJ5	*MAB_4002c*	Putative acyltransferase	0.114	0.086	7	18
517	B1MFZ1	*upp*	Uracil phosphoribosyltransferase	0.304	0.085	8	12

Table S4. Positively enriched proteins captured by VM055*p* probe from *M. abscessus* S culture through CC-ABPP by LC-ESI-MS/MS analysis, compared to DMSO-treated cells (*i.e*., non-specific conditions, NS)

	Protein IDs	Gene names	Protein names	VM055*p* *vs*. NS -LOG(p-value)	VM055*p* *vs*. NS Fold Change (Log2)	peptides counts all	nb Tryptic Peptides
518	B1MNQ7	*MAB_1875c*	Putative hydrolase (Alpha/beta fold)	0.214	0.085	5	14
519	B1MG97	*truA*	tRNA pseudouridine synthase A	0.209	0.084	13	23
520	B1MBW2	*MAB_2653c*	HTH_30 domain-containing protein	0.081	0.084	10	31
521	B1MC45	*MAB_2737c*	Probable enoyl-CoA hydratase/isomerase	0.221	0.083	30	19
522	B1MJP1	*MAB_4326c*	Uncharacterized protein	0.064	0.083	3	7
523	B1MNT6	*MAB_1904*	zf-RING_7 domain-containing protein	0.039	0.083	22	21
524	B1MPG2	*hisG*	ATP phosphoribosyltransferase	0.276	0.082	21	22
525	B1MG98	*rplQ*	50S ribosomal protein L17	0.162	0.082	11	14
526	B1MP84	*MAB_2053*	Luciferase-like	0.093	0.082	22	22
527	B1MC16	*MAB_2708*	Uncharacterized protein	0.073	0.082	6	25
528	B1MI01	*MAB_0727*	Putative dihydrodipicolinate reductase	0.143	0.081	6	14
529	B1MCK5	*MAB_2897c*	HIT domain-containing protein	0.064	0.081	6	12
530	B1MHY9	*MAB_0715c*	Putative HTH-type transcriptional regulator AraC	0.056	0.081	12	26
531	B1MHS3	*MAB_0647*	Possible transcriptional regulatory protein TetR	0.173	0.08	5	13
532	B1MDA1	*MAB_3145*	Uncharacterized protein	0.196	0.079	4	14
533	B1MJ98	*MAB_0962*	Probable cation-transporting ATPase E	0.171	0.079	19	39
534	B1MMQ0	*MAB_1526*	Putative short chain dehydrogenase/reductase	0.139	0.079	17	20
535	B1MFL5	*MAB_0316*	Probable enoyl-CoA hydratase	0.088	0.079	2	17
536	B1MKA0	*MAB_1103*	Uncharacterized protein	0.347	0.078	7	19
537	B1MJV8	*MAB_4394*	AHS2 domain-containing protein	0.105	0.078	6	16
538	B1MEW0	*MAB_3490c*	Uncharacterized protein	0.09	0.078	8	8
539	B1MP39	*ftsQ*	Cell division protein FtsQ	0.195	0.077	11	18
540	B1MCE5	*efp*	Elongation factor P	0.088	0.077	4	10
541	B1MMR0	*MAB_1537c*	Putative short chain dehydrogenase/reductase	0.088	0.077	23	19
542	B1MNW5	*MAB_1933c*	Glutamine synthetase	0.078	0.077	21	26
543	B1MB89	*MAB_2429c*	Probable NADH dehydrogenase (NDH)	0.117	0.076	28	26
544	B1MHK7	*MAB_4014*	Uncharacterized protein	0.191	0.075	22	16
545	B1MCP1	*MAB_2934*	Probable Asp/Glu racemase	0.081	0.075	5	11
546	B1MLB4	*MAB_4691c*	Mycobactin synthetase protein B	0.077	0.075	20	390
547	B1MH11	*MAB_0597*	Probable acyl-CoA dehydrogenase FadE	0.482	0.074	16	25
548	B1MH86	*rplK*	50S ribosomal protein L11	0.139	0.074	13	13
549	B1MB96	*MAB_2436*	Alanine and proline-rich secreted protein Apa	0.048	0.074	3	11
550	B1MCN5	*MAB_2928*	Guanine deaminase	0.312	0.073	15	26
551	B1MKW4	*MAB_4540c*	Putative acetyl-CoA carboxylase beta subunit AccD	0.165	0.073	23	36
552	B1MDQ2	*MAB_3295*	Putative transcriptional regulator, IclR family	0.141	0.073	11	18
553	B1MDI6	*rpsP*	30S ribosomal protein S16	0.127	0.073	13	10
554	B1MHI4	*MAB_3991c*	Possible Uroporphyrin-III C-methyltransferase	0.664	0.07	36	31
555	B1MIV1	*purA*	Adenylosuccinate synthetase	0.123	0.07	29	26
556	B1MN35	*MAB_1662c*	Assimilatory sulfite reductase (ferredoxin)	0.127	0.069	48	37
557	B1MEY4	*MAB_3514c*	Possible transmembrane cation transporter	0.033	0.069	8	22
558	B1MNA3	*MAB_1719*	Uncharacterized protein	0.265	0.068	32	26
559	B1MGI6	*MAB_3857c*	Probable enoyl-coa hydratase/isomerase	0.244	0.068	5	14
560	B1MNL4	*MAB_1832*	NLPC_P60 domain-containing protein	0.127	0.068	16	32
561	B1MJG6	*ku*	Non-homologous end joining protein Ku	0.081	0.068	20	19
562	B1MGG4	*MAB_3835c*	Probable coenzyme PQQ synthesis protein E PqqE	0.055	0.068	20	29
563	B1MM89	*MAB_4806c*	Uncharacterized protein	0.05	0.068	21	26
564	B1MHK9	*MAB_4016c*	Uncharacterized protein	0.049	0.068	1	12

Table S4. **Positively enriched proteins captured by VM055*p* probe from *M. abscessus* S culture through CC-ABPP by LC-ESI-MS/MS analysis, compared to DMSO-treated cells (*i.e*., non-specific conditions, NS)**

	Protein IDs	Gene names	Protein names	VM055*p* *vs*. NS -LOG(p-value)	VM055*p* *vs*. NS Fold Change (Log2)	peptides counts all	nb Tryptic Peptides
565	B1MJK5	*MAB_1071c*	Probable acetyl-/propionyl-CoA carboxylase alpha subunit AccA2	0.314	0.067	26	36
566	B1MK82	*MAB_1084c*	CxxC_CXXC_SSSS domain-containing protein	0.051	0.067	11	7
567	B1MBU1	*MAB_2632*	Probable ATP-binding protein ABC transporter CydD	0.05	0.067	10	30
568	B1MGI3	*MAB_3854*	Uncharacterized protein	0.136	0.066	2	5
569	B1MIZ3	*MAB_4291*	Uncharacterized protein	0.097	0.066	19	18
570	B1MM73	*MAB_4790*	Possible oxidoreductase	0.059	0.066	9	20
571	B1MKZ2	*MAB_4568c*	Putative Mce family protein	0.058	0.066	7	26
572	B1MIT2	*fgd*	F420-dependent glucose-6-phosphate dehydrogenase	0.233	0.065	29	24
573	B1MJ02	*moaC*	Cyclic pyranopterin monophosphate synthase	0.1	0.065	4	13
574	B1MFI3	*MAB_0283c*	Uncharacterized protein	0.169	0.064	7	13
575	B1MFN6	*leuA*	2-isopropylmalate synthase	0.083	0.064	52	34
576	B1MEF9	*MAB_0123*	Putative glycerophosphoryl diester phosphodiesterase	0.144	0.063	15	19
577	B1MKT7	*MAB_1291*	Uncharacterized protein	0.056	0.063	10	16
578	B1MMW5	*fdhD*	Sulfur carrier protein FdhD	0.158	0.062	11	17
579	B1MDL7	*MAB_3260c*	Uncharacterized protein	0.112	0.062	2	4
580	B1MF80	*MAB_3611c*	Putative sugar-phosphate nucleotidyl transferase	0.099	0.062	28	26
581	B1MB36	*MAB_2376c*	Uncharacterized protein	0.068	0.061	12	24
582	B1MEC4	*MAB_0088c*	Putative oxidoreductase	0.058	0.061	19	20
583	B1MB62	*gcvH*	Glycine cleavage system H protein	0.024	0.061	1	3
584	B1MIQ0	*MAB_4198*	Uncharacterized protein	0.339	0.06	10	14
585	B1MMS0	*MAB_1547c*	Probable sulfatase	0.084	0.059	12	25
586	B1MPB2	*MAB_2082*	Uncharacterized protein	0.063	0.058	13	26
587	B1MCU2	*MAB_2985c*	Coproporphyrinogen III oxidase	0.09	0.057	19	26
588	B1MGA8	*MAB_3778*	Bac_luciferase domain-containing protein	0.084	0.057	7	15
589	B1MP66	*MAB_2035*	Acyltransferase PapA5	0.063	0.057	4	28
590	B1MKL2	*MAB_1216c*	Probable cytochrome P450	0.04	0.057	6	32
591	B1MB72	*MAB_2412c*	Probable IMP dehydrogenase family protein	0.323	0.056	32	27
592	B1MI52	*MAB_0778*	Uncharacterized protein	0.063	0.056	8	12
593	B1MFX0	*MAB_0421*	Possible membrane-associated serine protease	0.035	0.056	12	20
594	B1MB31	*cmk*	Cytidylate kinase	0.089	0.055	6	13
595	B1MMZ0	*rbsK*	Ribokinase	0.129	0.054	12	15
596	B1MNZ4	*MAB_1963*	Putative membrane protein, MmpS family	0.065	0.054	2	7
597	B1MHW2	*MAB_0687*	Adenylosuccinate lyase	0.054	0.054	42	32
598	B1MMU5	*MAB_1572*	DSBA domain-containing protein	0.056	0.053	15	17
599	B1MHK3	*MAB_4010c*	Uncharacterized protein	0.146	0.052	8	13
600	B1MK84	*MAB_1086*	UTP--glucose-1-phosphate uridylyltransferase	0.088	0.051	15	20
601	B1MAS0	*MAB_2260*	Putative formyltransferase	0.061	0.051	14	23
602	B1MGC6	*rplR*	50S ribosomal protein L18	0.054	0.051	7	11
603	B1MB80	*MAB_2420c*	PknH_C domain-containing protein	0.096	0.05	14	16
604	B1MIC4	*MAB_0852*	Possible conserved polyketide synthase associated protein PapA2	0.076	0.05	11	25
605	B1MEX4	*MAB_3504*	Putative ABC transporter, ATP-binding protein	0.052	0.05	23	36
606	B1MNV2	*MAB_1920*	Glutamine synthetase	0.102	0.049	21	23
607	B1MLQ7	*MAB_1398c*	Putative oxidoreductase	0.1	0.049	22	28
608	B1MCZ3	*nrdR*	Transcriptional repressor NrdR	0.088	0.049	9	15
609	B1MLA1	*MAB_4678*	Putative_PNPOx domain-containing protein	0.069	0.049	5	11
610	B1MAF5	*MAB_2145*	NADH-quinone oxidoreductase, L subunit NuoL	0.049	0.049	4	18
611	B1MKL0	*MAB_1214c*	Probable cytochrome P450	0.036	0.049	8	31

Table S4. Positively enriched proteins captured by VM055*p* probe from *M. abscessus* S culture through CC-ABPP by LC-ESI-MS/MS analysis, compared to DMSO-treated cells (*i.e*., non-specific conditions, NS)

	Protein IDs	Gene names	Protein names	VM055*p* *vs*. NS -LOG(p-value)	VM055*p* *vs*. NS Fold Change (Log2)	peptides counts all	nb Tryptic Peptides
612	B1MIQ6	*MAB_4204*	Amine oxidase	0.034	0.049	74	38
613	B1MJL0	*MAB_1076*	Mycobacterial persistence regulator MrpA (Two component response transcriptional regulatory protein)	0.075	0.048	8	12
614	B1MH39	*MAB_0625*	Acetaldehyde dehydrogenase 1	0.058	0.048	11	16
615	B1ML01	*MAB_4577c*	NAD(P) transhydrogenase subunit beta	0.052	0.048	17	18
616	B1MJX8	*MAB_4415*	Probable amidase	0.049	0.048	5	18
617	B1MH73	*MAB_3880*	Probable lipase/esterase LipG	0.042	0.048	14	19
618	B1MDV9	*MAB_3352c*	Putative methionine synthase, vitamin-B12 independent	0.198	0.047	10	20
619	B1MKZ9	*MAB_4575*	Probable low temperature requirement protein A	0.162	0.046	7	15
620	B1MH75	*MAB_3882c*	Glycerol-3-phosphate dehydrogenase	0.132	0.046	17	34
621	B1MBG1	*MAB_2501*	Uncharacterized protein	0.118	0.046	6	8
622	B1MGZ7	*MAB_0583c*	Putative oxidoreductase	0.034	0.046	10	21
623	B1MIV5	*MAB_4253*	Uncharacterized protein	0.033	0.046	11	17
624	B1MKR2	*ychF*	Ribosome-binding ATPase YchF	0.128	0.045	24	25
625	B1MIS1	*thiE*	Thiamine-phosphate synthase	0.084	0.045	10	16
626	B1MM07	*MAB_1498c*	Putative GntR-family transcriptional regulator	0.069	0.045	6	21
627	B1MIF9	*MAB_4107c*	Glycosyltransferase GtfA	0.054	0.045	18	19
628	B1MJZ0	*MAB_4427c*	Putative transcriptional regulator	0.054	0.045	6	13
629	B1MNY4	*cobT*	Nicotinate-nucleotide--dimethylbenzimidazole phosphoribosyltransferase	0.044	0.045	9	15
630	B1MM20	*MAB_4737*	Putative citrate lyase/aldolase	0.108	0.044	16	19
631	B1MKG2	*MAB_1166*	Uncharacterized protein	0.035	0.044	7	13
632	B1MFF8	*MAB_0258c*	Putative dihydropicolinate reductase	0.133	0.043	14	11
633	B1MLI9	*MAB_1328*	Aminotransferase	0.112	0.043	16	21
634	B1MEI7	*MAB_0151c*	Septum_form domain-containing protein	0.087	0.043	16	21
635	B1MC96	*MAB_2788*	Probable gamma-glutamyltranspeptidase (GgtB)	0.064	0.043	17	29
636	B1MDP8	*MAB_3291*	Possible hydrolase MutT/NUDIX	0.05	0.043	20	22
637	B1MDC5	*ispG*	4-hydroxy-3-methylbut-2-en-1-yl diphosphate synthase (flavodoxin)	0.024	0.043	20	24
638	B1MH07	*MAB_0593c*	Probable acyl-CoA dehydrogenase FadE	0.066	0.042	11	15
639	B1MHI5	*hemC*	Porphobilinogen deaminase	0.064	0.042	14	22
640	B1MFB6	*MAB_3648*	DUF1338 domain-containing protein	0.049	0.042	14	28
641	B1MNS6	*MAB_1894c*	DUF2235 domain-containing protein	0.018	0.042	4	24
642	B1MI43	*MAB_0769c*	Hypothetical transcriptional regulator	0.109	0.041	6	7
643	B1MGT8	*MAB_0523*	Hypoxanthine phosphoribosyltransferase	0.095	0.041	10	12
644	B1MFM6	*MAB_0327*	Possible aminoglycoside phosphotransferase	0.05	0.041	8	21
645	B1MLV8	*atpF*	ATP synthase subunit b	0.02	0.041	12	12
646	B1MKW5	*MAB_4541*	Putative transcriptional regulator, TetR family	0.061	0.04	8	12
647	B1MKG0	*MAB_1164*	Putative conserved lipoprotein LpqU	0.029	0.04	7	16
648	B1MF81	*MAB_3612c*	Putative dTDP-rhamnosyltransferase	0.045	0.039	11	18
649	B1MDZ4	*MAB_3387*	Putative GntR-family regulatory protein	0.053	0.037	14	31
650	B1MFJ8	*MAB_0299*	Putative amidohydrolase (Aminocarboxymuconate-semialdehyde decarboxylase)	0.048	0.037	11	17
651	B1MLZ9	*MAB_1490*	Uncharacterized protein	0.097	0.036	16	26
652	B1MNY7	*MAB_1956*	Probable glycerate kinase	0.062	0.036	11	15
653	B1MI80	*MAB_0807*	Uncharacterized protein	0.06	0.036	3	21
654	B1MAQ8	*MAB_2248*	Mycobactin synthetase protein B	0.035	0.036	27	84
655	B1MDN6	*MAB_3279c*	Putative phosphatase/kinase	0.128	0.035	23	28
656	B1MIS4	*MAB_4222*	Uncharacterized protein	0.042	0.035	15	25
657	B1MGF7	*MAB_3827c*	Ethanolamine ammonia-lyase, small subunit	0.042	0.035	6	15
658	B1MK45	*MAB_4483*	Uncharacterized protein	0.04	0.035	6	20

Table S4. Positively enriched proteins captured by VM055*p* probe from *M. abscessus* S culture through CC-ABPP by LC-ESI-MS/MS analysis, compared to DMSO-treated cells (*i.e*., non-specific conditions, NS)

	Protein IDs	Gene names	Protein names	VM055*p* *vs*. NS -LOG(p-value)	VM055*p* *vs*. NS Fold Change (Log2)	peptides counts all	nb Tryptic Peptides
659	B1MEU7	*MAB_3477c*	MscS Mechanosensitive ion channel	0.03	0.035	15	20
660	B1MF86	*MAB_3618c*	Probable acyl-CoA dehydrogenase FadE	0.049	0.034	39	26
661	B1MCX5	*MAB_3018*	Putative transcriptional regulator, GntR family	0.043	0.034	5	18
662	B1MD45	*MAB_3089c*	Thioredoxin-like_fold domain-containing protein	0.026	0.034	4	10
663	B1MGQ6	*MAB_0491*	Probable DNA polymerase III, delta' subunit	0.073	0.033	26	31
664	B1MP15	*MAB_1984*	Probable 1-acylglycerol-3-phosphate O-acyltransferase	0.056	0.033	19	18
665	B1MIG6	*MAB_4114*	Uncharacterized protein	0.052	0.033	8	4
666	B1MCJ3	*MAB_2885*	Putative transcriptional regulator, TetR-family	0.048	0.033	5	11
667	B1MGT3	*ppa*	Inorganic pyrophosphatase	0.024	0.033	16	9
668	B1MDP3	*ddl*	D-alanine--D-alanine ligase	0.179	0.032	25	26
669	B1ME24	*MAB_3417c*	Probable NADPH-dependent FMN reductase	0.023	0.032	6	10
670	B1ME69	*MAB_0031*	Uncharacterized protein	0.023	0.032	5	15
671	B1MMX5	*MAB_1602*	Uncharacterized protein	0.023	0.032	7	6
672	B1MF68	*MAB_3599c*	Putative amino acid permease	0.009	0.032	3	19
673	B1MC56	*MAB_2748c*	Uncharacterized protein	0.091	0.031	22	26
674	B1MBA2	*MAB_2442c*	Uncharacterized protein	0.058	0.031	3	8
675	B1MBX3	*hisF*	Imidazole glycerol phosphate synthase subunit HisF	0.047	0.031	18	16
676	B1MIU8	*MAB_4246*	Putative Na+/H+ antiporter	0.042	0.03	16	26
677	B1MIK8	*MAB_4156c*	Putative short chain dehydrogenase/reductase	0.032	0.03	12	18
678	B1MHH6	*MAB_3983c*	Uncharacterized protein	0.113	0.029	18	15
679	B1MMZ1	*proA*	Gamma-glutamyl phosphate reductase	0.09	0.029	16	23
680	B1MHH0	*MAB_3977c*	Uncharacterized protein	0.078	0.029	7	17
681	B1MLL3	*glgC*	Glucose-1-phosphate adenylyltransferase	0.061	0.029	16	25
682	B1MEI3	*MAB_0147c*	Uncharacterized protein	0.054	0.029	27	20
683	B1MHW1	*MAB_0686*	Putative transcription regulator, TetR family	0.057	0.028	7	12
684	B1MCN1	*MAB_2924c*	Uncharacterized protein	0.045	0.028	5	22
685	B1MAV8	*coaE*	Dephospho-CoA kinase	0.037	0.028	26	24
686	B1MH85	*rplA*	50S ribosomal protein L1	0.047	0.027	23	20
687	B1MJF8	*MAB_1024*	Putative TetR-family transcriptional regulator	0.032	0.027	2	11
688	B1MIA1	*MAB_0829*	Putative oxidoreductase	0.019	0.026	2	10
689	B1MHR9	*MAB_4077*	Uncharacterized protein	0.016	0.026	9	12
690	B1MG40	*MAB_3711*	Putative nucleoside hydrolase IunH	0.051	0.025	9	15
691	B1MK66	*MAB_4504c*	Uncharacterized protein	0.033	0.025	13	32
692	B1MGJ2	*MAB_3863*	Uncharacterized protein	0.02	0.025	8	8
693	B1MFU8	*recB*	RecBCD enzyme subunit RecB	0.056	0.024	31	65
694	B1MKH5	*MAB_1179c*	Uncharacterized protein	0.04	0.024	5	9
695	B1MBD1	*MAB_2471c*	Ferric uptake regulation protein FurA	0.024	0.024	5	9
696	B1MEN3	*MAB_0197*	Putative alkylhydroperoxidase AhpD core	0.021	0.024	5	11
697	B1MEQ5	*MAB_3435*	Putative acyl-CoA ligase	0.106	0.023	11	21
698	B1MBX5	*priA*	Phosphoribosyl isomerase A	0.038	0.023	12	16
699	B1MJB7	*MAB_0981c*	Possible enoyl-CoA hydratase/isomerase	0.026	0.023	14	14
700	B1MAK8	*MAB_2198c*	Probable cobalamin biosynthesis protein CobI	0.024	0.023	14	32
701	B1MIQ9	*MAB_4207*	4-aminobutyrate aminotransferase (GabT)	0.023	0.023	22	19
702	B1MBK5	*MAB_2545c*	Hypothetical nitrilase/cyanide hydratase and apolipoprotein N-acyltransferase	0.028	0.022	12	17
703	B1MH34	*MAB_0620c*	Uncharacterized protein	0.027	0.022	4	8
704	B1MH42	*MAB_0628*	AAA_31 domain-containing protein	0.023	0.022	28	37
705	B1MF02	*MAB_3532*	Uncharacterized protein	0.011	0.022	14	27

Table S4. Positively enriched proteins captured by VM055*p* probe from *M. abscessus* S culture through CC-ABPP by LC-ESI-MS/MS analysis, compared to DMSO-treated cells (*i.e*., non-specific conditions, NS)

	Protein IDs	Gene names	Protein names	VM055*p* *vs*. NS -LOG(p-value)	VM055*p* *vs*. NS Fold Change (Log2)	peptides counts all	nb Tryptic Peptides
706	B1MGC3	*rplO*	50S ribosomal protein L15	0.028	0.021	14	10
707	B1MDV5	*MAB_3348*	Uncharacterized protein	0.01	0.021	4	11
708	B1MM70	*MAB_4787c*	Hypothetical regulatory protein, TetR family	0.027	0.02	9	10
709	B1MHE5	*MAB_3952*	Possible O-succinylbenzoic acid--CoA ligase MenE	0.027	0.02	13	18
710	B1MFX8	*MAB_0429*	Putative oligopeptide ABC transporter,ATP-binding protein	0.024	0.02	22	33
711	B1MB38	*MAB_2378c*	Uncharacterized protein	0.022	0.02	20	21
712	B1MLK2	*MAB_1341*	Cytokinin riboside 5'-monophosphate phosphoribohydrolase	0.051	0.019	14	12
713	B1MG04	*MAB_3675*	Succinate dehydrogenase flavoprotein subunit	0.024	0.019	55	41
714	B1MCR9	*MAB_2962*	Probable fatty-acid-CoA ligase FadD	0.018	0.019	26	56
715	B1MBD4	*MAB_2474*	NLPC_P60 domain-containing protein	0.011	0.019	4	7
716	B1MKI3	*MAB_1187c*	Probable enoyl-CoA hydratase	0.069	0.018	20	22
717	B1MGG7	*MAB_3838c*	Putative ferredoxin reductase	0.067	0.018	22	23
718	B1MGE0	*MAB_3810*	Putative hydrolase, alpha/beta fold	0.051	0.018	21	16
719	B1MC08	*MAB_2699c*	Pseudouridine synthase	0.048	0.018	15	22
720	B1MCK6	*thrS*	Threonine--tRNA ligase	0.032	0.018	61	47
721	B1MF62	*MAB_3593*	Possible Mg2+ transport P-type ATPase C MgtC	0.027	0.018	10	14
722	B1MBH3	*MAB_2513c*	Anti-sigma factor RsbW	0.037	0.017	13	9
723	B1MDS9	*MAB_3322c*	Acetohydroxy-acid synthase small subunit	0.028	0.017	9	14
724	B1MLR6	*MAB_1407c*	Probable oxidoreductase	0.028	0.017	21	22
725	B1ML56	*MAB_4632*	Putative short chain dehydrogenase/reductase	0.025	0.017	11	16
726	B1ME12	*MAB_3405*	Putative short chain dehydrogenase/reductase	0.024	0.017	10	15
727	B1MBF3	*MAB_2493c*	Similar to streptomycin adenyltransferase	0.017	0.017	8	18
728	B1MF12	*MAB_3542c*	zf-HC2 domain-containing protein	0.032	0.016	2	7
729	B1MLY7	*MAB_1478*	MPN domain-containing protein	0.02	0.016	15	8
730	B1MN84	*MAB_1711c*	DUF5642 domain-containing protein	0.017	0.016	14	16
731	B1MDU8	*gatA*	Glutamyl-tRNA(Gln) amidotransferase subunit A	0.061	0.015	32	25
732	B1MDX4	*MAB_3367*	Putative fatty-acid-CoA ligase	0.025	0.015	49	67
733	B1MFQ3	*MAB_0354c*	Oxidoreductase, 2-nitropropane dioxygenase family	0.024	0.015	7	14
734	B1MML2	*MAB_4929*	Uncharacterized protein	0.02	0.015	2	17
735	B1MEK0	*MAB_0164*	Probable short chain dehydrogenase/reductase	0.047	0.014	11	9
736	B1MPE7	*MAB_2117*	Hypothetical short-chain dehydrogenase/reductase	0.027	0.014	12	22
737	B1MCR5	*MAB_2958*	Putative transmembrane-transport protein	0.027	0.014	3	14
738	B1MEW2	*MAB_3492c*	Uncharacterized protein	0.013	0.014	11	19
739	B1MKL4	*MAB_1218*	Probable aldehyde dehydrogenase AldA	0.022	0.013	6	25
740	B1MML7	*MAB_4934c*	Poly(A) polymerase PcnA	0.017	0.013	28	26
741	B1MML9	*MAB_4936*	Uncharacterized protein	0.014	0.013	19	37
742	B1MNE2	*MAB_1758*	Uncharacterized protein	0.012	0.013	1	3
743	B1MMP0	*MAB_1516*	Uncharacterized protein	0.01	0.013	10	7
744	B1MM06	*tetR*	DNA-binding transcriptional repressor TetR	0.009	0.013	4	14
745	B1MH92	*rpmG2*	50S ribosomal protein L33 2	0.009	0.013	14	5
746	B1MNZ5	*MAB_1964*	Uncharacterized protein	0.037	0.012	8	11
747	B1MCN4	*MAB_2927c*	Putative hydrolase	0.024	0.012	12	26
748	B1MHI8	*MAB_3995*	Uncharacterized protein	0.023	0.012	3	16
749	B1MEN2	*MAB_0196c*	Putative transcriptional regulator, GntR family	0.013	0.012	9	28
750	B1MDC1	*MAB_3165c*	Uncharacterized protein	0.01	0.012	17	12
751	B1MKM5	*MAB_1229*	Uncharacterized protein	0.03	0.011	11	24
752	B1MNW4	*MAB_1932c*	Probable chaperone protein HchA (Hsp31)	0.013	0.011	22	16

Table S4. Positively enriched proteins captured by VM055*p* probe from *M. abscessus* S culture through CC-ABPP by LC-ESI-MS/MS analysis, compared to DMSO-treated cells (*i.e* ., non-specific conditions, NS)

	Protein IDs	Gene names	Protein names	VM055*p* *vs* . NS -LOG(p-value)	VM055*p* *vs* . NS Fold Change (Log2)	peptides counts all	nb Tryptic Peptides
753	B1MH54	*MAB_0640c*	Molybdopterin molybdenumtransferase	0.032	0.01	15	14
754	B1ME71	*MAB_0033c*	Serine/threonine-protein kinase PknB	0.057	0.009	43	41
755	B1MD05	*miaB*	tRNA-2-methylthio-N(6)-dimethylallyladenosine synthase	0.024	0.009	15	25
756	B1MBZ6	*MAB_2687c*	8-amino-7-oxononanoate synthase (BioF)	0.015	0.009	13	20
757	B1MHT0	*MAB_0654*	DUF58 domain-containing protein	0.013	0.009	10	24
758	B1MBI3	*MAB_2523c*	Putative transcription antitermination regulator	0.012	0.009	6	15
759	B1MDH4	*MAB_3219*	Uncharacterized protein	0.007	0.009	8	7
760	B1MKP9	*MAB_1253c*	D-ser_dehydrat domain-containing protein	0.021	0.008	8	16
761	B1MM68	*MAB_4785*	Uncharacterized protein	0.01	0.008	9	15
762	B1MLY4	*clpS*	ATP-dependent Clp protease adapter protein ClpS	0.016	0.007	4	5
763	B1MM01	*MAB_1492*	Uncharacterized protein	0.01	0.007	63	74
764	B1MET9	*MAB_3469*	MutT/NUDIX family protein	0.012	0.006	4	8
765	B1MI97	*MAB_0825*	Hypothetical peptidase	0.012	0.006	8	27
766	B1MAF4	*MAB_p21c*	Uncharacterized protein	0.011	0.006	10	20
767	B1MH38	*MAB_0624*	Probable hydratase/decarboxylase	0.011	0.006	11	17
768	B1MIJ2	*MAB_4140*	Uncharacterized protein	0.009	0.006	18	12
769	B1MGR6	*MAB_0501c*	Probable peptidase S15	0.008	0.006	17	33
770	B1MAI2	*prcB*	Proteasome subunit beta	0.006	0.006	13	20
771	B1MIX9	*MAB_4277*	Luciferase-like monooxygenase superfamily	0.005	0.006	18	17
772	B1MBY2	*MAB_2673c*	L-aspartate oxidase	0.013	0.005	19	30
773	B1MEK7	*MAB_0171*	Glft2_N domain-containing protein	0.012	0.005	52	40
774	B1MGP9	*MAB_0484*	Putative hydrolase/esterase/lipase	0.009	0.005	13	15
775	B1MCT2	*MAB_2975c*	AFG1-like ATPase	0.009	0.005	16	20
776	B1MNX2	*MAB_1940c*	Uncharacterized protein	0.005	0.005	13	15
777	B1MJM1	*MAB_4306*	Putative transglutaminase-like protein	0.005	0.005	6	12
778	B1MI93	*MAB_0821*	Probable 4-aminobutyrate aminotransferase (GabT)	0.013	0.004	31	22
779	B1MKW1	*MAB_4537c*	Uncharacterized protein	0.004	0.004	21	15
780	B1MME8	*MAB_4865*	Putative pyridine nucleotide-disulphide oxidoreductase	0.005	0.003	18	20
781	B1MJN9	*MAB_4324c*	Putative acetyltransferase, GNAT	0.003	0.003	14	21
782	B1MGD3	*MAB_3803c*	Carboxylic ester hydrolase	0.002	0.003	13	28
783	B1MI19	*mshD*	Mycothiol acetyltransferase	0.001	0.003	10	20
784	B1MLW5	*MAB_1456c*	Corrinoid adenosyltransferase	0.004	0.002	6	12
785	B1MMZ5	*rsfS*	Ribosomal silencing factor RsfS	0.004	0.002	6	7
786	B1MNR3	*MAB_1881c*	Putative transcriptional regulator, TetR family	0.002	0.002	12	13
787	B1MNN3	*MAB_1851*	Probable acyl-CoA dehydrogenase FadE	0.004	0.001	25	28
788	B1MIX3	*dnaJ*	Chaperone protein DnaJ	0.004	0.001	43	27
789	B1MD54	*MAB_3098*	Probable transmembrane carbonic anhydrase	0.002	0.001	15	26
790	B1MI24	*MAB_0750*	Putative oxidoreductase	0.001	0.001	3	14
791	B1MCJ1	*ruvA*	Holliday junction ATP-dependent DNA helicase RuvA	0.001	0.001	7	15
792	B1MH09	*MAB_0595c*	Probable acyl-CoA dehydrogenase FadE	0.001	0.001	6	30
793	B1MFP6	*MAB_0347*	Cell division control protein 48 CDC48	0.001	0.001	12	18
794	B1MAU7	*MAB_2287*	Putative metal dependent phosphohydrolase	0	0.001	6	17
795	B1MBW6	*MAB_2657c*	Uncharacterized protein	0	0	16	29
796	B1MN91	*MAB_4949c*	Probable chromosome partitioning protein ParB	0	0	16	22
797	B1MK43	*MAB_4481*	Uncharacterized methyltransferase MAB_4481	0	0	10	18

Table S5. **Positively enriched proteins captured by VM055*p* probe from pre-incubated VM043-*M. abscessus* S culture through CC-ABPP by LC-ESI-MS/MS analysis, compared to DMSO-treated cells (i.e., non-specific conditions, NS)**

				[VM043+VM055*p*] *vs.* NS			
	**Protein IDs**	**Gene names**	**Protein names**	**-LOG(p-value)**	**Fold Change (Log2)**	**peptides counts all**	**nb Tryptic Peptides**
1	B1MF33	*MAB_3564c*	Hypothetical dipeptidyl aminopeptidase/ acylaminoacyl-peptidase related protein	5.765	3.488	10	27
2	B1MKX5	*MAB_4551c*	Possible lysophospholipase/acylglycerol lipase	4.202	2.260	5	19
3	B1MJG0	*MAB_1026c*	Uncharacterized protein	3.481	1.935	3	9
4	B1MDI7	*MAB_3230c*	SnoaL-like domain-containing protein	1.347	1.874	3	6
5	B1MCT9	*MAB_2982c*	Uncharacterized protein	1.471	1.863	2	27
6	B1MG83	*MAB_3754c*	ESAT-6-like protein	2.344	1.841	14	7
7	B1MNS7	*MAB_1895c*	Uncharacterized protein	3.053	1.797	3	18
8	B1MEX1	*MAB_3501*	Uncharacterized protein	1.875	1.757	11	20
9	B1MEW6	*MAB_3496*	Uncharacterized protein	3.179	1.608	2	6
10	B1MMX9	*MAB_1606*	Nucleoside diphosphate kinase	1.975	1.551	2	10
11	B1MEQ6	*MAB_3436*	non-specific serine/threonine protein kinase	1.326	1.423	1	5
12	B1MBL9	*MAB_2559c*	Peptidyl-prolyl cis-trans isomerase	2.369	1.418	6	7
13	B1MMK7	*MAB_4924*	Uncharacterized protein	4.498	1.319	4	10
14	B1MP79	*MAB_2048c*	Probable cytochrome P450	1.363	1.270	29	32
15	B1MIY6	*MAB_4284c*	Uncharacterized protein	1.661	1.227	4	8
16	B1MHJ8	*MAB_4005c*	Pyrroline-5-carboxylate reductase	1.403	1.204	10	15
17	B1MAV6	*MAB_2296*	30S ribosomal protein S1	1.535	1.106	31	37
18	B1MPA8	*MAB_2078*	Probable cytochrome P450	1.615	1.088	13	39
19	B1MCH5	*MAB_2867*	Uncharacterized protein	1.952	1.085	11	13
20	B1MF35	*MAB_3566c*	Putative cyclase	2.574	1.064	4	15
21	B1MIW2	*MAB_4260c*	Orotate phosphoribosyltransferase	1.480	1.004	4	13
22	B1MBP3	*MAB_2583c*	Putative transcription regulator, AraC family	1.072	1.804	4	10
23	B1MM39	*MAB_4756c*	Monooxygenase, FAD-binding	0.997	1.324	14	30
24	B1MDK8	*MAB_3251c*	Histidine kinase	0.757	1.253	11	46
25	B1MFL8	*MAB_0319*	Nucleoid-associated protein MAB_0319	0.935	1.231	2	4
26	B1MNQ3	*MAB_1871*	Uncharacterized protein	0.82	1.225	5	9
27	B1MJ97	*MAB_0961c*	Methyltransf_25 domain-containing protein	0.961	1.173	5	10
28	B1MEQ0	*MAB_0214c*	Uncharacterized protein	1.149	1.17	3	8
29	B1MFY2	*MAB_3653*	Probable pyridoxine 5-phosphate oxidase	1.132	1.132	4	15
30	B1MBV2	*trpA*	Tryptophan synthase alpha chain	1.051	1.132	9	15
31	B1MKT6	*tpx*	Thiol peroxidase	1.198	1.12	9	9
32	B1MP40	*ftsZ*	Cell division protein FtsZ	0.953	1.113	33	19
33	B1MF07	*MAB_3537c*	Uncharacterized protein	1.201	1.106	2	2
34	B1MEI4	*MAB_0148c*	PPE family protein	1.28	1.052	8	14
35	B1MDW9	*MAB_3362c*	Electron transfer flavoprotein alpha-subunit FixB	0.931	1.032	8	16
36	B1MMI2	*rpsF*	30S ribosomal protein S6	0.789	1.027	4	7
37	B1MFB2	*MAB_3644*	Uncharacterized protein	1.123	1.026	7	7
38	B1MAS5	*ectC*	L-ectoine synthase	1.118	1.021	4	5
39	B1MF26	*MAB_3556*	Putative hydrolase, alpha/beta fold	0.686	1.013	8	18
40	B1MCH9	*MAB_2871c*	Uncharacterized protein	1.251	1.007	1	7

Table S5. Positively enriched proteins captured by VM055*p* probe from pre-incubated VM043-*M. abscessus* S culture through CC-ABPP by LC-ESI-MS/MS analysis, compared to DMSO-treated cells (i.e., non-specific conditions, NS)

	Protein IDs	Gene names	Protein names	[VM043+VM055*p*] *vs.* NS		peptides counts all	nb Tryptic Peptides
				-LOG(p-value)	Fold Change (Log2)		
41	B1MMF1	*MAB_4868c*	Uncharacterized protein	1.397	0.997	10	15
42	B1MF87	*purE*	N5-carboxyaminoimidazole ribonucleotide mutase	0.756	0.959	3	7
43	B1MI56	*MAB_0782*	Uncharacterized protein	1.066	0.955	12	9
44	B1MNR0	*acpP*	Acyl carrier protein	1.45	0.949	9	7
45	B1MLL1	*MAB_1350*	Uncharacterized protein	2.067	0.947	2	5
46	B1MB33	*MAB_2373*	Putative mannose-specific lectin	1.575	0.946	3	6
47	B1MCB6	*MAB_2808c*	Riboflavin biosynthesis protein RibD	1.486	0.917	11	23
48	B1MP35	*murD*	UDP-N-acetylmuramoylalanine--D-glutamate ligase	1.093	0.914	20	28
49	B1MLA5	*MAB_4682c*	Uncharacterized protein	1.269	0.91	2	5
50	B1MDV5	*MAB_3348*	Uncharacterized protein	0.973	0.907	4	11
51	B1MHD8	*menB*	1,4-dihydroxy-2-naphthoyl-CoA synthase	0.703	0.898	18	19
52	B1MD74	*MAB_3118c*	Metallophos domain-containing protein	1.038	0.898	7	18
53	B1ML47	*metE*	5-methyltetrahydropteroyltriglutamate--homocysteine methyltransferase	1.301	0.895	19	42
54	B1MES5	*MAB_3455c*	Putative acyl-CoA thiolase	0.979	0.886	8	24
55	B1MDG5	*MAB_3210c*	Uncharacterized protein	1.39	0.884	4	14
56	B1MGS4	*MAB_0509c*	Aminopeptidase N	1.043	0.884	16	23
57	B1MJB0	*MAB_0974*	Uncharacterized protein	1.132	0.879	13	29
58	B1MLK3	*MAB_1342*	Probable fatty-acid-CoA ligase FadD	1.448	0.874	51	36
59	B1MMQ6	*orn*	Oligoribonuclease	1.256	0.865	7	13
60	B1MCD2	*MAB_2824c*	Putative integration host factor (MihF)	0.642	0.852	11	9
61	B1MHE4	*MAB_3951*	Uncharacterized protein	1.248	0.846	2	10
62	B1MDY4	*MAB_3377*	Uncharacterized protein	1.651	0.845	3	12
63	B1MAK3	*MAB_2193c*	Putative_PNPOx domain-containing protein	1.129	0.844	4	12
64	B1MNR9	*MAB_1887*	Probable peroxiredoxin (Thioredoxin reductase) AhpE	0.71	0.828	10	9
65	B1MAV3	*MAB_2293*	Putative transcriptional regulator, MarR family	1.507	0.828	6	11
66	B1MDE3	*frr*	Ribosome-recycling factor	1.251	0.826	9	14
67	B1MJC0	*MAB_0984c*	Putative transcriptional regulator, TetR family	2.147	0.825	13	14
68	B1MK18	*MAB_4456*	Putative cytochrome P450	1.088	0.822	16	28
69	B1MLT6	*MAB_1427c*	Putative cytochrome P450	2.157	0.816	18	31
70	B1MKN7	*MAB_1241c*	CsbD domain-containing protein	1.337	0.814	6	6
71	B1MBD4	*MAB_2474*	NLPC_P60 domain-containing protein	1.109	0.812	4	7
72	B1MGQ2	*MAB_0487*	Probable cold shock protein A (CspA)	0.669	0.804	3	6
73	B1MKS6	*MAB_1280c*	Uncharacterized protein	1.806	0.782	2	17
74	B1MFK1	*aqdB*	2-heptyl-3-hydroxy-4(1H)-quinolone synthase	1.232	0.78	3	18
75	B1MJ23	*MAB_0885c*	Hypothetical lipoprotein lpqH	1.571	0.78	5	5
76	B1MDV1	*MAB_3344*	Probable glycosyl transferase	1.351	0.778	3	29
77	B1MM04	*MAB_1495*	Probable oxidoreductase	1.073	0.771	37	42
78	B1MK17	*MAB_4455c*	Probable acyl-CoA synthetase FadD	1.924	0.766	4	32
79	B1MBT6	*MAB_2627c*	Possible two-component response regulatory protein	1.269	0.763	11	12
80	B1MP12	*MAB_1981*	Uncharacterized protein	1.101	0.756	6	10

Table S5. Positively enriched proteins captured by VM055*p* probe from pre-incubated VM043-*M. abscessus* S culture through CC-ABPP by LC-ESI-MS/MS analysis, compared to DMSO-treated cells (i.e., non-specific conditions, NS)

				[VM043+VM055*p*] *vs.* NS			
	Protein IDs	Gene names	Protein names	-LOG(p-value)	Fold Change (Log2)	peptides counts all	nb Tryptic Peptides
81	B1MNV7	*MAB_1925*	MHB domain-containing protein	3.877	0.753	8	8
82	B1MK00	*MAB_4437*	Probable acyl-CoA dehydrogenase FadE	1.255	0.752	58	39
83	B1MIW7	*clpB*	Chaperone protein ClpB	0.952	0.749	77	60
84	B1MIX4	*grpE*	Protein GrpE	1.256	0.745	17	13
85	B1MKD7	*fabH*	3-oxoacyl-[acyl-carrier-protein] synthase 3	0.785	0.739	18	16
86	B1MCT6	*MAB_2979*	Peptide-methionine (R)-S-oxide reductase	0.808	0.737	12	9
87	B1MAW1	*MAB_2301*	Putative membrane protein, mmpL	0.978	0.735	22	55
88	B1ML28	*MAB_4604c*	Putative short-chain dehydrogenase/reductase	0.52	0.726	13	17
89	B1MGF4	*MAB_3824*	Uncharacterized protein	1.786	0.721	10	10
90	B1MFF0	*MAB_0250*	Hypothetical GMC-type oxidoreductase	1.256	0.72	39	32
91	B1MKH0	*MAB_1174c*	Probable acyl-CoA synthase FadD	0.904	0.716	15	32
92	B1MF72	*MAB_3603c*	Probable phosphomannomutase	0.985	0.715	19	25
93	B1MFR1	*MAB_0362c*	N-acetylglucosamine-6-phosphate deacetylase NagA	1.27	0.713	8	13
94	B1MHA5	*MAB_3912*	UPF0234 protein MAB_3912	0.989	0.704	9	14
95	B1ML11	*MAB_4587c*	Putative S-adenosyl-L-methionine-dependent methyltransferase MAB_4587c	1.283	0.699	10	21
96	B1ML07	*MAB_4583c*	Uncharacterized protein	1.504	0.699	9	9
97	B1MLD7	*MAB_4714c*	Probable fatty-acid-coa ligase FadD	1.242	0.692	66	74
98	B1MKF4	*MAB_1158*	Putative transcriptional regulator, MazG family	0.842	0.689	15	18
99	B1MKV0	*MAB_4526*	Uncharacterized protein	0.489	0.687	1	6
100	B1MGU0	*MAB_0525c*	Probable conserved lipoprotein LpqG	2.109	0.684	5	15
101	B1MCJ4	*MAB_2886c*	Uncharacterized protein	0.581	0.682	3	3
102	B1MPA9	*MAB_2079*	TGc domain-containing protein	2.507	0.679	4	18
103	B1MID5	*MAB_4083c*	Heparin-binding hemagglutinin (Adhesin)	1.898	0.674	14	15
104	B1MPB1	*MAB_2081*	Probable acyl CoA dehydrogenase	1.439	0.666	16	23
105	B1MB16	*MAB_2356*	TPR_5 domain-containing protein	1.83	0.656	12	26
106	B1MJ72	*MAB_0936c*	PE-PPE domain-containing protein	0.936	0.644	9	16
107	B1MP52	*MAB_2021c*	Putative carboxymuconolactone decarboxylase	1.112	0.642	2	13
108	B1MMM4	*MAB_4941*	Thioredoxin	1.292	0.641	9	7
109	B1ML36	*MAB_4612*	NTP_transf_9 domain-containing protein	0.696	0.639	4	8
110	B1MMV3	*tig*	Trigger factor	1.091	0.638	21	33
111	B1MJX0	*ahpD*	Alkyl hydroperoxide reductase AhpD	0.949	0.638	10	12
112	B1MN94	*MAB_4952c*	R3H domain-containing protein	0.817	0.636	4	12
113	B1MH69	*rplL*	50S ribosomal protein L7/L12	1.41	0.635	11	7
114	B1MCA6	*MAB_2798c*	Uncharacterized protein	1.507	0.63	3	6
115	B1MMM3	*MAB_4940*	Thioredoxin reductase	0.96	0.628	29	22
116	B1MGA7	*MAB_3777*	Band 7 protein	0.843	0.624	15	36
117	B1MD63	*MAB_3107c*	Possible lipoprotein LppU	1.109	0.623	4	8
118	B1MHW2	*MAB_0687*	Adenylosuccinate lyase	0.946	0.617	42	32
119	B1MDX0	*MAB_3363c*	Electron transfer flavoprotein beta-subunit FixA	0.87	0.612	20	20
120	B1MIA7	*MAB_0835c*	Putative transcriptional regulator, TetR family	0.885	0.608	6	14

Table S5. **Positively enriched proteins captured by VM055*p* probe from pre-incubated VM043-*M. abscessus* S culture through CC-ABPP by LC-ESI-MS/MS analysis, compared to DMSO-treated cells (i.e., non-specific conditions, NS)**

	Protein IDs	Gene names	Protein names	[VM043+VM055*p*] *vs.* NS		peptides counts all	nb Tryptic Peptides
				-LOG(p-value)	Fold Change (Log2)		
121	B1MLH9	*MAB_1318c*	Probable catechol-o-methyltransferase	1.569	0.605	7	19
122	B1MFQ0	*MAB_0351*	Catalase	0.999	0.603	8	31
123	B1MJ79	*MAB_0943*	DJ-1_PfpI domain-containing protein	1.132	0.6	7	12
124	B1MLK7	*MAB_1346*	Glyco_trans_2-like domain-containing protein	1.143	0.599	7	11
125	B1MJB9	*MAB_0983c*	Probable alcohol dehydrogenase, zinc-containing	0.801	0.588	21	16
126	B1MFR6	*MAB_0367c*	SnoaL-like domain-containing protein	1.726	0.587	6	10
127	B1MBP5	*MAB_2585*	L-ectoine synthase	0.8	0.583	4	6
128	B1MEP0	*MAB_0204c*	Bacterial proteasome activator	1.14	0.577	7	12
129	B1MLR6	*MAB_1407c*	Probable oxidoreductase	0.908	0.574	21	22
130	B1MJK7	*MAB_1073c*	Probable acyl-CoA dehydrogenase FadE	1.27	0.573	13	21
131	B1MF68	*MAB_3599c*	Putative amino acid permease	0.554	0.572	3	19
132	B1MNB1	*MAB_1727c*	Bacteriophage protein	0.662	0.57	6	10
133	B1MDH2	*MAB_3217*	CsbD domain-containing protein	1.332	0.563	7	11
134	B1MNW3	*MAB_1931c*	Conserved hypothetical integral membrane protein	0.789	0.562	1	2
135	B1MBQ5	*MAB_2595*	Putative pyridoxamine 5'-phosphate oxidase	1.382	0.561	5	12
136	B1MBJ7	*MAB_2537c*	Putative pyruvate decarboxylase	1.064	0.56	4	38
137	B1MG60	*groL*	60 kDa chaperonin	1.102	0.558	35	36
138	B1MLU1	*MAB_1432*	Uncharacterized protein	0.804	0.554	5	10
139	B1MHP0	*MAB_4047c*	Sensory transduction protein RegX3	0.54	0.553	15	16
140	B1MLJ7	*MAB_1336*	Probable succinyl-diaminopimelate desuccinylase	0.831	0.552	19	21
141	B1MK98	*MAB_1101*	Uncharacterized protein	0.899	0.551	33	46
142	B1MP80	*MAB_2049c*	Probable ferredoxin	0.706	0.551	2	2
143	B1MKU4	*MAB_4520c*	Putative two-component system sensor kinase	0.614	0.549	1	22
144	B1MIL1	*MAB_4159*	Probable acyl-CoA dehydrogenase	0.618	0.549	20	21
145	B1MDI2	*MAB_3225*	Putative lipoprotein LppW	0.94	0.541	16	16
146	B1MJB2	*MAB_0976*	Putative glyoxalase/bleomycin resistance protein	0.559	0.54	3	9
147	B1MDR0	*leuB*	3-isopropylmalate dehydrogenase	0.794	0.538	8	19
148	B1MHK9	*MAB_4016c*	Uncharacterized protein	1.051	0.534	1	12
149	B1MAD5	*MAB_p02*	Uncharacterized protein	0.653	0.532	3	12
150	B1ML70	*MAB_4646*	DUF2236 domain-containing protein	0.832	0.53	12	26
151	B1MDJ1	*MAB_3234*	Probable D-alanyl-D-alanine carboxypeptidase DacB	1.676	0.53	14	16
152	B1ML81	*MAB_4657c*	Putative short-chain dehydrogenase/reductase	1.057	0.529	14	17
153	B1MLP8	*MAB_1389c*	Probable short-chain dehydrogenase/reductase	0.612	0.524	7	23
154	B1MLY5	*MAB_1476*	DUF2017 domain-containing protein	0.645	0.522	5	9
155	B1MD17	*recA*	Protein RecA	1.618	0.522	26	23
156	B1MKD9	*rplY*	50S ribosomal protein L25	0.971	0.521	5	7
157	B1MM65	*MAB_4782*	Uncharacterized protein	0.657	0.519	12	16
158	B1MEV7	*MAB_3487*	Probable acyl-CoA dehydrogenase	1.443	0.513	27	25
159	B1ML25	*MAB_4601c*	Putative YrbE family protein	0.582	0.513	10	11
160	B1MHZ6	*MAB_0722*	Putative oligopeptide ABC transporter,ATP-binding protein	0.711	0.509	11	35

Table S5. Positively enriched proteins captured by VM055*p* probe from pre-incubated VM043-*M. abscessus* S culture through CC-ABPP by LC-ESI-MS/MS analysis, compared to DMSO-treated cells (i.e., non-specific conditions, NS)

	Protein IDs	Gene names	Protein names	[VM043+VM055*p*] *vs.* NS -LOG(p-value)	Fold Change (Log2)	peptides counts all	nb Tryptic Peptides
161	B1MEQ7	*MAB_3437c*	Putative transcriptional regulator, TetR family	2.677	0.5	4	15
162	B1MG98	*rplQ*	50S ribosomal protein L17	1.117	0.493	11	14
163	B1MKI1	*MAB_1185c*	Probable enoyl-CoA hydratase	1.812	0.492	14	16
164	B1MIM8	*MAB_4176c*	NAD_binding_9 domain-containing protein	0.867	0.488	2	25
165	B1MMS0	*MAB_1547c*	Probable sulfatase	1.107	0.487	12	25
166	B1MIN9	*def*	Peptide deformylase	0.457	0.487	4	8
167	B1MNK9	*MAB_1827c*	Bacteriophage protein	0.714	0.479	1	5
168	B1MKI6	*MAB_1190*	Uncharacterized protein	0.69	0.473	10	10
169	B1MNL1	*MAB_1829*	Uncharacterized protein	0.716	0.464	9	7
170	B1MGE2	*rpmC*	50S ribosomal protein L29	0.738	0.463	11	7
171	B1MES7	*MAB_3457*	DUF1990 domain-containing protein	0.637	0.461	4	14
172	B1MLZ3	*rph*	Ribonuclease PH	1.106	0.458	21	13
173	B1MI44	*MAB_0770*	tRNA_edit domain-containing protein	0.627	0.457	3	10
174	B1MGT9	*MAB_0524c*	Probable conserved lipoprotein LpqG	0.982	0.454	3	12
175	B1MH40	*MAB_0626*	4-hydroxy-2-oxovalerate aldolase 1	0.658	0.453	12	20
176	B1MKU3	*MAB_4519c*	Putative two-component system response regulator,LuxR family	0.754	0.451	3	17
177	B1MIH9	*MAB_4127c*	Dihydrolipoyl dehydrogenase	0.657	0.448	31	30
178	B1MIR5	*MAB_4213c*	Probable lipoprotein aminopeptidase LpqL	1.236	0.445	30	36
179	B1MEE4	*MAB_0108c*	Uncharacterized protein	1.318	0.445	12	9
180	B1MH89	*MAB_3896c*	UPF0336 protein MAB_3896c	0.721	0.445	16	9
181	B1MAF5	*MAB_2145*	NADH-quinone oxidoreductase, L subunit NuoL	0.614	0.439	4	18
182	B1MJZ3	*MAB_4430c*	Putative oxidoreductase	0.413	0.438	3	5
183	B1MNL4	*MAB_1832*	NLPC_P60 domain-containing protein	1.08	0.438	16	32
184	B1MD78	*MAB_3122*	Putative acyl-CoA dehydrogenase	0.746	0.438	29	27
185	B1MHH6	*MAB_3983c*	Uncharacterized protein	2.439	0.438	18	15
186	B1MDF9	*xerC*	Tyrosine recombinase XerC	0.686	0.437	4	20
187	B1MKG9	*MAB_1173c*	Uncharacterized protein	0.735	0.437	6	15
188	B1MCR9	*MAB_2962*	Probable fatty-acid-CoA ligase FadD	1.44	0.437	26	56
189	B1MLS1	*MAB_1412*	Uncharacterized protein	0.45	0.435	1	1
190	B1MAT6	*MAB_2276c*	Putative regulatory protein	0.826	0.434	2	11
191	B1MFQ3	*MAB_0354c*	Oxidoreductase, 2-nitropropane dioxygenase family	1.343	0.432	7	14
192	B1MKF5	*MAB_1159*	Putative iron permease FTR1	0.713	0.431	28	30
193	B1MMU9	*MAB_1576*	Putative TetR-family transcriptional regulator	0.479	0.431	4	16
194	B1ML99	*MAB_4676*	Putative carboxyl transferase/pyruvate carboxylase	1.062	0.43	17	30
195	B1MNS6	*MAB_1894c*	DUF2235 domain-containing protein	0.642	0.429	4	24
196	B1MLH3	*MAB_1312*	Beta-lactamase-like	0.827	0.429	32	39
197	B1MC42	*MAB_2734c*	Uncharacterized protein	0.619	0.429	12	16
198	B1MP38	*murC*	UDP-N-acetylmuramate--L-alanine ligase	0.813	0.425	16	20
199	B1MBX7	*hisB*	Imidazoleglycerol-phosphate dehydratase	0.604	0.42	4	7
200	B1MJV8	*MAB_4394*	AHS2 domain-containing protein	0.837	0.416	6	16

Table S5. **Positively enriched proteins captured by VM055*p* probe from pre-incubated VM043-*M. abscessus* S culture through CC-ABPP by LC-ESI-MS/MS analysis, compared to DMSO-treated cells (i.e., non-specific conditions, NS)**

	Protein IDs	Gene names	Protein names	[VM043+VM055*p*] *vs.* NS		peptides counts all	nb Tryptic Peptides
				-LOG(p-value)	Fold Change (Log2)		
201	B1MN41	*MAB_1668*	PhoH-like protein	0.847	0.41	7	24
202	B1MAY3	*rplT*	50S ribosomal protein L20	0.678	0.41	7	9
203	B1MHQ3	*MAB_4060*	Putative short chain dehydrogenase/reductase	0.933	0.409	10	15
204	B1MJ93	*MAB_0957*	Uncharacterized protein	0.569	0.407	5	6
205	B1MEA5	*MAB_0069*	Major facilitator family transporter	0.95	0.406	6	12
206	B1MJS5	*MAB_4361*	Hypothetical fumarylacetoacetate hydrolase family	0.555	0.406	15	16
207	B1MME0	*MAB_4857*	DUF1942 domain-containing protein	1.002	0.404	8	9
208	B1MME7	*MAB_4864*	Putative arsenate reductase	0.641	0.403	3	10
209	B1MD66	*MAB_3110*	Probable iron dependent transcriptional repressor FeoA	1.128	0.402	15	17
210	B1MAE1	*MAB_p08*	Alkylmercury lyase	0.501	0.402	8	9
211	B1MH86	*rplK*	50S ribosomal protein L11	1.084	0.398	13	13
212	B1MD52	*dapB*	4-hydroxy-tetrahydrodipicolinate reductase	0.749	0.398	10	18
213	B1MK94	*MAB_1097*	Uncharacterized protein	1.114	0.394	11	12
214	B1ME77	*MAB_0039c*	FHA domain-containing protein	0.79	0.392	10	21
215	B1MCF0	*aroK*	Shikimate kinase	0.771	0.392	2	16
216	B1MGG8	*MAB_3839c*	Putative transcriptional regulator, AsnC family	0.992	0.392	6	11
217	B1MDF2	*rpsB*	30S ribosomal protein S2	1.066	0.39	27	17
218	B1MFK0	*MAB_0301*	Carboxylic ester hydrolase	0.765	0.388	5	19
219	B1MD37	*MAB_3080*	Putative antibiotic biosynthesis monooxygenase	0.574	0.38	9	6
220	B1MLG1	*MAB_1300c*	Uncharacterized protein	0.613	0.378	2	20
221	B1MCZ0	*MAB_3033*	Uncharacterized protein	0.665	0.377	6	20
222	B1MF80	*MAB_3611c*	Putative sugar-phosphate nucleotidyl transferase	0.982	0.376	28	26
223	B1MCY5	*MAB_3028*	RNA polymerase sigma factor	0.588	0.376	12	25
224	B1MCU3	*hemE*	Uroporphyrinogen decarboxylase	1.04	0.373	7	17
225	B1MFB9	*MAB_0219*	TED domain-containing protein	0.646	0.372	7	19
226	B1MHY5	*MAB_0711*	SCP_3 domain-containing protein	0.682	0.371	4	9
227	B1MEY2	*MAB_3512*	Putative glutaredoxin-like protein	1.131	0.37	4	5
228	B1MHJ2	*MAB_3999*	MHB domain-containing protein	0.46	0.369	5	3
229	B1MMV5	*clpP*	ATP-dependent Clp protease proteolytic subunit	1.452	0.368	9	14
230	B1MAE6	*MAB_p13*	Uncharacterized protein	0.533	0.368	6	41
231	B1MP55	*MAB_2024*	Amidase family protein	1.273	0.368	22	39
232	B1ML03	*MAB_4579c*	Probable NAD(P) transhydrogenase, alpha1 subunit PntAA	0.866	0.365	27	21
233	B1MAZ9	*argD*	Acetylornithine aminotransferase	0.603	0.364	13	16
234	B1MF86	*MAB_3618c*	Probable acyl-CoA dehydrogenase FadE	1.641	0.364	39	26
235	B1MHI5	*hemC*	Porphobilinogen deaminase	1.087	0.363	14	22
236	B1MLB3	*MAB_4690c*	Mycobactin synthetase protein B	0.539	0.363	32	146
237	B1MHF2	*MAB_3959c*	Putative ATP-dependent Clp protease	0.568	0.361	6	14
238	B1MC53	*MAB_2745c*	Possible SUF system FeS assembly protein	1.05	0.361	4	9
239	B1MGD5	*rplE*	50S ribosomal protein L5	0.974	0.359	19	14
240	B1MP48	*MAB_2017*	Antigen 84	0.894	0.351	29	23

Table S5. Positively enriched proteins captured by VM055*p* probe from pre-incubated VM043-*M. abscessus* S culture through CC-ABPP by LC-ESI-MS/MS analysis, compared to DMSO-treated cells (i.e., non-specific conditions, NS)

	Protein IDs	Gene names	Protein names	[VM043+VM055*p*] *vs.* NS		peptides counts all	nb Tryptic Peptides
				-LOG(p-value)	Fold Change (Log2)		
241	B1MG99	*rpoA*	DNA-directed RNA polymerase subunit alpha	0.538	0.349	33	24
242	B1MCM3	*MAB_2916*	Uncharacterized protein	0.502	0.347	14	32
243	B1MKC5	*MAB_1129*	Probable deoxyribonuclease TatD	0.577	0.346	20	22
244	B1MBZ2	*MAB_2683c*	Uncharacterized protein	0.597	0.346	2	6
245	B1MNV1	*MAB_1919*	AB hydrolase-1 domain-containing protein	0.892	0.345	19	29
246	B1MCB2	*MAB_2804c*	Putative ABC-type transporter, periplasmic component	0.504	0.344	10	19
247	B1MGG9	*MAB_3840*	Uncharacterized protein	0.909	0.338	2	17
248	B1MAX6	*MAB_2316*	Probable acid-CoA ligase	0.479	0.338	10	22
249	B1MCJ1	*ruvA*	Holliday junction ATP-dependent DNA helicase RuvA	0.52	0.338	7	15
250	B1MG16	*MAB_3687*	Probable o-acetylhomoserine sulfhydrylase MetC (Homocysteine synthase)	0.557	0.336	10	20
251	B1MGT3	*ppa*	Inorganic pyrophosphatase	0.666	0.335	16	9
252	B1MAE2	*MAB_p09*	Probable FAD-dependent pyridine nucleotide-disulphide oxidoreductase	1.096	0.335	21	30
253	B1MEU1	*MAB_3471*	Succinate-semialdehyde dehydrogenase	0.607	0.331	12	26
254	B1MP63	*MAB_2032*	Probable 3-oxoacyl-[acyl-carrier protein] reductase	0.539	0.331	4	12
255	B1MNW5	*MAB_1933c*	Glutamine synthetase	0.746	0.33	21	26
256	B1MMS9	*MAB_1556*	Uncharacterized protein	0.813	0.329	4	9
257	B1MJC7	*MAB_0992*	Probable arsenate reductase (ArsC)	0.8	0.329	16	9
258	B1MPC2	*MAB_2092*	Uncharacterized protein	0.952	0.328	21	20
259	B1MID0	*MAB_0858*	Hypothetical luciferase-like monooxygenase	0.605	0.328	15	19
260	B1MPB5	*MAB_2085*	Probable acyl CoA dehydrogenase	3.41	0.328	19	25
261	B1MGI0	*rpsL*	30S ribosomal protein S12	3.031	0.327	6	9
262	B1MG94	*MAB_3765*	Cutinase	0.454	0.323	10	10
263	B1MKJ2	*MAB_1196*	Proline-rich antigen (36 kDa antigen)	0.762	0.322	9	6
264	B1MMP5	*MAB_1521*	HisKA_3 domain-containing protein	1.629	0.319	3	13
265	B1MEA0	*MAB_0064c*	Probable lipase LipE	0.982	0.319	26	26
266	B1MFX3	*MAB_0424*	Putative protease	0.557	0.318	2	10
267	B1MDW3	*mnmA*	tRNA-specific 2-thiouridylase MnmA	0.702	0.318	12	22
268	B1MP99	*MAB_2069*	Uncharacterized protein	0.574	0.317	15	18
269	B1MC96	*MAB_2788*	Probable gamma-glutamyltranspeptidase (GgtB)	0.634	0.316	17	29
270	B1MK84	*MAB_1086*	UTP--glucose-1-phosphate uridylyltransferase	0.776	0.316	15	20
271	B1MNA2	*MAB_1718*	Peptidase U62, modulator of DNA gyrase	1.051	0.315	45	36
272	B1MKL5	*MAB_1219*	Probable short-chain dehydrogenase/reductase	0.546	0.312	9	18
273	B1MNP5	*MAB_1863*	Probable acyl-CoA dehydrogenase FadE	0.587	0.312	19	27
274	B1MGP4	*MAB_0479*	Putative regulatory protein, MarR	0.48	0.311	4	11
275	B1MIA2	*MAB_0830*	Probable NADH-dependent flavin oxidoreductase	1.069	0.308	11	18
276	B1MCL8	*MAB_2911c*	Putative dipeptidase	0.502	0.306	10	21
277	B1MC52	*MAB_2744c*	FeS_assembly_P domain-containing protein	0.783	0.305	7	8
278	B1MH65	*MAB_3872c*	Probable drug-transport integral membrane protein	0.675	0.302	2	12
279	B1MCE4	*nusB*	Transcription antitermination protein NusB	0.619	0.3	4	9
280	B1MIF9	*MAB_4107c*	Glycosyltransferase GtfA	0.826	0.3	18	19

Table S5. Positively enriched proteins captured by VM055*p* probe from pre-incubated VM043-*M. abscessus* S culture through CC-ABPP by LC-ESI-MS/MS analysis, compared to DMSO-treated cells (i.e., non-specific conditions, NS)

	Protein IDs	Gene names	Protein names	[VM043+VM055*p*] *vs.* NS -LOG(p-value)	Fold Change (Log2)	peptides counts all	nb Tryptic Peptides
281	B1MIC9	*MAB_0857*	Putative monooxygenase	1.208	0.299	12	29
282	B1MIF0	*MAB_4098c*	Mycobactin synthetase protein B	0.518	0.297	86	136
283	B1MCX5	*MAB_3018*	Putative transcriptional regulator, GntR family	0.747	0.296	5	18
284	B1MN74	*MAB_1701*	Uncharacterized protein	0.467	0.296	1	20
285	B1MBJ4	*MAB_2534c*	ANTAR domain-containing protein	1.132	0.295	7	16
286	B1MPE0	*MAB_2110*	Uncharacterized protein	0.551	0.294	10	12
287	B1ME73	*MAB_0035c*	Probable penicillin-binding protein PbpA	0.667	0.292	22	29
288	B1MPG2	*hisG*	ATP phosphoribosyltransferase	0.762	0.292	21	22
289	B1MAI1	*pup*	Prokaryotic ubiquitin-like protein Pup	0.425	0.29	6	4
290	B1MMR0	*MAB_1537c*	Putative short chain dehydrogenase/reductase	0.7	0.287	23	19
291	B1MHQ4	*MAB_4061c*	Uncharacterized protein	1.408	0.287	10	15
292	B1MB98	*MAB_2438*	Probable oxidoreductase	0.552	0.285	6	71
293	B1MLU5	*MAB_1436*	Threonine synthase	0.442	0.28	35	23
294	B1MEV1	*MAB_3481*	Probable acyl-CoA dehydrogenase FadE	0.949	0.279	40	35
295	B1MF12	*MAB_3542c*	zf-HC2 domain-containing protein	0.664	0.279	2	7
296	B1MDA0	*MAB_3144*	Putative ABC transporter, ATP-binding protein	0.56	0.279	9	28
297	B1MGQ5	*MAB_0490c*	Putative adenylate cyclase	0.539	0.278	15	32
298	B1MGZ7	*MAB_0583c*	Putative oxidoreductase	0.48	0.277	10	21
299	B1MJA0	*MAB_0964*	Uncharacterized protein	0.631	0.277	13	10
300	B1MC67	*MAB_2759*	Transketolase	0.591	0.276	58	41
301	B1MH24	*MAB_0610*	Uncharacterized protein	0.804	0.275	7	11
302	B1MM07	*MAB_1498c*	Putative GntR-family transcriptional regulator	0.929	0.275	6	21
303	B1MF16	*aroA*	3-phosphoshikimate 1-carboxyvinyltransferase	0.783	0.274	21	22
304	B1MEI7	*MAB_0151c*	Septum_form domain-containing protein	0.937	0.269	16	21
305	B1MCE2	*MAB_2834c*	Methyltranfer_dom domain-containing protein	0.561	0.268	8	16
306	B1MN07	*rpsT*	30S ribosomal protein S20	0.606	0.268	4	7
307	B1MFY8	*MAB_3659c*	Peptidase M20 domain-containing protein 2	0.587	0.267	10	14
308	B1ME48	*MAB_0009*	Uncharacterized protein	0.594	0.265	6	10
309	B1MIB9	*MAB_0847c*	Probable aminotransferase	1.101	0.262	19	19
310	B1MFF2	*MAB_0252*	Uncharacterized protein	0.852	0.26	15	41
311	B1MN52	*MAB_1679c*	Probable ArsR-family transcriptional regulator	0.551	0.259	3	8
312	B1MGW1	*MAB_0546*	Probable ATP-dependent Clp protease ATP-binding subunit	0.543	0.259	69	57
313	B1MF70	*MAB_3601c*	Mannose-6-phosphate isomerase	0.917	0.259	17	23
314	B1MF62	*MAB_3593*	Possible Mg2+ transport P-type ATPase C MgtC	0.825	0.259	10	14
315	B1MCN2	*MAB_2925c*	Xanthine dehydrogenase family protein	0.7	0.258	14	54
316	B1MMB6	*MAB_4833c*	Uncharacterized protein	0.51	0.258	10	12
317	B1MJ81	*MAB_0945*	Putative drug resistance transporter, EmrB/QacA family	0.639	0.257	2	14
318	B1MFV4	*MAB_0405c*	Uncharacterized protein	0.683	0.256	8	9
319	B1MKB8	*MAB_1122c*	Dolichyl-phosphate-mannose--protein mannosyltransferase	0.738	0.256	11	23
320	B1MK68	*trmB*	tRNA (guanine-N(7)-)-methyltransferase	1.64	0.255	9	17

Table S5. **Positively enriched proteins captured by VM055*p* probe from pre-incubated VM043-*M. abscessus* S culture through CC-ABPP by LC-ESI-MS/MS analysis, compared to DMSO-treated cells (i.e., non-specific conditions, NS)**

	Protein IDs	Gene names	Protein names	[VM043+VM055*p*] *vs.* NS		peptides counts all	nb Tryptic Peptides
				-LOG(p-value)	Fold Change (Log2)		
321	B1MMV9	*MAB_1586c*	Uncharacterized protein	0.615	0.255	5	6
322	B1MGV4	*MAB_0539*	Conserved hypothetical transmembrane protein	0.602	0.255	14	19
323	B1MG80	*rpsI*	30S ribosomal protein S9	0.721	0.255	8	12
324	B1MM70	*MAB_4787c*	Hypothetical regulatory protein, TetR family	0.904	0.254	9	10
325	B1MKH2	*MAB_1176c*	Uncharacterized protein	0.433	0.254	2	10
326	B1MBX5	*priA*	Phosphoribosyl isomerase A	1.032	0.253	12	16
327	B1MGE5	*rplV*	50S ribosomal protein L22	0.644	0.253	7	10
328	B1MK06	*MAB_4443*	Probable 3-oxoacyl-[acyl-carrier protein] reductase	1.045	0.253	38	30
329	B1MM21	*MAB_4738c*	Putative amidohydrolase	0.67	0.253	19	21
330	B1MBD0	*katG*	Catalase-peroxidase	0.548	0.251	52	48
331	B1ME23	*MAB_3416*	Probable membrane transport protein	0.609	0.25	4	11
332	B1MAY2	*rpmI*	50S ribosomal protein L35	0.535	0.25	2	4
333	B1MNV2	*MAB_1920*	Glutamine synthetase	0.665	0.25	21	23
334	B1MDE8	*MAB_3192c*	Putative glycosyl hydrolase (Beta-glucosidase)	0.524	0.249	25	27
335	B1MAY5	*MAB_2325*	Putative short-chain dehydrogenase/reductase	0.777	0.249	18	21
336	B1MIX8	*MAB_4276c*	Probable conserved lipoprotein DsbF	0.638	0.248	8	12
337	B1MLH6	*MAB_1315*	Putative lipoprotein LpqW	0.663	0.248	14	29
338	B1MP01	*trpD*	Anthranilate phosphoribosyltransferase	0.853	0.247	12	18
339	B1ML82	*MAB_4658*	Putative transcriptional regulator, LysR family	0.649	0.247	8	20
340	B1ML14	*MAB_4590*	Xanthosine permease	0.555	0.247	1	13
341	B1MEU5	*ftsE*	Cell division ATP-binding protein FtsE	0.734	0.246	15	19
342	B1MAJ7	*tatA*	Sec-independent protein translocase protein TatA	0.606	0.245	2	4
343	B1MH12	*MAB_0598*	Short-chain dehydrogenase/reductase	0.631	0.244	24	16
344	B1MCG5	*MAB_2857c*	TGc domain-containing protein	0.65	0.244	8	19
345	B1MAK2	*MAB_2192*	Probable dipeptidase PepE	0.726	0.244	17	18
346	B1MNT5	*MAB_1903*	GTP cyclohydrolase 1 type 2 homolog	0.537	0.242	14	16
347	B1MFZ8	*MAB_3669*	Uncharacterized protein	1.098	0.241	10	18
348	B1MJ75	*MAB_0939*	Probable polyketide synthase	0.489	0.236	140	199
349	B1MJ83	*MAB_0947c*	Putative luciferase	0.633	0.236	6	21
350	B1MDK3	*smc*	Chromosome partition protein Smc	0.662	0.235	52	85
351	B1MD79	*MAB_3123*	Putative acyl-CoA dehydrogenase	0.682	0.234	13	20
352	B1MBF6	*MAB_2496*	Probable acyl-CoA dehydrogenase	0.457	0.233	7	22
353	B1MG28	*MAB_3699c*	Uncharacterized protein	1.626	0.233	18	16
354	B1MAY1	*MAB_2321*	Translation initiation factor IF-3	1.368	0.232	15	13
355	B1MLB4	*MAB_4691c*	Mycobactin synthetase protein B	0.481	0.232	20	390
356	B1MIN4	*cysC*	Adenylyl-sulfate kinase	0.532	0.232	6	38
357	B1MLG6	*MAB_1305*	Probable short-chain dehydrogenase/reductase	0.808	0.232	17	19
358	B1MGB5	*MAB_3785c*	Probable conserved lipoprotein LppF	0.682	0.23	19	26
359	B1MKA9	*MAB_1113c*	Putative_PNPOx domain-containing protein	0.444	0.229	6	9
360	B1MGD6	*rplX*	50S ribosomal protein L24	0.595	0.229	6	6

Table S5. Positively enriched proteins captured by VM055*p* probe from pre-incubated VM043-*M. abscessus* S culture through CC-ABPP by LC-ESI-MS/MS analysis, compared to DMSO-treated cells (i.e., non-specific conditions, NS)

	Protein IDs	Gene names	Protein names	[VM043+VM055*p*] *vs.* NS -LOG(p-value)	[VM043+VM055*p*] *vs.* NS Fold Change (Log2)	peptides counts all	nb Tryptic Peptides
361	B1MP15	*MAB_1984*	Probable 1-acylglycerol-3-phosphate O-acyltransferase	0.69	0.227	19	18
362	B1MDF1	*tsf*	Elongation factor Ts	0.473	0.227	40	19
363	B1MCB9	*fmt*	Methionyl-tRNA formyltransferase	0.419	0.224	12	16
364	B1MGG7	*MAB_3838c*	Putative ferredoxin reductase	0.756	0.224	22	23
365	B1MLF1	*MAB_4728c*	DNA-(apurinic or apyrimidinic site) lyase	0.471	0.223	6	21
366	B1MP41	*MAB_2010*	Purine nucleoside phosphorylase	0.359	0.223	9	13
367	B1MDV0	*MAB_3343*	ACT domain-containing protein	0.382	0.222	8	11
368	B1MKL2	*MAB_1216c*	Probable cytochrome P450	0.544	0.221	6	32
369	B1MG68	*MAB_3739c*	Alanine racemase	1.457	0.22	9	18
370	B1MG39	*MAB_3710*	Possible multi-functional enzyme with acyl-CoA-reductase activity AcrA1	0.927	0.219	26	45
371	B1MFI5	*MAB_0285*	Putative oxidoreductase	0.76	0.218	7	20
372	B1MC57	*MAB_2749c*	Putative FeS assembly protein SufB	2.292	0.218	44	33
373	B1MHG4	*MAB_3971*	Uncharacterized protein	0.467	0.216	3	4
374	B1MKH5	*MAB_1179c*	Uncharacterized protein	0.933	0.215	5	9
375	B1MC29	*cpfC*	Coproporphyrin III ferrochelatase	0.715	0.214	19	19
376	B1MMT3	*ettA*	Energy-dependent translational throttle protein EttA	1.432	0.214	39	33
377	B1MJX1	*MAB_4408c*	Alkyl hydroperoxide reductase C	0.579	0.213	21	13
378	B1MP20	*MAB_1989c*	Uncharacterized protein	0.76	0.213	10	9
379	B1MIP4	*MAB_4192*	Uncharacterized protein	0.412	0.212	9	13
380	B1MGD0	*MAB_3800c*	Glutamate dehydrogenase	0.495	0.21	33	34
381	B1MFZ4	*MAB_3665*	Uncharacterized protein	0.677	0.21	7	9
382	B1ME28	*MAB_3421*	Uncharacterized protein	0.727	0.21	9	23
383	B1MCT8	*MAB_2981c*	Putative liporotein LppU	0.566	0.208	4	9
384	B1MHJ9	*MAB_4006*	Putative lipase/esterase/beta-lactamase	0.453	0.207	10	27
385	B1MFL6	*MAB_0317*	Uncharacterized protein	0.625	0.207	9	9
386	B1MDQ2	*MAB_3295*	Putative transcriptional regulator, IclR family	0.83	0.207	11	18
387	B1MD03	*MAB_3046*	Uncharacterized protein	0.79	0.207	45	36
388	B1MN48	*recO*	DNA repair protein RecO	0.431	0.207	9	17
389	B1ME04	*MAB_3397*	HTH tetR-type domain-containing protein	0.495	0.207	5	11
390	B1MDU0	*MAB_3333*	Probable conserved lipoprotein LppZ	0.591	0.206	8	19
391	B1MM34	*MAB_4751*	PNPLA domain-containing protein	0.516	0.206	16	19
392	B1MI15	*MAB_0741c*	DUF4395 domain-containing protein	0.395	0.205	4	5
393	B1MEZ4	*MAB_3524c*	Uncharacterized protein	0.511	0.205	13	20
394	B1MC58	*MAB_2750c*	Probable transcriptional regulatory protein	0.886	0.205	9	12
395	B1MG21	*folD*	Bifunctional protein FolD	0.543	0.204	20	20
396	B1MNN1	*MAB_1849*	Neutral metalloproteinase	0.404	0.204	6	23
397	B1MMX4	*MAB_1601c*	Putative monooxygenase	0.454	0.202	11	26
398	B1MIL6	*MAB_4164*	Possible enoyl-CoA hydratase	0.695	0.202	5	19
399	B1MDW8	*MAB_3361*	Putative transcriptional regulator, LuxR family	0.519	0.201	11	57
400	B1MMI1	*MAB_4898c*	Single-stranded DNA-binding protein	0.43	0.201	11	12

Table S5. **Positively enriched proteins captured by VM055*p* probe from pre-incubated VM043-*M. abscessus* S culture through CC-ABPP by LC-ESI-MS/MS analysis, compared to DMSO-treated cells (i.e., non-specific conditions, NS)**

				[VM043+VM055*p*] *vs.* NS			
	**Protein IDs**	**Gene names**	**Protein names**	**-LOG(p-value)**	**Fold Change (Log2)**	**peptides counts all**	**nb Tryptic Peptides**
401	B1MFL7	*MAB_0318c*	MurNAc-LAA domain-containing protein	0.549	0.201	12	12
402	B1MN03	*MAB_1630c*	Uncharacterized protein	0.37	0.2	2	7
403	B1MPG8	*MAB_2138*	NADH-quinone oxidoreductase, E subunit NuoE	0.403	0.2	5	14
404	B1MCZ9	*hflX*	GTPase HflX	1.268	0.199	11	26
405	B1MN96	*rnpA*	Ribonuclease P protein component	0.532	0.198	3	11
406	B1MDE4	*pyrH*	Uridylate kinase	0.648	0.197	15	16
407	B1MMU6	*MAB_1573c*	Uncharacterized protein	0.427	0.196	5	13
408	B1MEL6	*MAB_0180*	Polyketide synthase PKS13	0.476	0.195	105	97
409	B1MD90	*MAB_3134c*	Uncharacterized protein	1.104	0.195	7	9
410	B1MEL5	*MAB_0179*	Probable fatty-acid-CoA ligase FadD	0.617	0.195	43	35
411	B1MDB8	*MAB_3162c*	Uncharacterized protein	0.454	0.193	8	15
412	B1MAL3	*MAB_2203*	Putative_PNPOx domain-containing protein	0.85	0.191	5	10
413	B1MIQ0	*MAB_4198*	Uncharacterized protein	1.4	0.191	10	14
414	B1MAM3	*MAB_2213*	Putative thioesterase	0.481	0.187	8	15
415	B1MEF1	*MAB_0115c*	Uncharacterized protein	0.614	0.186	20	12
416	B1MH07	*MAB_0593c*	Probable acyl-CoA dehydrogenase FadE	0.701	0.186	11	15
417	B1MMS2	*MAB_1549c*	Putative RNA binding protein, contains S1 domain	0.61	0.185	38	55
418	B1MGI6	*MAB_3857c*	Probable enoyl-coa hydratase/isomerase	0.811	0.185	5	14
419	B1MB81	*MAB_2421c*	PknH_C domain-containing protein	0.968	0.184	17	20
420	B1MCE5	*efp*	Elongation factor P	0.457	0.184	4	10
421	B1MFP3	*asd*	Aspartate-semialdehyde dehydrogenase	0.613	0.184	30	20
422	B1MGR8	*MAB_0503*	Probable UDP-glucose 4-epimerase GalE1	0.691	0.18	24	22
423	B1MIX3	*dnaJ*	Chaperone protein DnaJ	0.776	0.179	43	27
424	B1MAH2	*mpa*	Proteasome-associated ATPase	1.846	0.179	38	48
425	B1MAG3	*MAB_2153*	MOSC domain-containing protein	0.546	0.179	3	16
426	B1MDH4	*MAB_3219*	Uncharacterized protein	0.465	0.178	8	7
427	B1MCS6	*MAB_2969*	Putative ATP-binding protein	0.415	0.178	18	26
428	B1MBN7	*MAB_2577*	Putative TetR-family transcriptional regulator	0.647	0.177	10	15
429	B1MAM1	*MAB_2211c*	Putative membrane protein, MmpS	0.425	0.176	3	6
430	B1MFU8	*recB*	RecBCD enzyme subunit RecB	0.577	0.176	31	65
431	B1MB28	*MAB_2368*	Uncharacterized protein	0.39	0.176	2	14
432	B1MCU9	*MAB_2992c*	TRAM domain-containing protein	0.621	0.175	17	25
433	B1MJ15	*MAB_0877c*	Uncharacterized protein	0.398	0.175	5	12
434	B1MCD5	*carB*	Carbamoyl-phosphate synthase large chain	0.518	0.174	84	62
435	B1MBE6	*MAB_2486c*	Glutamate synthase, large subunit	0.505	0.172	37	103
436	B1MD24	*MAB_3067c*	Uncharacterized protein	0.601	0.171	37	19
437	B1MLQ2	*MAB_1393c*	2-hydroxy-3-oxoadipate synthase	0.498	0.171	103	73
438	B1MM06	*tetR*	DNA-binding transcriptional repressor TetR	0.446	0.17	4	14
439	B1ME45	*gyrB*	DNA gyrase subunit B	1.266	0.17	61	43
440	B1MJA6	*MAB_0970c*	Probable drug resistance transporter	0.771	0.169	15	26

Table S5. **Positively enriched proteins captured by VM055*p* probe from pre-incubated VM043-*M. abscessus* S culture through CC-ABPP by LC-ESI-MS/MS analysis, compared to DMSO-treated cells (i.e., non-specific conditions, NS)**

	Protein IDs	Gene names	Protein names	[VM043+VM055*p*] *vs.* NS		peptides counts all	nb Tryptic Peptides
				-LOG(p-value)	Fold Change (Log2)		
441	B1MKJ1	*MAB_1195*	Beta-thionase	0.43	0.168	32	23
442	B1MAW8	*uvrB*	UvrABC system protein B	0.841	0.166	40	51
443	B1MN84	*MAB_1711c*	DUF5642 domain-containing protein	0.497	0.166	14	16
444	B1MDB9	*cobQ*	Cobyric acid synthase	0.631	0.164	20	29
445	B1MPF9	*MAB_2129*	Methionine synthase	0.52	0.164	66	73
446	B1ML08	*MAB_4584c*	Putative S-adenosyl-L-methionine-dependent methyltransferase MAB_4584c	0.532	0.163	15	21
447	B1MJM7	*MAB_4312*	Putative TetR-family transcriptional regulator	0.623	0.163	2	13
448	B1MLV3	*MAB_1444*	YrdC-like domain-containing protein	0.608	0.162	7	12
449	B1MKC1	*MAB_1125c*	Hypothetical acetyltransferase, GNAT family	0.733	0.162	13	19
450	B1MIP9	*MAB_4197*	Hydroxymethylpyrimidine kinase	0.396	0.162	12	12
451	B1MEC1	*MAB_0085c*	Putative transcriptional regulator	0.589	0.161	2	15
452	B1MM47	*MAB_4764c*	Uncharacterized protein	0.478	0.158	10	23
453	B1MDQ1	*leuC*	3-isopropylmalate dehydratase large subunit	0.637	0.158	50	28
454	B1MBY8	*MAB_2679*	Uncharacterized protein	0.461	0.158	7	35
455	B1MCV4	*MAB_2997c*	RecG_wedge domain-containing protein	0.843	0.158	13	12
456	B1MLR8	*MAB_1409c*	Putative drug antiporter protein	0.38	0.155	3	13
457	B1ME20	*MAB_3413c*	Ribonucleoside-diphosphate reductase	0.486	0.154	51	46
458	B1MH39	*MAB_0625*	Acetaldehyde dehydrogenase 1	0.505	0.154	11	16
459	B1MI36	*MAB_0762*	Uncharacterized protein	0.405	0.154	17	25
460	B1MN97	*rpmH*	50S ribosomal protein L34	0.424	0.154	1	3
461	B1MLV1	*prfA*	Peptide chain release factor 1	0.619	0.153	20	24
462	B1MMT4	*MAB_1561*	Glutamate dehydrogenase	0.465	0.152	105	108
463	B1MEP1	*MAB_0205c*	Uncharacterized protein	0.539	0.151	12	25
464	B1MCW6	*sigA*	RNA polymerase sigma factor SigA	1.015	0.148	32	26
465	B1MIN3	*MAB_4181*	ATP-sulfurylase small subunit	0.488	0.148	7	20
466	B1MDM7	*MAB_3270c*	Probable lipase/esterase LipN	0.613	0.148	23	22
467	B1MES0	*MAB_3450c*	Probable phosphoglucomutase PgmA	0.634	0.148	27	35
468	B1MAK8	*MAB_2198c*	Probable cobalamin biosynthesis protein CobI	0.503	0.148	14	32
469	B1MG02	*MAB_3673*	Probable succinate dehydrogenase (Cytochrome b-556 subunit) SdhC	0.488	0.147	6	8
470	B1MGT4	*MAB_0519*	Peptidase S13 (D-alanyl-D-alanine carboxypeptidase)	0.566	0.147	31	28
471	B1MNX3	*lipA*	Lipoyl synthase	0.674	0.147	19	19
472	B1MJK4	*MAB_1070c*	Probable acyl-CoA dehydrogenase	0.574	0.147	29	23
473	B1MF49	*secA*	Protein translocase subunit SecA	0.589	0.145	81	60
474	B1MFX5	*MAB_0426*	Probable peptide ABC transporter DppA	0.662	0.144	19	25
475	B1MCI2	*MAB_2874*	Probable peptidyl-prolyl cis-trans isomerase	0.413	0.143	17	14
476	B1MIS7	*ackA*	Acetate kinase	0.439	0.142	19	21
477	B1MK13	*MAB_4451c*	Uncharacterized protein	0.48	0.142	1	17
478	B1MK05	*MAB_4442c*	Probable acetyl-CoA acyltransferase	0.605	0.142	31	27
479	B1MM01	*MAB_1492*	Uncharacterized protein	0.455	0.141	63	74
480	B1MJ64	*serC*	Phosphoserine aminotransferase	0.38	0.141	19	20

Table S5. Positively enriched proteins captured by VM055*p* probe from pre-incubated VM043-*M. abscessus* S culture through CC-ABPP by LC-ESI-MS/MS analysis, compared to DMSO-treated cells (i.e., non-specific conditions, NS)

	Protein IDs	Gene names	Protein names	[VM043+VM055*p*] *vs.* NS		peptides counts all	nb Tryptic Peptides
				-LOG(p-value)	Fold Change (Log2)		
481	B1MKG0	*MAB_1164*	Putative conserved lipoprotein LpqU	0.442	0.14	7	16
482	B1MHX5	*MAB_0700c*	Putative Dyp-type peroxidase	0.406	0.14	4	16
483	B1MGS5	*MAB_0510c*	Probable non-ribosomal peptide synthase	0.481	0.139	41	72
484	B1MHH1	*hemL*	Glutamate-1-semialdehyde 2,1-aminomutase	0.681	0.139	21	25
485	B1MET9	*MAB_3469*	MutT/NUDIX family protein	0.608	0.139	4	8
486	B1MK61	*MAB_4499*	Histidine kinase	0.62	0.139	9	35
487	B1MKW1	*MAB_4537c*	Uncharacterized protein	0.498	0.138	21	15
488	B1MEV3	*MAB_3483*	Putative oxygenase	0.514	0.138	21	33
489	B1MJ91	*MAB_0955c*	Probable sensor histidine kinase PrrB	0.431	0.138	18	29
490	B1MKY5	*MAB_4561*	Uncharacterized protein	0.438	0.138	1	17
491	B1MFP2	*MAB_0343*	Aspartokinase	0.677	0.138	35	29
492	B1MKH7	*MAB_1181c*	Putative lipoprotein LpqV	0.46	0.137	3	6
493	B1MDX4	*MAB_3367*	Putative fatty-acid-CoA ligase	0.5	0.137	49	67
494	B1MJV4	*MAB_4390*	Putative ABC transporter, periplasmic substrate-binding	0.468	0.135	16	19
495	B1MHH0	*MAB_3977c*	Uncharacterized protein	0.972	0.134	7	17
496	B1MHI4	*MAB_3991c*	Possible Uroporphyrin-III C-methyltransferase	0.82	0.134	36	31
497	B1MIJ2	*MAB_4140*	Uncharacterized protein	0.563	0.133	18	12
498	B1MLM3	*MAB_1364*	Probable serine protease HtrA	0.512	0.132	21	32
499	B1MIF2	*MAB_4100c*	MbtH-like protein	0.366	0.132	3	5
500	B1MMP6	*MAB_1522*	Putative two-component system response regulator LuxR	0.42	0.131	7	14
501	B1MLF7	*MAB_1296*	Uncharacterized protein	0.418	0.131	4	6
502	B1MMN6	*MAB_1512*	Probable fatty acid synthase Fas	0.365	0.129	114	161
503	B1MHJ1	*MAB_3998*	Uncharacterized protein	0.819	0.129	34	33
504	B1MB54	*MAB_2394*	Uncharacterized protein	0.39	0.128	3	10
505	B1MJ19	*MAB_0881*	Hypothetical tRNA/rRNA methyltransferase	0.513	0.126	16	21
506	B1MMB5	*MAB_4832c*	Uncharacterized protein	0.52	0.125	3	11
507	B1MNE2	*MAB_1758*	Uncharacterized protein	0.423	0.125	1	3
508	B1ME59	*MAB_0020*	DUF3566 domain-containing protein	0.384	0.123	12	11
509	B1MCN1	*MAB_2924c*	Uncharacterized protein	0.485	0.123	5	22
510	B1MFU5	*MAB_0396c*	Uncharacterized protein	0.543	0.122	8	11
511	B1MKR2	*ychF*	Ribosome-binding ATPase YchF	0.614	0.121	24	25
512	B1MH70	*rplJ*	50S ribosomal protein L10	0.375	0.121	11	17
513	B1ME46	*MAB_0007*	Uncharacterized protein	0.546	0.12	21	31
514	B1MN01	*MAB_1628c*	FAD_binding_3 domain-containing protein	0.415	0.117	23	33
515	B1MGC6	*rplR*	50S ribosomal protein L18	0.418	0.117	7	11
516	B1MD89	*MAB_3133c*	Flavohemoglobin	0.418	0.115	10	23
517	B1MK58	*MAB_4496c*	Luciferase-like monooxygenase	0.557	0.112	13	12
518	B1MCN5	*MAB_2928*	Guanine deaminase	0.734	0.111	15	26
519	B1MEZ6	*MAB_3526c*	ZnMc domain-containing protein	0.416	0.11	15	22
520	B1ME42	*gnD*	6-phosphogluconate dehydrogenase Gnd	0.434	0.11	22	19

Table S5. Positively enriched proteins captured by VM055*p* probe from pre-incubated VM043-*M. abscessus* S culture through CC-ABPP by LC-ESI-MS/MS analysis, compared to DMSO-treated cells (i.e., non-specific conditions, NS)

	Protein IDs	Gene names	Protein names	[VM043+VM055*p*] *vs.* NS -LOG(p-value)	[VM043+VM055*p*] *vs.* NS Fold Change (Log2)	peptides counts all	nb Tryptic Peptides
521	B1MHY2	*MAB_0708*	Uncharacterized protein	0.355	0.11	19	30
522	B1MIZ5	*MAB_4293*	Putative Fe-S oxidoreductase	0.483	0.11	70	49
523	B1MJN4	*MAB_4319*	Uncharacterized protein	0.401	0.11	3	6
524	B1MMI6	*MAB_4903*	Uncharacterized protein	0.412	0.109	3	7
525	B1MCF8	*MAB_2850c*	Putative pre-16S rRNA nuclease	0.527	0.109	7	14
526	B1MH92	*rpmG2*	50S ribosomal protein L33 2	0.398	0.108	14	5
527	B1MLU7	*rho*	Transcription termination factor Rho	0.667	0.108	64	41
528	B1MH14	*MAB_0600*	Probable acetyl-CoA acetyltransferase FadA	0.52	0.108	30	28
529	B1MHE5	*MAB_3952*	Possible O-succinylbenzoic acid--CoA ligase MenE	0.473	0.106	13	18
530	B1MC16	*MAB_2708*	Uncharacterized protein	0.467	0.106	6	25
531	B1MCF6	*MAB_2848c*	Probable shikimate-5-dehydrogenase (AroE)	0.465	0.104	5	14
532	B1MHA9	*MAB_3916c*	Probable conserved secreted protein	0.37	0.104	4	13
533	B1ME64	*MAB_0026*	Rhomboid family protein	0.423	0.104	7	12
534	B1MBX9	*hisD*	Histidinol dehydrogenase	0.435	0.104	25	19
535	B1MDN6	*MAB_3279c*	Putative phosphatase/kinase	0.582	0.104	23	28
536	B1MFI3	*MAB_0283c*	Uncharacterized protein	0.429	0.103	7	13
537	B1MDX9	*MAB_3372*	Putative transcriptional regulator, TetR family	0.36	0.103	1	12
538	B1MKS3	*MAB_1277*	PMT_2 domain-containing protein	0.371	0.103	4	25
539	B1MC08	*MAB_2699c*	Pseudouridine synthase	0.541	0.103	15	22
540	B1MKD5	*ispE*	4-diphosphocytidyl-2-C-methyl-D-erythritol kinase	0.377	0.101	9	14
541	B1MC59	*MAB_2751*	Uncharacterized protein	0.418	0.1	13	25
542	B1MH37	*MAB_0623*	Probable dehydrogenase	0.393	0.1	8	35
543	B1MIT2	*fgd*	F420-dependent glucose-6-phosphate dehydrogenase	0.772	0.1	29	24
544	B1MF81	*MAB_3612c*	Putative dTDP-rhamnosyltransferase	0.421	0.1	11	18
545	B1MMI8	*MAB_4905*	Uncharacterized protein	0.548	0.099	8	18
546	B1MDT4	*MAB_3327c*	Putative transcriptional regulator, AsnC family	0.479	0.098	7	9
547	B1MFG6	*MAB_0266c*	Probable prephenate dehydrogenase TyrA	0.63	0.098	13	16
548	B1MNQ7	*MAB_1875c*	Putative hydrolase (Alpha/beta fold)	0.553	0.098	5	14
549	B1MAL1	*MAB_2201*	Probable cobalamin biosynthesis protein CobN	0.379	0.097	56	65
550	B1MKN1	*MAB_1235*	Uncharacterized protein	0.464	0.097	26	29
551	B1MC69	*zwf*	Glucose-6-phosphate 1-dehydrogenase	0.669	0.097	29	30
552	B1MNP7	*MAB_1865*	Probable fatty-acid-CoA ligase FadD	0.416	0.095	5	30
553	B1MBG5	*MAB_2505c*	Probable phosphoketolase	0.442	0.095	8	47
554	B1MEG4	*MAB_0128c*	Possible transcriptional regulator	0.422	0.093	2	14
555	B1MC56	*MAB_2748c*	Uncharacterized protein	0.592	0.093	22	26
556	B1MLN7	*corA*	Magnesium transport protein CorA	0.393	0.092	27	21
557	B1MHX4	*MAB_0699c*	Probable 29 kDa antigen CFP29 (Bacteriocin CFP29)	0.462	0.092	16	17
558	B1MF96	*MAB_3628c*	Possible fatty-acid-CoA ligase FadD	0.481	0.092	9	33
559	B1MKH9	*MAB_1183*	Rhodanese domain-containing protein	0.377	0.091	3	8
560	B1MN46	*MAB_1673*	DUF559 domain-containing protein	0.382	0.09	2	18

Table S5. Positively enriched proteins captured by VM055*p* probe from pre-incubated VM043-*M. abscessus* S culture through CC-ABPP by LC-ESI-MS/MS analysis, compared to DMSO-treated cells (i.e., non-specific conditions, NS)

	Protein IDs	Gene names	Protein names	[VM043+VM055*p*] *vs.* NS		peptides counts all	nb Tryptic Peptides
				-LOG(p-value)	Fold Change (Log2)		
561	B1MGE6	*rpsS*	30S ribosomal protein S19	0.404	0.09	7	5
562	B1MEY6	*MAB_3516c*	DNA helicase	0.403	0.09	17	66
563	B1MDP7	*ppk*	Polyphosphate kinase	0.483	0.09	42	46
564	B1MJB7	*MAB_0981c*	Possible enoyl-CoA hydratase/isomerase	0.394	0.09	14	14
565	B1MGF5	*MAB_3825*	Cytochrome P450	0.407	0.089	8	25
566	B1MB72	*MAB_2412c*	Probable IMP dehydrogenase family protein	0.576	0.088	32	27
567	B1MEL3	*MAB_0177*	Antigen 85-A/B/C	0.495	0.088	13	13
568	B1MN32	*MAB_1659c*	ATP-sulfurylase large subunit	0.552	0.087	21	28
569	B1MLL3	*glgC*	Glucose-1-phosphate adenylyltransferase	0.525	0.087	16	25
570	B1MH77	*MAB_3884*	Possible flavoprotein	0.412	0.087	21	32
571	B1MGH2	*MAB_3843*	Uncharacterized protein	0.375	0.087	14	19
572	B1MBH3	*MAB_2513c*	Anti-sigma factor RsbW	0.528	0.086	13	9
573	B1MLQ7	*MAB_1398c*	Putative oxidoreductase	0.466	0.086	22	28
574	B1MKC8	*MAB_1132c*	Probable L-asparagine permease 1 (Transport protein)	0.377	0.086	9	21
575	B1MJS9	*MAB_4365c*	Putative dihydrodiol dehydrogenase	0.602	0.085	4	18
576	B1MMJ3	*MAB_4910c*	Putative aminoglycoside phosphotransferase	0.431	0.084	4	13
577	B1MDJ8	*ftsY*	Signal recognition particle receptor FtsY	0.379	0.084	13	23
578	B1MMG7	*MAB_4884c*	Uncharacterized protein	0.336	0.083	7	11
579	B1MB73	*MAB_2413c*	6-phosphogluconate dehydrogenase, decarboxylating	0.557	0.083	37	32
580	B1MNT8	*MAB_1906*	Pyridoxamine 5'-phosphate oxidase-related	0.48	0.082	15	17
581	B1ME24	*MAB_3417c*	Probable NADPH-dependent FMN reductase	0.352	0.082	6	10
582	B1MB80	*MAB_2420c*	PknH_C domain-containing protein	0.495	0.081	14	16
583	B1MGZ8	*MAB_0584*	Acyl-CoA_dh_2 domain-containing protein	0.448	0.081	23	20
584	B1MEL7	*MAB_0181*	Propionyl-CoA carboxylase, beta subunit (PccB)	0.443	0.08	54	34
585	B1MH62	*rpoB*	DNA-directed RNA polymerase subunit beta	0.417	0.08	130	81
586	B1MK27	*MAB_4465*	Tox-REase-7 domain-containing protein	0.489	0.08	14	35
587	B1MIG8	*MAB_4116c*	Putative membrane protein, MmpL	0.483	0.079	40	58
588	B1MF13	*MAB_3543c*	RNA polymerase sigma factor	0.39	0.078	11	17
589	B1MHI8	*MAB_3995*	Uncharacterized protein	0.588	0.078	3	16
590	B1MLI6	*MAB_1325c*	Probable NADPH dependent 2,4-dienoyl-CoA reductase FadH	0.426	0.078	52	46
591	B1MM41	*MAB_4758*	Hypothetical patatin-like protein	0.374	0.078	4	13
592	B1MEQ1	*MAB_0215*	Possible transcriptional regulator	0.343	0.077	5	14
593	B1MLY3	*MAB_1474c*	Nicotinate phosphoribosyltransferase	0.491	0.076	23	27
594	B1MJ09	*moaA*	GTP 3',8-cyclase	0.479	0.075	19	26
595	B1MHH4	*MAB_3981c*	Probable zinc metalloprotease	0.407	0.074	33	42
596	B1MJI8	*MAB_1054*	ATP-dependent DNA helicase	0.418	0.073	46	54
597	B1MM63	*MAB_4780*	MaoC-like dehydratase	0.407	0.07	24	15
598	B1MK70	*MAB_4508*	Putative membrane protein, MmpL	0.401	0.07	79	60
599	B1MGY1	*MAB_0567c*	Putative lipoprotein lpqE	0.338	0.068	13	11
600	B1MAX2	*MAB_2312*	Usp domain-containing protein	0.395	0.068	19	10

Table S5. **Positively enriched proteins captured by VM055*p* probe from pre-incubated VM043-*M. abscessus* S culture through CC-ABPP by LC-ESI-MS/MS analysis, compared to DMSO-treated cells (i.e., non-specific conditions, NS)**

	Protein IDs	Gene names	Protein names	[VM043+VM055*p*] *vs.* NS -LOG(p-value)	Fold Change (Log2)	peptides counts all	nb Tryptic Peptides
601	B1MKX2	*MAB_4548*	Probable O-methyltransferase	0.348	0.068	10	16
602	B1MKF8	*MAB_1162c*	Uncharacterized protein	0.429	0.068	24	26
603	B1MDB1	*cobB*	Hydrogenobyrinate a,c-diamide synthase	0.522	0.068	15	29
604	B1MIE7	*MAB_4095c*	Isocitrase	0.497	0.067	48	30
605	B1MD87	*infB*	Translation initiation factor IF-2	0.49	0.066	55	37
606	B1MD64	*rpsO*	30S ribosomal protein S15	0.381	0.066	14	7
607	B1ML15	*MAB_4591*	Putative phosphotyrosine protein phosphatase	0.442	0.066	14	19
608	B1MCL6	*MAB_2908c*	2-dehydropantoate 2-reductase	0.33	0.064	14	22
609	B1MAH0	*MAB_2160c*	Putative lipoprotein LppK	0.331	0.063	9	10
610	B1MJB5	*MAB_0979*	Putative HTH-type transcriptional regulator MarR	0.355	0.063	10	13
611	B1MBG3	*MAB_2503c*	Putative iron-sulfur binding oxidoreductase	0.369	0.063	2	30
612	B1MET0	*MAB_3460c*	Uncharacterized protein	0.335	0.062	4	16
613	B1MKQ3	*ispH*	4-hydroxy-3-methylbut-2-enyl diphosphate reductase	0.608	0.061	17	20
614	B1MLR3	*MAB_1404*	Probable acyltransferase	0.46	0.061	5	19
615	B1ME98	*MAB_0062*	Pyridine nucleotide-disulphide oxidoreductase family	0.422	0.06	17	29
616	B1MC25	*MAB_2717c*	Uncharacterized protein	0.356	0.06	21	26
617	B1MIE8	*MAB_4096c*	Uncharacterized protein	0.414	0.059	15	22
618	B1MIG6	*MAB_4114*	Uncharacterized protein	0.367	0.059	8	4
619	B1MC93	*MAB_2785*	Uncharacterized protein	0.341	0.059	2	4
620	B1MP42	*MAB_2011*	Pyridoxal phosphate homeostasis protein	0.369	0.059	22	19
621	B1MDN5	*recG*	ATP-dependent DNA helicase RecG	0.57	0.058	20	46
622	B1MMP9	*MAB_1525c*	Putative transcriptional regulator, TetR family	0.401	0.057	5	13
623	B1MCG9	*MAB_2861*	Uncharacterized protein	0.368	0.057	3	14
624	B1MFB7	*MAB_3649*	Probable aldehyde dehydrogenase	0.436	0.057	26	31
625	B1MKD8	*pth*	Peptidyl-tRNA hydrolase	0.38	0.056	11	13
626	B1MIU6	*MAB_4244c*	Uncharacterized protein	0.338	0.056	7	13
627	B1MKW2	*MAB_4538c*	Putative acyl-CoA dehydrogenase FadE	0.33	0.056	12	21
628	B1MC33	*MAB_2725c*	DUF58 domain-containing protein	0.386	0.055	23	26
629	B1MDJ4	*ffh*	Signal recognition particle protein	0.427	0.054	37	33
630	B1MPD2	*MAB_2102*	Probable peptidase	0.354	0.053	20	29
631	B1MCE7	*MAB_2839*	Uncharacterized protein	0.323	0.052	2	8
632	B1MN13	*lepA*	Elongation factor 4	0.388	0.051	37	38
633	B1MH11	*MAB_0597*	Probable acyl-CoA dehydrogenase FadE	0.382	0.05	16	25
634	B1MEK1	*MAB_0165*	Putative acyltransferase	0.408	0.049	20	16
635	B1MNM9	*MAB_1847*	Neutral metalloproteinase	0.327	0.049	4	21
636	B1MD20	*MAB_3063*	LEH domain-containing protein	0.563	0.049	15	11
637	B1MHH7	*MAB_3984c*	Putative metal transporter ATPase	0.345	0.048	31	32
638	B1MJK3	*MAB_1069c*	Probable enoyl-CoA hydratase/isomerase	0.498	0.047	14	15
639	B1MF17	*MAB_3547*	Uncharacterized protein	0.332	0.047	5	21
640	B1ML29	*MAB_4605c*	Aldehyde dehydrogenase	0.361	0.047	44	25

Table S5. **Positively enriched proteins captured by VM055*p* probe from pre-incubated VM043-*M. abscessus* S culture through CC-ABPP by LC-ESI-MS/MS analysis, compared to DMSO-treated cells (i.e., non-specific conditions, NS)**

	Protein IDs	Gene names	Protein names	[VM043+VM055*p*] *vs.* NS -LOG(p-value)	Fold Change (Log2)	peptides counts all	nb Tryptic Peptides
641	B1MMY9	*MAB_1616*	Uncharacterized protein	0.35	0.047	11	15
642	B1MLZ9	*MAB_1490*	Uncharacterized protein	0.426	0.046	16	26
643	B1MJK5	*MAB_1071c*	Probable acetyl-/propionyl-CoA carboxylase alpha subunit AccA2	0.45	0.046	26	36
644	B1MBV3	*trpB*	Tryptophan synthase beta chain	0.447	0.045	28	23
645	B1MGY5	*cysS*	Cysteine--tRNA ligase	0.367	0.045	32	29
646	B1MJG6	*ku*	Non-homologous end joining protein Ku	0.345	0.043	20	19
647	B1MFE3	*MAB_0243*	Uncharacterized protein	0.352	0.042	10	24
648	B1MK14	*MAB_4452*	Putative transcriptional regulator, MerR family	0.348	0.042	2	16
649	B1MFX2	*MAB_0423c*	Conserved hypothetical membrane protein	0.33	0.042	9	11
650	B1MCT2	*MAB_2975c*	AFG1-like ATPase	0.361	0.041	16	20
651	B1MJY3	*MAB_4420*	Uncharacterized protein	0.345	0.041	15	8
652	B1MKW5	*MAB_4541*	Putative transcriptional regulator, TetR family	0.372	0.04	8	12
653	B1MBK5	*MAB_2545c*	Hypothetical nitrilase/cyanide hydratase and apolipoprotein N-acyltransferase	0.335	0.04	12	17
654	B1MLW2	*atpD*	ATP synthase subunit beta	0.352	0.04	47	31
655	B1MB24	*pyrG*	CTP synthase	0.383	0.04	37	34
656	B1MBW8	*MAB_2659*	Putative flavin-containing monoamine oxidase AofH	0.351	0.04	13	22
657	B1MD54	*MAB_3098*	Probable transmembrane carbonic anhydrase	0.376	0.039	15	26
658	B1ML32	*MAB_4608*	Beta-ketoacyl-[acyl-carrier-protein] synthase I	0.337	0.038	37	21
659	B1MJE2	*MAB_1008c*	Putative MCE family protein	0.325	0.038	12	22
660	B1MLA1	*MAB_4678*	Putative_PNPOx domain-containing protein	0.351	0.038	5	11
661	B1MNZ2	*MAB_1961*	Cytochrome aa3 subunit 2	0.356	0.038	37	19
662	B1MKP9	*MAB_1253c*	D-ser_dehydrat domain-containing protein	0.368	0.038	8	16
663	B1MLG3	*MAB_1302*	ENDO3c domain-containing protein	0.363	0.034	11	11
664	B1MLP3	*MAB_1384*	Probable malate dehydrogenase	0.339	0.034	28	26
665	B1MHD5	*MAB_3942*	N-acetyltransferase domain-containing protein	0.327	0.033	10	7
666	B1MEY4	*MAB_3514c*	Possible transmembrane cation transporter	0.319	0.032	8	22
667	B1MEM8	*MAB_0192c*	Probable oxidoreductase	0.332	0.032	29	29
668	B1MPF8	*MAB_2128c*	Uncharacterized protein	0.359	0.032	12	11
669	B1MM90	*MAB_4807*	Bacteriophage protein	0.332	0.031	4	15
670	B1MIJ7	*MAB_4145*	Alpha,alpha-trehalose-phosphate synthase (ADP-forming)	0.344	0.031	30	31
671	B1MCZ3	*nrdR*	Transcriptional repressor NrdR	0.418	0.031	9	15
672	B1MJH9	*MAB_1045*	zf-CGNR domain-containing protein	0.387	0.03	8	17
673	B1MJS0	*MAB_4355*	Hypothetical fumarylacetoacetate (FAA) hydrolase family	0.338	0.029	7	18
674	B1MH53	*psd*	Phosphatidylserine decarboxylase proenzyme	0.34	0.029	17	17
675	B1MHA8	*gpsA*	Glycerol-3-phosphate dehydrogenase [NAD(P)+]	0.321	0.029	22	20
676	B1MDT9	*MAB_3332c*	Uncharacterized protein	0.351	0.028	9	11
677	B1MKY4	*MAB_4560*	Alcohol dehydrogenase	0.319	0.028	22	20
678	B1MDQ5	*gltX*	Glutamate--tRNA ligase	0.337	0.028	38	30
679	B1MN51	*MAB_1678c*	Putative ferric uptake regulator FurB	0.323	0.028	14	10
680	B1MCC8	*metK*	S-adenosylmethionine synthase	0.328	0.028	28	22

Table S5. **Positively enriched proteins captured by VM055*p* probe from pre-incubated VM043-*M. abscessus* S culture through CC-ABPP by LC-ESI-MS/MS analysis, compared to DMSO-treated cells (i.e., non-specific conditions, NS)**

	Protein IDs	Gene names	Protein names	[VM043+VM055*p*] *vs.* NS -LOG(p-value)	Fold Change (Log2)	peptides counts all	nb Tryptic Peptides
681	B1MK22	*MAB_4460*	Uncharacterized protein	0.322	0.025	2	15
682	B1MF57	*MAB_3588*	Putative acyl-CoA oxidase	0.325	0.025	28	41
683	B1MHY9	*MAB_0715c*	Putative HTH-type transcriptional regulator AraC	0.32	0.025	12	26
684	B1MLU4	*MAB_1435*	Homoserine dehydrogenase	0.48	0.024	23	26
685	B1MEN3	*MAB_0197*	Putative alkylhydroperoxidase AhpD core	0.325	0.023	5	11
686	B1MC48	*MAB_2740c*	Probable oxidoreductase	0.325	0.023	10	21
687	B1MD91	*nusA*	Transcription termination/antitermination protein NusA	0.359	0.022	28	31
688	B1MNY0	*gcvT*	Aminomethyltransferase	0.354	0.022	18	16
689	B1MAI0	*MAB_2170*	Uncharacterized protein	0.358	0.021	28	35
690	B1MFF8	*MAB_0258c*	Putative dihydropicolinate reductase	0.354	0.021	14	11
691	B1MJ80	*MAB_0944*	Citrate synthase	0.339	0.021	38	27
692	B1MDZ6	*MAB_3389c*	Cytochrome c oxidase subunit 1	0.313	0.021	8	13
693	B1MML7	*MAB_4934c*	Poly(A) polymerase PcnA	0.335	0.021	28	26
694	B1MBV6	*trpE*	Anthranilate synthase component 1	0.325	0.02	29	26
695	B1MKJ8	*MAB_1202c*	Uncharacterized protein	0.311	0.02	4	11
696	B1MDP5	*gpsA*	Glycerol-3-phosphate dehydrogenase [NAD(P)+]	0.339	0.02	19	20
697	B1MN35	*MAB_1662c*	Assimilatory sulfite reductase (ferredoxin)	0.338	0.02	48	37
698	B1MCI3	*MAB_2875*	Beta-lactamase	0.347	0.019	21	19
699	B1MKP8	*MAB_1252*	Uncharacterized protein	0.325	0.018	10	12
700	B1MGV9	*lysS*	Lysine--tRNA ligase	0.339	0.017	33	32
701	B1MIF7	*MAB_4105c*	Methyltransferase MtfD	0.328	0.017	14	18
702	B1MCC9	*coaBC*	Coenzyme A biosynthesis bifunctional protein CoaBC	0.366	0.016	19	24
703	B1MAV8	*coaE*	Dephospho-CoA kinase	0.32	0.016	26	24
704	B1MGG2	*MAB_3832c*	Uncharacterized protein	0.318	0.016	9	12
705	B1MCB8	*MAB_2810c*	Probable Fmu protein (SUN protein)	0.348	0.016	28	31
706	B1ML45	*MAB_4621c*	Putative acetyltransferase	0.322	0.015	3	8
707	B1MFW7	*nth*	Endonuclease III	0.331	0.014	16	20
708	B1MC13	*ileS*	Isoleucine--tRNA ligase	0.318	0.014	65	79
709	B1MIS8	*MAB_4226c*	Phosphate acetyltransferase	0.327	0.013	51	39
710	B1MB20	*nadK*	NAD kinase	0.334	0.012	14	17
711	B1MF83	*MAB_3614*	Uncharacterized protein	0.311	0.012	12	33
712	B1MFP8	*MAB_0349c*	Putative hydrolase, alpha/beta fold	0.319	0.012	10	15
713	B1MGU8	*ftsH*	ATP-dependent zinc metalloprotease FtsH	0.313	0.012	62	47
714	B1MH19	*MAB_0605c*	Putative CoA-transferase alpha subunit	0.32	0.012	18	17
715	B1MEW5	*MAB_3495*	MHB domain-containing protein	0.304	0.01	7	4
716	B1MK15	*MAB_4453c*	YCII domain-containing protein	0.305	0.01	1	5
717	B1MK34	*MAB_4472*	F5/8 type C domain-containing protein	0.313	0.01	16	58
718	B1MG13	*MAB_3684c*	Exodeoxyribonuclease III	0.316	0.009	14	21
719	B1MIU7	*MAB_4245c*	Phosphoribosylglycinamide formyltransferase 2	0.308	0.008	28	30
720	B1MHZ0	*MAB_0716c*	Putative transcriptional regulator, MarR	0.31	0.008	15	14

Table S5. Positively enriched proteins captured by VM055*p* probe from pre-incubated VM043-*M. abscessus* S culture through CC-ABPP by LC-ESI-MS/MS analysis, compared to DMSO-treated cells (i.e., non-specific conditions, NS)

				[VM043+VM055*p*] *vs.* NS			
	Protein IDs	Gene names	Protein names	-LOG(p-value)	Fold Change (Log2)	peptides counts all	nb Tryptic Peptides
721	B1MMK1	*MAB_4918c*	Pyruvate dehydrogenase E1 component alpha subunit	0.309	0.008	2	21
722	B1MGZ4	*MAB_0580*	Probable acyl-CoA dehydrogenase FadE	0.334	0.007	18	31
723	B1MIC4	*MAB_0852*	Possible conserved polyketide synthase associated protein PapA2	0.311	0.007	11	25
724	B1MMW7	*MAB_1594*	Probable ferredoxin oxidoreductase, alpha subunit	0.312	0.006	50	29
725	B1MDM4	*MAB_3267c*	Pyruvate carboxylase	0.307	0.006	73	68
726	B1MJD6	*MAB_1002*	Putative acetyl-CoA C-acyltransferase (Thiolase)	0.308	0.006	18	30
727	B1MAJ3	*pafA*	Pup--protein ligase	0.312	0.005	48	39
728	B1MH26	*MAB_0612c*	Probable acetyl-CoA acetyltransferase FadA	0.305	0.005	14	17
729	B1MHC1	*MAB_3928c*	Probable polyprenyl-diphosphate synthase GrcC1	0.313	0.004	17	20
730	B1MHX3	*purQ*	Phosphoribosylformylglycinamidine synthase subunit PurQ	0.315	0.004	19	13
731	B1MI93	*MAB_0821*	Probable 4-aminobutyrate aminotransferase (GabT)	0.313	0.004	31	22
732	B1MGW9	*MAB_0554*	Putative hydrolase, alpha/beta fold family	0.306	0.004	3	18
733	B1MLW5	*MAB_1456c*	Corrinoid adenosyltransferase	0.308	0.004	6	12
734	B1MCV0	*MAB_2993c*	Uncharacterized protein	0.309	0.004	15	31
735	B1MGE1	*rpsQ*	30S ribosomal protein S17	0.306	0.004	19	8
736	B1MEH9	*MAB_0143c*	Putative oxidoreductase EphD	0.306	0.004	4	18
737	B1MJQ1	*MAB_4336*	Probable acyl-CoA dehydrogenase FadE	0.307	0.003	27	39
738	B1MHD7	*MAB_3944*	Uncharacterized protein	0.302	0.003	4	12
739	B1MMP1	*MAB_1517c*	Probable O-methyltransferase Omt	0.304	0.003	5	17
740	B1MLC7	*MAB_4704c*	Probable membrane protein, MmpL	0.303	0.003	7	52
741	B1MGG0	*MAB_3830c*	Probable dehydrogenase (Glucose-methanol-choline oxidoreductase?)	0.306	0.003	12	28
742	B1MLY0	*MAB_1471c*	Probable ATP-dependent helicase DinG	0.304	0.002	14	39
743	B1MC45	*MAB_2737c*	Probable enoyl-CoA hydratase/isomerase	0.31	0.002	30	19
744	B1MHY1	*purL*	Phosphoribosylformylglycinamidine synthase subunit PurL	0.304	0.002	60	36
745	B1MGY6	*MAB_0572*	Uncharacterized tRNA/rRNA methyltransferase MAB_0572	0.301	0	20	25
746	B1MFE4	*MAB_0244*	Uncharacterized protein	0.301	0	22	15
747	B1MP18	*MAB_1987*	Phospho-2-dehydro-3-deoxyheptonate aldolase	0.301	0	37	25

Table S6. Differential analysis of the *M. abscessus* S labeled proteome of samples pre-incubated with VM043 inhibitor followed by VM055*p* probe labeling vs. VM055*p* probe-labeled samples only

	Protein IDs	Gene names	Protein names	[VM043+VM055*p*] *vs* . VM055*p* -LOG(p-value)	Fold Change (Log2)	peptides counts all	nb Tryptic Peptides
1	B1MF33	*MAB_3564c*	Hypothetical dipeptidyl aminopeptidase/ acylaminoacyl-peptidase related protein	3.543	2.606	10	27
2	B1MDI7	*MAB_3230c*	SnoaL-like domain-containing protein	1.098	1.712	3	6
3	B1MBL9	*MAB_2559c*	Peptidyl-prolyl cis-trans isomerase	1.897	1.431	6	7
4	B1MEW6	*MAB_3496*	Uncharacterized protein	3.839	1.03	2	6
5	B1MG83	*MAB_3754c*	ESAT-6-like protein	0.834	1.081	14	7
6	B1MBP3	*MAB_2583c*	Putative transcription regulator, AraC family	0.8	1.614	4	10
7	B1MJ97	*MAB_0961c*	Methyltransf_25 domain-containing protein	0.73	1.236	5	10
8	B1MM39	*MAB_4756c*	Monooxygenase, FAD-binding	0.52	1.056	14	30
9	B1ML47	*metE*	5-methyltetrahydropteroyltriglutamate--homocysteine methyltransferase	1.085	0.973	19	42
10	B1MBV2	*trpA*	Tryptophan synthase alpha chain	0.637	0.962	9	15
11	B1MEQ0	*MAB_0214c*	Uncharacterized protein	0.573	0.886	3	8
12	B1MDV5	*MAB_3348*	Uncharacterized protein	1.081	0.886	4	11
13	B1MBI8	*MAB_2528c*	Rho_N domain-containing protein	1.106	0.883	1	12
14	B1MDV1	*MAB_3344*	Probable glycosyl transferase	1.279	0.857	3	29
15	B1MNV7	*MAB_1925*	MHB domain-containing protein	3.278	0.841	8	8
16	B1MIW2	*pyrE*	Orotate phosphoribosyltransferase	0.799	0.803	4	13
17	B1MF26	*MAB_3556*	Putative hydrolase, alpha/beta fold	0.464	0.798	8	18
18	B1MBD4	*MAB_2474*	NLPC_P60 domain-containing protein	0.874	0.792	4	7
19	B1MF87	*purE*	N5-carboxyaminoimidazole ribonucleotide mutase	0.352	0.788	3	7
20	B1MKD7	*fabH*	3-oxoacyl-[acyl-carrier-protein] synthase 3	0.513	0.775	18	16
21	B1MF72	*MAB_3603c*	Probable phosphomannomutase	0.728	0.773	19	25
22	B1MCT9	*MAB_2982c*	Uncharacterized protein	0.226	0.767	2	27
23	B1ML07	*MAB_4583c*	Uncharacterized protein	2.492	0.766	9	9
24	B1MP79	*MAB_2048c*	Probable cytochrome P450	0.471	0.731	29	32
25	B1MB62	*gcvH*	Glycine cleavage system H protein	0.466	0.666	1	3
26	B1MGS4	*MAB_0509c*	Aminopeptidase N	0.624	0.634	16	23
27	B1ML70	*MAB_4646*	DUF2236 domain-containing protein	0.669	0.623	12	26
28	B1MDR0	*leuB*	3-isopropylmalate dehydrogenase	0.532	0.616	8	19
29	B1MM04	*MAB_1495*	Probable oxidoreductase	0.74	0.615	37	42
30	B1MMI2	*rpsF*	30S ribosomal protein S6	0.22	0.613	4	7
31	B1MFB2	*MAB_3644*	Uncharacterized protein	0.751	0.603	7	7
32	B1MAW1	*MAB_2301*	Putative membrane protein, mmpL	0.543	0.602	22	55
33	B1MAS5	*ectC*	L-ectoine synthase	0.452	0.598	4	5
34	B1MDY4	*MAB_3377*	Uncharacterized protein	0.921	0.593	3	12
35	B1MK98	*MAB_1101*	Uncharacterized protein	0.843	0.591	33	46
36	B1MBT6	*MAB_2627c*	Possible two-component response regulatory protein	0.541	0.565	11	12
37	B1MJC0	*MAB_0984c*	Putative transcriptional regulator, TetR family	1.218	0.565	13	14
38	B1MHW2	*MAB_0687*	Adenylosuccinate lyase	0.73	0.563	42	32
39	B1MCB6	*MAB_2808c*	Riboflavin biosynthesis protein RibD	0.637	0.557	11	23
40	B1MLR6	*MAB_1407c*	Probable oxidoreductase	0.591	0.557	21	22
41	B1MNR0	*acpP*	Acyl carrier protein	0.639	0.545	9	7
42	B1MEI4	*MAB_0148c*	PPE family protein	0.295	0.544	8	14
43	B1MF68	*MAB_3599c*	Putative amino acid permease	0.302	0.54	3	19

Table S6. Differential analysis of the *M. abscessus* S labeled proteome of samples pre-incubated with VM043 inhibitor followed by VM055*p* probe labeling vs. VM055*p* probe-labeled samples only

	Protein IDs	Gene names	Protein names	[VM043+VM055*p*] *vs* . VM055*p* -LOG(p-value)	Fold Change (Log2)	peptides counts all	nb Tryptic Peptides
44	B1MHA5	*MAB_3912*	UPF0234 protein MAB_3912	0.48	0.54	9	14
45	B1MID5	*MAB_4083c*	Heparin-binding hemagglutinin (Adhesin)	1.178	0.535	14	15
46	B1MGE2	*rpmC*	50S ribosomal protein L29	0.451	0.527	11	7
47	B1MJ23	*MAB_0885c*	Hypothetical lipoprotein lpqH	0.727	0.526	5	5
48	B1MPA8	*MAB_2078*	Probable cytochrome P450	0.5	0.509	13	39
49	B1MAT6	*MAB_2276c*	Putative regulatory protein	0.522	0.501	2	11
50	B1MCH5	*MAB_2867*	Uncharacterized protein	0.563	0.501	11	13
51	B1MIR5	*MAB_4213c*	Probable lipoprotein aminopeptidase LpqL	1.748	0.5	30	36
52	B1MFR1	*MAB_0362c*	N-acetylglucosamine-6-phosphate deacetylase NagA	0.615	0.498	8	13
53	B1MDX0	*MAB_3363c*	Electron transfer flavoprotein beta-subunit FixA	0.404	0.485	20	20
54	B1MHJ8	*proC*	Pyrroline-5-carboxylate reductase	0.503	0.484	10	15
55	B1MKT6	*tpx*	Thiol peroxidase	0.397	0.479	9	9
56	B1MHK9	*MAB_4016c*	Uncharacterized protein	0.626	0.467	1	12
57	B1MF07	*MAB_3537c*	Uncharacterized protein	0.407	0.462	2	2
58	B1MLS1	*MAB_1412*	Uncharacterized protein	0.146	0.46	1	1
59	B1MLD7	*MAB_4714c*	Probable fatty-acid-coa ligase FadD	0.88	0.452	66	74
60	B1MEY2	*MAB_3512*	Putative glutaredoxin-like protein	1.399	0.451	4	5
61	B1MC42	*MAB_2734c*	Uncharacterized protein	0.352	0.448	12	16
62	B1MME0	*MAB_4857*	DUF1942 domain-containing protein	1.208	0.448	8	9
63	B1ML03	*MAB_4579c*	Probable NAD(P) transhydrogenase, alpha1 subunit PntAA	0.702	0.442	27	21
64	B1MAY3	*rplT*	50S ribosomal protein L20	0.376	0.441	7	9
65	B1MD52	*dapB*	4-hydroxy-tetrahydrodipicolinate reductase	0.55	0.436	10	18
66	B1MMS0	*MAB_1547c*	Probable sulfatase	0.669	0.428	12	25
67	B1MCR9	*MAB_2962*	Probable fatty-acid-CoA ligase FadD	0.563	0.418	26	56
68	B1MLL1	*MAB_1350*	Uncharacterized protein	0.222	0.417	2	5
69	B1MFQ3	*MAB_0354c*	Oxidoreductase, 2-nitropropane dioxygenase family	0.885	0.417	7	14
70	B1MG98	*rplQ*	50S ribosomal protein L17	0.576	0.411	11	14
71	B1MHH6	*MAB_3983c*	Uncharacterized protein	1.777	0.409	18	15
72	B1MG99	*rpoA*	DNA-directed RNA polymerase subunit alpha	0.313	0.409	33	24
73	B1MAE1	*MAB_p08*	Alkylmercury lyase	0.266	0.403	8	9
74	B1MH65	*MAB_3872c*	Probable drug-transport integral membrane protein	0.496	0.401	2	12
75	B1MD63	*MAB_3107c*	Possible lipoprotein LppU	0.591	0.395	4	8
76	B1MLB3	*MAB_4690c*	Mycobactin synthetase protein B	0.281	0.394	32	146
77	B1MNP5	*MAB_1863*	Probable acyl-CoA dehydrogenase FadE	0.351	0.392	19	27
78	B1MAF5	*MAB_2145*	NADH-quinone oxidoreductase, L subunit NuoL	0.244	0.391	4	18
79	B1MP55	*MAB_2024*	Amidase family protein	1.074	0.389	22	39
80	B1MNS6	*MAB_1894c*	DUF2235 domain-containing protein	0.27	0.387	4	24
81	B1MHD8	*menB*	1,4-dihydroxy-2-naphthoyl-CoA synthase	0.218	0.386	18	19
82	B1MKL5	*MAB_1219*	Probable short-chain dehydrogenase/reductase	0.317	0.385	9	18
83	B1MES5	*MAB_3455c*	Putative acyl-CoA thiolase	0.237	0.384	8	24
84	B1MAZ9	*argD*	Acetylornithine aminotransferase	0.335	0.384	13	16
85	B1MNA2	*MAB_1718*	Peptidase U62, modulator of DNA gyrase	0.885	0.376	45	36
86	B1MCH9	*MAB_2871c*	Uncharacterized protein	0.61	0.375	1	7

Table S6. Differential analysis of the *M. abscessus* S labeled proteome of samples pre-incubated with VM043 inhibitor followed by VM055*p* probe labeling vs. VM055*p* probe-labeled samples only

	Protein IDs	Gene names	Protein names	[VM043+VM055*p*] *vs* . VM055*p*		peptides counts all	nb Tryptic Peptides
				-LOG(p-value)	Fold Change (Log2)		
87	B1ML25	*MAB_4601c*	Putative YrbE family protein	0.206	0.37	10	11
88	B1MNL4	*MAB_1832*	NLPC_P60 domain-containing protein	0.717	0.37	16	32
89	B1MMX9	*ndk*	Nucleoside diphosphate kinase	0.287	0.368	2	10
90	B1MNS7	*MAB_1895c*	Uncharacterized protein	0.356	0.358	3	18
91	B1MBQ5	*MAB_2595*	Putative pyridoxamine 5'-phosphate oxidase	0.708	0.356	5	12
92	B1MEX1	*MAB_3501*	Uncharacterized protein	0.222	0.353	11	20
93	B1MHQ4	*MAB_4061c*	Uncharacterized protein	1.102	0.353	10	15
94	B1MB16	*MAB_2356*	TPR_5 domain-containing protein	1.154	0.352	12	26
95	B1MCE2	*MAB_2834c*	Methyltranfer_dom domain-containing protein	0.399	0.35	8	16
96	B1MM21	*MAB_4738c*	Putative amidohydrolase	0.566	0.348	19	21
97	B1MN07	*rpsT*	30S ribosomal protein S20	0.377	0.347	4	7
98	B1ME48	*MAB_0009*	Uncharacterized protein	0.498	0.347	6	10
99	B1MLH9	*MAB_1318c*	Probable catechol-o-methyltransferase	0.744	0.342	7	19
100	B1MHE4	*MAB_3951*	Uncharacterized protein	0.406	0.339	2	10
101	B1MJV8	*MAB_4394*	AHS2 domain-containing protein	0.362	0.338	6	16
102	B1MCJ1	*ruvA*	Holliday junction ATP-dependent DNA helicase RuvA	0.225	0.337	7	15
103	B1MFZ8	*MAB_3669*	Uncharacterized protein	1.307	0.337	10	18
104	B1MH12	*MAB_0598*	Short-chain dehydrogenase/reductase	0.414	0.332	24	16
105	B1MF86	*MAB_3618c*	Probable acyl-CoA dehydrogenase FadE	0.67	0.33	39	26
106	B1MMF1	*MAB_4868c*	Uncharacterized protein	0.467	0.327	10	15
107	B1MH86	*rplK*	50S ribosomal protein L11	0.751	0.324	13	13
108	B1MHI5	*hemC*	Porphobilinogen deaminase	1.036	0.321	14	22
109	B1MF80	*MAB_3611c*	Putative sugar-phosphate nucleotidyl transferase	0.688	0.314	28	26
110	B1MBX7	*hisB*	Imidazoleglycerol-phosphate dehydratase	0.214	0.314	4	7
111	B1ME73	*MAB_0035c*	Probable penicillin-binding protein PbpA	0.486	0.307	22	29
112	B1MH24	*MAB_0610*	Uncharacterized protein	0.627	0.307	7	11
113	B1MGT3	*ppa*	Inorganic pyrophosphatase	0.285	0.302	16	9
114	B1MCG5	*MAB_2857c*	TGc domain-containing protein	0.584	0.302	8	19
115	B1ML11	*MAB_4587c*	Putative S-adenosyl-L-methionine-dependent methyltransferase MAB_4587c	0.307	0.301	10	21
116	B1MEP0	*MAB_0204c*	Bacterial proteasome activator	0.443	0.299	7	12
117	B1MP12	*MAB_1981*	Uncharacterized protein	0.302	0.299	6	10
118	B1MAK2	*MAB_2192*	Probable dipeptidase PepE	0.686	0.295	17	18
119	B1MIH9	*MAB_4127c*	Dihydrolipoyl dehydrogenase	0.25	0.293	31	30
120	B1MF70	*MAB_3601c*	Mannose-6-phosphate isomerase	0.63	0.287	17	23
121	B1MP80	*MAB_2049c*	Probable ferredoxin	0.25	0.286	2	2
122	B1MGA7	*MAB_3777*	Band 7 protein	0.209	0.282	15	36
123	B1MG28	*MAB_3699c*	Uncharacterized protein	1.013	0.281	18	16
124	B1MAY1	*MAB_2321*	Translation initiation factor IF-3	0.718	0.278	15	13
125	B1MJ93	*MAB_0957*	Uncharacterized protein	0.21	0.276	5	6
126	B1MKU3	*MAB_4519c*	Putative two-component system response regulator,LuxR family	0.277	0.275	3	17
127	B1MMS2	*MAB_1549c*	Putative RNA binding protein, contains S1 domain	0.665	0.274	38	55
128	B1MC96	*MAB_2788*	Probable gamma-glutamyltranspeptidase (GgtB)	0.291	0.273	17	29
129	B1MFF2	*MAB_0252*	Uncharacterized protein	0.631	0.27	15	41

Table S6. Differential analysis of the *M. abscessus* S labeled proteome of samples pre-incubated with VM043 inhibitor followed by VM055*p* probe labeling vs. VM055*p* probe-labeled samples only

	Protein IDs	Gene names	Protein names	[VM043+VM055*p*] *vs* . VM055*p* -LOG(p-value)	Fold Change (Log2)	peptides counts all	nb Tryptic Peptides
130	B1MGD5	*rplE*	50S ribosomal protein L5	0.45	0.266	19	14
131	B1MB81	*MAB_2421c*	PknH_C domain-containing protein	1.683	0.266	17	20
132	B1MAH2	*mpa*	Proteasome-associated ATPase	2.107	0.266	38	48
133	B1MK84	*MAB_1086*	UTP--glucose-1-phosphate uridylyltransferase	0.377	0.265	15	20
134	B1MKH0	*MAB_1174c*	Probable acyl-CoA synthase FadD	0.17	0.263	15	32
135	B1MF12	*MAB_3542c*	zf-HC2 domain-containing protein	0.351	0.263	2	7
136	B1MCX5	*MAB_3018*	Putative transcriptional regulator, GntR family	0.645	0.262	5	18
137	B1MD03	*MAB_3046*	Uncharacterized protein	0.517	0.261	45	36
138	B1ME45	*gyrB*	DNA gyrase subunit B	1.307	0.261	61	43
139	B1MCD5	*carB*	Carbamoyl-phosphate synthase large chain	0.371	0.258	84	62
140	B1ML14	*MAB_4590*	Xanthosine permease	0.274	0.258	1	13
141	B1MLK7	*MAB_1346*	Glyco_trans_2-like domain-containing protein	0.664	0.257	7	11
142	B1MBP5	*MAB_2585*	L-ectoine synthase	0.227	0.255	4	6
143	B1MIF9	*MAB_4107c*	Glycosyltransferase GtfA	0.501	0.255	18	19
144	B1MNW5	*MAB_1933c*	Glutamine synthetase	0.252	0.253	21	26
145	B1MCM3	*MAB_2916*	Uncharacterized protein	0.123	0.252	14	32
146	B1MNN1	*MAB_1849*	Neutral metalloproteinase	0.141	0.246	6	23
147	B1MH69	*rplL*	50S ribosomal protein L7/L12	0.332	0.245	11	7
148	B1MCW6	*sigA*	RNA polymerase sigma factor SigA	1.107	0.244	32	26
149	B1MAW8	*uvrB*	UvrABC system protein B	0.827	0.243	40	51
150	B1MES0	*MAB_3450c*	Probable phosphoglucomutase PgmA	0.522	0.242	27	35
151	B1MC57	*MAB_2749c*	Putative FeS assembly protein SufB	2.125	0.242	44	33
152	B1MF62	*MAB_3593*	Possible Mg2+ transport P-type ATPase C MgtC	0.431	0.241	10	14
153	B1MMV3	*tig*	Trigger factor	0.274	0.24	21	33
154	B1MAL3	*MAB_2203*	Putative_PNPOx domain-containing protein	1.109	0.238	5	10
155	B1MGQ2	*MAB_0487*	Probable cold shock protein A (CspA)	0.095	0.237	3	6
156	B1MH40	*MAB_0626*	4-hydroxy-2-oxovalerate aldolase 1	0.165	0.235	12	20
157	B1MM70	*MAB_4787c*	Hypothetical regulatory protein, TetR family	0.445	0.234	9	10
158	B1MLG1	*MAB_1300c*	Uncharacterized protein	0.239	0.234	2	20
159	B1MDE3	*frr*	Ribosome-recycling factor	0.214	0.233	9	14
160	B1MGZ7	*MAB_0583c*	Putative oxidoreductase	0.136	0.231	10	21
161	B1MBX5	*priA*	Phosphoribosyl isomerase A	0.625	0.23	12	16
162	B1MM07	*MAB_1498c*	Putative GntR-family transcriptional regulator	0.555	0.23	6	21
163	B1MPG8	*MAB_2138*	NADH-quinone oxidoreductase, E subunit NuoE	0.149	0.229	5	14
164	B1MEI7	*MAB_0151c*	Septum_form domain-containing protein	0.721	0.226	16	21
165	B1MFL7	*MAB_0318c*	MurNAc-LAA domain-containing protein	0.259	0.221	12	12
166	B1MGR8	*MAB_0503*	Probable UDP-glucose 4-epimerase GalE1	0.777	0.219	24	22
167	B1MN94	*MAB_4952c*	R3H domain-containing protein	0.129	0.217	4	12
168	B1MFQ0	*MAB_0351*	Catalase	0.206	0.217	8	31
169	B1MI15	*MAB_0741c*	DUF4395 domain-containing protein	0.087	0.215	4	5
170	B1MG60	*groL*	60 kDa chaperonin	0.283	0.212	35	36
171	B1MMR0	*MAB_1537c*	Putative short chain dehydrogenase/reductase	0.286	0.21	23	19
172	B1MPG2	*hisG*	ATP phosphoribosyltransferase	0.323	0.209	21	22

Table S6. Differential analysis of the *M. abscessus* S labeled proteome of samples pre-incubated with VM043 inhibitor followed by VM055*p* probe labeling vs. VM055*p* probe-labeled samples only

	Protein IDs	Gene names	Protein names	[VM043+VM055*p*] *vs* . VM055*p* -LOG(p-value)	Fold Change (Log2)	peptides counts all	nb Tryptic Peptides
173	B1MDJ1	*MAB_3234*	Probable D-alanyl-D-alanine carboxypeptidase DacB	0.311	0.208	14	16
174	B1MLQ2	*MAB_1393c*	2-hydroxy-3-oxoadipate synthase	0.287	0.207	103	73
175	B1MGG7	*MAB_3838c*	Putative ferredoxin reductase	0.39	0.206	22	23
176	B1MDW8	*MAB_3361*	Putative transcriptional regulator, LuxR family	0.24	0.205	11	57
177	B1MGI0	*rpsL*	30S ribosomal protein S12	0.998	0.203	6	9
178	B1MNV2	*MAB_1920*	Glutamine synthetase	0.291	0.201	21	23
179	B1MAV6	*rpsA*	30S ribosomal protein S1	0.182	0.201	31	37
180	B1MIF2	*MAB_4100c*	MbtH-like protein	0.103	0.2	3	5
181	B1MFU5	*MAB_0396c*	Uncharacterized protein	0.53	0.199	8	11
182	B1MIX4	*grpE*	Protein GrpE	0.172	0.199	17	13
183	B1MEA0	*MAB_0064c*	Probable lipase LipE	0.373	0.198	26	26
184	B1MP15	*MAB_1984*	Probable 1-acylglycerol-3-phosphate O-acyltransferase	0.279	0.194	19	18
185	B1MKH5	*MAB_1179c*	Uncharacterized protein	0.456	0.192	5	9
186	B1MKN1	*MAB_1235*	Uncharacterized protein	0.319	0.191	26	29
187	B1MLV3	*MAB_1444*	YrdC-like domain-containing protein	0.375	0.19	7	12
188	B1MK05	*MAB_4442c*	Probable acetyl-CoA acyltransferase	0.362	0.187	31	27
189	B1MKC1	*MAB_1125c*	Hypothetical acetyltransferase, GNAT family	0.559	0.186	13	19
190	B1MKX5	*MAB_4551c*	Possible lysophospholipase	0.172	0.183	5	19
191	B1MDF2	*rpsB*	30S ribosomal protein S2	0.386	0.182	27	17
192	B1MJ64	*serC*	Phosphoserine aminotransferase	0.125	0.181	19	20
193	B1MD24	*MAB_3067c*	Uncharacterized protein	0.432	0.179	37	19
194	B1MIA7	*MAB_0835c*	Putative transcriptional regulator, TetR family	0.165	0.178	6	14
195	B1MIX3	*dnaJ*	Chaperone protein DnaJ	0.424	0.178	43	27
196	B1MAM1	*MAB_2211c*	Putative membrane protein, MmpS	0.182	0.177	3	6
197	B1MLI6	*MAB_1325c*	Probable NADPH dependent 2,4-dienoyl-CoA reductase FadH	0.453	0.177	52	46
198	B1MEU1	*MAB_3471*	Succinate-semialdehyde dehydrogenase	0.192	0.177	12	26
199	B1MEL3	*MAB_0177*	Antigen 85-A/B/C	0.514	0.175	13	13
200	B1MFR6	*MAB_0367c*	SnoaL-like domain-containing protein	0.311	0.174	6	10
201	B1MCB2	*MAB_2804c*	Putative ABC-type transporter, periplasmic component	0.107	0.171	10	19
202	B1MPB5	*MAB_2085*	Probable acyl CoA dehydrogenase	1.077	0.17	19	25
203	B1MHJ1	*MAB_3998*	Uncharacterized protein	1.282	0.17	34	33
204	B1MDH4	*MAB_3219*	Uncharacterized protein	0.178	0.169	8	7
205	B1MGV4	*MAB_0539*	Conserved hypothetical transmembrane protein	0.249	0.167	14	19
206	B1MM63	*MAB_4780*	MaoC-like dehydratase	0.314	0.165	24	15
207	B1MKL2	*MAB_1216c*	Probable cytochrome P450	0.176	0.164	6	32
208	B1MAI1	*pup*	Prokaryotic ubiquitin-like protein Pup	0.069	0.161	6	4
209	B1MG16	*MAB_3687*	Probable o-acetylhomoserine sulfhydrylase MetC (Homocysteine synthase)	0.142	0.16	10	20
210	B1MNX3	*lipA*	Lipoyl synthase	1.012	0.158	19	19
211	B1MDG5	*MAB_3210c*	Uncharacterized protein	0.134	0.158	4	14
212	B1MHH4	*MAB_3981c*	Probable zinc metalloprotease	0.235	0.157	33	42
213	B1MM06	*tetR*	DNA-binding transcriptional repressor TetR	0.149	0.157	4	14
214	B1MLB4	*MAB_4691c*	Mycobactin synthetase protein B	0.121	0.157	20	390
215	B1MLV1	*prfA*	Peptide chain release factor 1	0.313	0.156	20	24

Table S6. Differential analysis of the *M. abscessus* S labeled proteome of samples pre-incubated with VM043 inhibitor followed by VM055*p* probe labeling vs. VM055*p* probe-labeled samples only

	Protein IDs	Gene names	Protein names	[VM043+VM055*p*] *vs* . VM055*p* -LOG(p-value)	Fold Change (Log2)	peptides counts all	nb Tryptic Peptides
216	B1MEE4	*MAB_0108c*	Uncharacterized protein	0.251	0.156	12	9
217	B1MLU1	*MAB_1432*	Uncharacterized protein	0.101	0.156	5	10
218	B1MCZ0	*MAB_3033*	Uncharacterized protein	0.133	0.155	6	20
219	B1MC33	*MAB_2725c*	DUF58 domain-containing protein	0.297	0.154	23	26
220	B1MKQ3	*ispH*	4-hydroxy-3-methylbut-2-enyl diphosphate reductase	0.857	0.153	17	20
221	B1MFU8	*recB*	RecBCD enzyme subunit RecB	0.227	0.152	31	65
222	B1MC53	*MAB_2745c*	Possible SUF system FeS assembly protein	0.357	0.151	4	9
223	B1MN84	*MAB_1711c*	DUF5642 domain-containing protein	0.209	0.151	14	16
224	B1MGW1	*MAB_0546*	Probable ATP-dependent Clp protease ATP-binding subunit	0.17	0.15	69	57
225	B1MKD8	*pth*	Peptidyl-tRNA hydrolase	0.255	0.148	11	13
226	B1MKY5	*MAB_4561*	Uncharacterized protein	0.193	0.147	1	17
227	B1MF13	*MAB_3543c*	RNA polymerase sigma factor	0.182	0.147	11	17
228	B1MFP2	*MAB_0343*	Aspartokinase	0.41	0.143	35	29
229	B1MH07	*MAB_0593c*	Probable acyl-CoA dehydrogenase FadE	0.5	0.143	11	15
230	B1MAE2	*MAB_p09*	Probable FAD-dependent pyridine nucleotide-disulphide oxidoreductase	0.275	0.143	21	30
231	B1MI44	*MAB_0770*	tRNA_edit domain-containing protein	0.106	0.142	3	10
232	B1MLR3	*MAB_1404*	Probable acyltransferase	0.308	0.141	5	19
233	B1MCG9	*MAB_2861*	Uncharacterized protein	0.18	0.139	3	14
234	B1MBF6	*MAB_2496*	Probable acyl-CoA dehydrogenase	0.09	0.139	7	22
235	B1MIZ5	*MAB_4293*	Putative Fe-S oxidoreductase	0.263	0.139	70	49
236	B1ME46	*MAB_0007*	Uncharacterized protein	0.339	0.138	21	31
237	B1MLN7	*corA*	Magnesium transport protein CorA	0.131	0.138	27	21
238	B1MKC5	*MAB_1129*	Probable deoxyribonuclease TatD	0.106	0.138	20	22
239	B1MDP7	*ppk*	Polyphosphate kinase	0.463	0.137	42	46
240	B1MM01	*MAB_1492*	Uncharacterized protein	0.166	0.135	63	74
241	B1MDQ2	*MAB_3295*	Putative transcriptional regulator, IclR family	0.317	0.134	11	18
242	B1MKW1	*MAB_4537c*	Uncharacterized protein	0.238	0.134	21	15
243	B1MET9	*MAB_3469*	MutT/NUDIX family protein	0.35	0.133	4	8
244	B1MD89	*MAB_3133c*	Flavohemoglobin	0.105	0.132	10	23
245	B1MIQ0	*MAB_4198*	Uncharacterized protein	0.852	0.131	10	14
246	B1MHZ6	*MAB_0722*	Putative oligopeptide ABC transporter,ATP-binding protein	0.162	0.13	11	35
247	B1MPA9	*MAB_2079*	TGc domain-containing protein	0.213	0.129	4	18
248	B1MIJ2	*MAB_4140*	Uncharacterized protein	0.207	0.127	18	12
249	B1MNZ2	*MAB_1961*	Cytochrome aa3 subunit 2	0.222	0.126	37	19
250	B1MGG8	*MAB_3839c*	Putative transcriptional regulator, AsnC family	0.175	0.125	6	11
251	B1MN13	*lepA*	Elongation factor 4	0.367	0.125	37	38
252	B1MAK8	*MAB_2198c*	Probable cobalamin biosynthesis protein CobI	0.134	0.124	14	32
253	B1MNT5	*MAB_1903*	GTP cyclohydrolase 1 type 2 homolog	0.117	0.124	14	16
254	B1MGY5	*cysS*	Cysteine--tRNA ligase	0.177	0.124	32	29
255	B1MK17	*MAB_4455c*	Probable acyl-CoA synthetase FadD	0.338	0.123	4	32
256	B1MGE6	*rpsS*	30S ribosomal protein S19	0.154	0.123	7	5
257	B1MDX4	*MAB_3367*	Putative fatty-acid-CoA ligase	0.231	0.122	49	67
258	B1MIW7	*clpB*	Chaperone protein ClpB	0.091	0.121	77	60

Table S6. Differential analysis of the *M. abscessus* S labeled proteome of samples pre-incubated with VM043 inhibitor followed by VM055*p* probe labeling vs. VM055*p* probe-labeled samples only

	Protein IDs	Gene names	Protein names	[VM043+VM055*p*] *vs* . VM055*p*		peptides counts all	nb Tryptic Peptides
				-LOG(p-value)	Fold Change (Log2)		
259	B1MLG3	*MAB_1302*	ENDO3c domain-containing protein	0.212	0.12	11	11
260	B1MGI6	*MAB_3857c*	Probable enoyl-coa hydratase/isomerase	0.322	0.117	5	14
261	B1MAX6	*MAB_2316*	Probable acid-CoA ligase	0.062	0.117	10	22
262	B1MFL6	*MAB_0317*	Uncharacterized protein	0.17	0.116	9	9
263	B1MKJ2	*MAB_1196*	Proline-rich antigen (36 kDa antigen)	0.183	0.116	9	6
264	B1MFX2	*MAB_0423c*	Conserved hypothetical membrane protein	0.148	0.115	9	11
265	B1MKS3	*MAB_1277*	PMT_2 domain-containing protein	0.086	0.114	4	25
266	B1MMV9	*MAB_1586c*	Uncharacterized protein	0.113	0.114	5	6
267	B1MJH9	*MAB_1045*	zf-CGNR domain-containing protein	0.215	0.113	8	17
268	B1MNE2	*MAB_1758*	Uncharacterized protein	0.121	0.112	1	3
269	B1MD91	*nusA*	Transcription termination/antitermination protein NusA	0.463	0.11	28	31
270	B1MJB5	*MAB_0979*	Putative HTH-type transcriptional regulator MarR	0.093	0.11	10	13
271	B1MGV9	*lysS*	Lysine--tRNA ligase	0.245	0.109	33	32
272	B1MCE5	*efp*	Elongation factor P	0.078	0.107	4	10
273	B1MD90	*MAB_3134c*	Uncharacterized protein	0.428	0.107	7	9
274	B1MHH0	*MAB_3977c*	Uncharacterized protein	0.468	0.105	7	17
275	B1MH39	*MAB_0625*	Acetaldehyde dehydrogenase 1	0.132	0.105	11	16
276	B1MKG9	*MAB_1173c*	Uncharacterized protein	0.107	0.104	6	15
277	B1MG68	*MAB_3739c*	Alanine racemase	0.314	0.103	9	18
278	B1MME7	*MAB_4864*	Putative arsenate reductase	0.088	0.102	3	10
279	B1MFE3	*MAB_0243*	Uncharacterized protein	0.115	0.102	10	24
280	B1MCT8	*MAB_2981c*	Putative liporotein LppU	0.111	0.101	4	9
281	B1MGU8	*ftsH*	ATP-dependent zinc metalloprotease FtsH	0.124	0.101	62	47
282	B1MKG0	*MAB_1164*	Putative conserved lipoprotein LpqU	0.085	0.101	7	16
283	B1MHX3	*purQ*	Phosphoribosylformylglycinamidine synthase subunit PurQ	0.28	0.099	19	13
284	B1MCT6	*MAB_2979*	Peptide-methionine (R)-S-oxide reductase	0.053	0.098	12	9
285	B1MLP3	*MAB_1384*	Probable malate dehydrogenase	0.103	0.096	28	26
286	B1MG80	*rpsI*	30S ribosomal protein S9	0.13	0.096	8	12
287	B1MIE8	*MAB_4096c*	Uncharacterized protein	0.251	0.096	15	22
288	B1MH92	*rpmG2*	50S ribosomal protein L33 2	0.138	0.095	14	5
289	B1MCN1	*MAB_2924c*	Uncharacterized protein	0.143	0.095	5	22
290	B1MGG2	*MAB_3832c*	Uncharacterized protein	0.102	0.09	9	12
291	B1MEA5	*MAB_0069*	Major facilitator family transporter	0.091	0.09	6	12
292	B1MK27	*MAB_4465*	Tox-REase-7 domain-containing protein	0.223	0.089	14	35
293	B1MHV7	*purD*	Phosphoribosylamine--glycine ligase	0.247	0.088	19	20
294	B1MJD6	*MAB_1002*	Putative acetyl-CoA C-acyltransferase (Thiolase)	0.095	0.087	18	30
295	B1MHE5	*MAB_3952*	Possible O-succinylbenzoic acid--CoA ligase MenE	0.171	0.086	13	18
296	B1MH26	*MAB_0612c*	Probable acetyl-CoA acetyltransferase FadA	0.07	0.085	14	17
297	B1MFY2	*MAB_3653*	Probable pyridoxine 5-phosphate oxidase	0.04	0.084	4	15
298	B1MC08	*MAB_2699c*	Pseudouridine synthase	0.229	0.084	15	22
299	B1MJ09	*moaA*	GTP 3',8-cyclase	0.143	0.084	19	26
300	B1MKN7	*MAB_1241c*	CsbD domain-containing protein	0.086	0.084	6	6
301	B1MGU0	*MAB_0525c*	Probable conserved lipoprotein LpqG	0.163	0.083	5	15

Table S6. Differential analysis of the *M. abscessus* S labeled proteome of samples pre-incubated with VM043 inhibitor followed by VM055*p* probe labeling vs. VM055*p* probe-labeled samples only

				[VM043+VM055*p*] *vs* . VM055*p*			
	Protein IDs	Gene names	Protein names	-LOG(p-value)	Fold Change (Log2)	peptides counts all	nb Tryptic Peptides
302	B1MGW6	*MAB_0551*	Uncharacterized protein	0.137	0.08	3	12
303	B1MCU9	*MAB_2992c*	TRAM domain-containing protein	0.129	0.08	17	25
304	B1MB17	*MAB_2357*	HAD-superfamily hydrolase	0.083	0.079	13	14
305	B1MGF1	*rpsJ*	30S ribosomal protein S10	0.126	0.078	17	9
306	B1MMP5	*MAB_1521*	HisKA_3 domain-containing protein	0.232	0.077	3	13
307	B1MAV3	*MAB_2293*	Putative transcriptional regulator, MarR family	0.075	0.076	6	11
308	B1MBU3	*MAB_2634*	DUF4190 domain-containing protein	0.061	0.076	5	6
309	B1MKR2	*ychF*	Ribosome-binding ATPase YchF	0.203	0.076	24	25
310	B1MFB7	*MAB_3649*	Probable aldehyde dehydrogenase	0.158	0.075	26	31
311	B1MLF1	*MAB_4728c*	DNA-(apurinic or apyrimidinic site) lyase	0.07	0.073	6	21
312	B1ME58	*gyrA*	DNA gyrase subunit A	0.159	0.072	78	57
313	B1MM34	*MAB_4751*	PNPLA domain-containing protein	0.061	0.072	16	19
314	B1MKW2	*MAB_4538c*	Putative acyl-CoA dehydrogenase FadE	0.039	0.071	12	21
315	B1MDP5	*gpsA*	Glycerol-3-phosphate dehydrogenase [NAD(P)+]	0.145	0.071	19	20
316	B1MMV5	*clpP*	ATP-dependent Clp protease proteolytic subunit	0.172	0.071	9	14
317	B1MCK3	*MAB_2895c*	Putative acyltransferase	0.095	0.07	14	21
318	B1MLT6	*MAB_1427c*	Putative cytochrome P450	0.089	0.07	18	31
319	B1MMK7	*MAB_4924*	Uncharacterized protein	0.116	0.07	4	10
320	B1MBH3	*MAB_2513c*	Anti-sigma factor RsbW	0.15	0.07	13	9
321	B1MDN6	*MAB_3279c*	Putative phosphatase/kinase	0.152	0.069	23	28
322	B1MDB1	*cobB*	Hydrogenobyrinate a,c-diamide synthase	0.243	0.069	15	29
323	B1MC63	*MAB_2755c*	Uncharacterized protein	0.179	0.069	17	22
324	B1ME77	*MAB_0039c*	FHA domain-containing protein	0.05	0.068	10	21
325	B1MMS9	*MAB_1556*	Uncharacterized protein	0.105	0.068	4	9
326	B1MGD0	*MAB_3800c*	Glutamate dehydrogenase	0.071	0.067	33	34
327	B1MC25	*MAB_2717c*	Uncharacterized protein	0.046	0.067	21	26
328	B1MJB7	*MAB_0981c*	Possible enoyl-CoA hydratase/isomerase	0.09	0.067	14	14
329	B1MDZ5	*MAB_3388c*	O-phosphoserine phosphohydrolase	0.12	0.066	12	20
330	B1MHI8	*MAB_3995*	Uncharacterized protein	0.145	0.066	3	16
331	B1MGC6	*rplR*	50S ribosomal protein L18	0.057	0.066	7	11
332	B1MAI0	*MAB_2170*	Uncharacterized protein	0.142	0.065	28	35
333	B1MEF1	*MAB_0115c*	Uncharacterized protein	0.078	0.065	20	12
334	B1MPF3	*MAB_2123*	Mycobactin synthetase protein B	0.073	0.065	49	87
335	B1MHI4	*MAB_3991c*	Possible Uroporphyrin-III C-methyltransferase	0.224	0.064	36	31
336	B1MJA6	*MAB_0970c*	Probable drug resistance transporter	0.13	0.063	15	26
337	B1MIF7	*MAB_4105c*	Methyltransferase MtfD	0.091	0.063	14	18
338	B1MLY5	*MAB_1476*	DUF2017 domain-containing protein	0.045	0.063	5	9
339	B1MDQ1	*leuC*	3-isopropylmalate dehydratase large subunit	0.122	0.063	50	28
340	B1MLU4	*MAB_1435*	Homoserine dehydrogenase	0.446	0.062	23	26
341	B1MC29	*cpfC*	Coproporphyrin III ferrochelatase	0.093	0.062	19	19
342	B1MC56	*MAB_2748c*	Uncharacterized protein	0.158	0.062	22	26
343	B1MF81	*MAB_3612c*	Putative dTDP-rhamnosyltransferase	0.068	0.061	11	18
344	B1MCZ8	*MAB_3041*	Putative biotin sulfoxide reductase BisC	0.088	0.06	31	40

Table S6. Differential analysis of the *M. abscessus* S labeled proteome of samples pre-incubated with VM043 inhibitor followed by VM055*p* probe labeling vs. VM055*p* probe-labeled samples only

	Protein IDs	Gene names	Protein names	[VM043+VM055*p*] *vs* . VM055*p* -LOG(p-value)	Fold Change (Log2)	peptides counts all	nb Tryptic Peptides
345	B1MJA0	*MAB_0964*	Uncharacterized protein	0.084	0.06	13	10
346	B1MMI1	*MAB_4898c*	Single-stranded DNA-binding protein	0.057	0.059	11	12
347	B1MLL3	*glgC*	Glucose-1-phosphate adenylyltransferase	0.177	0.058	16	25
348	B1MP03	*MAB_1972c*	Methyltransf_11 domain-containing protein	0.145	0.057	13	15
349	B1MFB9	*MAB_0219*	TED domain-containing protein	0.034	0.057	7	19
350	B1MDW3	*mnmA*	tRNA-specific 2-thiouridylase MnmA	0.074	0.055	12	22
351	B1MH19	*MAB_0605c*	Putative CoA-transferase alpha subunit	0.095	0.055	18	17
352	B1MJX1	*MAB_4408c*	Alkyl hydroperoxide reductase C	0.098	0.055	21	13
353	B1MIG0	*MAB_4108c*	Methyltransferase MtfB	0.106	0.054	19	20
354	B1MJ66	*MAB_0930*	Putative ferredoxin/ferredoxin--NADP reductase	0.147	0.054	37	36
355	B1MEM1	*MAB_0185c*	Probable arabinosyltransferase B	0.117	0.053	39	54
356	B1MED3	*MAB_0097*	POLIIIAc domain-containing protein	0.11	0.053	24	24
357	B1MEQ7	*MAB_3437c*	Putative transcriptional regulator, TetR family	0.162	0.052	4	15
358	B1MHY1	*purL*	Phosphoribosylformylglycinamidine synthase subunit PurL	0.144	0.052	60	36
359	B1MCV4	*MAB_2997c*	RecG_wedge domain-containing protein	0.126	0.051	13	12
360	B1MAJ3	*pafA*	Pup--protein ligase	0.085	0.051	48	39
361	B1MJ80	*MAB_0944*	Citrate synthase	0.082	0.051	38	27
362	B1MMU2	*MAB_1569*	Uncharacterized protein	0.035	0.051	5	10
363	B1ME24	*MAB_3417c*	Probable NADPH-dependent FMN reductase	0.048	0.05	6	10
364	B1MJY2	*MAB_4419*	Tartrate dehydrogenase	0.09	0.05	10	12
365	B1MD31	*MAB_3074c*	Probable cell division protein FtsK	0.049	0.048	49	52
366	B1MFY8	*MAB_3659c*	Peptidase M20 domain-containing protein 2	0.039	0.046	10	14
367	B1MBZ9	*MAB_2690*	Glycogen operon protein GlgX homolog	0.062	0.045	15	39
368	B1MIX8	*MAB_4276c*	Probable conserved lipoprotein DsbF	0.047	0.044	8	12
369	B1MNU6	*MAB_1914c*	Probable adenylate cyclase	0.056	0.042	29	35
370	B1MMW7	*MAB_1594*	Probable ferredoxin oxidoreductase, alpha subunit	0.098	0.042	50	29
371	B1MJM7	*MAB_4312*	Putative TetR-family transcriptional regulator	0.038	0.041	2	13
372	B1MEH9	*MAB_0143c*	Putative oxidoreductase EphD	0.055	0.041	4	18
373	B1MDM6	*MAB_3269c*	Thioredoxin-like_fold domain-containing protein	0.032	0.04	25	22
374	B1MHW8	*MAB_0693*	Glutathione peroxidase	0.049	0.04	6	11
375	B1MIL6	*MAB_4164*	Possible enoyl-CoA hydratase	0.053	0.04	5	19
376	B1MAG3	*MAB_2153*	MOSC domain-containing protein	0.05	0.04	3	16
377	B1MFI3	*MAB_0283c*	Uncharacterized protein	0.049	0.04	7	13
378	B1MD54	*MAB_3098*	Probable transmembrane carbonic anhydrase	0.067	0.039	15	26
379	B1MCN5	*MAB_2928*	Guanine deaminase	0.192	0.038	15	26
380	B1MM47	*MAB_4764c*	Uncharacterized protein	0.035	0.038	10	23
381	B1MLQ7	*MAB_1398c*	Putative oxidoreductase	0.057	0.037	22	28
382	B1MCT2	*MAB_2975c*	AFG1-like ATPase	0.05	0.036	16	20
383	B1MF76	*MAB_3607*	LPPG:FO 2-phospho--lactate transferase (CofD)	0.061	0.036	28	26
384	B1MGZ9	*MAB_0585*	Possible 2,3-dihydroxybiphenyl 1,2-dioxygenase	0.08	0.035	20	21
385	B1MJ79	*MAB_0943*	DJ-1_PfpI domain-containing protein	0.028	0.035	7	12
386	B1MIT2	*fgd*	F420-dependent glucose-6-phosphate dehydrogenase	0.108	0.035	29	24
387	B1MJZ3	*MAB_4430c*	Putative oxidoreductase	0.009	0.034	3	5

Table S6. Differential analysis of the *M. abscessus* S labeled proteome of samples pre-incubated with VM043 inhibitor followed by VM055*p* probe labeling vs. VM055*p* probe-labeled samples only

	Protein IDs	Gene names	Protein names	[VM043+VM055*p*] *vs* . VM055*p*		peptides counts all	nb Tryptic Peptides
				-LOG(p-value)	Fold Change (Log2)		
388	B1MLM3	*MAB_1364*	Probable serine protease HtrA	0.085	0.033	21	32
389	B1MB72	*MAB_2412c*	Probable IMP dehydrogenase family protein	0.088	0.032	32	27
390	B1MK64	*pckG*	Phosphoenolpyruvate carboxykinase [GTP]	0.127	0.031	60	41
391	B1MDE4	*pyrH*	Uridylate kinase	0.083	0.031	15	16
392	B1MB80	*MAB_2420c*	PknH_C domain-containing protein	0.092	0.031	14	16
393	B1MKP9	*MAB_1253c*	D-ser_dehydrat domain-containing protein	0.048	0.03	8	16
394	B1MK13	*MAB_4451c*	Uncharacterized protein	0.037	0.029	1	17
395	B1MGQ5	*MAB_0490c*	Putative adenylate cyclase	0.026	0.029	15	32
396	B1MMW6	*MAB_1593c*	Probable formate dehydrogenase, A chain	0.072	0.028	62	55
397	B1MIG6	*MAB_4114*	Uncharacterized protein	0.036	0.027	8	4
398	B1MG47	*guaA*	GMP synthase [glutamine-hydrolyzing]	0.053	0.027	24	23
399	B1MKF9	*MAB_1163c*	Putative iron permease FTR1	0.069	0.024	2	10
400	B1MC16	*MAB_2708*	Uncharacterized protein	0.023	0.023	6	25
401	B1MD70	*MAB_3114*	Ntox37 domain-containing protein	0.07	0.023	17	22
402	B1MLJ7	*MAB_1336*	Probable succinyl-diaminopimelate desuccinylase	0.024	0.023	19	21
403	B1MCF1	*aroC*	Chorismate synthase	0.035	0.023	39	24
404	B1MIA2	*MAB_0830*	Probable NADH-dependent flavin oxidoreductase	0.04	0.022	11	18
405	B1MJB2	*MAB_0976*	Putative glyoxalase/bleomycin resistance protein	0.022	0.02	3	9
406	B1MI95	*MAB_0823*	Probable aldehyde dehydrogenase	0.026	0.019	19	27
407	B1MAX9	*lysX*	Lysylphosphatidylglycerol biosynthesis bifunctional protein LysX	0.037	0.019	44	67
408	B1MBK5	*MAB_2545c*	Hypothetical nitrilase/cyanide hydratase and apolipoprotein N-acyltransferase	0.015	0.018	12	17
409	B1MIM8	*MAB_4176c*	NAD_binding_9 domain-containing protein	0.026	0.018	2	25
410	B1MHE0	*MAB_3947*	Possible oxidoreductase	0.01	0.014	28	20
411	B1MN52	*MAB_1679c*	Probable ArsR-family transcriptional regulator	0.013	0.014	3	8
412	B1MNY1	*MAB_1950*	Branched-chain-amino-acid aminotransferase	0.02	0.013	28	18
413	B1MNQ7	*MAB_1875c*	Putative hydrolase (Alpha/beta fold)	0.025	0.013	5	14
414	B1MLZ9	*MAB_1490*	Uncharacterized protein	0.025	0.01	16	26
415	B1MK32	*MAB_4470c*	Uncharacterized protein	0.023	0.01	7	14
416	B1MB33	*MAB_2373*	Putative mannose-specific lectin	0.004	0.009	3	6
417	B1MML7	*MAB_4934c*	Poly(A) polymerase PcnA	0.01	0.008	28	26
418	B1MN16	*MAB_1643*	Putative glycosyl hydrolase	0.018	0.007	42	43
419	B1MHC1	*MAB_3928c*	Probable polyprenyl-diphosphate synthase GrcC1	0.017	0.006	17	20
420	B1MNZ7	*MAB_1966c*	Cytochrome bc1 complex cytochrome b subunit	0.011	0.006	15	25
421	B1MEH4	*MAB_0138*	Putative transcriptional regulator, TetR family	0.01	0.006	9	11
422	B1MG89	*MAB_3760*	Uncharacterized protein	0.017	0.006	23	29
423	B1MDU2	*pfkA*	ATP-dependent 6-phosphofructokinase	0.012	0.005	19	22
424	B1ML36	*MAB_4612*	NTP_transf_9 domain-containing protein	0.004	0.004	4	8
425	B1MEE2	*MAB_0106c*	Ala_racemase_N domain-containing protein	0.004	0.003	17	21
426	B1MHD4	*MAB_3941c*	Uncharacterized protein	0.005	0.002	21	18
427	B1MLW5	*MAB_1456c*	Corrinoid adenosyltransferase	0.004	0.002	6	12
428	B1MKW5	*MAB_4541*	Putative transcriptional regulator, TetR family	0.000	0.000	8	12
429	B1MI93	*MAB_0821*	Probable 4-aminobutyrate aminotransferase (GabT)	0.001	0.000	31	22
430	B1MAY2	*rpmI*	50S ribosomal protein L35	0.000	0.000	2	4

Table S6. Differential analysis of the *M. abscessus* S labeled proteome of samples pre-incubated with VM043 inhibitor followed by VM055*p* probe labeling vs. VM055*p* probe-labeled samples only

	Protein IDs	Gene names	Protein names	[VM043+VM055*p*] *vs* . VM055*p* -LOG(p-value)	Fold Change (Log2)	peptides counts all	nb Tryptic Peptides
1	B1MEN3	*MAB_0197*	Putative alkylhydroperoxidase AhpD core	0.001	-0.001	5	11
2	B1MGG9	*MAB_3840*	Uncharacterized protein	0.004	-0.003	2	17
3	B1MKH9	*MAB_1183*	Rhodanese domain-containing protein	0.007	-0.005	3	8
4	B1MMB5	*MAB_4832c*	Uncharacterized protein	0.008	-0.006	3	11
5	B1MAM3	*MAB_2213*	Putative thioesterase	0.005	-0.006	8	15
6	B1MK68	*trmB*	tRNA (guanine-N(7)-)-methyltransferase	0.021	-0.009	9	17
7	B1MEZ6	*MAB_3526c*	ZnMc domain-containing protein	0.012	-0.01	15	22
8	B1MLA1	*MAB_4678*	Putative_PNPOx domain-containing protein	0.015	-0.011	5	11
9	B1MAV8	*coaE*	Dephospho-CoA kinase	0.011	-0.011	26	24
10	B1MAJ7	*tatA*	Sec-independent protein translocase protein TatA	0.025	-0.016	2	4
11	B1MCZ3	*nrdR*	Transcriptional repressor NrdR	0.033	-0.018	9	15
12	B1MK18	*MAB_4456*	Putative cytochrome P450	0.014	-0.02	16	28
13	B1MKA9	*MAB_1113c*	Putative_PNPOx domain-containing protein	0.011	-0.02	6	9
14	B1MFF8	*MAB_0258c*	Putative dihydropicolinate reductase	0.049	-0.021	14	11
15	B1MJK5	*MAB_1071c*	Probable acetyl-/propionyl-CoA carboxylase alpha subunit AccA2	0.066	-0.022	26	36
16	B1MGD3	*MAB_3803c*	Carboxylic ester hydrolase	0.022	-0.023	13	28
17	B1MH11	*MAB_0597*	Probable acyl-CoA dehydrogenase FadE	0.038	-0.024	16	25
18	B1MDF9	*xerC*	Tyrosine recombinase XerC	0.028	-0.025	4	20
19	B1MJG6	*ku*	Non-homologous end joining protein Ku	0.036	-0.025	20	19
20	B1ML15	*MAB_4591*	Putative phosphotyrosine protein phosphatase	0.072	-0.025	14	19
21	B1MGD6	*rplX*	50S ribosomal protein L24	0.023	-0.025	6	6
22	B1MD79	*MAB_3123*	Putative acyl-CoA dehydrogenase	0.037	-0.026	13	20
23	B1MEY6	*MAB_3516c*	DNA helicase	0.034	-0.027	17	66
24	B1MEQ1	*MAB_0215*	Possible transcriptional regulator	0.016	-0.028	5	14
25	B1MEK0	*MAB_0164*	Probable short chain dehydrogenase/reductase	0.1	-0.032	11	9
26	B1MFI5	*MAB_0285*	Putative oxidoreductase	0.049	-0.033	7	20
27	B1MMB6	*MAB_4833c*	Uncharacterized protein	0.05	-0.035	10	12
28	B1MH77	*MAB_3884*	Possible flavoprotein	0.048	-0.035	21	32
29	B1MAK3	*MAB_2193c*	Putative_PNPOx domain-containing protein	0.037	-0.036	4	12
30	B1MME8	*MAB_4865*	Putative pyridine nucleotide-disulphide oxidoreductase	0.07	-0.036	18	20
31	B1MEY4	*MAB_3514c*	Possible transmembrane cation transporter	0.019	-0.037	8	22
32	B1MAD5	*MAB_p02*	Uncharacterized protein	0.021	-0.039	3	12
33	B1MK61	*MAB_4499*	Histidine kinase	0.061	-0.039	9	35
34	B1ME28	*MAB_3421*	Uncharacterized protein	0.062	-0.039	9	23
35	B1MFZ4	*MAB_3665*	Uncharacterized protein	0.063	-0.041	7	9
36	B1MIC4	*MAB_0852*	Possible conserved polyketide synthase associated protein PapA2	0.143	-0.044	11	25
37	B1MJS9	*MAB_4365c*	Putative dihydrodiol dehydrogenase	0.146	-0.044	4	18
38	B1MJ81	*MAB_0945*	Putative drug resistance transporter, EmrB/QacA family	0.075	-0.045	2	14
39	B1MCF8	*MAB_2850c*	Putative pre-16S rRNA nuclease	0.081	-0.045	7	14
40	B1ME98	*MAB_0062*	Pyridine nucleotide-disulphide oxidoreductase family	0.143	-0.046	17	29
41	B1MDE8	*MAB_3192c*	Putative glycosyl hydrolase (Beta-glucosidase)	0.033	-0.047	25	27
42	B1MHG4	*MAB_3971*	Uncharacterized protein	0.032	-0.048	3	4
43	B1MFX3	*MAB_0424*	Putative protease	0.046	-0.048	2	10

Table S6. **Differential analysis of the *M. abscessus* S labeled proteome of samples pre-incubated with VM043 inhibitor followed by VM055*p* probe labeling vs. VM055*p* probe-labeled samples only**

				[VM043+VM055*p*] *vs* . VM055*p*			
	**Protein IDs**	**Gene names**	**Protein names**	**-LOG(p-value)**	**Fold Change (Log2)**	**peptides counts all**	**nb Tryptic Peptides**
44	B1MN35	*MAB_1662c*	Assimilatory sulfite reductase (ferredoxin)	0.164	-0.049	48	37
45	B1MI24	*MAB_0750*	Putative oxidoreductase	0.045	-0.049	3	14
46	B1MNK9	*MAB_1827c*	Bacteriophage protein	0.08	-0.054	1	5
47	B1MHY9	*MAB_0715c*	Putative HTH-type transcriptional regulator AraC	0.045	-0.056	12	26
48	B1MIU6	*MAB_4244c*	Uncharacterized protein	0.039	-0.057	7	13
49	B1MIQ6	*MAB_4204*	Amine oxidase	0.031	-0.058	74	38
50	B1MMJ3	*MAB_4910c*	Putative aminoglycoside phosphotransferase	0.066	-0.058	4	13
51	B1MP63	*MAB_2032*	Probable 3-oxoacyl-[acyl-carrier protein] reductase	0.038	-0.058	4	12
52	B1MJG0	*MAB_1026c*	DUF1942 domain-containing protein	0.074	-0.064	3	9
53	B1MBV3	*trpB*	Tryptophan synthase beta chain	0.199	-0.07	28	23
54	B1MGE0	*MAB_3810*	Putative hydrolase, alpha/beta fold	0.092	-0.07	21	16
55	B1MP48	*MAB_2017*	Antigen 84	0.091	-0.071	29	23
56	B1MF02	*MAB_3532*	Uncharacterized protein	0.038	-0.071	14	27
57	B1MCU2	*MAB_2985c*	Coproporphyrinogen III oxidase	0.193	-0.075	19	26
58	B1MIU8	*MAB_4246*	Putative Na+/H+ antiporter	0.066	-0.076	16	26
59	B1MN41	*MAB_1668*	PhoH-like protein	0.129	-0.076	7	24
60	B1MK58	*MAB_4496c*	Luciferase-like monooxygenase	0.204	-0.076	13	12
61	B1MF57	*MAB_3588*	Putative acyl-CoA oxidase	0.113	-0.079	28	41
62	B1MHY5	*MAB_0711*	SCP_3 domain-containing protein	0.091	-0.079	4	9
63	B1MBW6	*MAB_2657c*	Uncharacterized protein	0.068	-0.08	16	29
64	B1MC45	*MAB_2737c*	Probable enoyl-CoA hydratase/isomerase	0.211	-0.081	30	19
65	B1MPD2	*MAB_2102*	Probable peptidase	0.112	-0.084	20	29
66	B1MGQ6	*MAB_0491*	Probable DNA polymerase III, delta' subunit	0.223	-0.088	26	31
67	B1MGF5	*MAB_3825*	Cytochrome P450	0.201	-0.093	8	25
68	B1MGZ4	*MAB_0580*	Probable acyl-CoA dehydrogenase FadE	0.586	-0.093	18	31
69	B1MIS1	*thiE*	Thiamine-phosphate synthase	0.207	-0.094	10	16
70	B1MAQ8	*MAB_2248*	Mycobactin synthetase protein B	0.076	-0.094	27	84
71	B1MGY2	*MAB_0568*	Putative CarD-like transcriptional regulator	0.086	-0.095	11	13
72	B1MIS4	*MAB_4222*	Uncharacterized protein	0.16	-0.096	15	25
73	B1MHS3	*MAB_0647*	Possible transcriptional regulatory protein TetR	0.141	-0.096	5	13
74	B1ML32	*MAB_4608*	Beta-ketoacyl-[acyl-carrier-protein] synthase I	0.13	-0.098	37	21
75	B1ML82	*MAB_4658*	Putative transcriptional regulator, LysR family	0.126	-0.099	8	20
76	B1MG04	*MAB_3675*	Succinate dehydrogenase flavoprotein subunit	0.501	-0.099	55	41
77	B1MKS6	*MAB_1280c*	Uncharacterized protein	0.129	-0.1	2	17
78	B1MBG3	*MAB_2503c*	Putative iron-sulfur binding oxidoreductase	0.087	-0.1	2	30
79	B1MHK7	*MAB_4014*	Uncharacterized protein	0.489	-0.101	22	16
80	B1MNZ5	*MAB_1964*	Uncharacterized protein	0.466	-0.102	8	11
81	B1MMP9	*MAB_1525c*	Putative transcriptional regulator, TetR family	0.209	-0.102	5	13
82	B1MKZ9	*MAB_4575*	Probable low temperature requirement protein A	0.459	-0.103	7	15
83	B1MN74	*MAB_1701*	Uncharacterized protein	0.079	-0.107	1	20
84	B1MBG1	*MAB_2501*	Uncharacterized protein	0.283	-0.108	6	8
85	B1MJ98	*MAB_0962*	Probable cation-transporting ATPase E	0.596	-0.11	19	39
86	B1ML45	*MAB_4621c*	Putative acetyltransferase	0.106	-0.111	3	8

Table S6. Differential analysis of the *M. abscessus* S labeled proteome of samples pre-incubated with VM043 inhibitor followed by VM055*p* probe labeling vs. VM055*p* probe-labeled samples only

				[VM043+VM055*p*] *vs* . VM055*p*			
	Protein IDs	Gene names	Protein names	-LOG(p-value)	Fold Change (Log2)	peptides counts all	nb Tryptic Peptides
87	B1MB89	*MAB_2429c*	Probable NADH dehydrogenase (NDH)	0.14	-0.112	28	26
88	B1MFM6	*MAB_0327*	Possible aminoglycoside phosphotransferase	0.122	-0.116	8	21
89	B1MGJ9	*MAB_0434c*	Uncharacterized protein	0.2	-0.117	12	27
90	B1MEK1	*MAB_0165*	Putative acyltransferase	0.22	-0.119	20	16
91	B1MDH2	*MAB_3217*	CsbD domain-containing protein	0.107	-0.121	7	11
92	B1MGE1	*rpsQ*	30S ribosomal protein S17	0.222	-0.121	19	8
93	B1MI80	*MAB_0807*	Uncharacterized protein	0.239	-0.121	3	21
94	B1MGA8	*MAB_3778*	Bac_luciferase domain-containing protein	0.189	-0.123	7	15
95	B1MCF0	*aroK*	Shikimate kinase	0.109	-0.127	2	16
96	B1MIS7	*ackA*	Acetate kinase	0.09	-0.128	19	21
97	B1MKL0	*MAB_1214c*	Probable cytochrome P450	0.119	-0.131	8	31
98	B1MLC7	*MAB_4704c*	Probable membrane protein, MmpL	0.267	-0.134	7	52
99	B1MJ72	*MAB_0936c*	PE-PPE domain-containing protein	0.096	-0.137	9	16
100	B1MFX0	*MAB_0421*	Possible membrane-associated serine protease	0.082	-0.137	12	20
101	B1ME23	*MAB_3416*	Probable membrane transport protein	0.212	-0.146	4	11
102	B1MKI1	*MAB_1185c*	Probable enoyl-CoA hydratase	0.521	-0.147	14	16
103	B1MFE4	*MAB_0244*	Uncharacterized protein	0.124	-0.149	22	15
104	B1MEB7	*MAB_0081*	Putative 3-ketosteroid 1-dehydrogenase or fumarate reductase/succinate dehydrogenase	0.143	-0.152	6	35
105	B1MKY4	*MAB_4560*	Alcohol dehydrogenase	0.11	-0.154	22	20
106	B1MDJ8	*ftsY*	Signal recognition particle receptor FtsY	0.262	-0.155	13	23
107	B1MFW7	*nth*	Endonuclease III	0.216	-0.157	16	20
108	B1MHU6	*MAB_0670*	Uncharacterized protein	0.188	-0.158	4	16
109	B1MC58	*MAB_2750c*	Probable transcriptional regulatory protein	0.347	-0.158	9	12
110	B1MMK8	*MAB_4925*	Uncharacterized protein	0.114	-0.159	2	4
111	B1MCE7	*MAB_2839*	Uncharacterized protein	0.088	-0.16	2	8
112	B1MB98	*MAB_2438*	Probable oxidoreductase	0.209	-0.165	6	71
113	B1MLS6	*MAB_1417*	Putative lipoprotein LprC	0.144	-0.166	8	13
114	B1MCL8	*MAB_2911c*	Putative dipeptidase	0.087	-0.166	10	21
115	B1ME26	*nadE*	NH(3)-dependent NAD(+) synthetase	0.418	-0.166	21	20
116	B1MM90	*MAB_4807*	Bacteriophage protein	0.213	-0.166	4	15
117	B1MG02	*MAB_3673*	Probable succinate dehydrogenase (Cytochrome b-556 subunit) SdhC	0.28	-0.17	6	8
118	B1MC70	*MAB_2762*	Putative OxpP cycle protein OpcA	0.586	-0.171	19	18
119	B1MKI4	*MAB_1188c*	Probable acyl-CoA dehydrogenase	0.367	-0.172	31	29
120	B1MCY6	*MAB_3029*	Iron-dependent repressor IdeR	0.363	-0.173	23	14
121	B1MIU7	*MAB_4245c*	Phosphoribosylglycinamide formyltransferase 2	0.187	-0.173	28	30
122	B1MMI6	*MAB_4903*	Uncharacterized protein	0.231	-0.174	3	7
123	B1MD17	*recA*	Protein RecA	0.347	-0.176	26	23
124	B1MIN9	*def*	Peptide deformylase	0.073	-0.187	4	8
125	B1MF35	*MAB_3566c*	Putative cyclase	0.322	-0.189	4	15
126	B1MGZ8	*MAB_0584*	Acyl-CoA_dh_2 domain-containing protein	0.298	-0.189	23	20
127	B1MJS0	*MAB_4355*	Hypothetical fumarylacetoacetate (FAA) hydrolase family	0.335	-0.19	7	18
128	B1MDX9	*MAB_3372*	Putative transcriptional regulator, TetR family	0.137	-0.192	1	12
129	B1ML69	*MAB_4645*	Uncharacterized protein	0.197	-0.194	1	4

Table S6. Differential analysis of the *M. abscessus* S labeled proteome of samples pre-incubated with VM043 inhibitor followed by VM055*p* probe labeling vs. VM055*p* probe-labeled samples only

				[VM043+VM055*p*] *vs* . VM055*p*			
	**Protein IDs**	**Gene names**	**Protein names**	**-LOG(p-value)**	**Fold Change (Log2)**	**peptides counts all**	**nb Tryptic Peptides**
130	B1MJ04	*MAB_0866*	Probable molybdopterin-converting factor subunit 2 (MoaE)	0.5	-0.194	6	12
131	B1MCI9	*MAB_2881c*	Uncharacterized protein	0.196	-0.198	3	5
132	B1MJZ8	*MAB_4435*	Non-specific serine/threonine protein kinase	0.59	-0.207	12	33
133	B1MHY7	*purF*	Amidophosphoribosyltransferase	0.481	-0.209	20	31
134	B1MIY7	*MAB_4285*	Thioredoxin domain-containing protein	0.357	-0.211	3	15
135	B1MN46	*MAB_1673*	DUF559 domain-containing protein	0.239	-0.212	2	18
136	B1MLN4	*MAB_1375*	Probable sugar ABC transporter, ATP-binding protein SugC	0.368	-0.217	11	21
137	B1MIN3	*MAB_4181*	ATP-sulfurylase small subunit	0.243	-0.218	7	20
138	B1MC14	*MAB_2706c*	Putative transporter	0.302	-0.223	3	13
139	B1MBG5	*MAB_2505c*	Probable phosphoketolase	0.357	-0.227	8	47
140	B1MBY1	*MAB_2672c*	Quinolinate phosphoribosyltransferase [decarboxylating]	0.207	-0.228	15	17
141	B1MH70	*rplJ*	50S ribosomal protein L10	0.162	-0.229	11	17
142	B1MLR8	*MAB_1409c*	Putative drug antiporter protein	0.411	-0.231	3	13
143	B1ML09	*MAB_4585c*	Putative S-adenosyl-L-methionine-dependent methyltransferase MAB_4585c	0.194	-0.231	21	20
144	B1MP99	*MAB_2069*	Uncharacterized protein	0.307	-0.232	15	18
145	B1MJU1	*MAB_4377c*	Probable fatty-acid-CoA ligase	0.154	-0.239	7	36
146	B1MIY6	*MAB_4284c*	Uncharacterized protein	0.464	-0.239	4	8
147	B1MDT9	*MAB_3332c*	Uncharacterized protein	0.363	-0.242	9	11
148	B1MNW3	*MAB_1931c*	Conserved hypothetical integral membrane protein	0.287	-0.251	1	2
149	B1MCL6	*MAB_2908c*	2-dehydropantoate 2-reductase	0.151	-0.265	14	22
150	B1MP42	*MAB_2011*	Pyridoxal phosphate homeostasis protein	0.462	-0.265	22	19
151	B1MK14	*MAB_4452*	Putative transcriptional regulator, MerR family	0.411	-0.268	2	16
152	B1MCB9	*fmt*	Methionyl-tRNA formyltransferase	0.146	-0.274	12	16
153	B1MI56	*MAB_0782*	Uncharacterized protein	0.43	-0.276	12	9
154	B1ME59	*MAB_0020*	DUF3566 domain-containing protein	0.284	-0.281	12	11
155	B1MK15	*MAB_4453c*	YCII domain-containing protein	0.132	-0.283	1	5
156	B1MKH7	*MAB_1181c*	Putative lipoprotein LpqV	0.415	-0.298	3	6
157	B1MMK1	*MAB_4918c*	Pyruvate dehydrogenase E1 component alpha subunit	0.316	-0.304	2	21
158	B1MCY5	*MAB_3028*	RNA polymerase sigma factor	0.196	-0.311	12	25
159	B1MIN6	*MAB_4184c*	Superoxide dismutase [Cu-Zn]	0.452	-0.323	9	10
160	B1MFC2	*MAB_0222c*	Putative DNA-binding protein	0.27	-0.328	7	15
161	B1MGP4	*MAB_0479*	Putative regulatory protein, MarR	1.034	-0.334	4	11
162	B1MDV0	*MAB_3343*	ACT domain-containing protein	0.129	-0.336	8	11
163	B1MNA5	*MAB_1721*	Histidine kinase	0.626	-0.345	10	21
164	B1MMG7	*MAB_4884c*	Uncharacterized protein	0.206	-0.346	7	11
165	B1ME04	*MAB_3397*	HTH tetR-type domain-containing protein	0.445	-0.354	5	11
166	B1MD64	*rpsO*	30S ribosomal protein S15	0.807	-0.359	14	7
167	B1MMU6	*MAB_1573c*	Uncharacterized protein	0.464	-0.361	5	13
168	B1MGT9	*MAB_0524c*	Probable conserved lipoprotein LpqG	0.68	-0.362	3	12
169	B1MLF7	*MAB_1296*	Uncharacterized protein	0.361	-0.377	4	6
170	B1MBJ7	*MAB_2537c*	Putative pyruvate decarboxylase	0.491	-0.383	4	38
171	B1MHV5	*MAB_0680c*	Amidohydro_3 domain-containing protein	0.286	-0.384	44	38
172	B1MM33	*MAB_4750*	Putative short chain dehydrogenase/reductase	0.499	-0.387	1	15

Table S6. **Differential analysis of the *M. abscessus* S labeled proteome of samples pre-incubated with VM043 inhibitor followed by VM055*p* probe labeling vs. VM055*p* probe-labeled samples only**

				[VM043+VM055*p*] *vs* . VM055*p*			
	**Protein IDs**	**Gene names**	**Protein names**	**-LOG(p-value)**	**Fold Change (Log2)**	**peptides counts all**	**nb Tryptic Peptides**
173	B1MHQ3	*MAB_4060*	Putative short chain dehydrogenase/reductase	1.243	-0.392	10	15
174	B1MI00	*MAB_0726*	Rieske domain-containing protein	1.098	-0.406	4	22
175	B1MM40	*MAB_4757*	Putative transcriptional regulator, TetR family	0.318	-0.425	5	18
176	B1MHH7	*MAB_3984c*	Putative metal transporter ATPase	1.354	-0.429	31	32
177	B1MAE3	*MAB_p10*	Putative resolvase/invertase/recombinase	0.362	-0.436	6	15
178	B1MJ83	*MAB_0947c*	Putative luciferase	0.513	-0.437	6	21
179	B1MJB8	*MAB_0982c*	Putative acyl-CoA hydrolase/thioesterase	0.259	-0.44	4	10
180	B1MK42	*MAB_4480c*	Putative glycosyl transferase	1.86	-0.497	7	32
181	B1MKX2	*MAB_4548*	Probable O-methyltransferase	0.424	-0.502	10	16
182	B1MB28	*MAB_2368*	Uncharacterized protein	0.554	-0.511	2	14
183	B1MJS5	*MAB_4361*	Hypothetical fumarylacetoacetate hydrolase family	0.393	-0.517	15	16
184	B1MJB0	*MAB_0974*	Uncharacterized protein	0.416	-0.532	13	29
185	B1MF17	*MAB_3547*	Uncharacterized protein	0.737	-0.536	5	21
186	B1MET0	*MAB_3460c*	Uncharacterized protein	0.357	-0.594	4	16
187	B1MMX4	*MAB_1601c*	Putative monooxygenase	0.784	-0.599	11	26
188	B1MLP6	*MAB_1387*	Putative esterase/lipase/beta-lactamase	0.78	-0.63	5	28
189	B1MAH0	*MAB_2160c*	Putative lipoprotein LppK	0.598	-0.686	9	10
190	B1MKH2	*MAB_1176c*	Uncharacterized protein	0.714	-0.704	2	10
191	B1MIP4	*MAB_4192*	Uncharacterized protein	1.039	-0.713	9	13
192	B1MCJ4	*MAB_2886c*	Uncharacterized protein	0.346	-0.752	3	3
193	B1MFL8	*MAB_0319*	Nucleoid-associated protein MAB_0319	0.341	-0.789	2	4
194	B1MKV0	*MAB_4526*	Uncharacterized protein	0.387	-1.064	1	6
195	B1MB54	*MAB_2394*	Uncharacterized protein	1.201	-1.073	3	10
196	B1MEQ6	*MAB_3436*	non-specific serine/threonine protein kinase	1.169	-1.08	1	5
197	B1MFK1	*aqdB*	2-heptyl-3-hydroxy-4(1H)-quinolone synthase	1.411	-1.189	3	18
198	B1MCL9	*MAB_2912c*	Probable aldolase	1.319	-1.81	8	13

Table S7. **Cytotoxic activities of the β-lactone analogs towards Raw264.7 murine macrophage cells.** [a]

Compounds	CC_{50} (µg/mL) Raw264.7
**VM001 (C16:1 ω9)**	>125
**VM008 = *trans* -(R,R)-VM001**	>125
**VM009**	>125
**VM013 = *trans* -VM009**	>125
**VM020 = *cis* -VM009**	>125
**VM010**	>125
**VM017 = *trans* -VM010**	>125
**VM018= *cis* -VM010**	>125
**VM011**	>125
**VM022 = *trans* -VM011**	>125
**VM024 = *cis* -VM011**	>125
**VM012**	>125
**VM021 = *trans* -VM012**	>125
**VM023 = *cis* -VM012**	>125
**VM019 (*trans*)**	>125
**VM025**	>125
**VM026 = *trans* -VM025**	>125
**VM027 = *cis* -VM025**	>125
**VM028 (C18:1 ω9)**	>125
**VM029= *trans* -VM028**	>125
**VM030 = *cis* -VM028**	>125
**VM037**	>125
**VM036 = *trans* -VM037**	>125

Compounds	CC_{50} (µg/mL) Raw264.7
**VM039**	119±4.0
**VM038 = *trans* -VM039**	>125
**VM040 (*trans*)**	>125
**VM041 (*cis*)**	>125
**VM042**	>125
**VM043 = fluorinated VM038-039**	>125
**VM044 = fluorinated VM040-041**	>125
**VM045**	>125
**VM046 = fluorinated VM045**	>125
**VM047**	>125
**VM048**	>125
**VM056 = VM040 β-lactam**	30.6±1.1
**VM057 = trans-VM058 β-lactam**	52.7±1.1
**VM058 = β-lactam**	40.1±0.6
**ABP probes**	
**VM035*p*** (= **VM028** probe)	>125
**VM049*p*** (= **VM019** probe)	68.1±3.1
**VM050*p*** (= **VM019** probe)	44.1±2.4
**VM051*p*** (= **VM025** probe)	>125
**VM052*p*** (= **VM038, VM039, VM043** probe)	>125
**VM053*p*** (= **VM038-039, 043** probe)	>125
**VM054*p*** (= **VM040, VM041** probe)	>125
**VM055*p*** (= **VM038, VM039, VM043** probe)	>125

[a] Cytotoxic concentration of compound leading to 50% cell toxicity (CC_{50}) determined on Raw264.7 macrophages by the resazurin microtiter assay (REMA). All reported values are expressed as mean ± SD of three independent assays. In blue, are molecules tested in our previous study (see Santucci et al. ChemMedChem (2019) 14:349-358. https://doi.org/10.1002/cmdc.201800720**)**
